# The pitfalls of platform comparison: DNA copy number array technologies assessed

**DOI:** 10.1186/1471-2164-10-588

**Published:** 2009-12-08

**Authors:** Christina Curtis, Andy G Lynch, Mark J Dunning, Inmaculada Spiteri, John C Marioni, James Hadfield, Suet-Feung Chin, James D Brenton, Simon Tavaré, Carlos Caldas

**Affiliations:** 1 Department of Oncology, University of Cambridge, Addenbrooke's Hopsital, Hills Road, Cambridge, CB20XZ, UK; 2 Cancer Research UK Cambridge Research Institute, Li Ka Shing Centre, Robinson Way, Cambridge CB20RE, UK; 3 Department of Human Genetics, University of Chicago, 920 E 58th Street, Chicago, IL 60637, USA

## Abstract

**Background:**

The accurate and high resolution mapping of DNA copy number aberrations has become an important tool by which to gain insight into the mechanisms of tumourigenesis. There are various commercially available platforms for such studies, but there remains no general consensus as to the optimal platform. There have been several previous platform comparison studies, but they have either described older technologies, used less-complex samples, or have not addressed the issue of the inherent biases in such comparisons. Here we describe a systematic comparison of data from four leading microarray technologies (the Affymetrix Genome-wide SNP 5.0 array, Agilent High-Density CGH Human 244A array, Illumina HumanCNV370-Duo DNA Analysis BeadChip, and the Nimblegen 385 K oligonucleotide array). We compare samples derived from primary breast tumours and their corresponding matched normals, well-established cancer cell lines, and HapMap individuals. By careful consideration and avoidance of potential sources of bias, we aim to provide a fair assessment of platform performance.

**Results:**

By performing a theoretical assessment of the reproducibility, noise, and sensitivity of each platform, notable differences were revealed. Nimblegen exhibited between-replicate array variances an order of magnitude greater than the other three platforms, with Agilent slightly outperforming the others, and a comparison of self-self hybridizations revealed similar patterns. An assessment of the single probe power revealed that Agilent exhibits the highest sensitivity. Additionally, we performed an in-depth visual assessment of the ability of each platform to detect aberrations of varying sizes. As expected, all platforms were able to identify large aberrations in a robust manner. However, some focal amplifications and deletions were only detected in a subset of the platforms.

**Conclusion:**

Although there are substantial differences in the design, density, and number of replicate probes, the comparison indicates a generally high level of concordance between platforms, despite differences in the reproducibility, noise, and sensitivity. In general, Agilent tended to be the best aCGH platform and Affymetrix, the superior SNP-CGH platform, but for specific decisions the results described herein provide a guide for platform selection and study design, and the dataset a resource for more tailored comparisons.

## Background

The accurate and high-resolution mapping of DNA copy number aberrations (CNA) has become an important tool for biological and medical research. From understanding the extent of natural genetic variation [1], to associations with diseases such as HIV [2], to elucidating the mechanisms of tumourigenesis [3], such research is dependent on the quality of the data generated.

Numerous reports on the use and comparison of copy number profiling platforms have appeared [4-10] and more recently an approach to perform meta-analyses across such platforms has been described [11]. Early studies [12] suggested a high level of concordance between BAC-based aCGH and SNP-based platforms (Affymetrix 10 K array) in detecting CNA, but did not formally compare them. Greshock *et al*. [5] performed the first systematic comparison of multiple platforms on melanoma cell lines and found that a high level of sensitivity and specificity was observed for the Agilent 185 K arrays and that the increased probe density of Affymetrix arrays (100 K and 500 K) results in increased confidence in detection for these platforms. These results were echoed by Gunnarsson *et al*. [8] who also examined the performance of several older copy number profiling platforms (a 32 K BAC array, the Affymetrix 250 K SNP array, the Agilent 185 K oligonucleotide array, and the Illumina 317 K SNP) array in 10 chronic lymphocyte leukaemia (CLL) samples. They concluded that all platforms performed reasonably well at detecting large alterations, but that BAC probes were too large to detect small alterations. While Agilent offered the highest sensitivity, the increased density of SNP-CGH platforms (Affymetrix and Illumina) compensated for their increased technical variability, with Affymetrix detecting a higher degree of CNA compared to Illumina. A further aCGH study did not compare platforms, but did investigate the influence of cellularity on copy number detection [13] and concluded that modern high-resolution arrays could cope with high levels of contamination.

To attempt a fair and formal comparison of copy-number profiling platforms in a general setting is an almost futile exercise. Quantification of performance is difficult even with idealized data, and while measurements have been proposed such as the theoretical power to discover a single copy loss or gain [7], or the 'functional resolution' of the platform [6], these tend either to measure a very specific aspect of the platform, or appear flawed under close examination. Such idealized data are, in any case, difficult to obtain, as one has to ask what is fair in terms of numbers entering the experimental design. Should one Illumina array be compared to one Nimblegen array or should the two-channel Nimblegen array be compared to two arrays from the single colour technology? Should the two-colour platform be penalized by an inefficient design to allow easier comparison, or the SNP-based platform credited for the additional information that it brings? If, as often is the case, the main experimental constraint is financial, then comparing $1000 of one technology to $1000 of another technology would seem sensible. However, the relative costs of platforms will vary from laboratory to laboratory and with time, and such an approach would foist the authors' view of microarray economics on the reader.

Additionally, the results from such an exercise are only as good as the analysis methods used and in that regard one has two options, both flawed. Naturally, the platforms will require different pre-processing strategies, but if different methods of analysis are also used for segmentation, then the performance of the technology will be confounded with the adequacy of the algorithm. This then punishes newer technologies for which analytical methodologies are not yet mature. The alternative, to use a common approach for the analysis of all platforms, is undesirable firstly because that approach is likely to have been developed for one of the technologies and may thus introduce bias, and secondly because the deliberate use of a sub-optimal analysis does not provide useful information to inform decisions in the real world. Nonetheless, informative qualitative comparisons can be made without performing segmentation that illuminate the relative strengths and weaknesses of each platform. We acknowledge that some users will be primarily interested in a comparison based on using existing analytical tools, rather than concerning themselves with the potential of each platform, but that is not the purpose of this study.

This study differs from previous comparative assessments of copy number profiling platforms in that we have attempted to characterize the strengths and weaknesses of various platforms in as unbiased a fashion as possible by avoiding measures that cannot be fairly computed, highlighting areas of potential bias, and emphasizing a graphical assessment of performance that provides insight about the underlying technology as well as the specific platform. Inevitably, despite considerable effort, these comparisons will be shaped by our own prejudices concerning copy number analysis, but we have made the raw data available for others to draw their own conclusions.

Due to the speed of platform development, it is typical for a platform to be superseded by one with a greater number of features before comparisons involving it are published. The generation of platforms described here have not yet been the subject of an in-depth comparison, but have indeed already been superseded since this study was performed. Nonetheless, the underlying technologies are similar and a comparison is still informative. Implications for the new generations are discussed in the New Platforms section.

Herein we describe a comparison based on the analysis of two cell lines, six primary breast tumours, including matched normal samples, and two HapMap individuals. The SUM159 and MT3 cell lines and HapMap samples were selected based on the presence of known chromosomal aberrations, while the tumours are highly heterogeneous and hence present additional complexity for copy number analysis, not least with regard to their varying degrees of cellularity.

Here we present an analysis of probe coverage on each of the microarray platforms and a technical description of their reproducibility, sensitivity, and noise. We also provide an in-depth visual assessment of the ability of the different platforms to identify a range of sizes of copy number aberration. Lastly, we provide a publicly available dataset resulting from the processing of a range of samples (chosen to evaluate different abilities) on each platform. This information will allow interested parties to make decisions based on their own circumstances, preferences, and constraints.

## Results

### Theoretical and technical performance

#### Probe coverage and resolution

We present a summary of probe numbers in Table 1. Appreciation of the basic differences between the platforms is crucial for understanding the differences in performance. The Affymetrix platform has by far the most features, with the Illumina and Nimblegen arrays having a little under half of that number, and the Agilent array having markedly fewer still. More detailed summaries, including range of coverage and breakdown by chromosomal arm are presented in Additional File 1.

**Table 1:** Basic summary of platform contents

Chromosome	Affymetrix	Agilent	Illumina	Nimblegen
**1**	64442	17259	27151	30220

**2**	69304	17382	28903	32900

**3**	58067	14802	24393	27255

**4**	55531	12863	22136	25940

**5**	52788	12486	22016	24223

**6**	51362	12438	26824	23138

**7**	43909	12201	20022	20549

**8**	45407	10309	20369	19870

**9**	34991	8461	17551	15160

**10**	42890	10297	18063	17820

**11**	41597	10114	16916	17901

**12**	40517	10169	16965	17991

**13**	30495	7375	13134	13541

**14**	25712	7512	11140	12130

**15**	23131	7314	10540	10735

**16**	22875	5610	10454	10206

**17**	19375	6220	9990	10025

**18**	24091	5586	11407	10682

**19**	12122	4081	7251	6828

**20**	19498	4715	8659	8403

**21**	11510	3077	5982	4733

**22**	10590	3181	6209	4442

**X**	27536	10179	12556	19151

**Y**	996	1191	1412	1963

**Total**	828736	214822	370043	385806

For the probes used in this study (i.e., only 60-mer Agilent probes, and well-annotated Affymetrix probes), the number of features broken down by chromosome.

We choose not to present the theoretical functional resolution of these platforms as calculated by ResCalc [6] for three reasons, each of which is, in itself, revealing with regard to the inter-platform differences. Firstly, the results presented in Coe *et al*. [6] obscure a large degree of inter- and intra- chromosome variability. As a proportion of their total, Illumina have more probes on chromosome 6 than do the other platforms, with the result that even though there are more probes in total on the Nimblegen platform, for this particular chromosome Illumina have 16% more probes than Nimblegen. On chromosome 19, Affymetrix put a noticeably higher proportion of probes on the q arm than do Agilent, a situation that is reversed on chromosome 7.

The second problem of comparing by ResCalc is that the tool allows the platforms to define their own range of coverage from telomere to centromere. This makes it possible for a platform to improve its functional resolution by removing probes (essentially by dropping peripheral loosely spaced probes, while retaining the central tightly spaced ones), which is undesirable. To take an example, on arm 7p, in the core region covered by all of the platforms, Affymetrix average a probe every 3 to 4 Kb. However on the telomeric side of that core region, they have two probes covering 80 Kb. Undoubtedly the functional resolution as calculated by ResCalc would improve if such probes were removed (indeed, in this example, the removal of a single telomeric probe improves the reported functional resolution by 140 bases). Taking a more extreme example, the p arm of chromosome 9 has 13,643 probes on the Affymetrix platform and has a reported single probe functional resolution of 222,000 bases, but by removing 6 extreme telomeric probes and 166 extreme centromeric probes that are more sparsely positioned, we can improve the reported resolution to 8,900 bases. In general, the SNP-based platforms cover a wider region, with Nimblegen coming third and Agilent, in effect, often defining the core region of common coverage.

Finally, the hypothesis of uniform occurrence of CNA is doubtful and some of the platforms have been designed to provide non-uniform coverage by tiling more probes in known regions of variation (see Methods section for further details), or in areas where variation would be of particular interest. For example, Nimblegen have chosen, for the second generation of the product featured here, to switch from a uniform spacing along the genome to a 'designed' layout. This move would appear detrimental using tools such as ResCalc, but is clearly done for a purpose.

Reasons that one might adopt a non-uniform spacing include the desire to incorporate prior knowledge of genomic structure (e.g. to target CNVs, promoter regions, genes etc. and avoid repetitive elements), empirical evidence of probe performance from previous array designs, and lastly to achieve uniformity of probe performance. We show in the Methods section that there are a number of probe properties (most notably GC content) that affect the consistency of probe performance. These trends were visible in our data for all four platforms. There may, of course, be effects that are less visible, from these data, such as saturation levels and dynamic ranges. Naturally, increased probe coverage can address issues of variation, but technical biases will not be salved by increasing the number of probes.

#### Replicate probes

All of the platforms provide some replicate probes, by which we mean probes carrying the same sequence. For the SNP-CGH arrays, this is an integral aspect of the platforms and nearly all of the observations are actually averaged from replicate probes, 4 replicates for the Affymetrix SNP probes, and an average of 16 replicates for Illumina probes (although this ranges from 0 to over 40). With the Agilent and Nimblegen arrays, such probes are a rarity, and the majority of observations are based on only one probe. As such, for these two platforms, it makes sense to use the few probes with replicate information to characterize the performance of all observations. We can do this most informatively by calculating the variance of the replicate log-ratios between two samples.

Agilent provide, in addition to control probes, 916 60-mer probes for which there are three replicates. Nimblegen do not nominally provide any replication, but the coverage of the pseudoautosomal regions of the X and Y chromosomes results in 314 probes that are apparently replicated. However, we should note that these probes are treated as lying on different chromosomes, and thus if any within-chromosome normalization has taken place then their apparent reproducibility will be adversely affected. Neither Agilent nor Nimblegen show a strong association between the magnitude of log-ratio and variance of replicate observations (this is after all one of the reasons for analysing the log-ratio). To enable between-array comparisons, when we have resisted performing between-array normalizations, we summarize for the HapMap-HapMap comparisons the variance of replicate probes scaled by the mean difference in log-ratios observed in chromosomes X and 13, a difference that should be 1 for this comparison. Since this scaling does not share information between arrays, it is not a between-array normalization method.

For Agilent, the median variance of replicate probes is 0.042, 0.048, and 0.058 on three different arrays with third quartile values of 0.087, 0.111, and 0.120 respectively. In contrast, for Nimblegen, the median variance of replicate probes is 0.125, 0.142, and 0.144 with third quartile values of 0.309, 0.429, and 0.504, respectively. Thus Nimblegen exhibits 2-4 fold greater variability amongst replicate probes than Agilent. However, we note that the interpretation of the third quartile, in particular, should be tempered by our knowledge of the autocorrelation of probes along the genome.

Note that while the SNP-CGH platforms enable the quantification of allele-specific copy number [14-16], similar results cannot be obtained for the aCGH platforms. As such, we will focus strictly on the analysis of total copy number values. To quantify DNA abundance (or raw total copy number), the SNP-CGH platforms essentially sum the fluorescence intensities from the two alleles investigated for a given SNP. This involves, for each allele, averaging over the replicate probes and then summing.

Because of these replicate probes, for Affymetrix and Illumina estimating the variance of individual probes is of limited value, since the values of individual probes will not be reported. Yet, for Illumina we cannot provide a good estimate of the variance after averaging over the replicate probes and then summing over alleles because the covariance of the two channels is not estimable from the data provided by *BeadStudio *[17], but can be presumed not to be zero due to the array design.

#### Replicate arrays

After scaling within arrays to obtain a difference of 1 for the chromosome X to chromosome 13 comparison, the variances of three replicate HapMap-HapMap comparisons were calculated. As can be seen in Table 2, Nimblegen exhibits between replicate array variances an order of magnitude greater than the rest.

**Table 2:** Variance among three replicate HapMap-HapMap comparisons

Platform	1^st ^Quart	Median	Mean	3^rd ^Quart
**Affymetrix**	0.067	0.173	0.356	0.391

**Agilent**	0.046	0.122	0.304	0.284

**Illumina**	0.058	0.151	0.372	0.352

**Nimblegen**	1.21	3.03	5.65	6.47

After calculating the variance of each feature from three suitable scaled HapMap/HapMap comparisons, the mean, and quartile values of the variances are presented.

#### Self-self comparisons

The ability of a copy number profiling platform to detect aberrations is largely determined by the noise observed in the measurements from that platform. This is a measure not only of the variance of the noise (although this is important), but also the kurtosis of the noise (i.e., if the noise is relatively heavy tailed, then more false calls will be made) and the independence of neighbouring probes. Not only are there known autocorrelation effects along the genome [18], possibly driven or exacerbated by autocorrelation in the quality of probe design caused by regions of high GC content or highly repetitive elements, but if probes are too close then they may compete to register the same DNA fragments. In such a case, the lack of independence of measurements from the probes would detract from the benefits of having improved probe density.

The ideal test for such a comparison would be a set of log-ratios generated from two replicate normal samples, as any departure from a log-ratio of 0 for these platforms must be noise and can be easily quantified. Since for two platforms, one of the pooled normal samples intended for this task was of lower quality, instead we again use chromosome 13 from a comparison of the two HapMap samples. Not only does this have no known changes, but adds the benefit that again we can scale our observations so that the difference in log-ratios between chromosomes 13 and X is a standard 1.

We summarize the noise by four measures in Table 3: the variance (after scaling as described), the autocorrelation of measurements at lag 1 along the chromosome, the percentage of observations beyond two standard deviations, and the percentage of observations beyond three standard deviations. The first measure will ideally be low and gives an indication of the noise-to-signal ratio, the second gives a measure of the independence of neighbouring probes, while the third and fourth give an idea of the false calling rates that might arise.

**Table 3:** Characteristics of a surrogate self-self hybridization

	Variance (scaled)	Autocorrelation	% z > 2	% z > 3
Affymetrix	0.33, 0.34, 0.29	0.040, 0.039, 0.036	4.5, 4.8, 4.7	1.3, 1.4, 1.3

Agilent	0.22, 0.24, 0.21	-0.001, 0.027, 0.019	4.4, 4.6, 4.9	0.5, 0.8, 0.7

Illumina	0.28, 0.36, 0.31	0.086, 0.066, 0.076	5.2, 5.3, 5.3	1.4, 1.4, 1.2

Nimblegen	0.81, 0.85, 0.60	0.009, 0.035, 0.026	4.3, 4.8, 4.5	0.5, 0.5, 0.5

For chromosome 13 of a HapMap/HapMap comparison (a surrogate self-self hybridization), presented are the variance, autocorrelation, and percentage of observations beyond two or three standard deviations.

These results indicate that Nimbelgen is noisy, exhibiting poor variance (2-4 fold greater than the other platforms). Additionally, Illumina has relatively poor autocorrelation for its probe density and has more outliers at a standard deviation of 2. Further, both SNP-CGH platforms have more outliers beyond a standard deviation of 3, which may be related to the autocorrelation. It is worth noting that Agilent has relatively few probes on chromosome 13 (see Table 1, Additional File 1), but based on other performance measures, this is unlikely to influence significantly its superior performance.

#### Male-Female comparisons based on X and Y chromosomes

**Figure 1:**
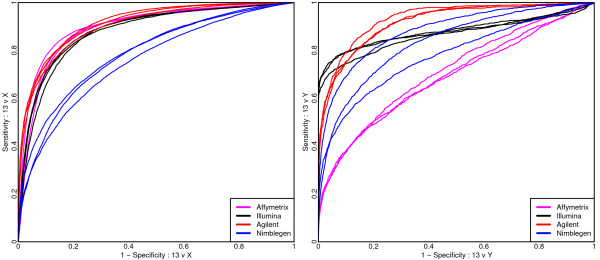
**For a comparison of the HapMap samples ROC curves are presented to assess the performance of a single probe/probe-set for distinguishing the log-ratios associated with differing copy numbers from the log-ratios of chromosome 13 where copy-numbers should agree**. Note the contrast from the left-hand panel, where the performances of Affymetrix and Agilent are indistinguishable, and the right hand panel, where the performance of Affymetrix has substantially declined.

Since the two HapMap samples consist of a male (NA10851) and a female (NA15510), but for the autosomal chromosomes exhibit few copy number differences, we can use these samples to investigate the ability of a single probe on these platforms to distinguish between the diploid state and an altered copy number state due to regions of physical loss. We compare the log-ratios arising from chromosome 13 with those arising from chromosome X in order to test the ability to detect a 2:1 copy number alteration, and also with those arising from chromosome Y in order to test the ability to detect a 1:0 copy number alteration. The single-probe abilities of the four platforms are depicted in Figure 1.

For distinguishing between sites where both samples have two copies and sites where one sample has two copies while the other has one (13 versus X), Affymetrix and Agilent marginally outperform Illumina, while Nimblegen performs noticeably worse. In contrast, when distinguishing between sites where both samples have two copies and sites where one sample has no copies while the other has one (chromosome 13 versus Y), Agilent generally exhibits the highest sensitivity, although Illumina outperforms Agilent if very high specificity is sought. These are followed in performance by Nimblegen, with Affymetrix performing considerably worse.

Notably, the Affymetrix Human Mapping 100 K, 500 K, and SNP5 platforms include chromosome X SNPs but no chromosome Y or mitochondrial SNPs. With the SNP5 platform, copy number non-polymorphic (CN probes) were introduced and for the Y chromosome there are 996 such probes with sufficient genomic information (1994 in total) all of which map outside the pseudoautosomal region. As such, for the SNP5 platform, the Y chromosome is not representative of other chromosomes in that it does not include any SNP probes and contains 0.1% of all probes on the platform. The lack of SNP probes is one possible explanation for the poor discrimination of a single copy loss on the Y chromosome. As noted in the Methods section, the CN probes are generally unreplicated and while few in number, the actual number of probes is on par with the other platforms.

### Qualitative assessment of copy number aberration detection

The platforms investigated in this study differ substantially in their design, the number of probes, and their experimental utility. To obtain an overview of platform performance, the ability to detect several types of common chromosomal changes was assessed. In particular, the following alterations were considered based on raw copy number changes: whole chromosome gains or losses, chromosome arm gains or losses, high amplitude focal amplifications as well as subchromosomal gains and losses, small regions of gain or loss as exemplified by normal copy number variation.

#### i.) Whole chromosome gains or losses

**Figure 2:**
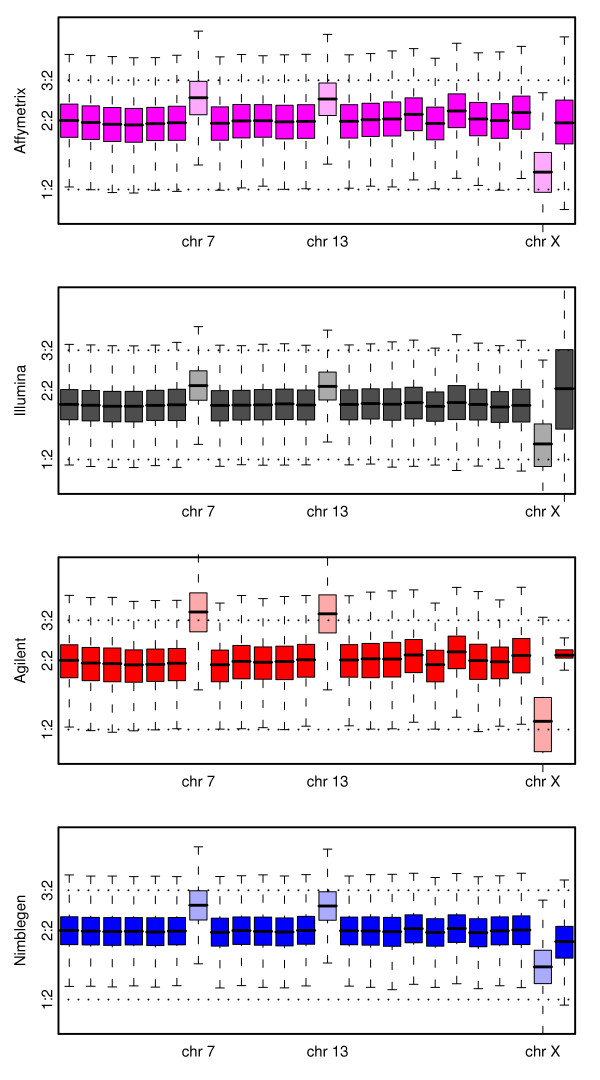
**Showing, for a comparison of the MT3 cell-line to a pooled normal reference, a boxplot of the log-ratios from each platform broken down by chromosome**. Also indicated are theoretical markers for a single copy gain and a single copy loss. The three chromosomes with known aberrant copy number are indicated.

This simple type of genomic aberration allows for examination of consistency at the level of probe log-ratioss (or potentially segmented means) along the whole chromosome. Note that this is similar to the comparison of the HapMap samples in the male-female comparison. Here we use the MT3 cell-line, which is known to have single-copy gains of chromosomes 7 and 13, and a single copy loss of chromosome X. As would be expected, all four platforms can identify whole-chromosome events (Figure 2), but there are differences in the abilities to quantify the change and also in the discrimination of different copy number states that will be influential for the classification of smaller regions. Agilent performs best on both of these measures. Nimblegen includes probes targeting the pseudoautosomal region, which explains the apparent departure from zero for chromosome Y.

Also of note is the performance in terms of Y chromosome detection and the effect of normalization on the Illumina array. The performance of Illumina in detecting the absence of the Y chromosome in females is of concern. It is not unreasonable that what would ideally be an estimate of log_2_(0/0) should be unstable (although due to non-specific binding the extremes of this instability will not be observed). If the observed values are indicative of any bias in the probe design, then the apparently strong performance of Illumina in the chromosome Y versus chromosome 13 comparison may have been misleading.

#### ii.) Chromosome arm gains or losses

**Figure 3:**
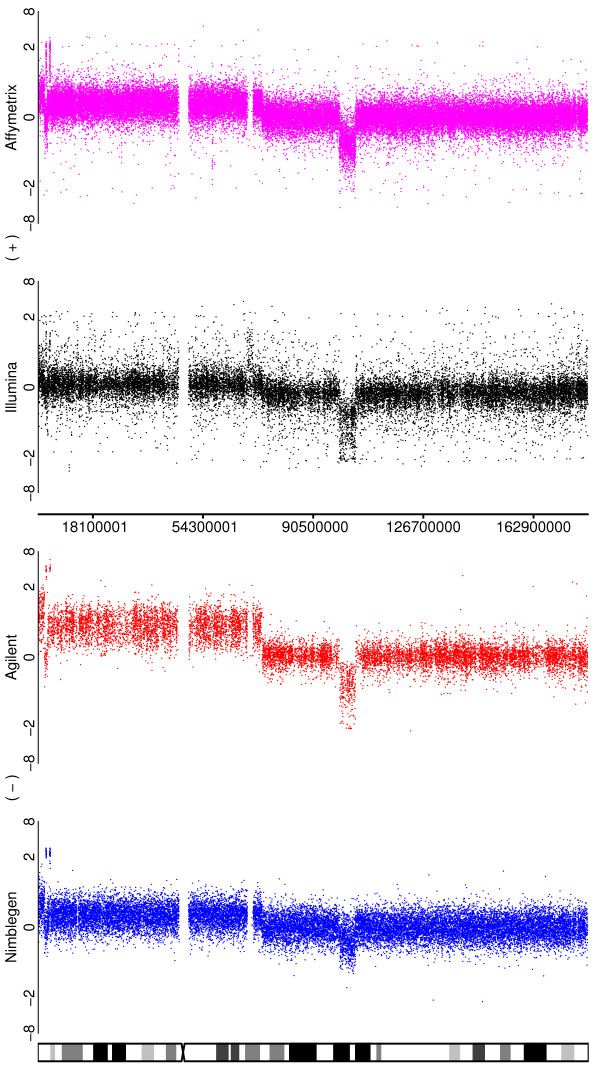
**Illustrating the ability of the platforms to detect the duplication of a chromosomal arm**. Depicted are the log-ratios for a comparison of the SUM159 cell line to a pooled normal reference for chromosome 5. In addition to a number of smaller aberrations, there is a duplication of the p arm of the chromosome for this sample.

We illustrate the ability of the platforms to detect a gain on a single arm of chromosome 5 in the SUM159 cell-line where, in addition to other variations, the 5p arm has an extra copy. Figure 3 illustrates the performance of the platforms for this chromosome. All of the platforms are able to detect the alteration, manifested as an upward deflection, but the clarity of signal is greatest for Agilent, followed by Affymetrix, Illumina and Nimblegen. This region is depicted in greater detail in Additional File 2. Of note is the duplication visible only in Illumina, at about 70 Mb into the chromosome. This is an area of known intra-chromosomal segmental duplication [2] and the other platforms place few probes in this region, as it is difficult to tile in these regions.

#### iii.) High amplitude focal amplifications and subchromosomal gains and losses

**Figure 4:**
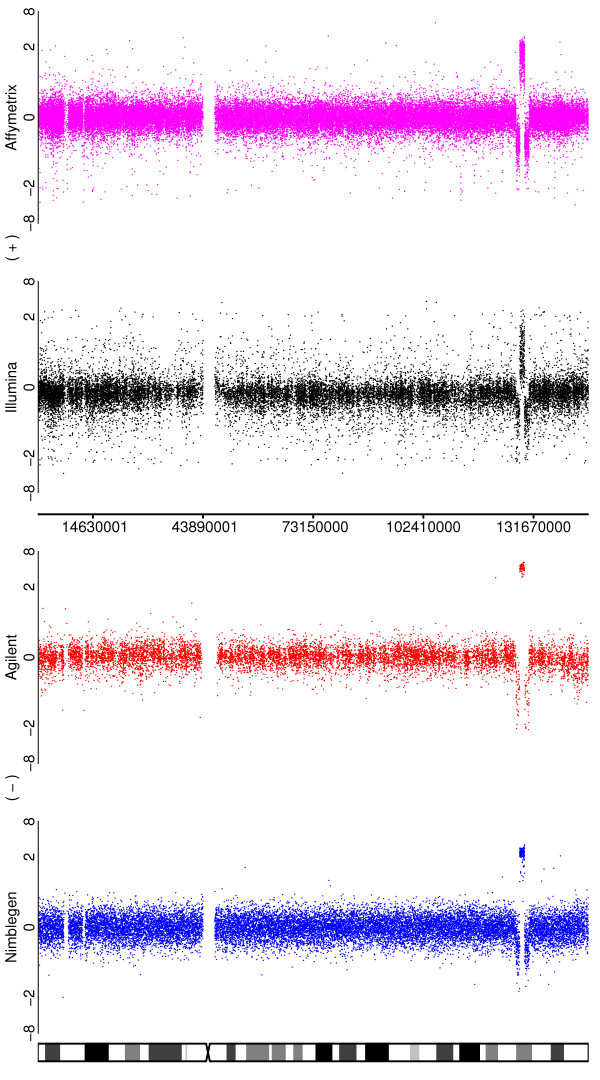
**Illustrating the ability of the platforms to detect high amplitude focal amplifications and other subchromosomal events**. Depicted are the log-ratios for a comparison of the SUM159 cell line to a pooled normal reference for chromosome 8. A deletion, duplication, deletion aberrations pattern is clearly visible for all four platforms in the region of x = 130 Mb.

These smaller variants are relatively complex aberrations and test the abilities of the platforms to determine breakpoints accurately. These types of alterations would also allow for the easiest assessment of segmentation algorithms, if such a task were desired. Three examples occur on chromosome 5 of the SUM159 cell-line (Figure 3). The most obvious alteration (a deletion at approximately 100 Mb) is clearly observed in all four platforms, although again the difference is less obvious for Nimblegen. The second aberration, a complex change towards the telomere of arm 5p, is also seen by all four platforms, but the clarity of the pattern is variable. Once again, Agilent is generally clearest, but the two amplified regions are seen more clearly by Nimblegen than by the two SNP arrays, although they would still be detected by those platforms. The deletions follow the usual order of being the most clear for Agilent > Affymetrix > Illumina > Nimblegen. The third much smaller change is most obvious for Affymetrix at about 55 Mb and is just barely detectable with Illumina, being so narrow as to fall between probes for Agilent and Nimblegen. A similar pattern is seen for the change at approximately 130 Mb on chromosome 8 for the SUM159 cell-line (Figure 4). Again, Agilent and Affymetrix do generally best, but Nimblegen does a much better job of identifying the amplifications than it does for the neighbouring deletions.

#### iv.) Small regions of gain/loss as exemplified by copy number variation between normal HapMap individuals

A total of 79 sites of copy number variation have been identified between the two HapMap individuals assayed in this study using an older technology, namely a custom whole-genome tiling path array developed at the Wellcome Trust Sanger Institute [19]. These variants were validated across multiple hybridizations and also via PCR. For a full list of locations see Additional File 3. Examination with these higher resolution technologies suggests that some of the sites actually form one larger variant, but we shall treat them as separate sites for this analysis. Many of the sites showed no sign of variation with any of the platforms, and concordance amongst platforms was high. Due to the nature of these small changes, it is not uncommon for a platform simply to have no probes in the region of interest. This varies between platforms, with probe density being influential, but not the only factor.

The 79 sites were assessed by eye to see if they provided evidence of variation (the plots of all these regions are available in Additional File 4). Rating each CNV as clear, tentative, absent, or not covered, we summarize the results in Table 4. Naturally, there is an element of subjectivity in this type of assessment, but the overall picture is clear. Affymetrix and Agilent identify the greatest number of variants, but Agilent fails to cover a fair number (18 out of 79).

**Table 4:** CNVs observed between two HapMap samples

Platform	Clear	Tentative	Not Covered
Affymetrix	20	14	10

Agilent	19	16	18

Illumina	8	21	5

Nimblegen	9	13	14

Detection of reported germline CNVs between two HapMap samples across each platform, as adjudged from the plots in Additional File 4 by three analysts. Differences in probe density prohibited the blinding of analysts, but each platform was scored independent of the others. Indicated are the number of CNVs that are clearly apparent, the number for which there may be some evidence, which are labelled tentative, and those which are not covered. Naturally, there is a fourth category (not shown), which includes regions that are covered, but appear not to be CNVs.

Notably, since some platforms (both Affymetrix and Illumina) have been designed to cover known CNVs and to target 'unSNPable' regions of the genome with copy-number non-polymorphic probes, this rate will by misleading if one is interested in identifying novel CNVs. Nimblegen has more probes than Agilent, and a similar number to Illumina, but does not attempt to target known interesting regions with this version of the array. Thus Nimblegen may well do relatively better with novel sites. That said, the evidence here is that even if novel sites have coverage, the platform may struggle to identify them as CNVs. Illumina cover more of the regions than do Affymetrix, but do not provide the clarity of change over these small intervals.

**Figure 5:**
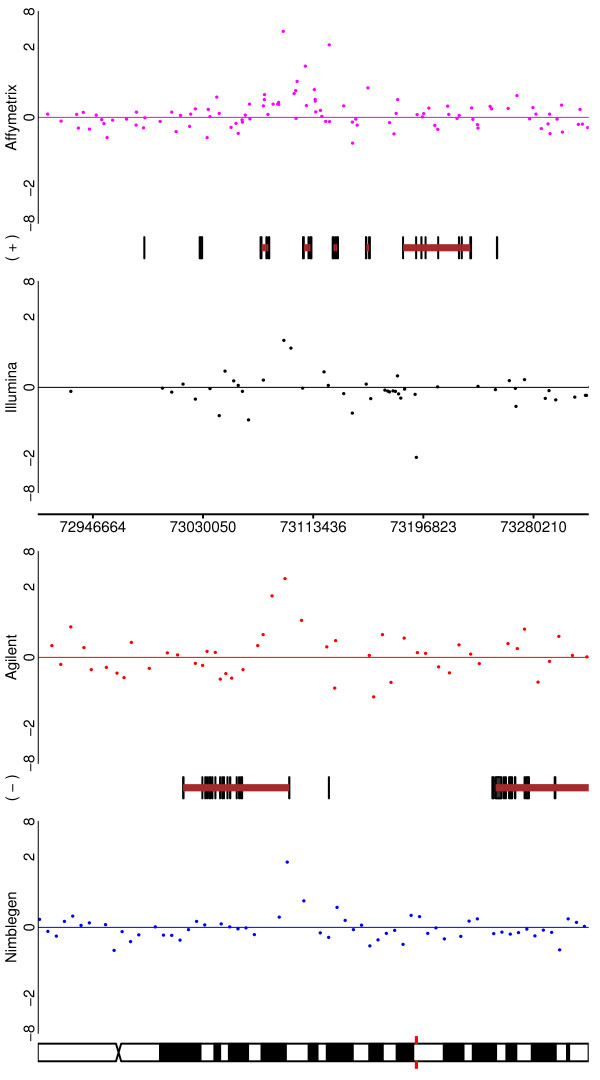
**For one of the HapMap - HapMap CNVs (CNV58 from Additional File **3), **depicted are the performances of all four platforms**. The change is visible in each case, but with differing degrees of clarity.

**Figure 6:**
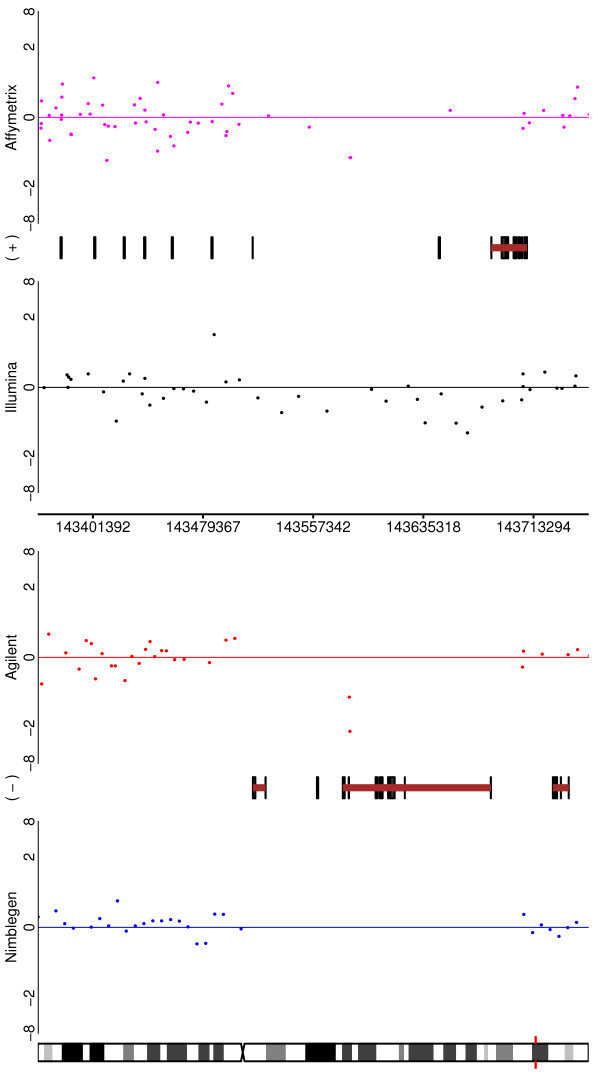
**For one of the HapMap - HapMap CNVs (CNV38 from Additional File **3), **depicted are the performances of all four platforms**. In this case the variant is not obvious (or even apparent) in three of the platforms due to poor coverage of the region. Nimblegen has no coverage, and Agilent and Affymetrix have relatively low coverage. However, these last two platforms do show the copy number variation with what probes they have. Illumina is less convincing on a probe-by-probe basis, but successfully demonstrates the CNV through sheer number of probes in the region.

Two CNV regions are shown in Figures 5 and 6. In Figure 5, CNV #58 is depicted and one can see that all of the platforms would identify it (with Affymetrix perhaps being the least clear). The 'typical' CNV #38 is depicted in Figure 6. Here, three of the platforms greatly reduce their coverage in the region of interest (Nimblegen being absent altogether), while Illumina exhibits good coverage. Despite this, the Affymetrix and Agilent probes that are in the region are quite clear, whilst Illumina is only convincing through weight of numbers.

### Detection of characterized copy number aberrations

**Figure 7:**
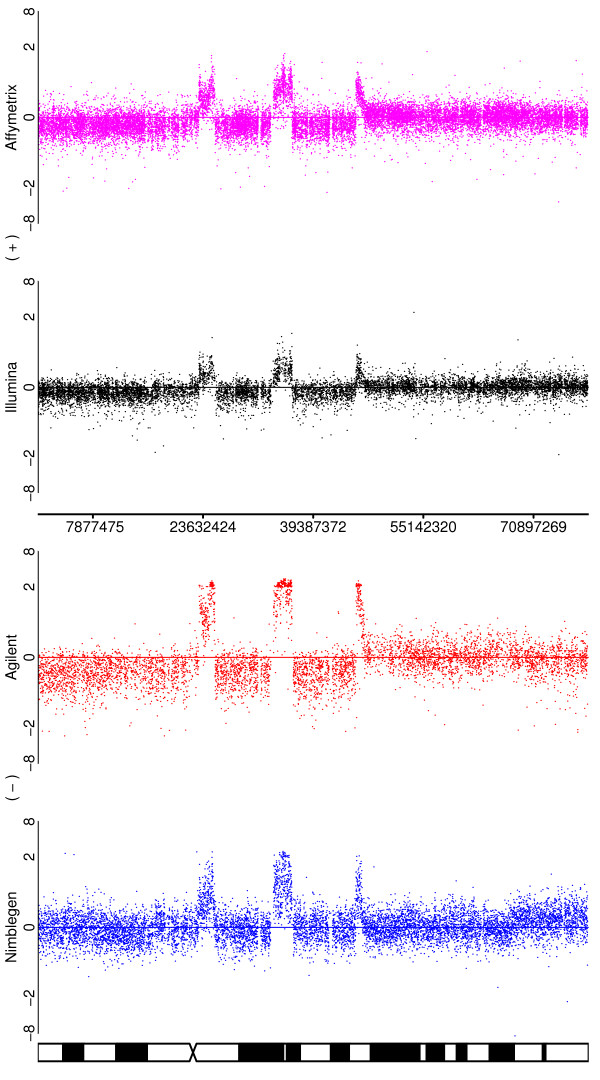
**Depicting Tumour 7214, Chromosome 17 for the four platforms (genes not shown)**.

**Figure 8:**
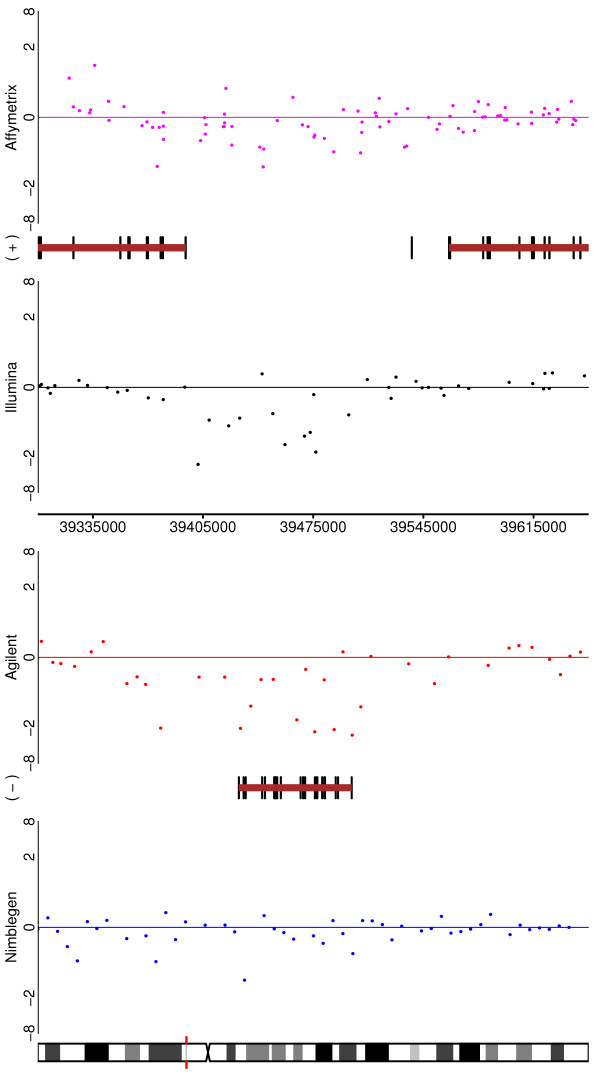
**Depicting, for Tumour 7207, the area around the ADAM3A gene for the four platforms**.

**Figure 9:**
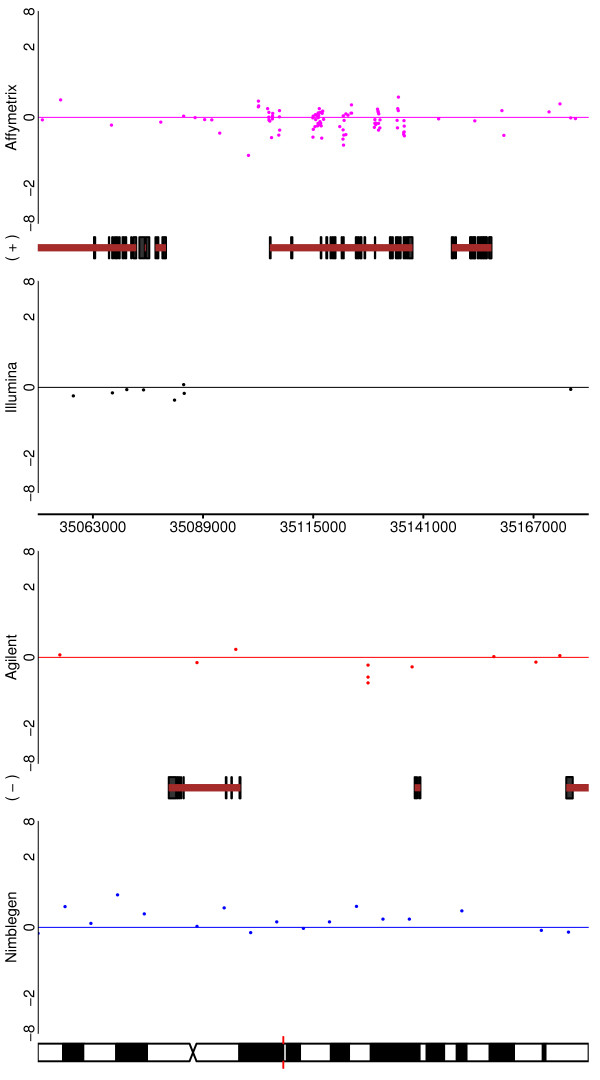
**Depicting, for Tumour 7207, the area around the ERBB2 gene for the four platforms**. Note the poor coverage of the Illumina platform.

We address other measures of performance by making use of aberrations that have previously been reported to occur in the cell lines or have been broadly described to manifest in breast cancer. An examination of the six tumour samples (Table 5) reveals that there is little difference in the ability of the platforms to spot the large aberrations associated with cancer, with the exception that changes are harder to spot in Nimblegen that with the other platforms. The tumours themselves differ substantially, with T2704, T2706 and T2707 exhibiting far fewer aberrations, although we note that this may be a reflection of the sample's cellularity. Figures 7, 8, and 9 highlight some of the aberrations observed in these tumours. For example, Figure 7 depicts Chromosome 17 for Tumour 7214 on all four platforms. Figure 8 reveals for Tumour 7207, the area surrounding the ADAM3A gene and Figure 9 the ERBB2 gene is shown. While none of the tumours here exhibits amplification of ERBB2, it is surprising to note how poorly represented this frequently amplified cancer gene is on the Illumina platform, although coverage is greater in the latest generation of the array.

**Table 5:** Detection of anticipated aberrations across platforms for the 6 tumour samples

	T1975	T2701	T2704	T2706	T2707	T2714
Gain 8p	possibly 8q (all)	also 8q (all)	none	none	none	none, but gain on 8q (all but Nimb)

Gain 1q	all	all		all	none	all but Nimb

Loss 16q	partial (all)	and gain 16p (all)	none	and gain 16p (all)	none	none

Amp 8q24	all	all	none	none	none	all but Nimb

Amp 11q13	none	all	none	none	none	none

Amp 17q12	none	none	none	none	none	all

Amp 20q13	all	Affy and Agil	none	none	none	none

Del 13q14	all	all	none	none	none	none

Del 9p21	all but Nimb	all	none	none	none	none

Del 17p13	none	all	all	none	none	all

The detection of anticipated aberrations in each of the 6 tumour samples is reported for each platform.

### Cellularity

The data set we present allows for the realistic comparison of platforms when considering copy-number changes in tumours. Tumour samples are often affected by stromal contamination [13] and to represent this, not only do we present 6 tumour samples of varying degrees of cellularity (see Additional File 5 for cellularity and clinical information for all samples), but a number of samples with simulated stromal contamination. Essentially, two of the tumour samples were diluted with their respective matched normal samples (7206: 30% tumour, 70% normal; 7207: 50% tumour, 50% normal) and two cancer cell lines were similarly treated (MT3 and SUM159: 30% tumour, 70% normal 7214).

**Figure 10:**
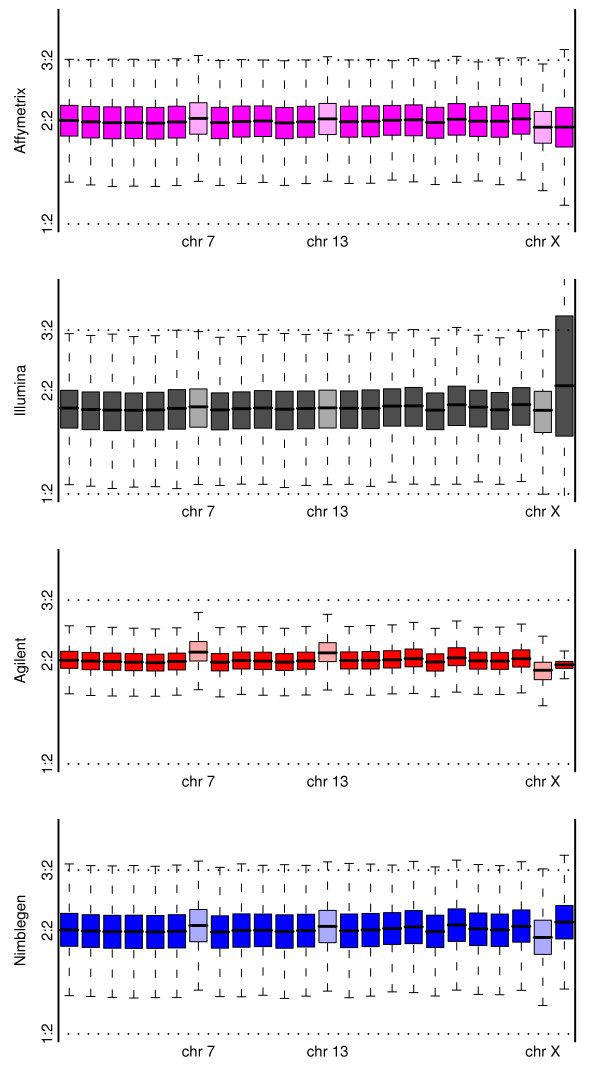
**For comparison with figure 2**: Depicting, for a dilution of the MT3 cell-line, compared to a pooled normal reference, a boxplot of the log-ratios from each platform broken down by chromosome. Also indicated are theoretical markers for a single copy gain and a single copy loss at this dilution level. The three chromosomes with known aberrant copy number are indicated.

We again consider the MT3 cell-line, this time in dilution, to see whether the anticipated copy number aberrations are visible (details of the expected copy number alterations for the cell-lines are given in Additional File 6). In Figure 10, equivalent to Figure 2 but for simulated 70% stromal contamination, the benefits of direct competitive hybridization are seen. The two CGH platforms provide much clearer evidence of copy number differences between the chromosomes (as might be anticipated following previous studies [13]), and of the two, Agilent outperforms Nimblegen. It is not unreasonable that a direct comparison is better able to detect small changes such as those anticipated here. Note that as in Figure 2, no allowance has been made for probes targeting pseudoautosomal regions, which may explain the odd behaviour of the Y chromosome.

**Figure 11:**
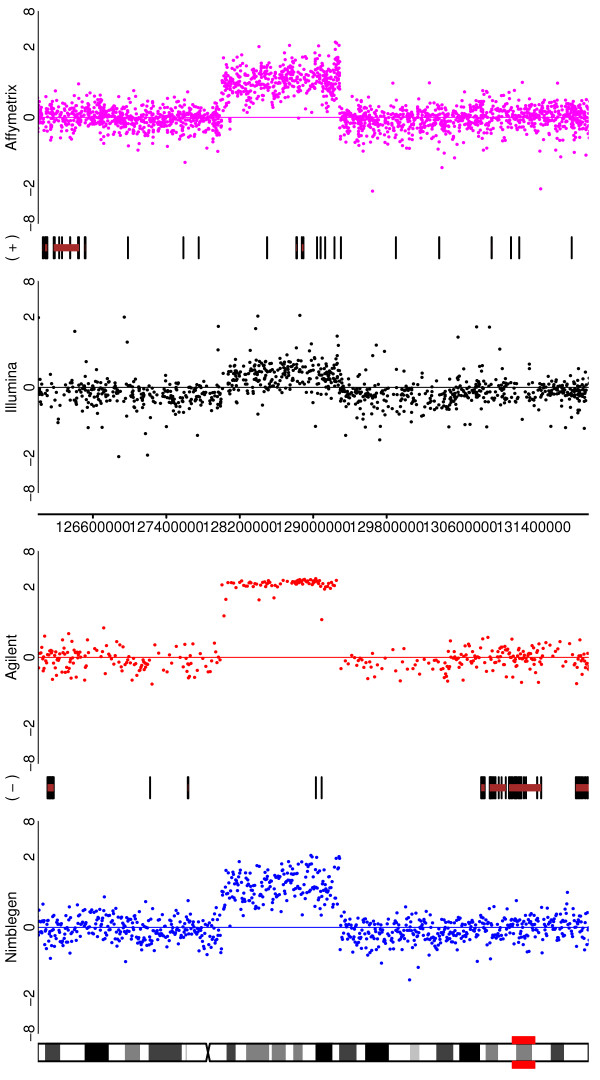
**Depicting, for a dilution of SUM159, the 8q region for the four platforms**.

Figure 11 illustrates a zoomed-in region of chromosome 8q for a SUM159 dilution, similar to Figure 4 for the undiluted samples. As expected, all of the platforms exhibit some signal attenuation, but each is still able to detect the amplification. Notably, Agilent is clearly the least affected and in fact robustly detects the alteration at nearly the same level as the undiluted case, attesting to the sensitivity of this platform. In contrast, all of the platforms struggled to detect a moderate loss in the same sample.

## Discussion

### Discussion of results

The ability of a platform to detect a particular aberration is a function of the distribution of probes in that region and the reliability of those probes. Of the two SNP-based platforms, there is little difference in terms of quality of individual probes, but those on the Affymetrix arrays are more numerous. That said, Illumina's strategy for locating probes means that there are locations where this platform offers greater coverage (cf. the known CNVs and the MHC-similar region 5q13, consistent with Illumina's stated design intent which also sees a greater focus on SNPs near RefSeq genes than does Affymetrix) but also some (such as the ERBB2 region) where they are lacking. The coverage of smaller features such as CNVs and genes is an important consideration in the choosing of a copy-number platform, as broadly speaking all of the platforms examined can identify large deletions and duplications.

A curiosity is that Illumina fails to identify robustly the chromosome 13 arm gain in the MT3 cell lines, suggesting an issue with the normalization applied by *BeadStudio*, but the main concern is the Nimblegen platform, which fails to spot some large aberrations in tumours T7195 and T7214. Of the two standard arrayCGH platforms, Agilent's performance is clearly superior. Not only is the Agilent data of high-enough quality to call aberrations from fewer probes than the other platforms, but also the ability of the Agilent platform to quantify aberrations appears to be superior. All of the platforms suffer from variation induced by probe design, related either to probe length, GC content or other aspects. Additionally, the quality of SNP and CGH probes on the Affymetrix and Illumina platforms may not be equivalent. Thus when choosing a platform one must consider not only the probe coverage in regions of interest, but also the quality of those probes.

### Explanation of cellularity findings

**Figure 12:**
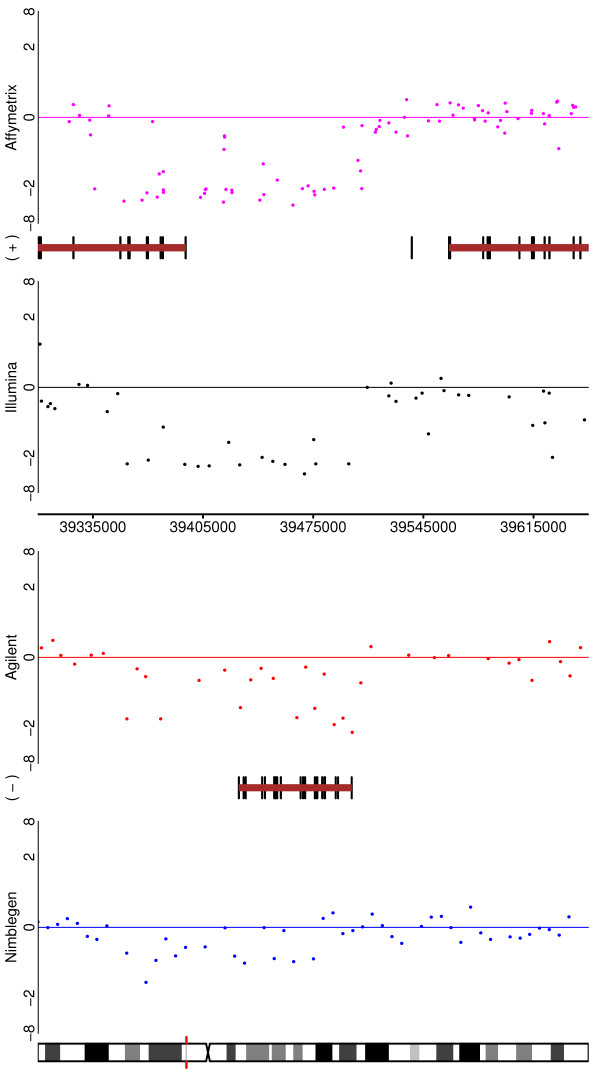
**Depicting, for a dilution of Tumour 7207, the area around the ADAM3A gene for the four platforms**.

The comparison for the diluted tumours is more complicated due to their pre-existing stromal contamination and the fact that aberrations of these tumours have not previously been well documented. Inspection of aberrations in the dilution of Tumour 7207 revealed one curiosity. Figure 12 depicts the area around the ADAM3A gene for each of the four platforms as in Figure 8, but here for a dilution (50%). Surprisingly, we observe that all platforms more robustly detect the loss in the dilution hybridization. We note that this sample had the lowest cellularity (40%) of all the tumours assayed and that many common aberrations were not observed in this sample. This raises the possibility that the normal sample might actually represent a preplasia and brings into question the composition of the tumour. Indeed, subsequent expert histopathological examination of this sample revealed that the tumour section was likely comprised of inflammatory infliltrate rather than invasive tumour cells. Upon examination of the matched normal sample for another tumour (7214) it was noted that the tissue contains substantial ductal carcinoma *in situ*. Despite the observation that this sample was used for dilution of the SUM159 cell line, this should not affect the previous discussion of the results since the 8q change was specific to that cell line and not observed in tumour 7214. It is noteworthy that examination of the array results prompted these findings, as this highlights the utility of microarray-based copy number assessment to detect subtleties in sample composition.

### Chemistry

In comparing microarray technologies, it is also important to keep in mind some of the more subtle differences between them in terms of the protocols, chemistry, and detection methods. For example, while both Affymetrix and Illumina are SNP-based copy number profiling platforms, there are important differences in their chemistries. The Affymetrix GenomeWide SNP 5.0 whole genome genotyping assay (as well as the newer SNP 6.0 array and older generations of this platform, namely the 10 K - 500 K arrays) all employ a complexity reduction procedure similar to that first described for representational oligonucleotide microarray analysis (ROMA) [20] in order to increase the signal-to-noise ratio. Essentially, the DNA is digested with the restriction enzymes *Nsp*I and *Sty*I, ligated to adaptors that recognize the cohesive four base-pair overhangs, and amplified using a universal primer that recognizes the adaptor sequence. The amplified DNA is subsequently fragmented, labelled, and hybridized to the oligonucleotide array. While the amplification of only the smaller restriction fragments improves the signal-to-noise ratio, these values still remain below that observed for BAC arrays, and the complexity reduction can potentially lead to the differential representation of certain genome regions and hence false positives. Also, since individuals vary in their restriction digestion profiles, certain probe ratio values may depend on differences in restriction fragment size rather that actual copy number variation [21].

In contrast, the Illumina whole genome genotyping protocol for the 370 HapMap Duo bead array (Infinium II technology) involves an isothermal genome amplification step (non-PCR based), fragmentation, hybridization to an oligonucleotide bead array, SNP detection based on a single-base extension reaction (SBE) on a single bead type with differentially-labelled terminators, and signal amplification. Thus the detection step, for the Illumina Infinium II assay is based on an enzymatic discrimination step (SBE for Infinium II, allele-specific extensions for Infinium I) rather than by hybridization as for Affymetrix. Illumina claims that the isothermal amplification step does not result in the preferential amplification of one allele [22].

### New Platforms

All of the manufacturers now offer products with more features than those compared in this report: the Affymetrix GenomeWide SNP 6.0 array, the Illumina 1 M-Duo array, the Nimblegen Ultra-High Density CGH array with 2.1 million features, and the Agilent Human CGH 1 × 1 M array. All but the Affymetrix chip come available with fewer features but multiple arrays on the chip (the Illumina platform starting with 2 arrays on the chip); the ability to run multiple samples in parallel is of great potential value for sensitive experiments. Also worthy of note is that the Nimblegen and Agilent platforms offer full customization of content, while Illumina offer limited customization.

As the coverage of platforms increases, many of the subtleties that we have observed will have decreased impact on the conclusions. The Illumina coverage of ERBB2, for example, is satisfactory in the latest generation of chip. It remains to be seen whether the manufacturers have been able to maintain probe quality in the next generation of products. We have already commented that the second generation of the Nimblegen platform featured here has seen a revision of probes to improve performance.

**Figure 13:**
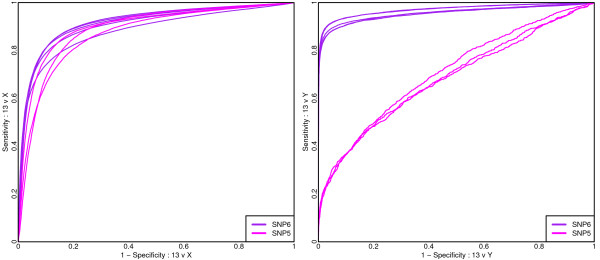
**For comparison with Figure 3**. Here a comparison of the Affymetrix SNP5 and SNP6 platforms are shown. ROC curves are presented to assess the performance of a single probe/probe-set for distinguishing the log-ratios associated with differing copy numbers from the log-ratios of chromosome 13 (where copy-numbers should agree) for the HapMap pair of samples. For SNP6 five replicate HapMap/HapMap (NA15510 vs NA10851) comparisons are shown using raw data available from the Affymetrix X chromosome titration study.

The other disappointing performance we have witnessed was that of the Affymetrix SNP5 platform for the Y chromosome. The newer Affymetrix SNP6 platform contains nearly 10 times as many Y chromosome probes, including approximately 900 SNP probes (recall that SNP5 contained only non-polymorphic probes for the Y chromosome). Of the 997 SNP5 Y chromosome probes, 127 (12.74%) are retained on SNP6. Hence SNP5 probes makeup only 1.34% of the total SNP6 Y chromosome repertoire. Using a publicly available Affymetrix SNP6 HapMap X chromosome titration data set [23], we compare the sensitivity and specificity of the SNP5 and SNP6 platforms in Figure 13. The SNP6 platform performs similarly to SNP5 in the detection of a 2:1 copy number alteration, whereas for a 1:0 alteration the improvement is striking.

### Alternative analysis methods

It should be noted that we have made use of manufacturer-provided tools, where available, for pre-processing this dataset. This was intentional, as the choice of optimal tools is platform-specific, especially since older platforms will likely benefit from more mature analysis tools. For example, we employed the *BeadStudio *software to summarize the Illumina data as this is manufacturer-supplied. Likewise, Nimblegen supplied NimbleScan pre-processed data. In contrast, Affymetrix do not offer comprehensive support for copy number analysis of the GenomeWide SNP5.0 platform so we employed the open source *aroma.affymetrix *[24] software, one of the few tools for pre-processing this relatively new SNP-CGH platform. Similarly, as Agilent do not provide free software for pre-processing of their aCGH data, standard open source methods were employed for this well-established platform. Although beyond the scope of this study, it is of interest to compare alternate pre-processing (and segmentation) methods for each of these platforms as this could influence the results obtained. In particular, the consideration of Illumina SNP data at the bead level could yield considerable improvements since this would enable calculation of the within-bead-type correlation or covariance [17] as well as more detailed quality assessment. For example, we were able to identify spatial artefacts in the Illumina data in this study (Additional File 7) that would benefit from the BASH tool [25] implemented in *beadarray *[26] although this method has not yet been fully implemented for Illumina genotyping data. Additionally, there were some issues with small, localized failures of image registration that could only be addressed by bead-level pre-processing and that would undoubtedly improve the quality of the Illumina results if addressed successfully.

## Conclusion

It is important to stress that there is no straightforward way to compare fairly copy number profiling platforms in a general manner. As such, the results presented here describe the detection and qualitative comparison of raw copy number alterations across four platforms in tumour samples for which both matched and pooled normal DNA were available and in two established cell-lines. Copy number variation in normal HapMap individuals was also compared using the same platforms. Whilst we have sought to avoid analytical techniques that are objective, but that we deem undesirable for the stated reasons, we have focused on graphical comparisons that are, of course, prone to subjectivity. In any case, the competing platforms have different merits, and users need to make subjective decisions based on their individual requirements.

Although there are substantial differences in the design, density, and replicate structure of the probes, the comparison indicates a generally high level of concordance between platforms. As expected, all platforms were able to detect large aberrations in a robust manner. However, some focal amplifications and deletions were only detected on a subset of the platforms. In particular, Nimblegen failed to detect numerous aberrations that were clear in the other platforms even when probes were tiled in the region of interest. This finding is perhaps not surprising given that this platform exhibits 2-4 fold greater variance amongst replicate probes and variances an order of magnitude greater for replicate array comparisons. In general, for the aCGH-based platform Agilent was the best performer and for the SNP-CGH platform, Affymetrix tended to outperform Illumina. An added bonus is that both Affymetrix and Agilent require only 0.5 μg DNA as starting material, thus removing this consideration from the platform decision. Another potential consideration is the quality or source of DNA (e.g. the use of paraffin-embedded samples [13]), for which some platforms may be more forgiving.

Our study differs from previously published ones in that we employ primary breast tumour samples rather then cell-lines. As noted previously, this introduces additional complexity due to the possibility of stromal contamination [13]. Further to this, we have also made use of cell-line dilutions and well-characterized HapMap samples to evaluate copy number alterations across platforms. That we also conclude that Agilent performs best on a single-probe comparison is of interest because we are comparing newer platforms, yet we must keep in mind that the performance of platforms from generation to generation cannot be assumed to be constant.

In the new generation of arrays, Agilent have addressed their primary weakness by increasing probe coverage. Similarly, Nimblegen have modified their probe design in order to improve performance. Both Affymetrix and Illumina have increased probe coverage with Affymetrix introducing slight modifications to probe design. If Agilent have maintained probe quality, it seems likely they will remain the leader, but Nimblegen may close the observed gap. For the SNP-CGH arrays, it seems likely that Affymetrix will continue to perform well. The availability of data from these new platforms will enable comparisons with previous generations of arrays for the purposes of meta-analyses and the like.

Obtaining reproducible, high-resolution copy number data with high sensitivity and few false positives is the gold-standard objective for any such study. However, there are always tradeoffs and a critical assessment of the goals of the project and underpinning biological questions can help select the most suitable platform. For example, breakpoint precision, which is dependent on the local resolution, is likely more critical for mapping novel tumour suppressor genes and oncogenes, than for a more general survey of aberrations where little follow-up validation is planned. Additional considerations that might influence the choice of platform include probe coverage (whether gene-centric or uniformly spaced, targeting non-coding elements) and the ability to assay genotypic information, and hence allele-specific copy number and copy neutral loss of heterozygosity. If matched normal samples are available, it might be advantageous to exploit the direct comparison design offered by dual-channel technologies. In large-scale studies, it may also be useful to validate the higher-density SNP-CGH findings using a subset of samples on a lower-density, but more sensitive, platform. The results described here provide a guide for platform selection and study design, and the dataset a resource for more tailored comparisons.

## Methods

### Study design

The state of the art in terms of commercially available platforms for genome-wide CNA is constantly evolving. Here, four leading platforms were compared: the Affymetrix Genome-Wide Human SNP Array 5.0, the Agilent High-Density CGH Human 244A array, the Illumina HumanCNV370-Duo DNA Analysis BeadChip, and the Nimblegen 385 K oligonucleotide array. Several important differences exist between these platforms. Beyond the fact that the Affymetrix and Illumina employ a single-channel hybridization scheme, whereas Agilent and Nimblegen use a dual-channel competitive hybridization protocol, the former are also SNP-CGH platforms, while the latter are not. Other differences in the design of these platforms include the probe-length and probe-density. Whereas Nimblegen employs 45-mer to 85-mer probes, Agilent 60-mer probes and Illumina 50-mer probes, Affymetrix probes are considerably shorter at 25 nucleotides. In terms of probe-density, the Affymetrix SNP 5.0 array contains 500,568 SNP probes and an additional 420,000 non-polymorphic probes to facilitate studies of germline copy number variation in association studies. The Agilent 244A array contains computationally pre-selected probes that have been experimentally optimized for genomic hybridization with a bias towards gene-rich regions. The Illumina CNV370 array includes 318,000 SNP markers plus 52,000 markers targeting 14,000 additional CNV regions. Lastly, the Nimblegen 385 K array contains 386,165 isothermal oligonucleotide probes with relatively uniform genome coverage. Due to resource availability, two of the platforms (Agilent and Illumina) were processed in-house, whereas for the other platforms the samples were hybridized at a commercial vendor (Affymetrix and Nimblegen).

### Sample Choice

Two representative cell lines (MT3 and SUM159) were selected based on the presence of known chromosomal aberrations so as to provide markers of a platform's performance. The MT3 colorectal cell line contains a single copy gain of chromosome 7 and isochromosome 13 [27,28]. The SUM159 breast carcinoma cell line is also reported to have several notable changes including a loss on chromosome 5q and gain on chromosome 8q24 [27,28]. The ability of the various platforms to detect known focal amplifications was assessed using a panel of six tumour samples. To assess the effect of using a matched normal as compared to a pooled normal as the reference against tumour samples, a single replicate was included for each matched normal sample. Additionally, to ascertain the effect of cellular heterogeneity due to stromal contamination in detecting CNA, several dilution experiments were included for the two cell lines and two of the tumours such that a mixture of either 30% cell line (tumour) with 70% normal or a 1:1 ratio was hybridized to the arrays.

Two 'normal' samples (NA15510 and NA10851) from the Human HapMap study [29] were also selected to assess the detection of naturally occurring regions of copy number variation, as they have been characterized extensively [30-33] and are recommended for use as a standard control in all studies [21]. Further, they provide an example in which gross abnormalities are not expected. Moreover, sample NA10851 is male, allowing for a controlled assessment of the platforms performances by examination of the sex chromosomes in the HapMap comparisons.

Each sample was hybridized to the single-channel platforms in triplicate, with the exception of the pooled normal samples, which were performed in duplicate. For the dual-channel platforms, tumours and cell-lines were hybridized against pooled normal tissue in duplicate, and the tumours were additionally hybridized against matched normal tissue. The HapMap samples were hybridized against each other in duplicate, as was a pool vs. pool hybridization. Additionally dye-swap hybridizations were performed for the HapMap samples and the MT3 cell-line. In all platforms, save for Nimblegen, some hybridizations were discarded under quality control procedures. Nimblegen only returned data for hybridizations that satisfied their quality control criteria.

### Patient material and cell lines

Samples were collected in the year 2000 at Addenbrooke's Hospital, Cambridge, UK from female patients ranging from 41 to 83 years old. These samples correspond to fresh frozen biopsies and surgical resection samples and the resultant fresh breast tissue was stored in the Addenbrooke's Hospital tumour bank. Ethical consent was obtained for all patient samples. The MT3 cell line (with a single X chromosome, suggesting male origin) was obtained from its originators [34], and has been shown to be identical to the colorectal cancer cell line LS174T based on SNP analysis [35]. This cell line exhibits an almost normal karyotype, apart from trisomy 7 and isochromosme 13. The SUM159 breast carcinoma cell line was obtained from the originators [27]. SUM159 is a hyperdiploid cell line with a modal chromosome number of 47 and nine structural translocations.

All human samples used for this analysis were obtained with informed consent from patients and the study was performed with appropriate REC and NHS R&D approval.

### DNA extraction and purification

Tumour DNA was extracted from 25 × 10 um sections manually using the DNAeasy kit (Qiagen, Valencia, CA). Matched normal DNA was obtained by homogenizing tissue in 180 μl of ATL buffer with Precellys, followed by extraction with the DNAeasy kit (Qiagen). For the cell lines, DNA was extracted using the proteinase-SDS method [36].

### Array hybridization and analysis

#### Affymetrix

**Figure 14:**
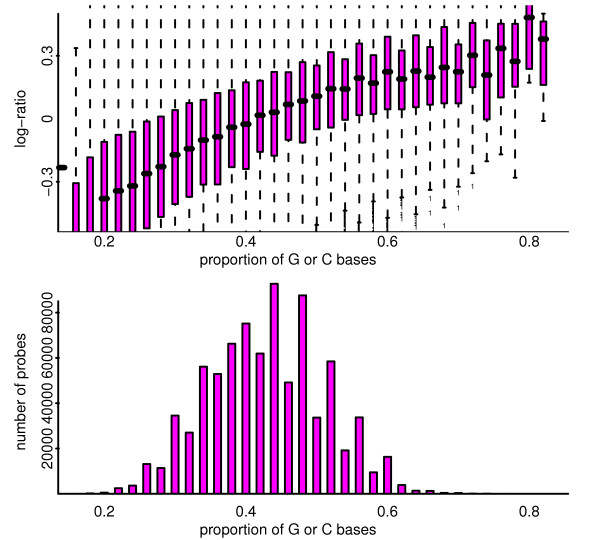
**For a pool-pool log-ratio comparison from the Affymetrix platform, depicted are the effect and distribution of probe GC content**. Top: Showing the effect of GC content on log-ratio. Bottom: Showing the distribution of probe GC content.

Genotyping using Affymetrix Genome-Wide SNP 5.0 arrays was performed according to standard Affymetrix protocol (at AROS, Denmark) using 0.5 μg DNA. Log_2 _signal intensities were measured from the raw data derived from the scanned image. Signal intensities were corrected for allelic crosstalk and offset for SNP probes and for offset for copy number non-polymorphic probes (CN probes), and probe signals were rescaled so that all probes (excluding those on the X & Y chromosomes) have the same average across arrays [24]. Probe-level data were summarized, wherein probe signals for SNP probes were averaged across replicates and summarized between alleles; probe signals for CN probes were unchanged since they are generally-unreplicated single-probe units. Signal intensities were shifted by 300 units to avoid negative signals that might result following calibration for allelic crosstalk and due to random errors around zero. Fragment-level normalization was then performed to correct for systematic differences in the amplification efficiency of PCR on fragments of varying length and deviations from the 50/50 NspI/StyI mixture. This procedure is a multi-chip method, which estimates the baseline effects as effects observed in a robust average across all arrays and hence should cause systematic effects across arrays to cancel out. Raw total copy number estimates (on the log_2 _scale) were obtained by comparing the summarized and normalized intensity values for a given cell line or tumour sample to the corresponding intensities from the reference array. Although 920,928 SNP probes and non-polymorphic copy number probes are present on the array, due to incomplete information concerning a subset of the probes, 828,737 are analysed in this study. As noted above, Affymetrix data were corrected for fragment length effects as it has been noted that fragment length influences probe intensity, as does GC content [14]. Further, as for gene expression arrays, the sequence effect is position-dependent for Affymetrix SNP chips and importantly, fairly large differences in intensities can be observed for the different alleles as a result of sequence alone [37]. The effects of GC content are illustrated in Figure 14. The Affymetrix dataset consists of 50 arrays, as detailed in Additional File 8.

#### Agilent

The Agilent platform used is the Agilent Human Genome CGH Microarray Kit 244A This platform uses just under 240,000 unique 60-mer oligonucleotide probes across the genome, with tighter coverage in the region of RefSeq genes, and claims to emphasize other interesting genomic features (miRNAs, promoters, etc) also. Experiments were performed in-house using 0.5 μg of DNA and either the Agilent labelling kit or the Enzo labeling kit. After hybridization and washing, the slides were scanned on an Agilent Microarray Scanner and captured images were analysed with Feature Extraction Software v 9.1.

Arrays were considered for analysis using a guideline DLRS threshold of 0.3. This is higher than the threshold advised by Agilent, but that threshold does not allow for the large aberrations associated with tumour samples that will inevitably inflate this score. Where necessary (if multiple repeat hybridizations for a sample failed to bring the score down), hybridizations with a higher score were used to fill in gaps in the experimental design if they were judged to be acceptable. Similarly, some samples were not used despite passing the threshold if they were clearly problematic from a visual inspection. This resulted in 40 arrays remaining in the study (see Additional File 9). The Enzo protocol used for Agilent saw generally lower scores for this quality control measure, but saw an increase in the influence of probe length on the results from the array.

**Figure 15:**
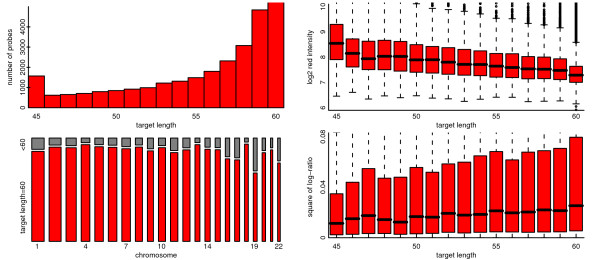
**For a pool-pool hybridization from the Agilent platform depicted are the distribution and effect of probe target length**. Top left: depicted are numbers of probes apparently targeting sequences of different lengths, with modes at 60 and 45. Bottom left: Shown are the proportions of probes, for each autosomal chromosome, that have target length 60; a proportion that is lowest for chromosome 19. Note that the width of the bar is proportional to the total number of probes on that array. Right: Two boxplots depict the associations between probe target length and intensity, and probe target length and log-ratio. 60 mer target sequence lengths are associated with lower intensities and greater variance of log-ratio.

Based on the annotation information included in the Agilent output files, only 215,002 out of 238,162 (90%) of Agilent probes appear to be targeting 60 mer sequences (Figure 15), with the rest being shorter (as short as 45 mers in some cases). There is a marked relationship between observed intensities and target sequence lengths for the platform, with the probes targeting longer sequences generally generating lower intensities. This feeds through to having greater variance of log-ratio for the longer sequences. The effects are often more marked than in the example shown, and as a result the non-60 mer targets have been dropped from the analysis. Intensities were background corrected via the *normexp *function in the *limma *package [38,39] and loess normalized to return log-ratios. No between-array normalization was performed; where between-array comparisons are made, we specify the steps taken to scale the arrays in question.

#### Illumina

**Figure 16:**
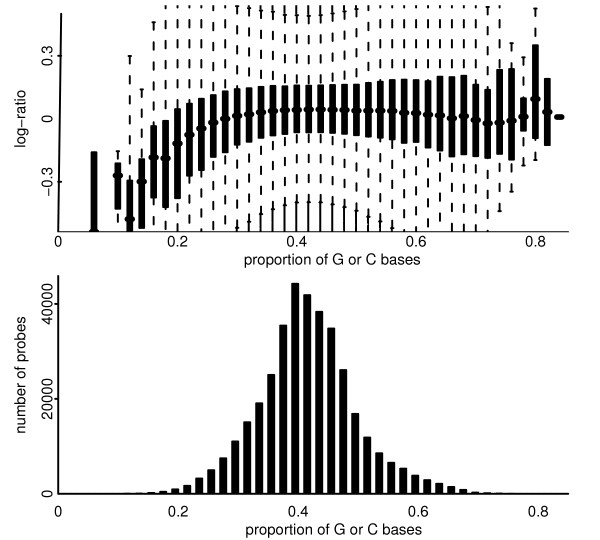
**For a pool-pool log-ratio comparison from the Illumina platform, depicted are the effect and distribution of probe GC content**. Top: Showing the effect of GC content on log-ratio. Bottom: Showing the distribution of probe GC content.

Genotyping using Illumina CNV370-duo arrays was performed in-house according to the standard protocol with 0.75 μg DNA. Log_2 _signal intensities were obtained using Illumina's *BeadStudio *software (ver.3). Following averaging of the per-allele replicates (16 on average), the A and B alleles are summarized, scaled and rotated to reduce allelic crosstalk on a per-array basis. Within *BeadStudio*, a paired analysis was performed for all contrasts of interest. The resultant log2 ratios were then exported from the *BeadStudio *software to facilitate comparisons between platforms. Since the log-ratios were not centred around zero for the tumour samples relative to a pooled normal (while this was the case for the tumour samples relative to the matched normals), both subsets of assays were normalized under the assumption that median copy number is 2 and the median log2 ratio is zero. The effects of the GC proportion on log-ratios are shown in Figure 16. The Illumina dataset consists of 48 arrays, as detailed in Additional File 10.

#### Nimblegen

**Figure 17:**
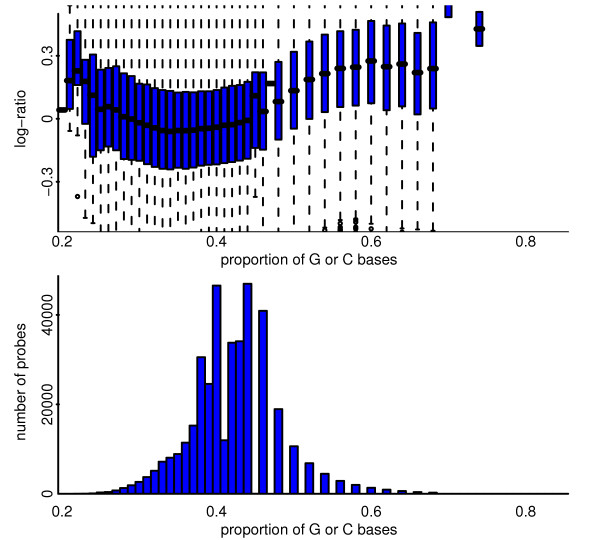
**For a pool-pool hybridization from the Nimblegen platform depicted are the effect and distribution of probe GC content**. Top: Showing the effect of GC content on log-ratio. Bottom: Showing the distribution of probe GC content. The median GC content is 0.42 (IQR 0.38 to 0.44), but is noticeably lower for chromosomes 4 and 13, and noticeably higher for chromosomes 19 and 22. Naturally there is a high spatial auotcorrelation of probe GC content along the genome.

The Nimblegen platform used here is the HG18 CGH 385 K WG Tiling v1.0 array. This platform makes use of 385,000 oligonucleotide probes of length 50 mer to 75 mer. These probes are spaced along the genome with reasonable uniformity, unlike for the v2.0 array that followed, where probe locations were subject to more involved design. The experiments were performed by Nimblegen according to their standard protocol using 2.5 μg DNA, and were analysed using the processed and normalized data files supplied by Nimblegen. Nimblegen also report the lengths of the individual probes and the proportion of bases that are either G or C. The effects of the GC proportion are shown in Figure 17; there is a strong association between probe length and GC content, but still some evidence that probe length is influential even after GC content is considered (not shown). The Nimblegen dataset consists of 44 arrays, the details of which are in Additional File 11.

All analysis was performed in the R statistical programming language [40]. The arrays described in this study been deposited in the Gene Expression Omnibus [41] with accession number GSE16400. Plots of each chromosome for each sample and platform are available in Additional File 12 and 13.

### Plotting conventions

Where we have plotted relative copy number (log-ratio) against genomic location, we have used the best quality example for each platform. This may be the cause of a slight bias, as different platforms may have different numbers of replicates from which to choose, but since we are looking to establish the potential of the platforms, it is the appropriate approach. Replicates have not been averaged, as between-array standardization has not been performed, save for the case of CNV comparisons, where three replicates of each platform are comparable without standardization and the improved signal-to-noise allows for acceptable clarity with so few probes. Genomic location was taken from the supplied annotations for Agilent and Nimblegen, and likewise for Affymetrix and Illumina. For the different platforms the genomic location represents different properties (probe start, SNP location etc). However, on the scale on which we are plotting, this does not affect interpretation.

The scale for the y-axis for the plots is linear from -2 to 2, and linear also outside this region, but at a different rate. Most values lie in the -2 to 2 range and this needs to be our focus, but it is also important that we can depict more extreme cases. The discontinuity in the first derivative of the scale allows us to achieve this. As well as the log-ratios for the four platforms, we depict genes lying on the plus and minus strands, and a guide to the section of chromosome being illustrated. The information for these additional items was obtained from the *GenomeGraphs *[42] package in Bioconductor.

Where CNV locations are plotted, the nominal location lies within the middle two-fifths of the x-axis, allowing for easy use of the provided axis coordinates to identify that region. Throughout the paper, we adopt a convention of colour-coding for platforms: Affymetrix are represented by magenta, Agilent by red, Illumina by black, and Nimblegen by blue.

## Competing interests

CNC owns Illumina shares.

## Authors' contributions

CNC co-conceived the analysis strategy, analysed the Affymetrix and Illumina data, and drafted the manuscript. AGL co-conceived the analysis strategy, analysed the Agilent and Nimblegen data, and drafted the manuscript. MJD helped process and analyse the Illumina data. IS participated in the study design and prepared samples. JCM helped design the study and provided CNV expertise. JH supervised the Agilent and Illumina array hybridizations. SFC supervised the sample preparation. JB participated in the study design. ST contributed to manuscript preparation. CC conceived the study, participated in its design, and contributed to manuscript preparation. All authors have read and approved the manuscript.

## Supplementary Material

Additional file 1 **Detailed description of platform features**. An extension of Table 1. In order the columns represent i) The numbers and percentages of features by chromosome for each of the four technologies (cols B-I), ii) Within chromosomes, the numbers and percentages of features within the p arm (cols J-Q), iii) the core region in the p arm covered by all four platforms (cols R, S), iv) for each technology, in that core region, the probe density, the number of extra probes towards the telomere, the extra distance covered towards the telomere, the probe density in the extra region towards the telomere, the number of extra probes towards the centromere, the extra distance covered towards the centromere, and the probe density in the extra region towards the centromere (Affymetrix cols T-Z, Agilent cols AA-AG, Illumina cols AH-AM, Nimblegen cols AN-AU), v) as per ii) to iv), but for the q arm. Click here for file

Additional file 2 **Details of SUM159**. Plots detailing the loss-gain-loss aberration on chromosome 8 of SUM159. Click here for file

Additional file 3 **A list of validated CNV sites for the HapMap/HapMap comparison**. The full list of the 79 sites of copy number difference between HapMap samples NA15510 and NA10851 [1,30]. Click here for file

Additional file 4 **Plots of the 79 CNV sites**. Plots of the 79 sites of copy number difference between HapMap samples NA15510 and NA10851 (as listed in Additional File 3). Click here for file

Additional file 5 **Pathological and clinical summaries for the 6 tumours**. Details of the sample identity and cellularity composition as well as the construction of the pooled normal sample and the dilutions. Click here for file

Additional file 6 **Anticipated aberrations and known copy number changes in various samples**. A list of known copy number changes for the cell-lines (SUM159, MT3, NA15510 and NA10851) and anticipated copy number changes for the tumours. Click here for file

Additional file 7 **Image plots of BASH processed Illumina data**. False-colour image representation of six different raw images from the Illumina dataset that had significant spatial artefacts as identified using the BASH method from the *beadarray *Bioconductor package. As *BeadStudio *does not take spatial information into account during pre-processing, the resultant summarized values may be compromised in the presence of such artefacts. Click here for file

Additional file 8 ***Experimental design for the Affymetrix platform***. Targets file detailing the sample hybridized to each Affymetrix array. Click here for file

Additional file 9 **Experimental design for the Agilent platform**. Targets file detailing the samples hybridized to each Agilent array. Click here for file

Additional file 10 **Experimental design for the Illumina platform**. Targets file detailing the sample hybridized to each Illumina array. Click here for file

Additional file 11 **Experimental design for the Nimblegen platform**. Targets file detailing the samples hybridized to each Nimblegen array. Click here for file

Additional file 12 **All sample/chromosome plots for the tumours**. Zip folder containing PNGs of all whole-chromosome plots for the tumours. Click here for file

Additional file 13 **All sample/chromosome plots for the cell-lines**. Zip folder containing PNGs of all whole-chromosome plots for the cell-lines. Click here for file
