# Supplementary figures and images for: The pitfalls of platform comparison: DNA copy number array technologies assessed (part 1 of 3)
Source: BMC Genomics. 2009 Dec 8;10:588. doi: 10.1186/1471-2164-10-588 (PMC2797821; doi:10.1186/1471-2164-10-588)

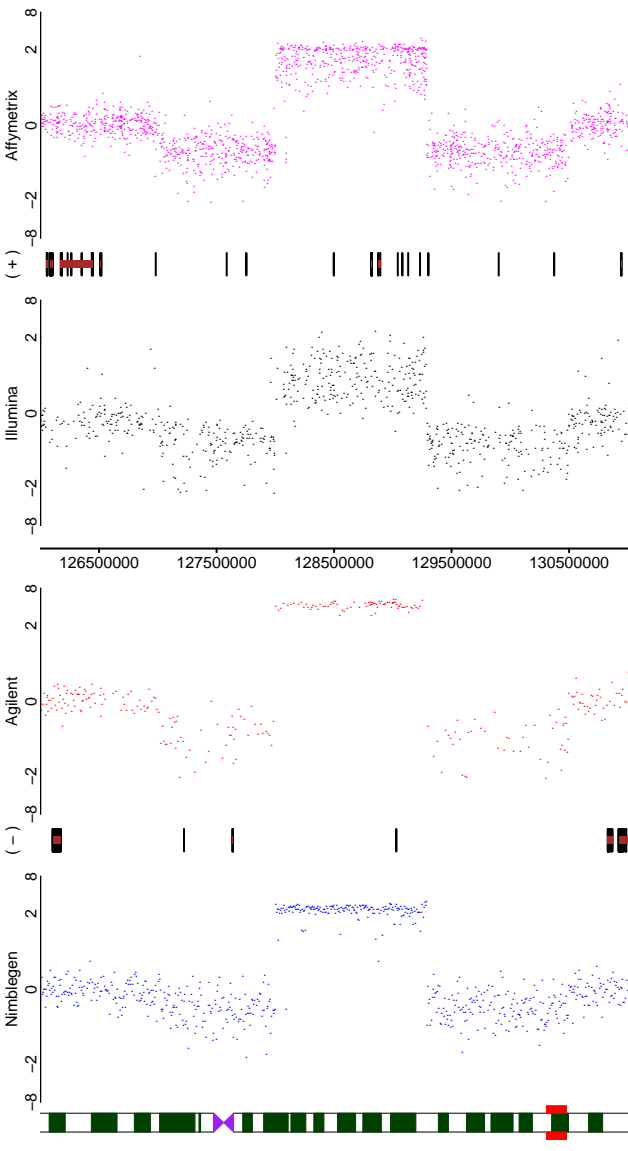

Supplement: Additional file 2 — Details of SUM159. Plots detailing the loss-gain-loss aberration on chromosome 8 of SUM159. [file 1471-2164-10-588-S2.PDF]

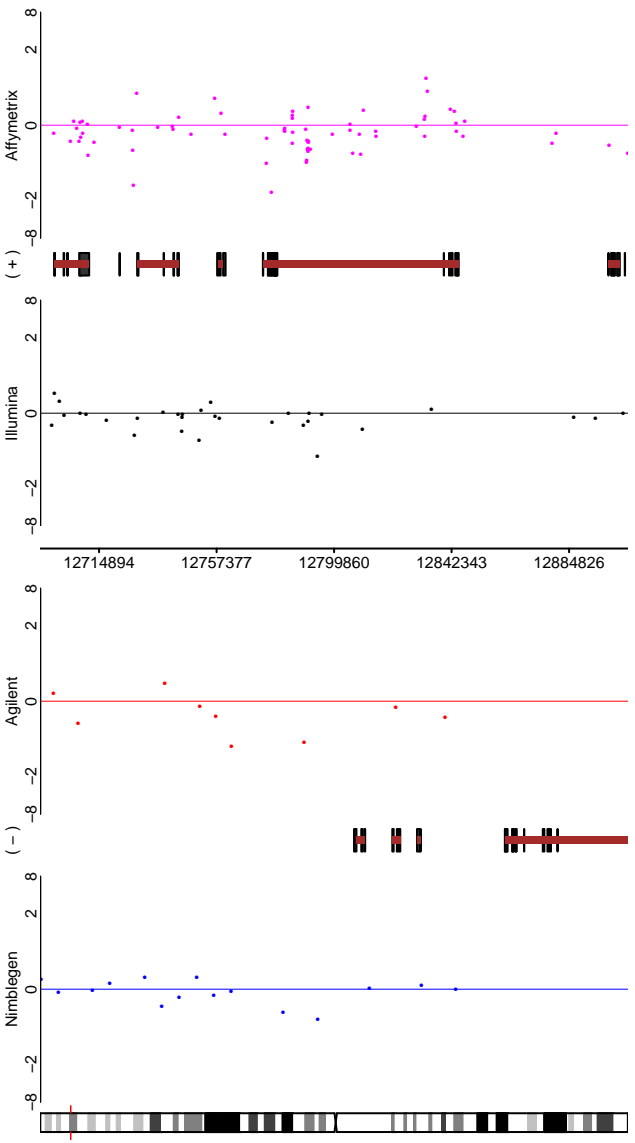

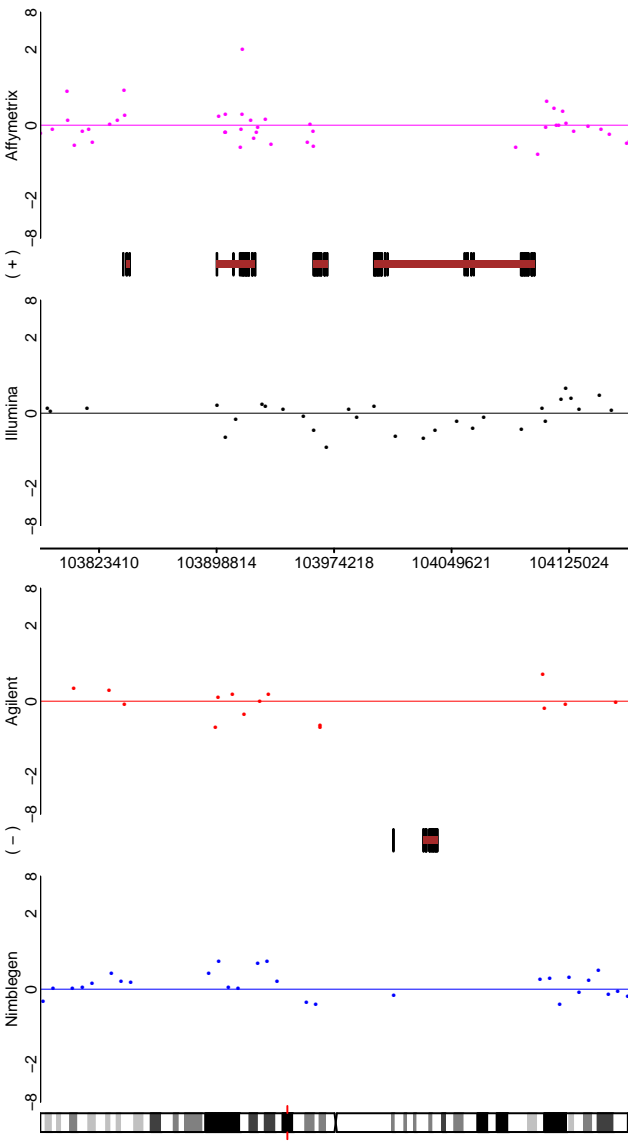

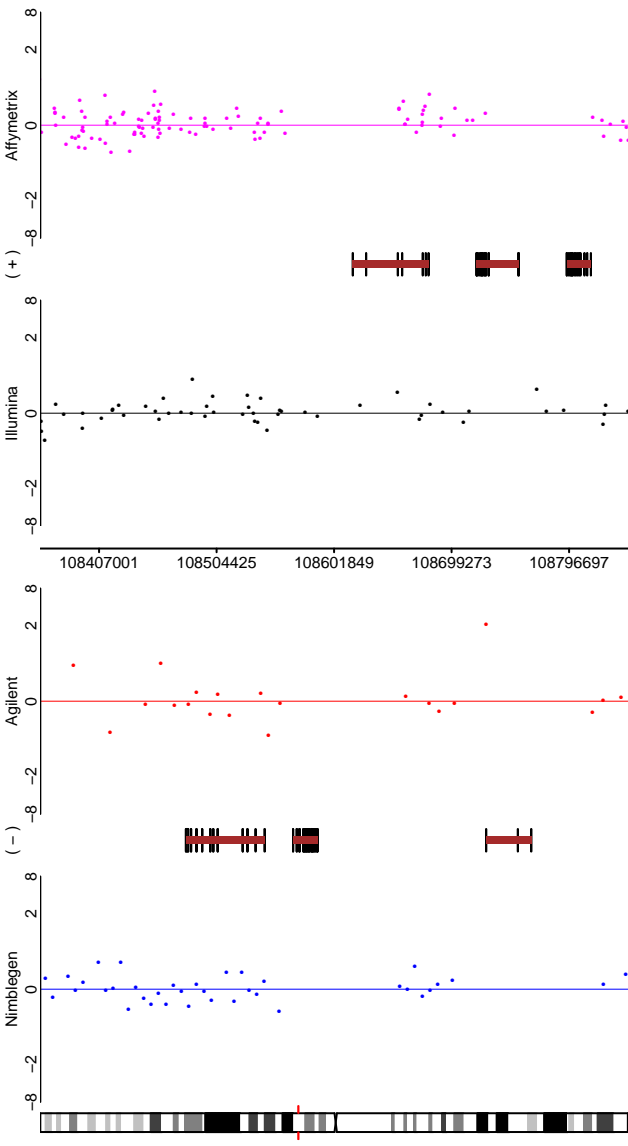

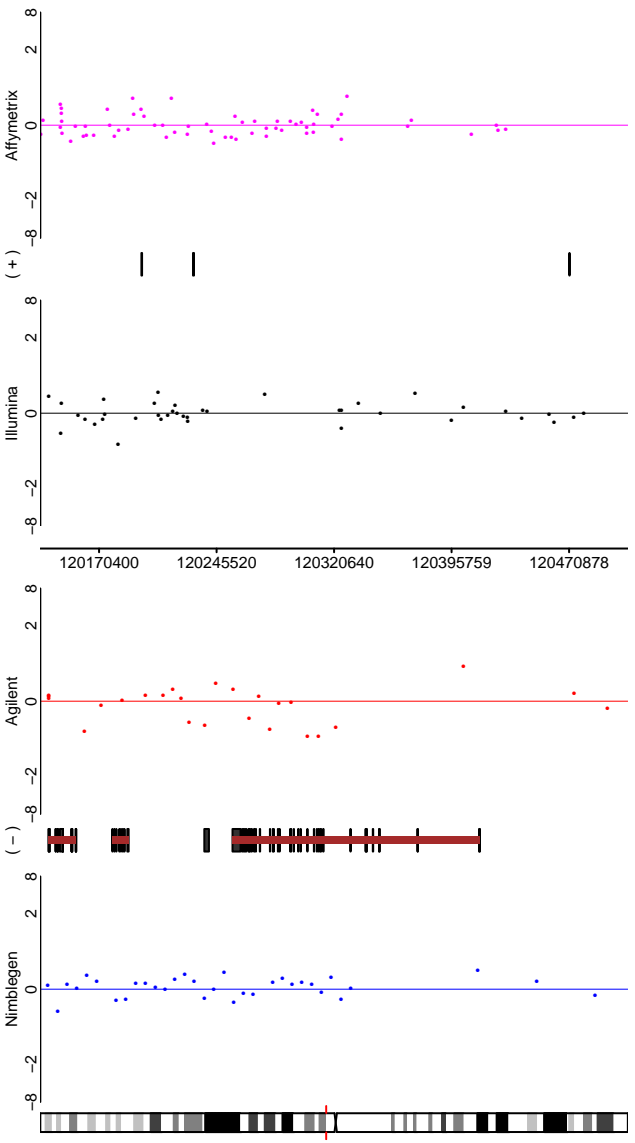

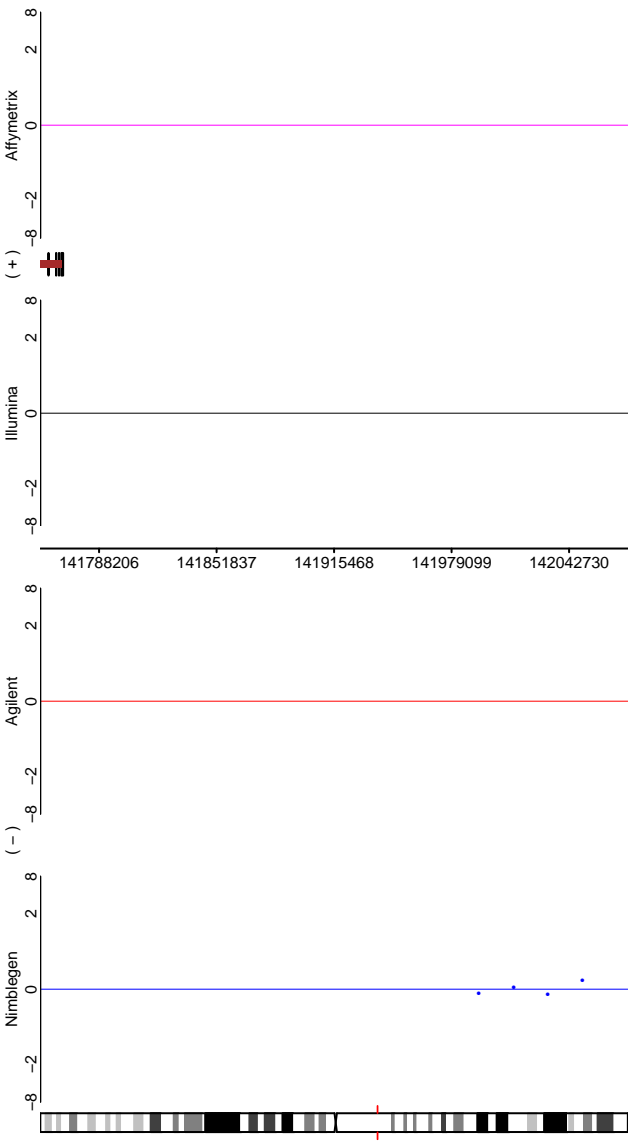

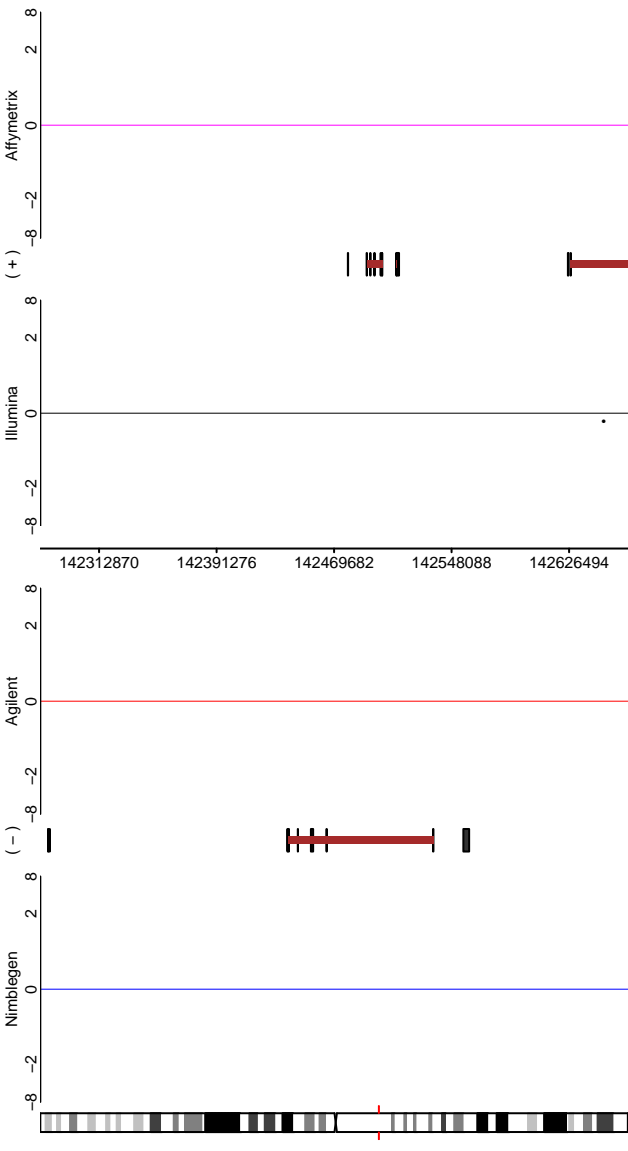

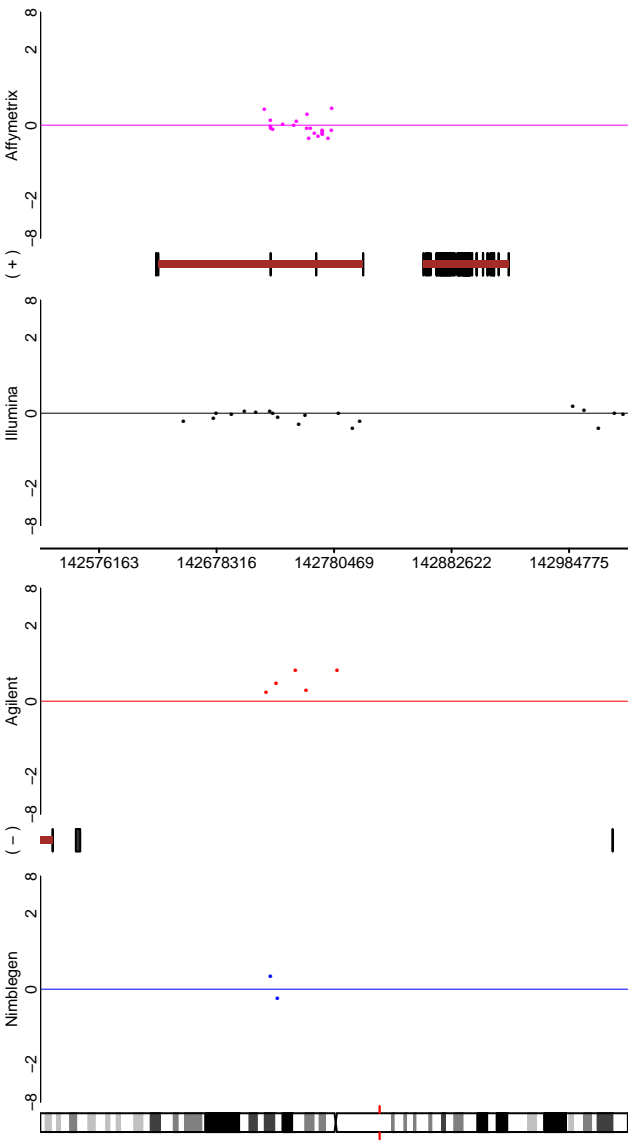

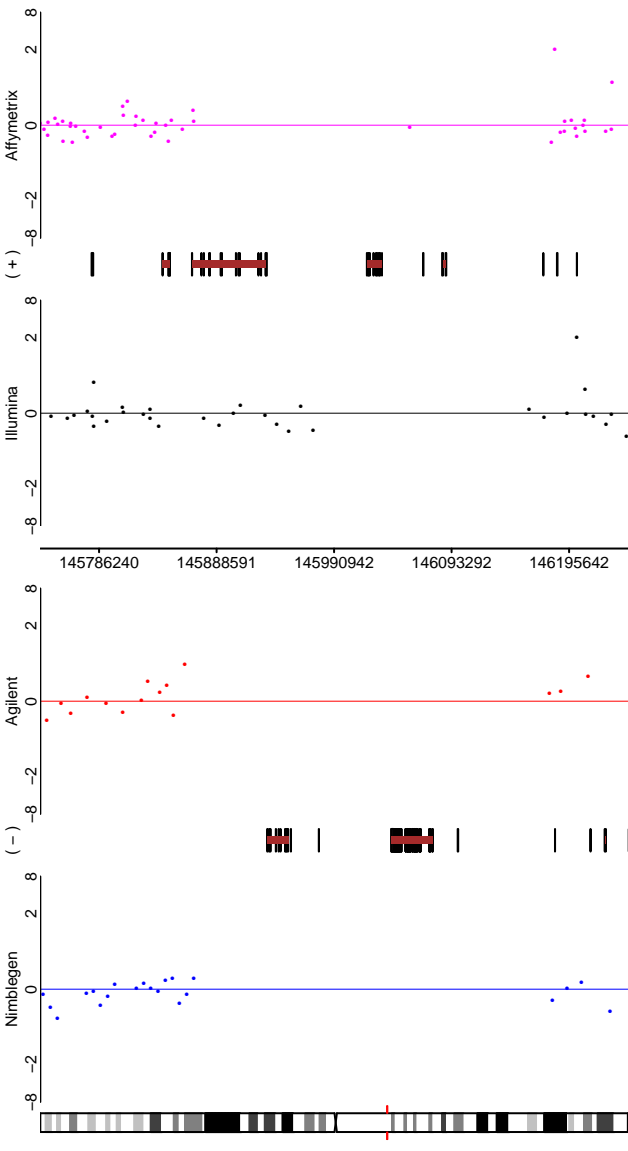

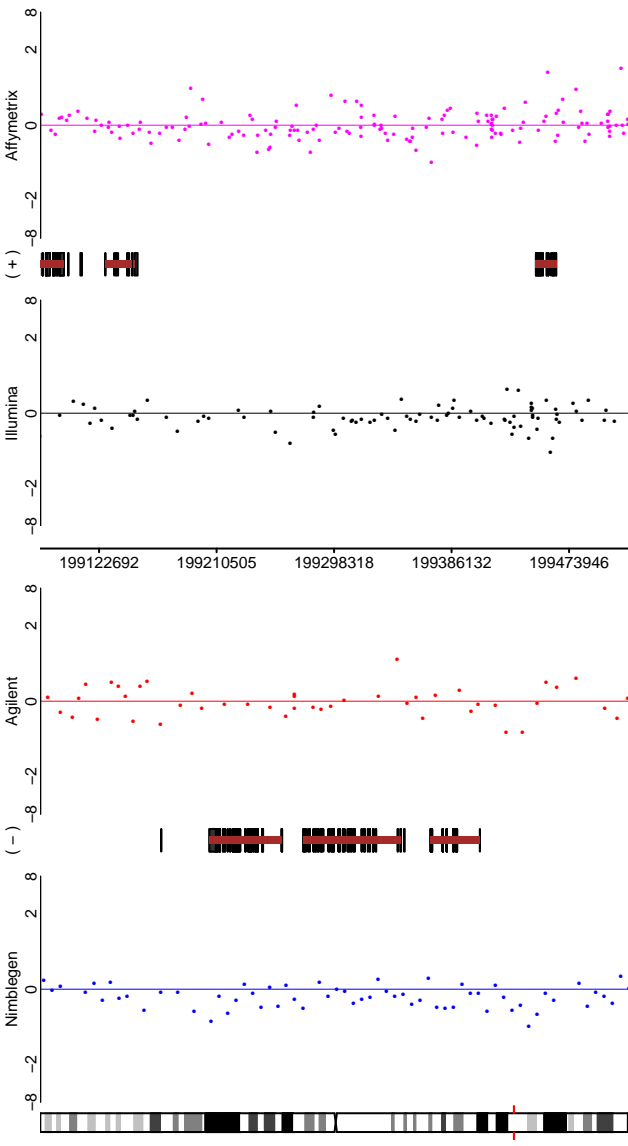

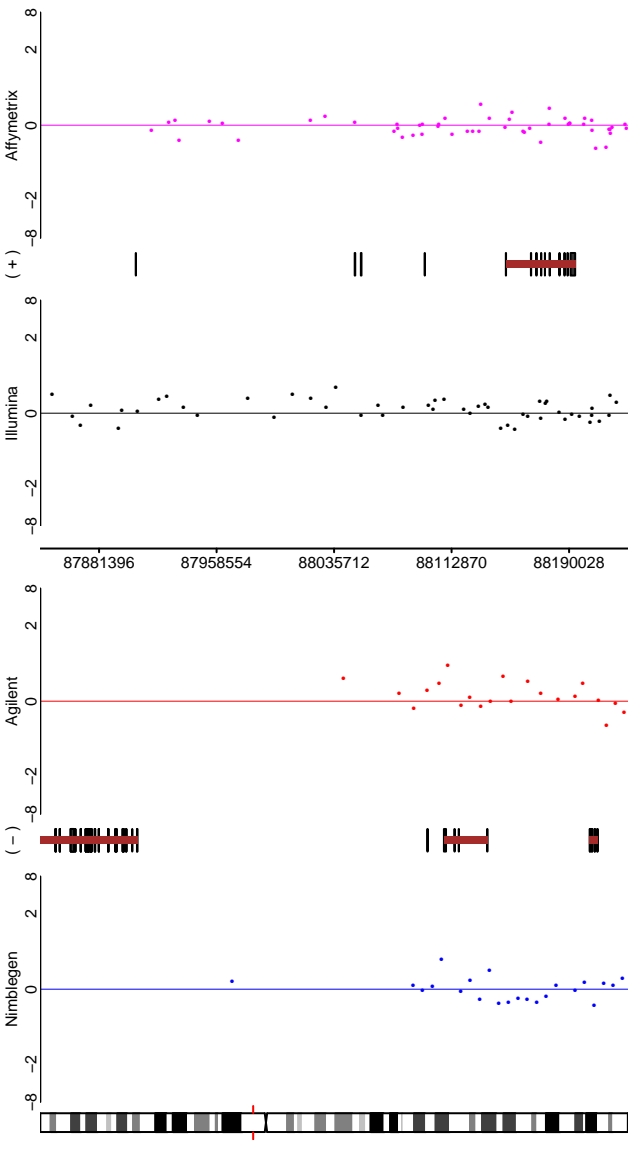

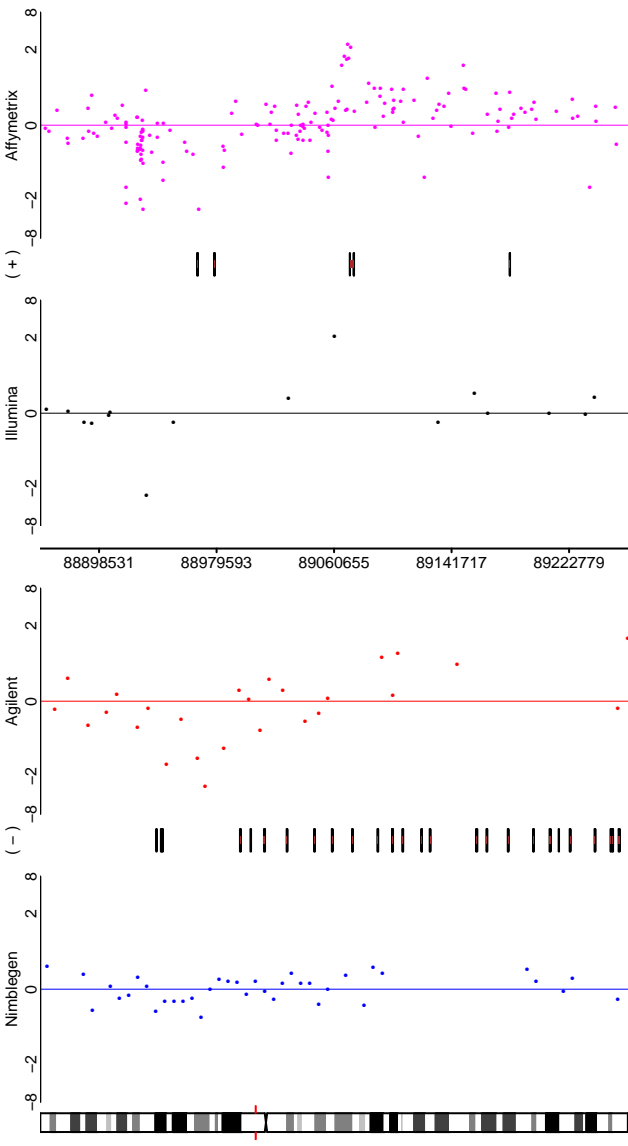

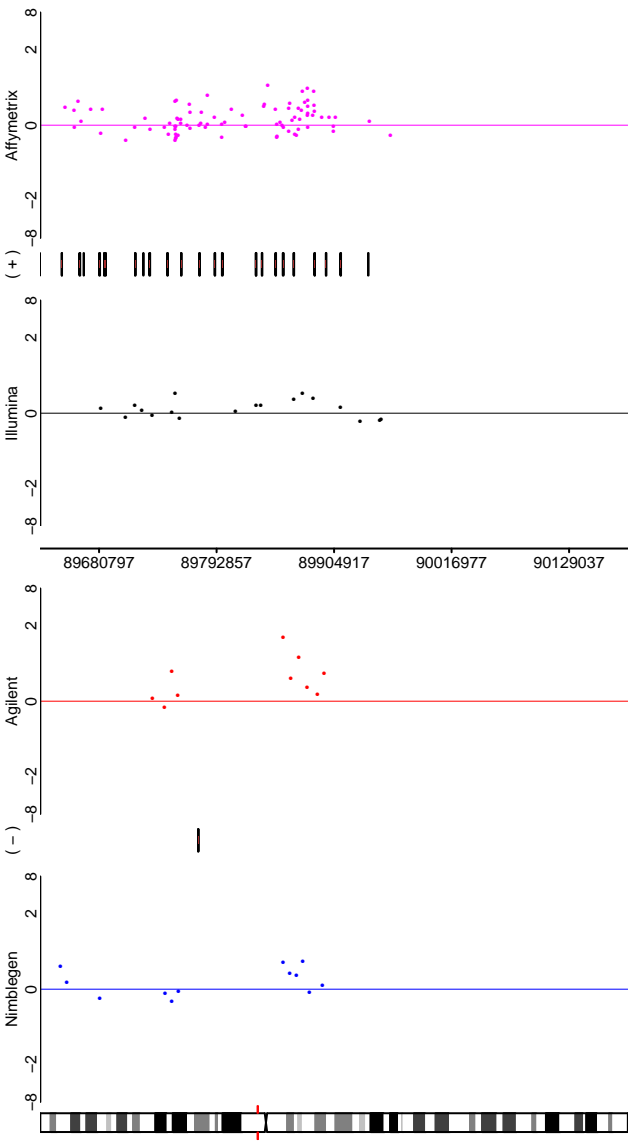

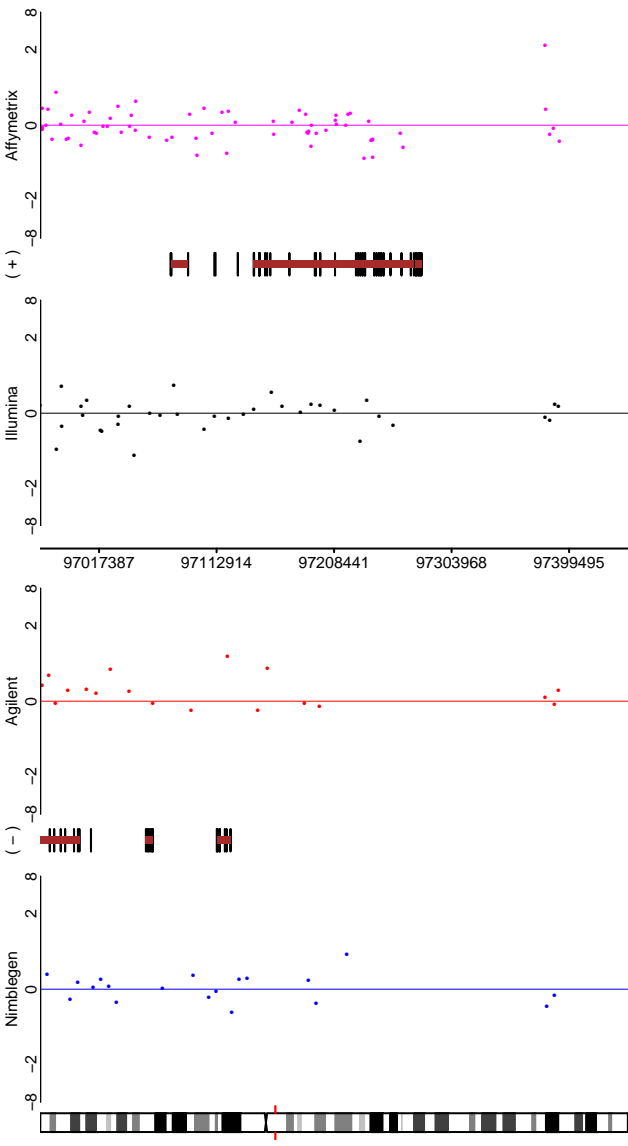

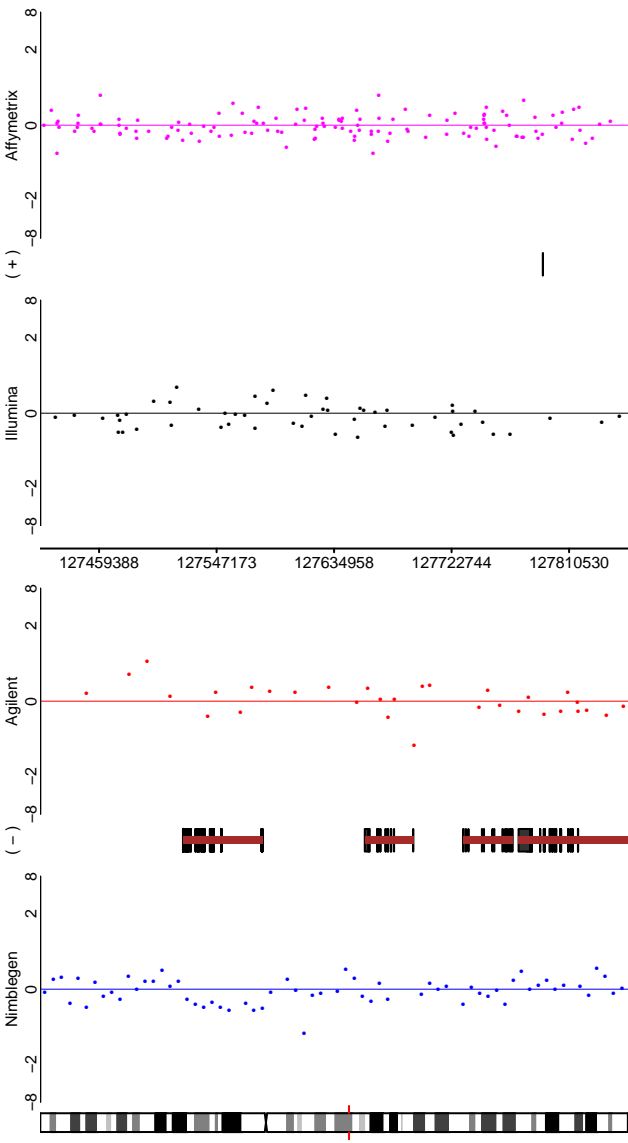

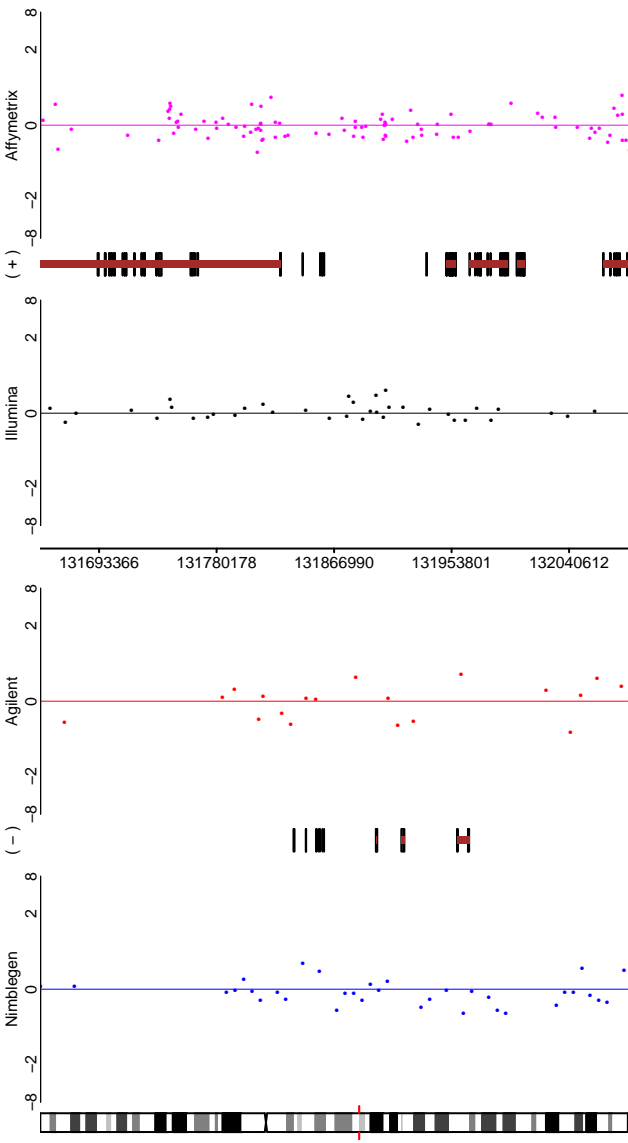

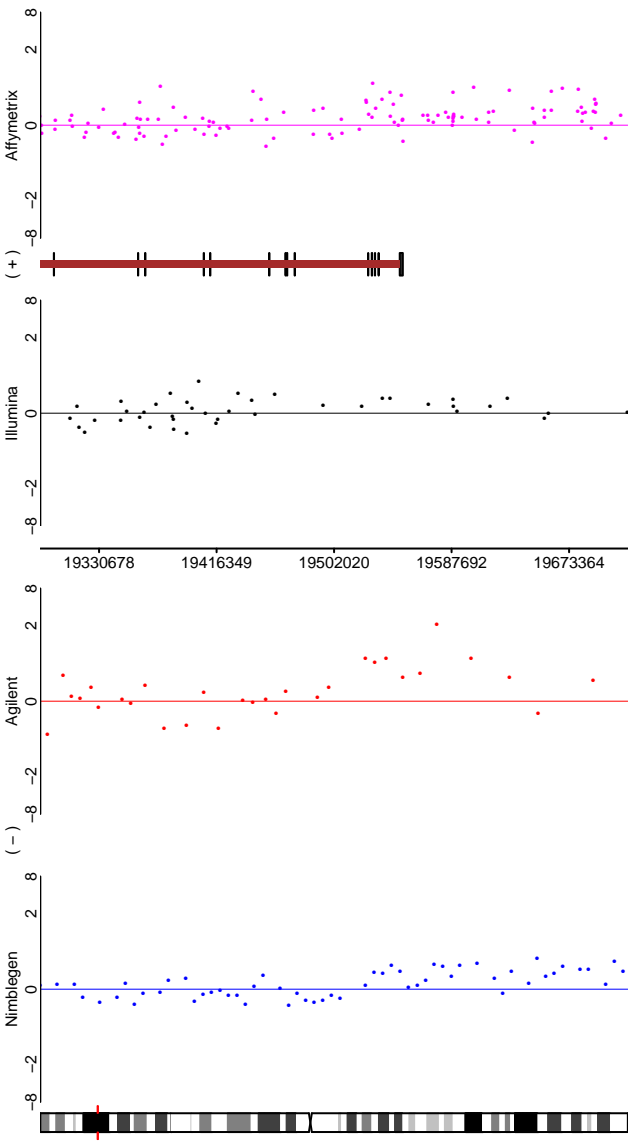

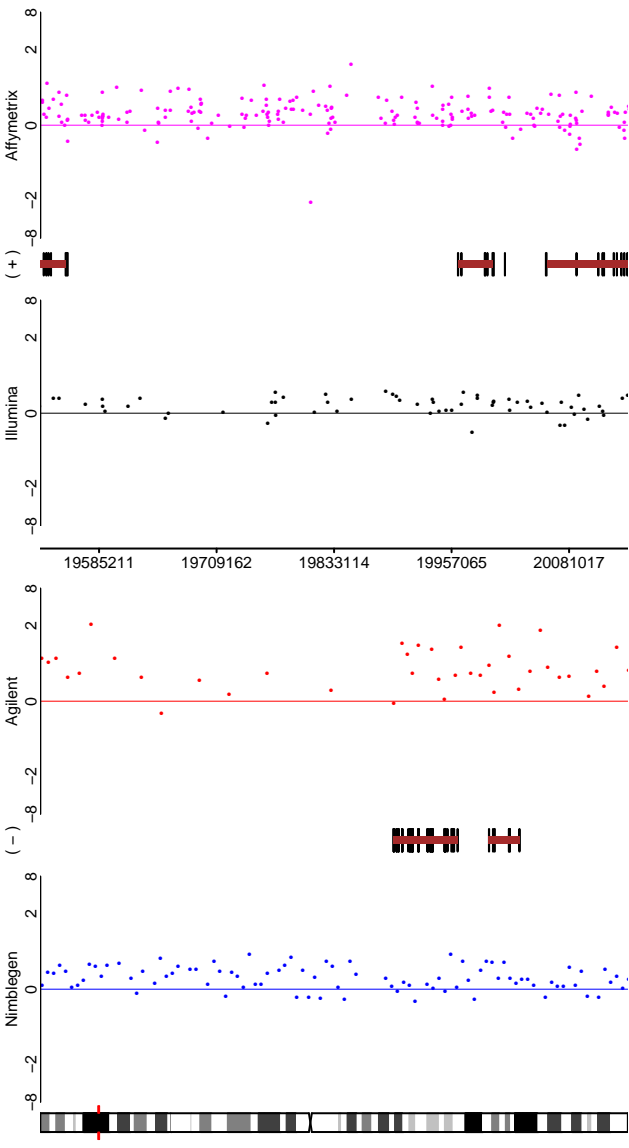

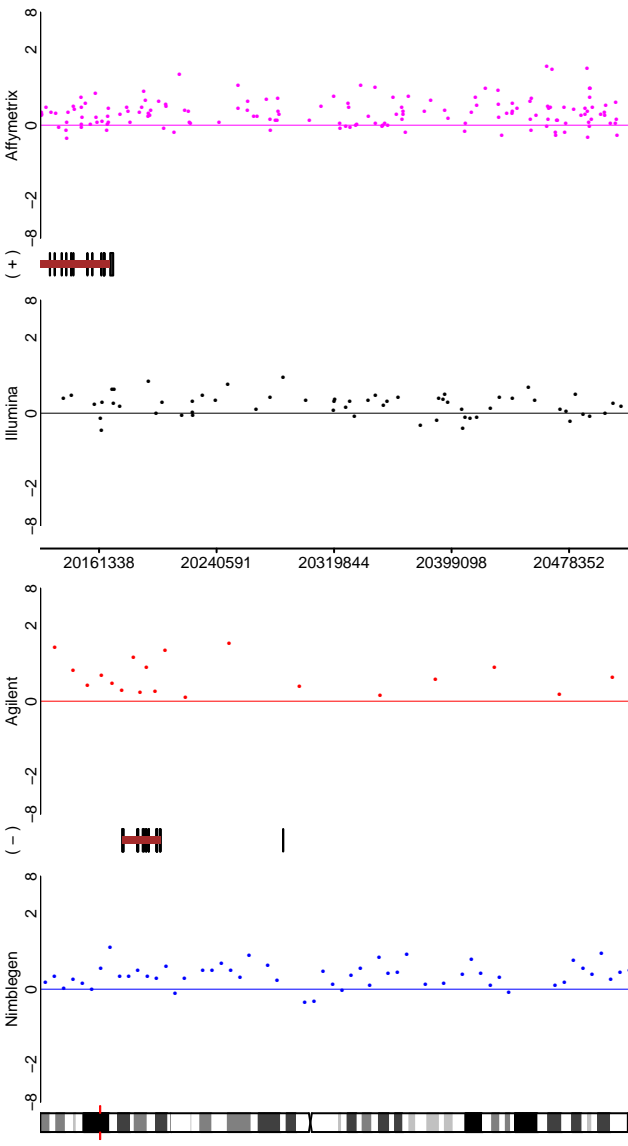

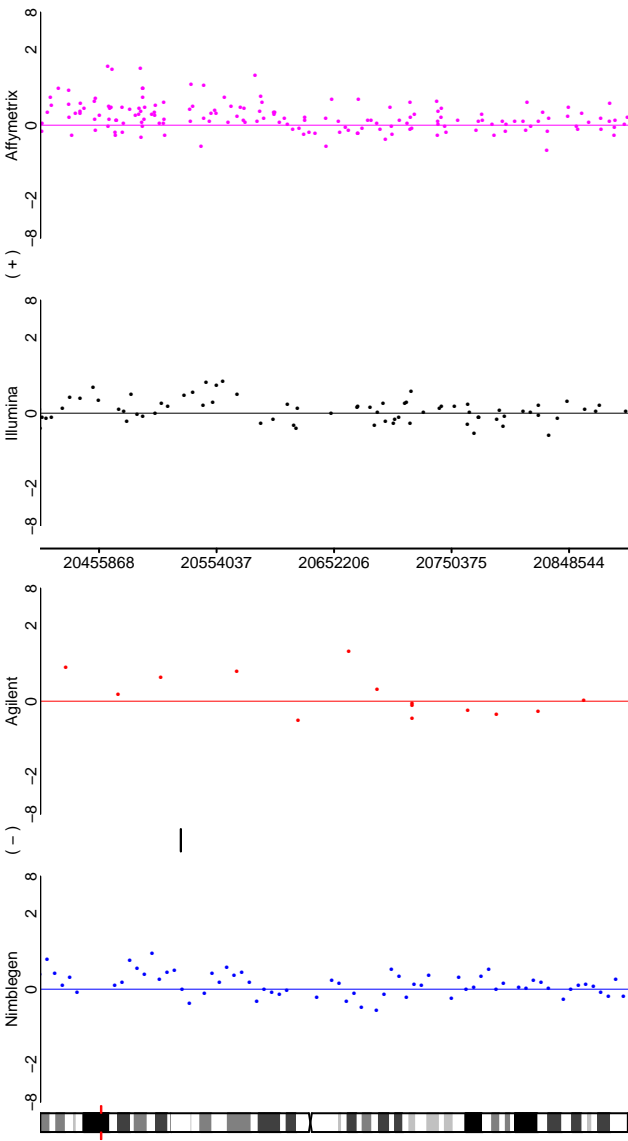

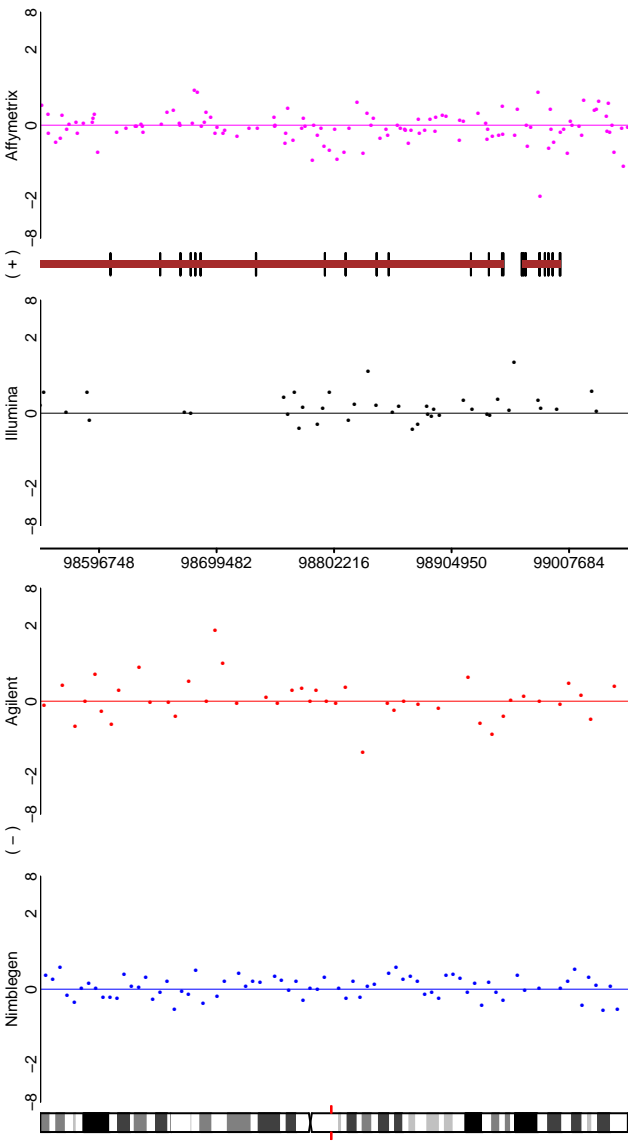

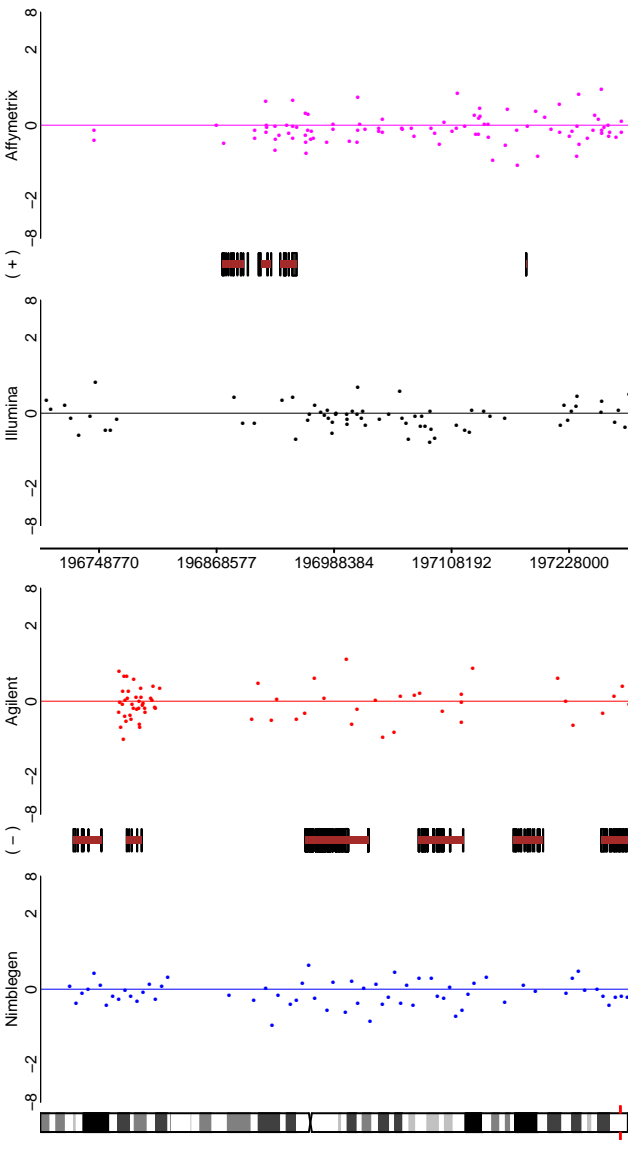

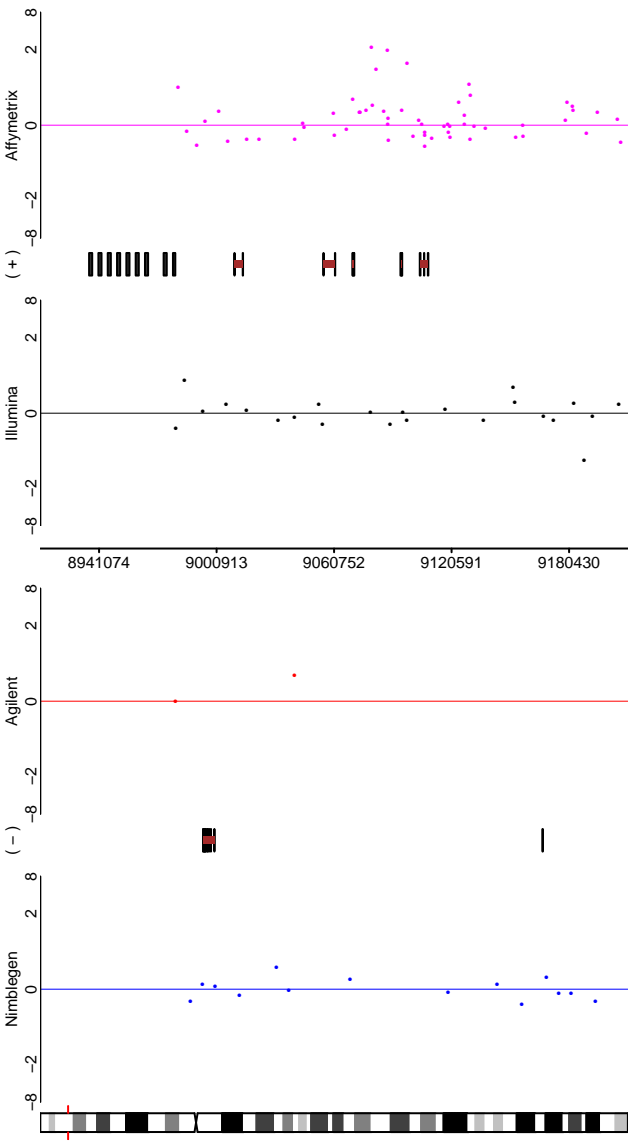

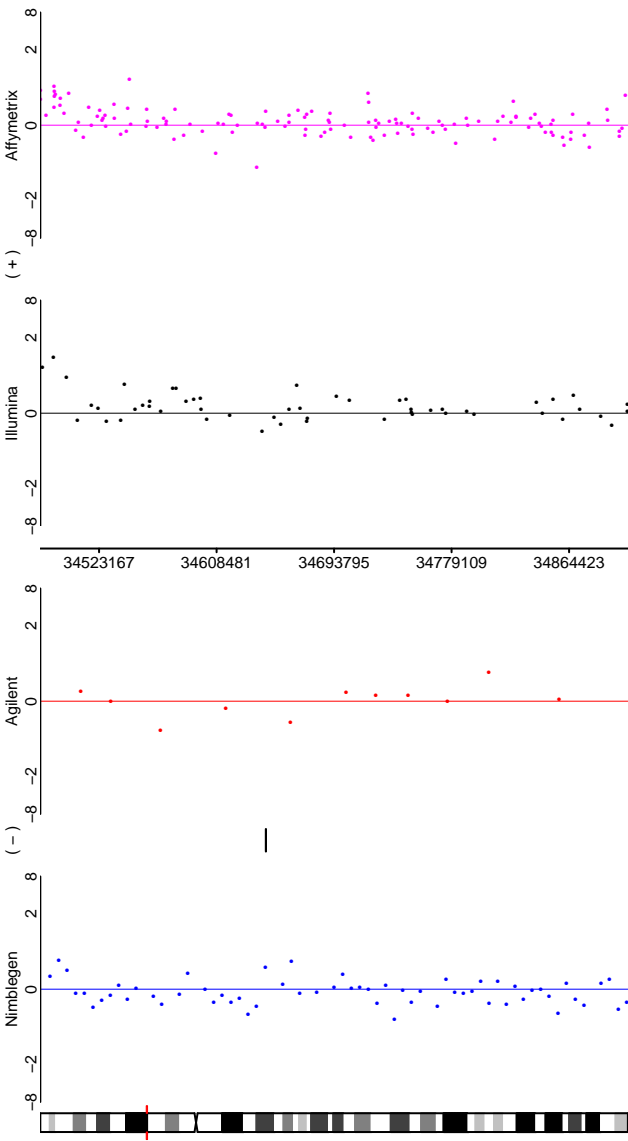

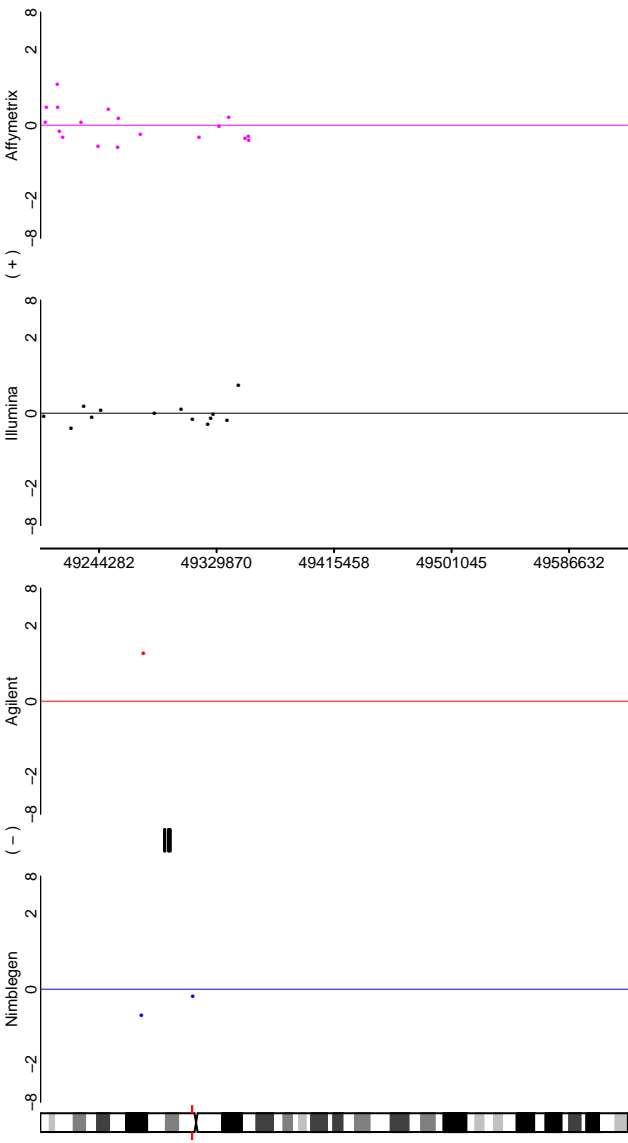

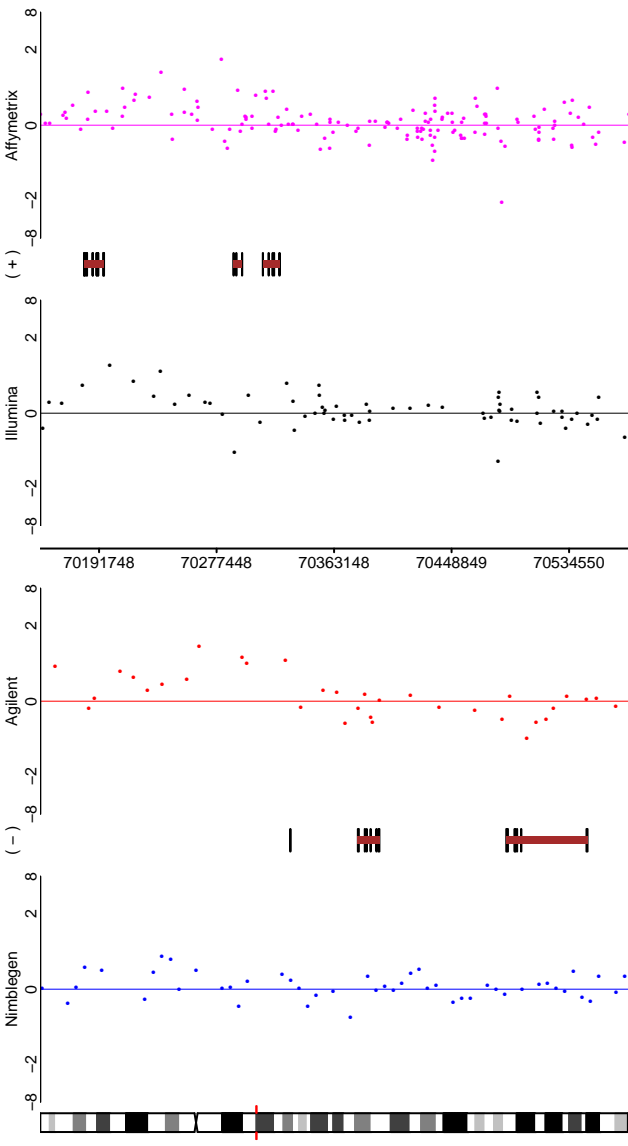

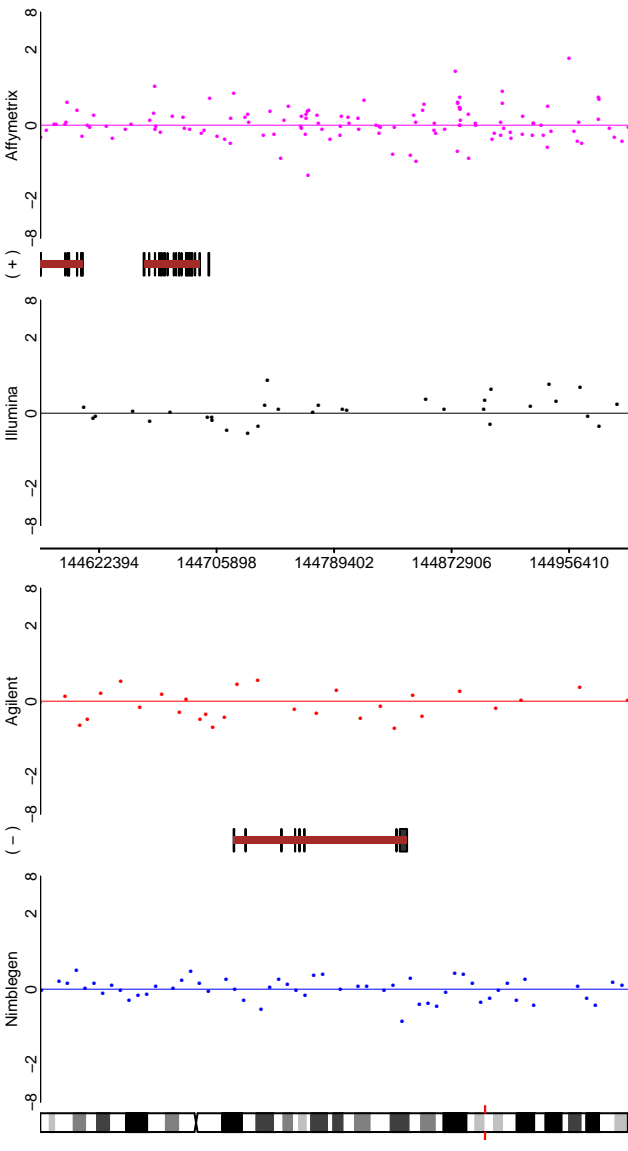

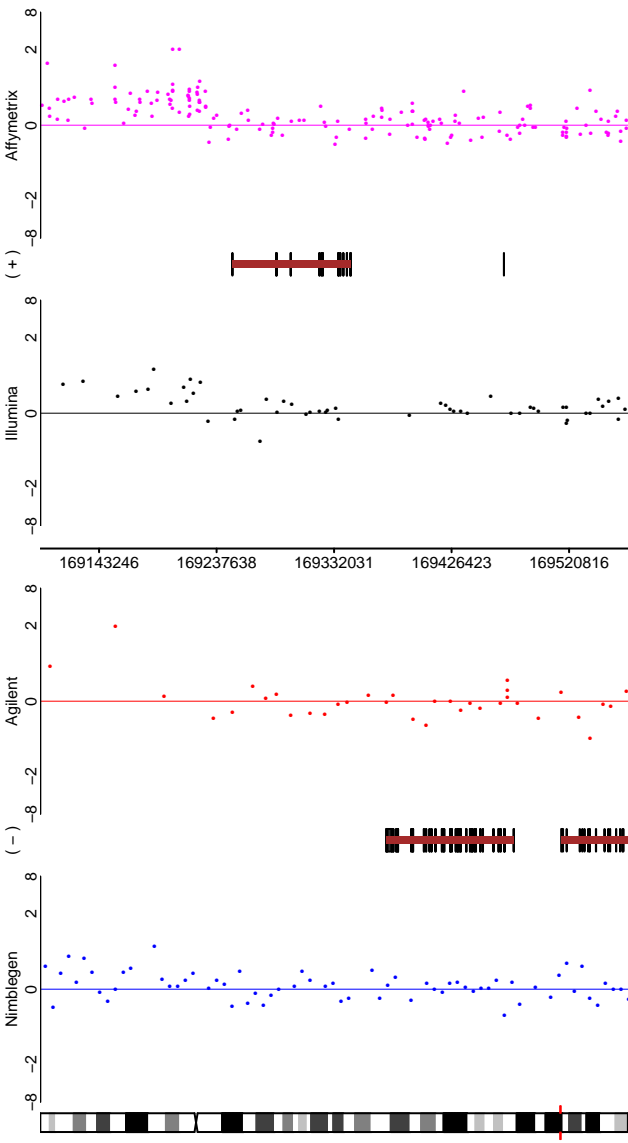

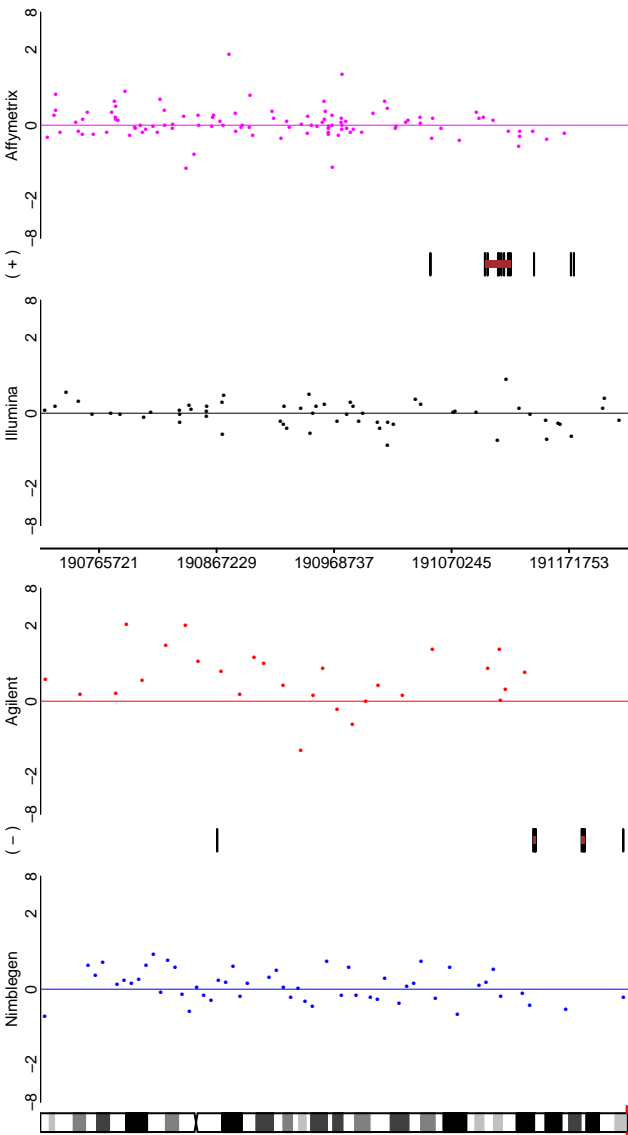

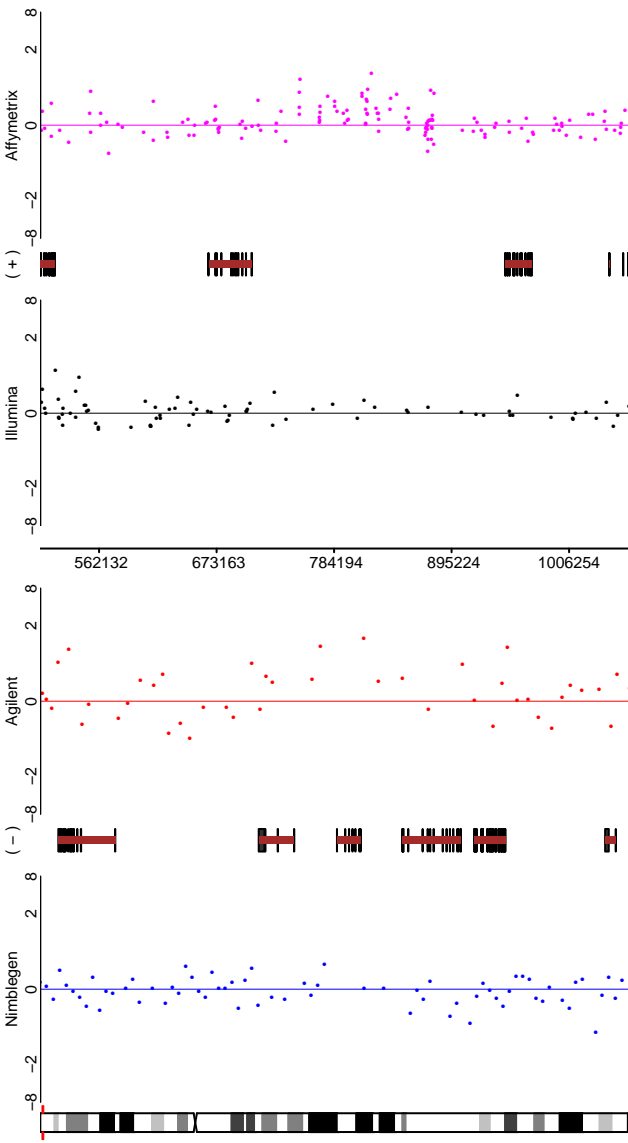

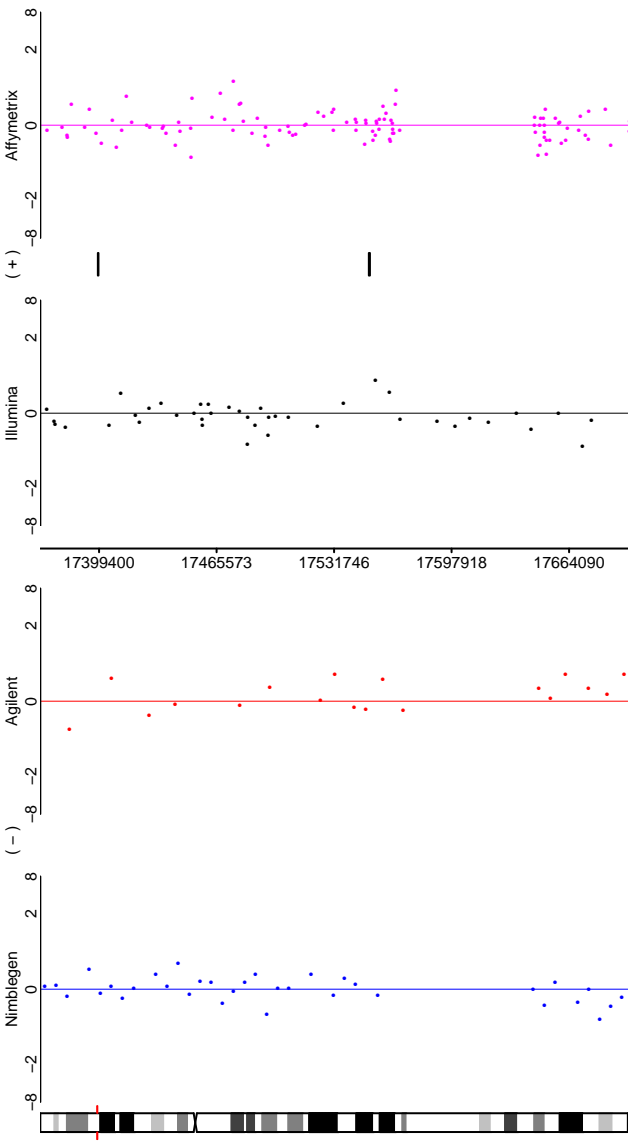

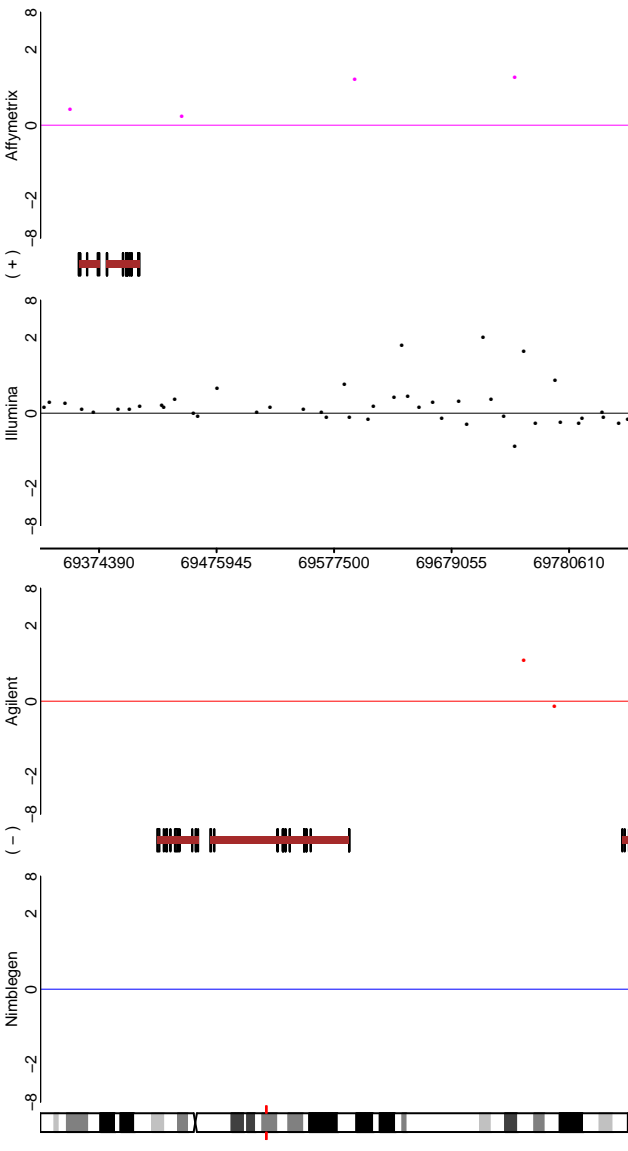

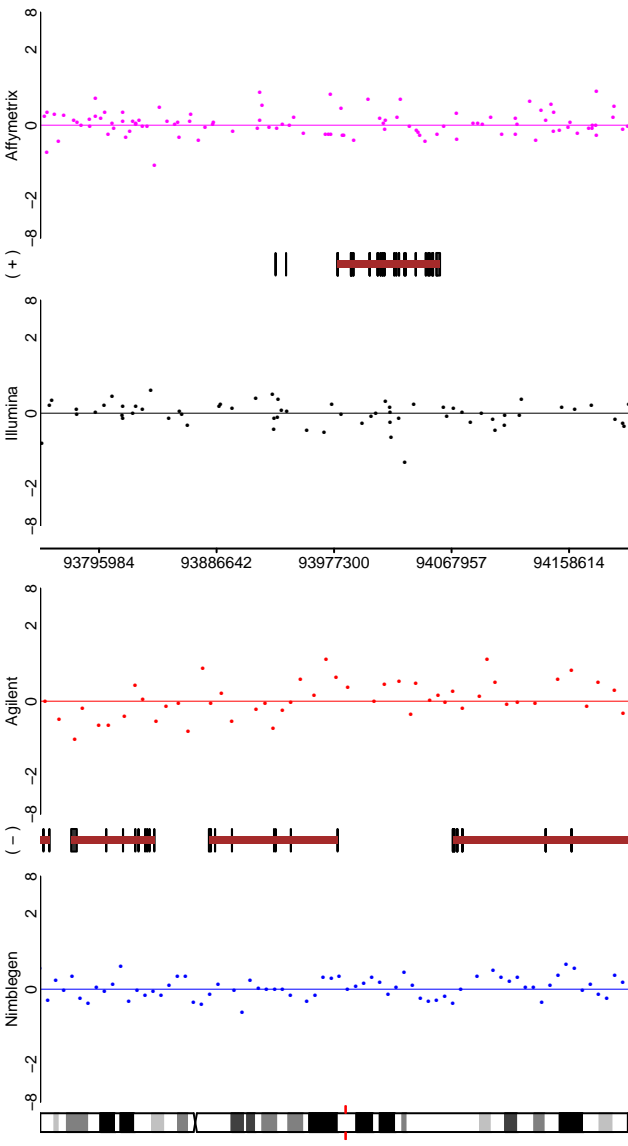

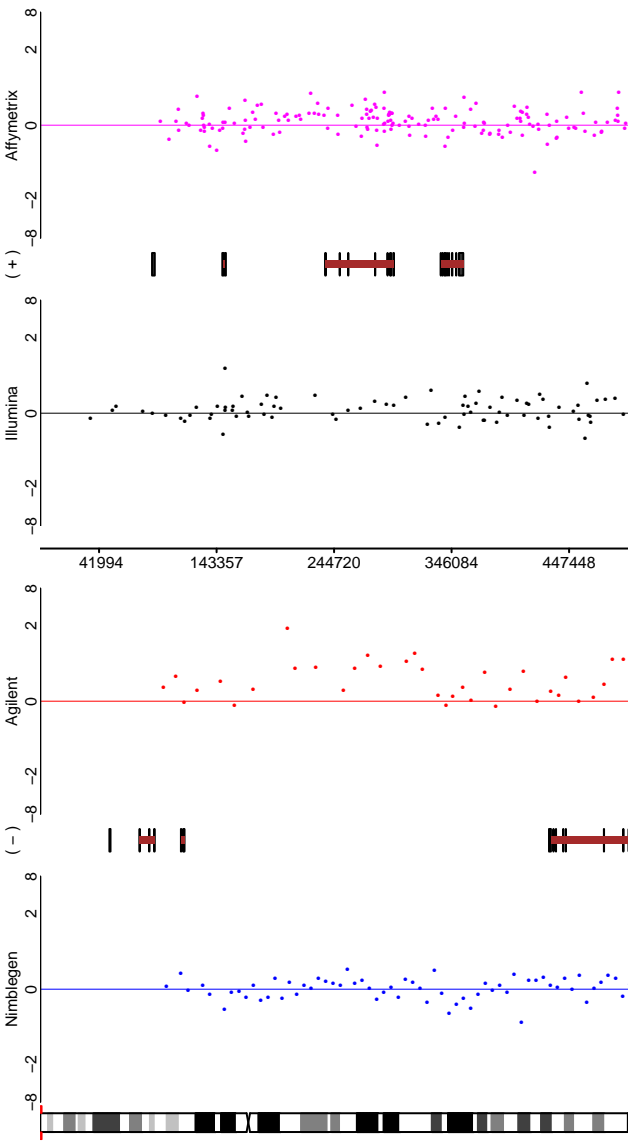

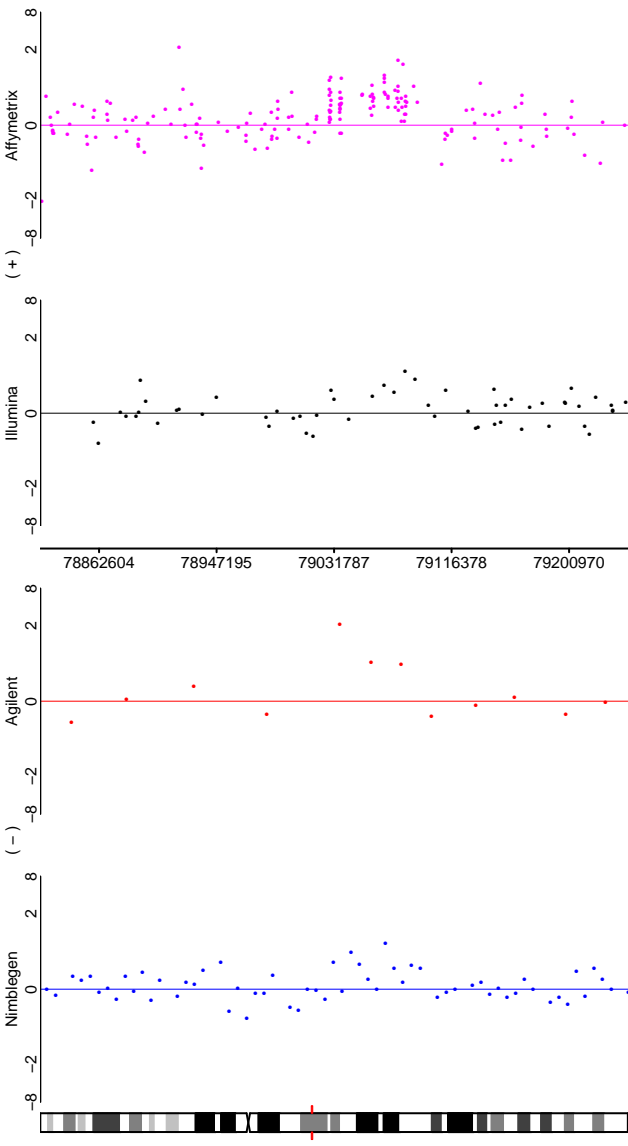

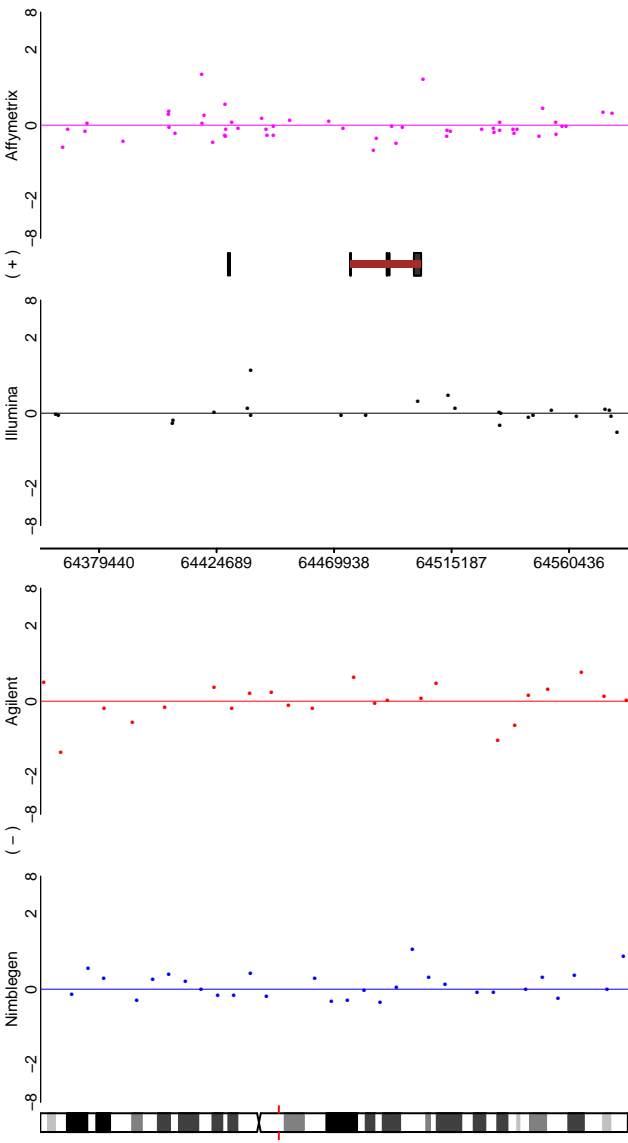

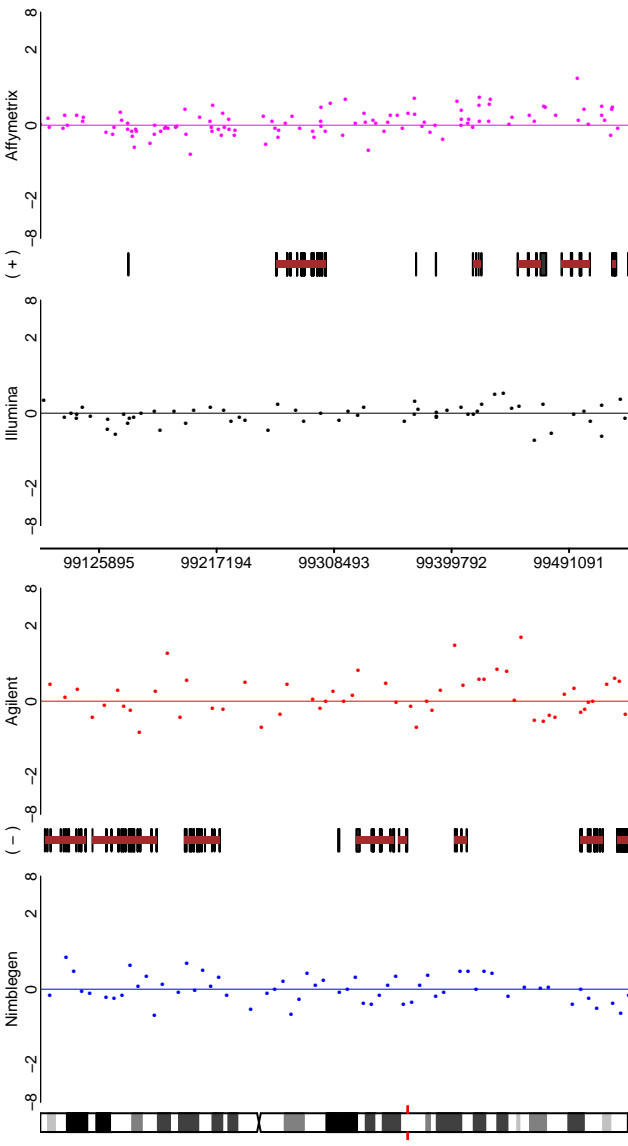

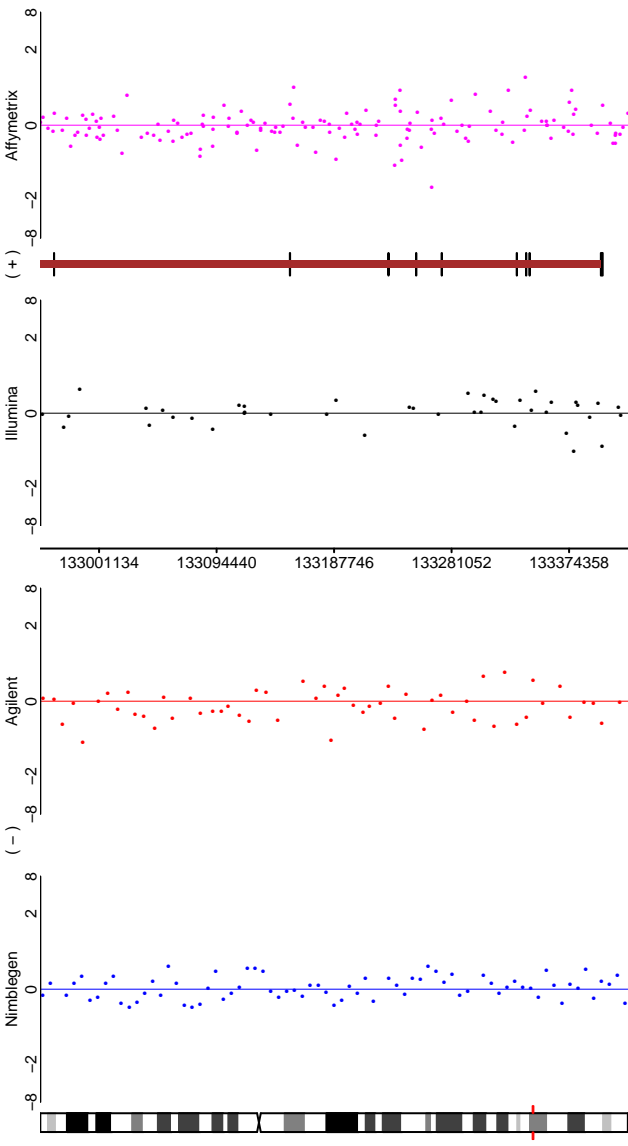

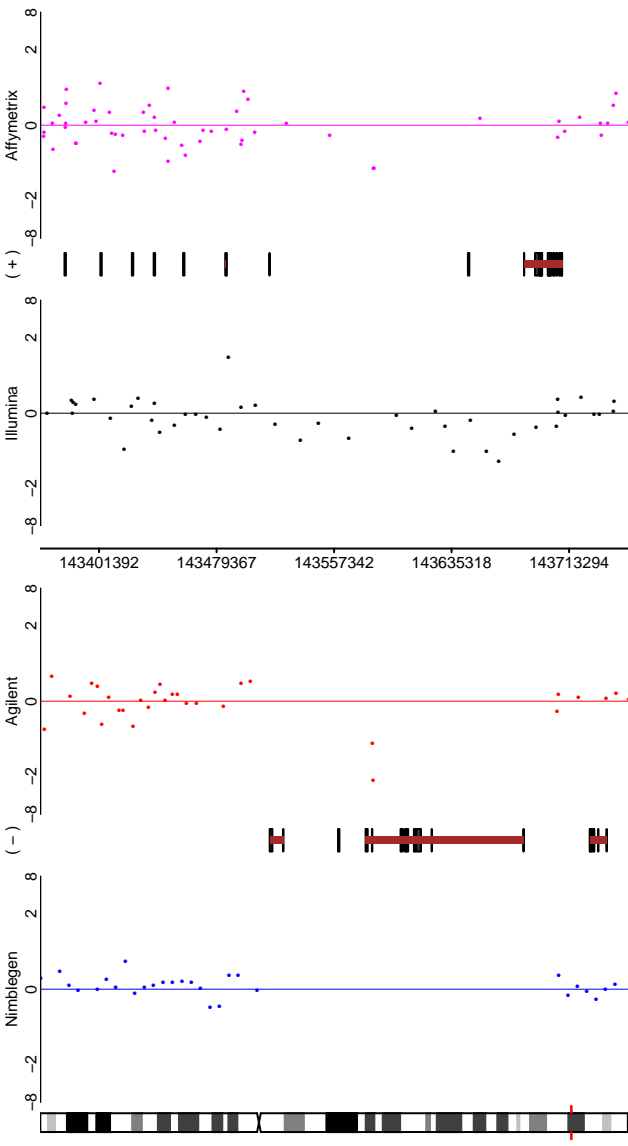

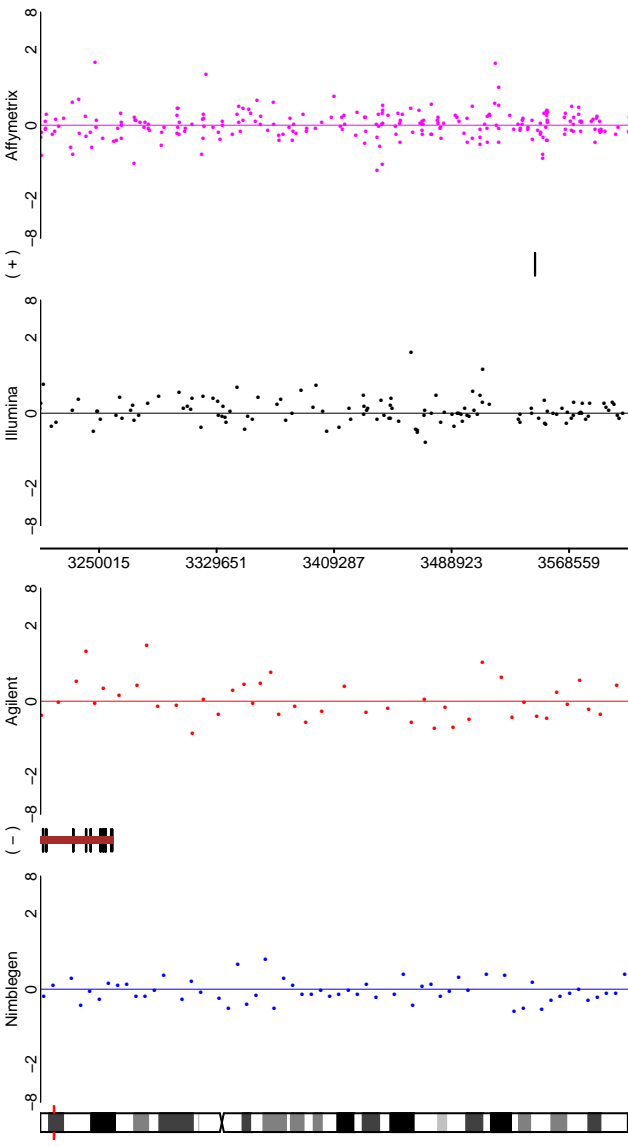

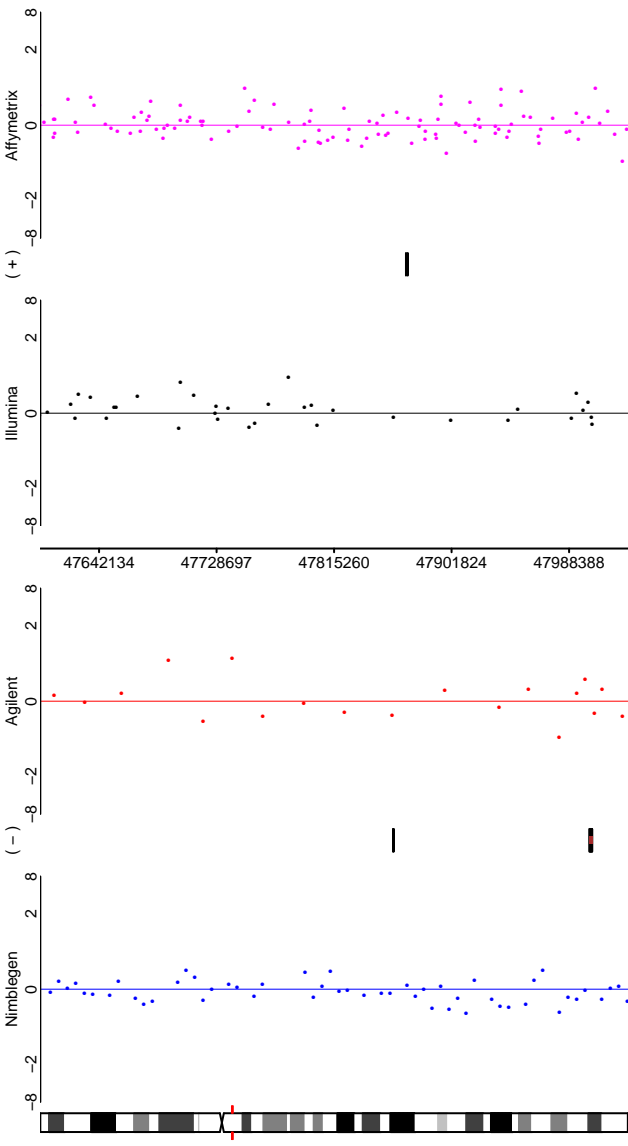

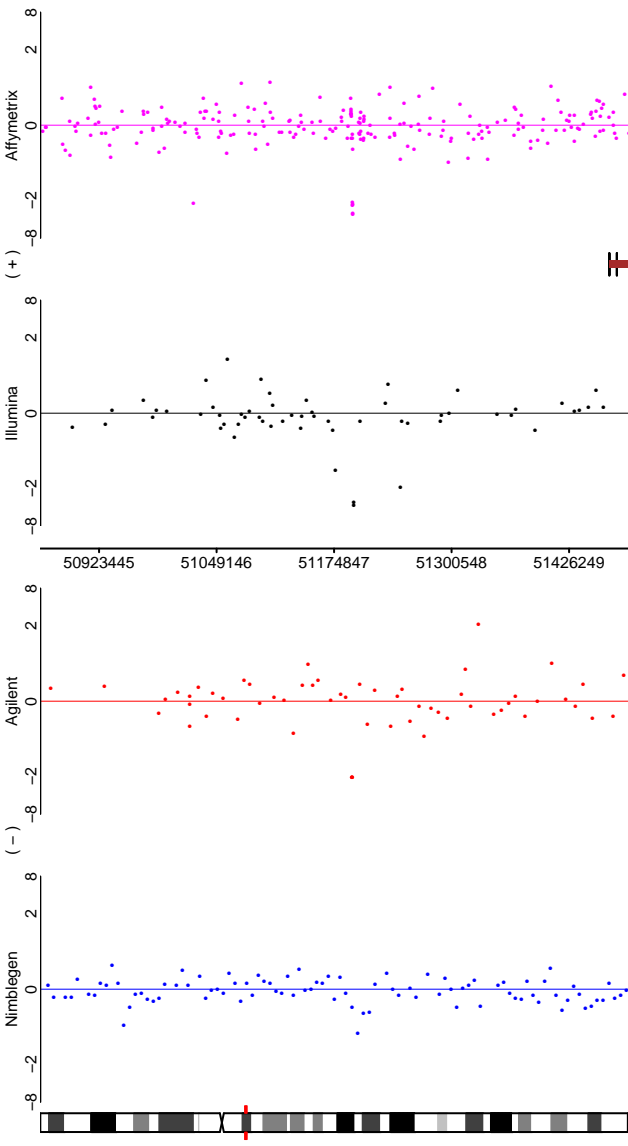

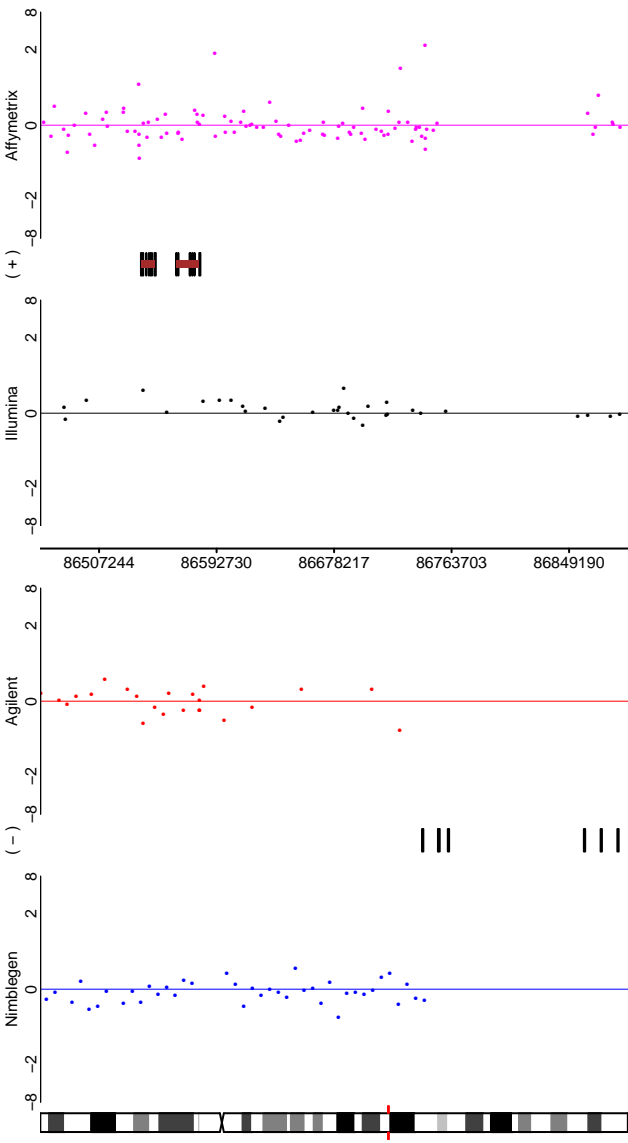

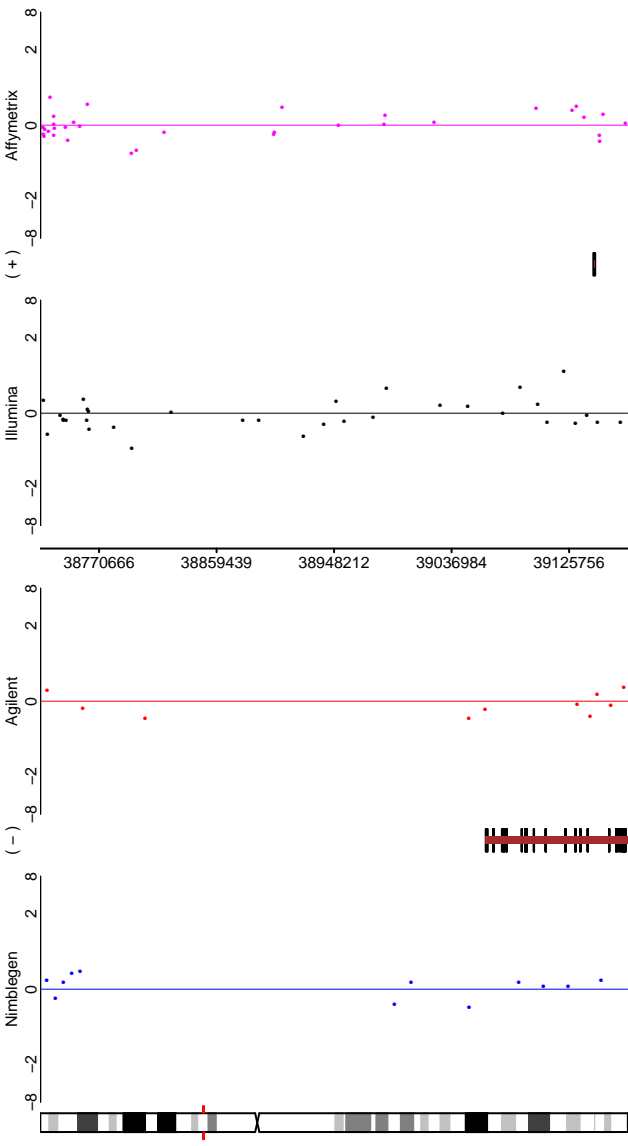

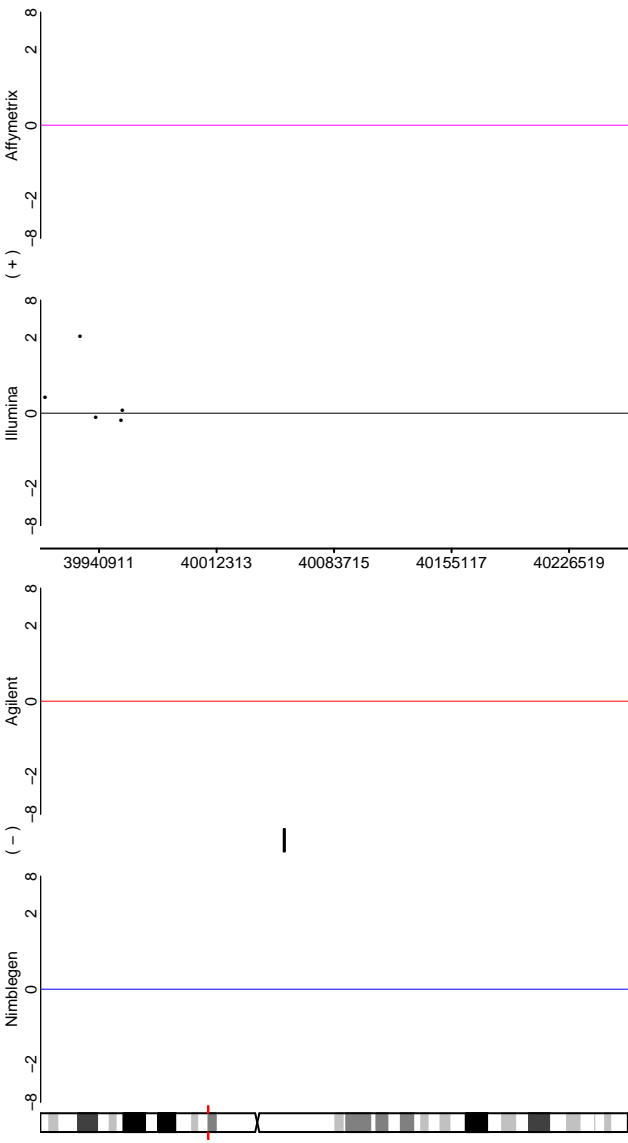

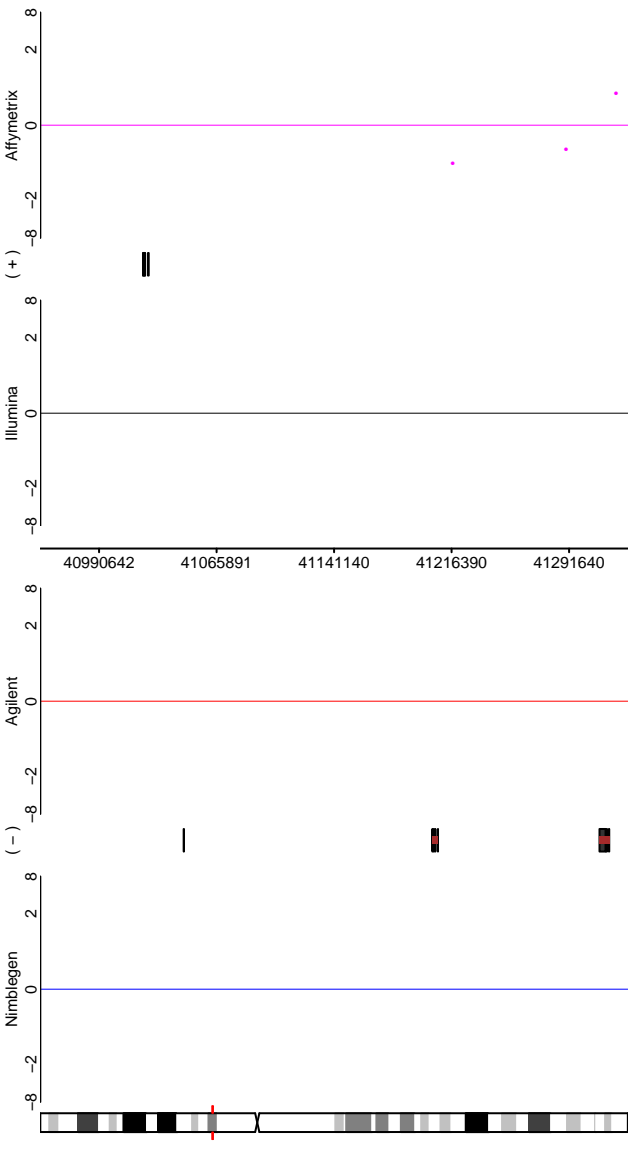

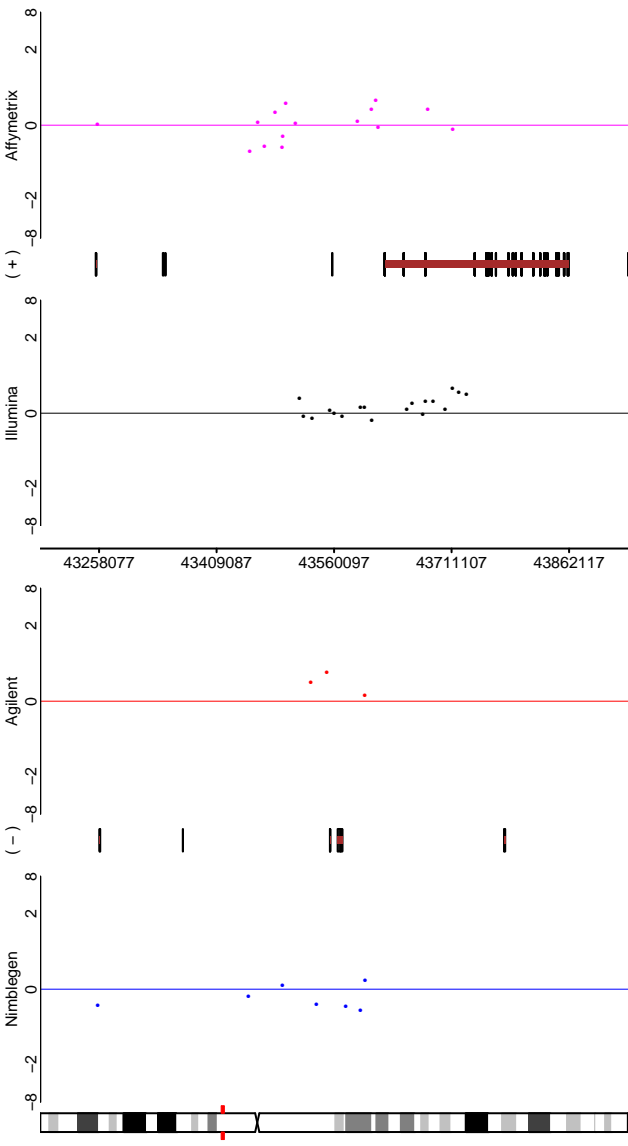

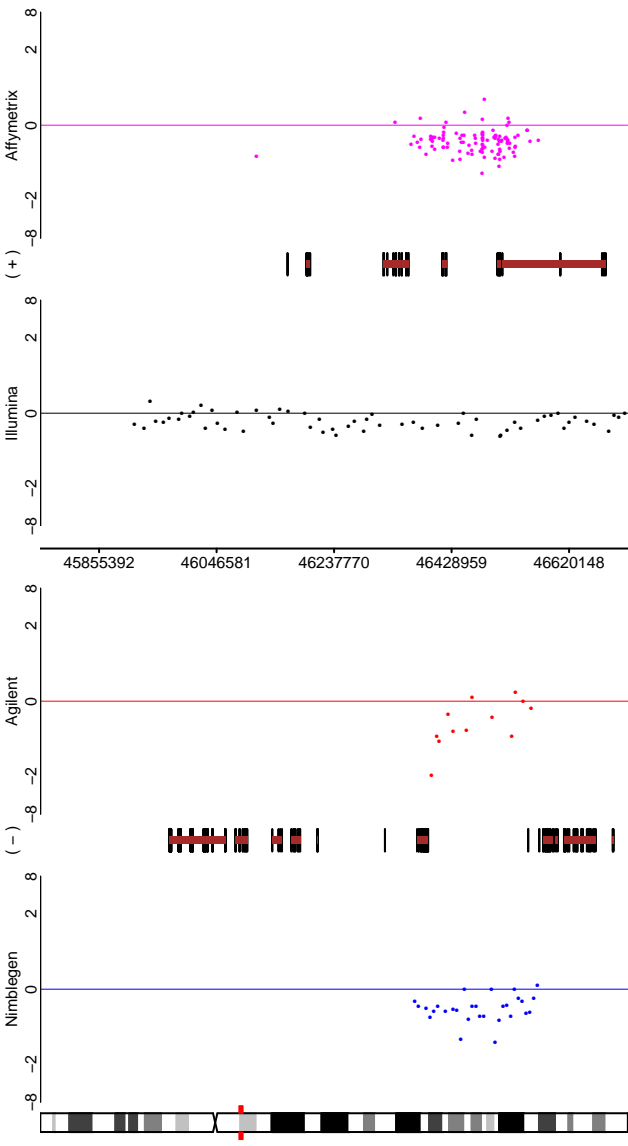

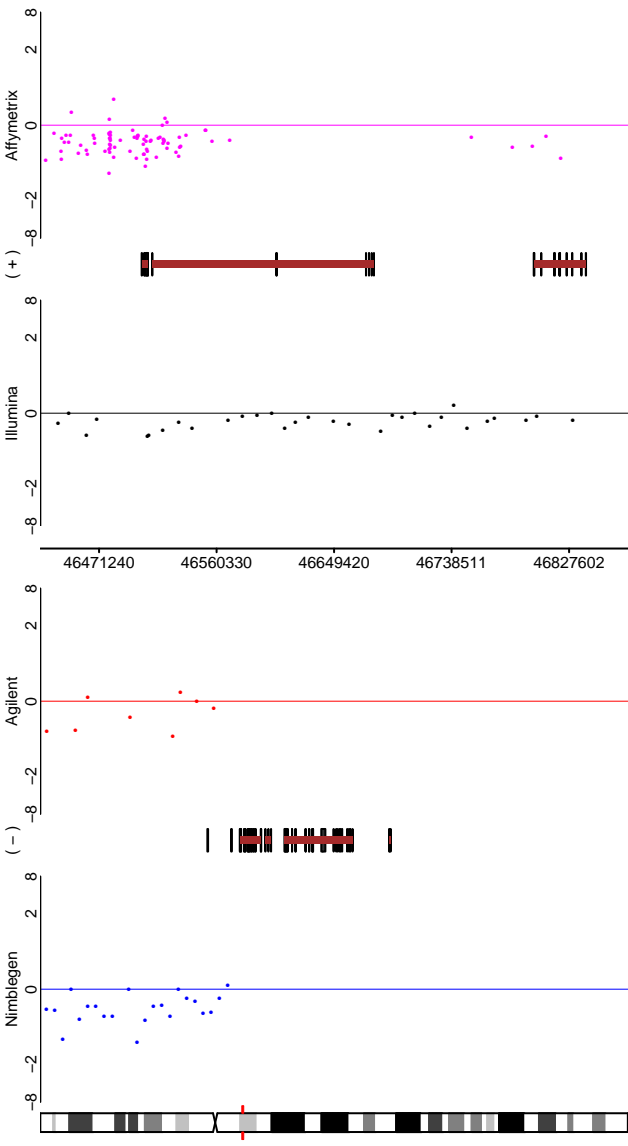

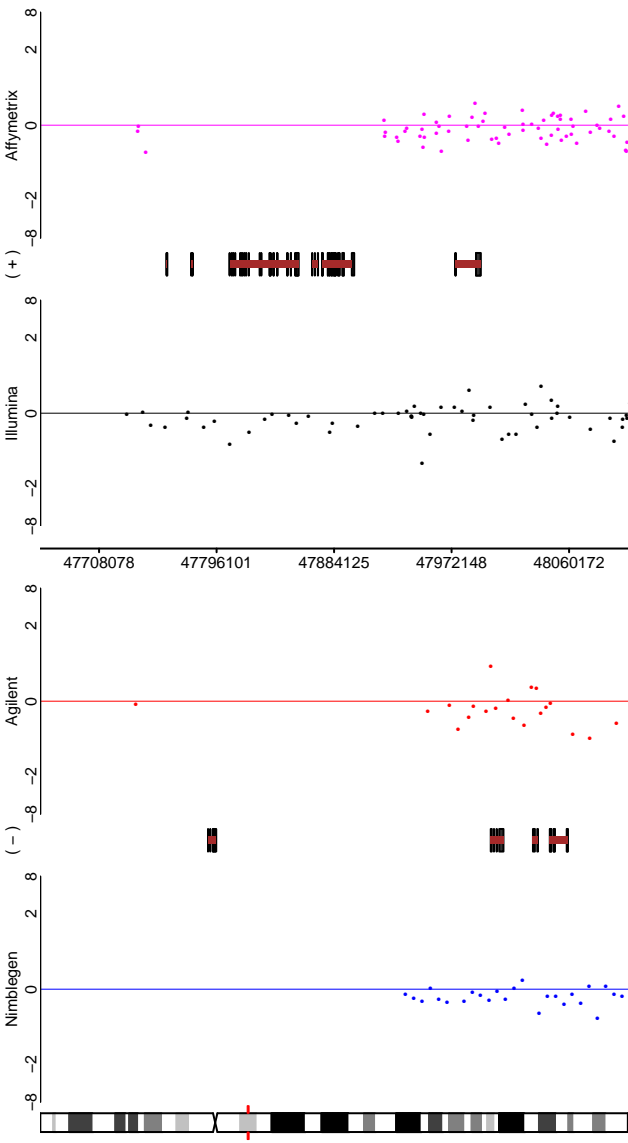

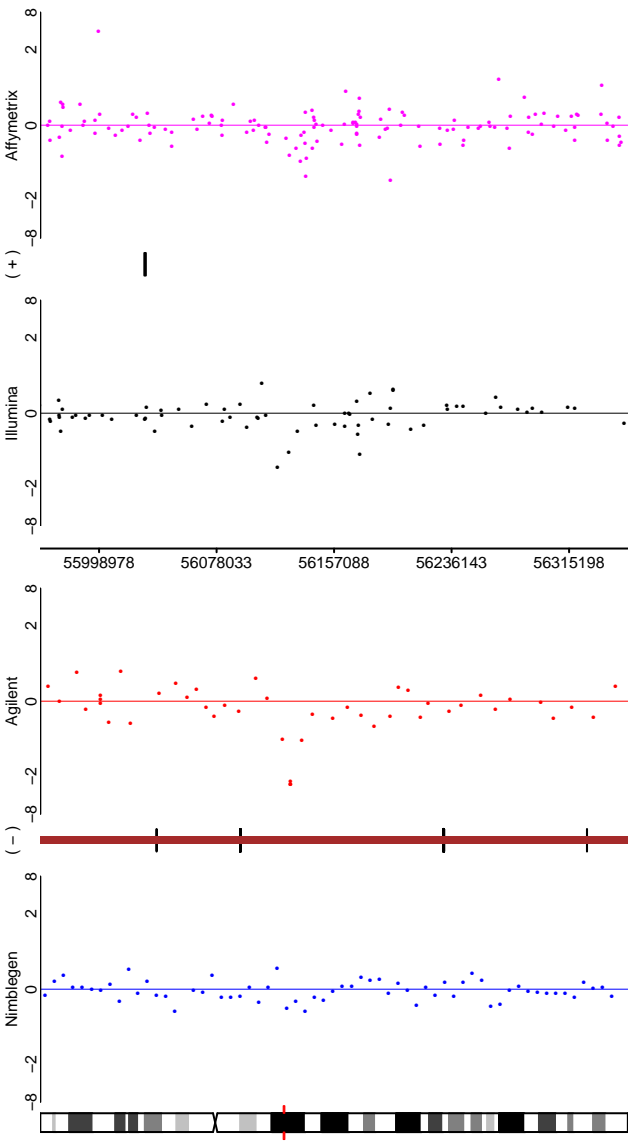

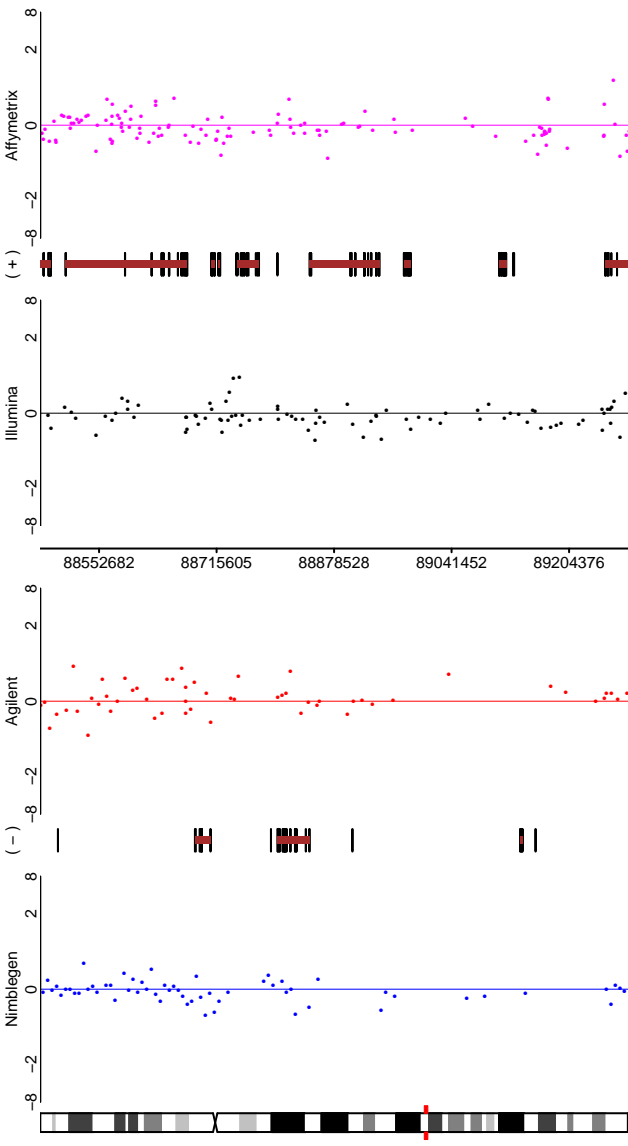

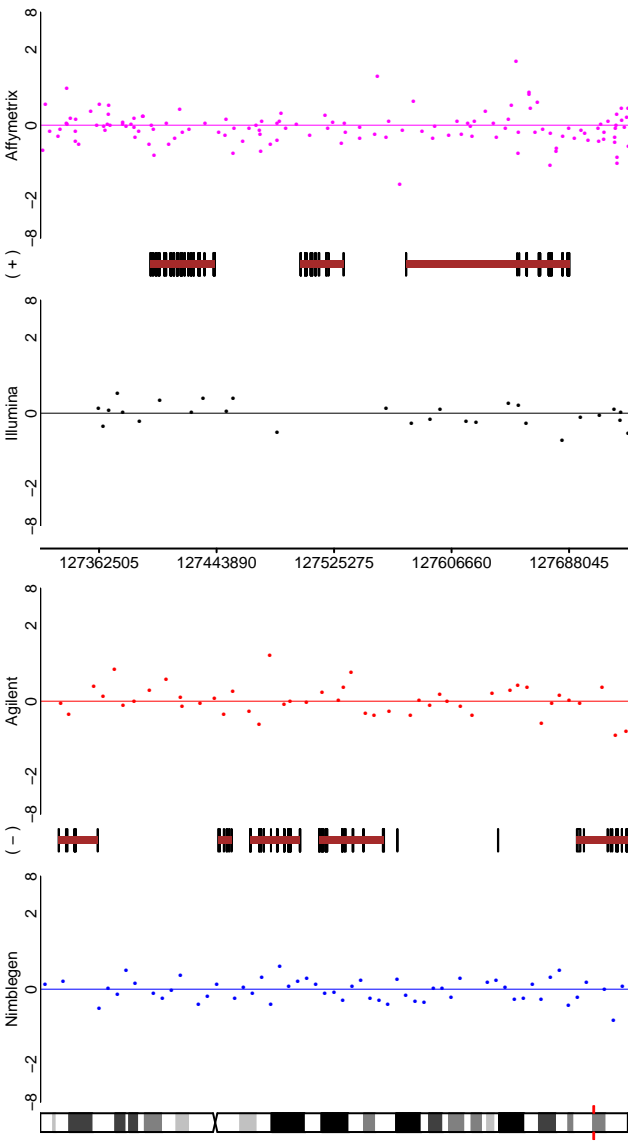

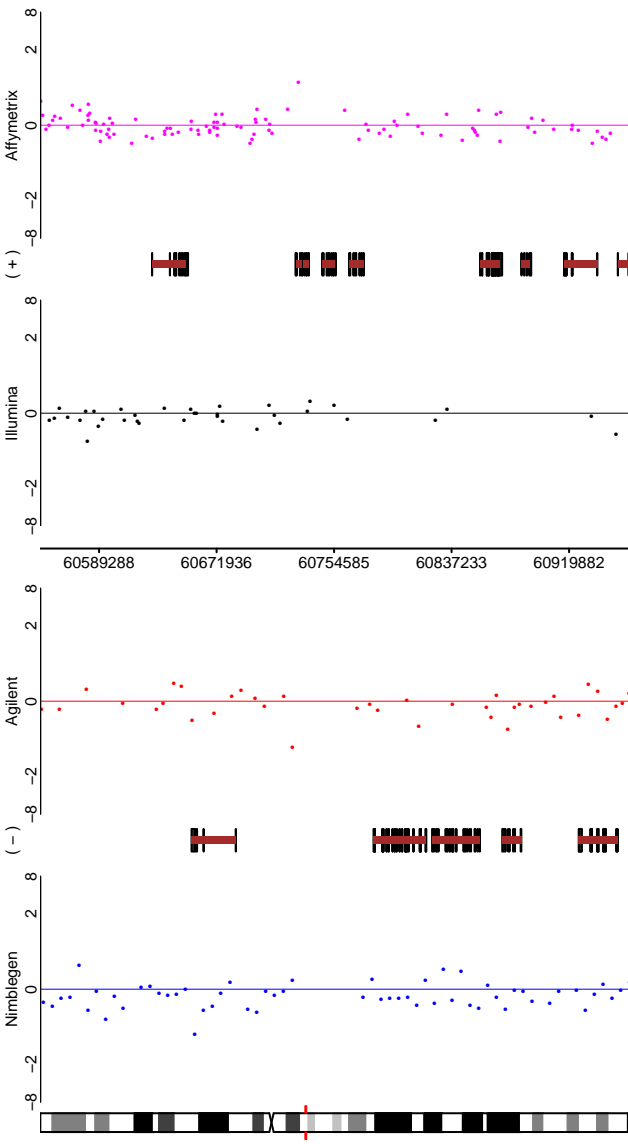

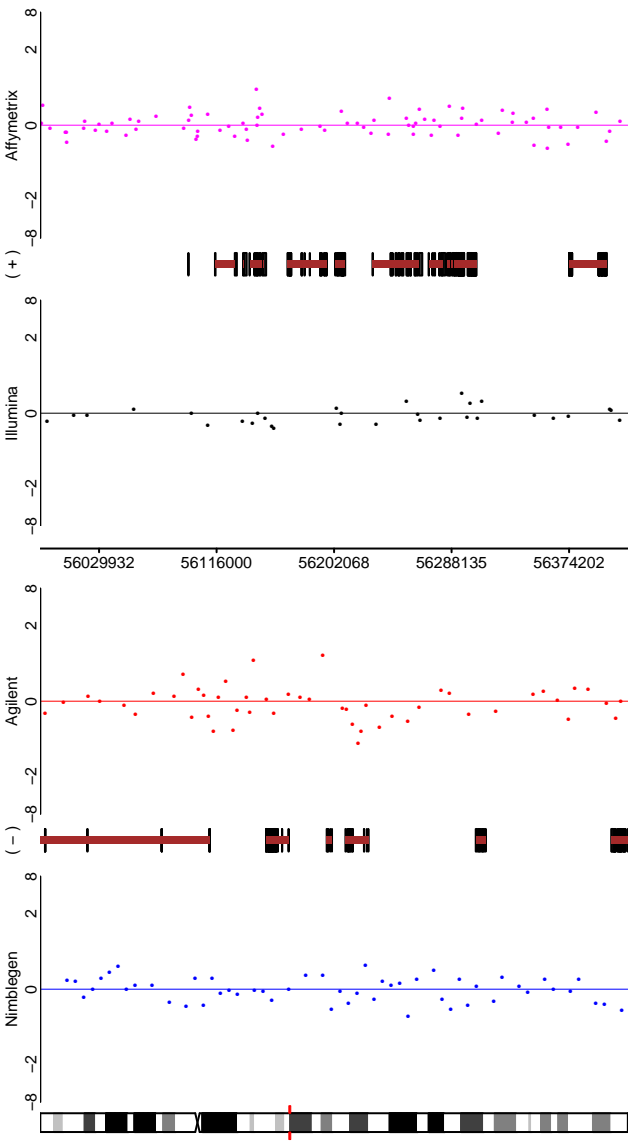

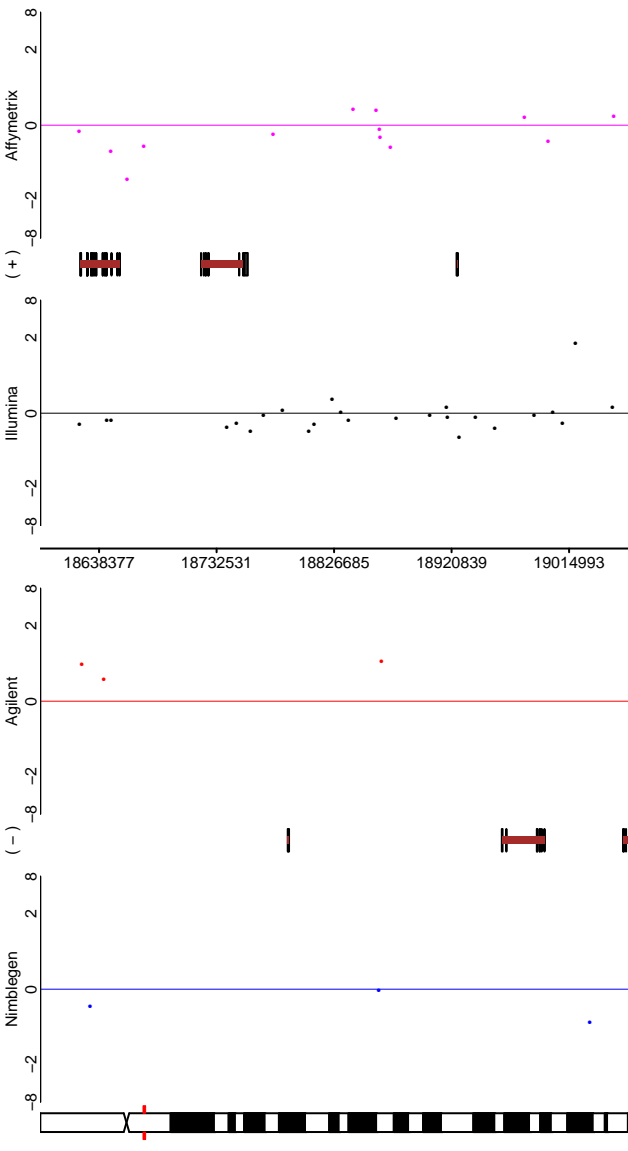

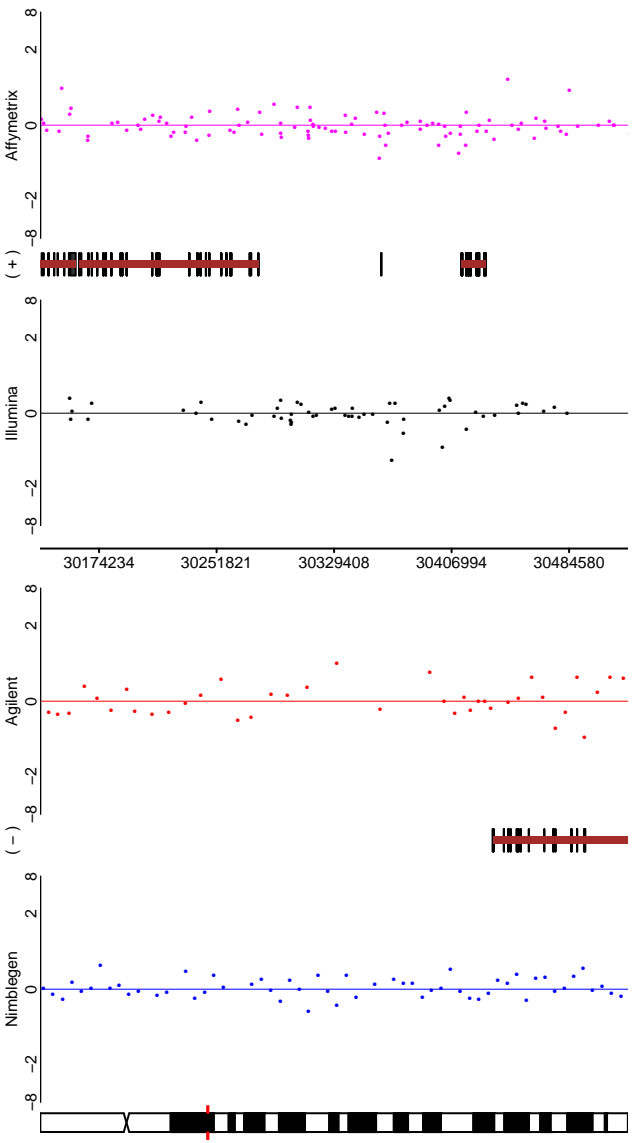

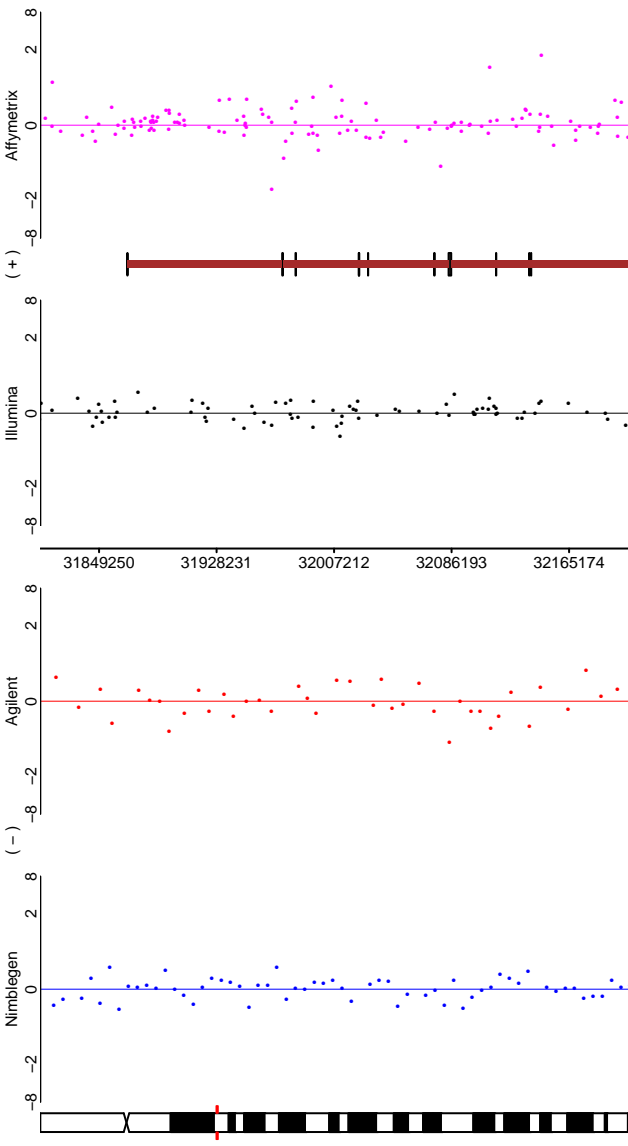

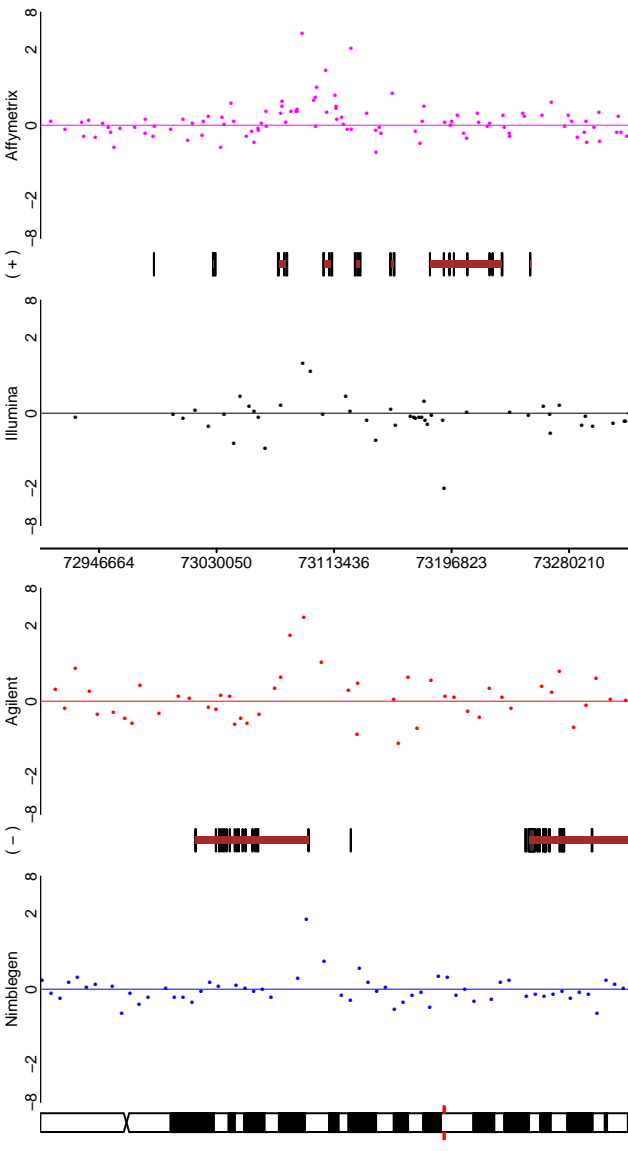

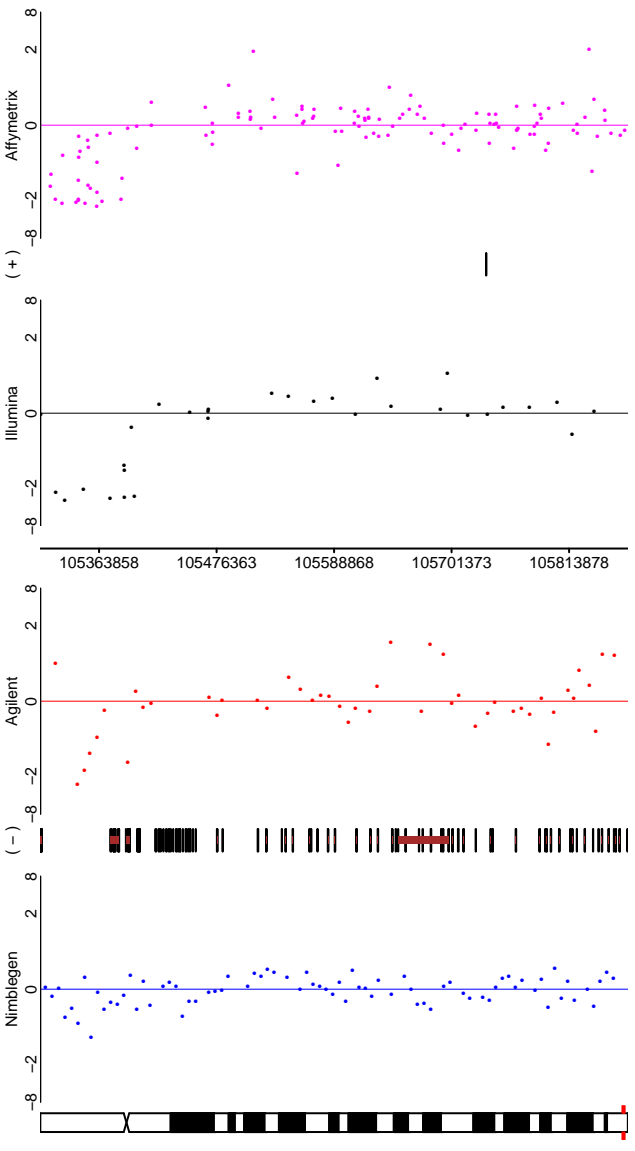

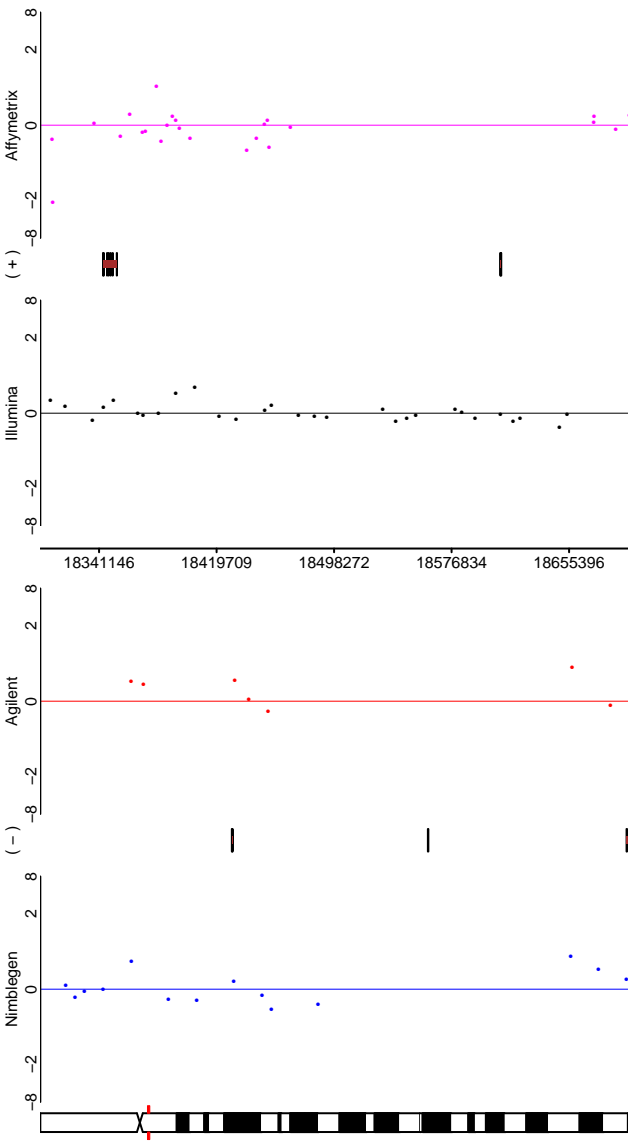

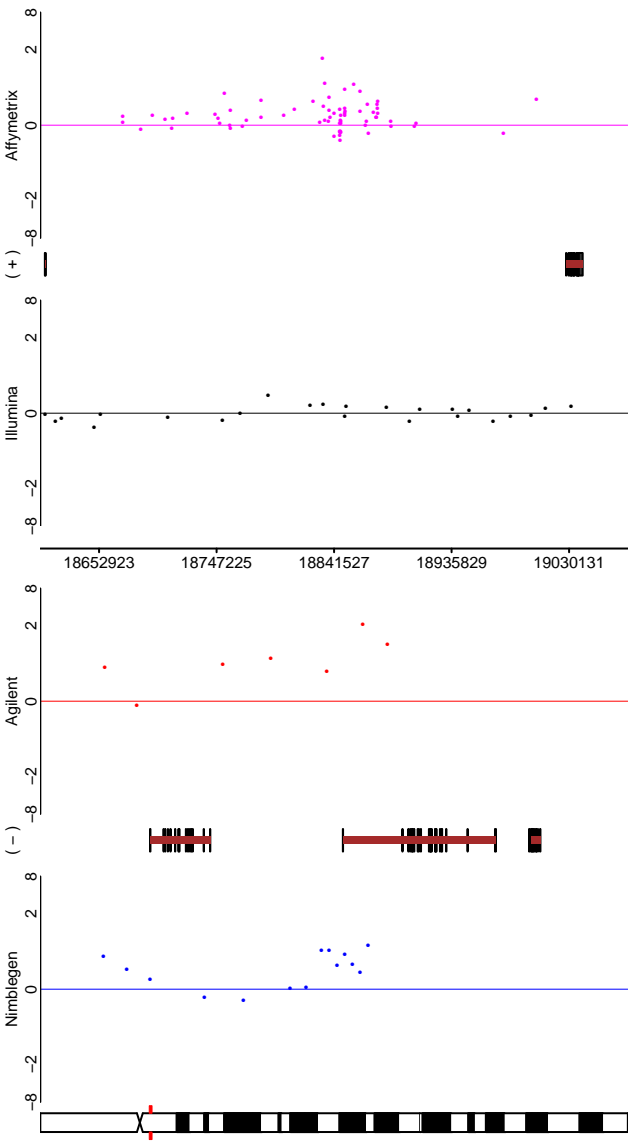

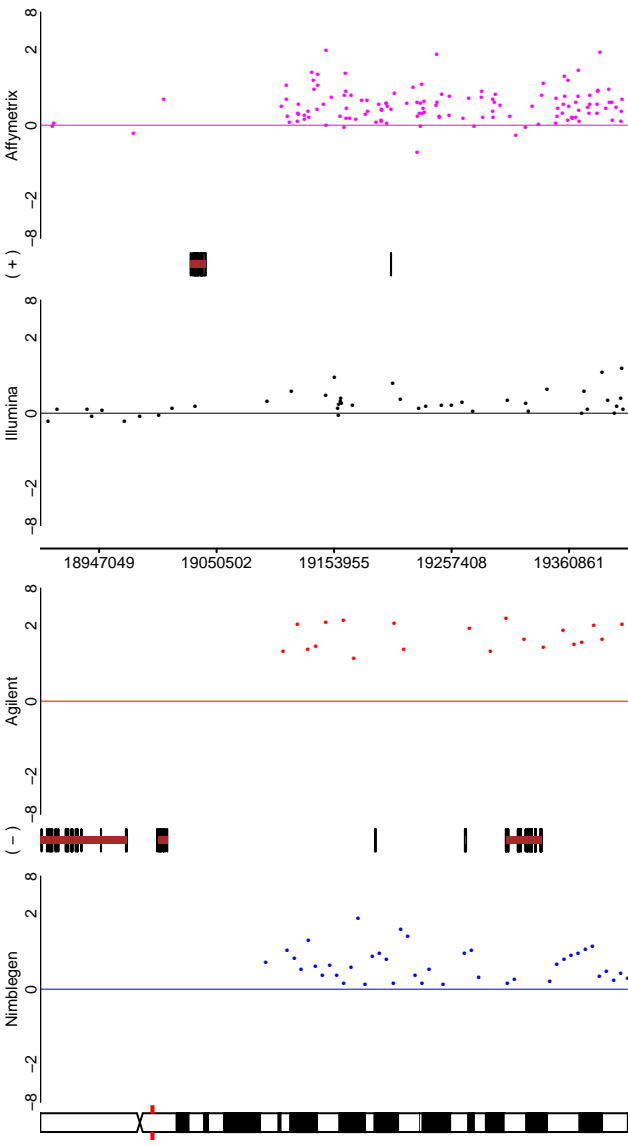

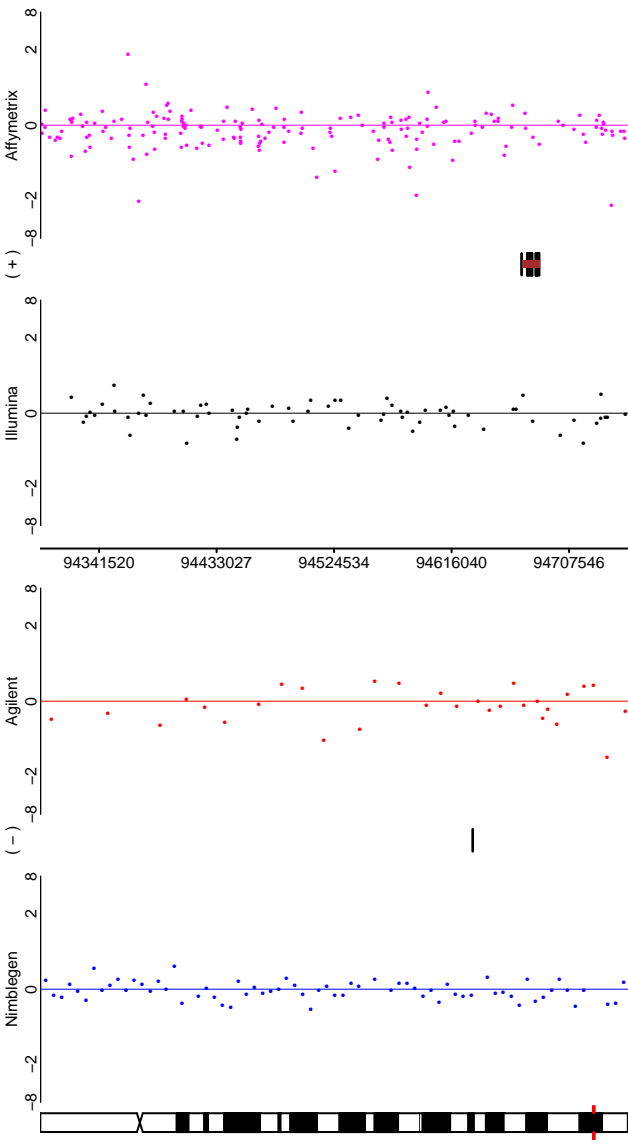

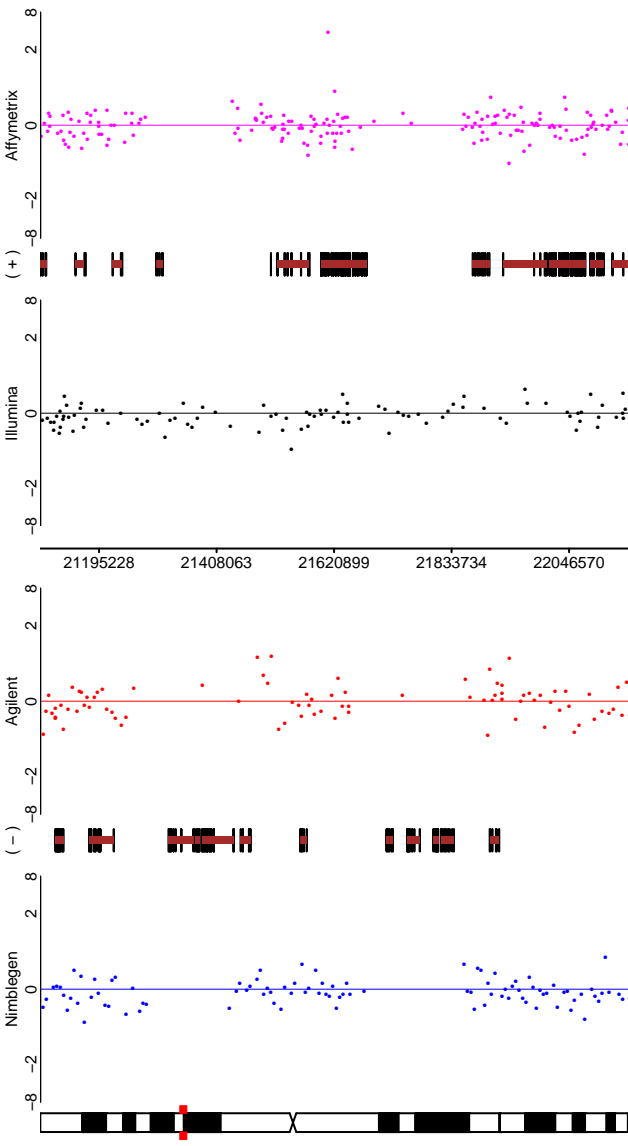

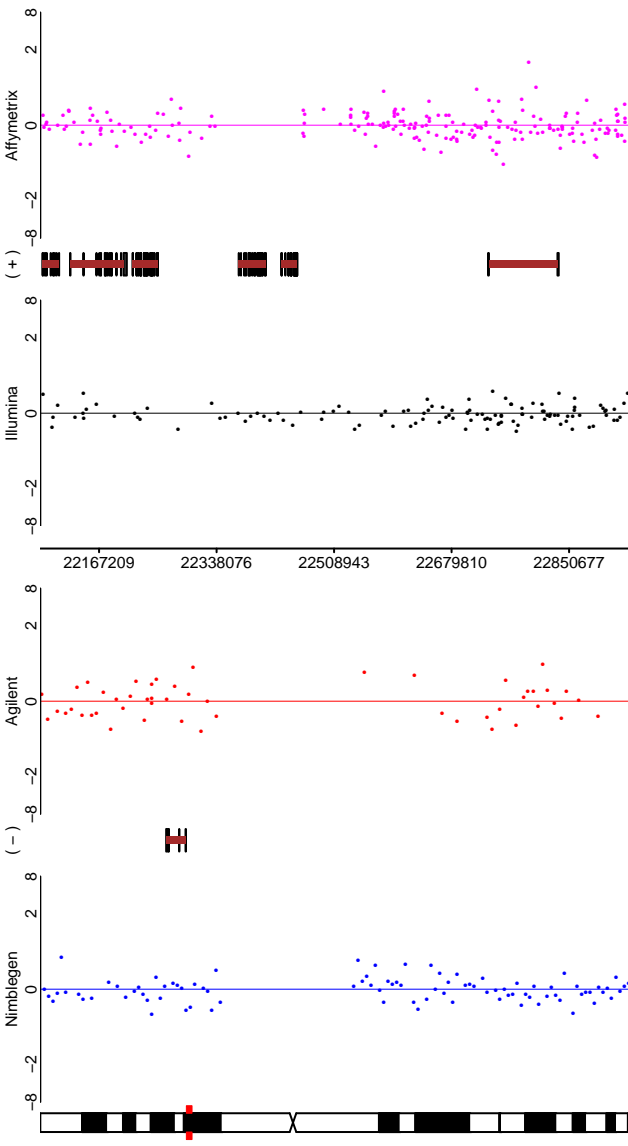

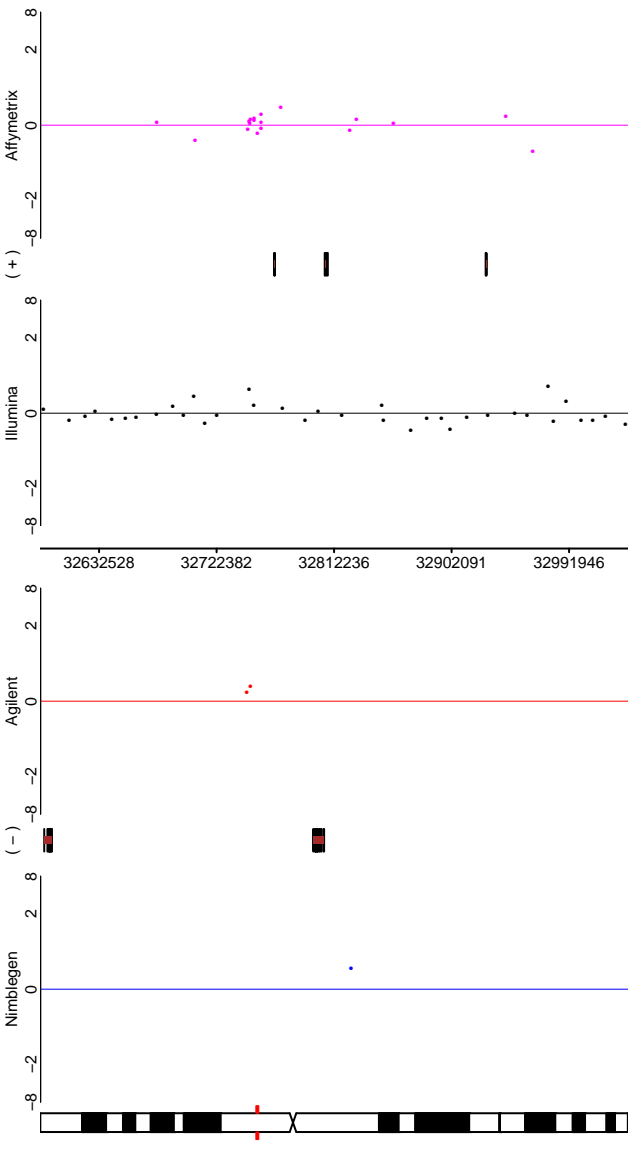

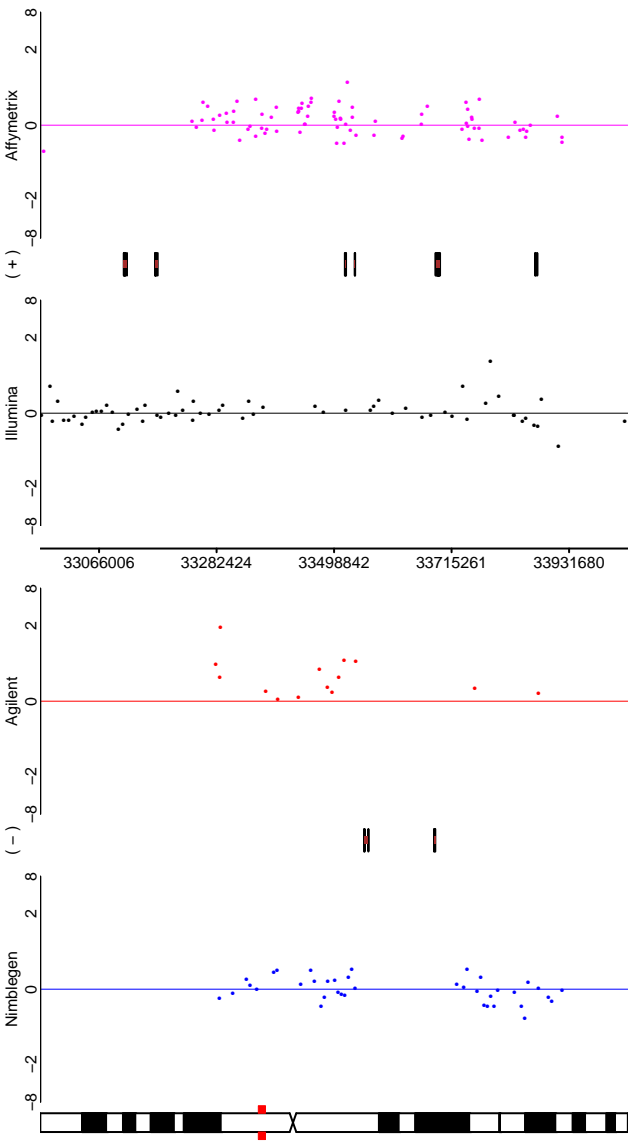

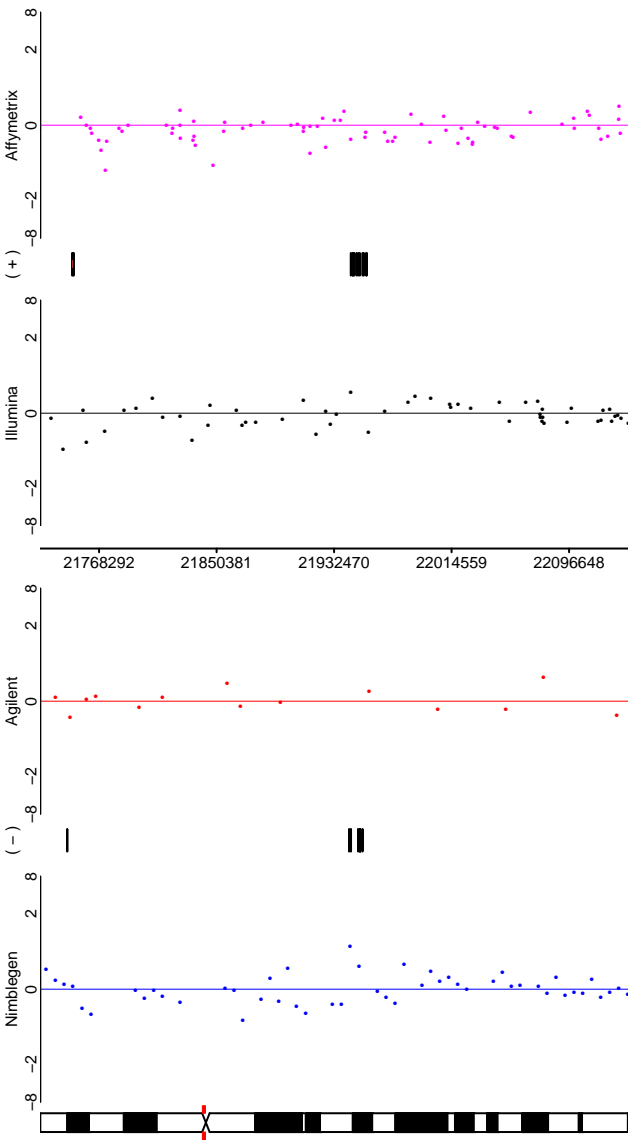

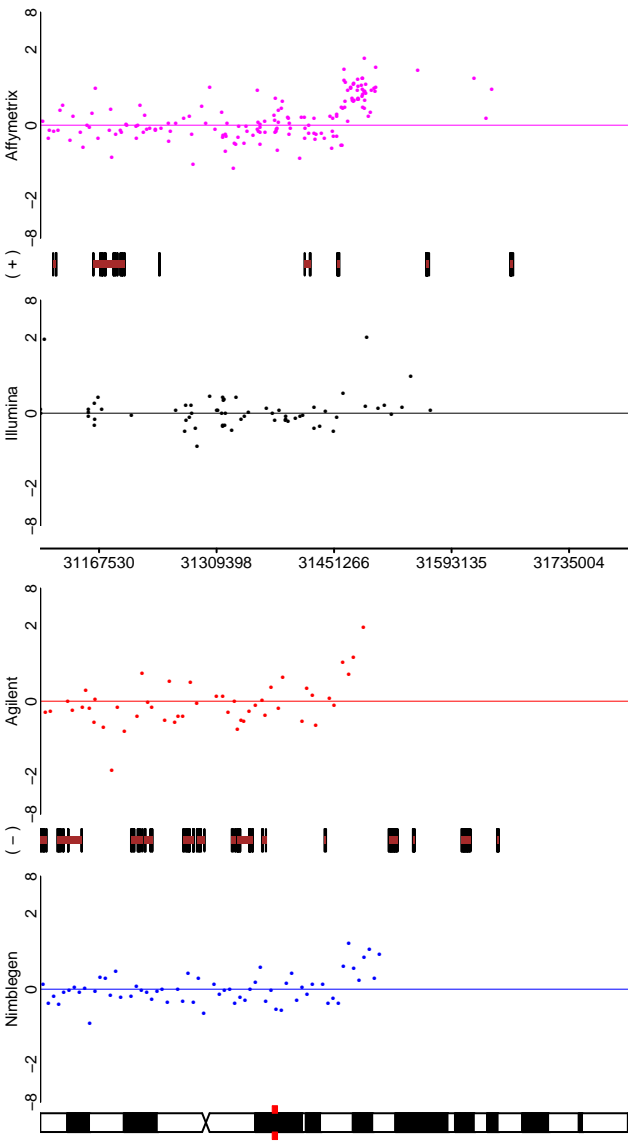

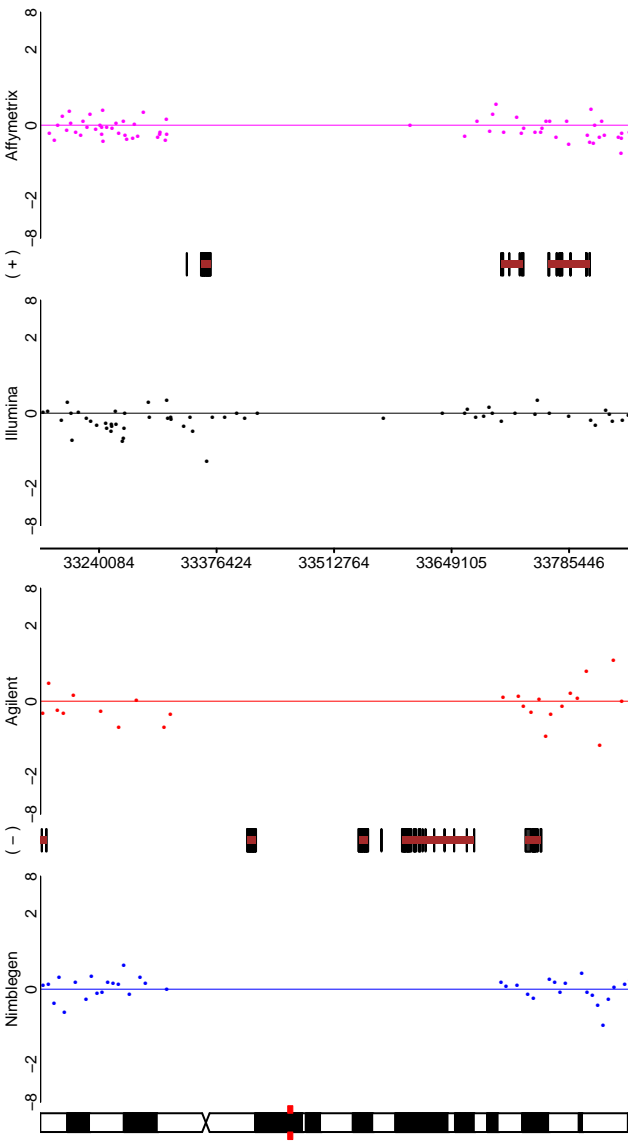

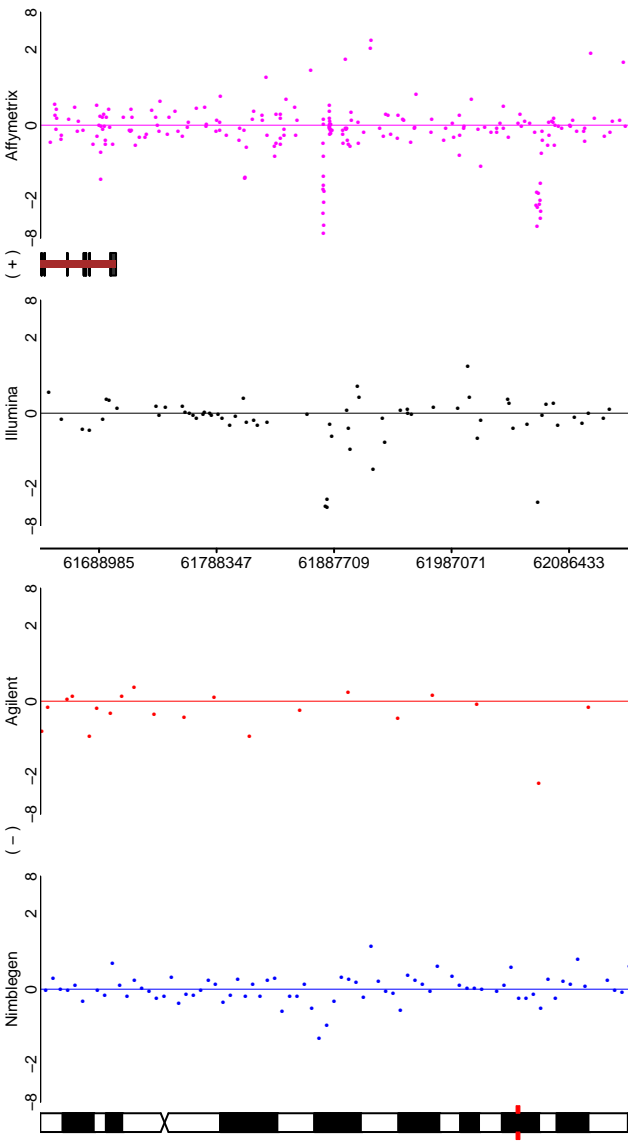

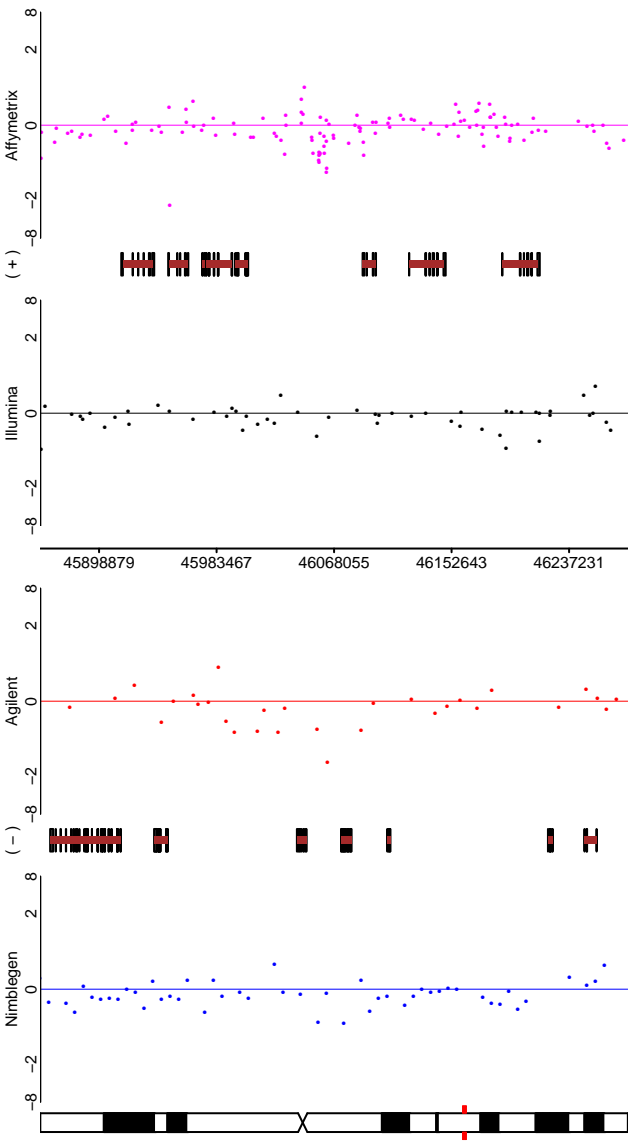

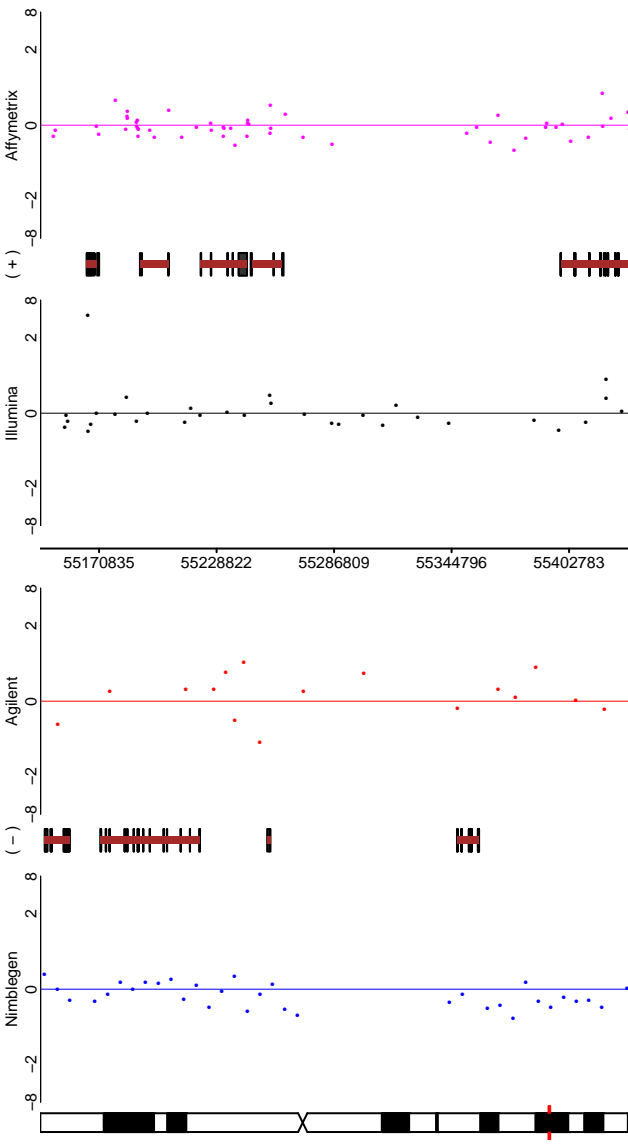

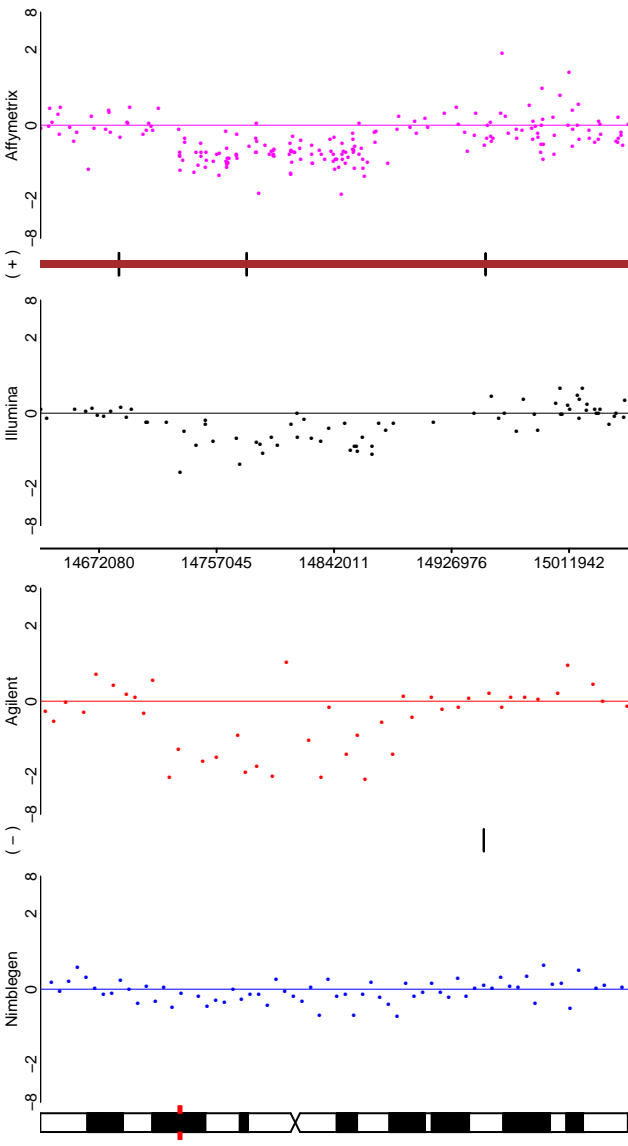

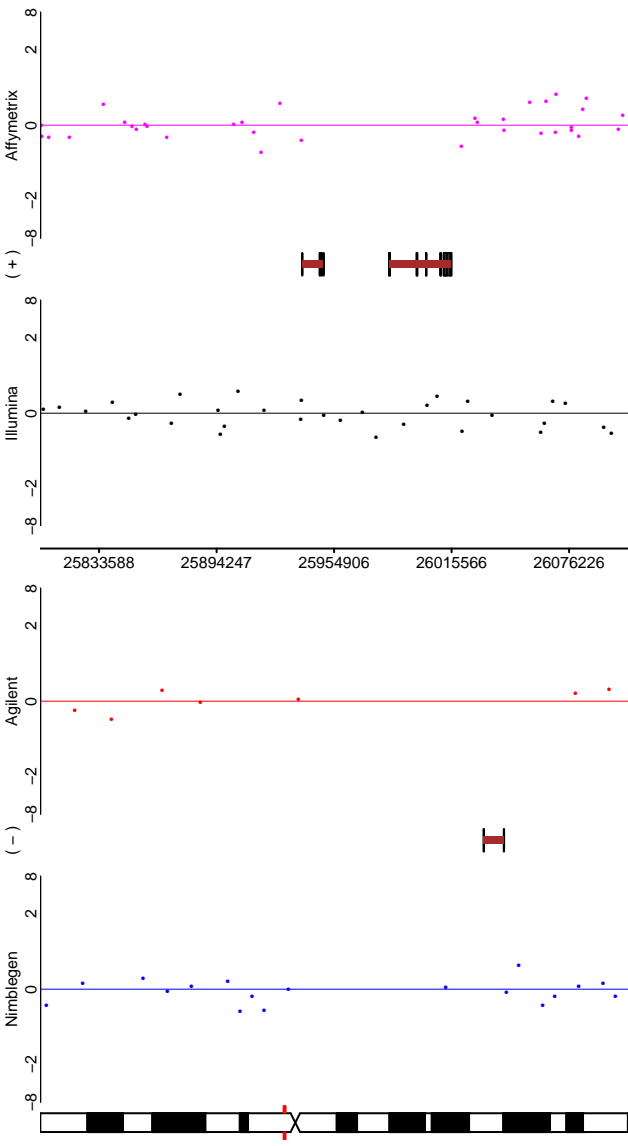

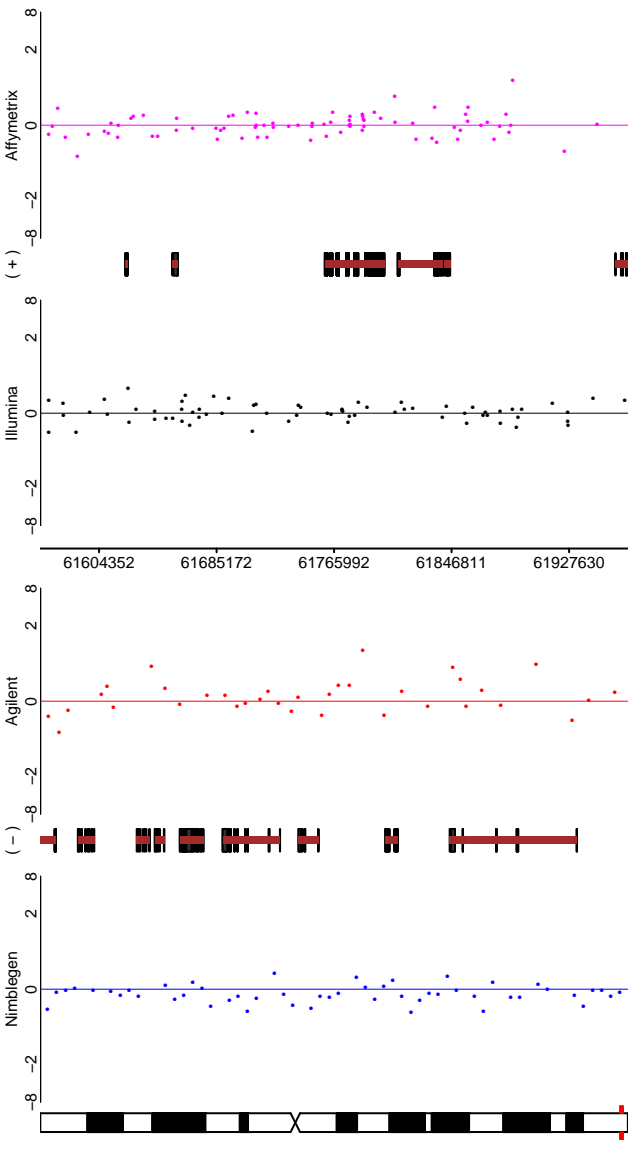

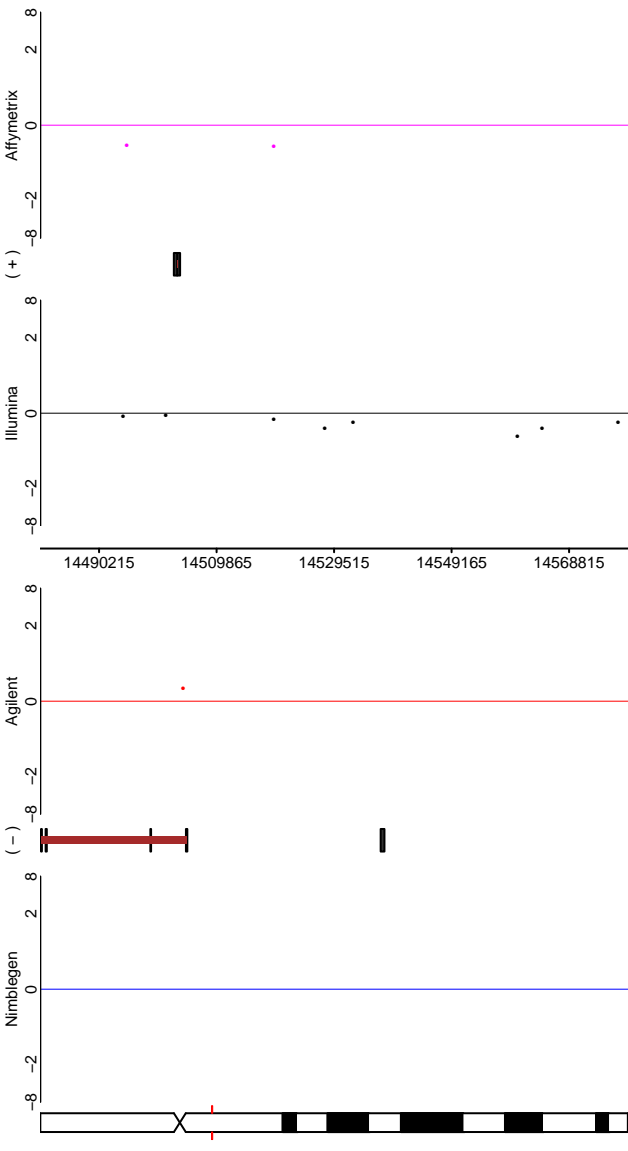

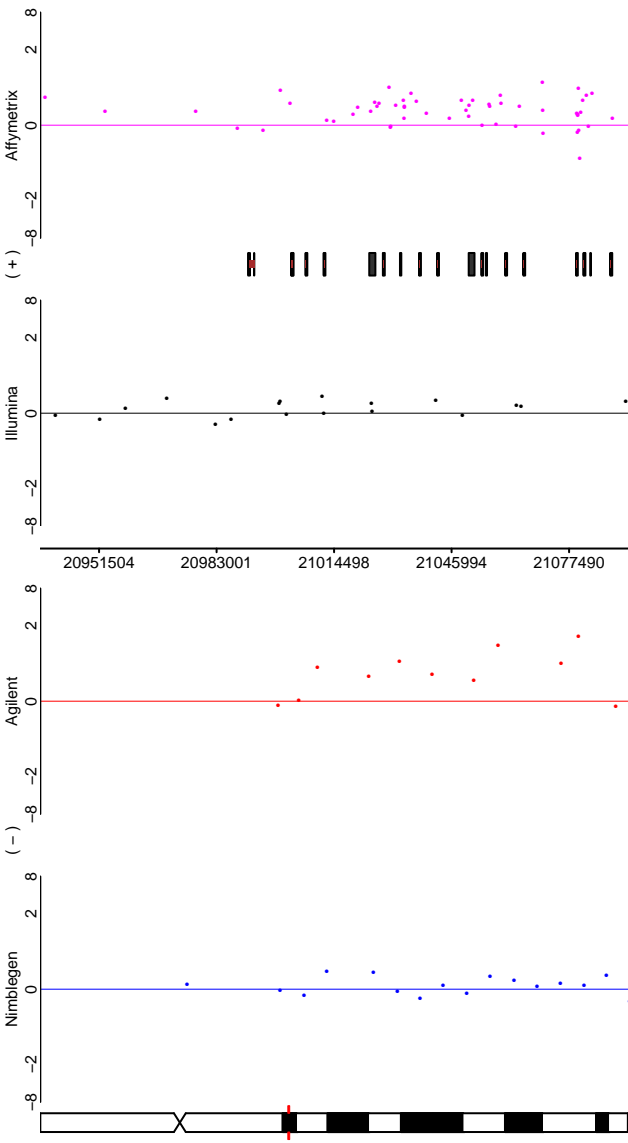

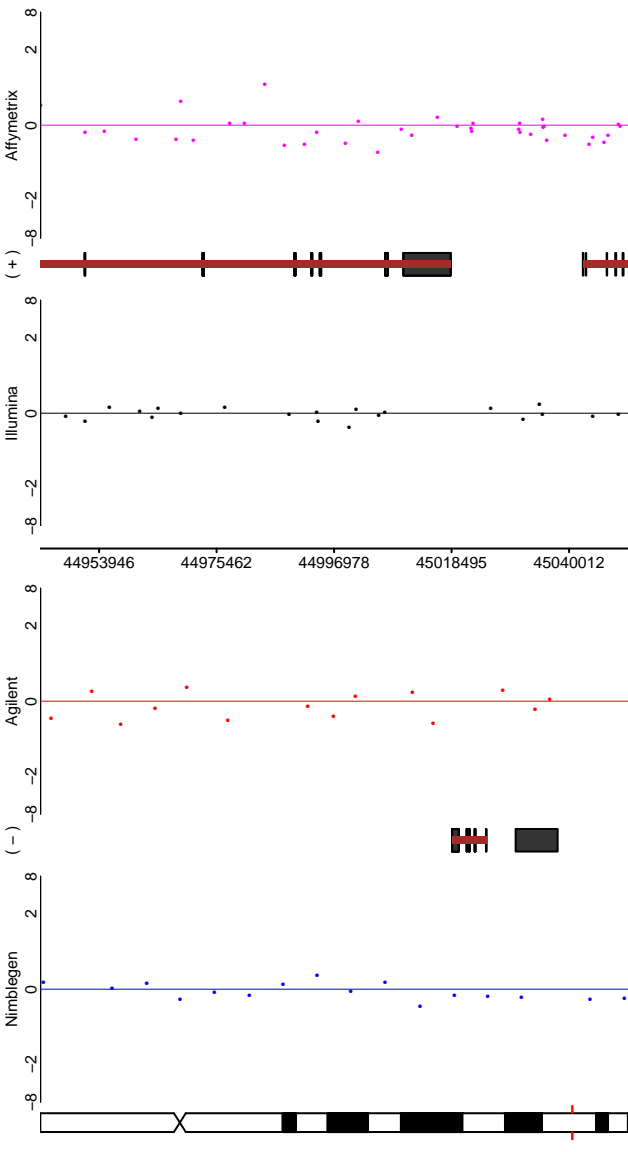

Supplement: Additional file 4 — Plots of the 79 CNV sites. Plots of the 79 sites of copy number difference between HapMap samples NA15510 and NA10851 (as listed in Additional File 3). [file 1471-2164-10-588-S4.PDF]

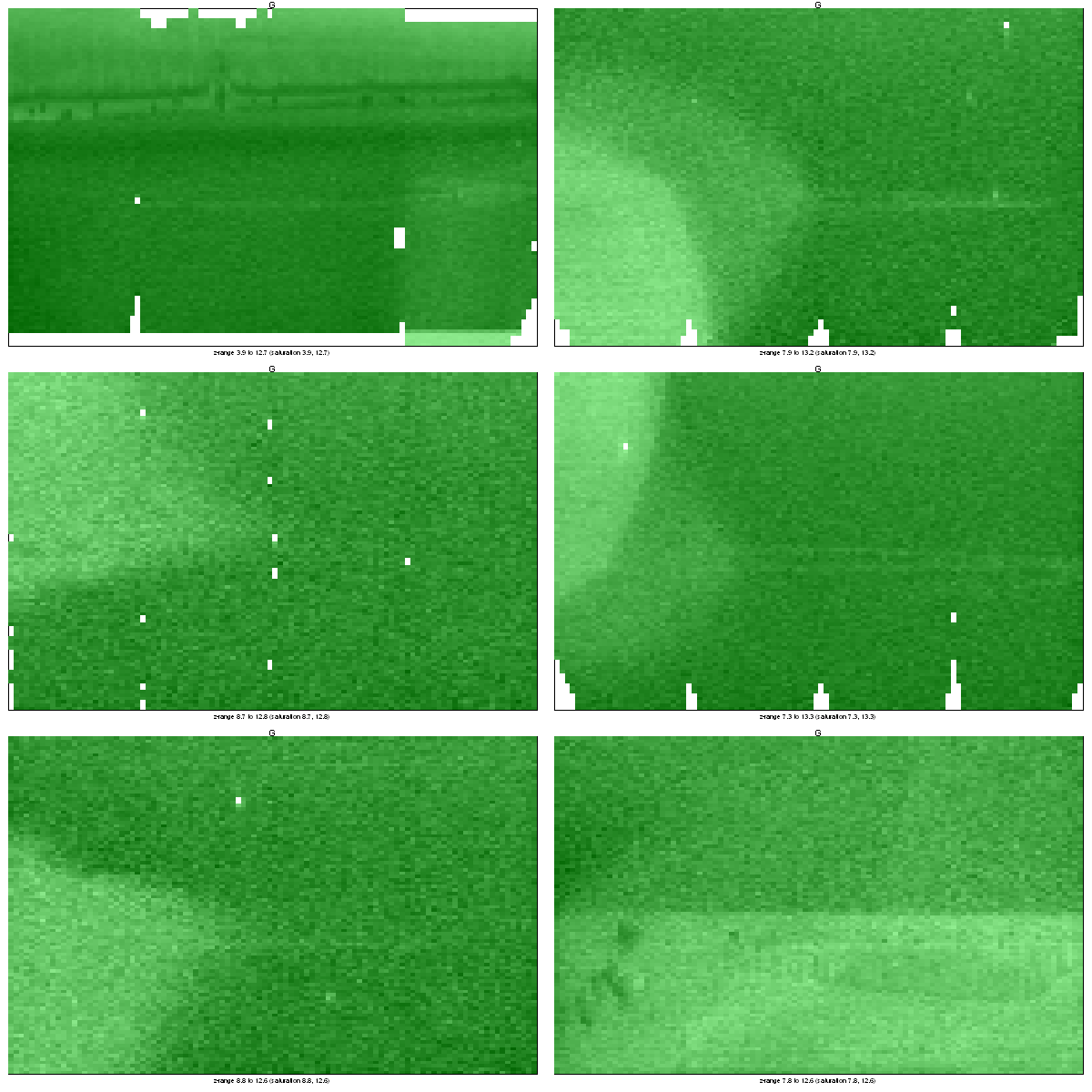

Supplement: Additional file 7 — Image plots of BASH processed Illumina data. False-colour image representation of six different raw images from the Illumina dataset that had significant spatial artefacts as identified using the BASH method from the beadarray Bioconductor package. As BeadStudio does not take spatial information into account during pre-processing, the resultant summarized values may be compromised in the presence of such artefacts. [file 1471-2164-10-588-S7.PNG]

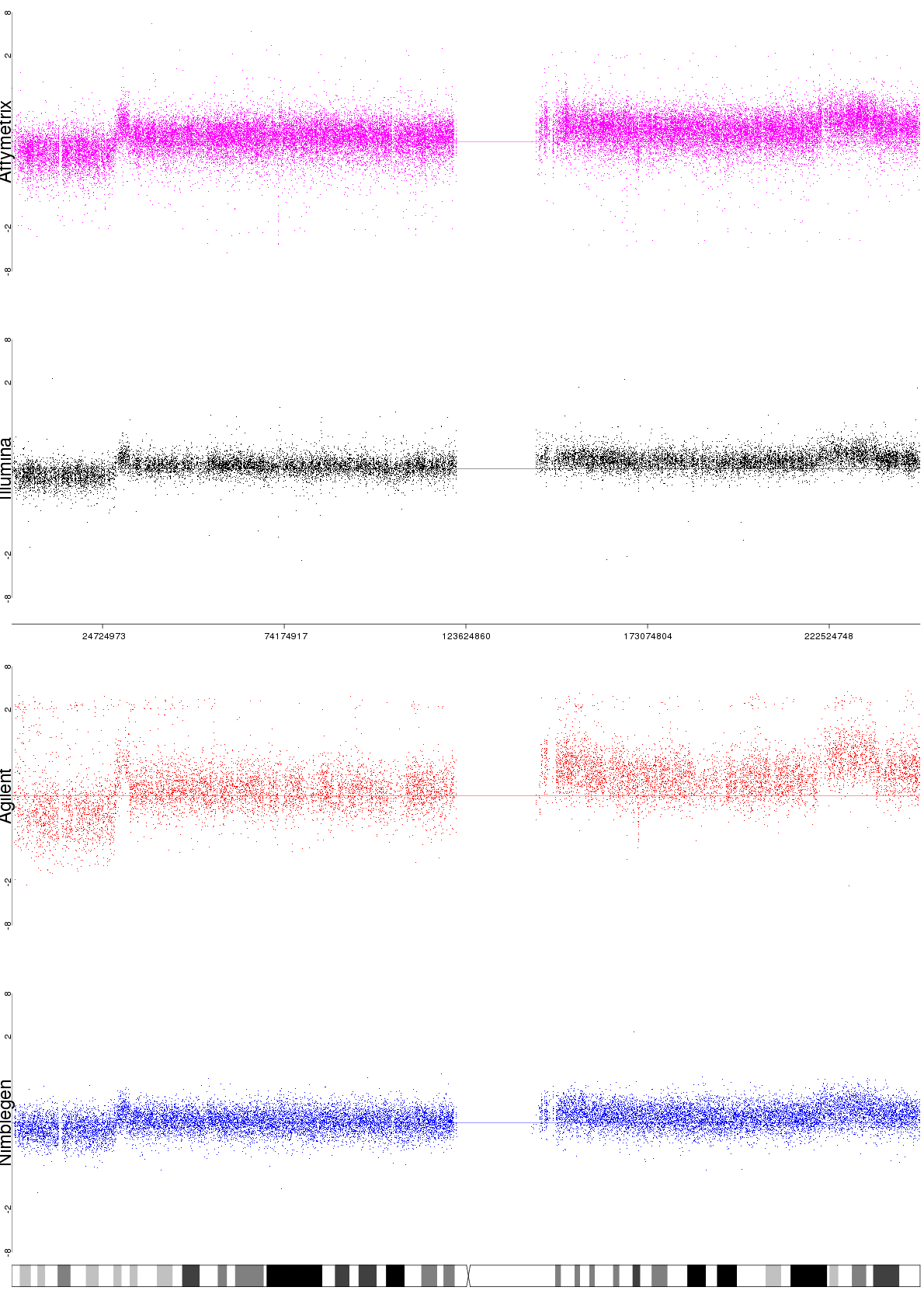

Supplement: Additional file 12 — All sample/chromosome plots for the tumours. Zip folder containing PNGs of all whole-chromosome plots for the tumours. [file 1471-2164-10-588-S12.ZIP › T7195/T7195 chromosome 1.png]

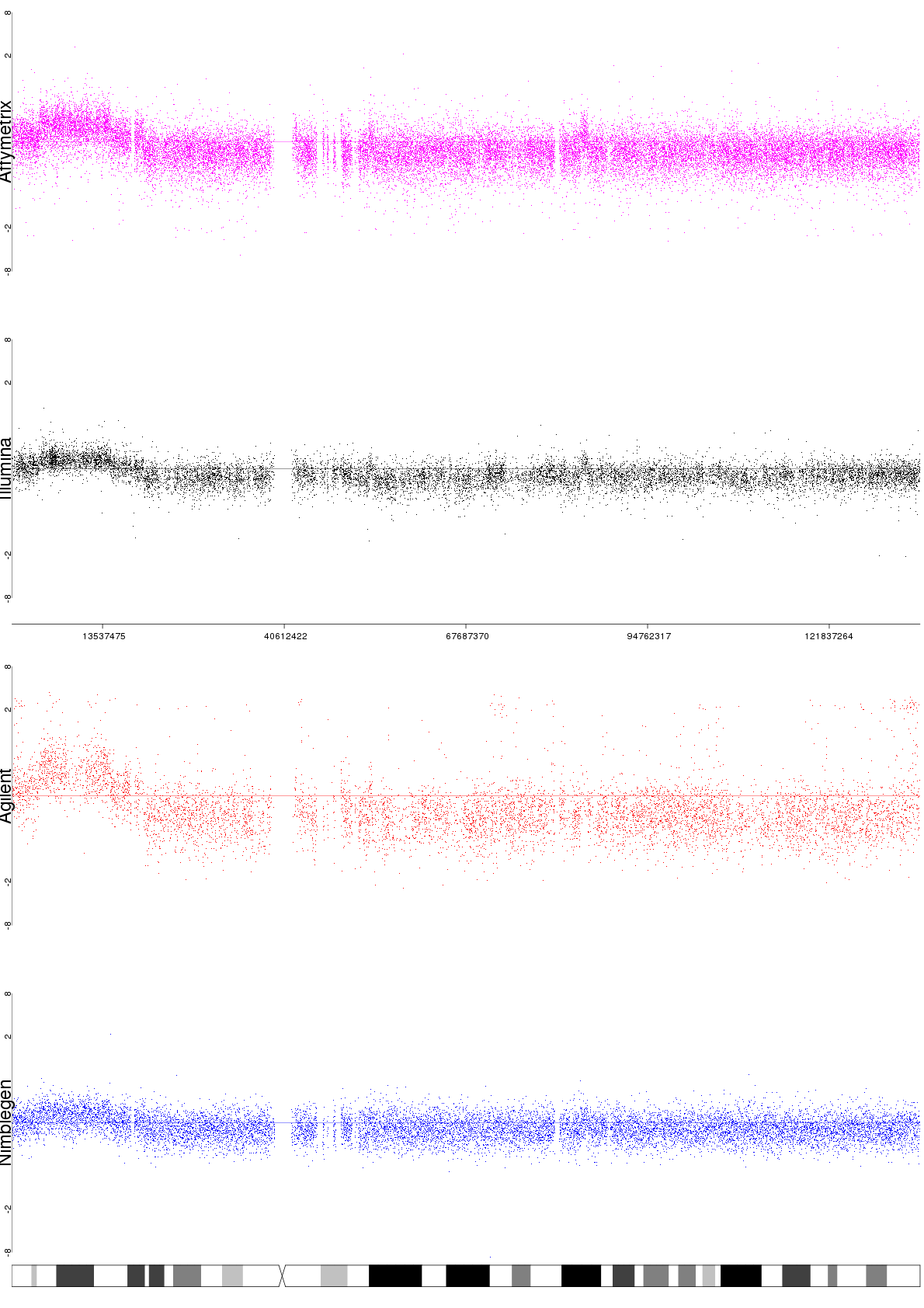

Supplement: Additional file 12 — All sample/chromosome plots for the tumours. Zip folder containing PNGs of all whole-chromosome plots for the tumours. [file 1471-2164-10-588-S12.ZIP › T7195/T7195 chromosome 10.png]

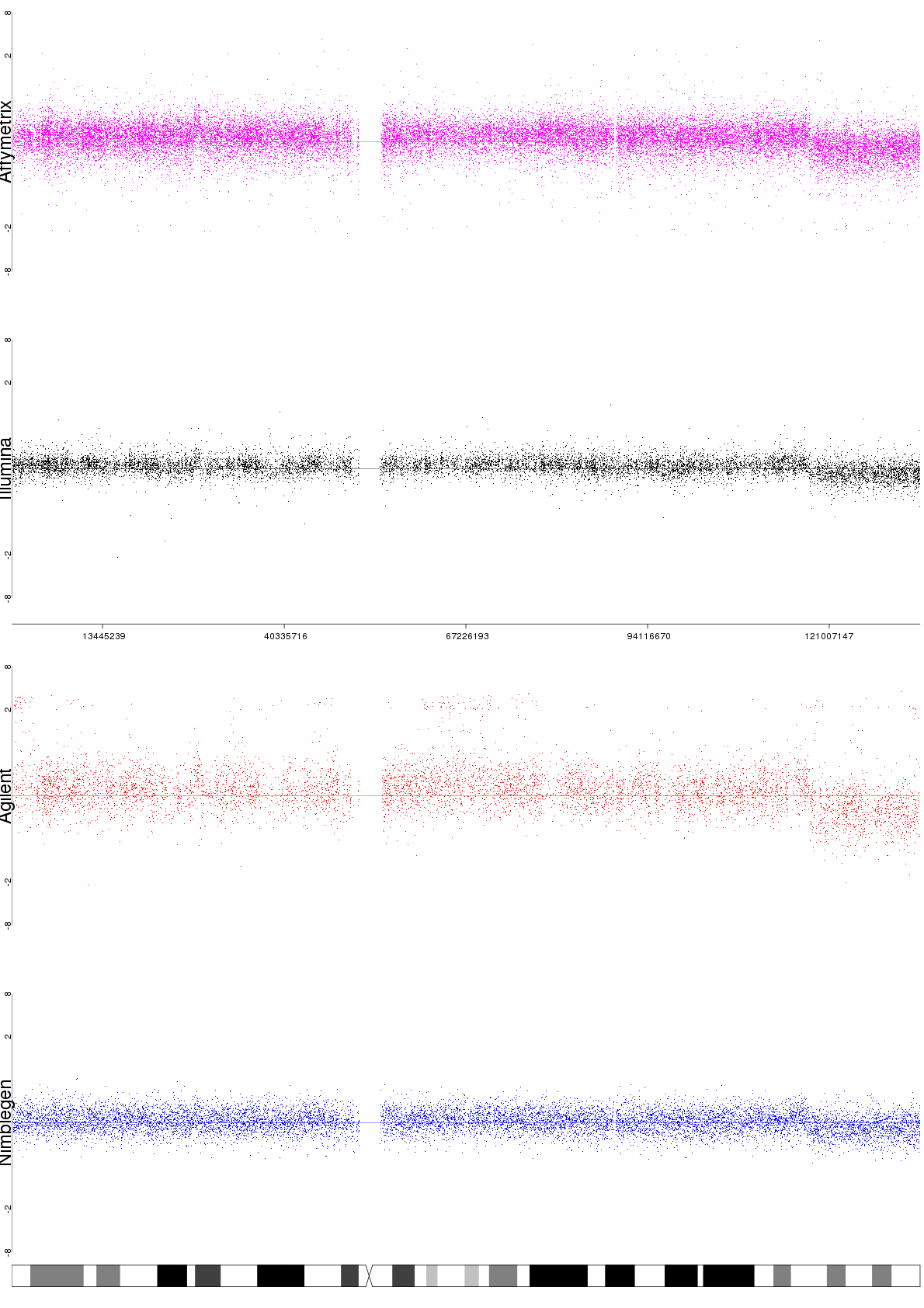

Supplement: Additional file 12 — All sample/chromosome plots for the tumours. Zip folder containing PNGs of all whole-chromosome plots for the tumours. [file 1471-2164-10-588-S12.ZIP › T7195/T7195 chromosome 11.png]

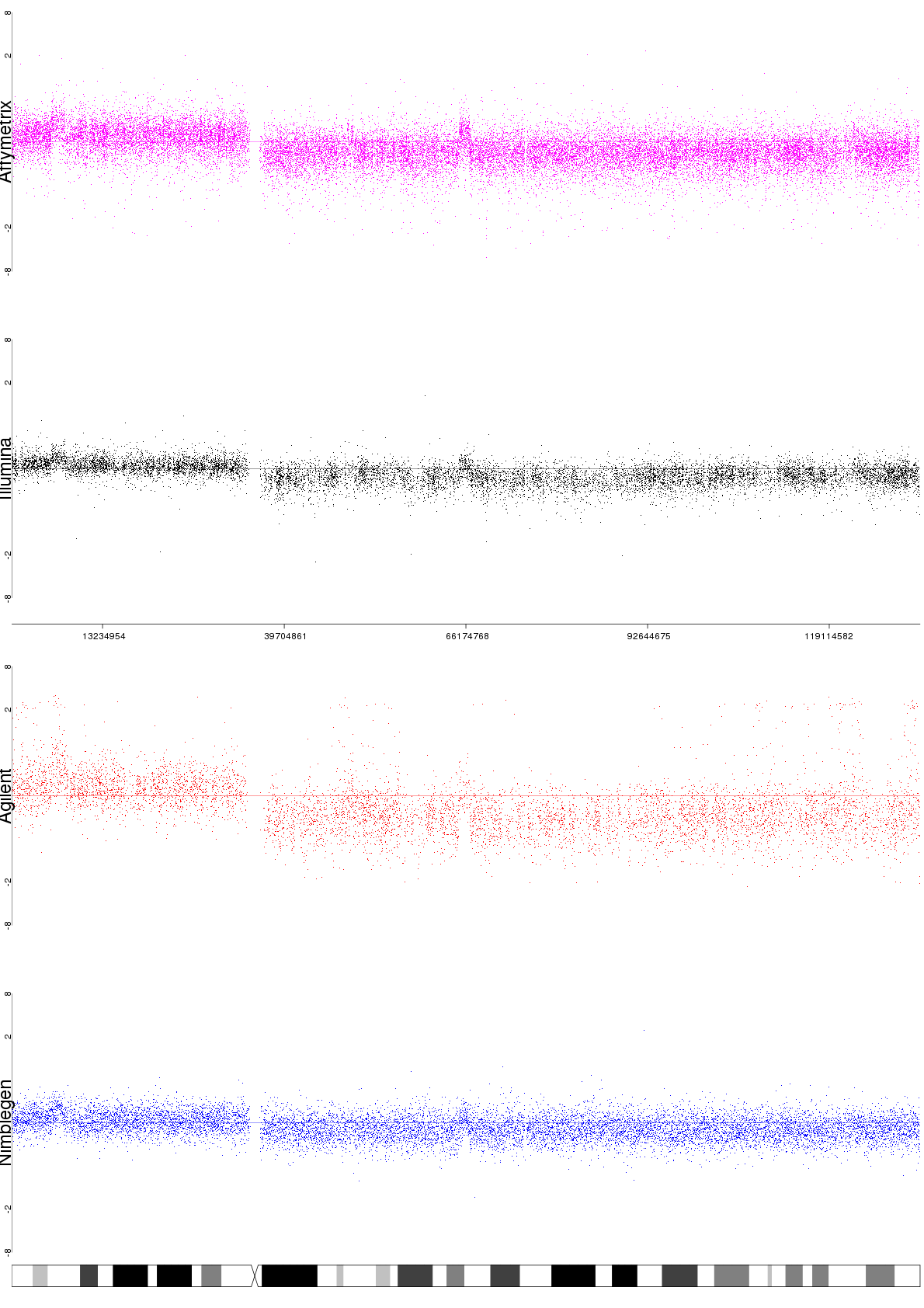

Supplement: Additional file 12 — All sample/chromosome plots for the tumours. Zip folder containing PNGs of all whole-chromosome plots for the tumours. [file 1471-2164-10-588-S12.ZIP › T7195/T7195 chromosome 12.png]

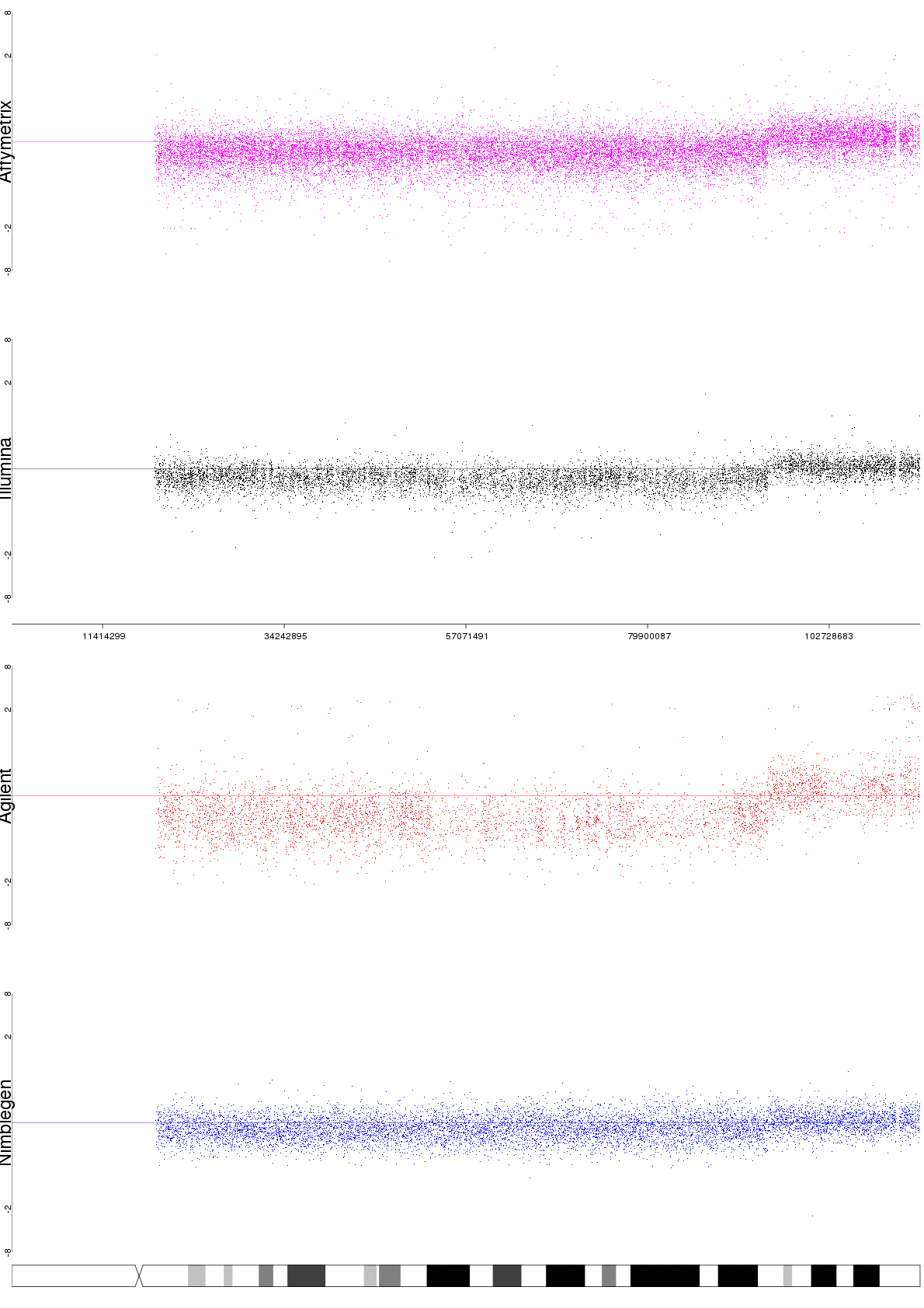

Supplement: Additional file 12 — All sample/chromosome plots for the tumours. Zip folder containing PNGs of all whole-chromosome plots for the tumours. [file 1471-2164-10-588-S12.ZIP › T7195/T7195 chromosome 13.png]

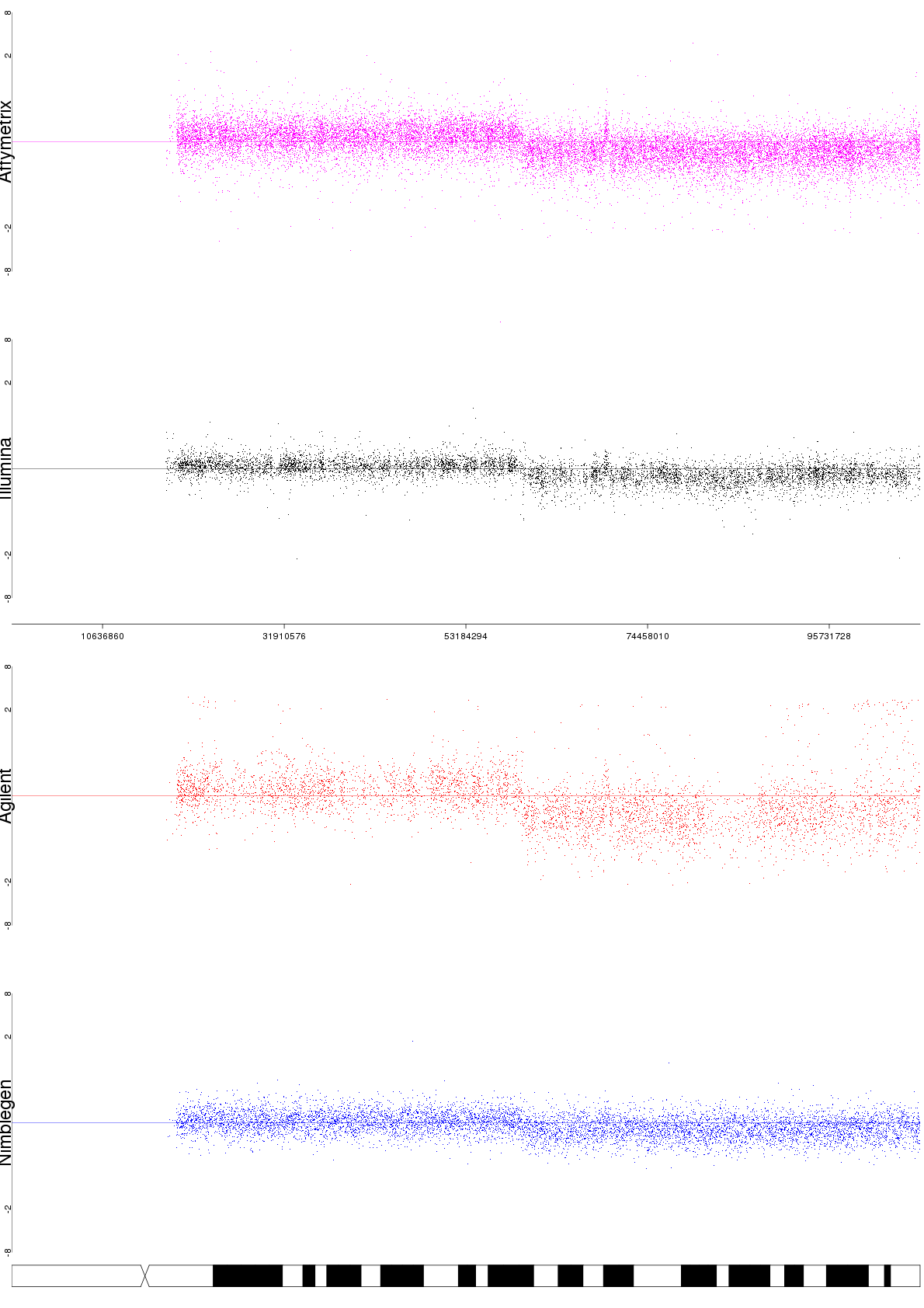

Supplement: Additional file 12 — All sample/chromosome plots for the tumours. Zip folder containing PNGs of all whole-chromosome plots for the tumours. [file 1471-2164-10-588-S12.ZIP › T7195/T7195 chromosome 14.png]

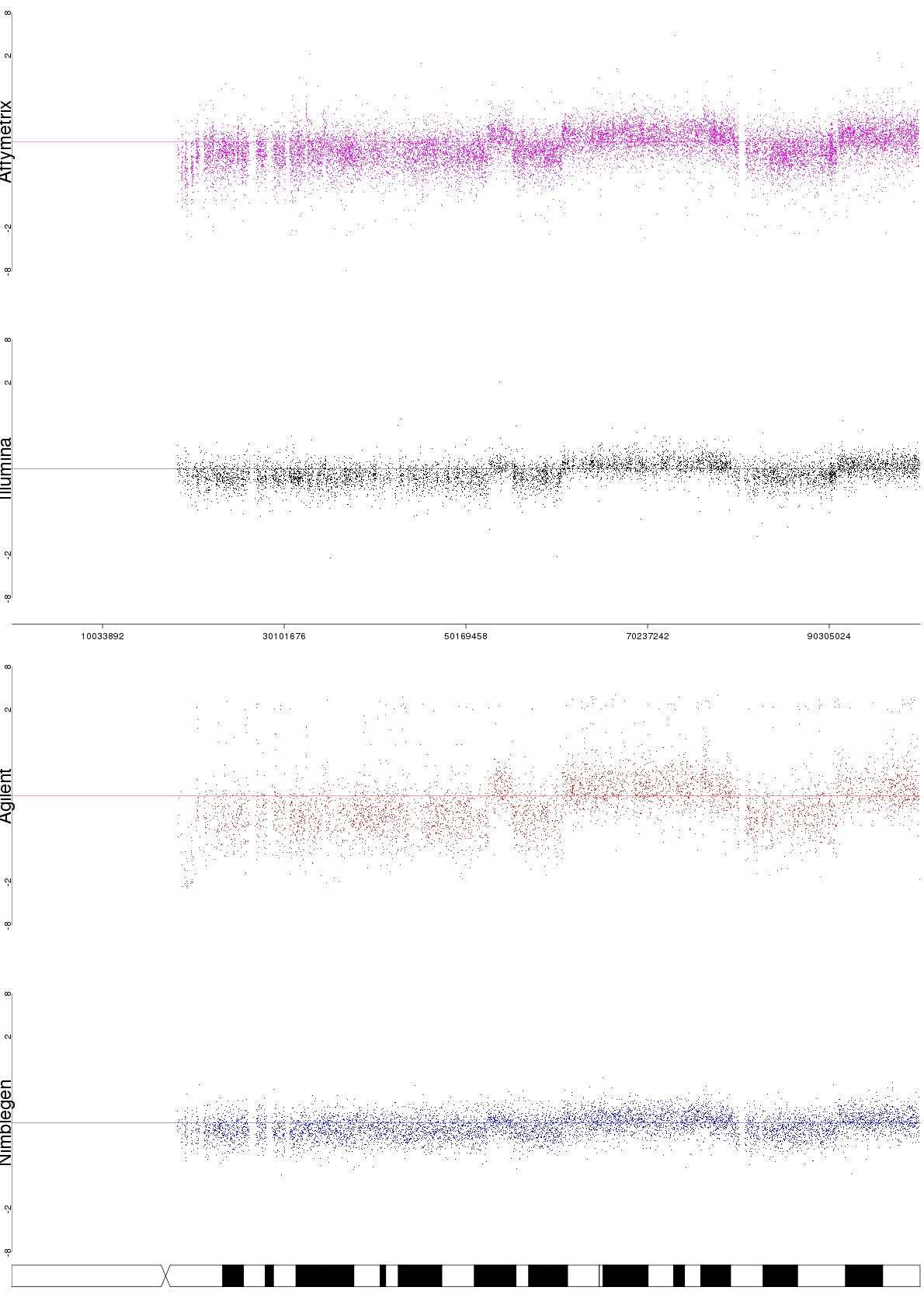

Supplement: Additional file 12 — All sample/chromosome plots for the tumours. Zip folder containing PNGs of all whole-chromosome plots for the tumours. [file 1471-2164-10-588-S12.ZIP › T7195/T7195 chromosome 15.png]

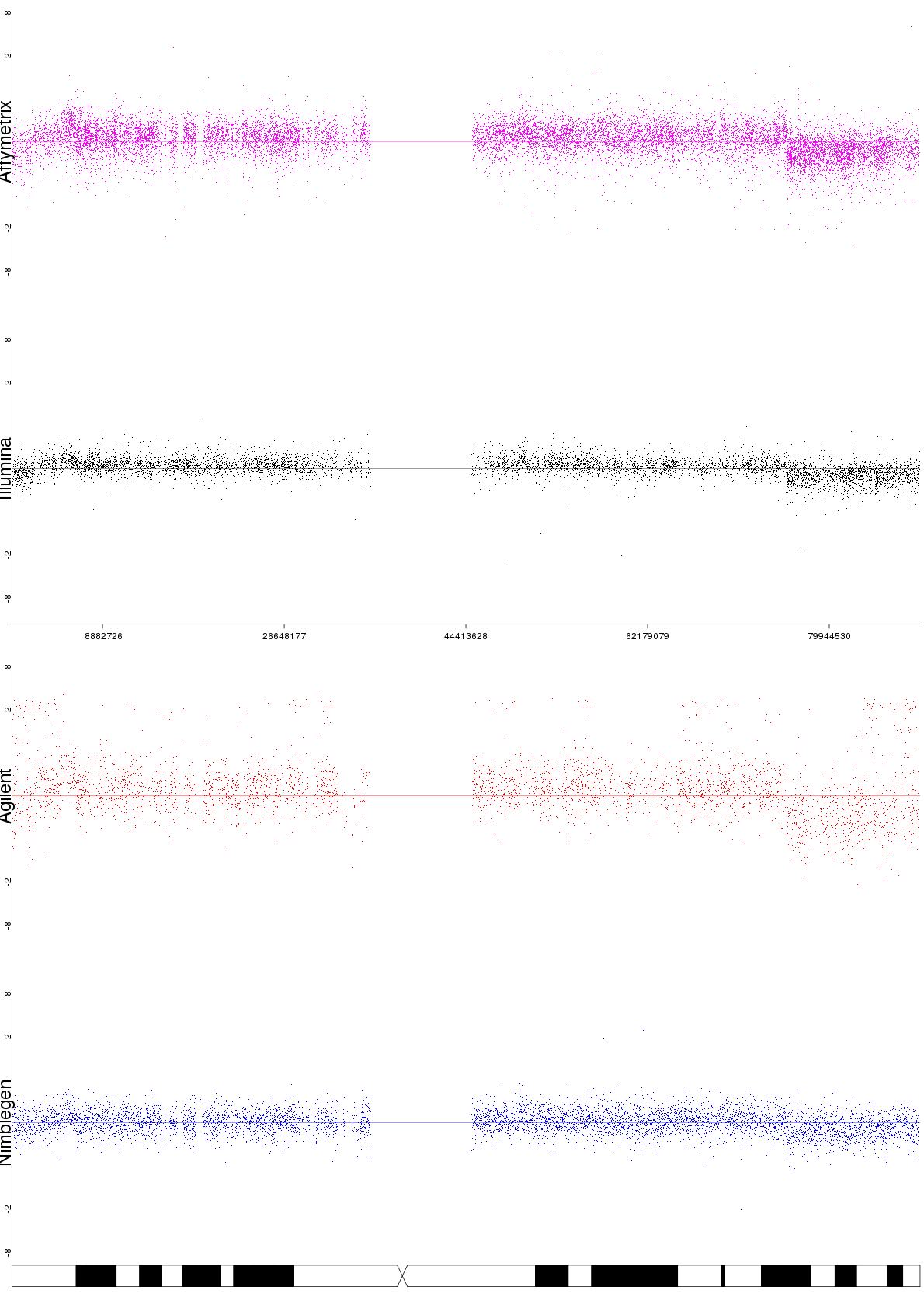

Supplement: Additional file 12 — All sample/chromosome plots for the tumours. Zip folder containing PNGs of all whole-chromosome plots for the tumours. [file 1471-2164-10-588-S12.ZIP › T7195/T7195 chromosome 16.png]

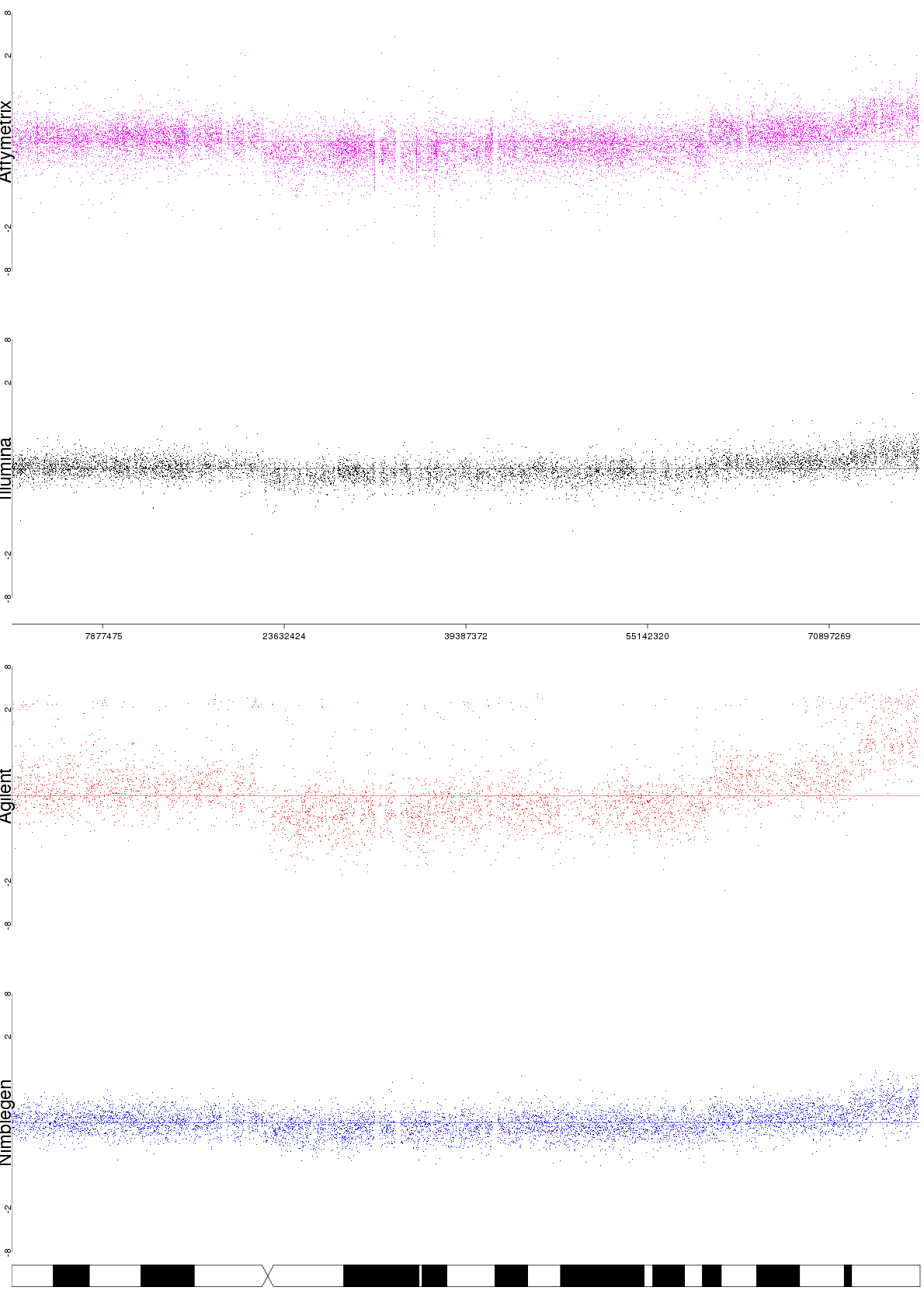

Supplement: Additional file 12 — All sample/chromosome plots for the tumours. Zip folder containing PNGs of all whole-chromosome plots for the tumours. [file 1471-2164-10-588-S12.ZIP › T7195/T7195 chromosome 17.png]

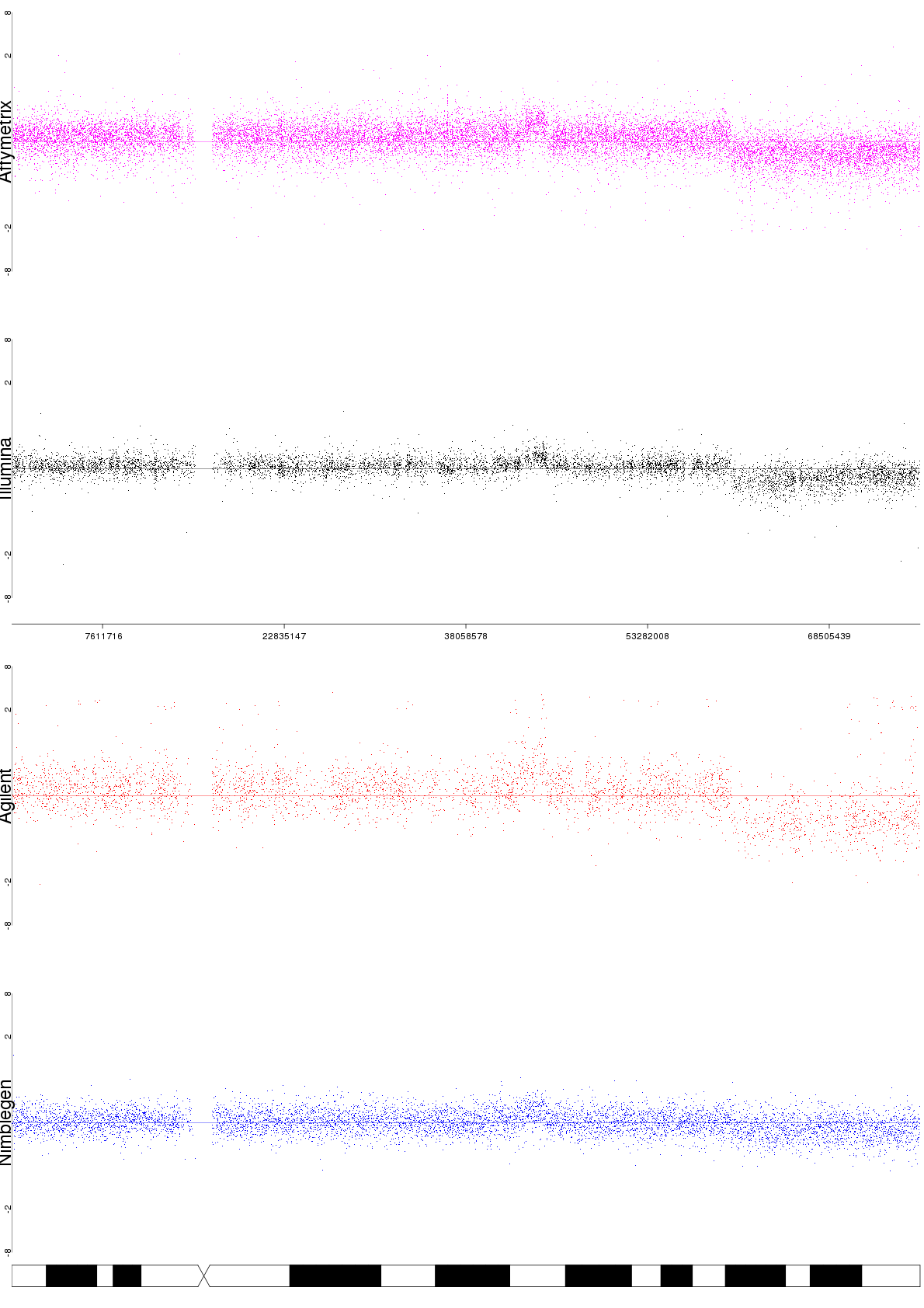

Supplement: Additional file 12 — All sample/chromosome plots for the tumours. Zip folder containing PNGs of all whole-chromosome plots for the tumours. [file 1471-2164-10-588-S12.ZIP › T7195/T7195 chromosome 18.png]

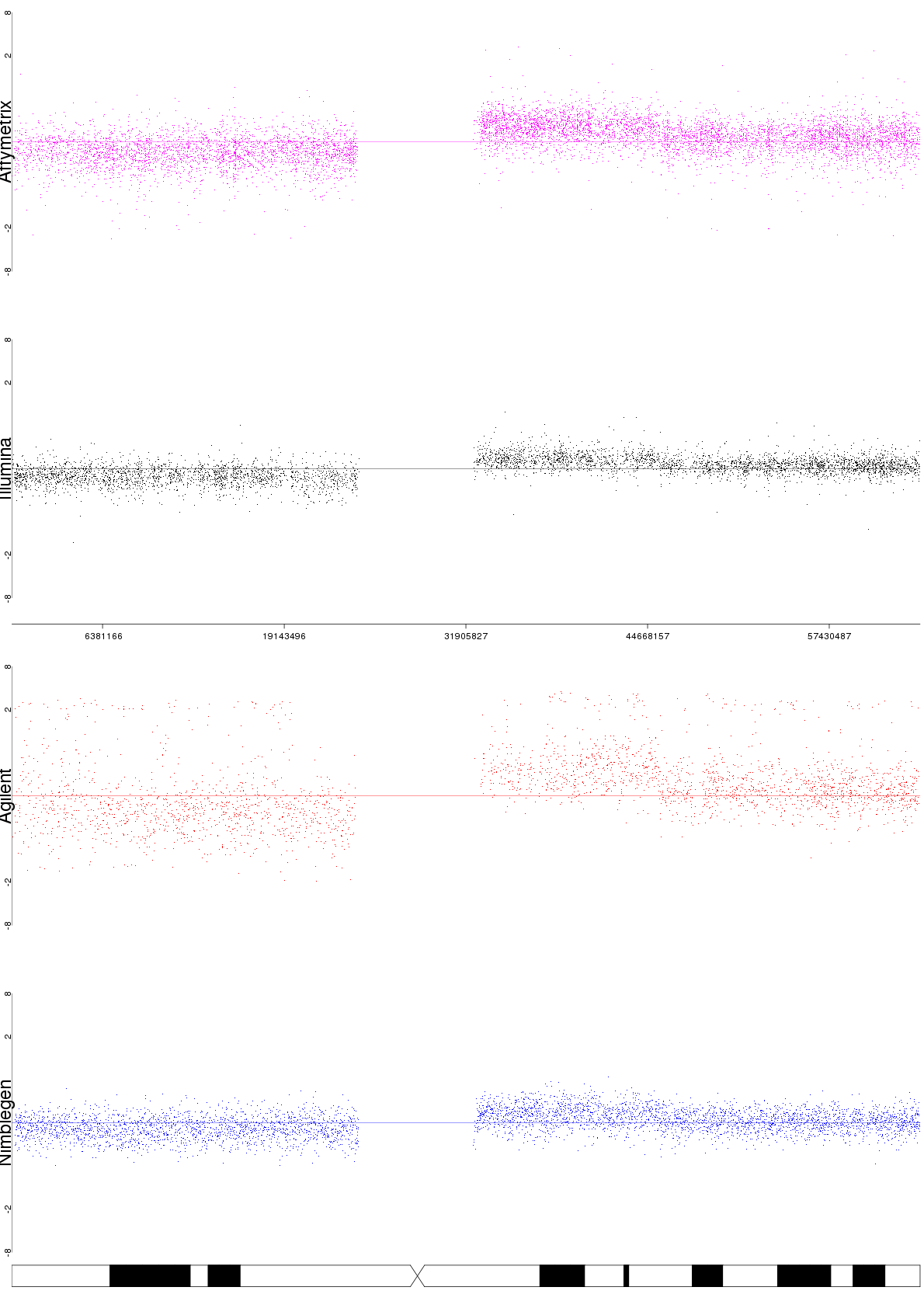

Supplement: Additional file 12 — All sample/chromosome plots for the tumours. Zip folder containing PNGs of all whole-chromosome plots for the tumours. [file 1471-2164-10-588-S12.ZIP › T7195/T7195 chromosome 19.png]

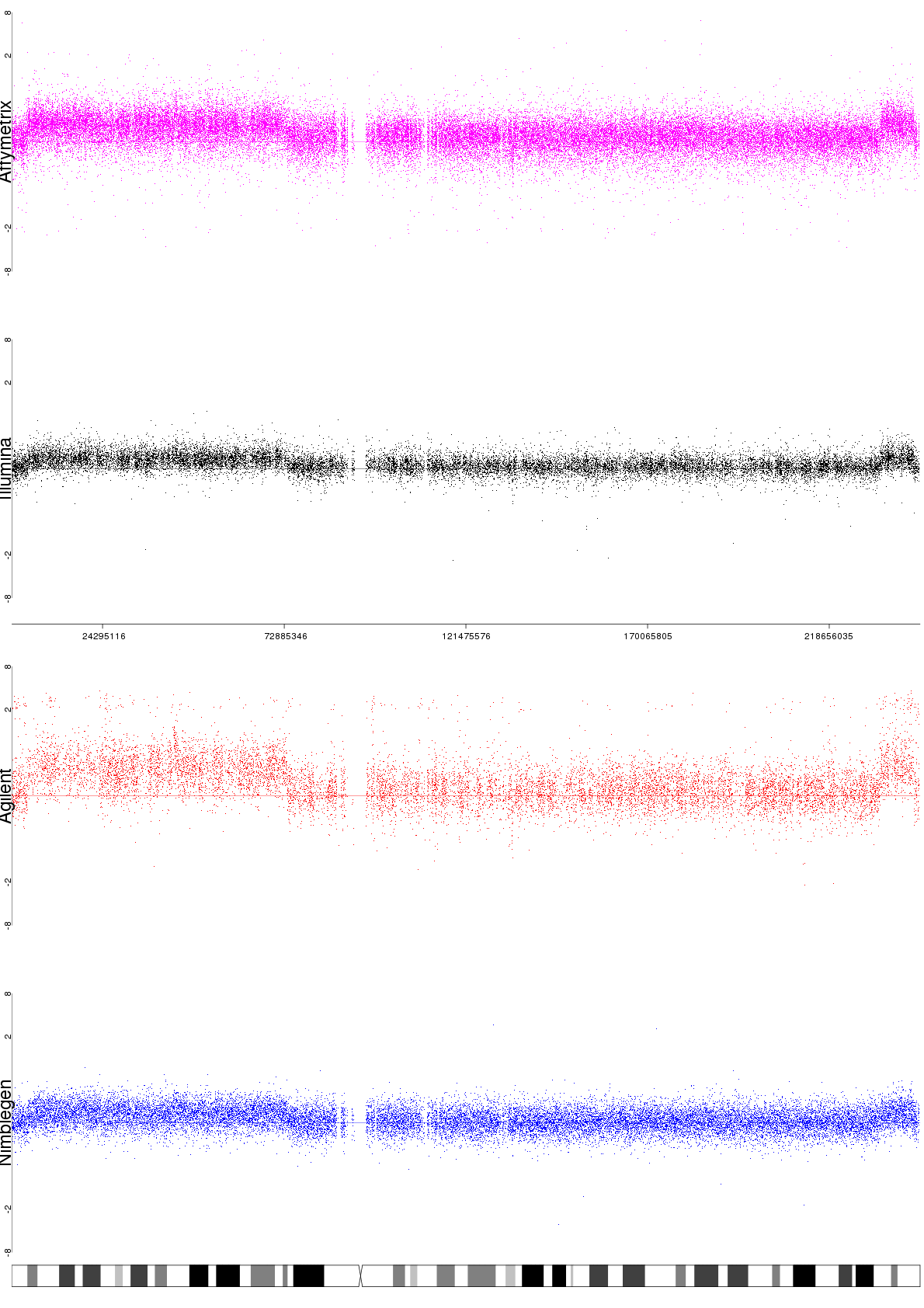

Supplement: Additional file 12 — All sample/chromosome plots for the tumours. Zip folder containing PNGs of all whole-chromosome plots for the tumours. [file 1471-2164-10-588-S12.ZIP › T7195/T7195 chromosome 2.png]

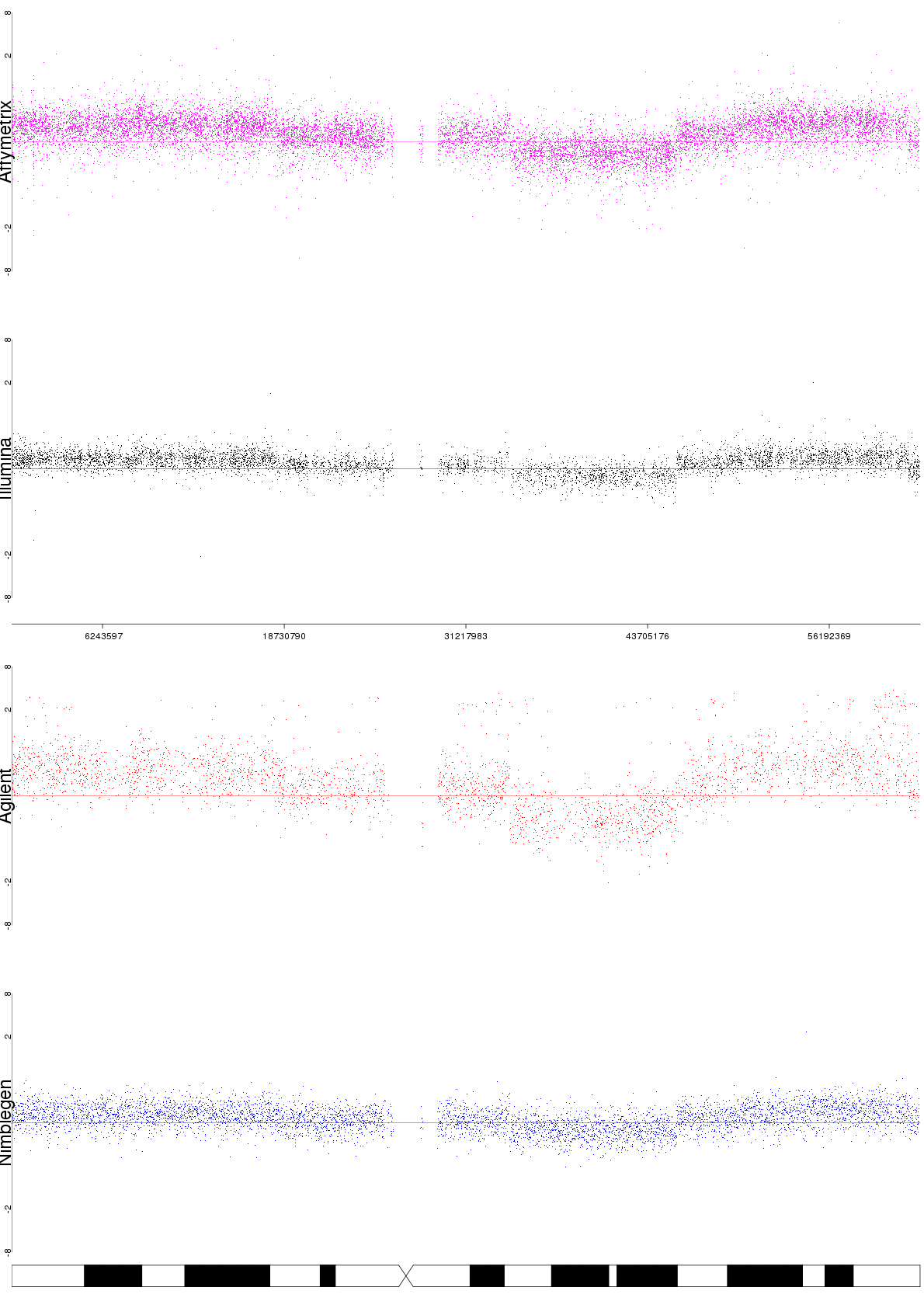

Supplement: Additional file 12 — All sample/chromosome plots for the tumours. Zip folder containing PNGs of all whole-chromosome plots for the tumours. [file 1471-2164-10-588-S12.ZIP › T7195/T7195 chromosome 20.png]

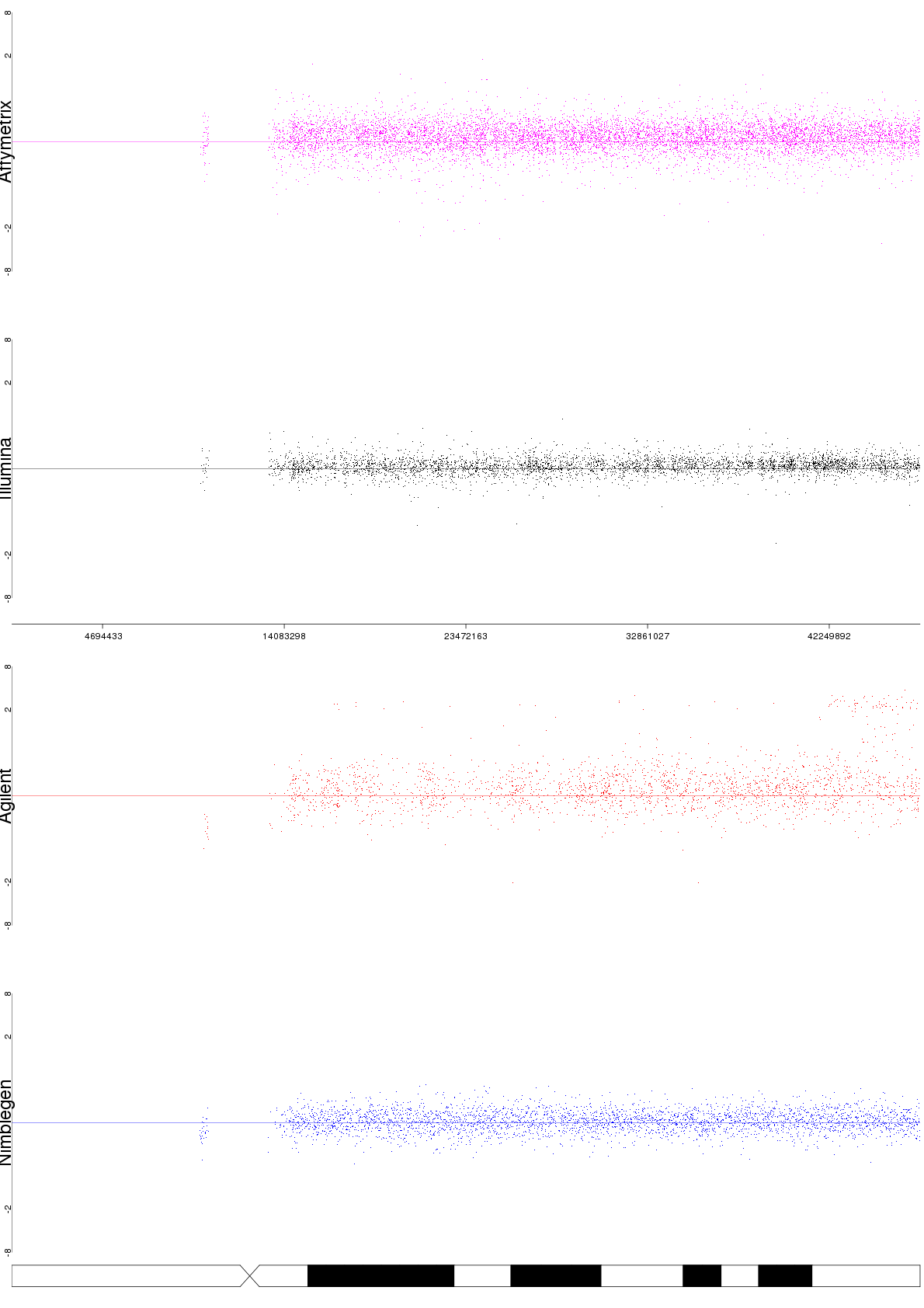

Supplement: Additional file 12 — All sample/chromosome plots for the tumours. Zip folder containing PNGs of all whole-chromosome plots for the tumours. [file 1471-2164-10-588-S12.ZIP › T7195/T7195 chromosome 21.png]

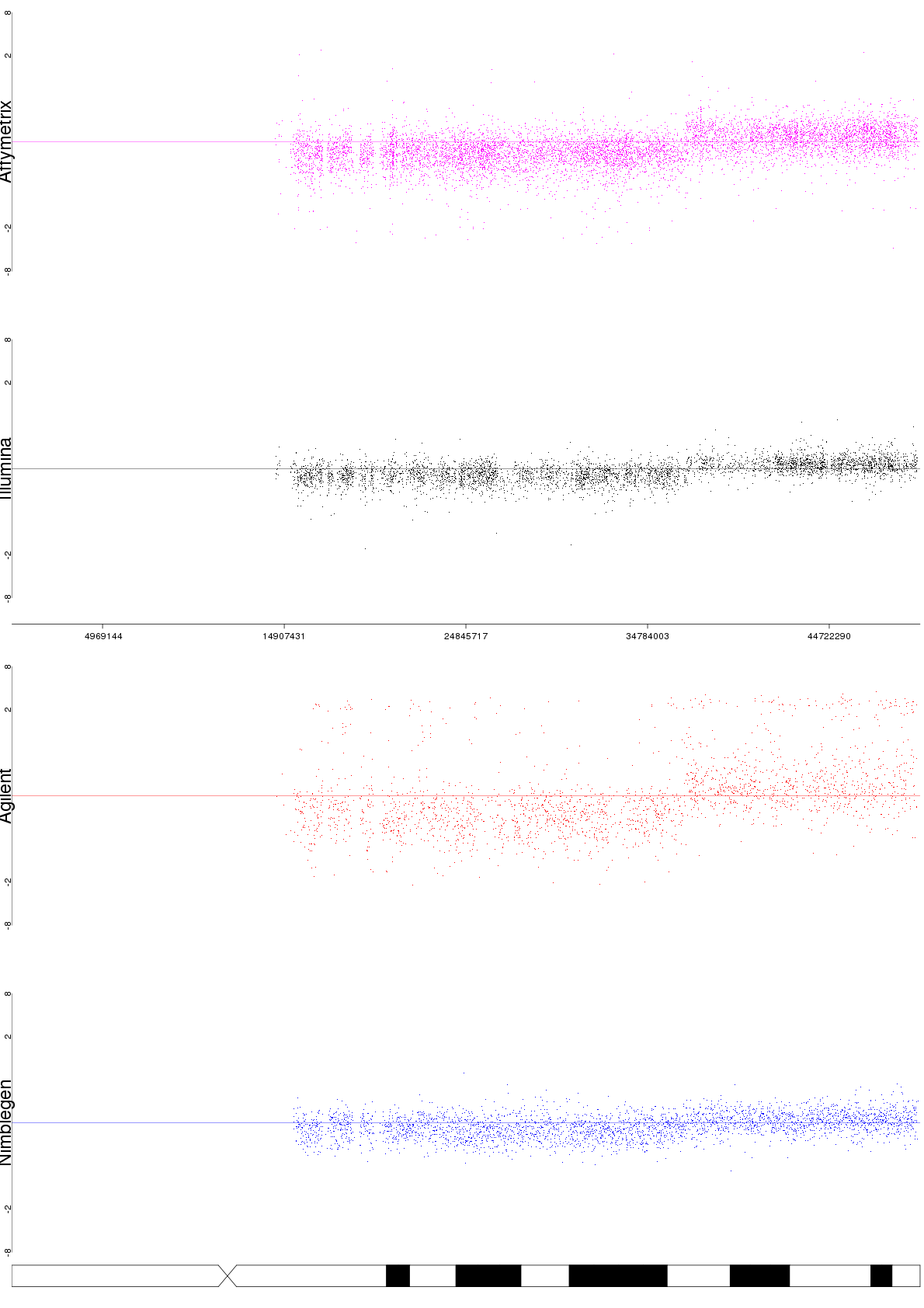

Supplement: Additional file 12 — All sample/chromosome plots for the tumours. Zip folder containing PNGs of all whole-chromosome plots for the tumours. [file 1471-2164-10-588-S12.ZIP › T7195/T7195 chromosome 22.png]

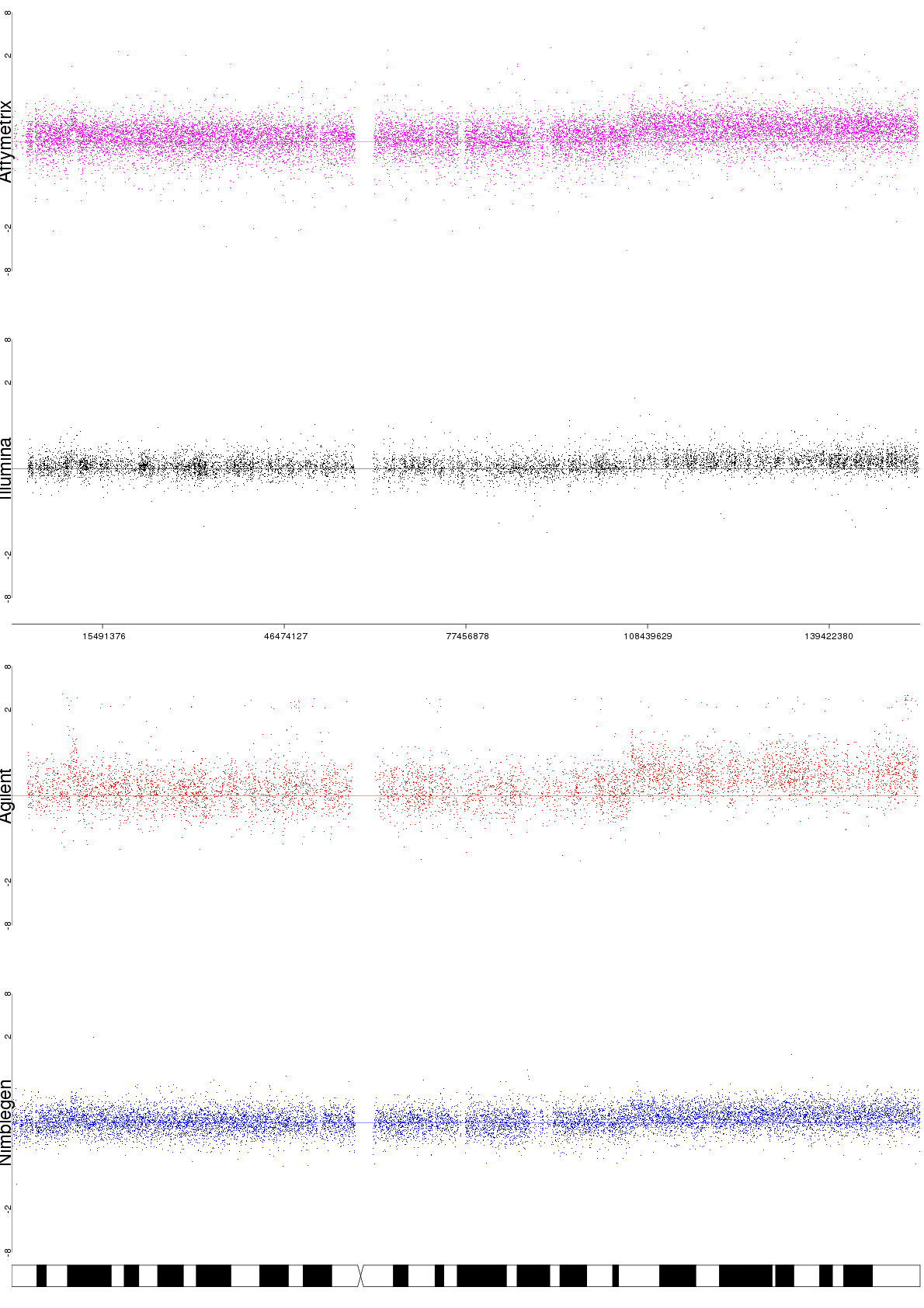

Supplement: Additional file 12 — All sample/chromosome plots for the tumours. Zip folder containing PNGs of all whole-chromosome plots for the tumours. [file 1471-2164-10-588-S12.ZIP › T7195/T7195 chromosome 23.png]

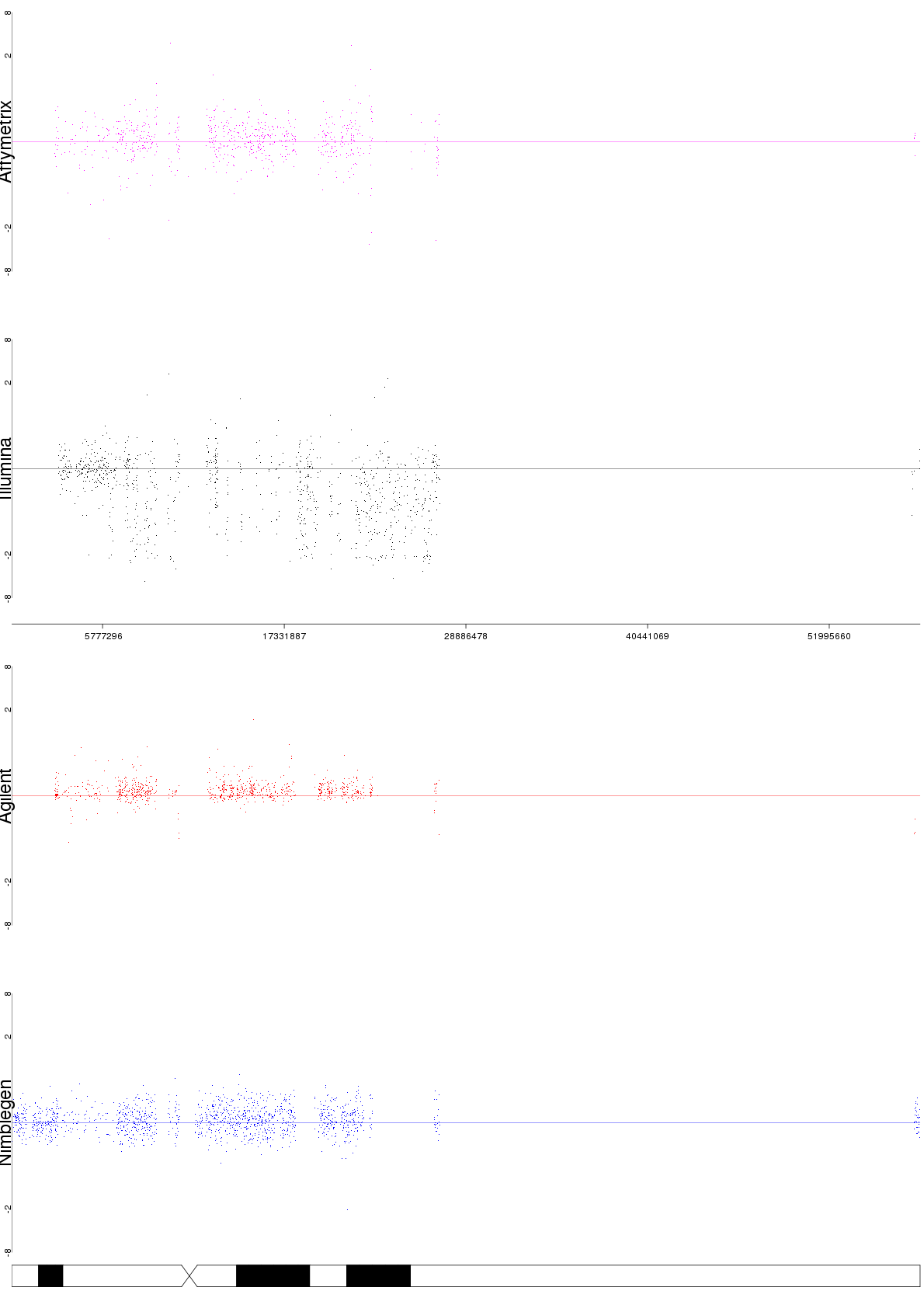

Supplement: Additional file 12 — All sample/chromosome plots for the tumours. Zip folder containing PNGs of all whole-chromosome plots for the tumours. [file 1471-2164-10-588-S12.ZIP › T7195/T7195 chromosome 24.png]

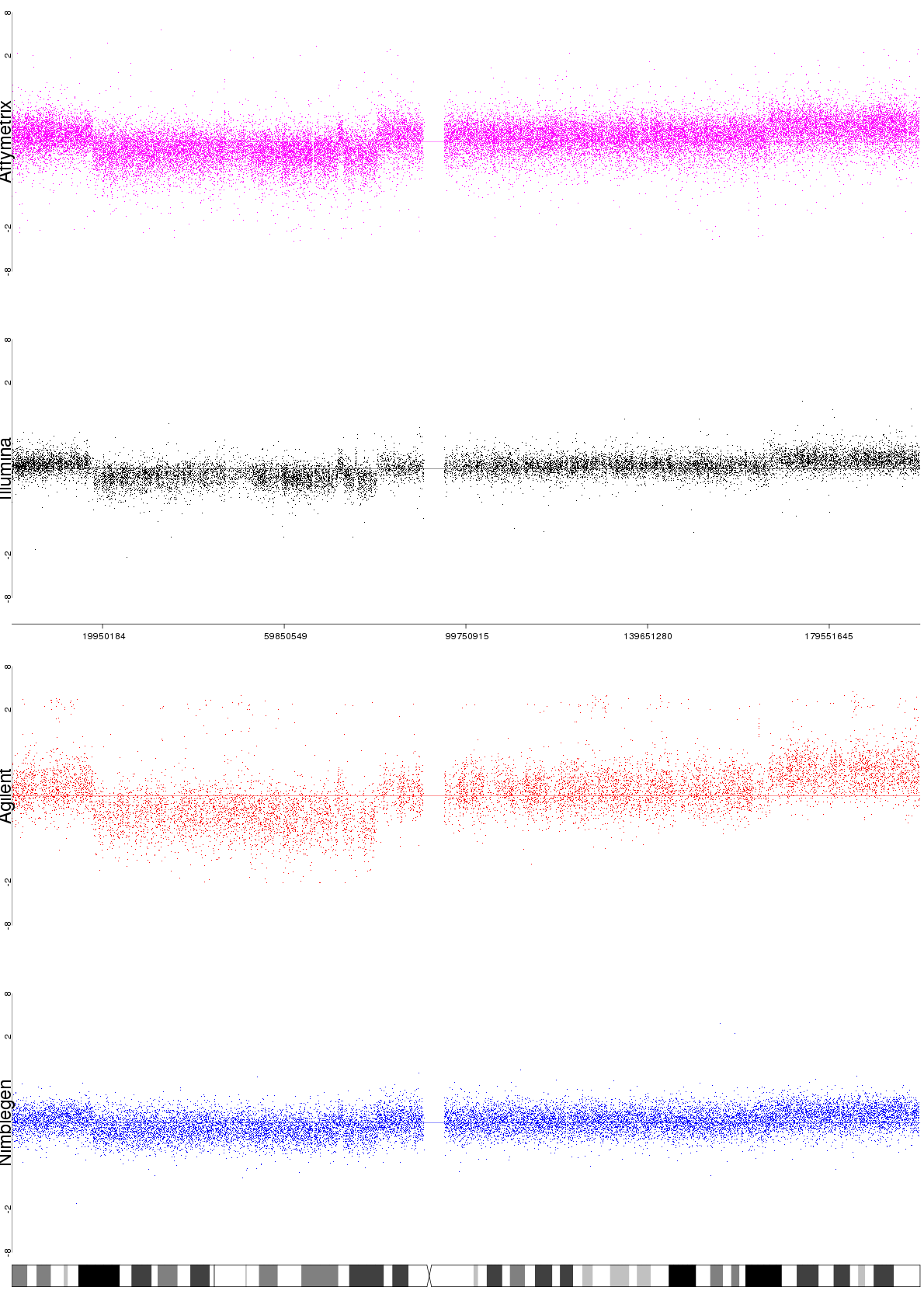

Supplement: Additional file 12 — All sample/chromosome plots for the tumours. Zip folder containing PNGs of all whole-chromosome plots for the tumours. [file 1471-2164-10-588-S12.ZIP › T7195/T7195 chromosome 3.png]

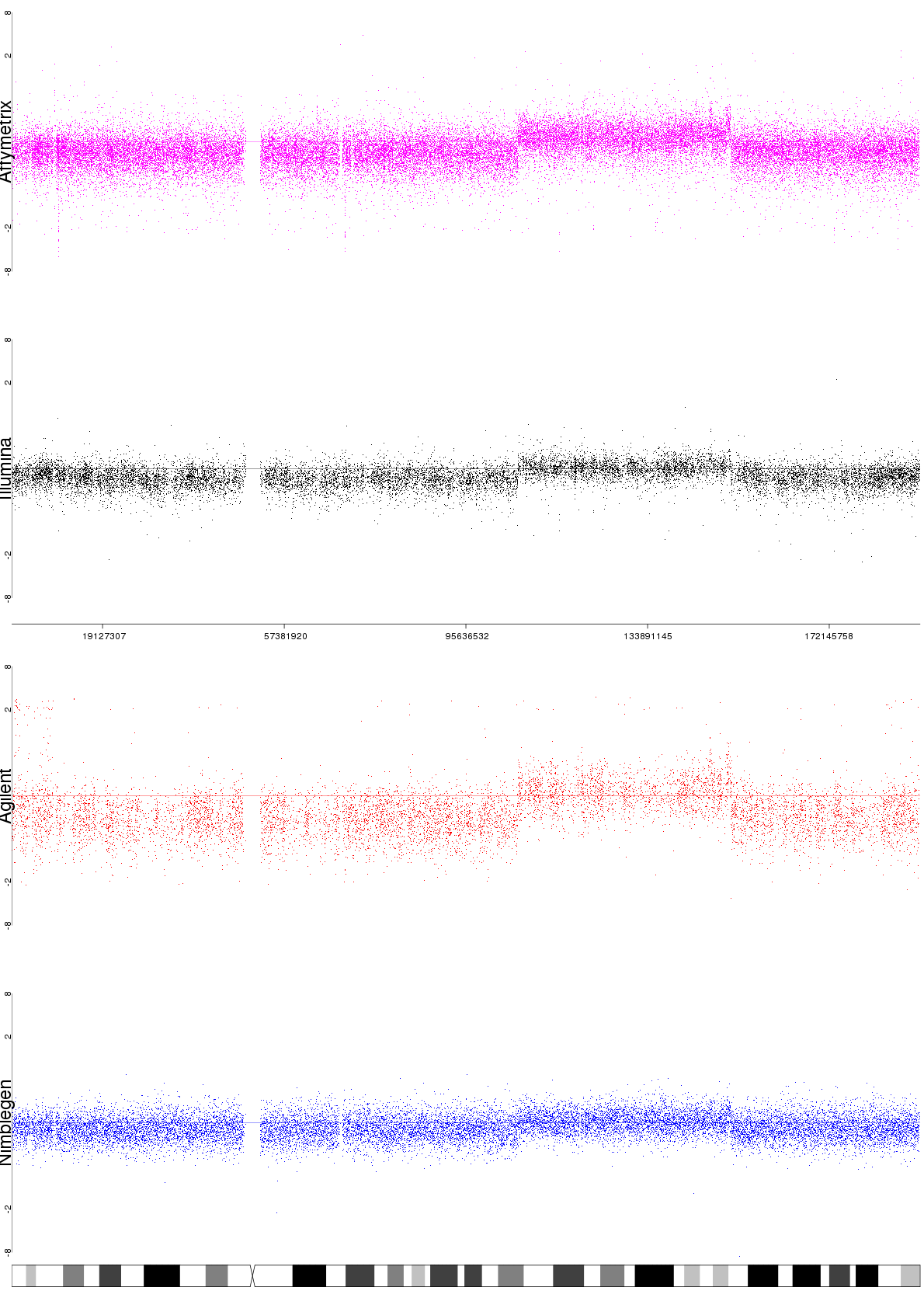

Supplement: Additional file 12 — All sample/chromosome plots for the tumours. Zip folder containing PNGs of all whole-chromosome plots for the tumours. [file 1471-2164-10-588-S12.ZIP › T7195/T7195 chromosome 4.png]

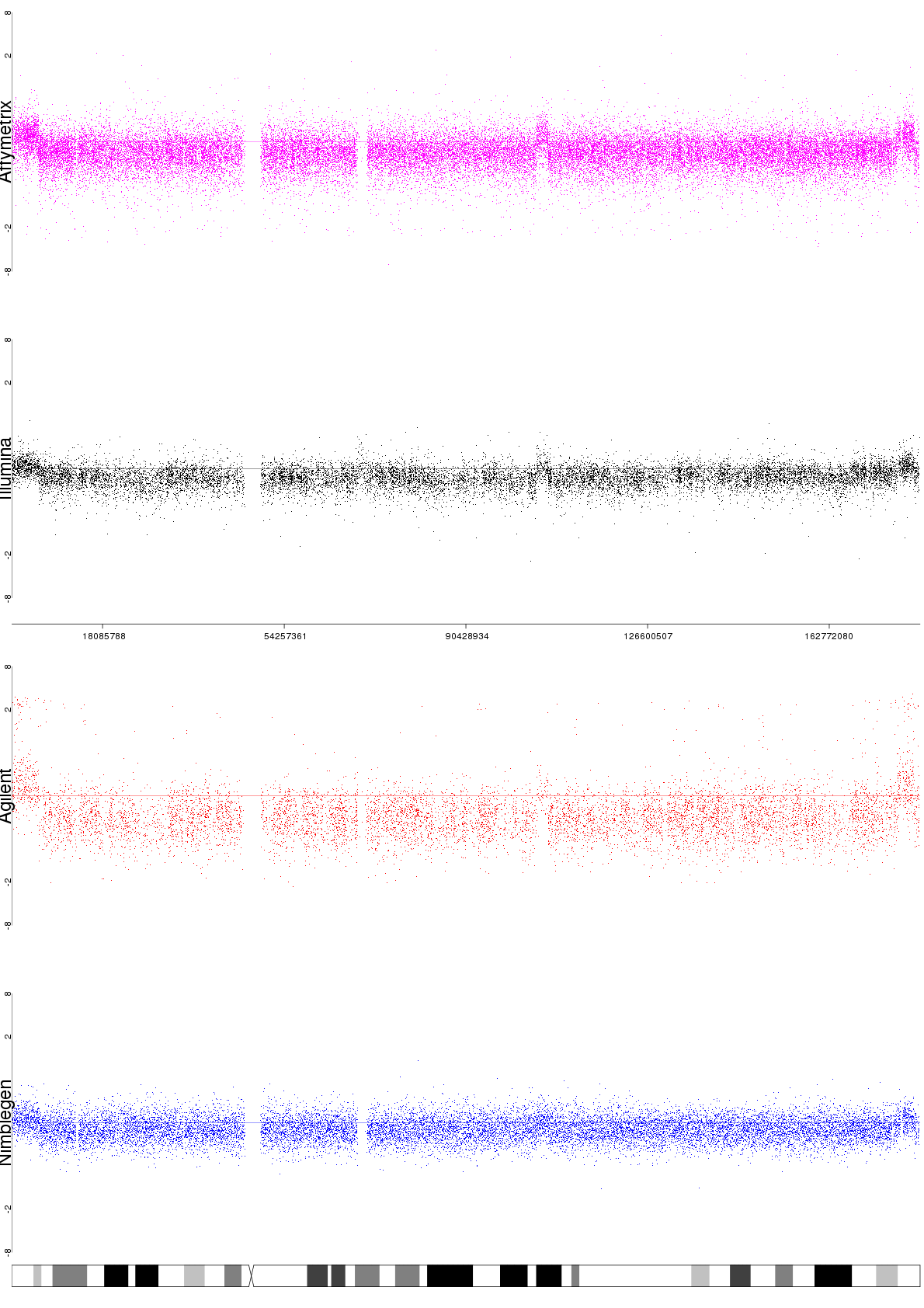

Supplement: Additional file 12 — All sample/chromosome plots for the tumours. Zip folder containing PNGs of all whole-chromosome plots for the tumours. [file 1471-2164-10-588-S12.ZIP › T7195/T7195 chromosome 5.png]

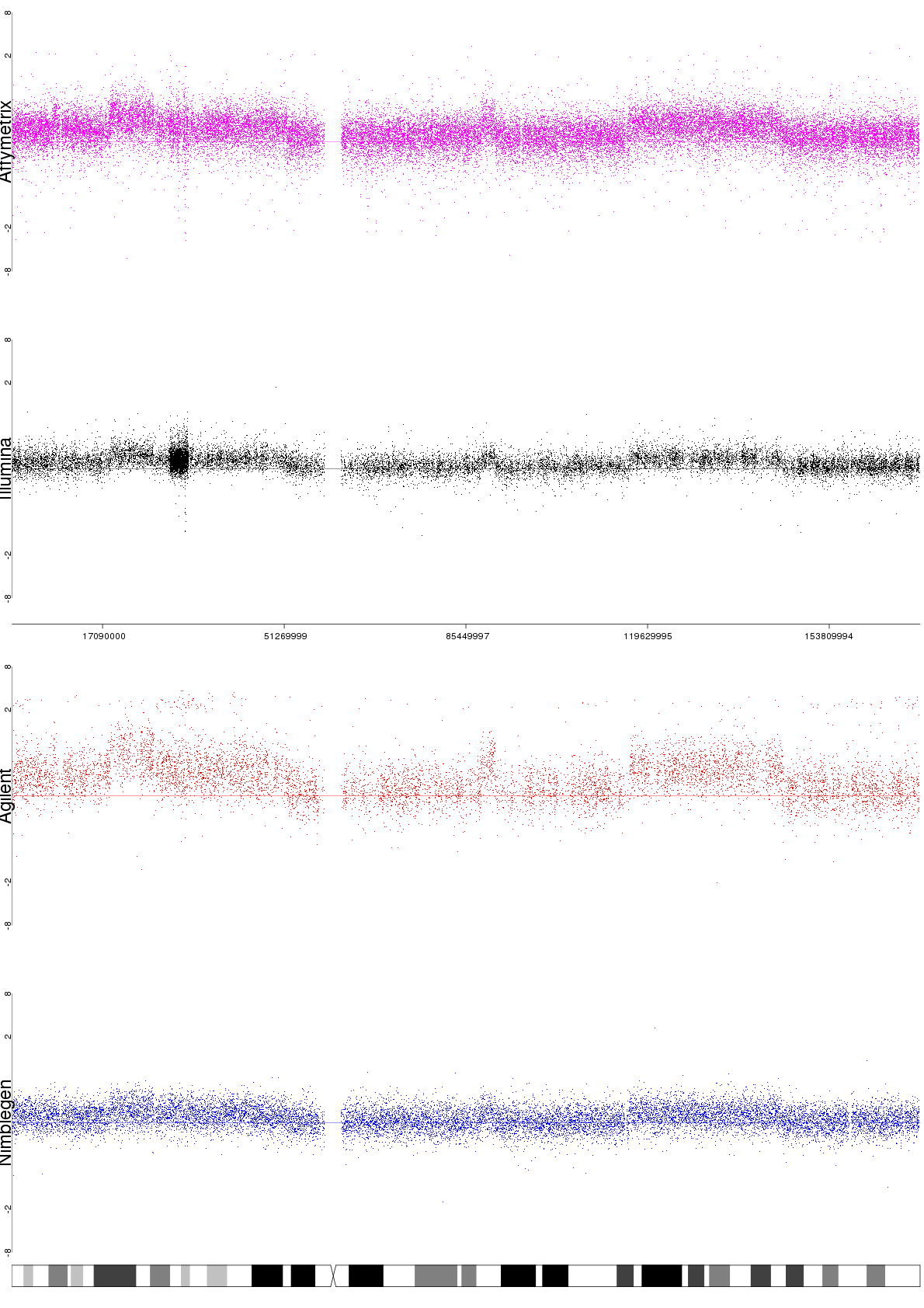

Supplement: Additional file 12 — All sample/chromosome plots for the tumours. Zip folder containing PNGs of all whole-chromosome plots for the tumours. [file 1471-2164-10-588-S12.ZIP › T7195/T7195 chromosome 6.png]

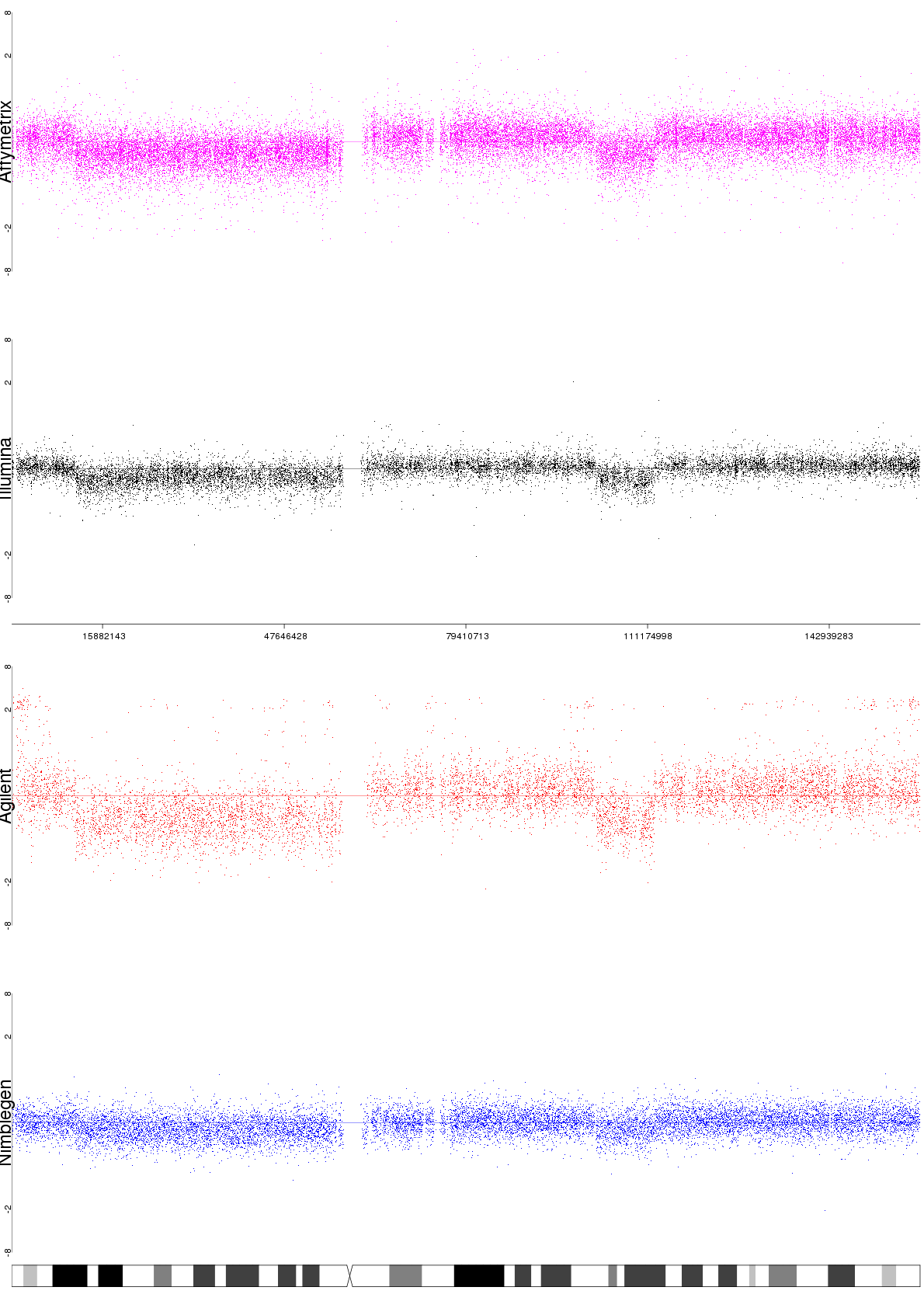

Supplement: Additional file 12 — All sample/chromosome plots for the tumours. Zip folder containing PNGs of all whole-chromosome plots for the tumours. [file 1471-2164-10-588-S12.ZIP › T7195/T7195 chromosome 7.png]

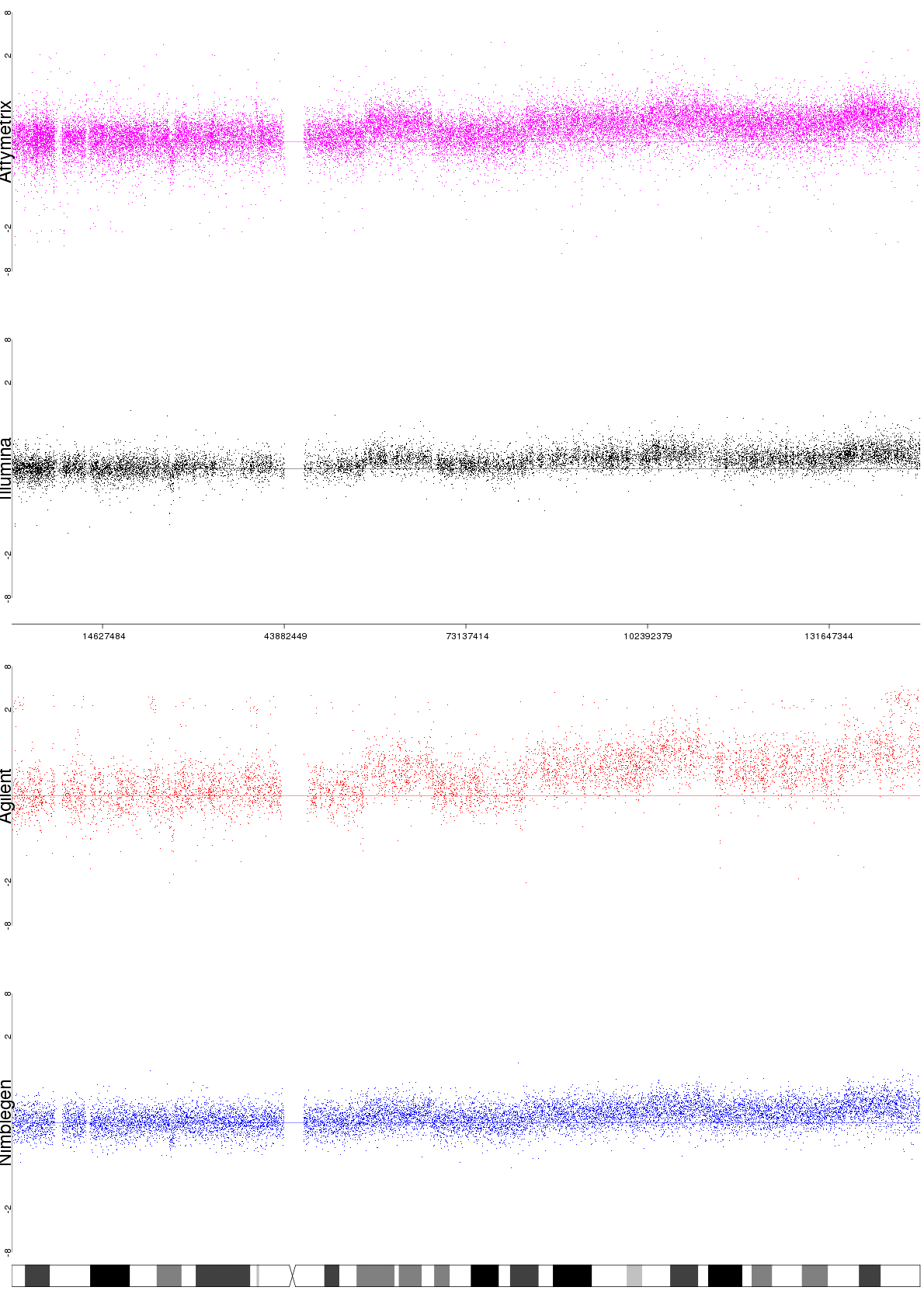

Supplement: Additional file 12 — All sample/chromosome plots for the tumours. Zip folder containing PNGs of all whole-chromosome plots for the tumours. [file 1471-2164-10-588-S12.ZIP › T7195/T7195 chromosome 8.png]

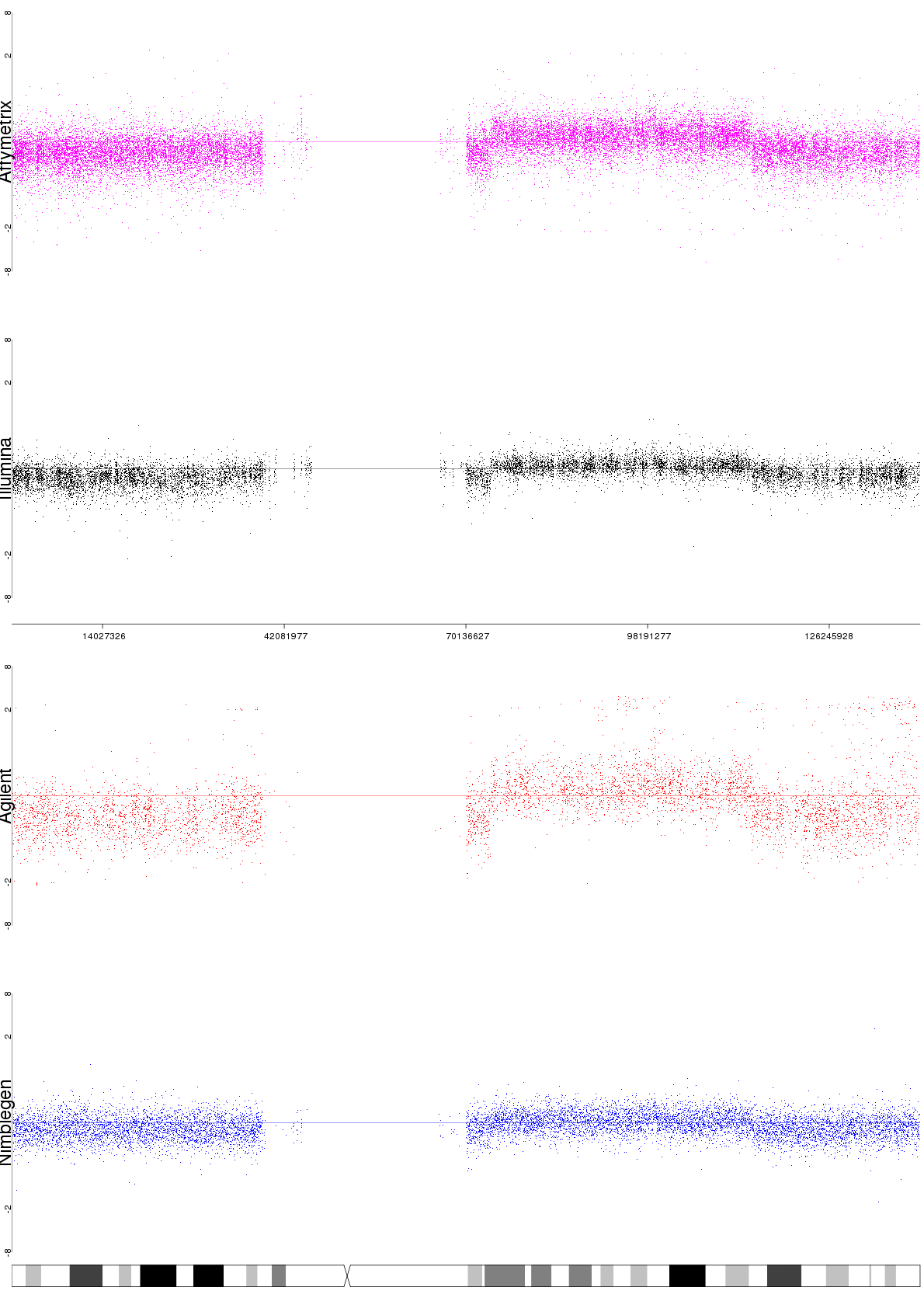

Supplement: Additional file 12 — All sample/chromosome plots for the tumours. Zip folder containing PNGs of all whole-chromosome plots for the tumours. [file 1471-2164-10-588-S12.ZIP › T7195/T7195 chromosome 9.png]

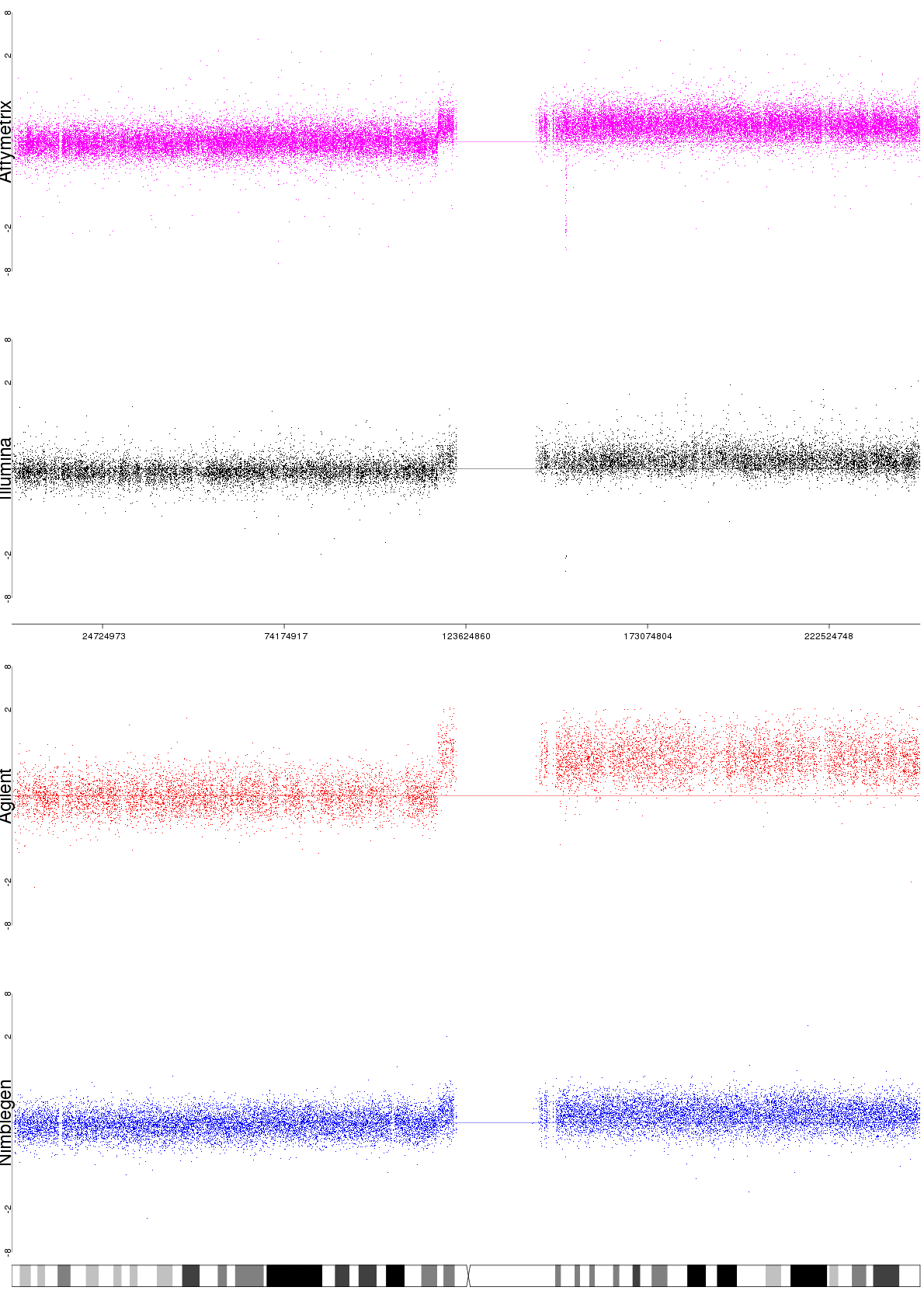

Supplement: Additional file 12 — All sample/chromosome plots for the tumours. Zip folder containing PNGs of all whole-chromosome plots for the tumours. [file 1471-2164-10-588-S12.ZIP › T7201/T7201 chromosome 1.png]

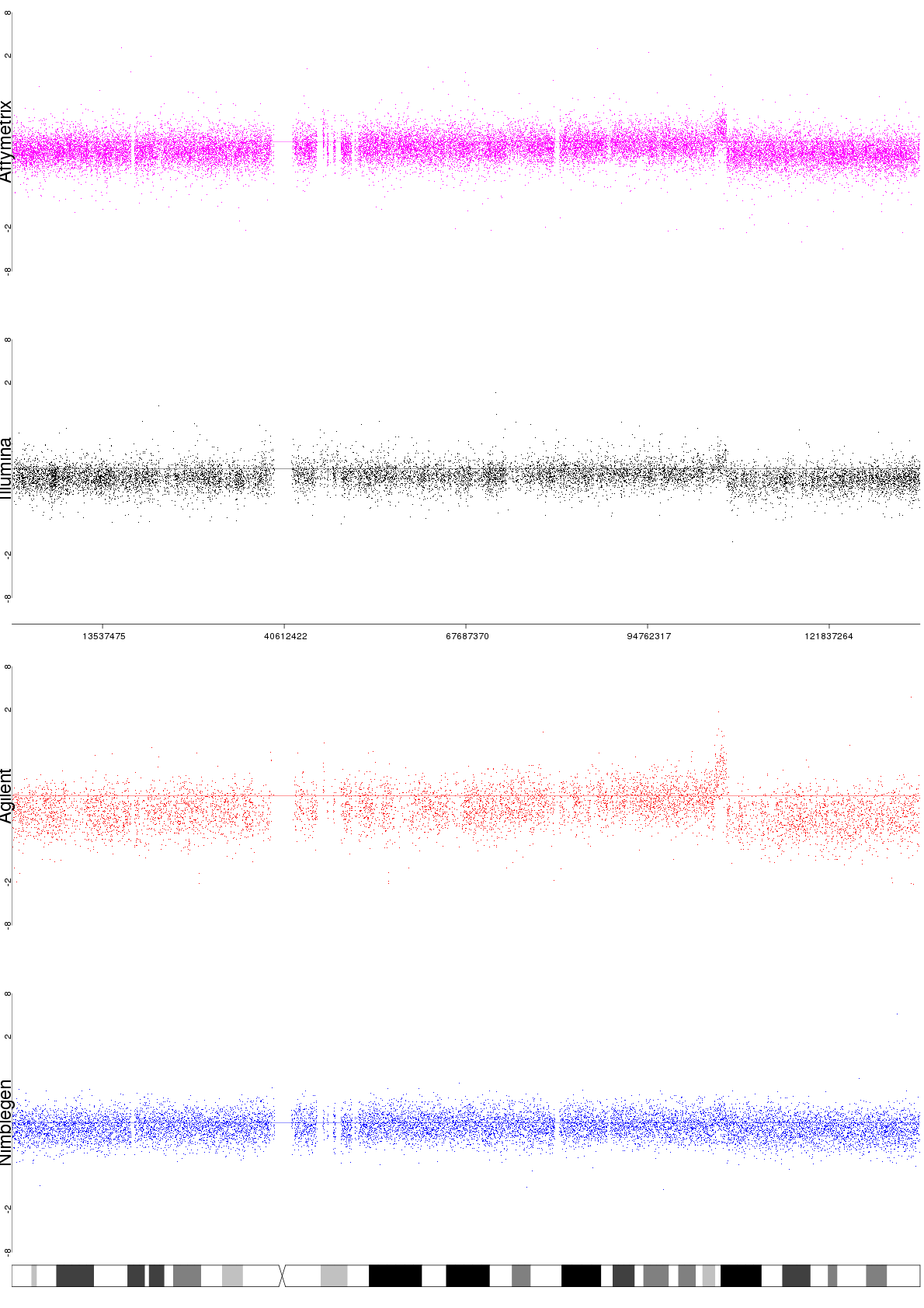

Supplement: Additional file 12 — All sample/chromosome plots for the tumours. Zip folder containing PNGs of all whole-chromosome plots for the tumours. [file 1471-2164-10-588-S12.ZIP › T7201/T7201 chromosome 10.png]

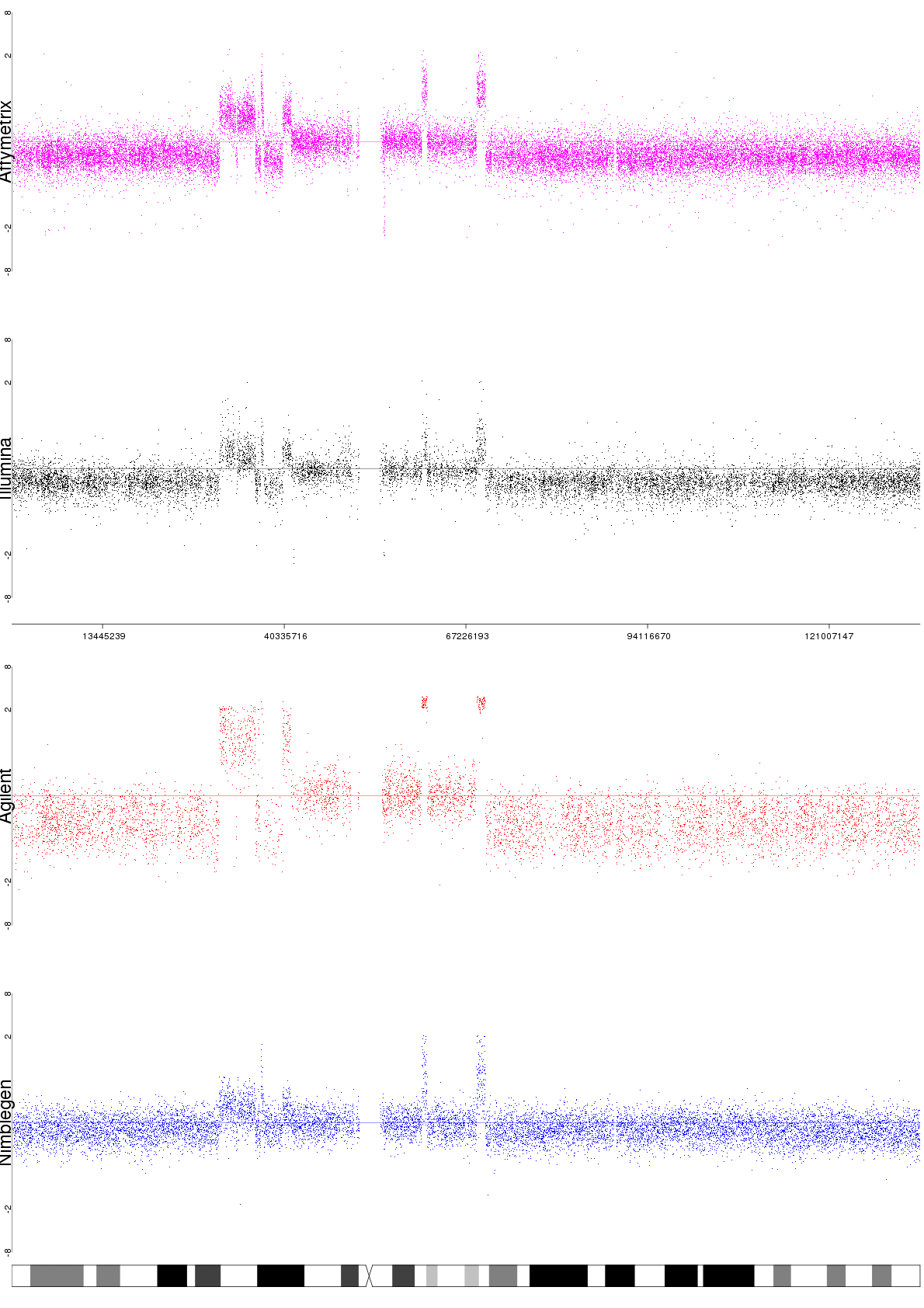

Supplement: Additional file 12 — All sample/chromosome plots for the tumours. Zip folder containing PNGs of all whole-chromosome plots for the tumours. [file 1471-2164-10-588-S12.ZIP › T7201/T7201 chromosome 11.png]

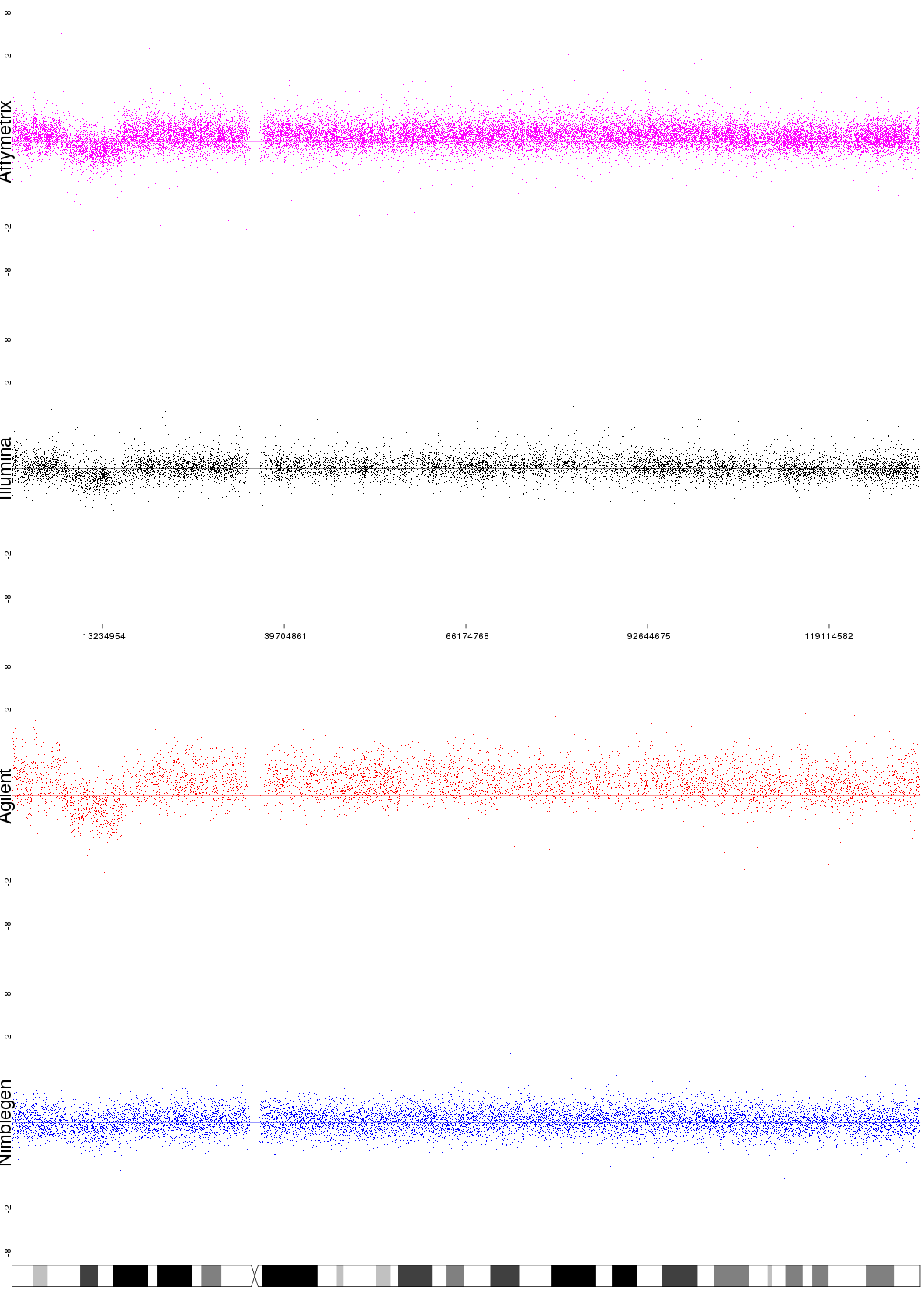

Supplement: Additional file 12 — All sample/chromosome plots for the tumours. Zip folder containing PNGs of all whole-chromosome plots for the tumours. [file 1471-2164-10-588-S12.ZIP › T7201/T7201 chromosome 12.png]

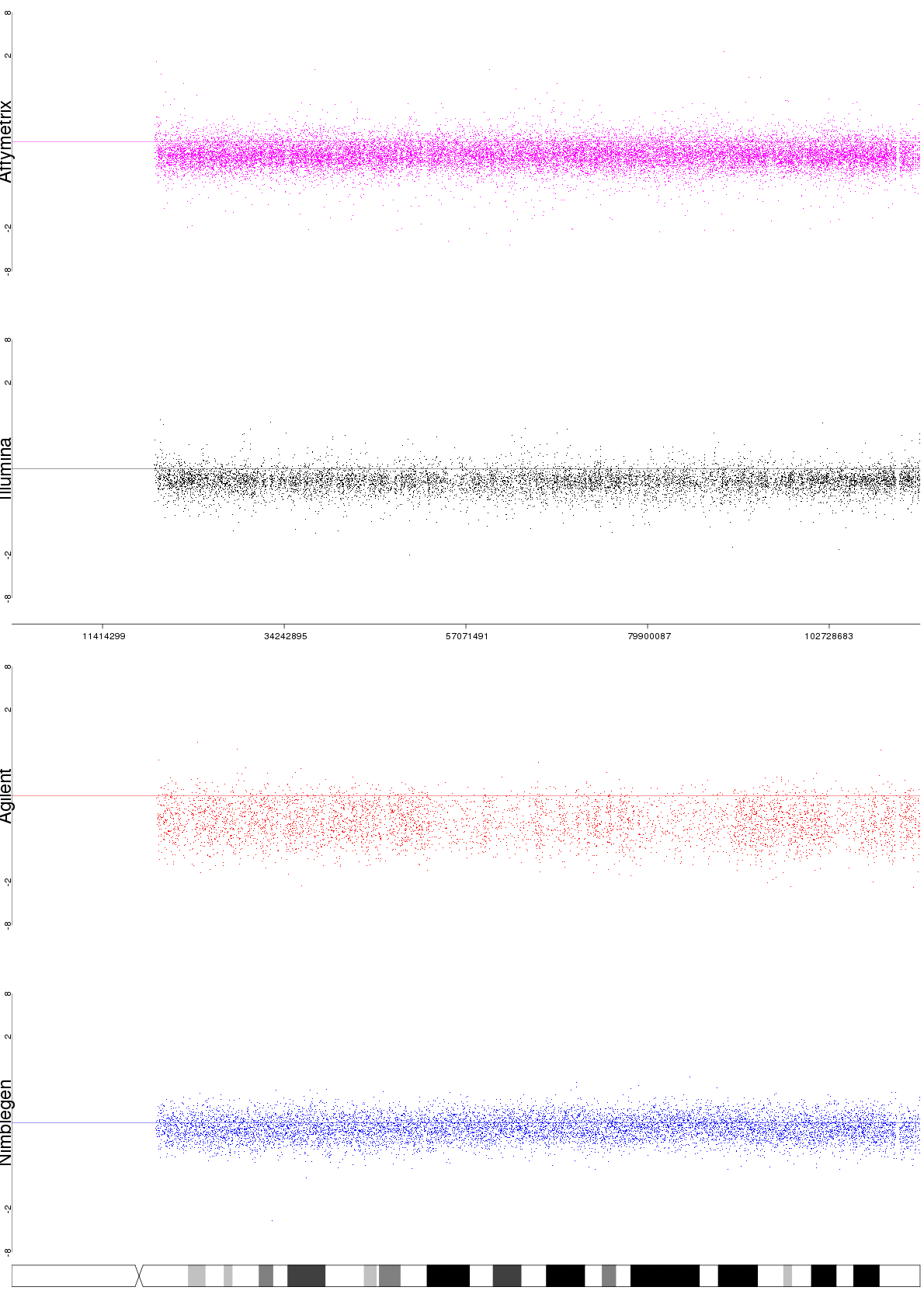

Supplement: Additional file 12 — All sample/chromosome plots for the tumours. Zip folder containing PNGs of all whole-chromosome plots for the tumours. [file 1471-2164-10-588-S12.ZIP › T7201/T7201 chromosome 13.png]

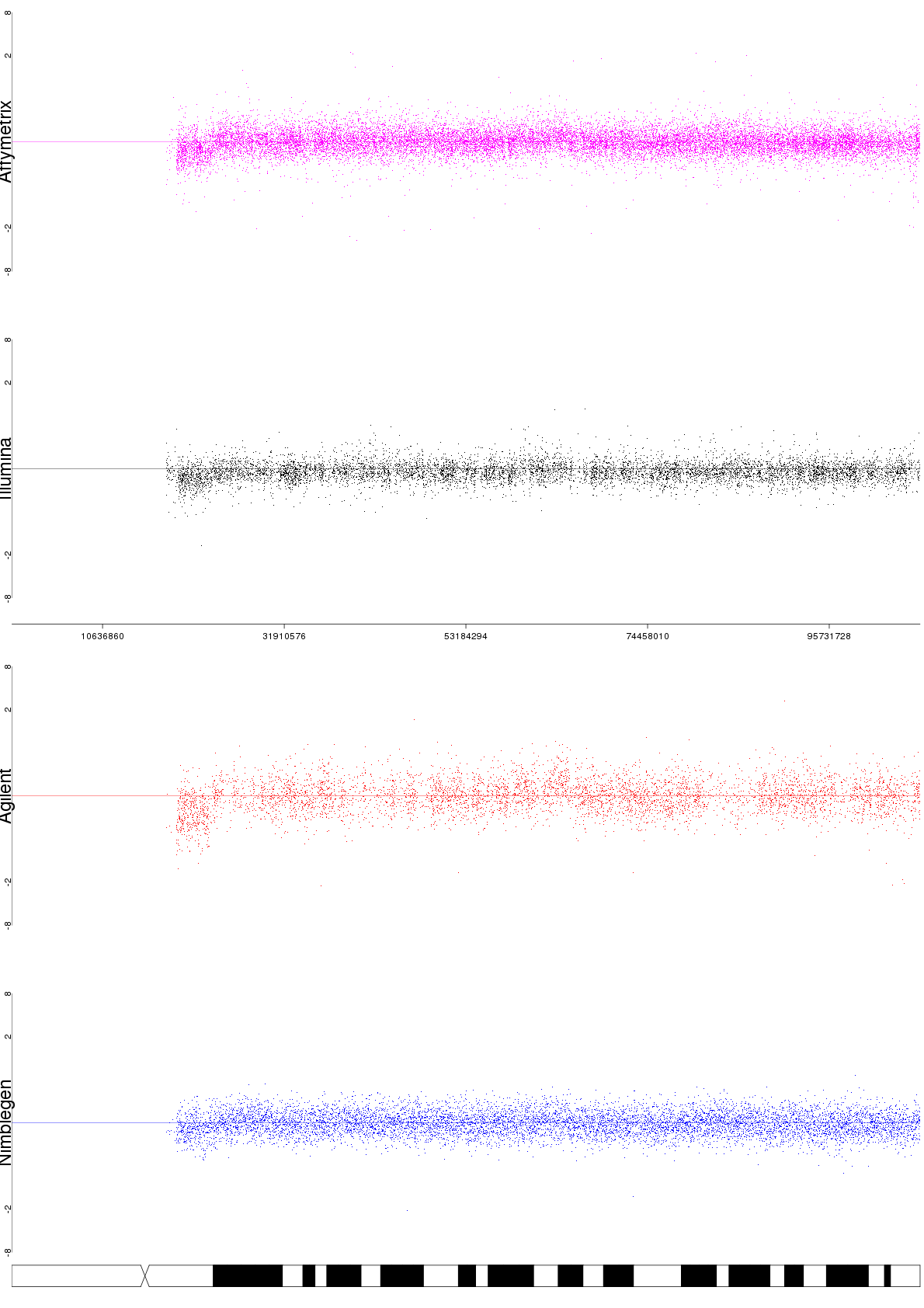

Supplement: Additional file 12 — All sample/chromosome plots for the tumours. Zip folder containing PNGs of all whole-chromosome plots for the tumours. [file 1471-2164-10-588-S12.ZIP › T7201/T7201 chromosome 14.png]

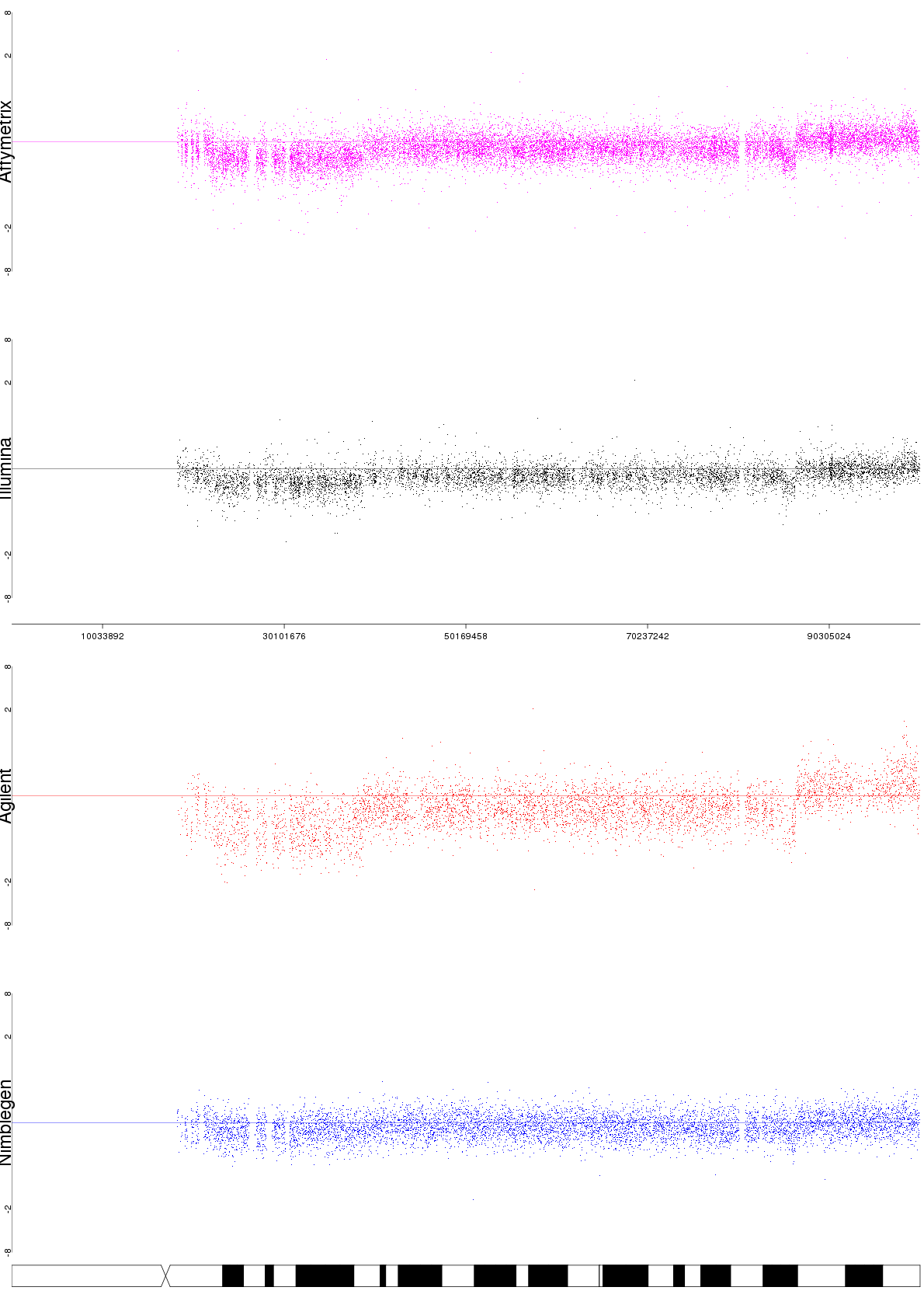

Supplement: Additional file 12 — All sample/chromosome plots for the tumours. Zip folder containing PNGs of all whole-chromosome plots for the tumours. [file 1471-2164-10-588-S12.ZIP › T7201/T7201 chromosome 15.png]

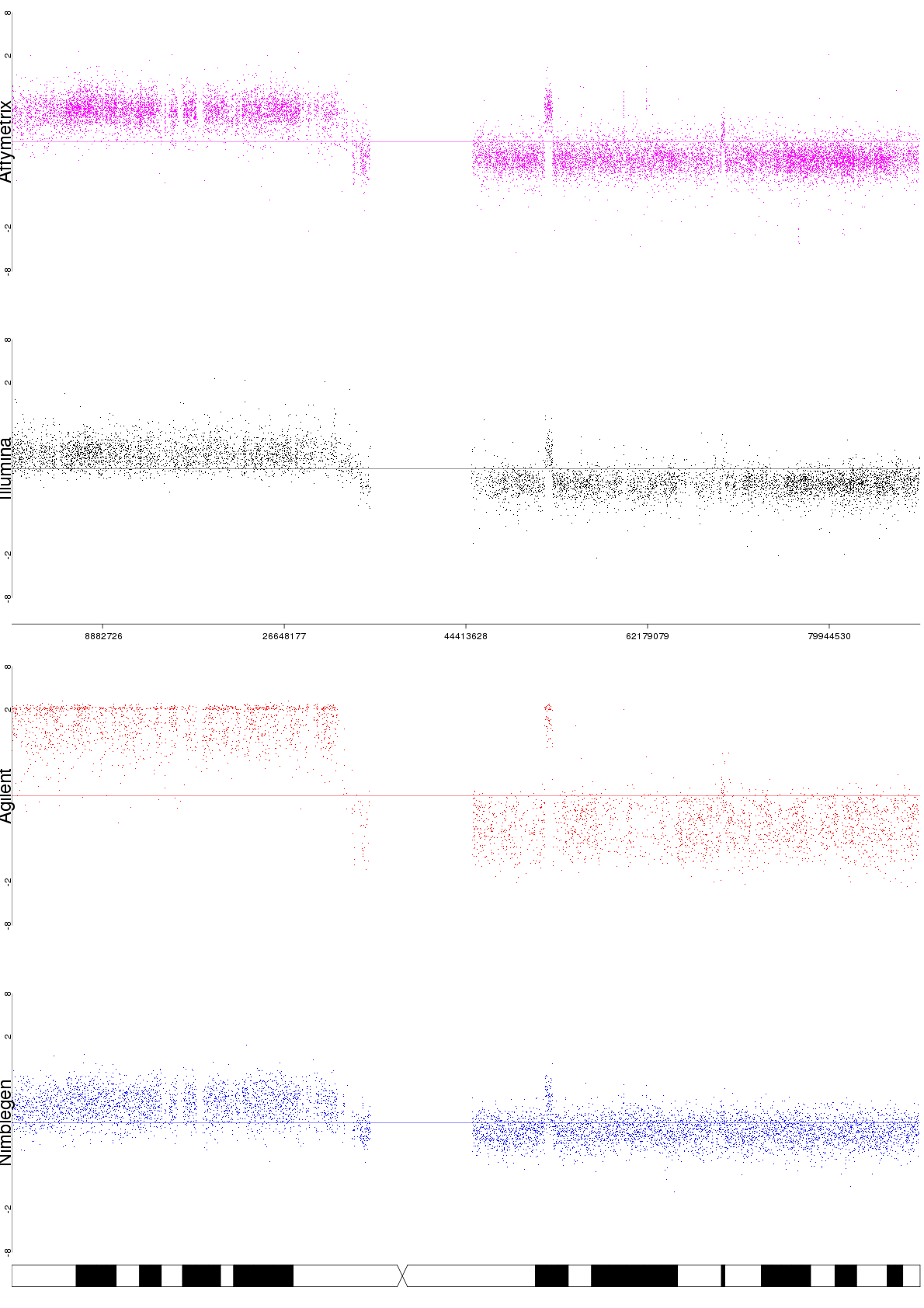

Supplement: Additional file 12 — All sample/chromosome plots for the tumours. Zip folder containing PNGs of all whole-chromosome plots for the tumours. [file 1471-2164-10-588-S12.ZIP › T7201/T7201 chromosome 16.png]

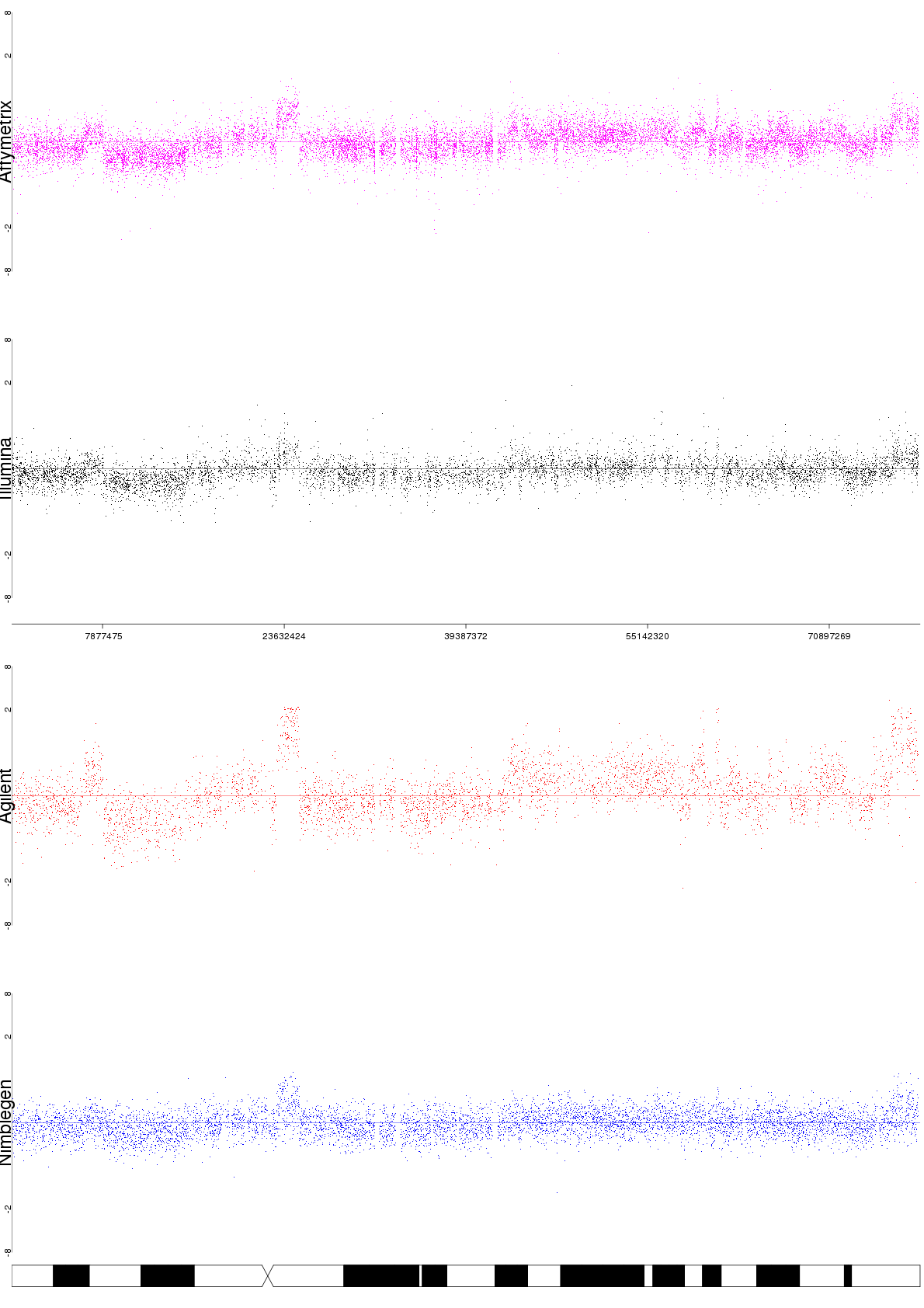

Supplement: Additional file 12 — All sample/chromosome plots for the tumours. Zip folder containing PNGs of all whole-chromosome plots for the tumours. [file 1471-2164-10-588-S12.ZIP › T7201/T7201 chromosome 17.png]

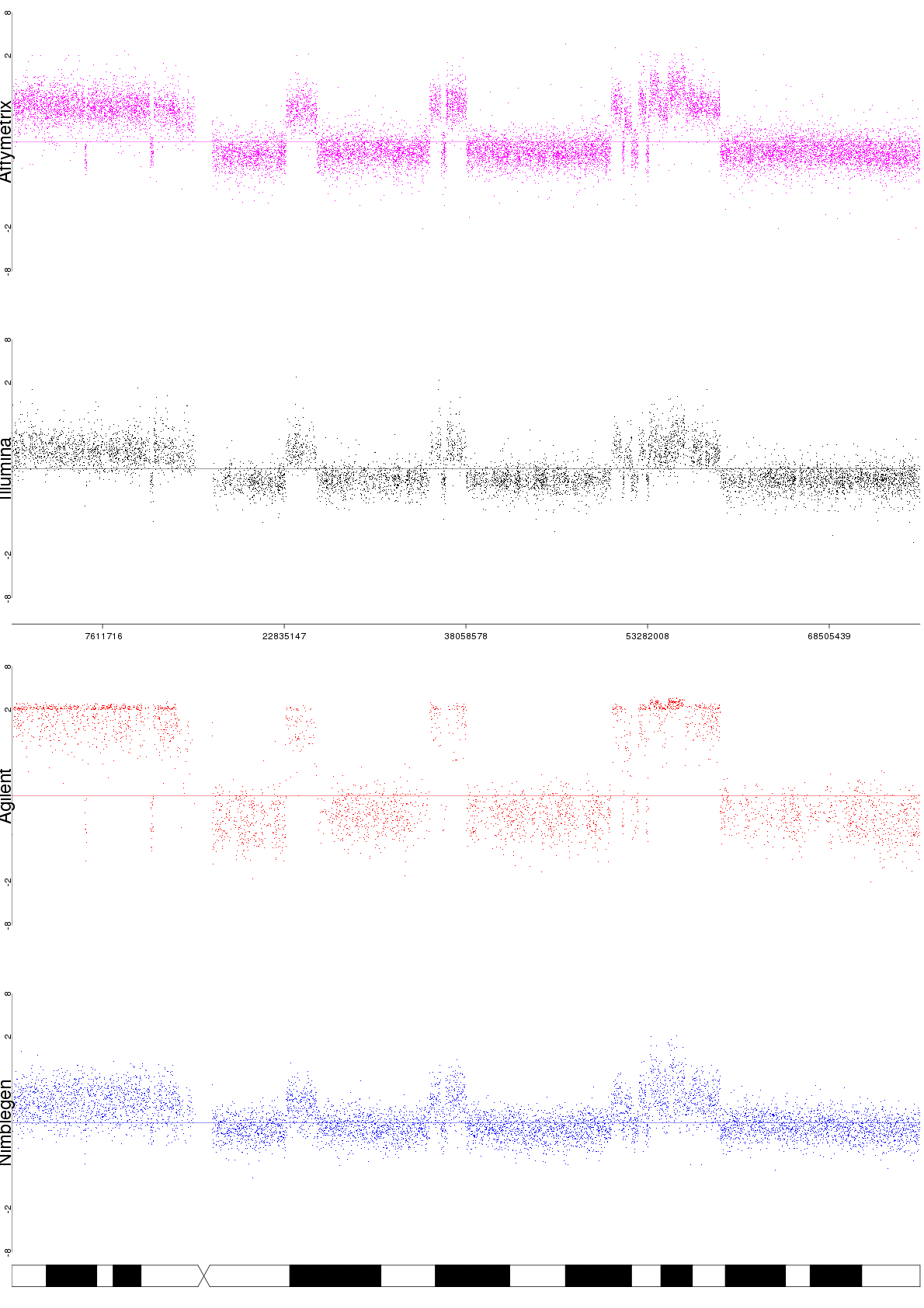

Supplement: Additional file 12 — All sample/chromosome plots for the tumours. Zip folder containing PNGs of all whole-chromosome plots for the tumours. [file 1471-2164-10-588-S12.ZIP › T7201/T7201 chromosome 18.png]

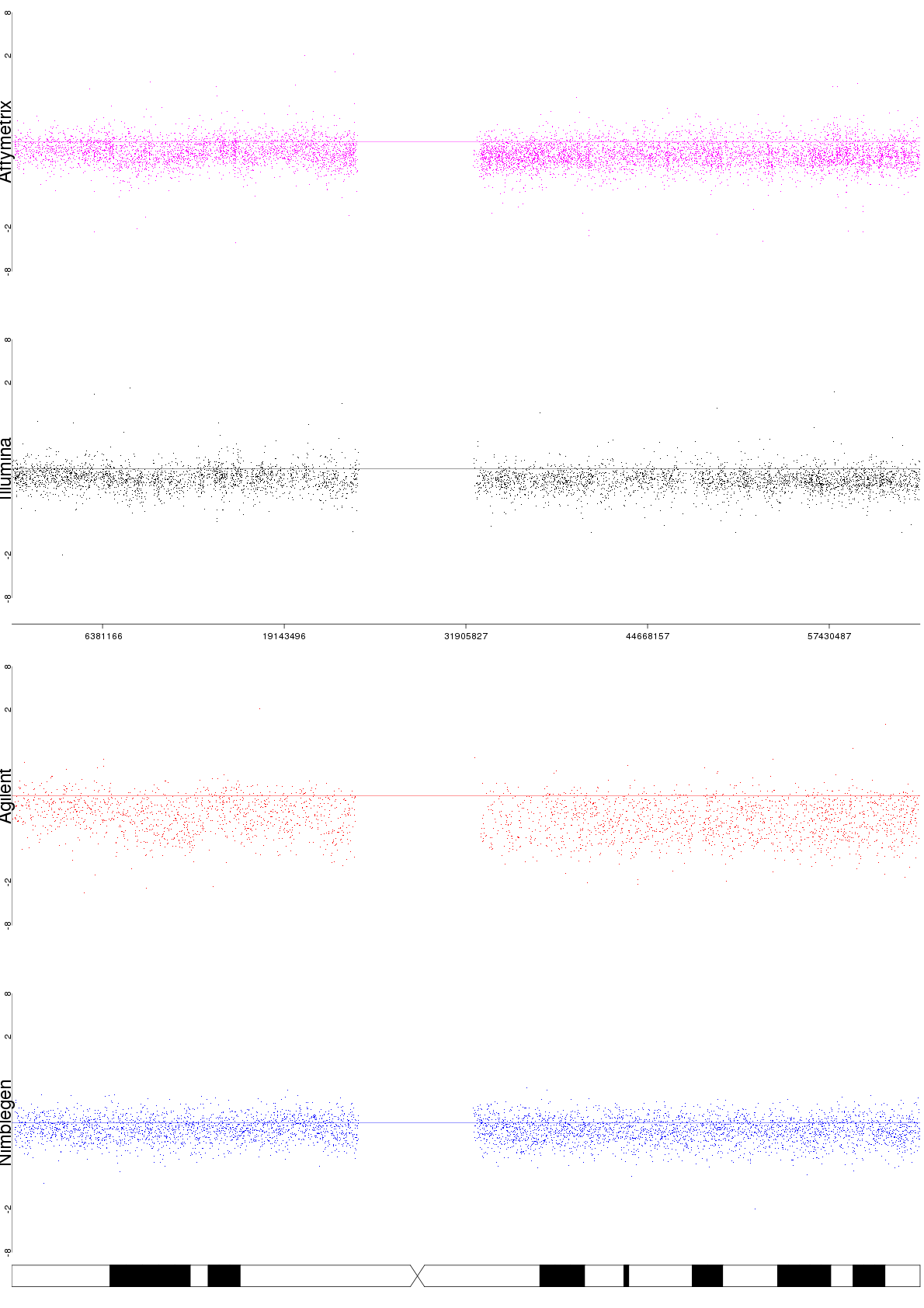

Supplement: Additional file 12 — All sample/chromosome plots for the tumours. Zip folder containing PNGs of all whole-chromosome plots for the tumours. [file 1471-2164-10-588-S12.ZIP › T7201/T7201 chromosome 19.png]

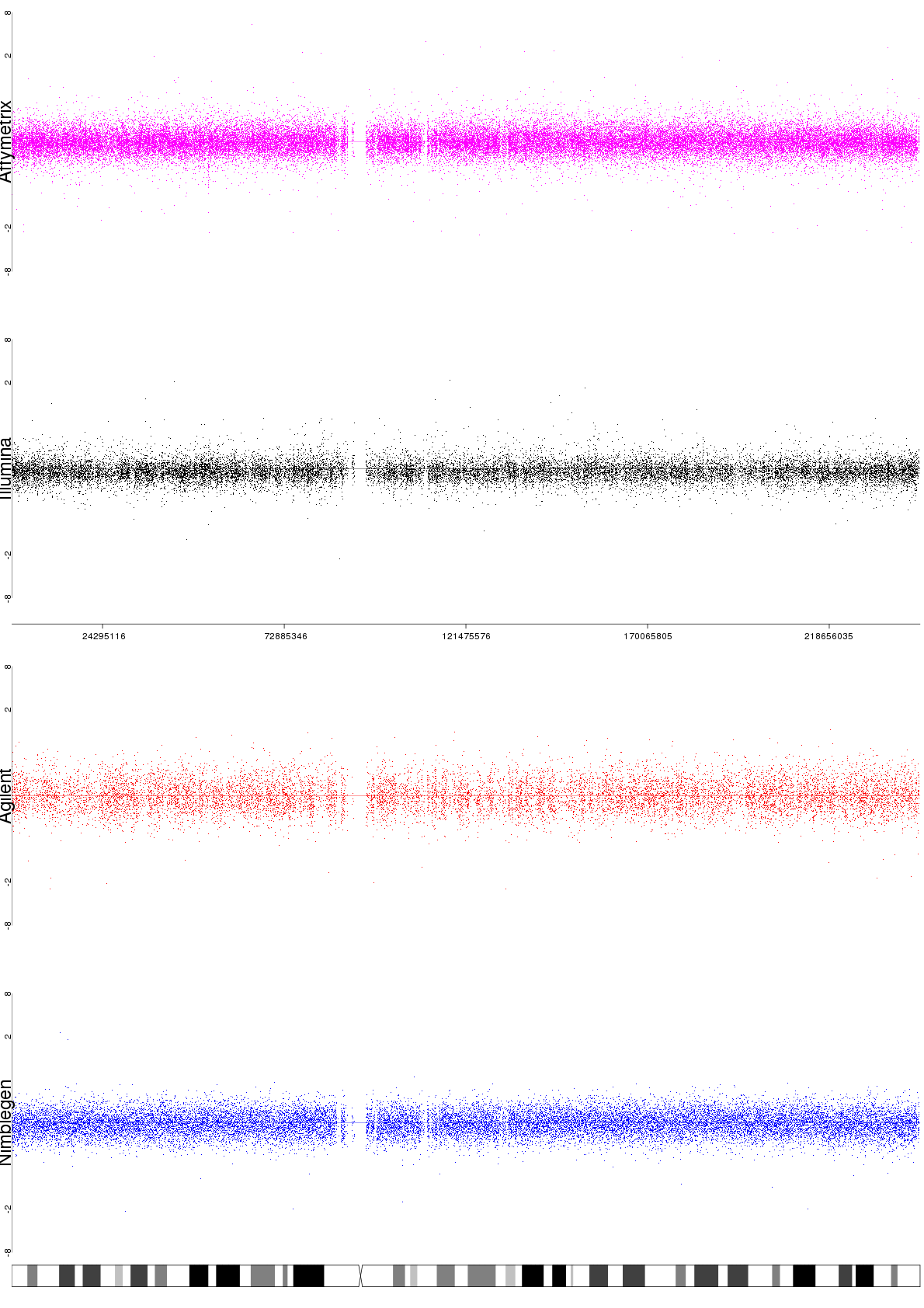

Supplement: Additional file 12 — All sample/chromosome plots for the tumours. Zip folder containing PNGs of all whole-chromosome plots for the tumours. [file 1471-2164-10-588-S12.ZIP › T7201/T7201 chromosome 2.png]

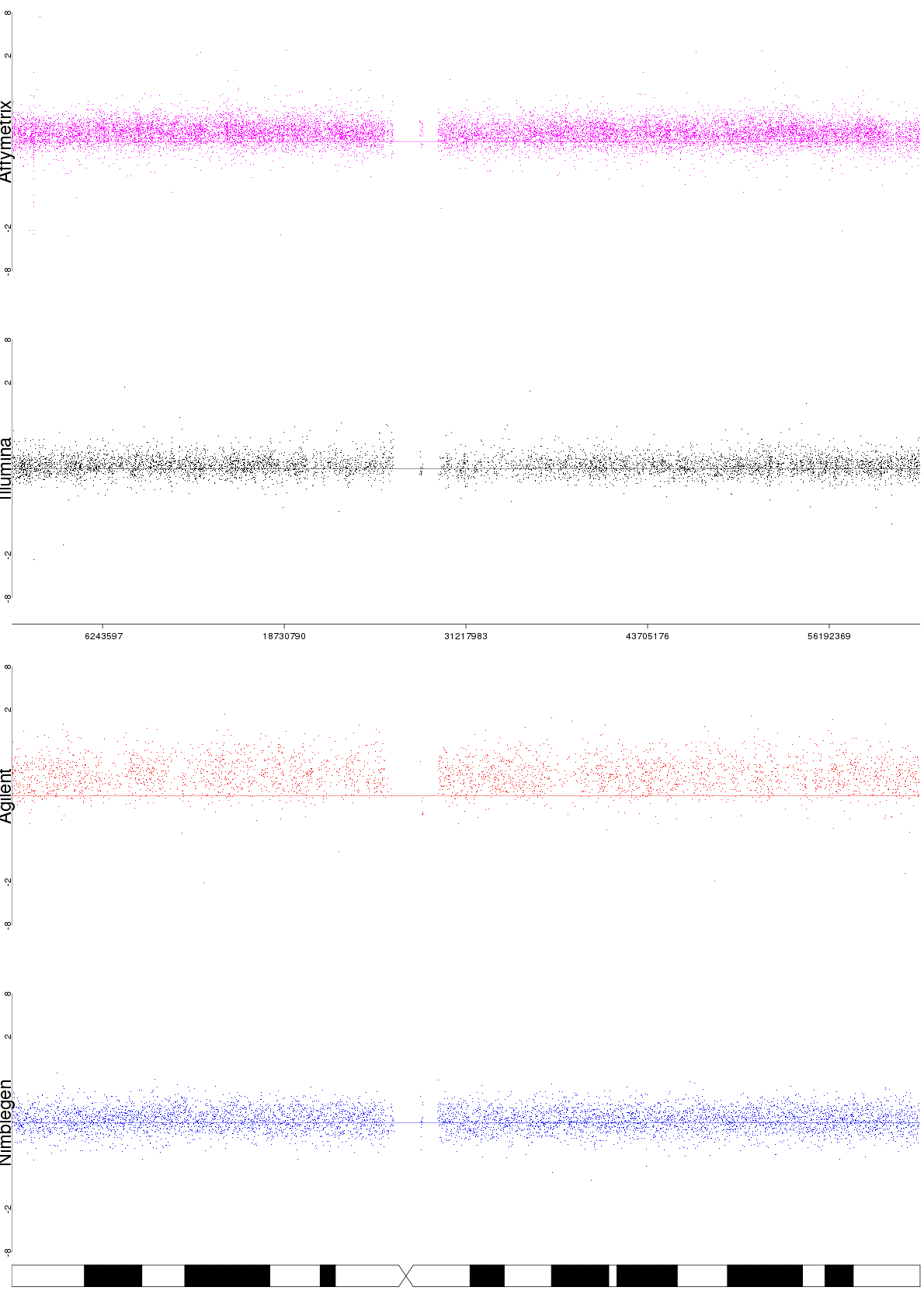

Supplement: Additional file 12 — All sample/chromosome plots for the tumours. Zip folder containing PNGs of all whole-chromosome plots for the tumours. [file 1471-2164-10-588-S12.ZIP › T7201/T7201 chromosome 20.png]

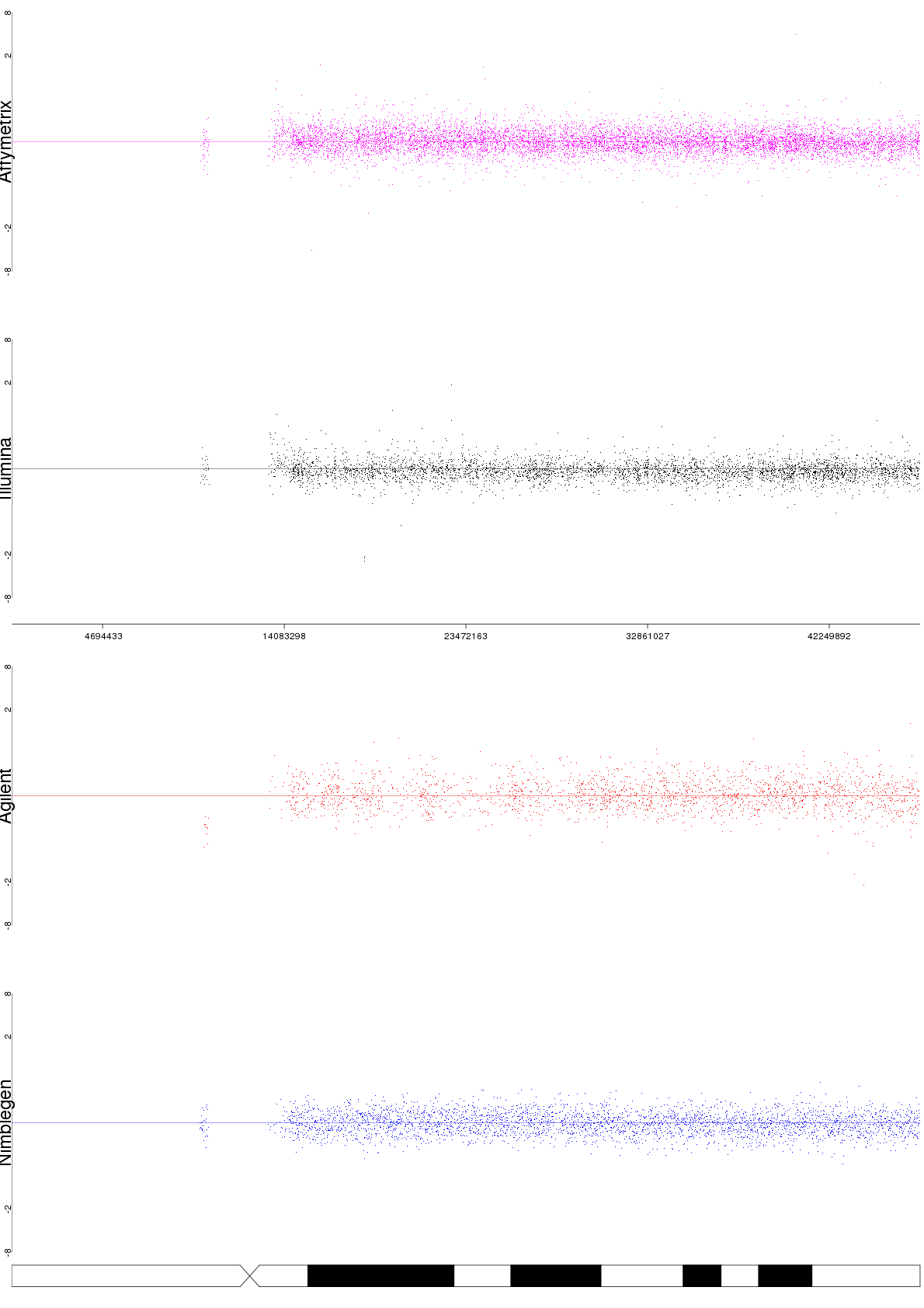

Supplement: Additional file 12 — All sample/chromosome plots for the tumours. Zip folder containing PNGs of all whole-chromosome plots for the tumours. [file 1471-2164-10-588-S12.ZIP › T7201/T7201 chromosome 21.png]

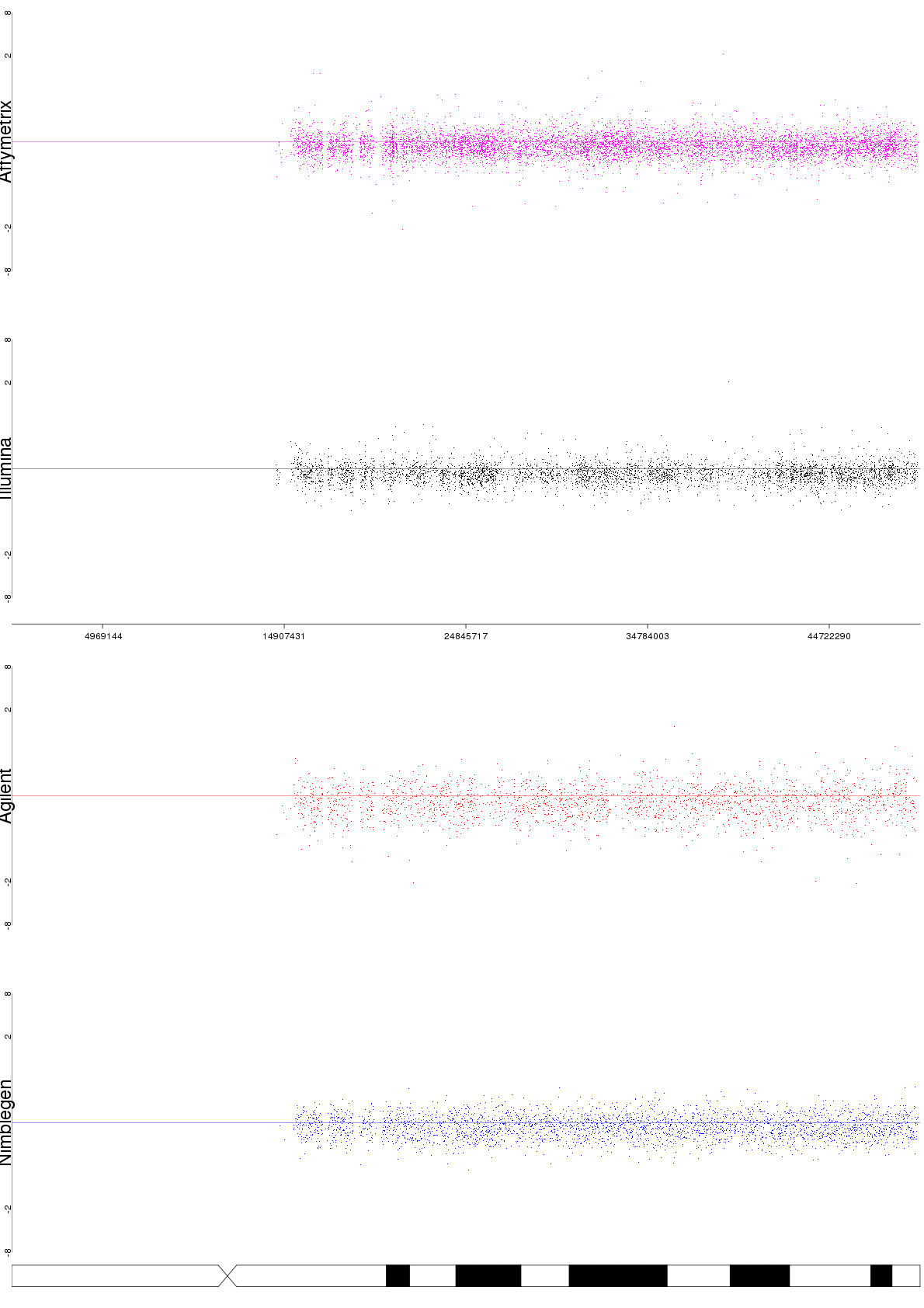

Supplement: Additional file 12 — All sample/chromosome plots for the tumours. Zip folder containing PNGs of all whole-chromosome plots for the tumours. [file 1471-2164-10-588-S12.ZIP › T7201/T7201 chromosome 22.png]

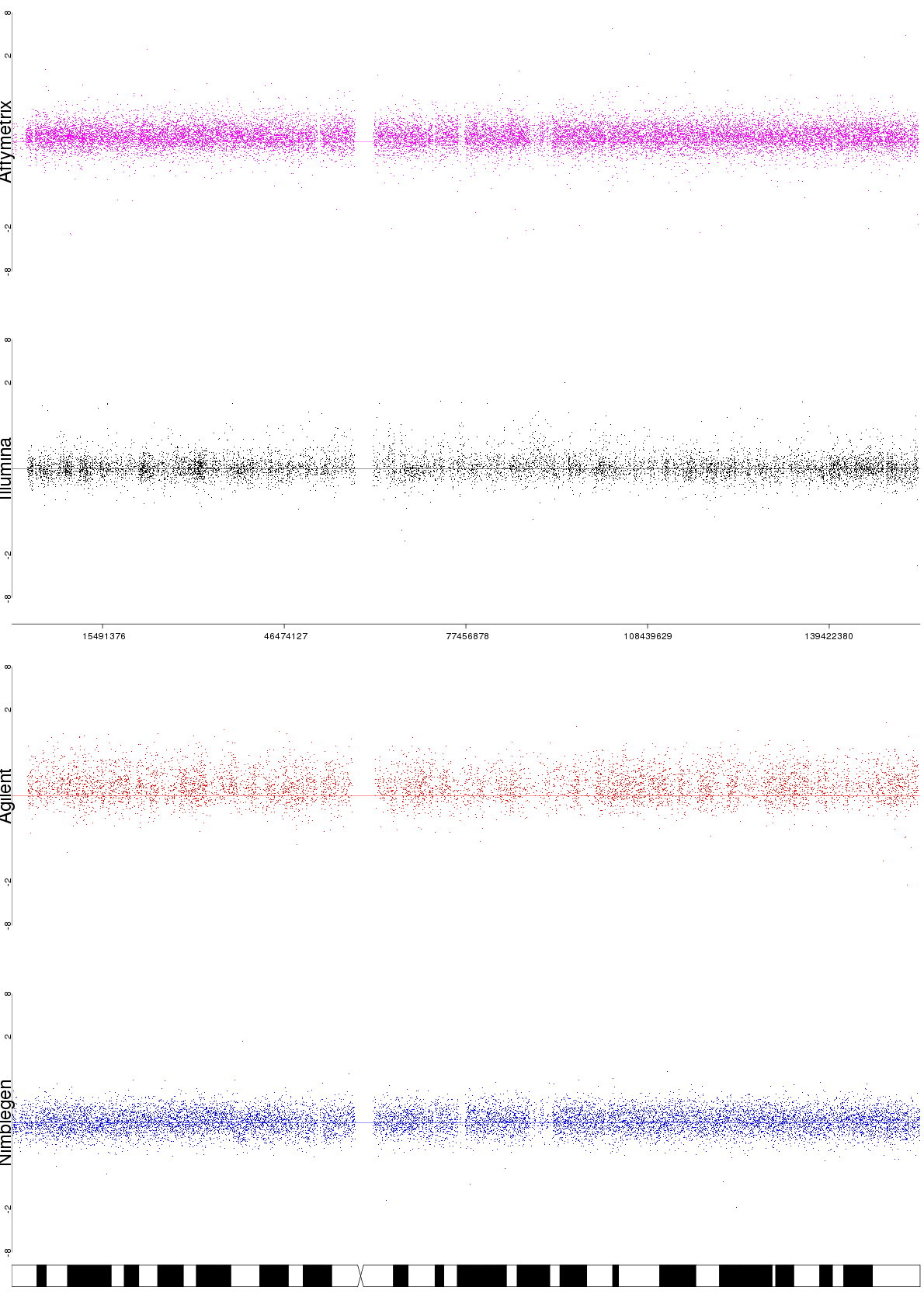

Supplement: Additional file 12 — All sample/chromosome plots for the tumours. Zip folder containing PNGs of all whole-chromosome plots for the tumours. [file 1471-2164-10-588-S12.ZIP › T7201/T7201 chromosome 23.png]

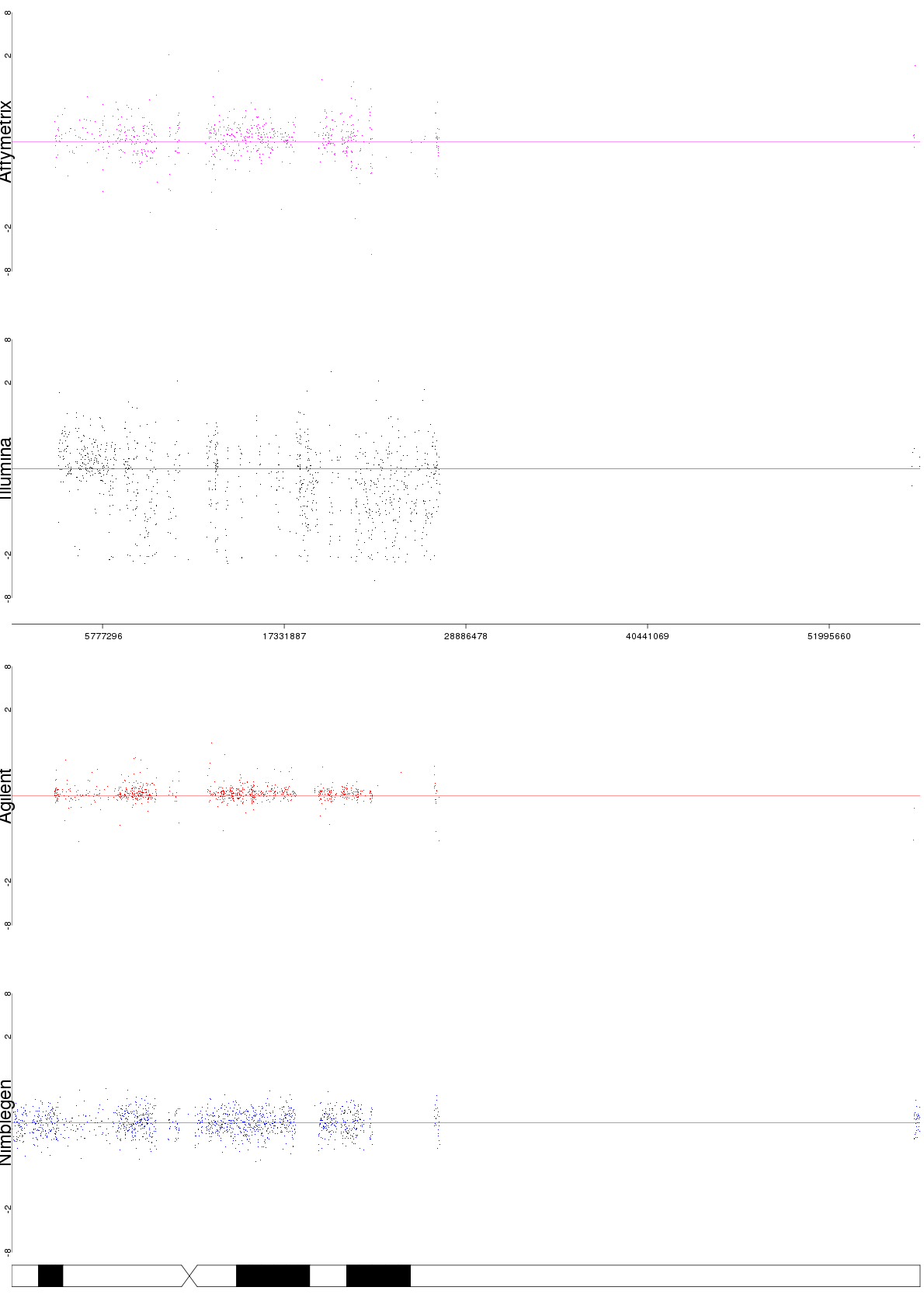

Supplement: Additional file 12 — All sample/chromosome plots for the tumours. Zip folder containing PNGs of all whole-chromosome plots for the tumours. [file 1471-2164-10-588-S12.ZIP › T7201/T7201 chromosome 24.png]

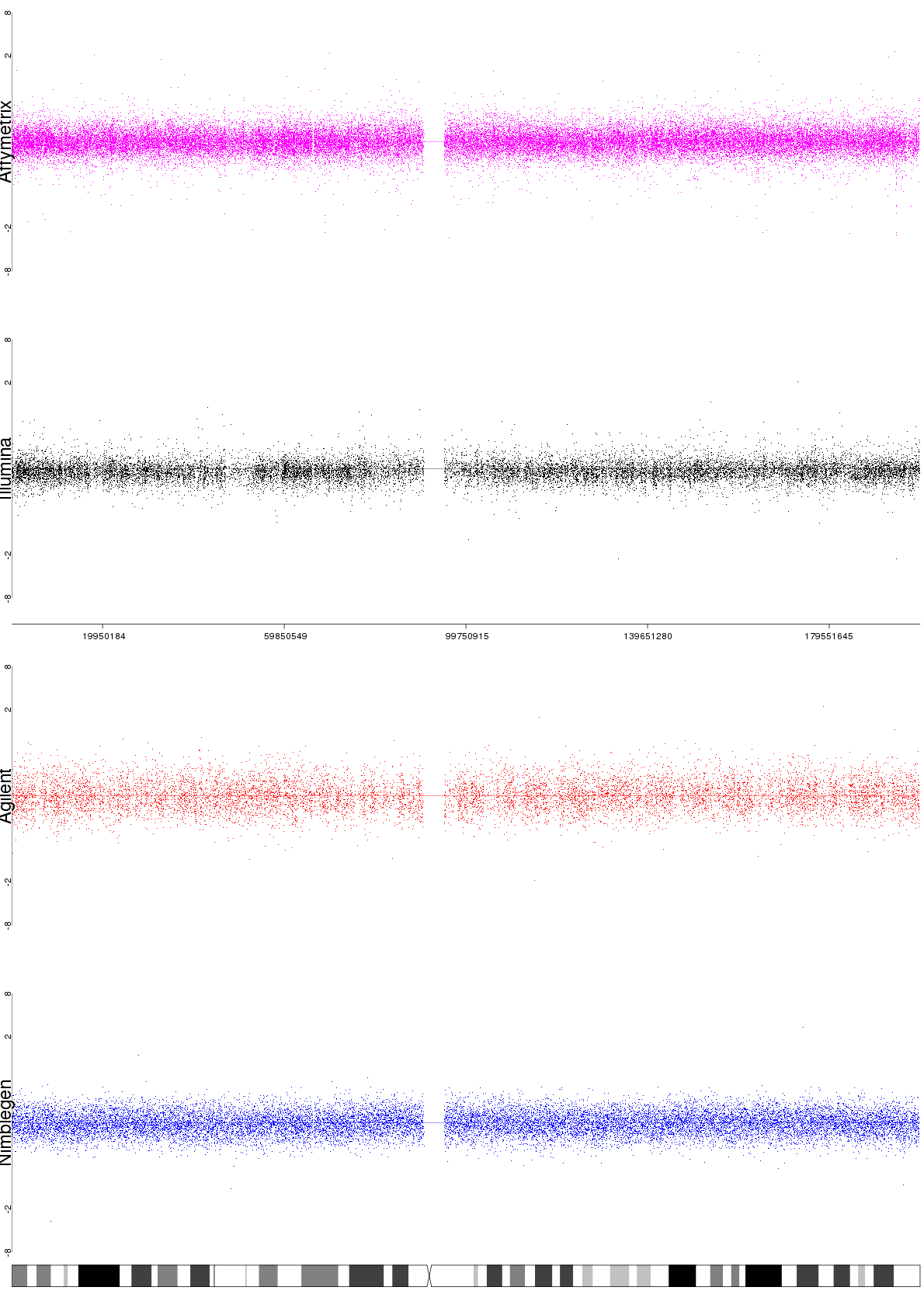

Supplement: Additional file 12 — All sample/chromosome plots for the tumours. Zip folder containing PNGs of all whole-chromosome plots for the tumours. [file 1471-2164-10-588-S12.ZIP › T7201/T7201 chromosome 3.png]

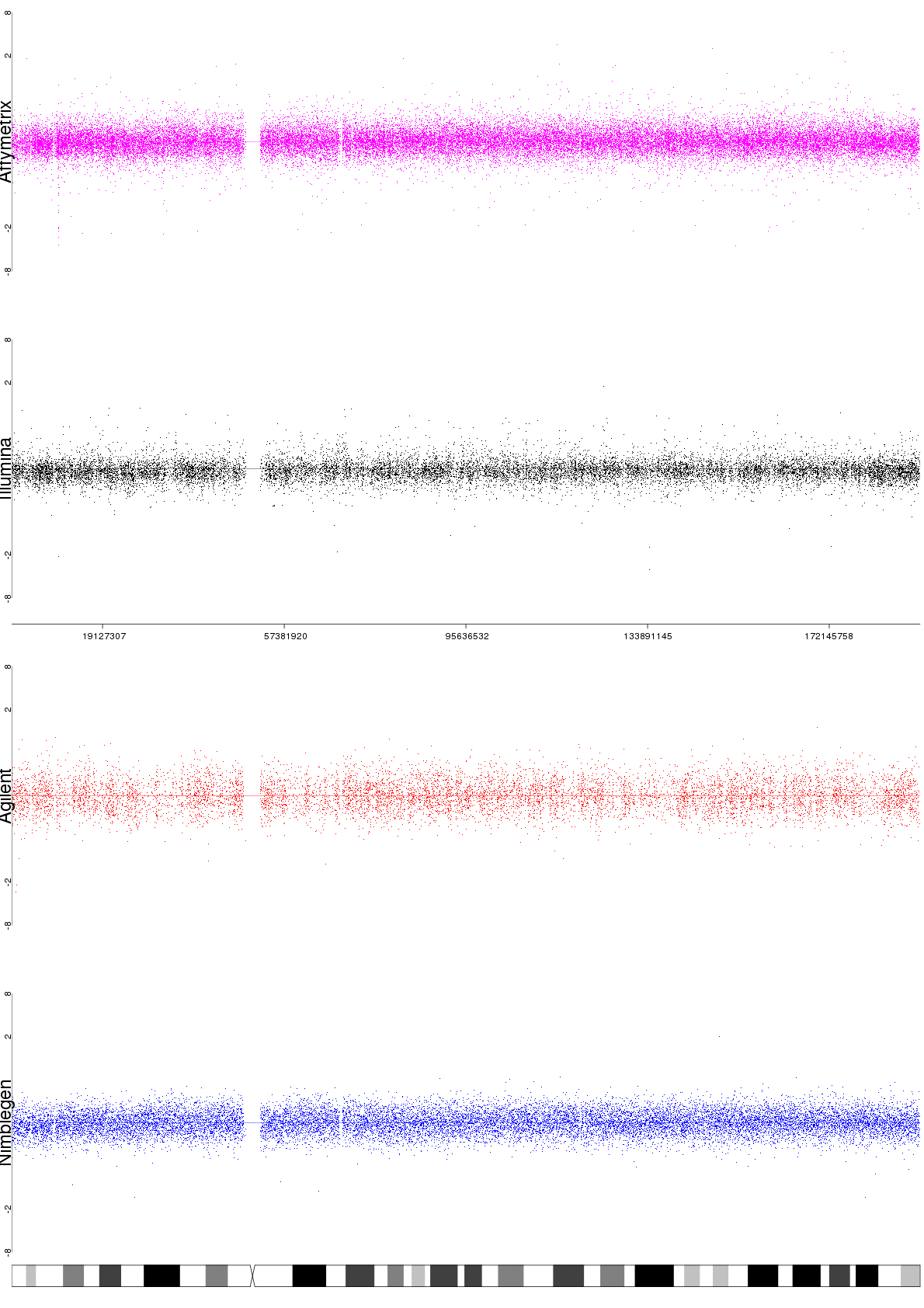

Supplement: Additional file 12 — All sample/chromosome plots for the tumours. Zip folder containing PNGs of all whole-chromosome plots for the tumours. [file 1471-2164-10-588-S12.ZIP › T7201/T7201 chromosome 4.png]

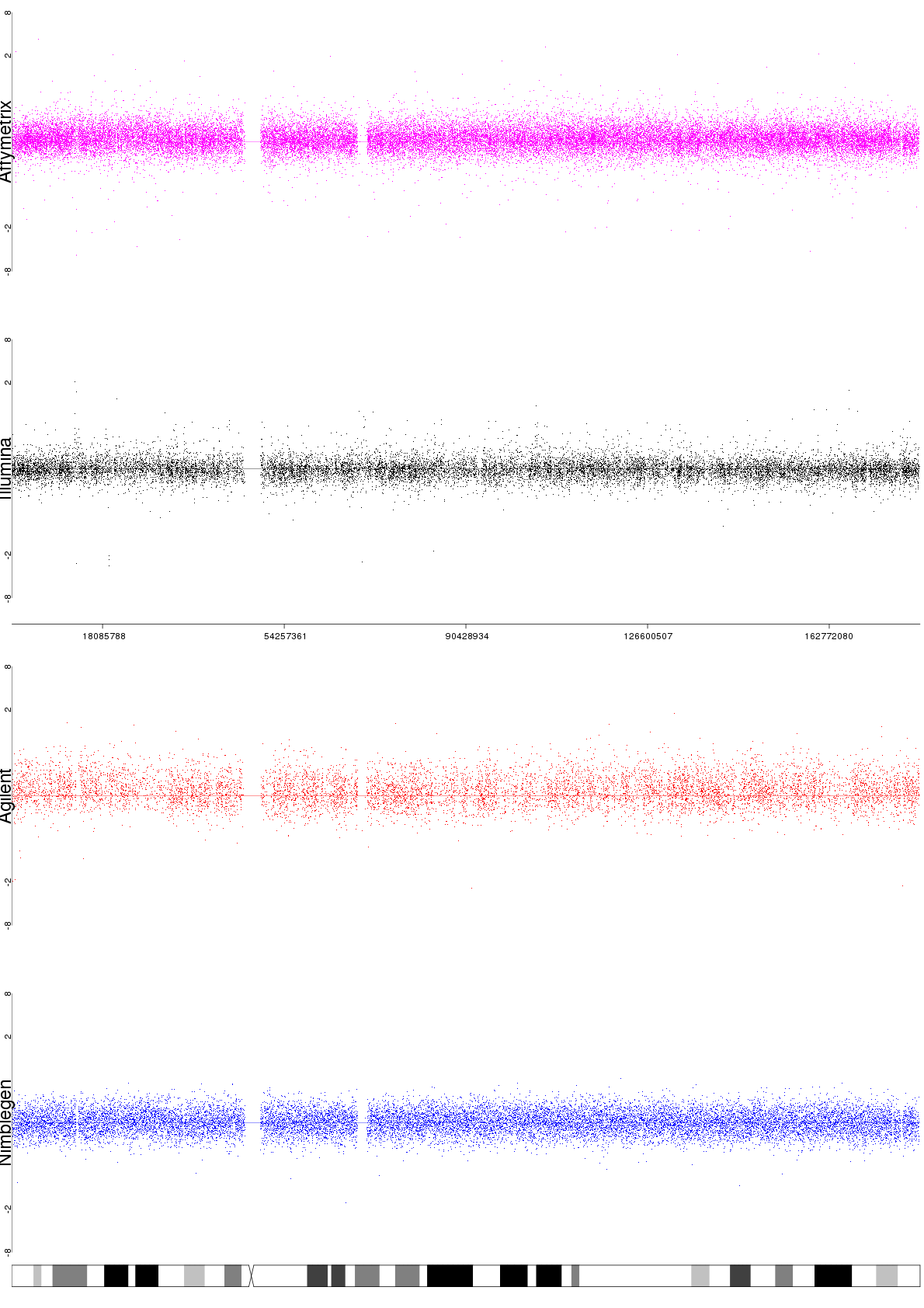

Supplement: Additional file 12 — All sample/chromosome plots for the tumours. Zip folder containing PNGs of all whole-chromosome plots for the tumours. [file 1471-2164-10-588-S12.ZIP › T7201/T7201 chromosome 5.png]

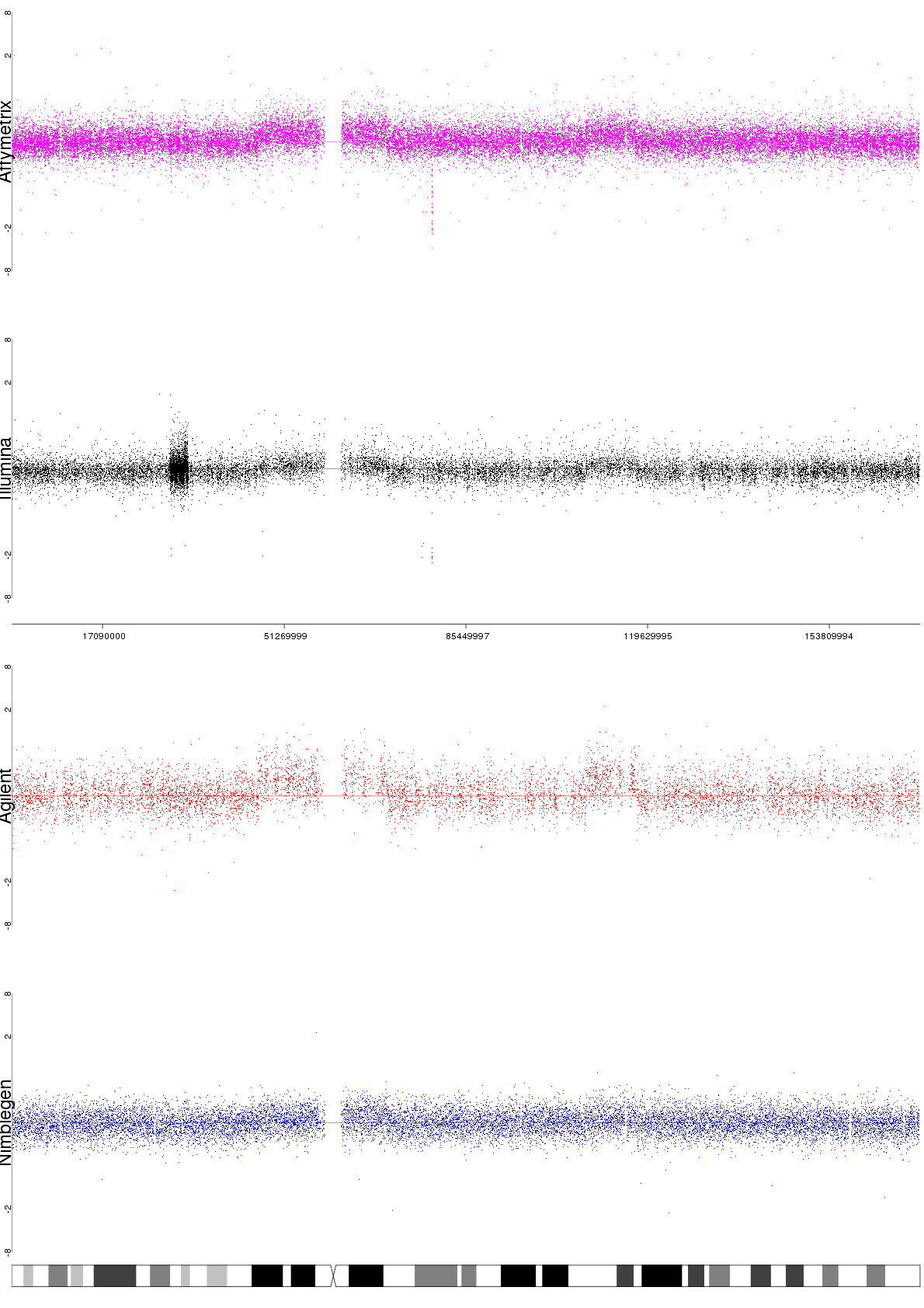

Supplement: Additional file 12 — All sample/chromosome plots for the tumours. Zip folder containing PNGs of all whole-chromosome plots for the tumours. [file 1471-2164-10-588-S12.ZIP › T7201/T7201 chromosome 6.png]

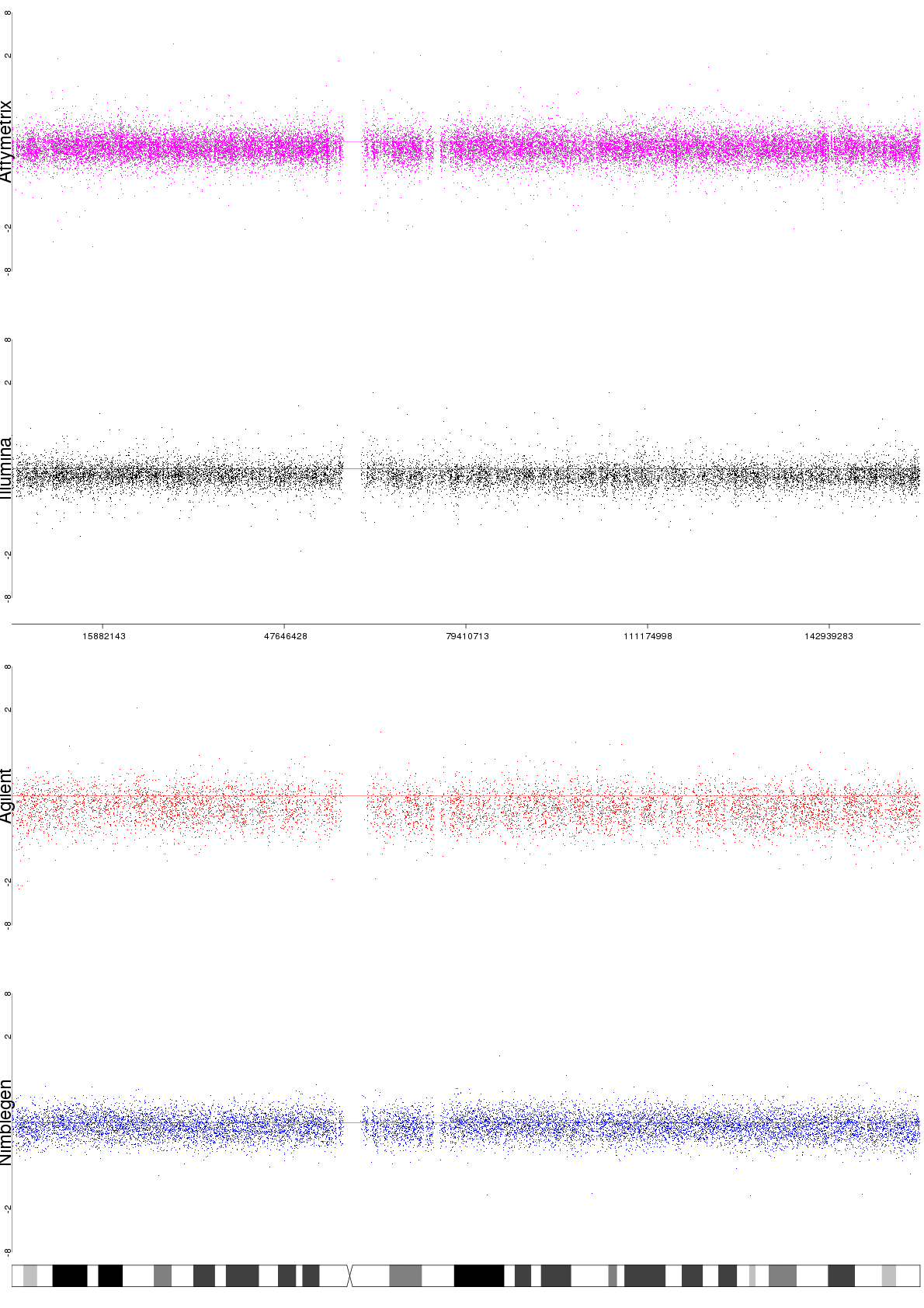

Supplement: Additional file 12 — All sample/chromosome plots for the tumours. Zip folder containing PNGs of all whole-chromosome plots for the tumours. [file 1471-2164-10-588-S12.ZIP › T7201/T7201 chromosome 7.png]

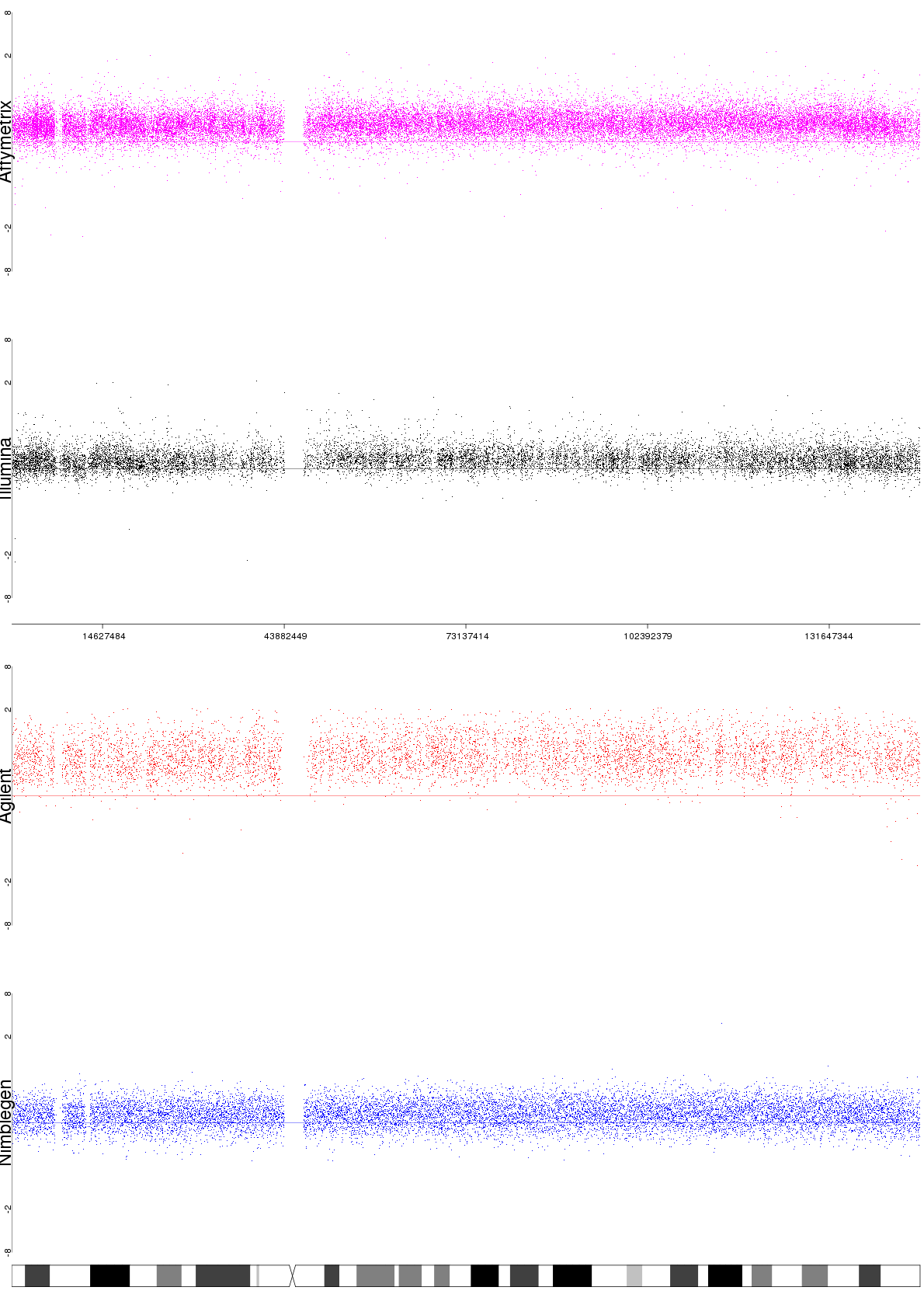

Supplement: Additional file 12 — All sample/chromosome plots for the tumours. Zip folder containing PNGs of all whole-chromosome plots for the tumours. [file 1471-2164-10-588-S12.ZIP › T7201/T7201 chromosome 8.png]

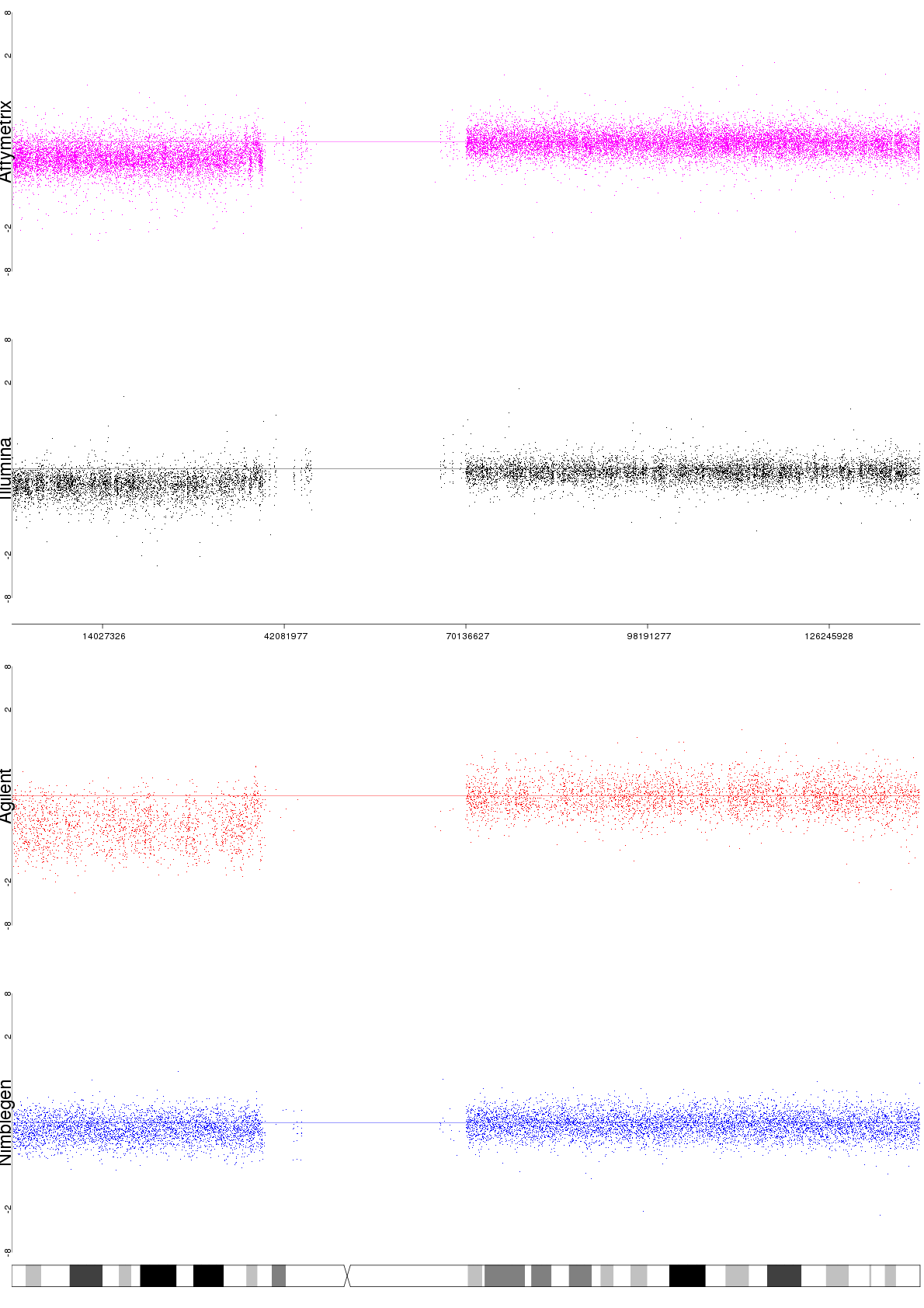

Supplement: Additional file 12 — All sample/chromosome plots for the tumours. Zip folder containing PNGs of all whole-chromosome plots for the tumours. [file 1471-2164-10-588-S12.ZIP › T7201/T7201 chromosome 9.png]

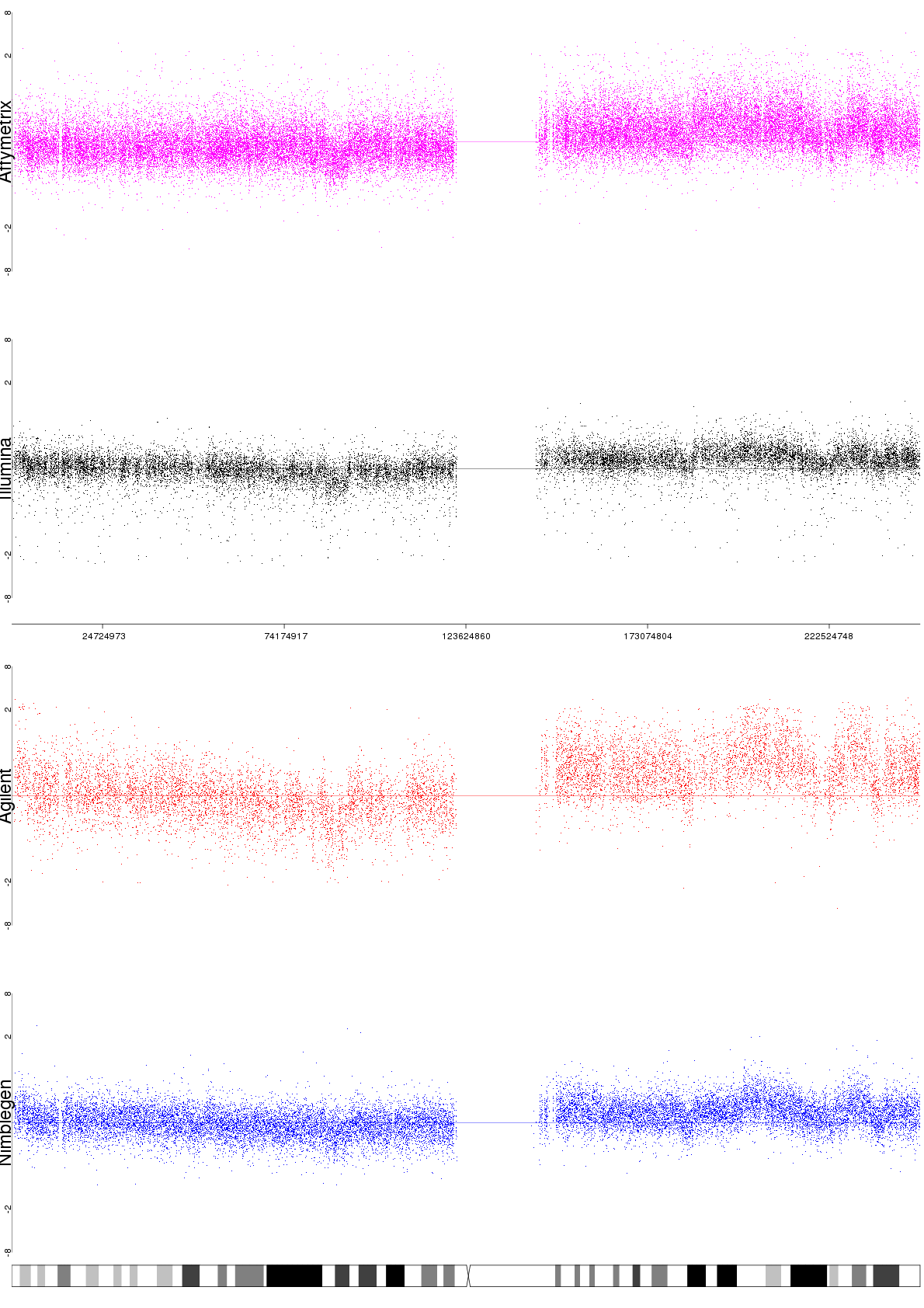

Supplement: Additional file 12 — All sample/chromosome plots for the tumours. Zip folder containing PNGs of all whole-chromosome plots for the tumours. [file 1471-2164-10-588-S12.ZIP › T7204/T7204 chromosome 1.png]

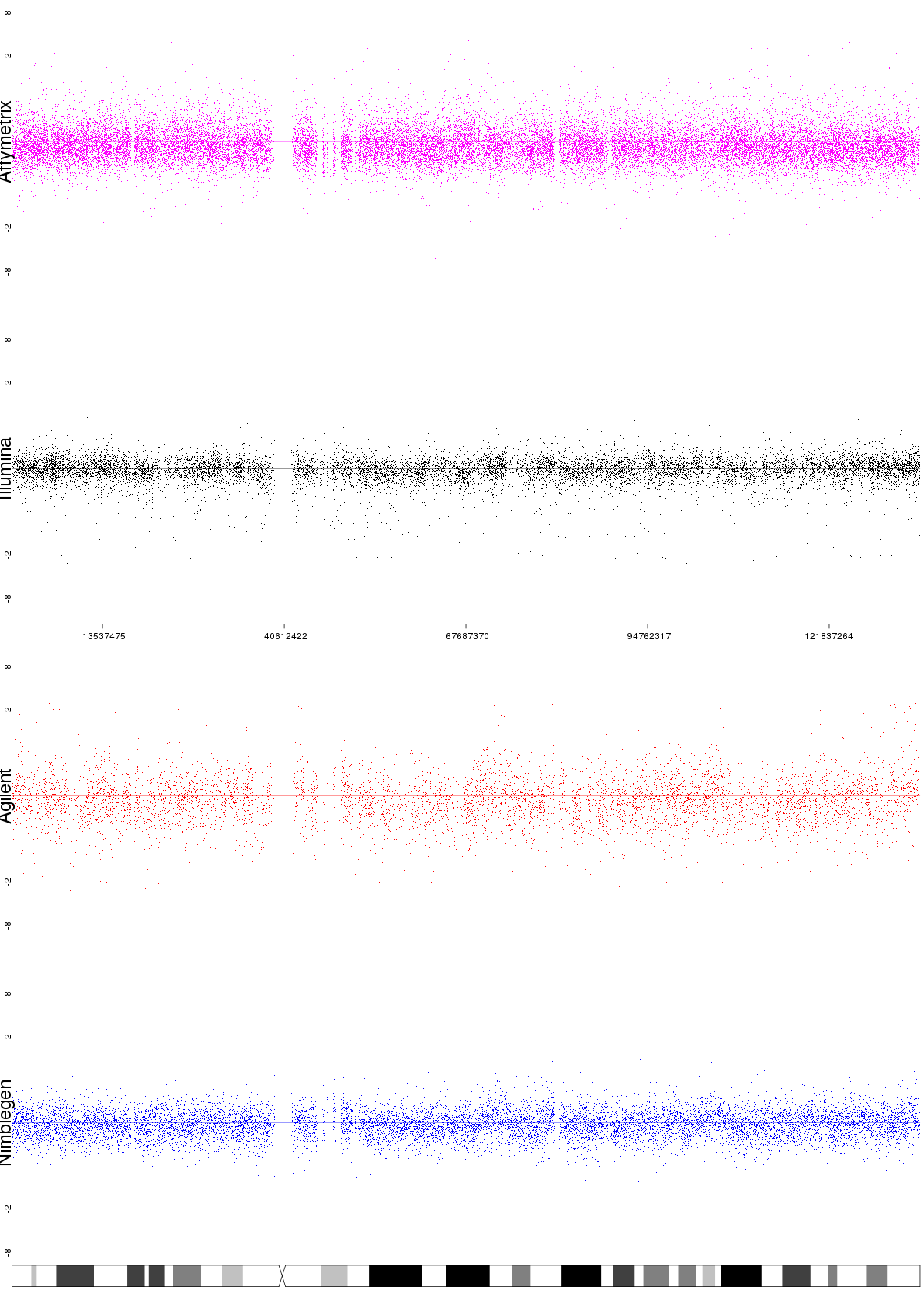

Supplement: Additional file 12 — All sample/chromosome plots for the tumours. Zip folder containing PNGs of all whole-chromosome plots for the tumours. [file 1471-2164-10-588-S12.ZIP › T7204/T7204 chromosome 10.png]

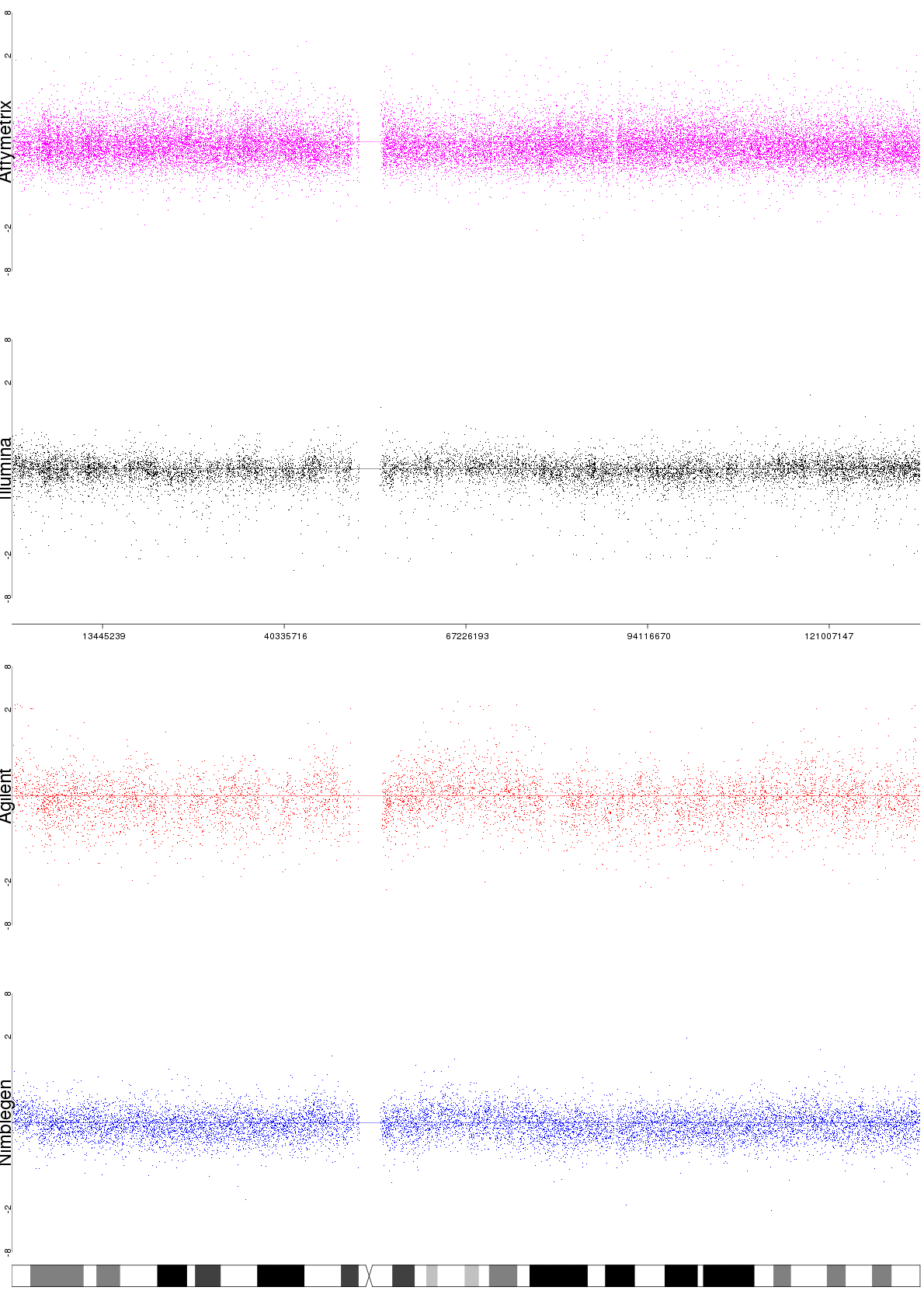

Supplement: Additional file 12 — All sample/chromosome plots for the tumours. Zip folder containing PNGs of all whole-chromosome plots for the tumours. [file 1471-2164-10-588-S12.ZIP › T7204/T7204 chromosome 11.png]

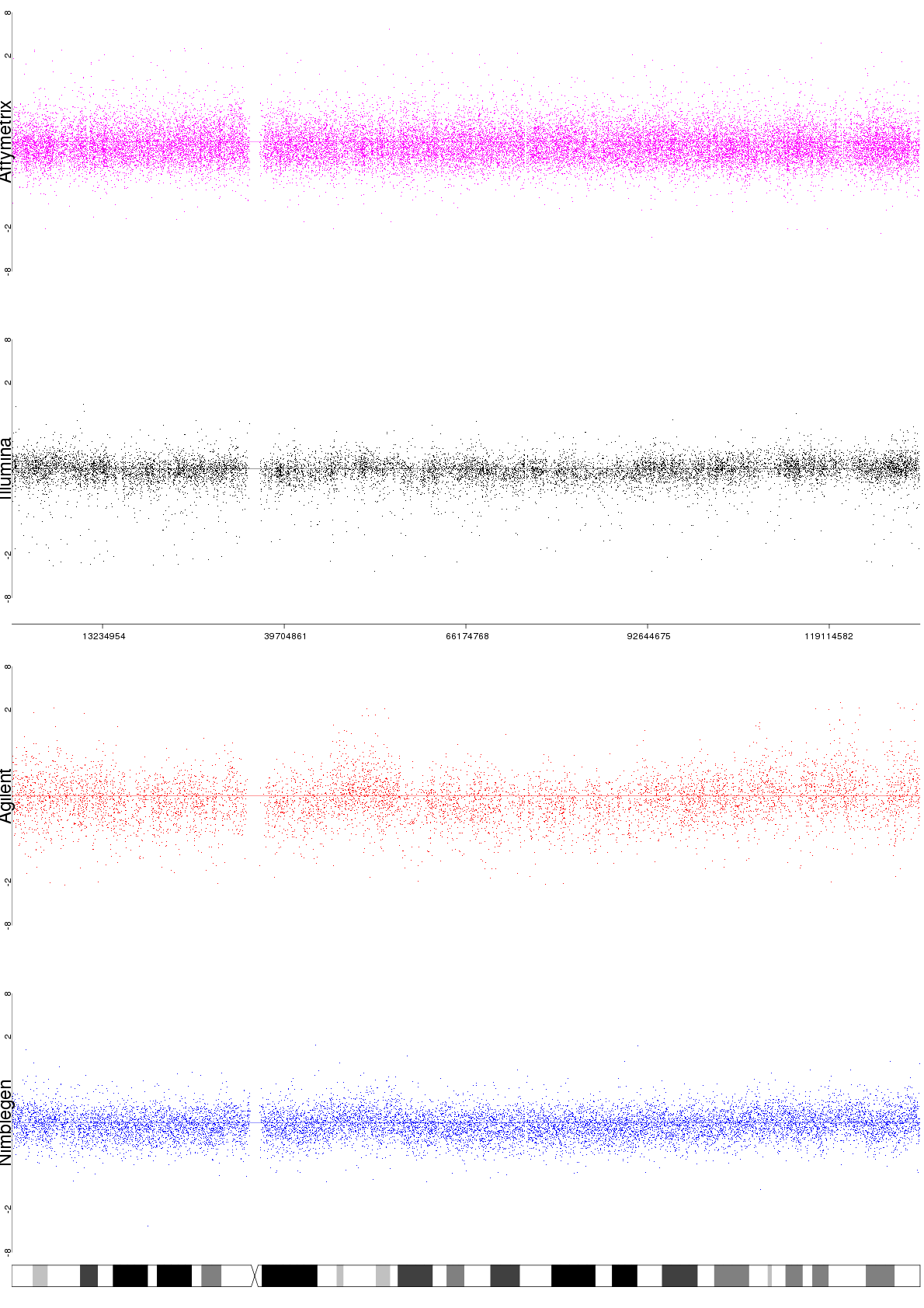

Supplement: Additional file 12 — All sample/chromosome plots for the tumours. Zip folder containing PNGs of all whole-chromosome plots for the tumours. [file 1471-2164-10-588-S12.ZIP › T7204/T7204 chromosome 12.png]

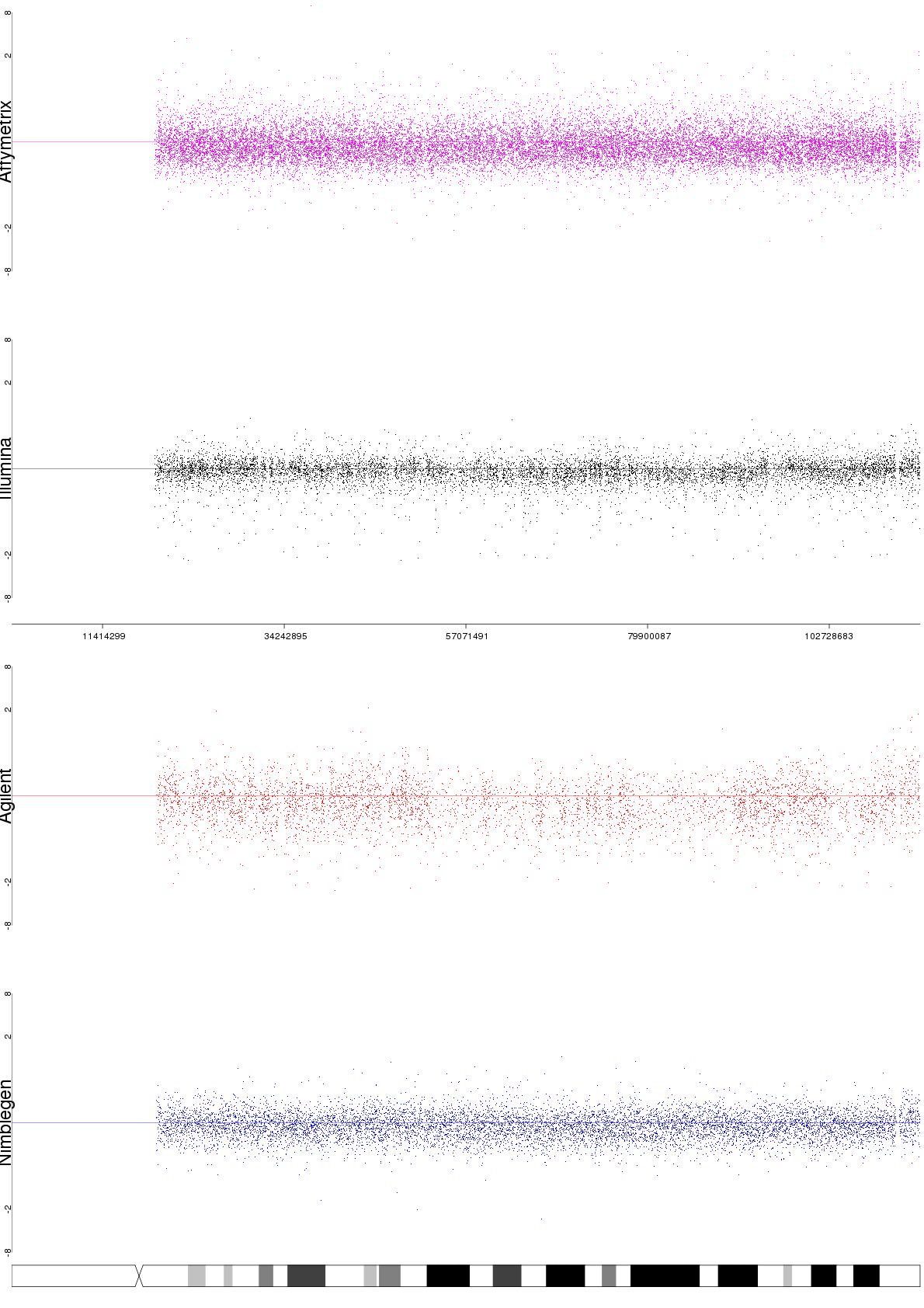

Supplement: Additional file 12 — All sample/chromosome plots for the tumours. Zip folder containing PNGs of all whole-chromosome plots for the tumours. [file 1471-2164-10-588-S12.ZIP › T7204/T7204 chromosome 13.png]

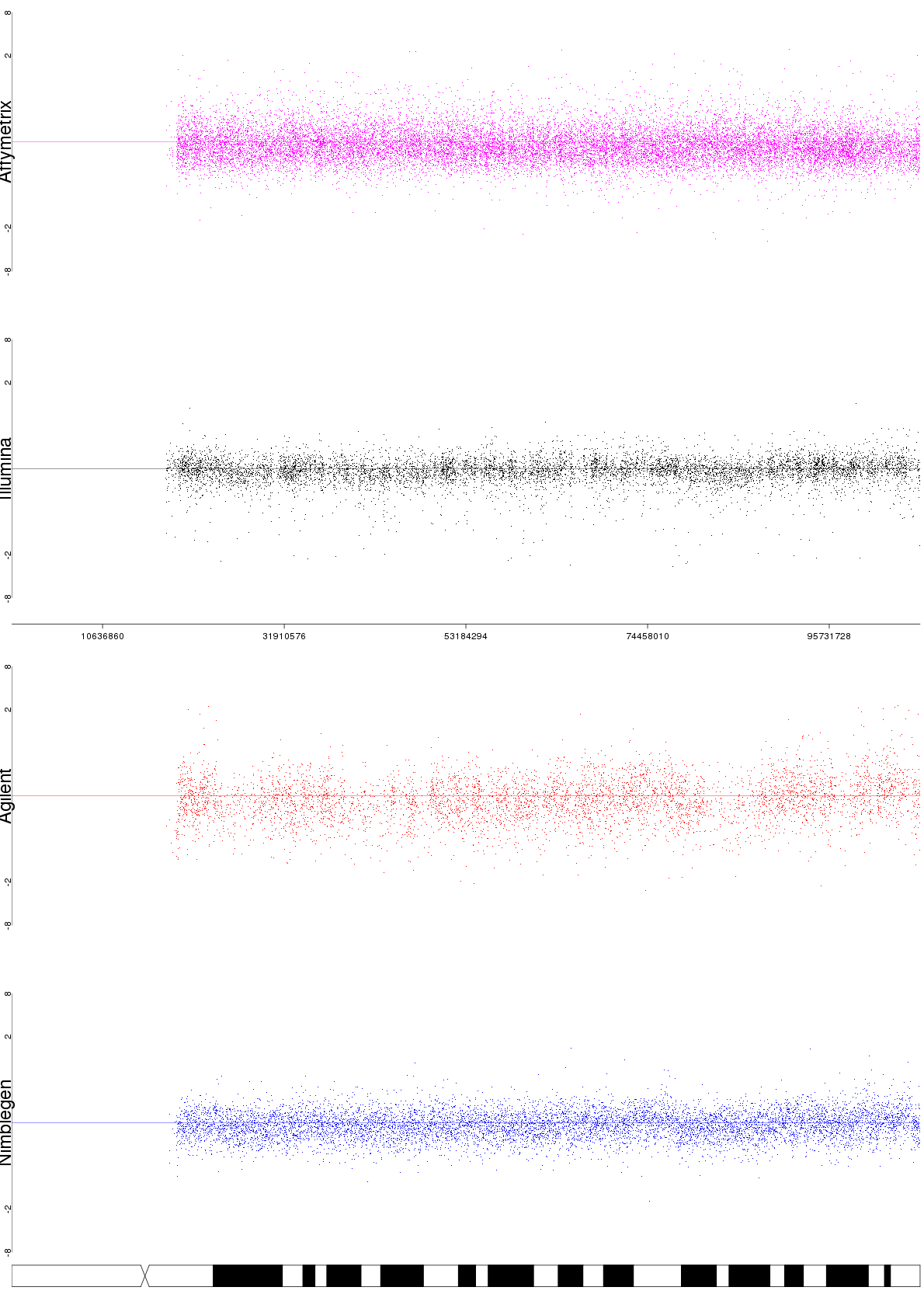

Supplement: Additional file 12 — All sample/chromosome plots for the tumours. Zip folder containing PNGs of all whole-chromosome plots for the tumours. [file 1471-2164-10-588-S12.ZIP › T7204/T7204 chromosome 14.png]

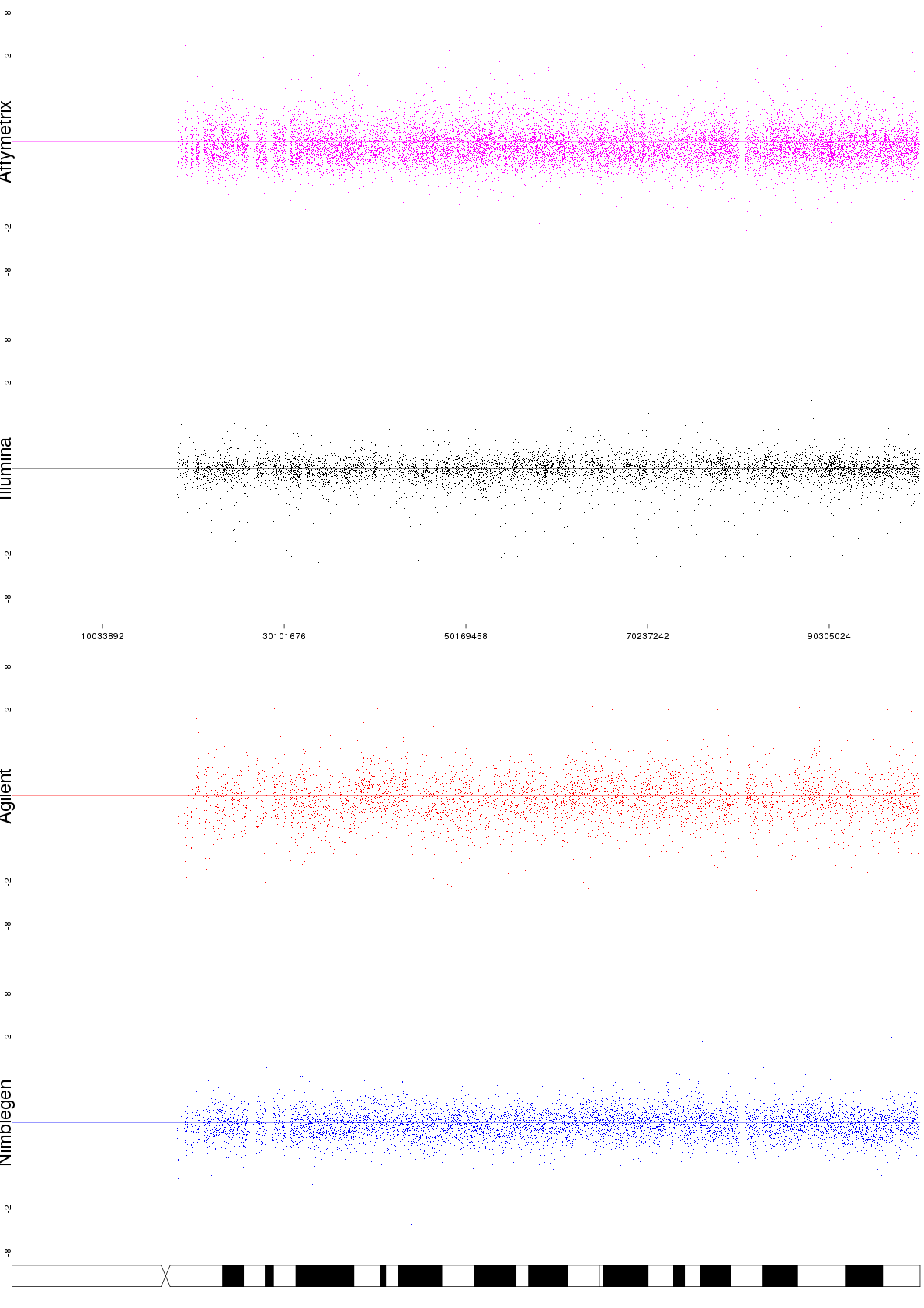

Supplement: Additional file 12 — All sample/chromosome plots for the tumours. Zip folder containing PNGs of all whole-chromosome plots for the tumours. [file 1471-2164-10-588-S12.ZIP › T7204/T7204 chromosome 15.png]

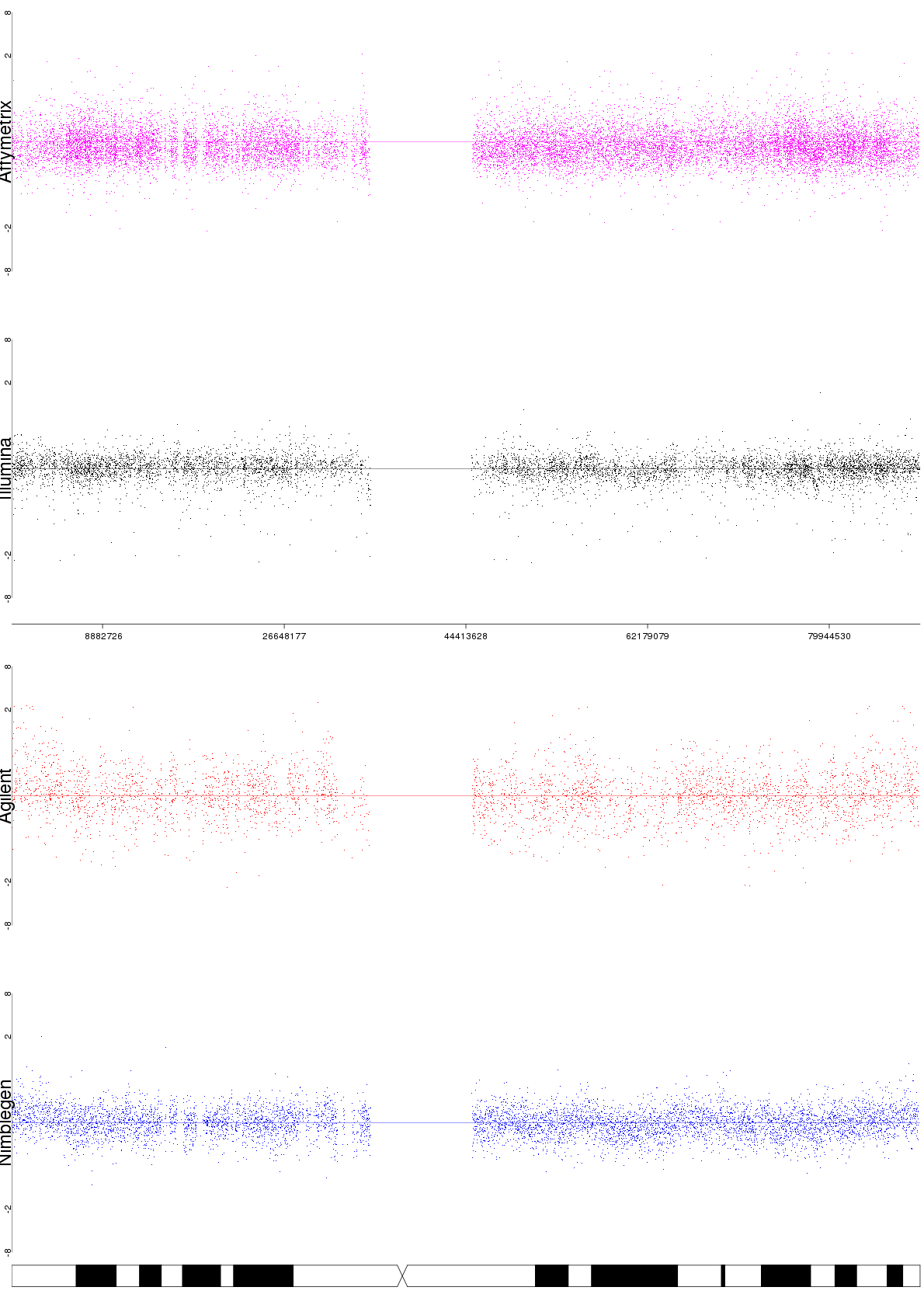

Supplement: Additional file 12 — All sample/chromosome plots for the tumours. Zip folder containing PNGs of all whole-chromosome plots for the tumours. [file 1471-2164-10-588-S12.ZIP › T7204/T7204 chromosome 16.png]

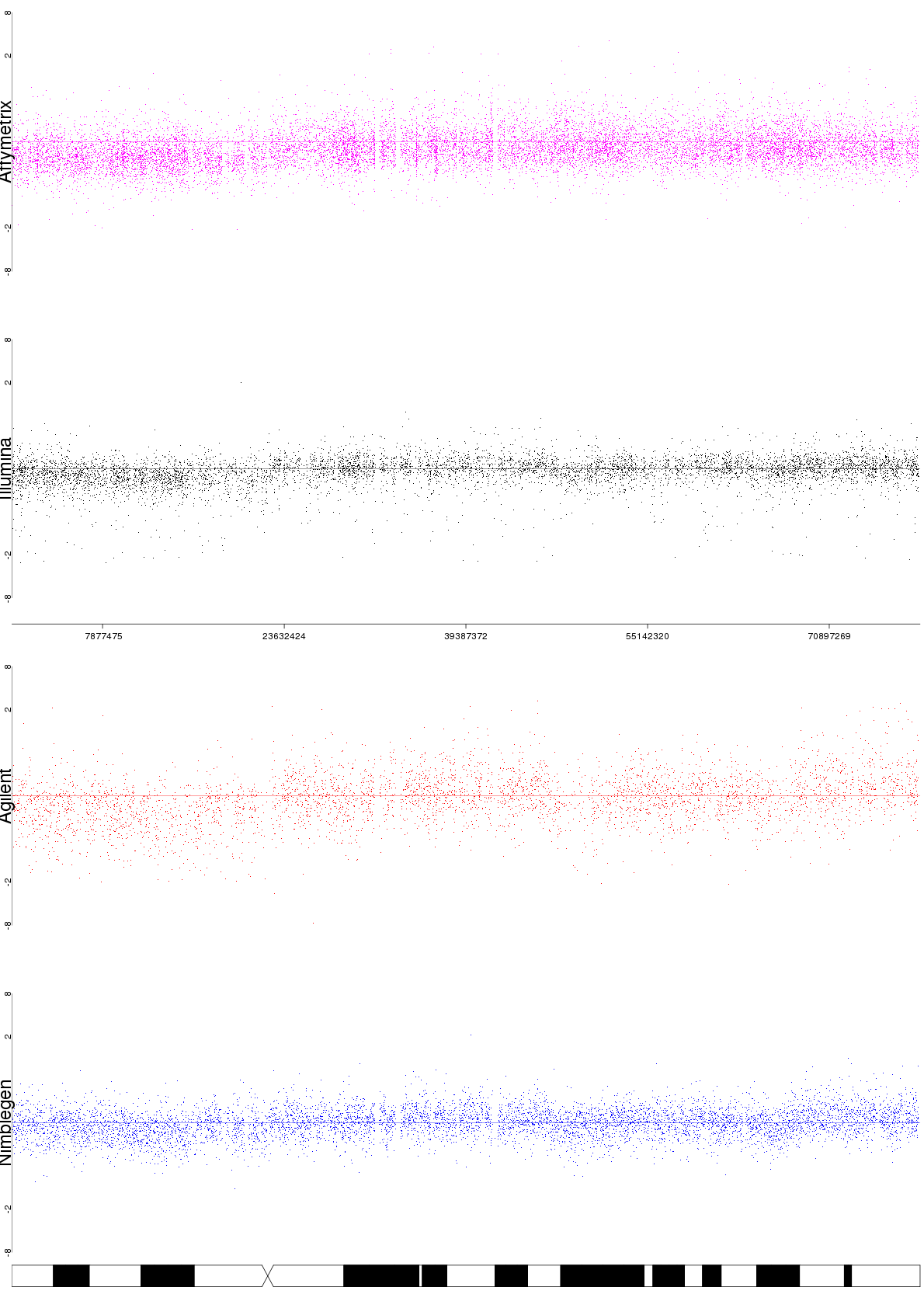

Supplement: Additional file 12 — All sample/chromosome plots for the tumours. Zip folder containing PNGs of all whole-chromosome plots for the tumours. [file 1471-2164-10-588-S12.ZIP › T7204/T7204 chromosome 17.png]

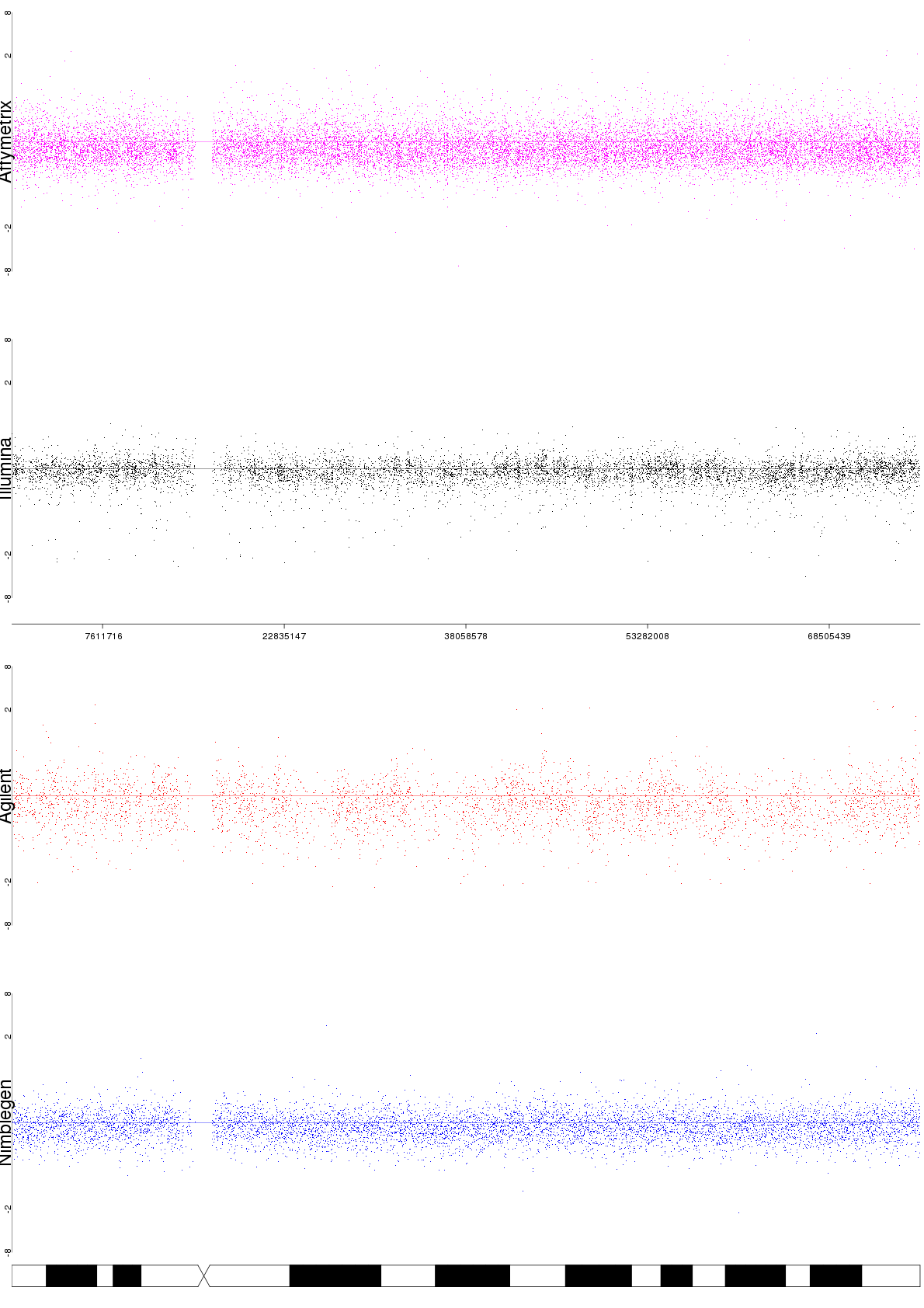

Supplement: Additional file 12 — All sample/chromosome plots for the tumours. Zip folder containing PNGs of all whole-chromosome plots for the tumours. [file 1471-2164-10-588-S12.ZIP › T7204/T7204 chromosome 18.png]

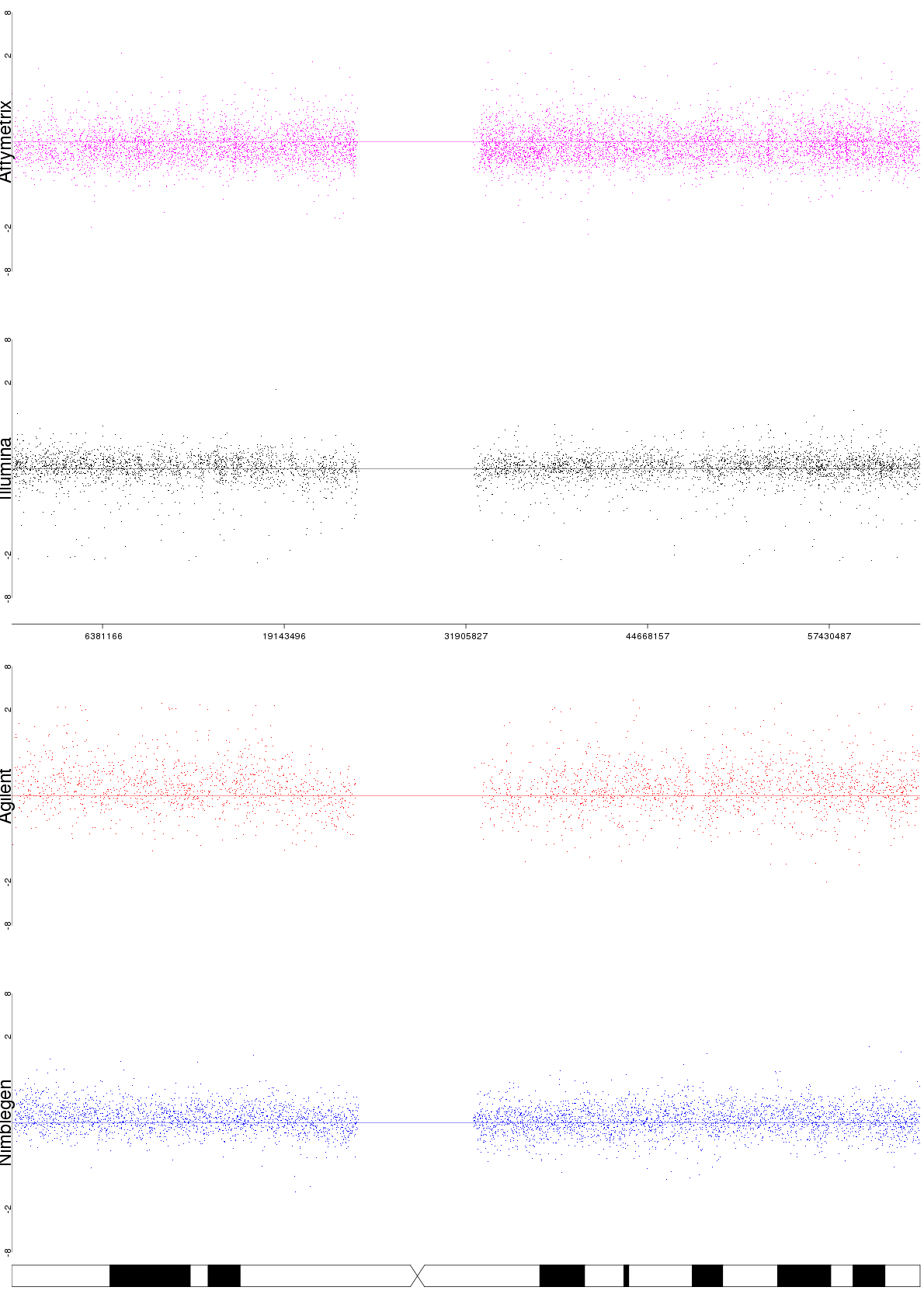

Supplement: Additional file 12 — All sample/chromosome plots for the tumours. Zip folder containing PNGs of all whole-chromosome plots for the tumours. [file 1471-2164-10-588-S12.ZIP › T7204/T7204 chromosome 19.png]

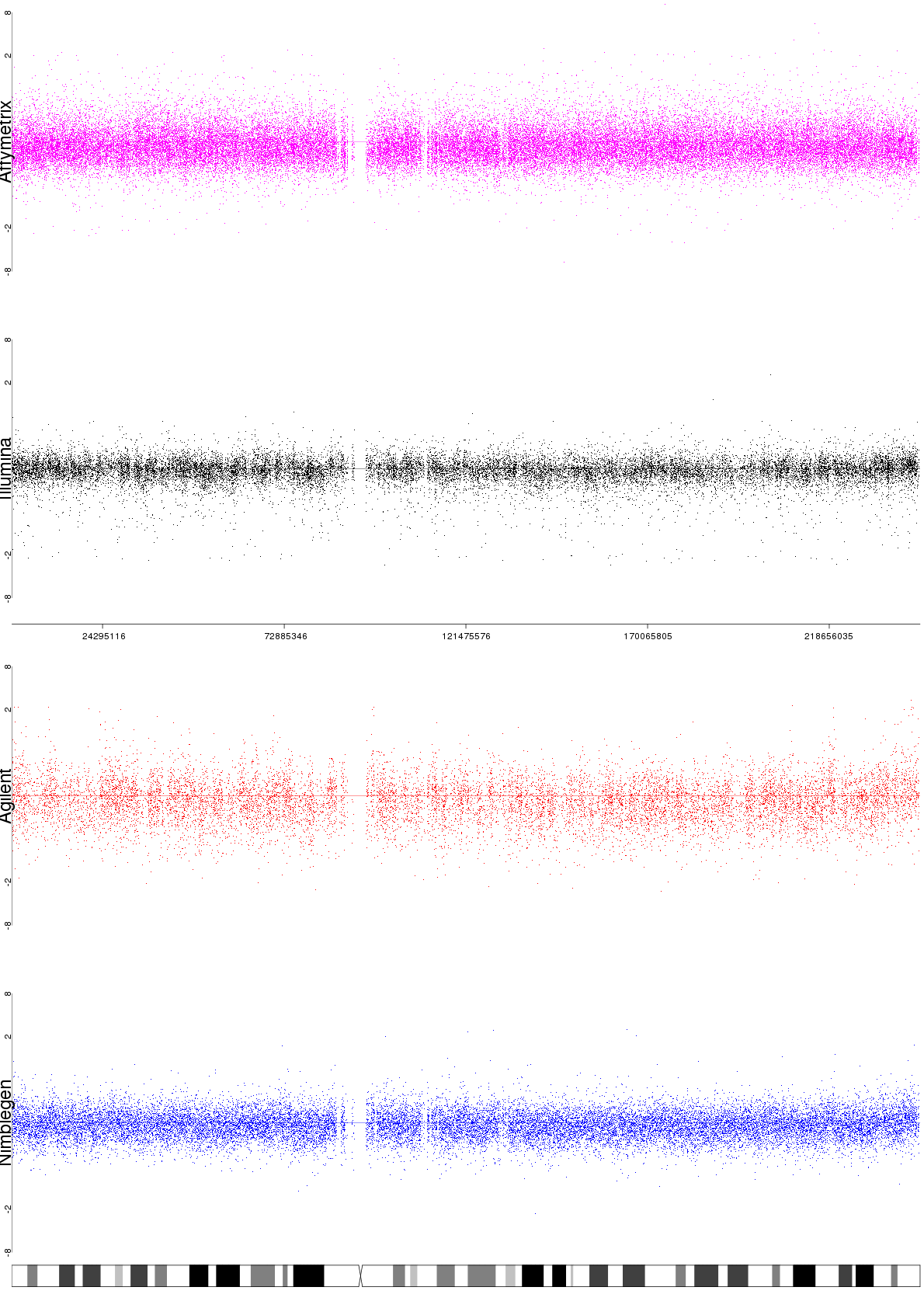

Supplement: Additional file 12 — All sample/chromosome plots for the tumours. Zip folder containing PNGs of all whole-chromosome plots for the tumours. [file 1471-2164-10-588-S12.ZIP › T7204/T7204 chromosome 2.png]

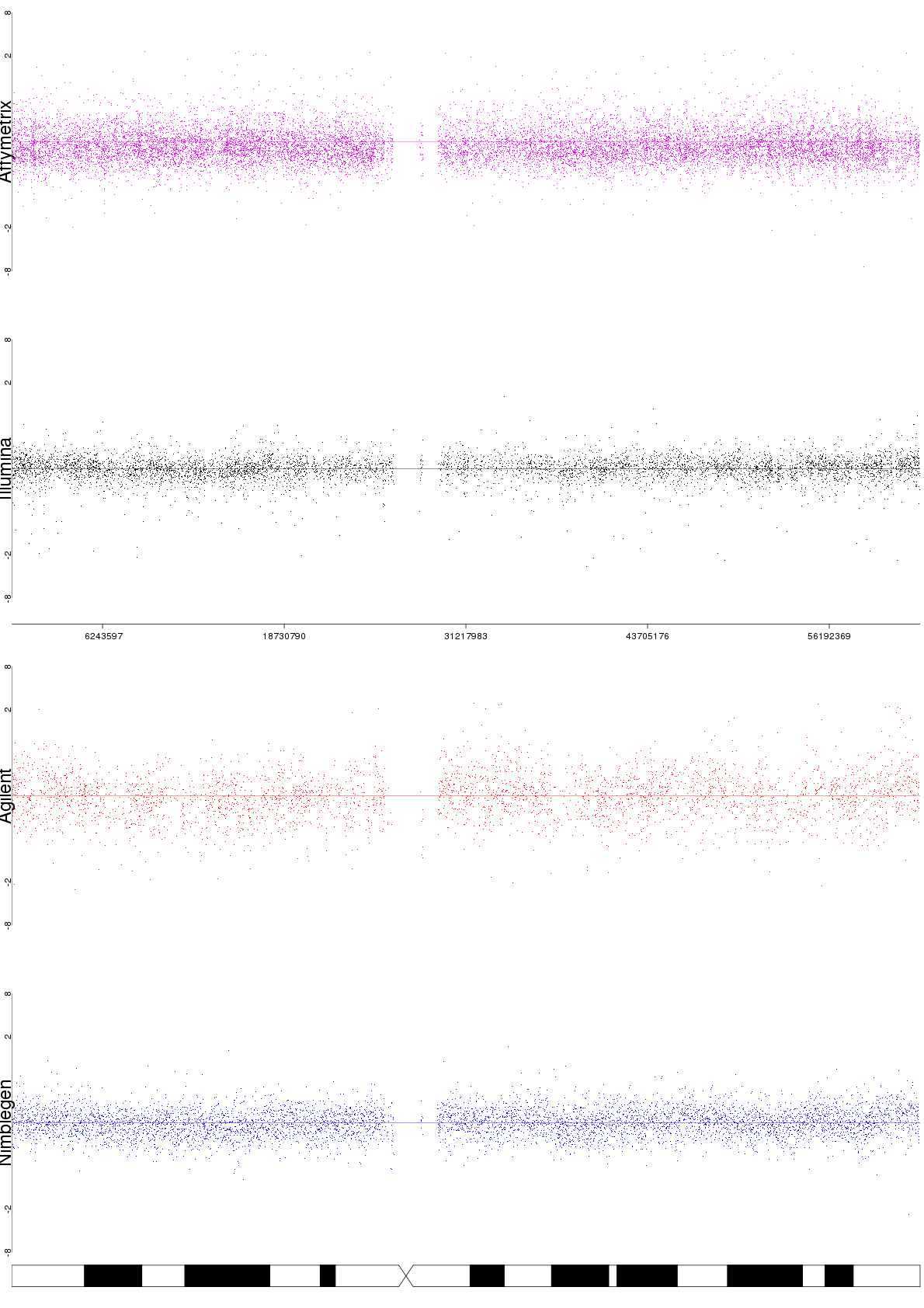

Supplement: Additional file 12 — All sample/chromosome plots for the tumours. Zip folder containing PNGs of all whole-chromosome plots for the tumours. [file 1471-2164-10-588-S12.ZIP › T7204/T7204 chromosome 20.png]

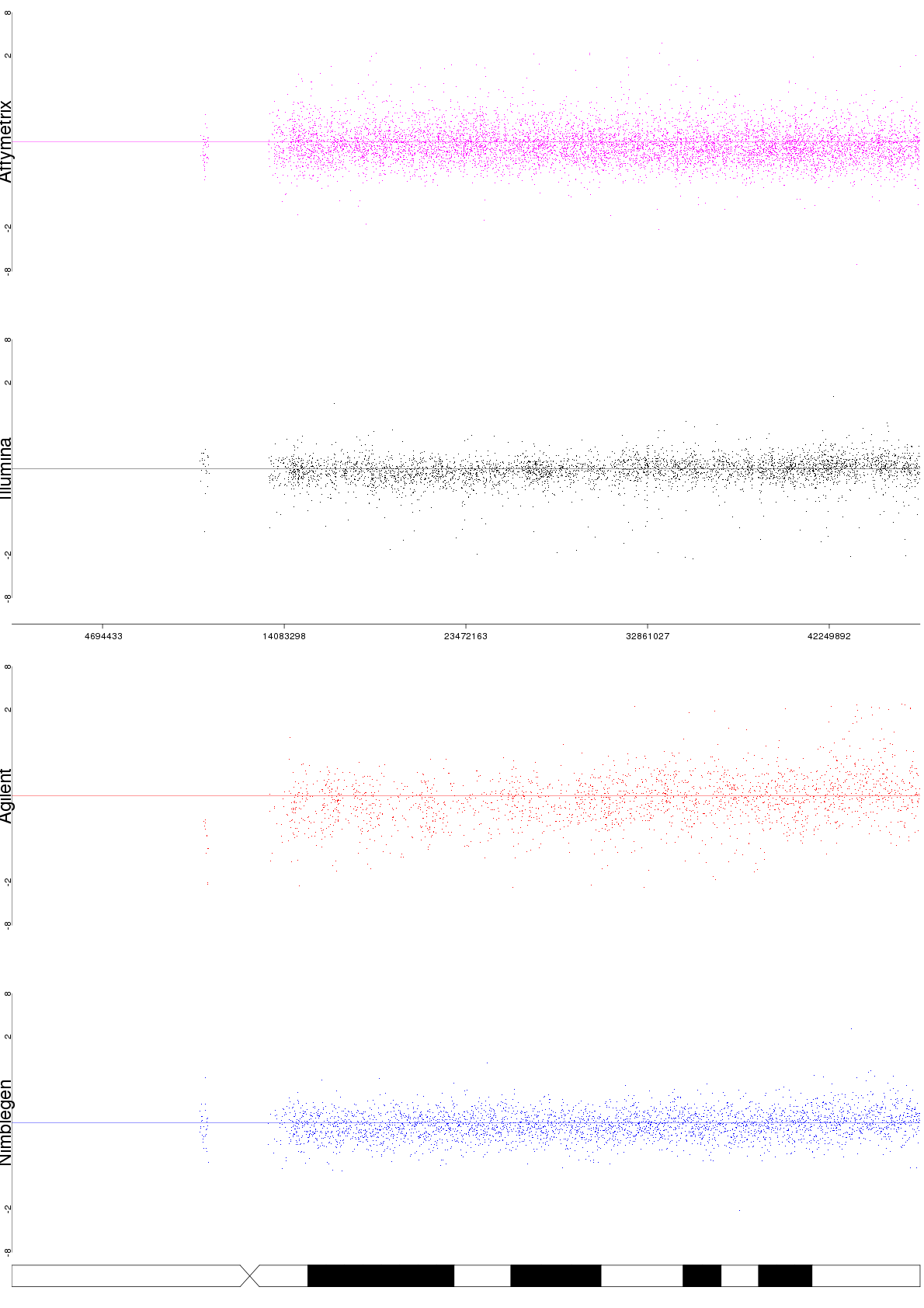

Supplement: Additional file 12 — All sample/chromosome plots for the tumours. Zip folder containing PNGs of all whole-chromosome plots for the tumours. [file 1471-2164-10-588-S12.ZIP › T7204/T7204 chromosome 21.png]

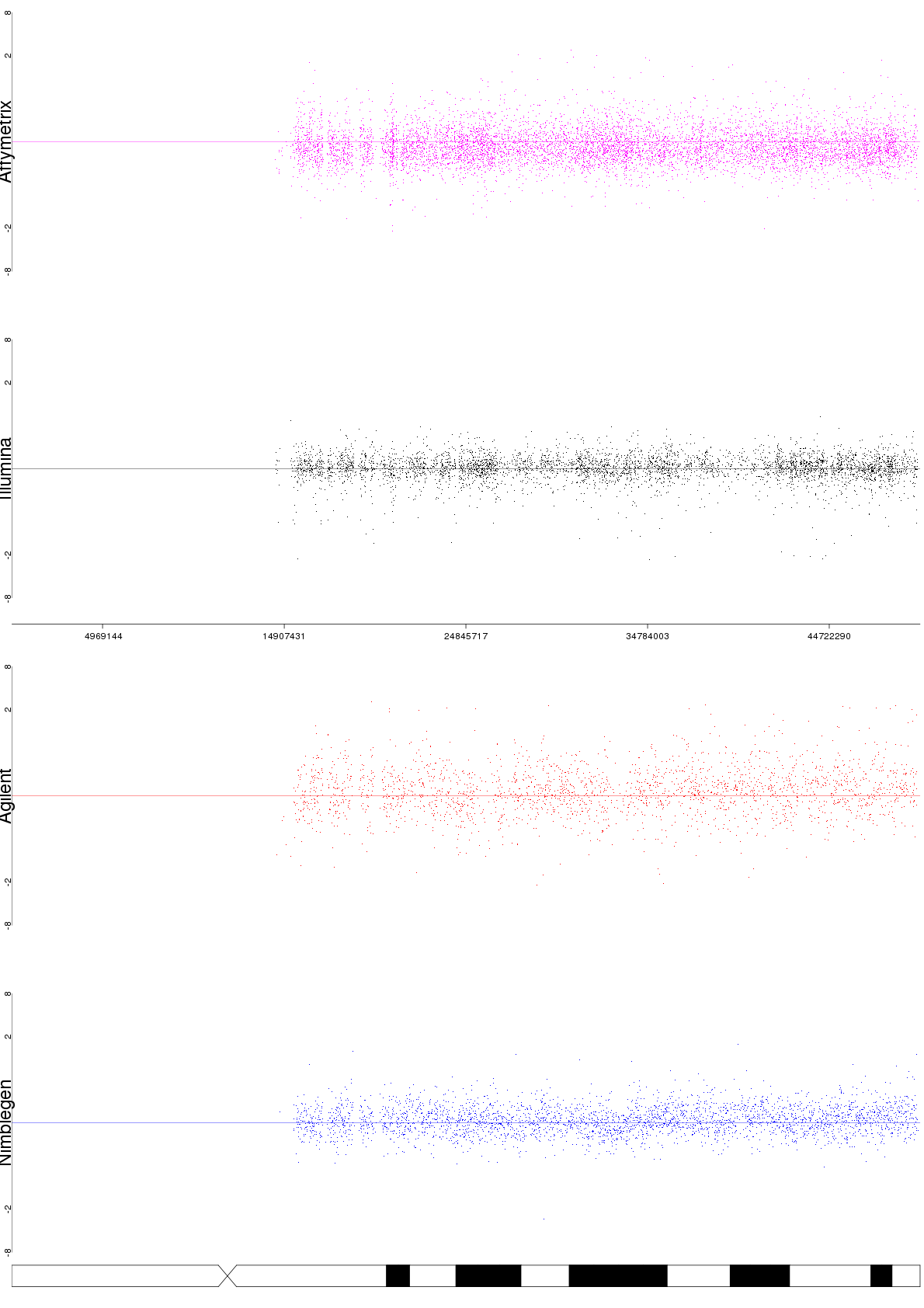

Supplement: Additional file 12 — All sample/chromosome plots for the tumours. Zip folder containing PNGs of all whole-chromosome plots for the tumours. [file 1471-2164-10-588-S12.ZIP › T7204/T7204 chromosome 22.png]

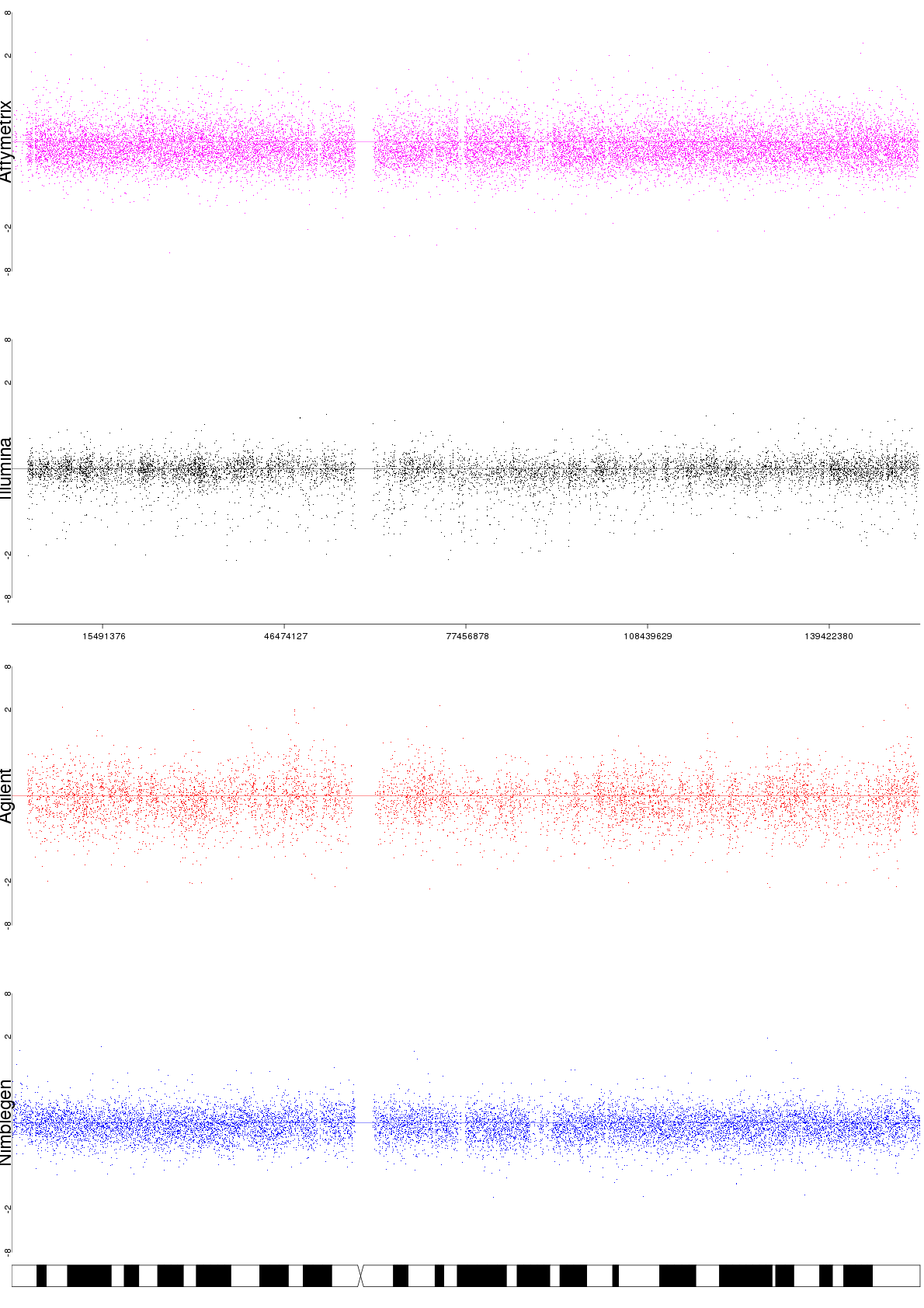

Supplement: Additional file 12 — All sample/chromosome plots for the tumours. Zip folder containing PNGs of all whole-chromosome plots for the tumours. [file 1471-2164-10-588-S12.ZIP › T7204/T7204 chromosome 23.png]

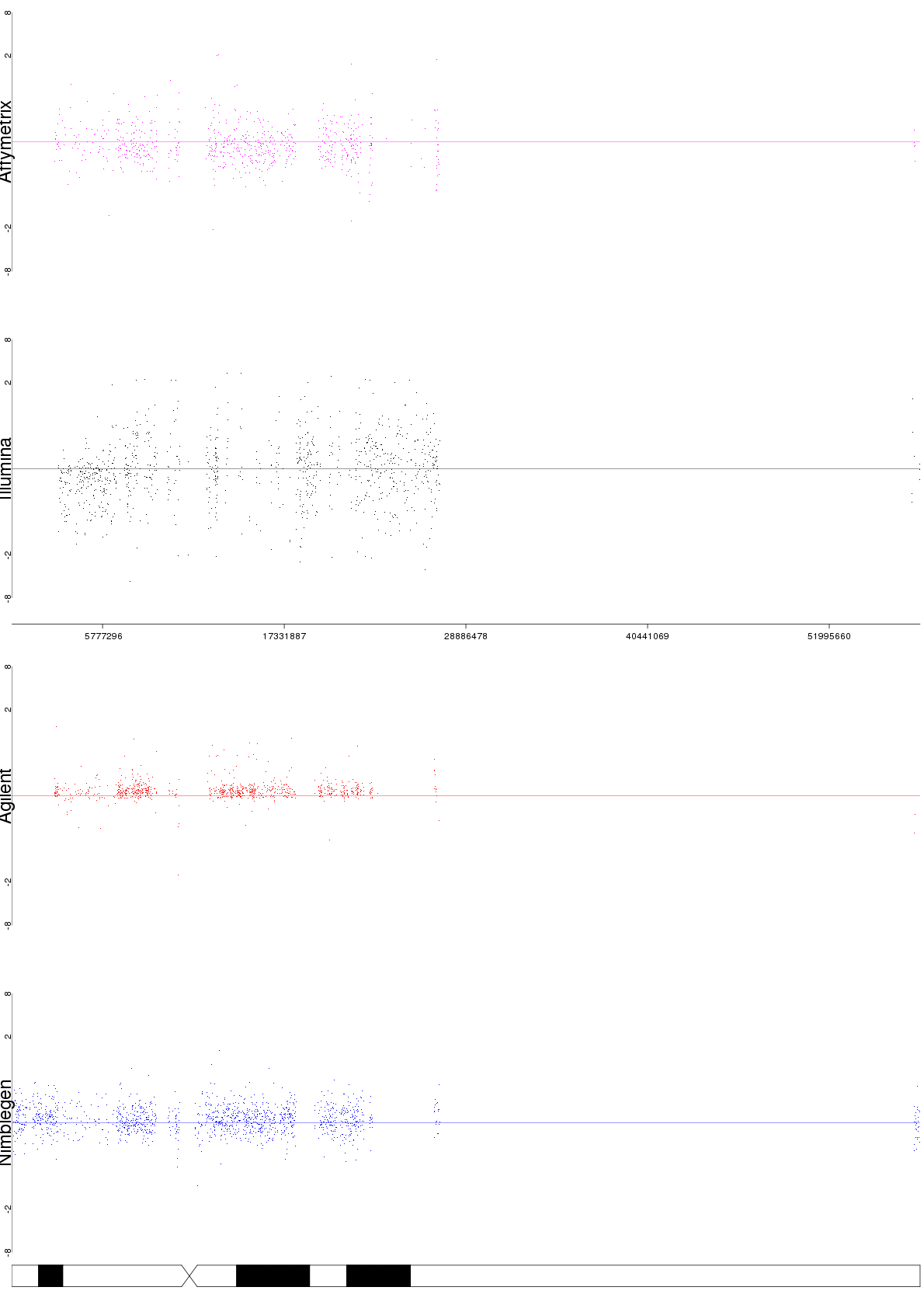

Supplement: Additional file 12 — All sample/chromosome plots for the tumours. Zip folder containing PNGs of all whole-chromosome plots for the tumours. [file 1471-2164-10-588-S12.ZIP › T7204/T7204 chromosome 24.png]

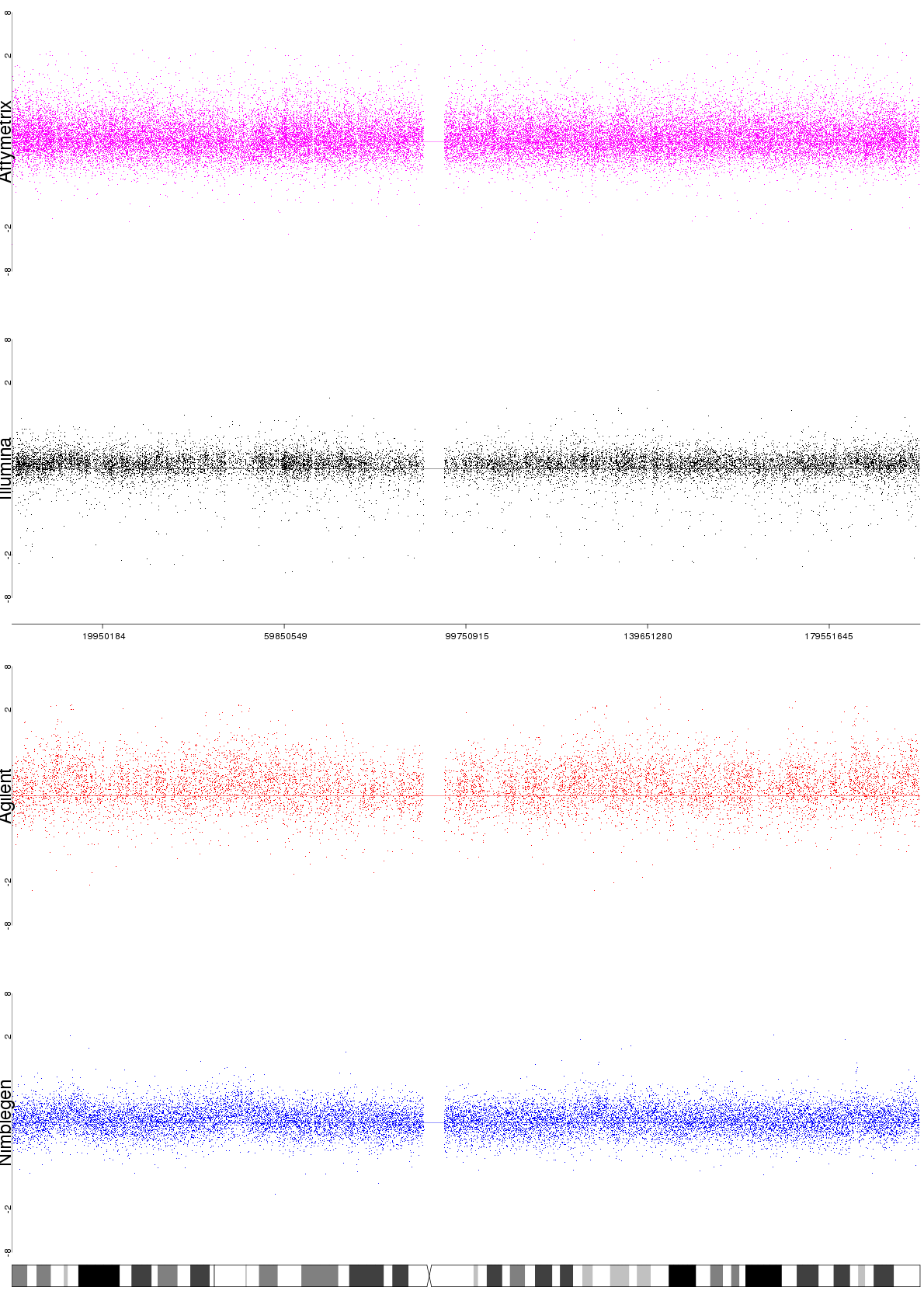

Supplement: Additional file 12 — All sample/chromosome plots for the tumours. Zip folder containing PNGs of all whole-chromosome plots for the tumours. [file 1471-2164-10-588-S12.ZIP › T7204/T7204 chromosome 3.png]

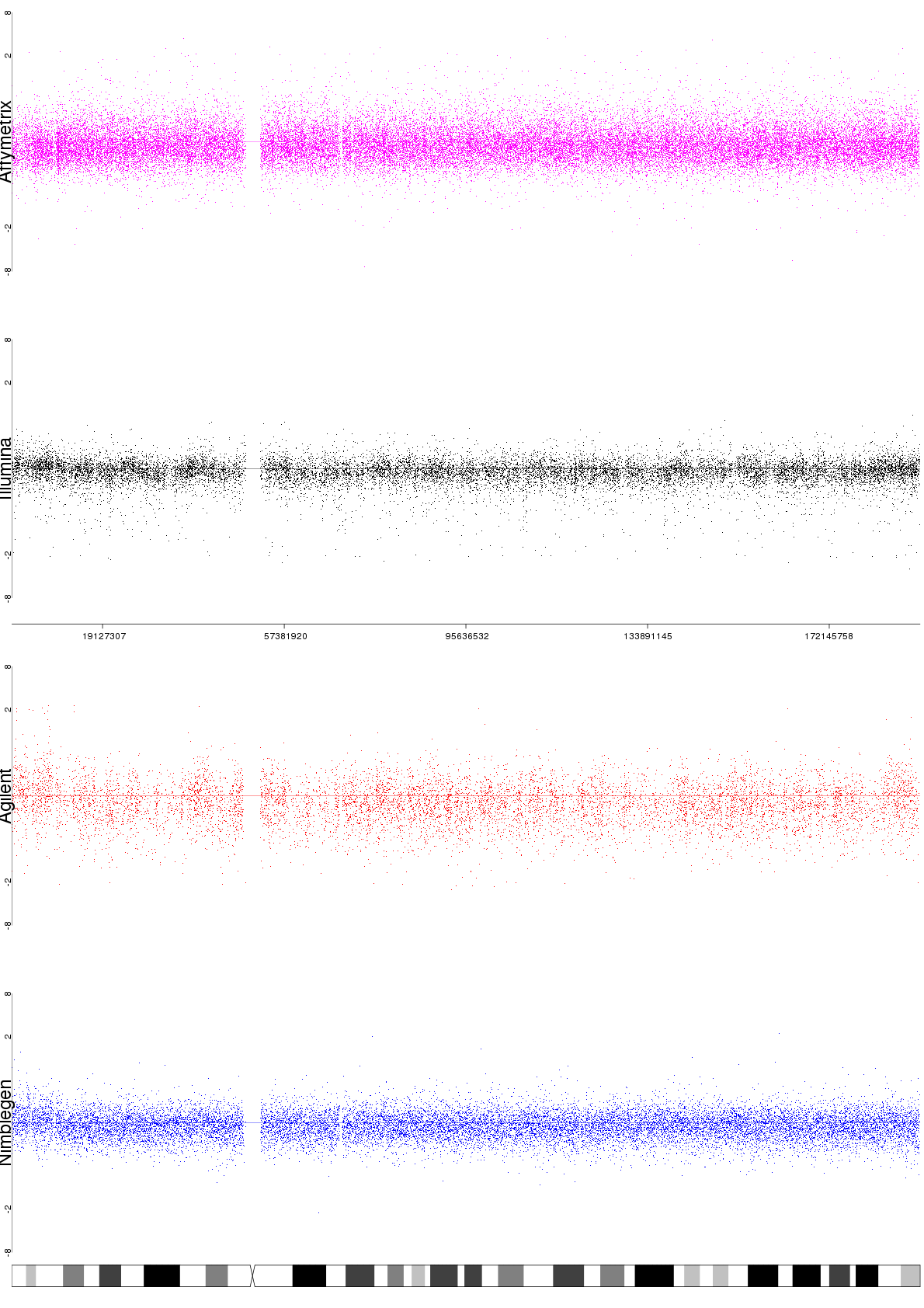

Supplement: Additional file 12 — All sample/chromosome plots for the tumours. Zip folder containing PNGs of all whole-chromosome plots for the tumours. [file 1471-2164-10-588-S12.ZIP › T7204/T7204 chromosome 4.png]

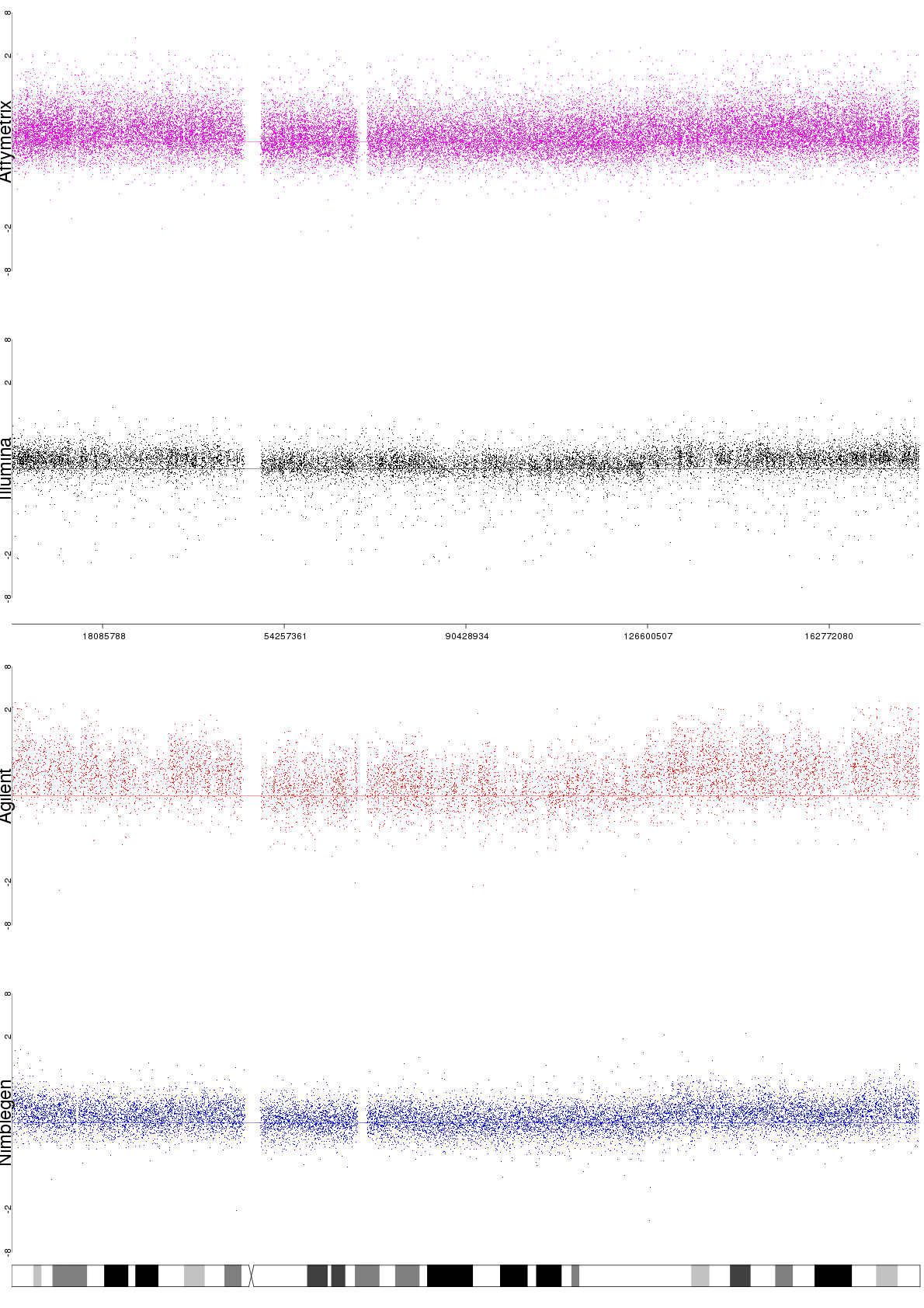

Supplement: Additional file 12 — All sample/chromosome plots for the tumours. Zip folder containing PNGs of all whole-chromosome plots for the tumours. [file 1471-2164-10-588-S12.ZIP › T7204/T7204 chromosome 5.png]

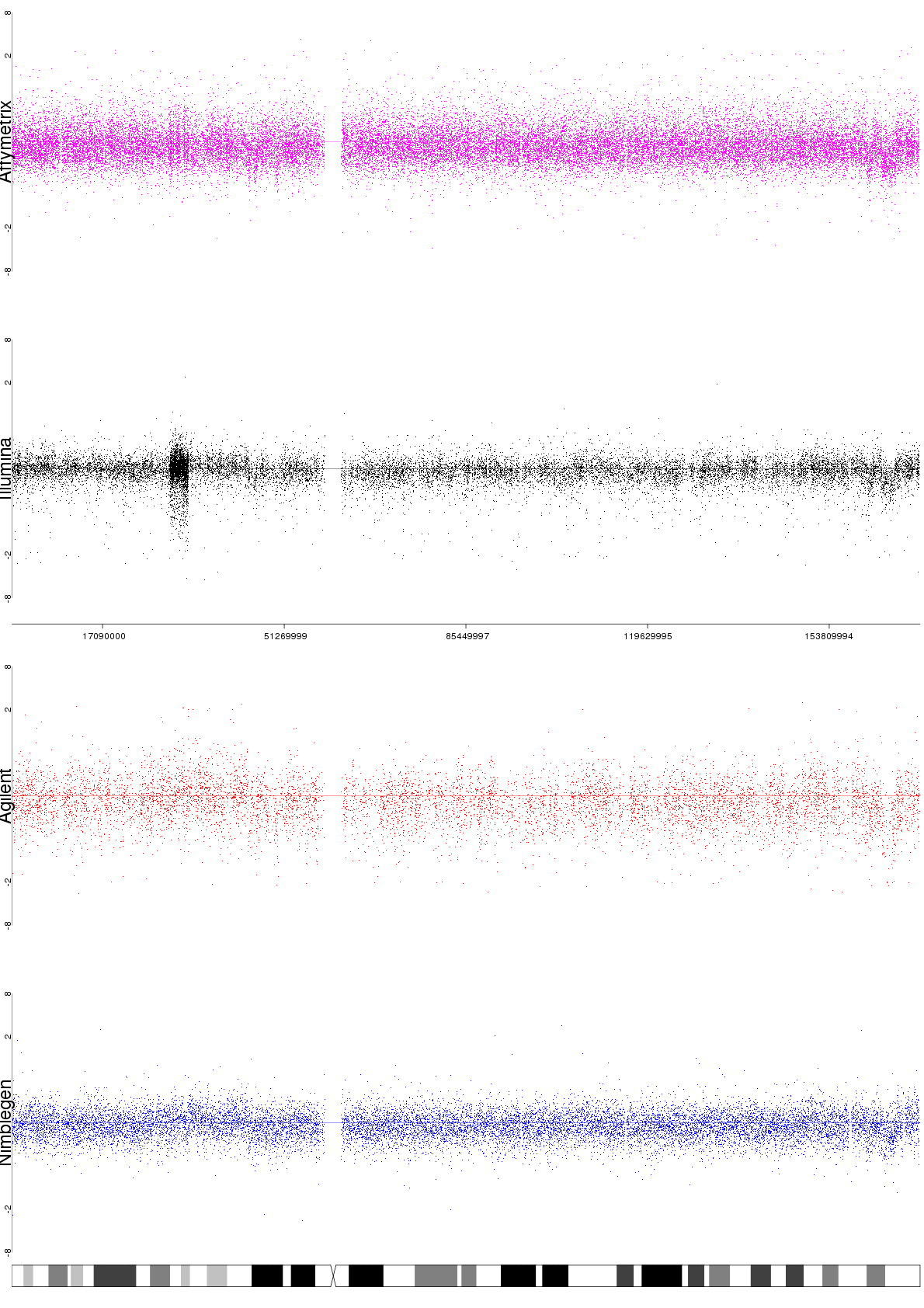

Supplement: Additional file 12 — All sample/chromosome plots for the tumours. Zip folder containing PNGs of all whole-chromosome plots for the tumours. [file 1471-2164-10-588-S12.ZIP › T7204/T7204 chromosome 6.png]

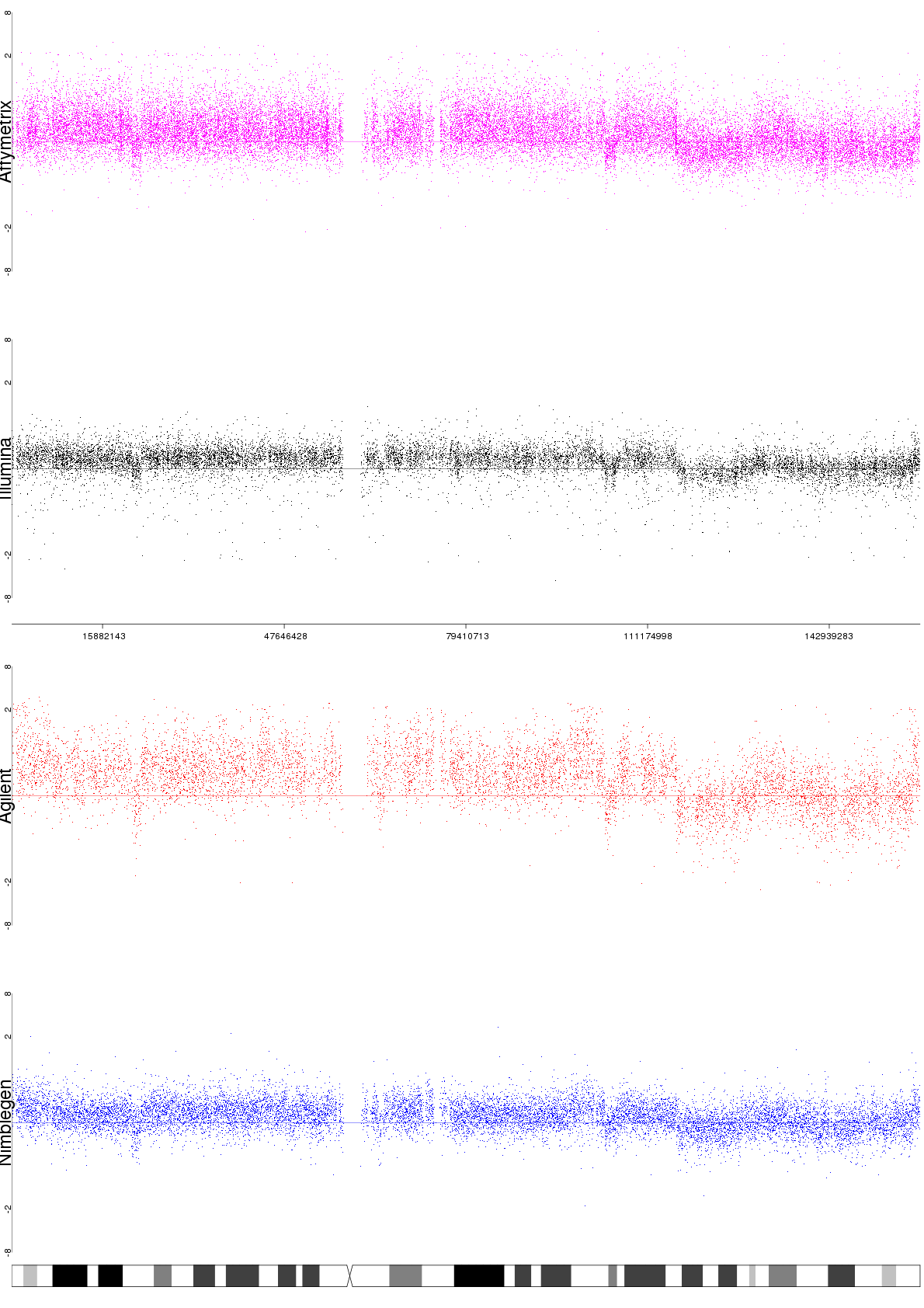

Supplement: Additional file 12 — All sample/chromosome plots for the tumours. Zip folder containing PNGs of all whole-chromosome plots for the tumours. [file 1471-2164-10-588-S12.ZIP › T7204/T7204 chromosome 7.png]

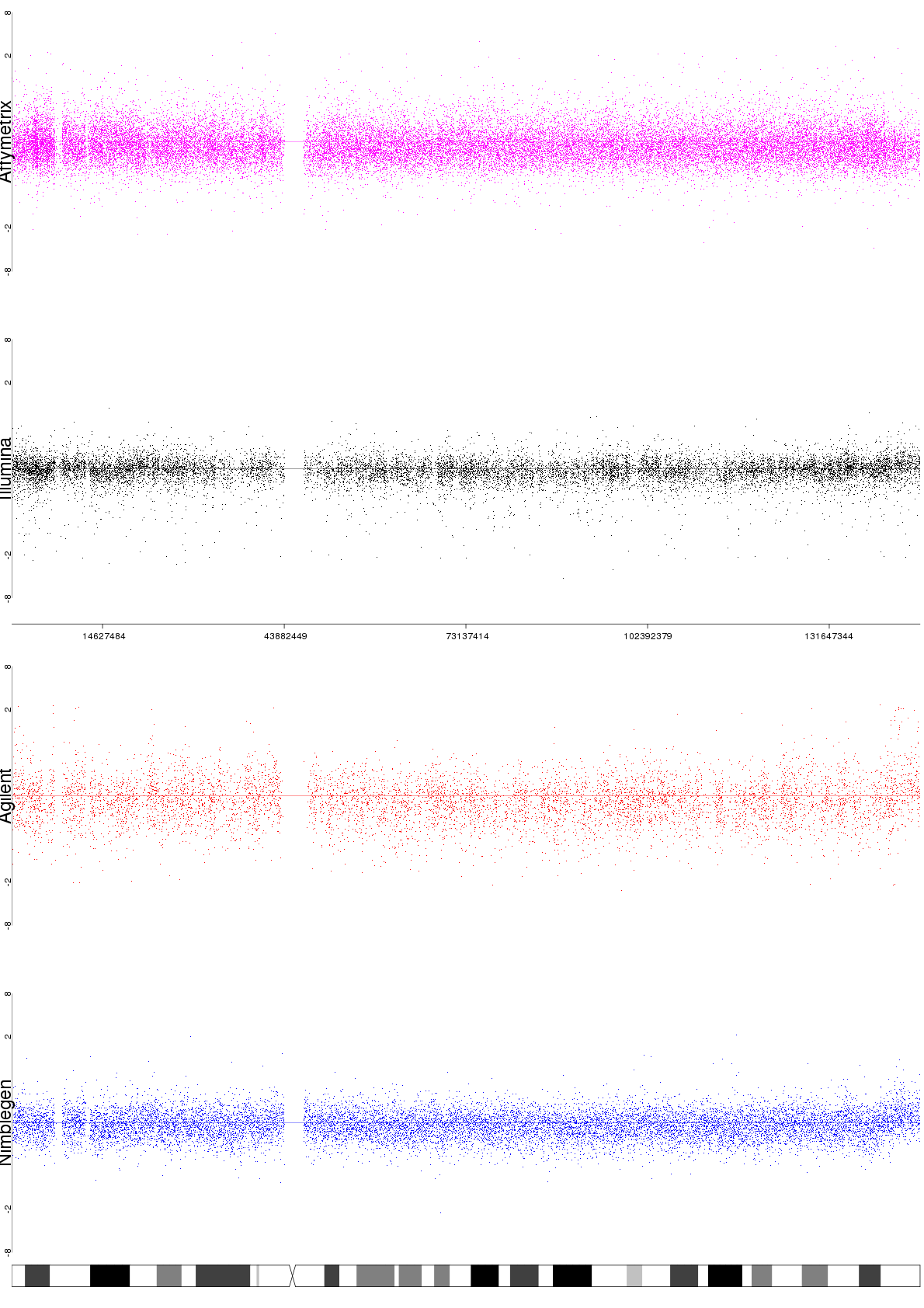

Supplement: Additional file 12 — All sample/chromosome plots for the tumours. Zip folder containing PNGs of all whole-chromosome plots for the tumours. [file 1471-2164-10-588-S12.ZIP › T7204/T7204 chromosome 8.png]

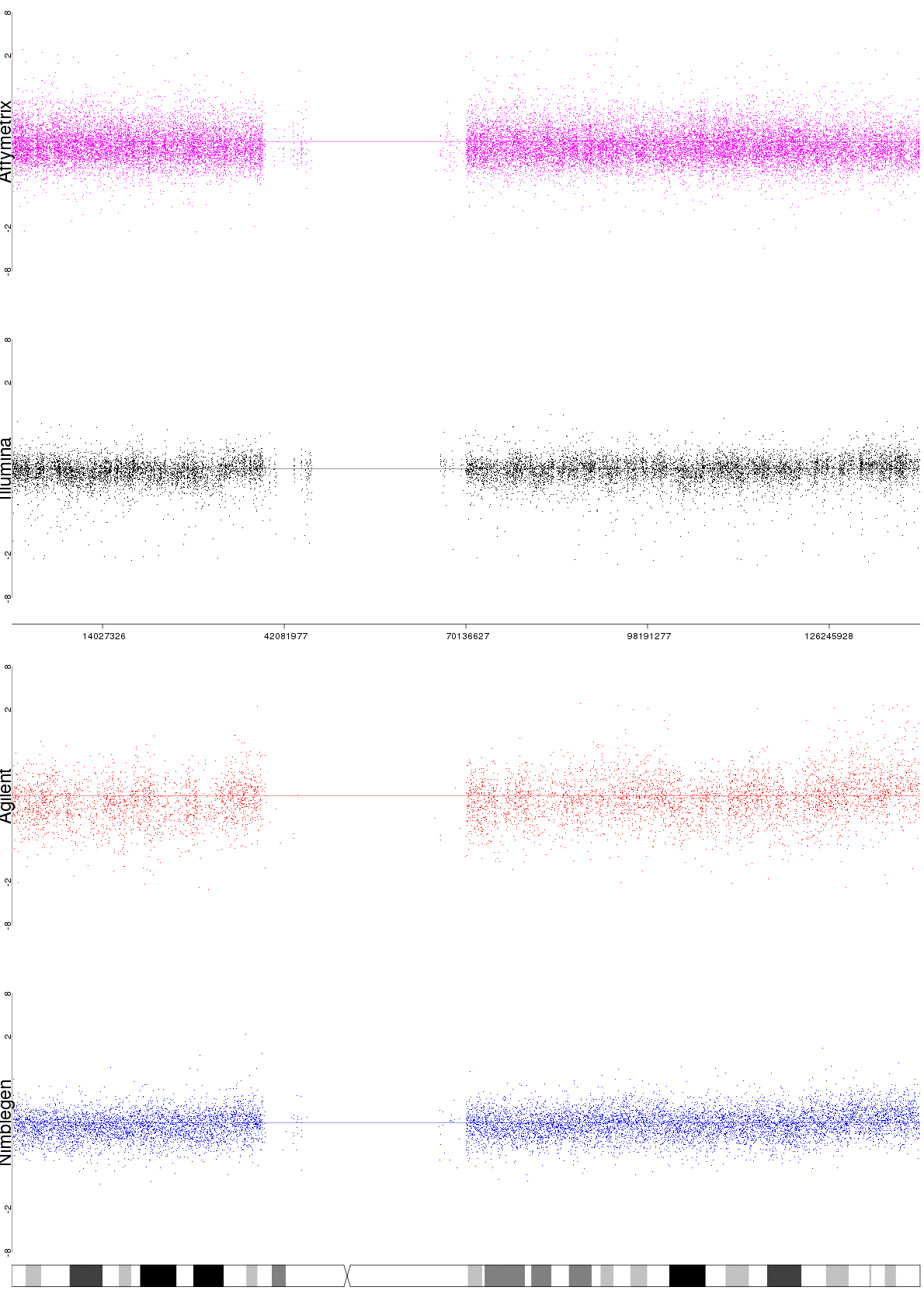

Supplement: Additional file 12 — All sample/chromosome plots for the tumours. Zip folder containing PNGs of all whole-chromosome plots for the tumours. [file 1471-2164-10-588-S12.ZIP › T7204/T7204 chromosome 9.png]

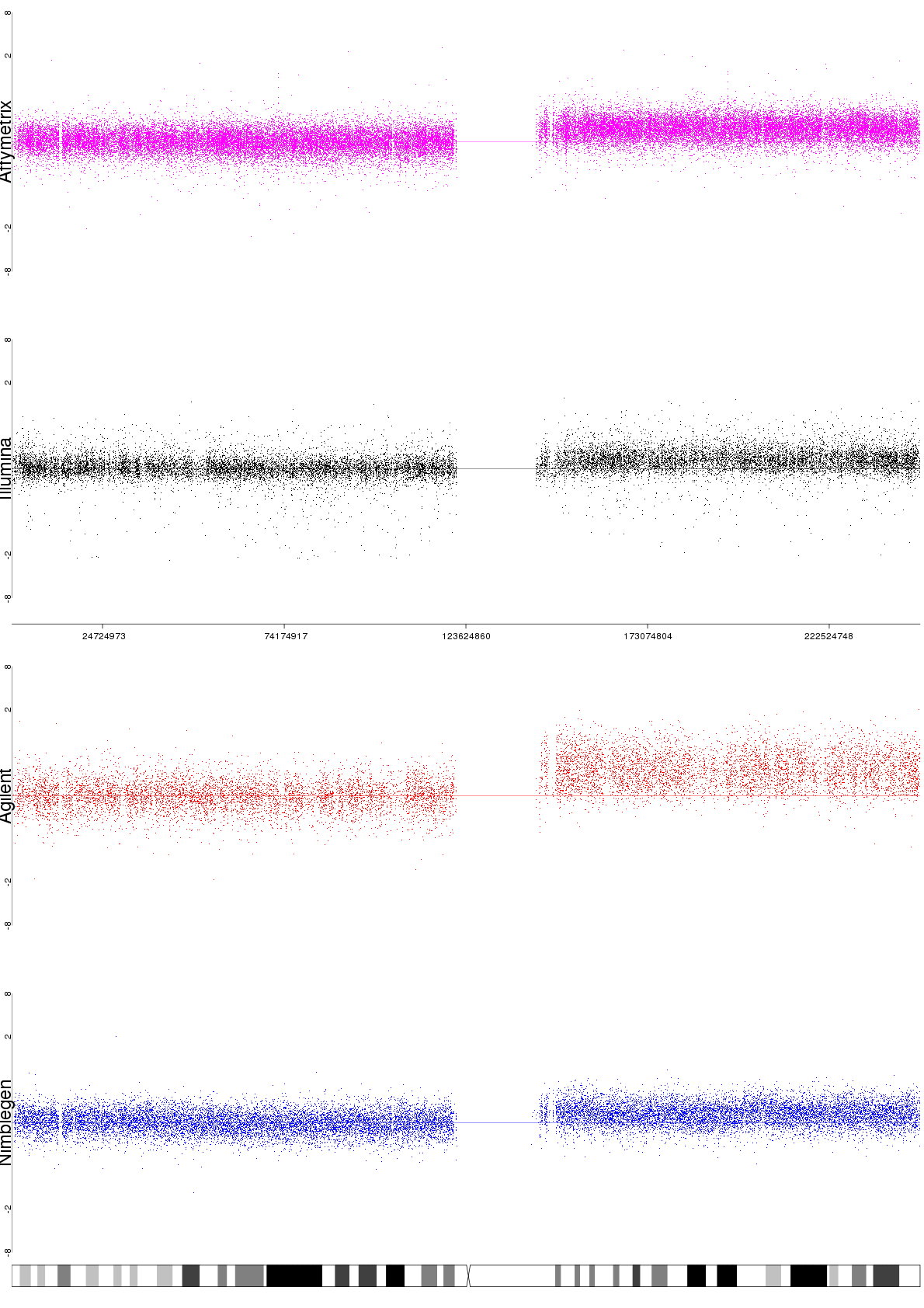

Supplement: Additional file 12 — All sample/chromosome plots for the tumours. Zip folder containing PNGs of all whole-chromosome plots for the tumours. [file 1471-2164-10-588-S12.ZIP › T7206/T7206 chromosome 1.png]

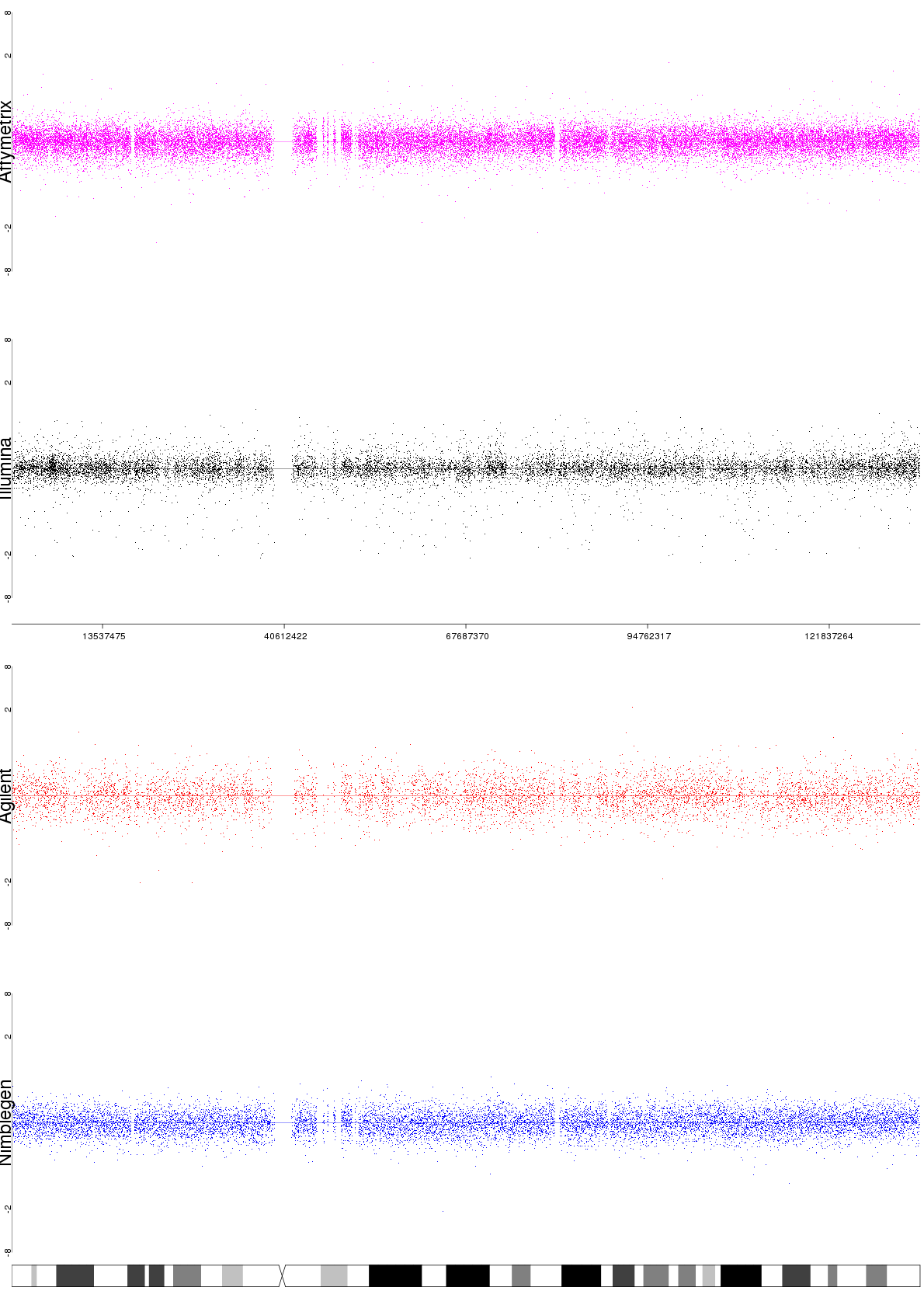

Supplement: Additional file 12 — All sample/chromosome plots for the tumours. Zip folder containing PNGs of all whole-chromosome plots for the tumours. [file 1471-2164-10-588-S12.ZIP › T7206/T7206 chromosome 10.png]

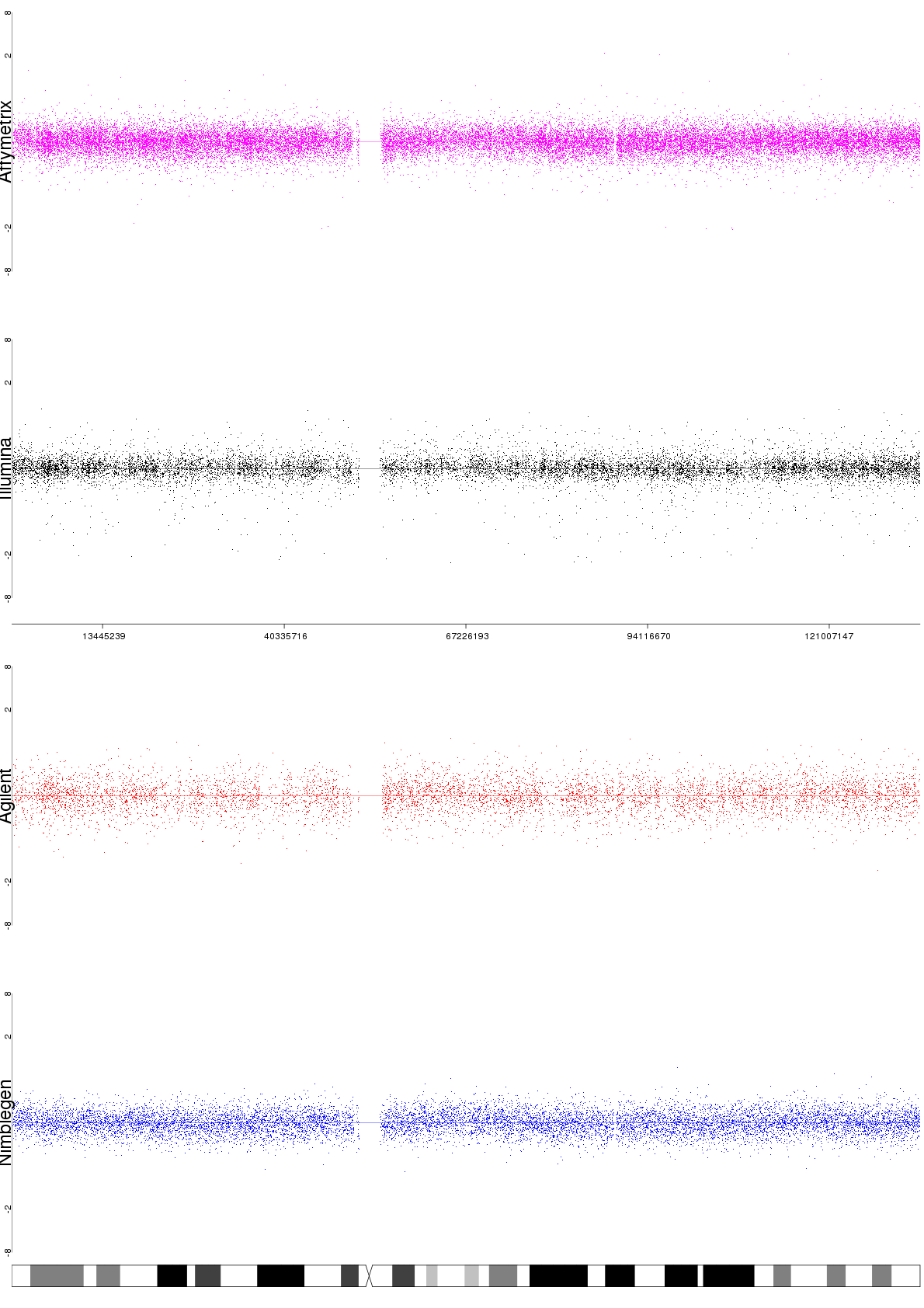

Supplement: Additional file 12 — All sample/chromosome plots for the tumours. Zip folder containing PNGs of all whole-chromosome plots for the tumours. [file 1471-2164-10-588-S12.ZIP › T7206/T7206 chromosome 11.png]

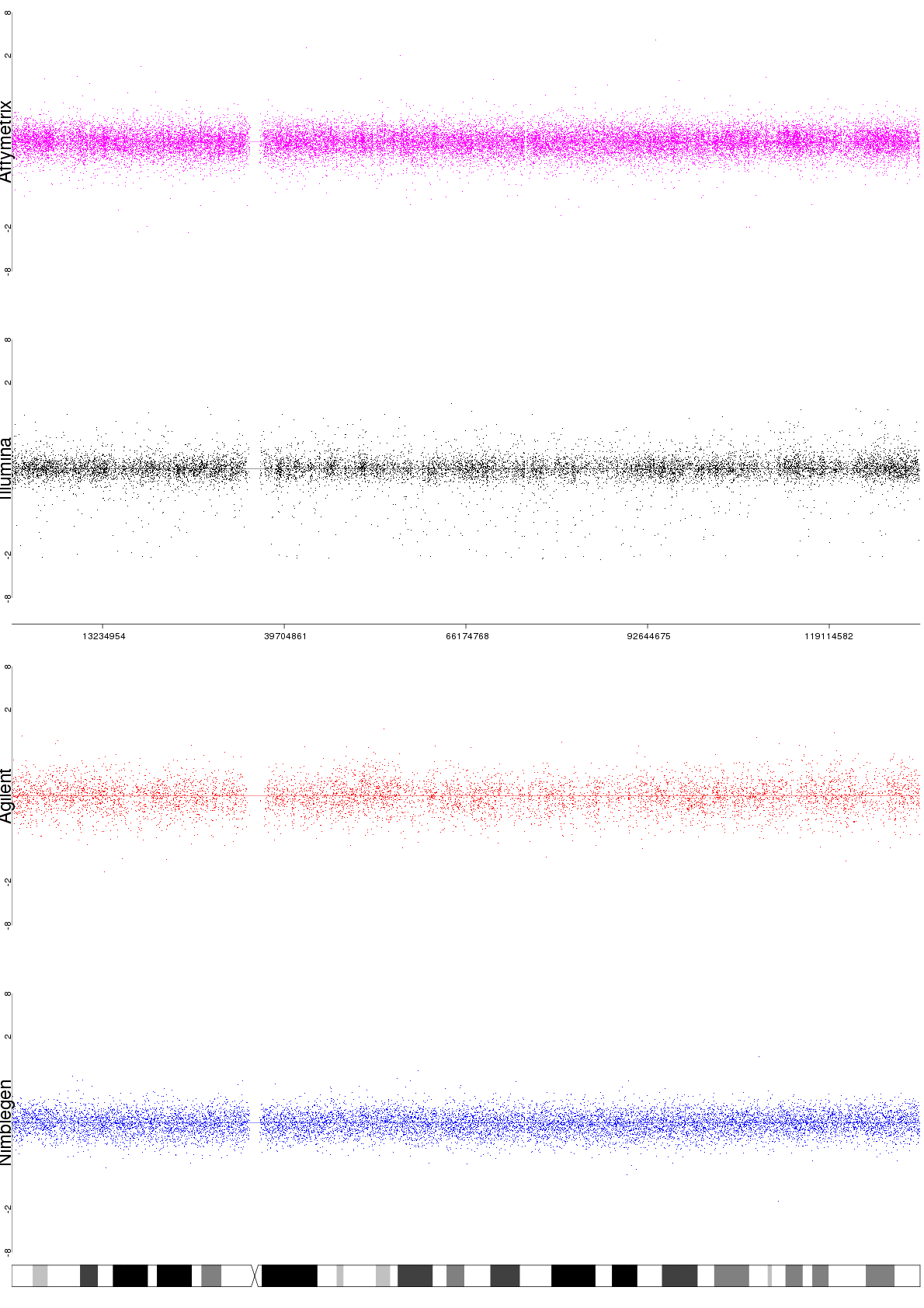

Supplement: Additional file 12 — All sample/chromosome plots for the tumours. Zip folder containing PNGs of all whole-chromosome plots for the tumours. [file 1471-2164-10-588-S12.ZIP › T7206/T7206 chromosome 12.png]

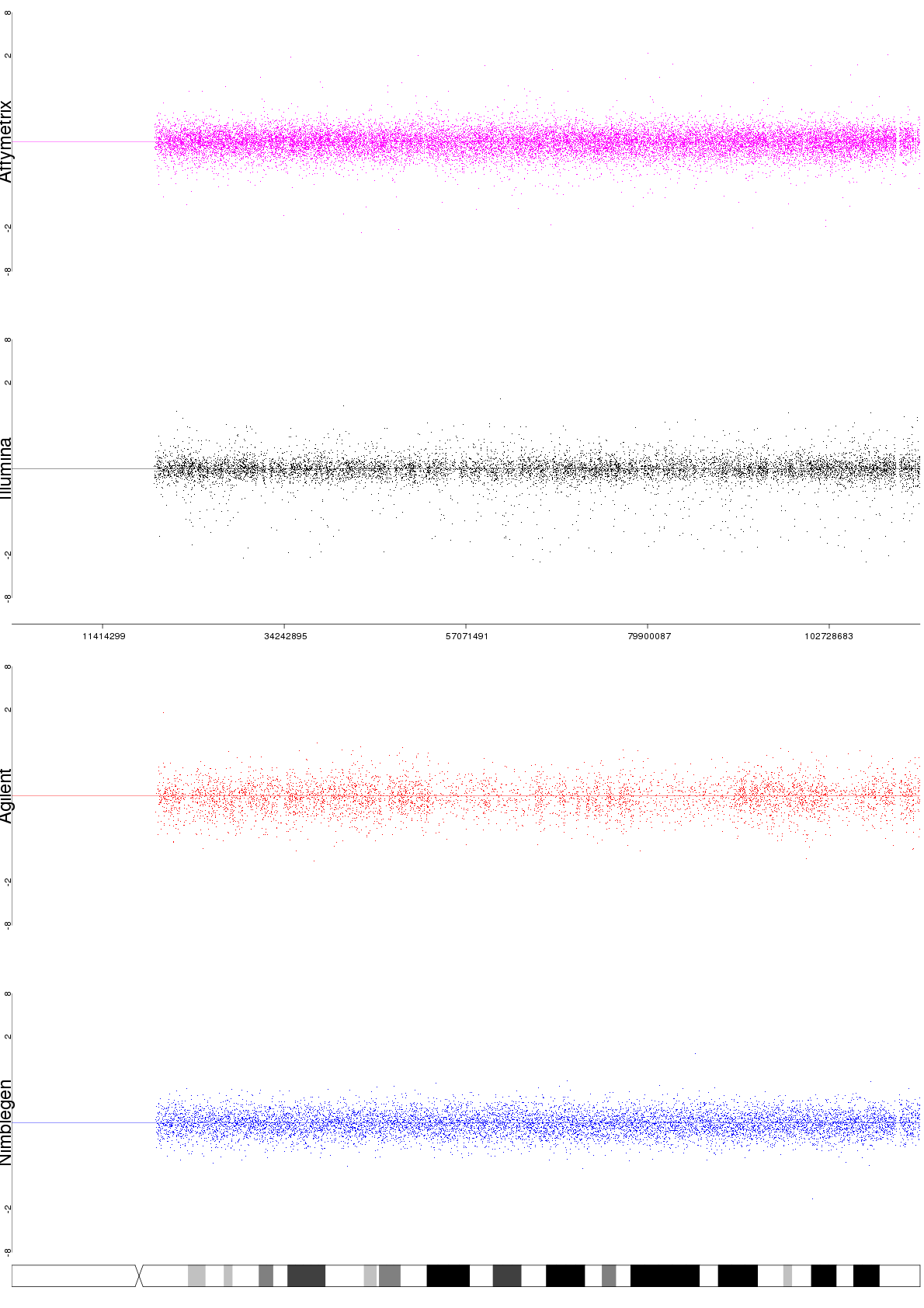

Supplement: Additional file 12 — All sample/chromosome plots for the tumours. Zip folder containing PNGs of all whole-chromosome plots for the tumours. [file 1471-2164-10-588-S12.ZIP › T7206/T7206 chromosome 13.png]

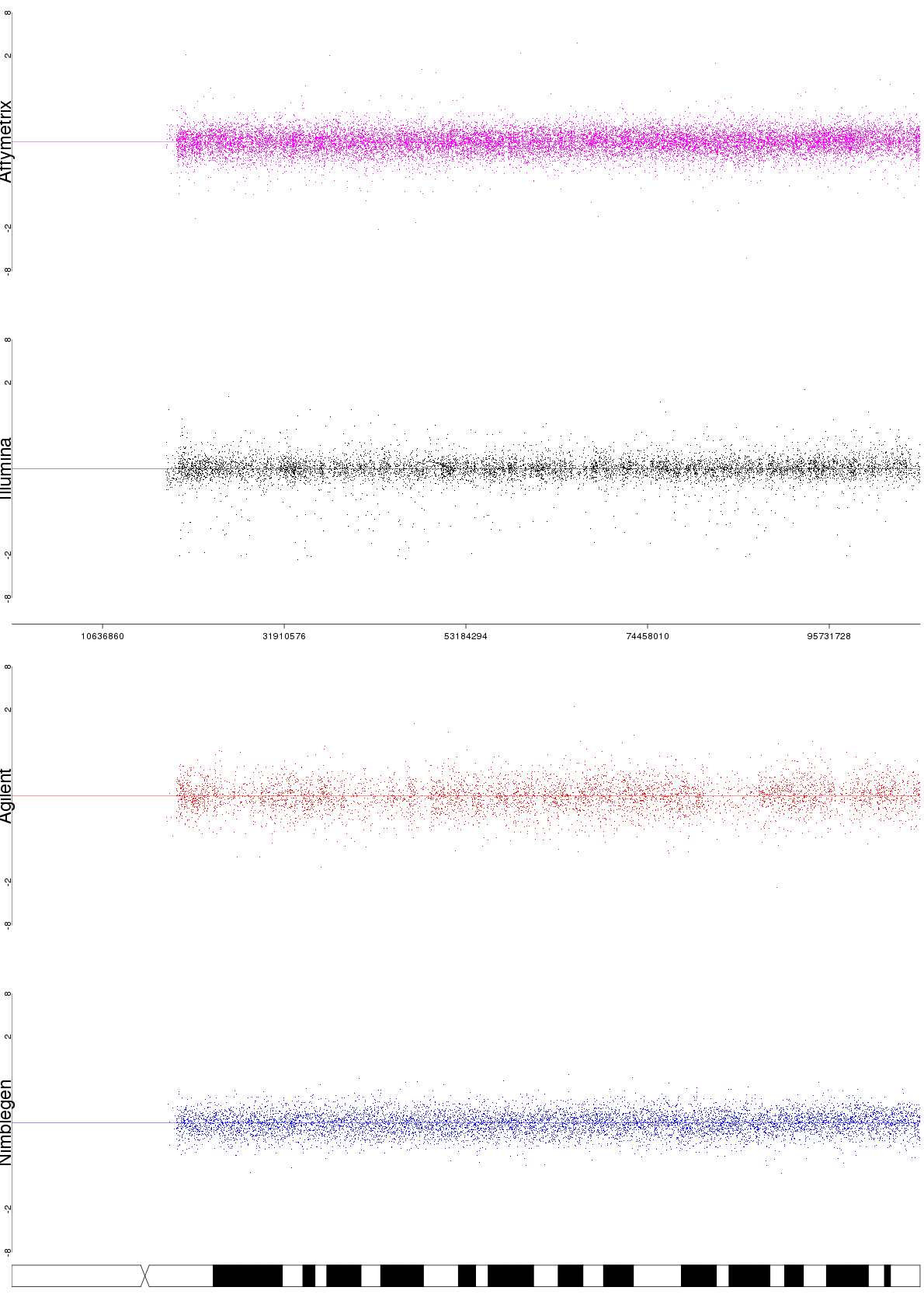

Supplement: Additional file 12 — All sample/chromosome plots for the tumours. Zip folder containing PNGs of all whole-chromosome plots for the tumours. [file 1471-2164-10-588-S12.ZIP › T7206/T7206 chromosome 14.png]

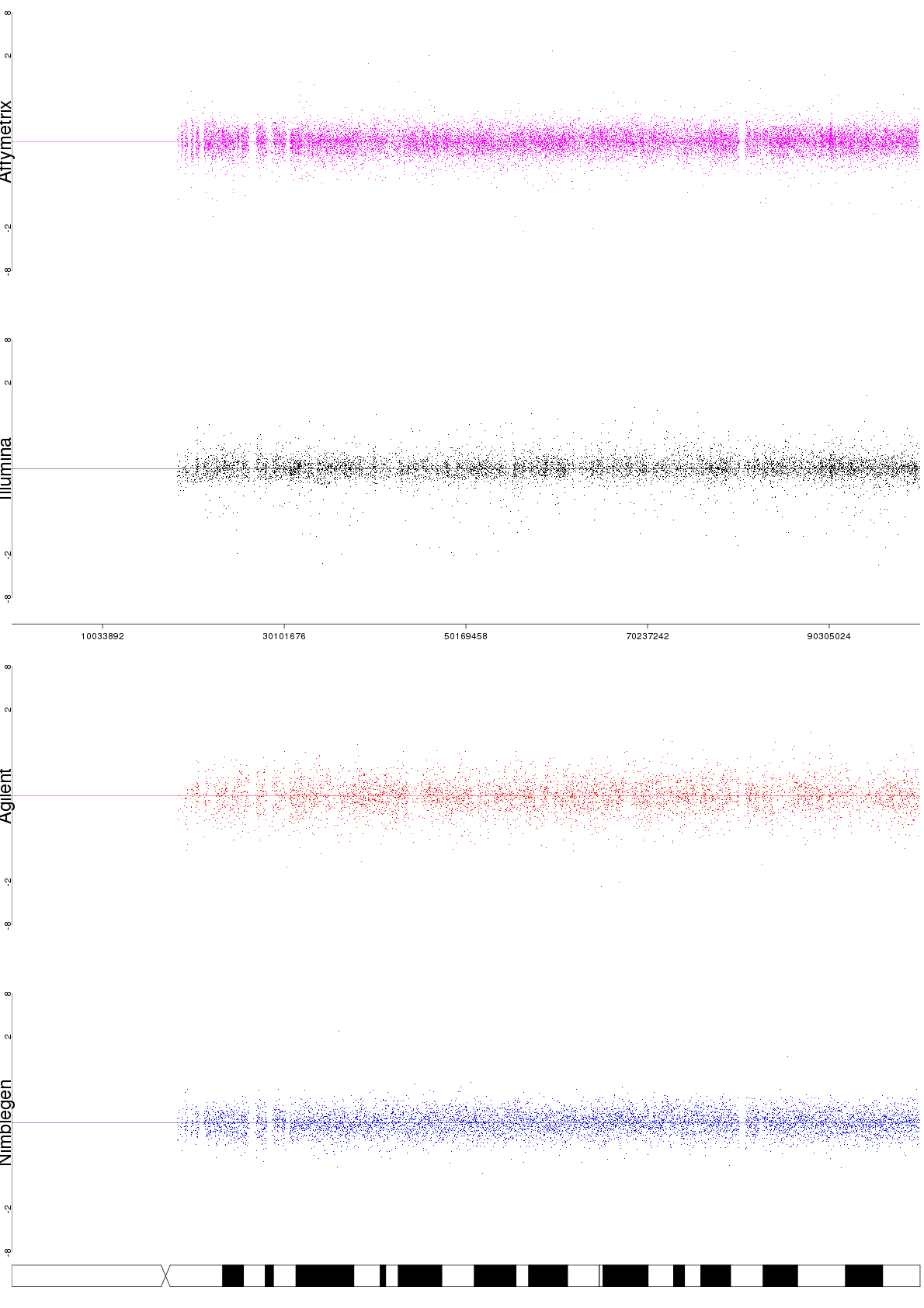

Supplement: Additional file 12 — All sample/chromosome plots for the tumours. Zip folder containing PNGs of all whole-chromosome plots for the tumours. [file 1471-2164-10-588-S12.ZIP › T7206/T7206 chromosome 15.png]

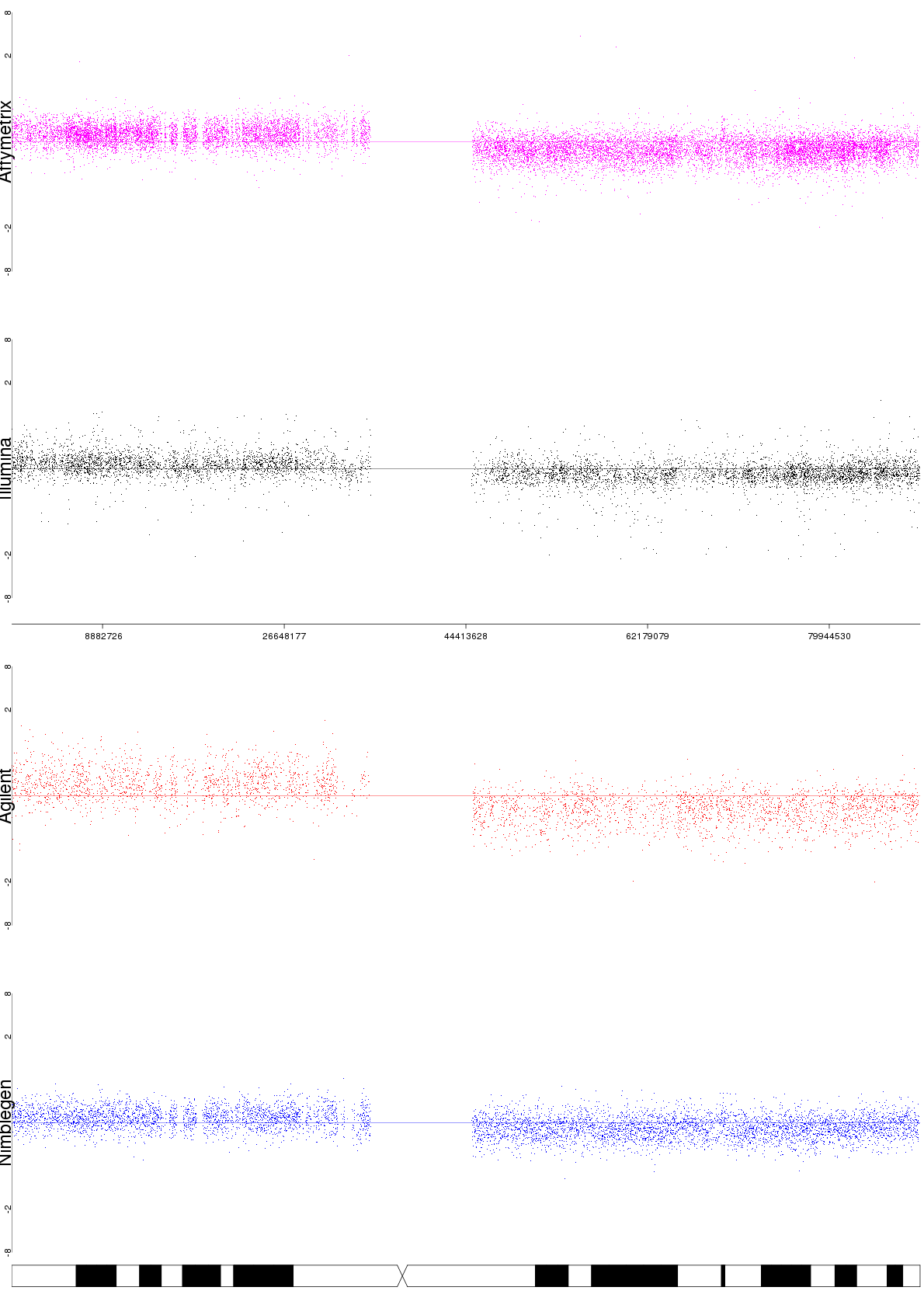

Supplement: Additional file 12 — All sample/chromosome plots for the tumours. Zip folder containing PNGs of all whole-chromosome plots for the tumours. [file 1471-2164-10-588-S12.ZIP › T7206/T7206 chromosome 16.png]

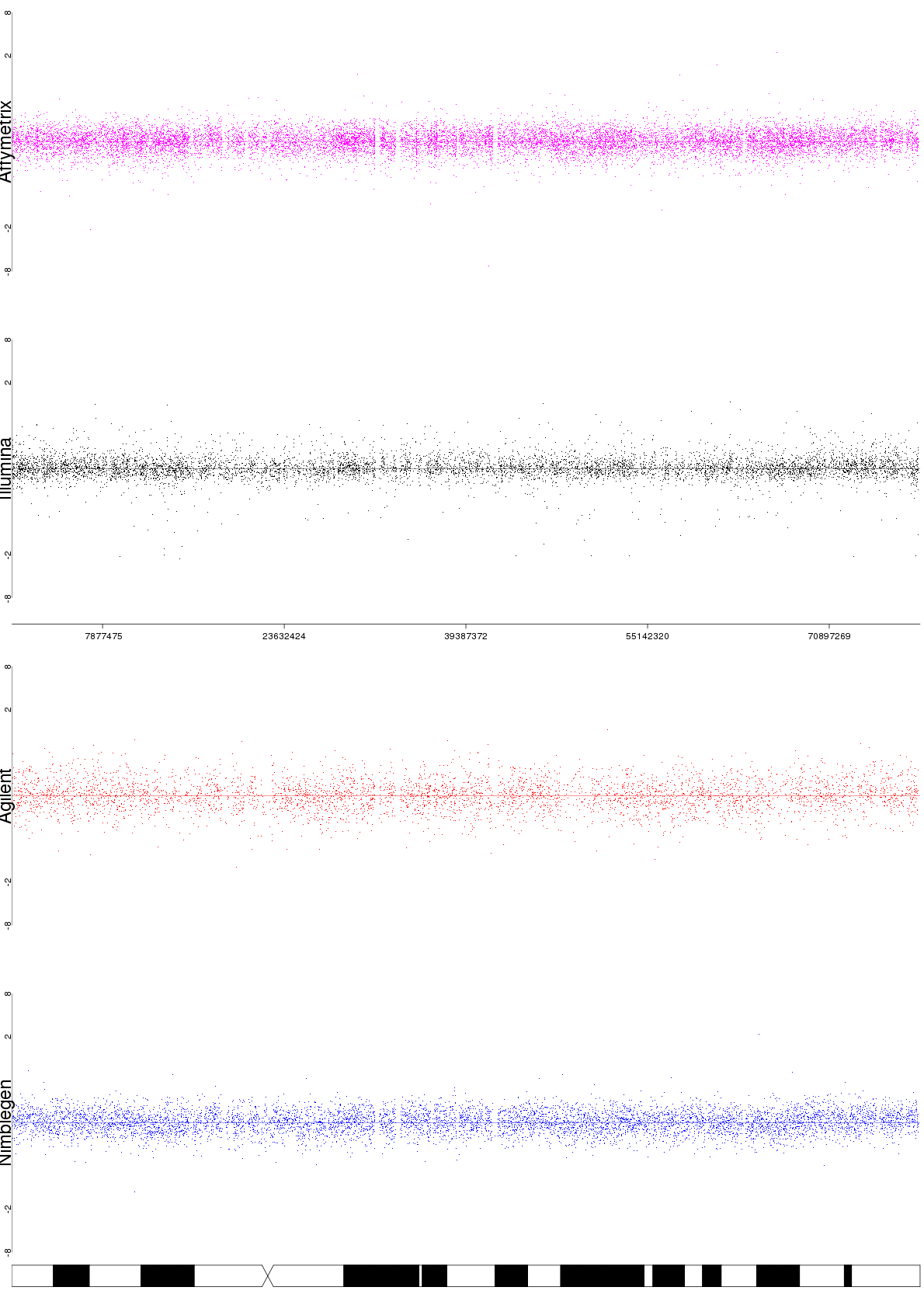

Supplement: Additional file 12 — All sample/chromosome plots for the tumours. Zip folder containing PNGs of all whole-chromosome plots for the tumours. [file 1471-2164-10-588-S12.ZIP › T7206/T7206 chromosome 17.png]

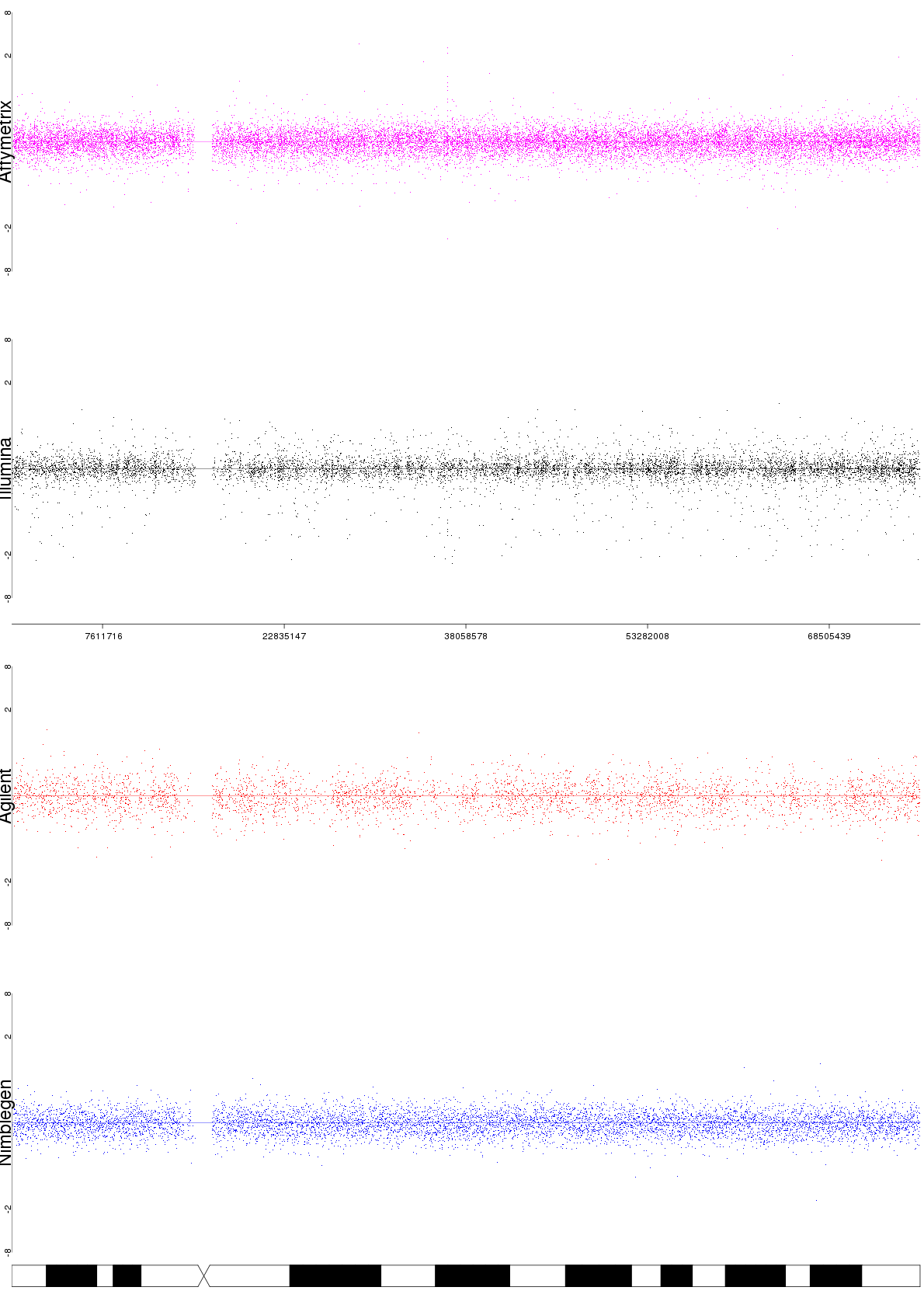

Supplement: Additional file 12 — All sample/chromosome plots for the tumours. Zip folder containing PNGs of all whole-chromosome plots for the tumours. [file 1471-2164-10-588-S12.ZIP › T7206/T7206 chromosome 18.png]

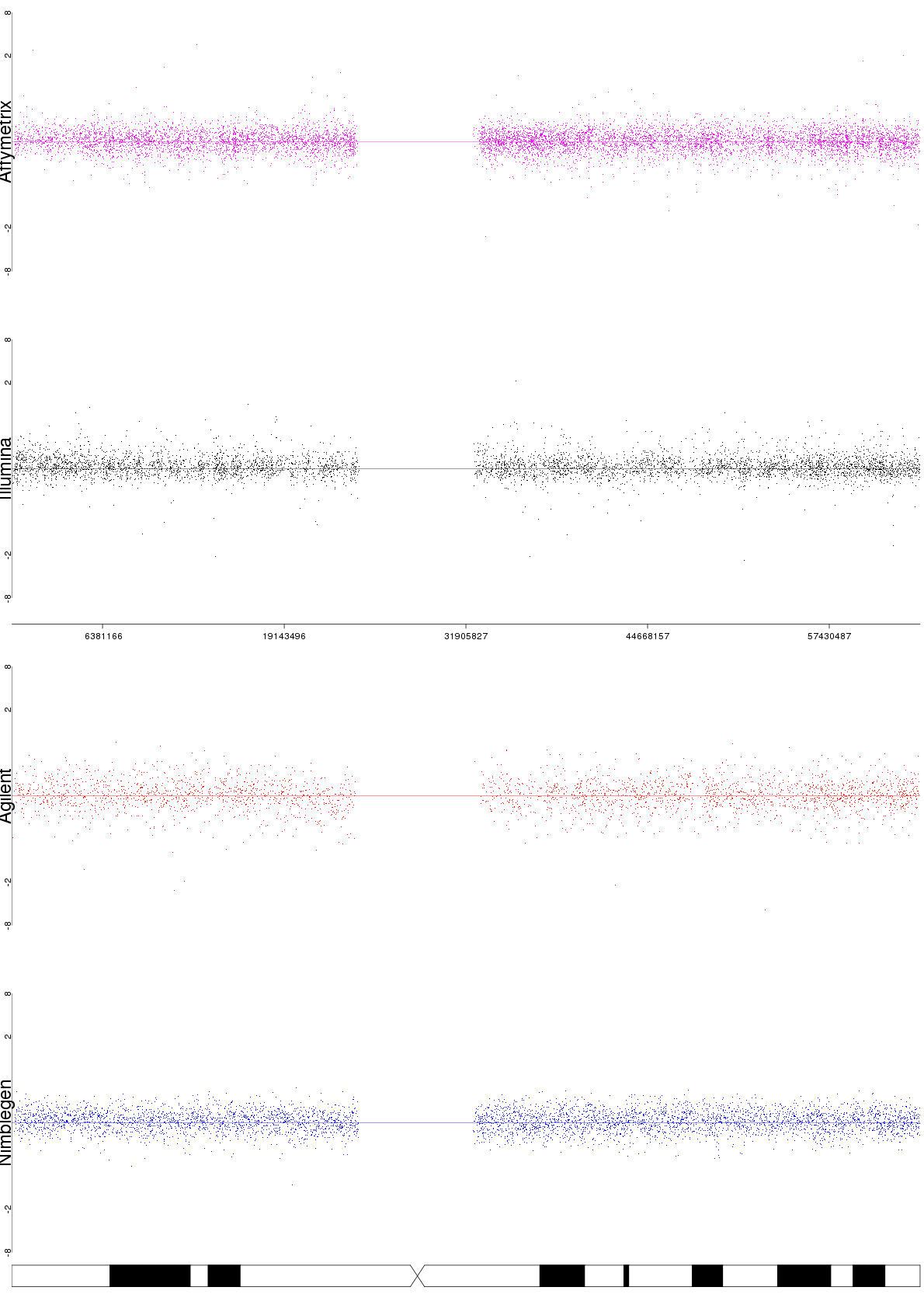

Supplement: Additional file 12 — All sample/chromosome plots for the tumours. Zip folder containing PNGs of all whole-chromosome plots for the tumours. [file 1471-2164-10-588-S12.ZIP › T7206/T7206 chromosome 19.png]

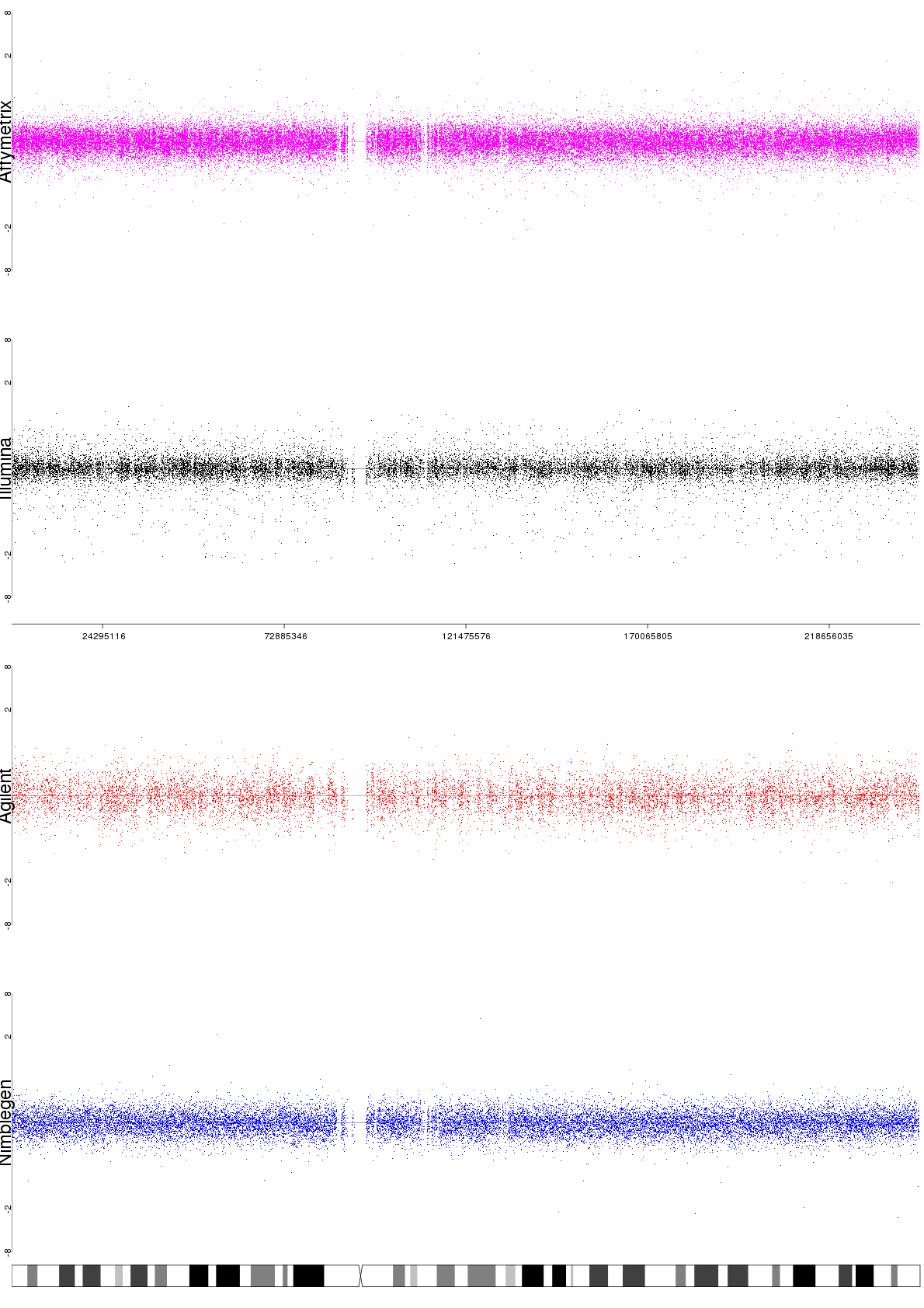

Supplement: Additional file 12 — All sample/chromosome plots for the tumours. Zip folder containing PNGs of all whole-chromosome plots for the tumours. [file 1471-2164-10-588-S12.ZIP › T7206/T7206 chromosome 2.png]

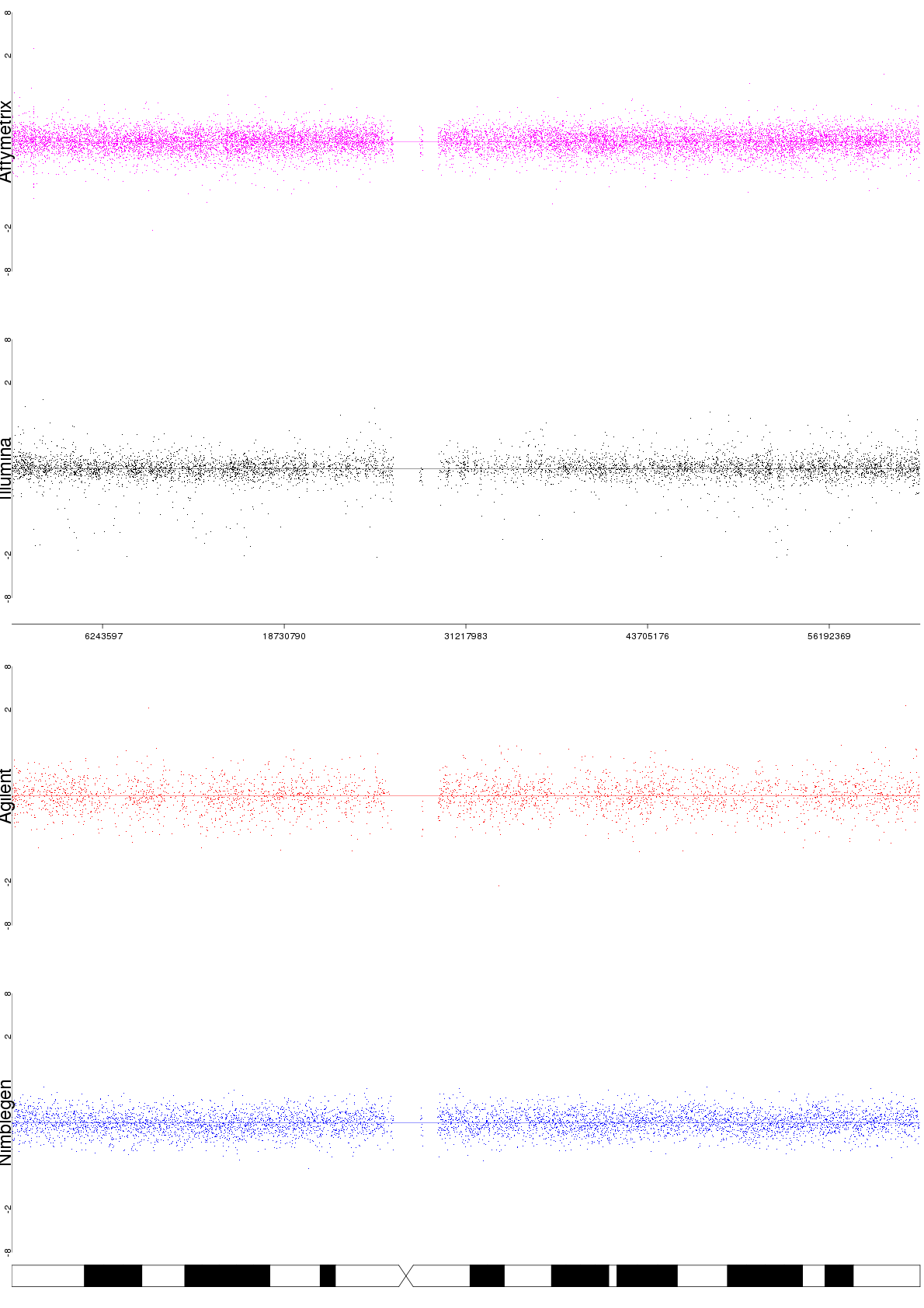

Supplement: Additional file 12 — All sample/chromosome plots for the tumours. Zip folder containing PNGs of all whole-chromosome plots for the tumours. [file 1471-2164-10-588-S12.ZIP › T7206/T7206 chromosome 20.png]

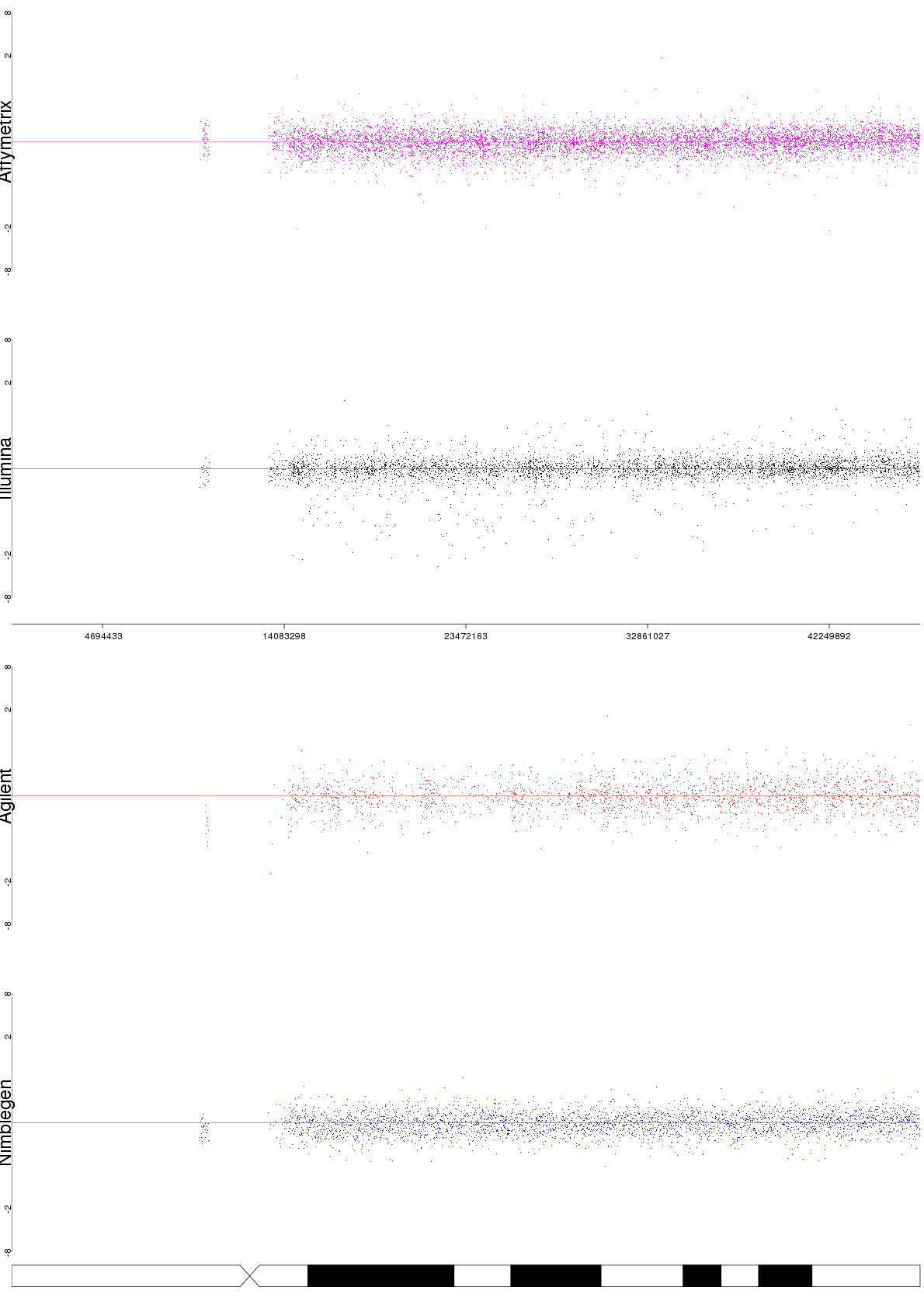

Supplement: Additional file 12 — All sample/chromosome plots for the tumours. Zip folder containing PNGs of all whole-chromosome plots for the tumours. [file 1471-2164-10-588-S12.ZIP › T7206/T7206 chromosome 21.png]

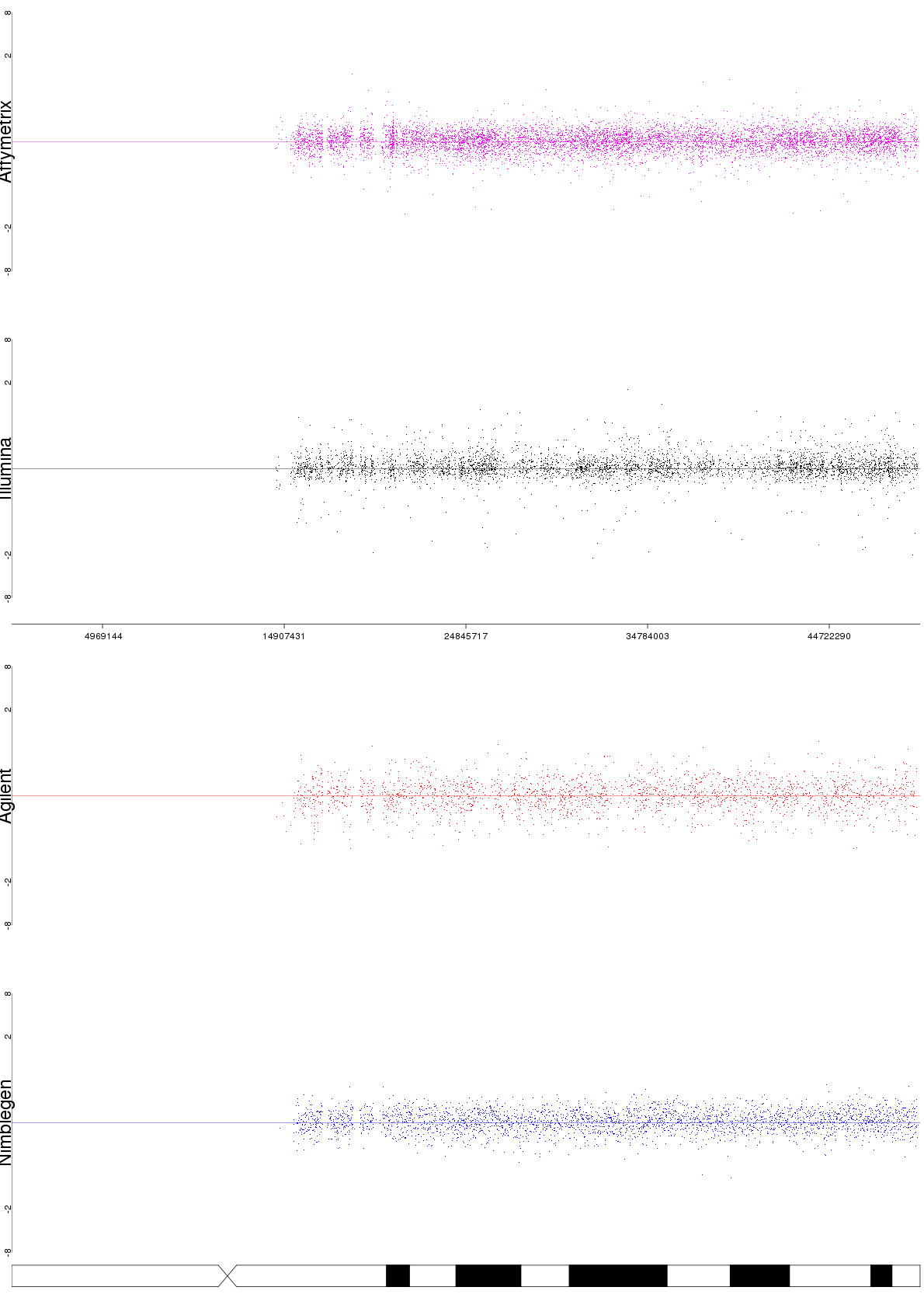

Supplement: Additional file 12 — All sample/chromosome plots for the tumours. Zip folder containing PNGs of all whole-chromosome plots for the tumours. [file 1471-2164-10-588-S12.ZIP › T7206/T7206 chromosome 22.png]

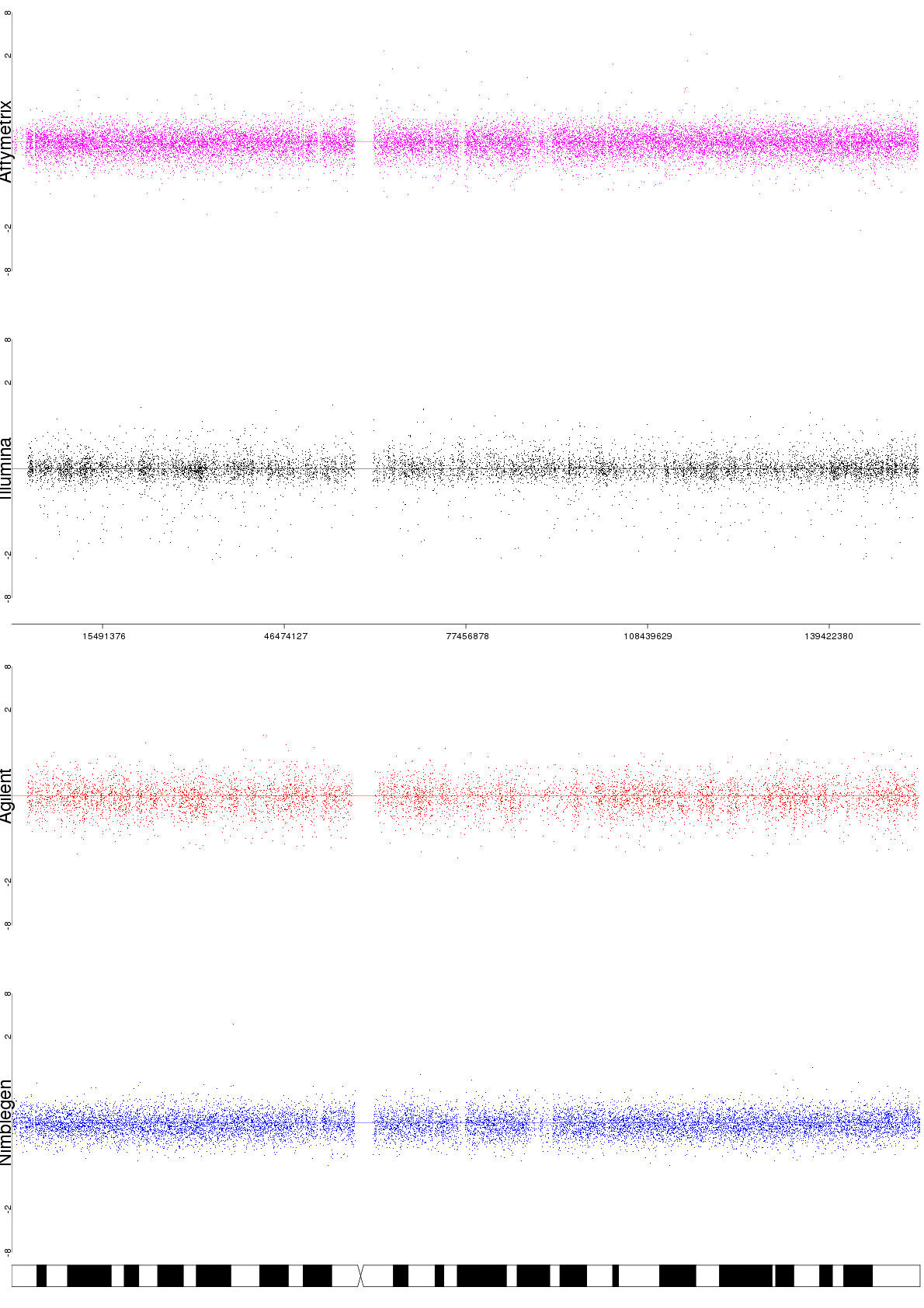

Supplement: Additional file 12 — All sample/chromosome plots for the tumours. Zip folder containing PNGs of all whole-chromosome plots for the tumours. [file 1471-2164-10-588-S12.ZIP › T7206/T7206 chromosome 23.png]

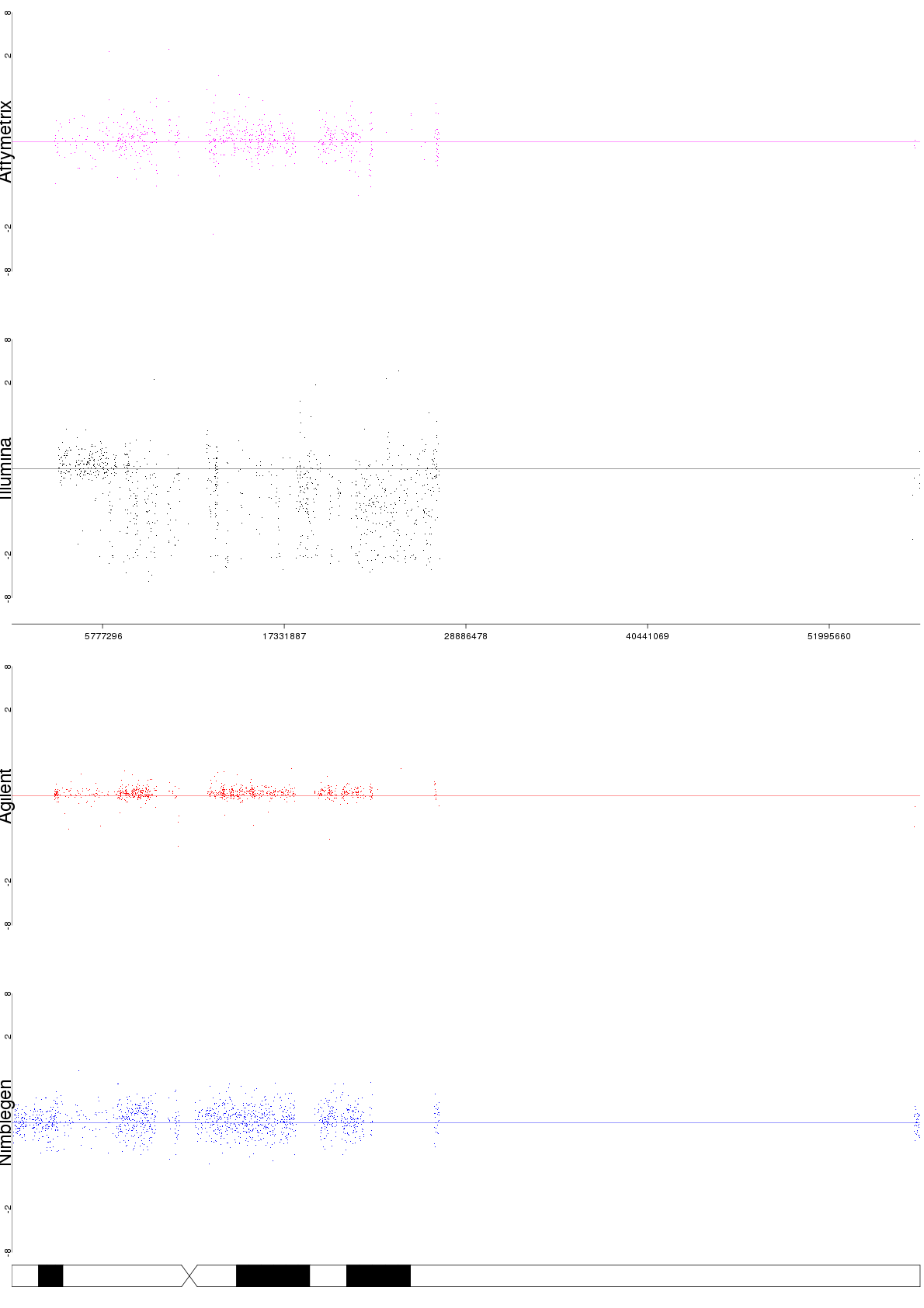

Supplement: Additional file 12 — All sample/chromosome plots for the tumours. Zip folder containing PNGs of all whole-chromosome plots for the tumours. [file 1471-2164-10-588-S12.ZIP › T7206/T7206 chromosome 24.png]

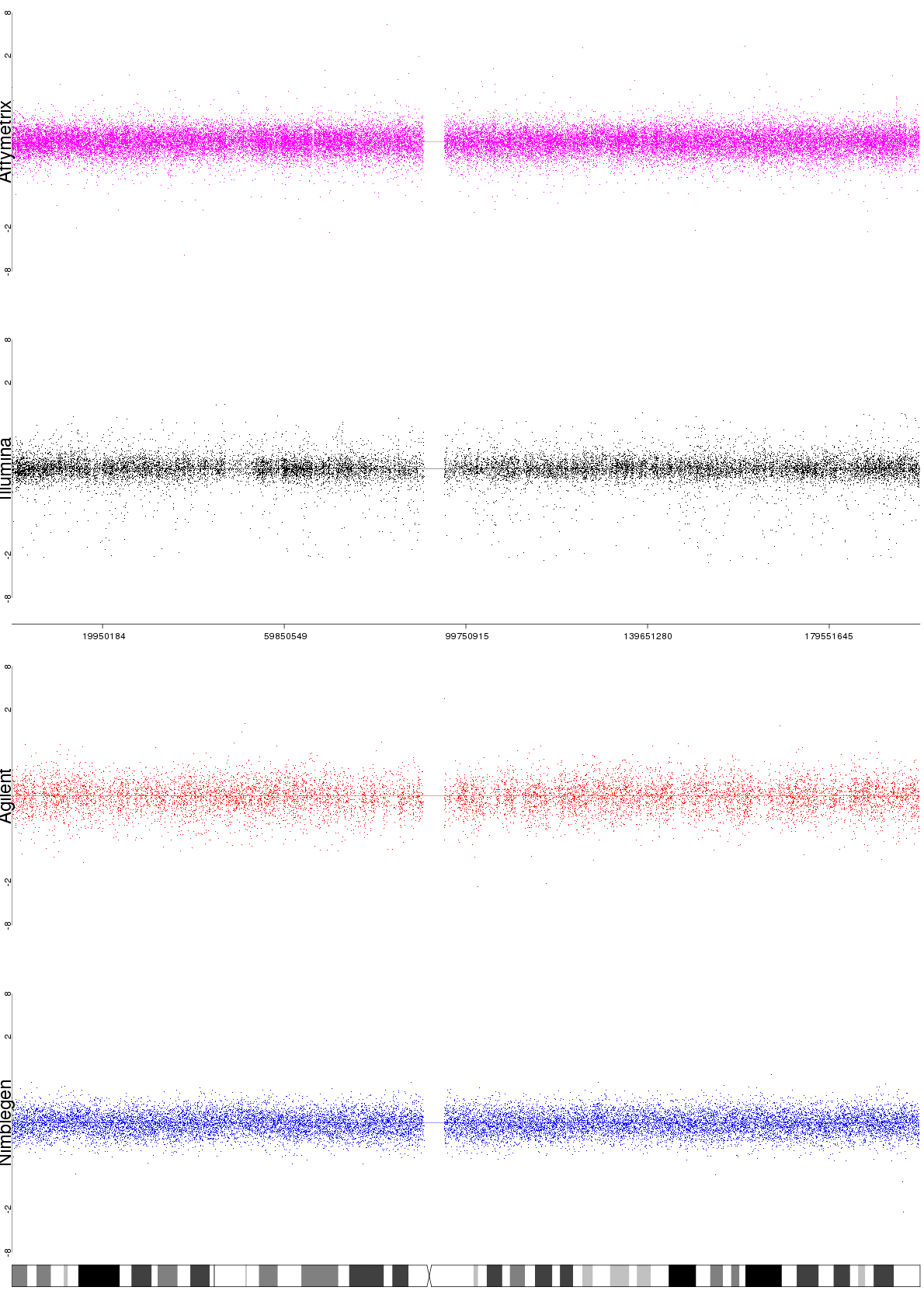

Supplement: Additional file 12 — All sample/chromosome plots for the tumours. Zip folder containing PNGs of all whole-chromosome plots for the tumours. [file 1471-2164-10-588-S12.ZIP › T7206/T7206 chromosome 3.png]

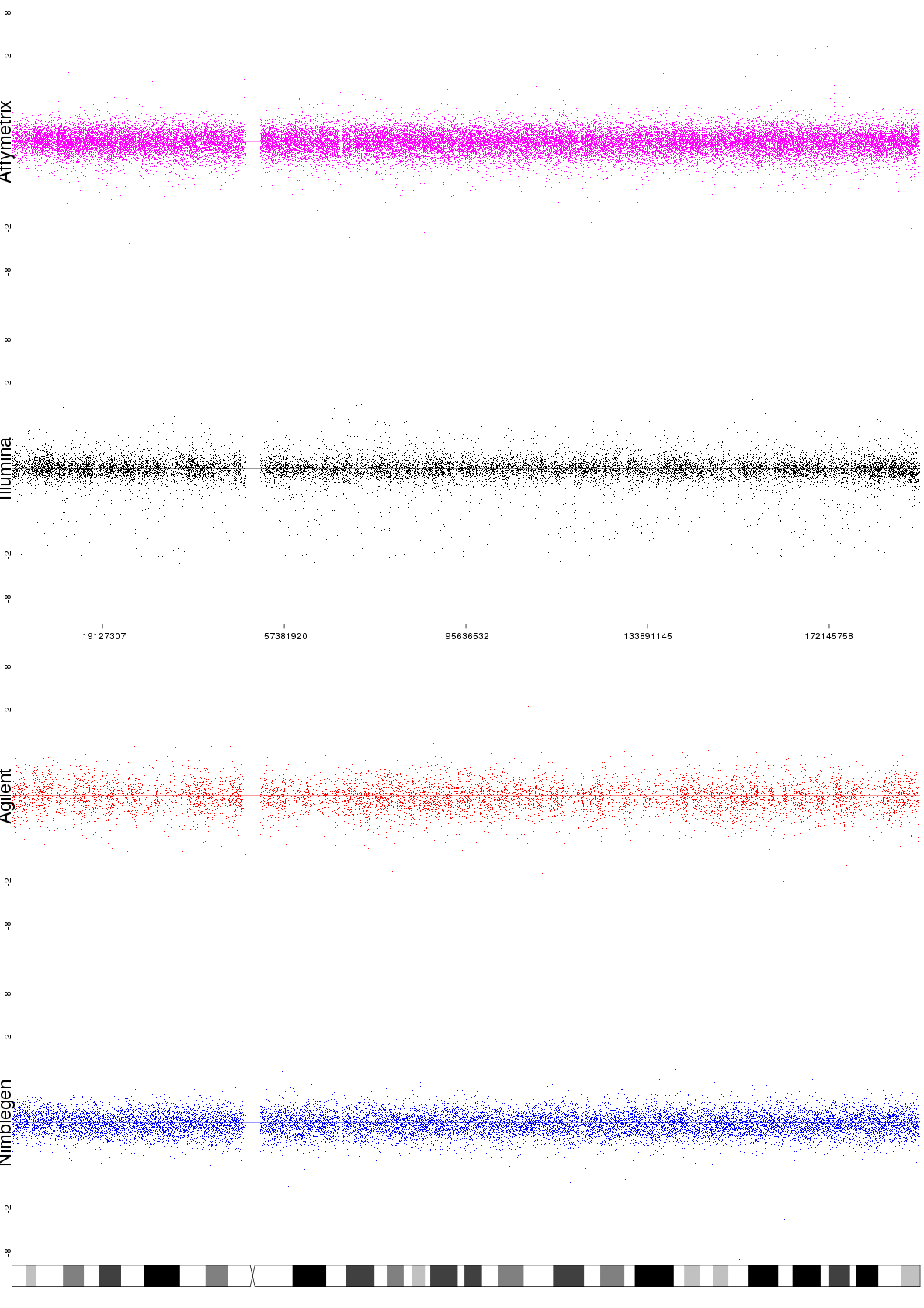

Supplement: Additional file 12 — All sample/chromosome plots for the tumours. Zip folder containing PNGs of all whole-chromosome plots for the tumours. [file 1471-2164-10-588-S12.ZIP › T7206/T7206 chromosome 4.png]

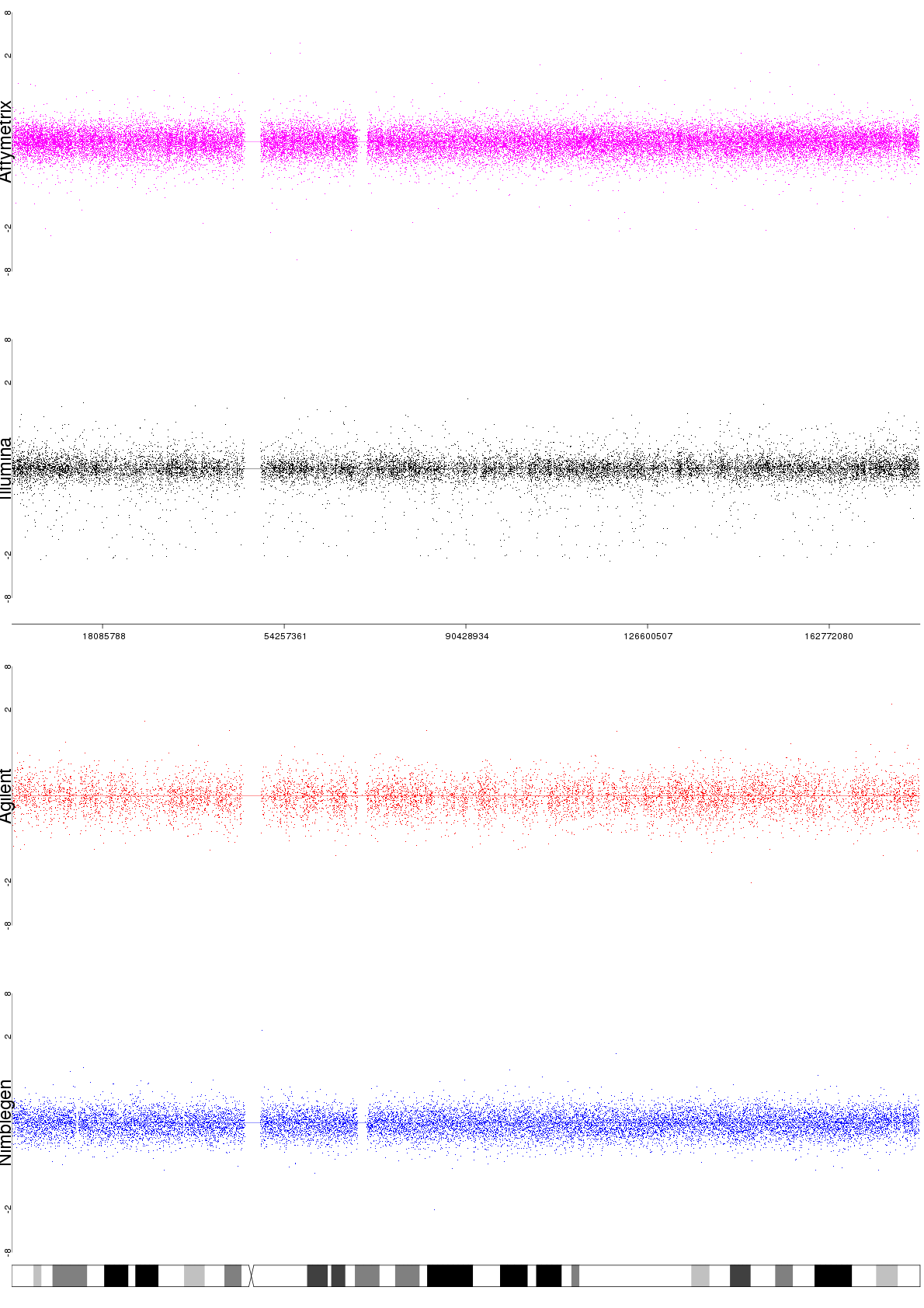

Supplement: Additional file 12 — All sample/chromosome plots for the tumours. Zip folder containing PNGs of all whole-chromosome plots for the tumours. [file 1471-2164-10-588-S12.ZIP › T7206/T7206 chromosome 5.png]

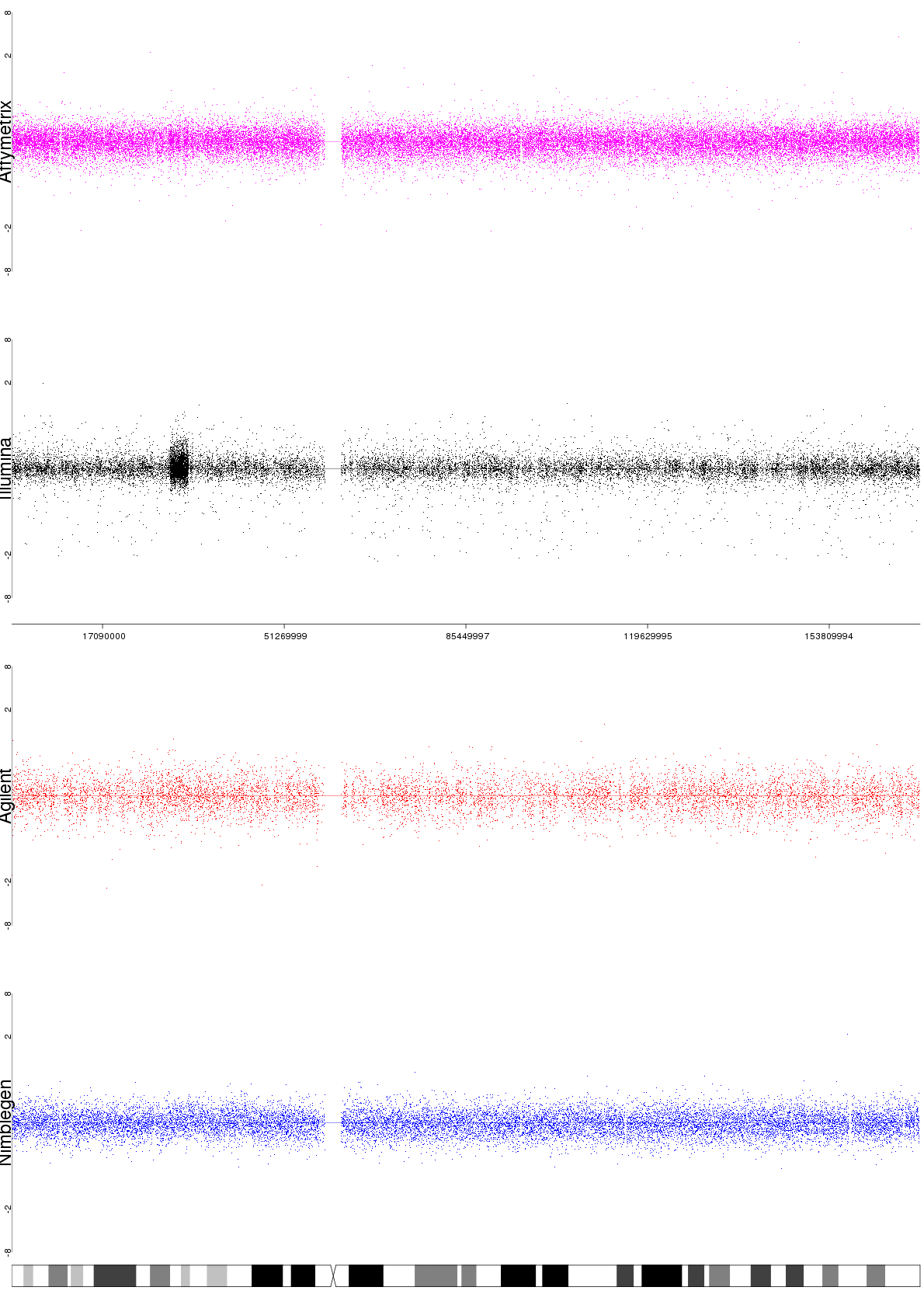

Supplement: Additional file 12 — All sample/chromosome plots for the tumours. Zip folder containing PNGs of all whole-chromosome plots for the tumours. [file 1471-2164-10-588-S12.ZIP › T7206/T7206 chromosome 6.png]

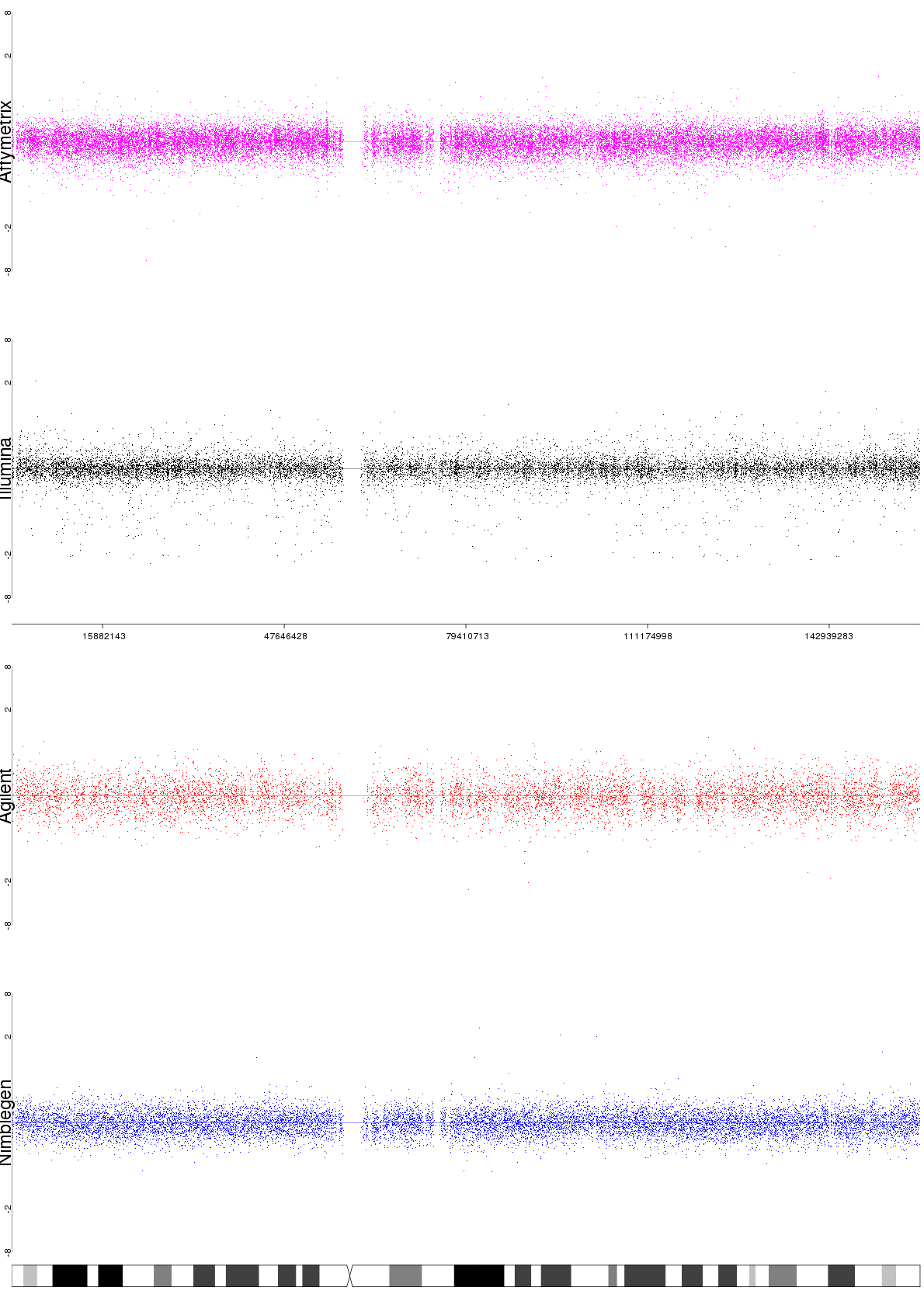

Supplement: Additional file 12 — All sample/chromosome plots for the tumours. Zip folder containing PNGs of all whole-chromosome plots for the tumours. [file 1471-2164-10-588-S12.ZIP › T7206/T7206 chromosome 7.png]

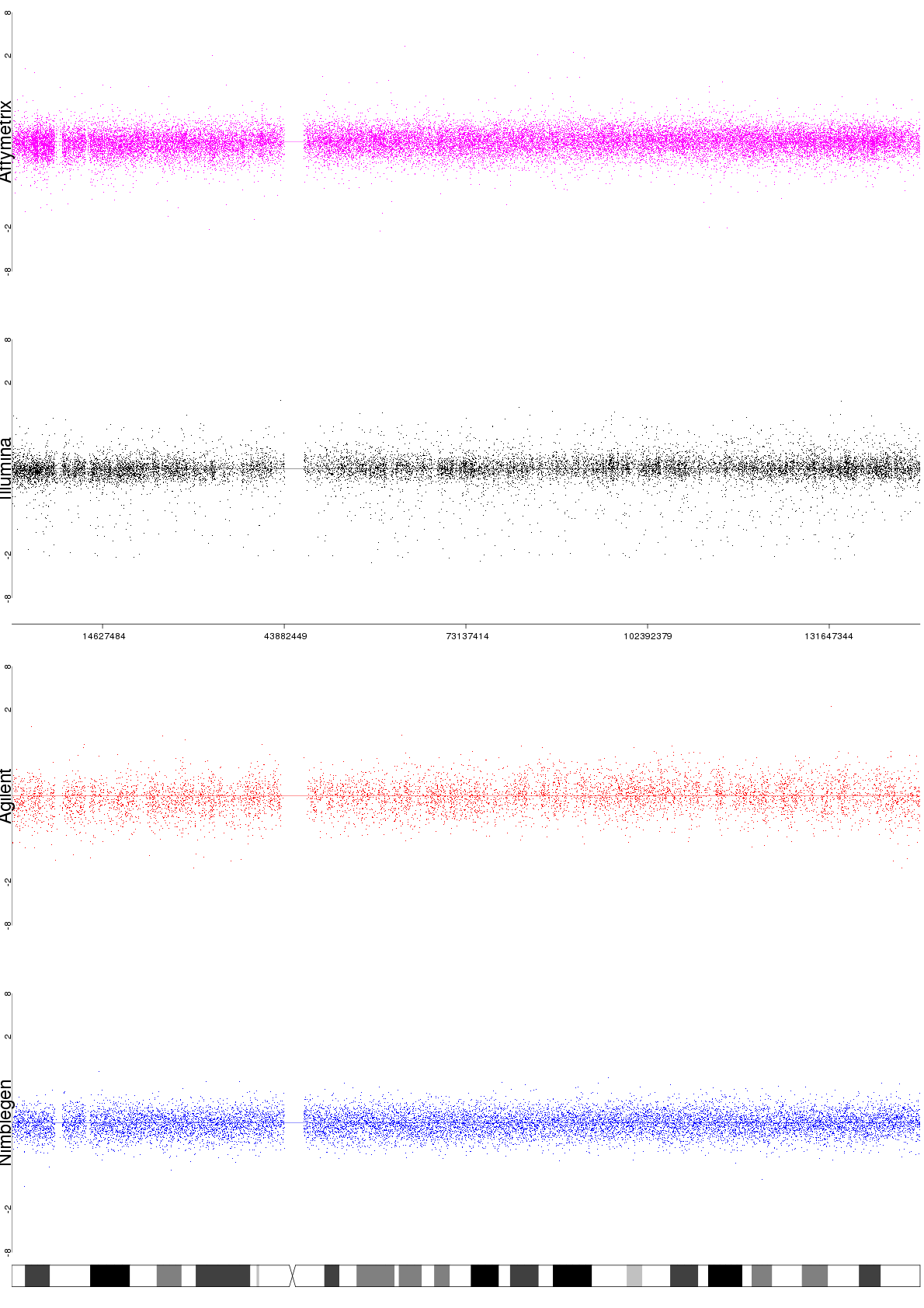

Supplement: Additional file 12 — All sample/chromosome plots for the tumours. Zip folder containing PNGs of all whole-chromosome plots for the tumours. [file 1471-2164-10-588-S12.ZIP › T7206/T7206 chromosome 8.png]

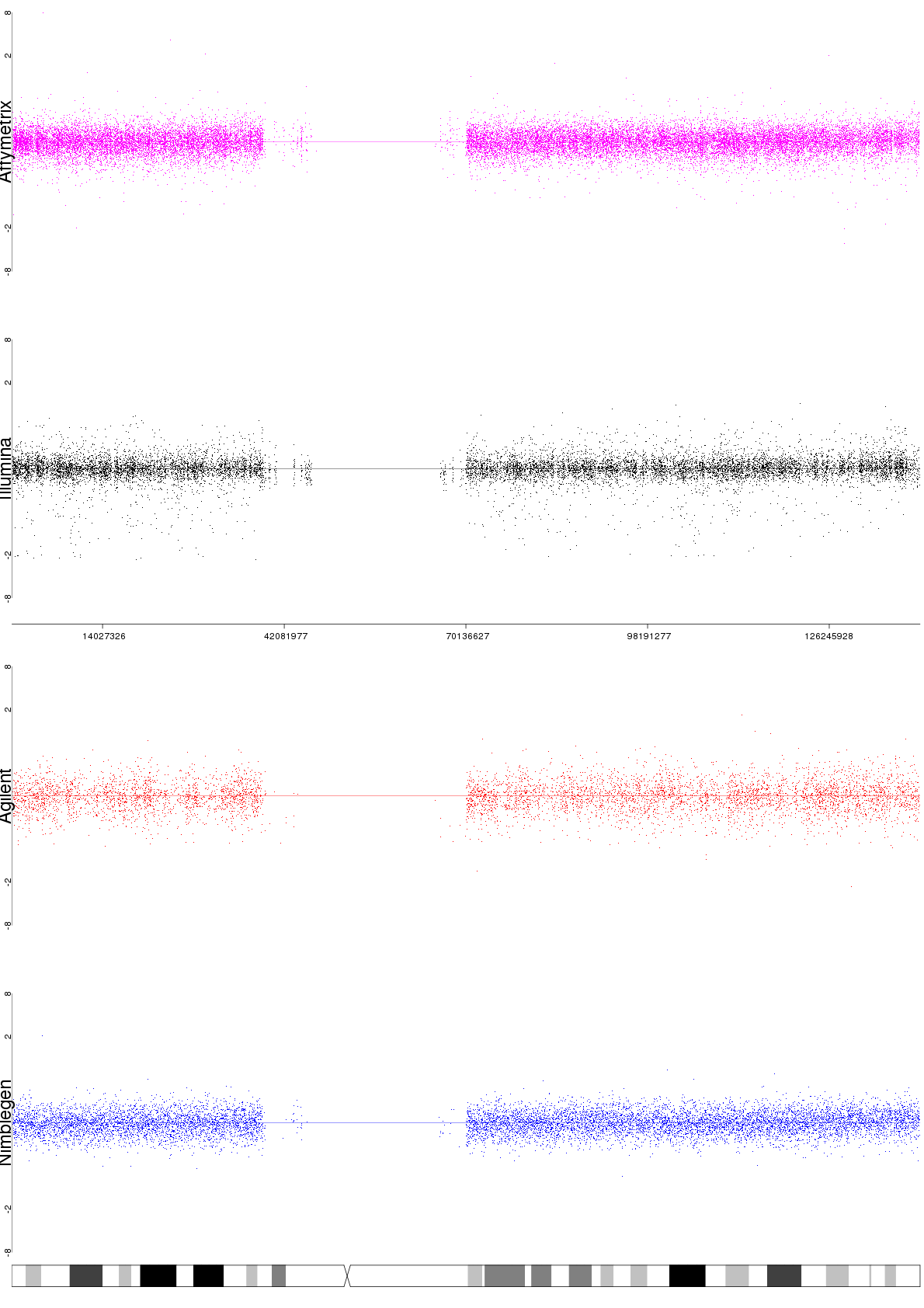

Supplement: Additional file 12 — All sample/chromosome plots for the tumours. Zip folder containing PNGs of all whole-chromosome plots for the tumours. [file 1471-2164-10-588-S12.ZIP › T7206/T7206 chromosome 9.png]

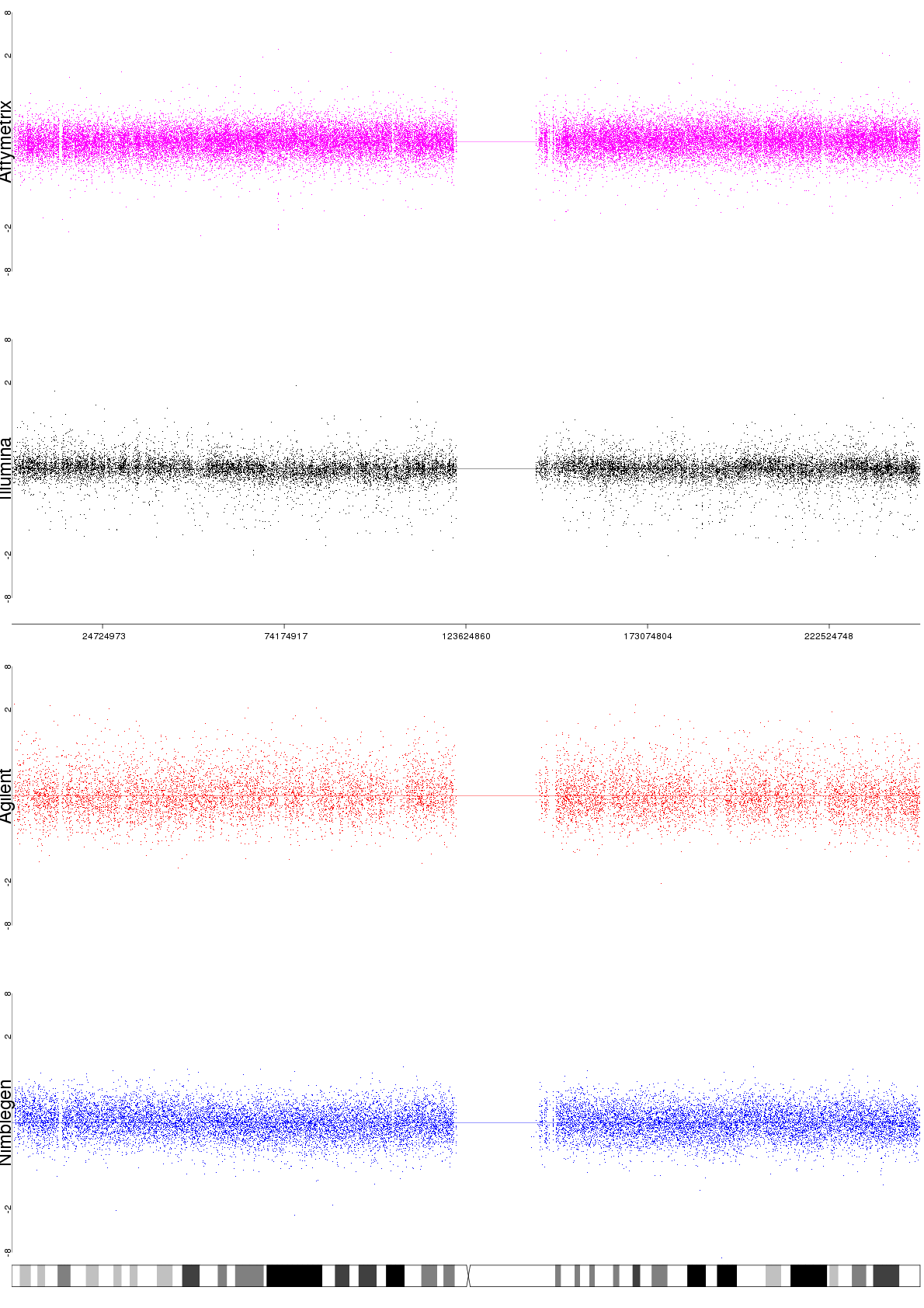

Supplement: Additional file 12 — All sample/chromosome plots for the tumours. Zip folder containing PNGs of all whole-chromosome plots for the tumours. [file 1471-2164-10-588-S12.ZIP › T7207/T7207 chromosome 1.png]
