# Supplementary material for: The pitfalls of platform comparison: DNA copy number array technologies assessed
Source: BMC Genomics. 2009 Dec 8;10:588. doi: 10.1186/1471-2164-10-588 (PMC2797821; doi:10.1186/1471-2164-10-588)
Supplement: Additional file 6 — Anticipated aberrations and known copy number changes in various samples. A list of known copy number changes for the cell-lines (SUM159, MT3, NA15510 and NA10851) and anticipated copy number changes for the tumours. [file 1471-2164-10-588-S6.PDF]

Sample	Location	Abberation
MT3 Cell line	Chromosome 7	Single copy gain
MT3 Cell line	Isochromosome 13	Single copy gain
MT3 Cell line	Chromosome X	loss
SUM159 Cell line	Chromosome 5q	loss
SUM159 Cell line	Chromosome 5p	gain
SUM159 Cell line	Chromosome 8q24	gain
SUM159 Cell line	Chromosome 17p	loss
SUM159 Cell line	Chromosome 9p21	loss
SUM159 Cell line	Chromosome 8q24	gain
SUM159 Cell line	Chromosome 7pTel	gain
SUM159 Cell line	Chromosome 3pCent	loss
SUM159 Cell line	Chromosome 17	translocation
Tumours	Chromosome 17 (ERBB2)	Gain
Tumours	Chromosome 11q13	gain
Tumours	Chromosome 17q12	gain
Tumours	Chromosome 18	gains
Tumours	Chromosome 8p	gain
Tumours	Chromosome 1q	gain
Tumours	Chromosome 16q	loss
Tumours	Chromosome 8q24	gain
Tumours	Chromosome 17q12	gain
Tumours	Chromosome 17p13	loss
Tumours	Chromosome 20q13	gain
Tumours	Chromosome 13q14	loss
Tumours	Chromosome 9p21	loss
HapMap Pair	1 12757377-12842343	CNV
HapMap Pair	1 103898814-104049621	CNV
HapMap Pair	1 108504425-108699273	CNV
HapMap Pair	1 120245520-120395759	CNV
HapMap Pair	1 141851837-141979099	CNV
HapMap Pair	1 142391276-142548088	CNV
HapMap Pair	1 142678316-142882622	CNV
HapMap Pair	1 145888591-146093292	CNV
HapMap Pair	1 199210505-199386132	CNV
HapMap Pair	2 87958554-88112870	CNV
HapMap Pair	2 88979593-89141717	CNV
HapMap Pair	2 89792857-90016977	CNV
HapMap Pair	2 97112914-97303968	CNV
HapMap Pair	2 127547173-127722744	CNV
HapMap Pair	2 131780178-131953801	CNV
HapMap Pair	3 19416349-19587692	CNV
HapMap Pair	3 19709162-19957065	CNV
HapMap Pair	3 20240591-20399098	CNV
HapMap Pair	3 20554037-20750375	CNV
HapMap Pair	3 98699482-98904950	CNV
HapMap Pair	3 196868577-197108192	CNV
HapMap Pair	4 9000913-9120591	CNV
HapMap Pair	4 34608481-34779109	CNV
HapMap Pair	4 49329870-49501045	CNV
HapMap Pair	4 70277448-70448849	CNV
HapMap Pair	4 144705898-144872906	CNV
HapMap Pair	4 169237638-169426423	CNV
HapMap Pair	4 190867229-191070245	CNV
HapMap Pair	5 673163-895224	CNV
HapMap Pair	5 17465573-17597918	CNV
HapMap Pair	5 69475945-69679055	CNV
HapMap Pair	5 93886642-94067957	CNV

HapMap Pair	6 143357-346084	CNV
HapMap Pair	6 78947195-79116378	CNV
HapMap Pair	7 64424689-64515187	CNV
HapMap Pair	7 99217194-99399792	CNV
HapMap Pair	7 133094440-133281052	CNV
HapMap Pair	7 143479367-143635318	CNV
HapMap Pair	8 3329651-3488923	CNV
HapMap Pair	8 47728697-47901824	CNV
HapMap Pair	8 51049146-51300548	CNV
HapMap Pair	8 86592730-86763703	CNV
HapMap Pair	9 38859439-39036984	CNV
HapMap Pair	9 40012313-40155117	CNV
HapMap Pair	9 41065891-41216390	CNV
HapMap Pair	9 43409087-43711107	CNV
HapMap Pair	10 46046581-46428959	CNV
HapMap Pair	10 46560330-46738511	CNV
HapMap Pair	10 47796101-47972148	CNV
HapMap Pair	10 56078033-56236143	CNV
HapMap Pair	10 88715605-89041452	CNV
HapMap Pair	10 127443890-127606660	CNV
HapMap Pair	11 60671936-60837233	CNV
HapMap Pair	12 56116000-56288135	CNV
HapMap Pair	14 18732531-18920839	CNV
HapMap Pair	14 30251821-30406994	CNV
HapMap Pair	14 31928231-32086193	CNV
HapMap Pair	14 73030050-73196823	CNV
HapMap Pair	14 105476363-105701373	CNV
HapMap Pair	15 18419709-18576834	CNV
HapMap Pair	15 18747225-18935829	CNV
HapMap Pair	15 19050502-19257408	CNV
HapMap Pair	15 94433027-94616040	CNV
HapMap Pair	16 21408063-21833734	CNV
HapMap Pair	16 22338076-22679810	CNV
HapMap Pair	16 32722382-32902091	CNV
HapMap Pair	16 33282424-33715261	CNV
HapMap Pair	17 21850381-22014559	CNV
HapMap Pair	17 31309398-31593135	CNV
HapMap Pair	17 33376424-33649105	CNV
HapMap Pair	18 61788347-61987071	CNV
HapMap Pair	19 45983467-46152643	CNV
HapMap Pair	19 55228822-55344796	CNV
HapMap Pair	20 14757045-14926976	CNV
HapMap Pair	20 25894247-26015566	CNV
HapMap Pair	20 61685172-61846811	CNV
HapMap Pair	22 14509865-14549165	CNV
HapMap Pair	22 20983001-21045994	CNV
HapMap Pair	22 44975462-45018495	CNV
