# Supplementary figures and images for: The pitfalls of platform comparison: DNA copy number array technologies assessed (part 2 of 3)
Source: BMC Genomics. 2009 Dec 8;10:588. doi: 10.1186/1471-2164-10-588 (PMC2797821; doi:10.1186/1471-2164-10-588)

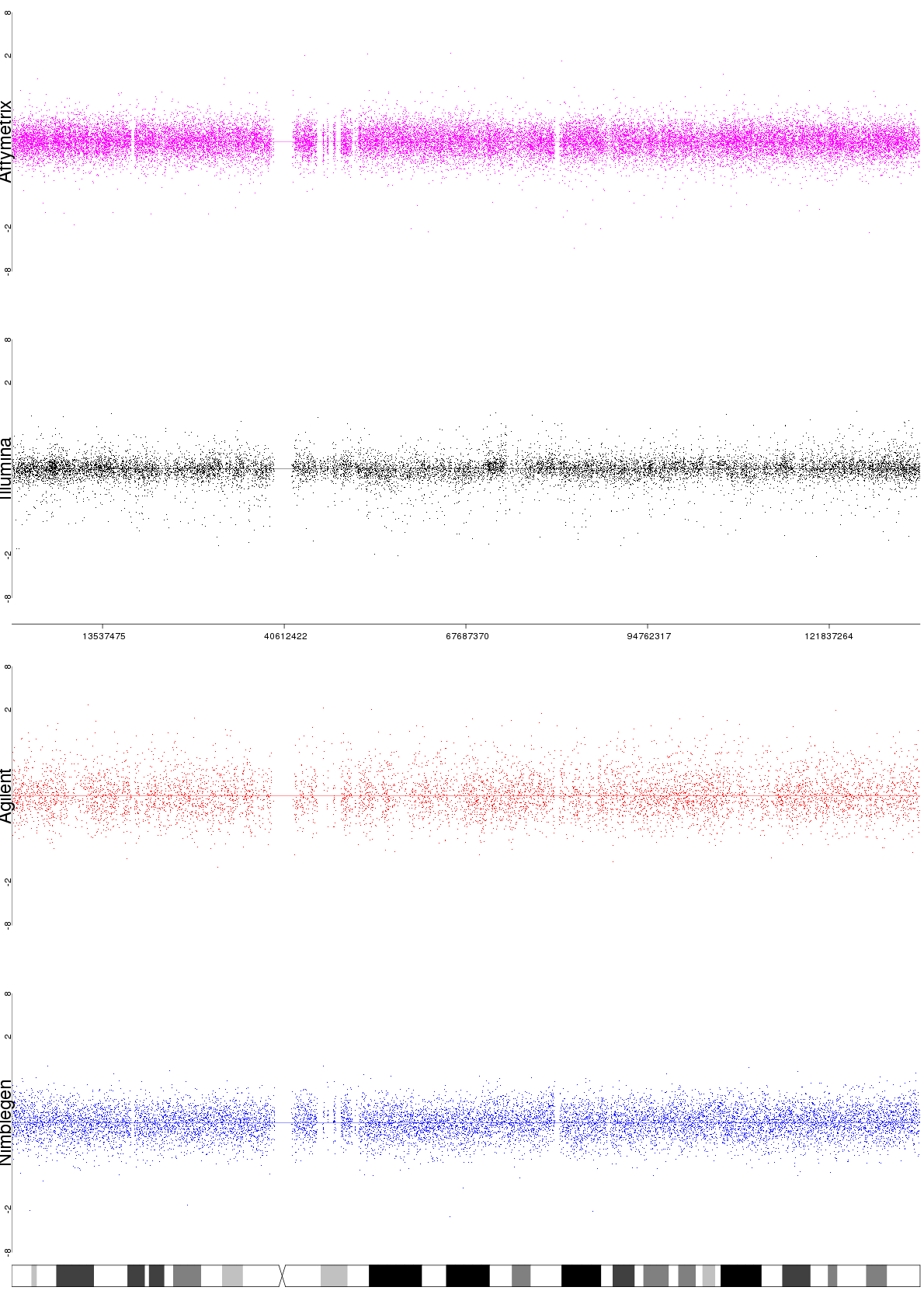

Supplement: Additional file 12 — All sample/chromosome plots for the tumours. Zip folder containing PNGs of all whole-chromosome plots for the tumours. [file 1471-2164-10-588-S12.ZIP › T7207/T7207 chromosome 10.png]

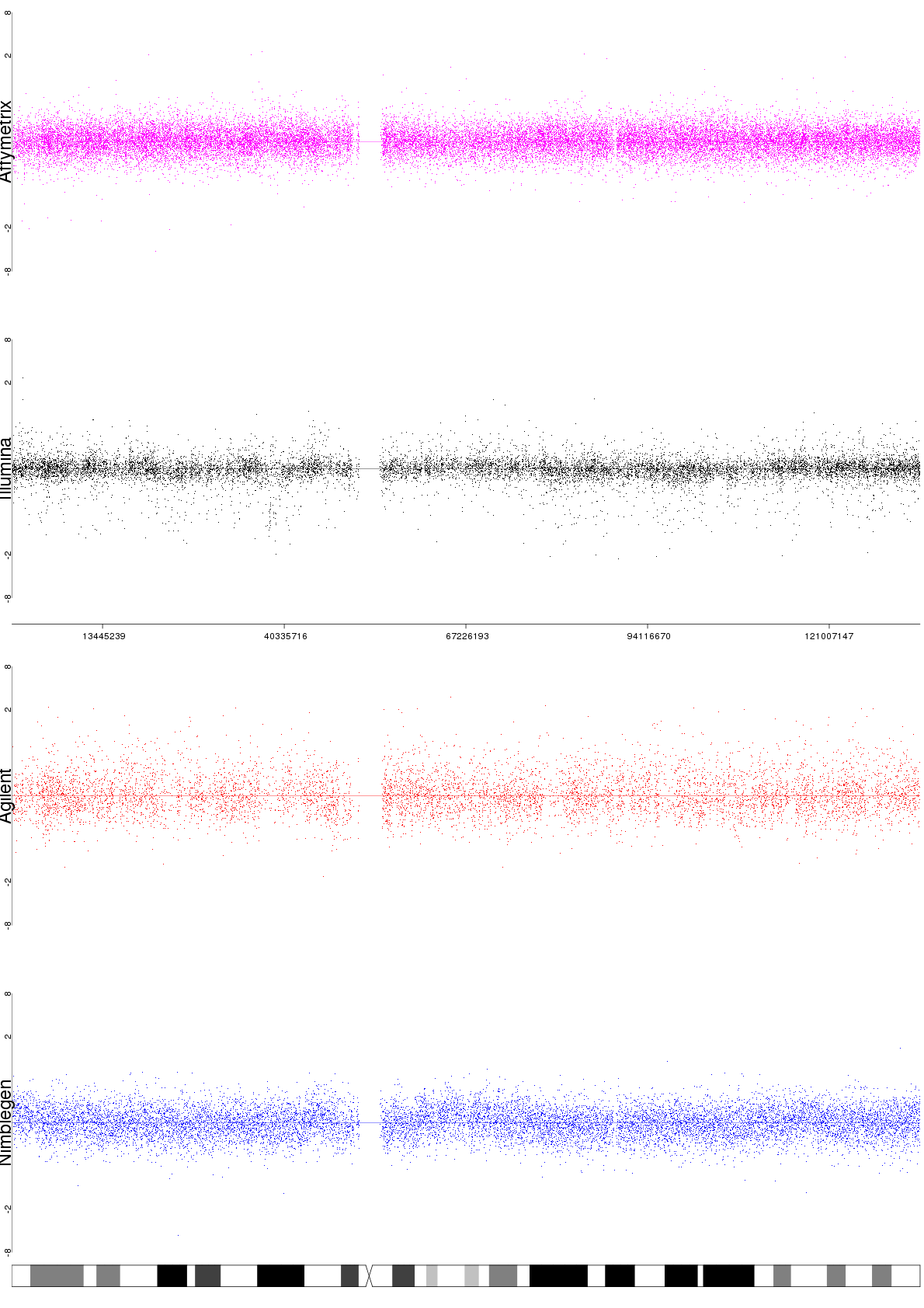

Supplement: Additional file 12 — All sample/chromosome plots for the tumours. Zip folder containing PNGs of all whole-chromosome plots for the tumours. [file 1471-2164-10-588-S12.ZIP › T7207/T7207 chromosome 11.png]

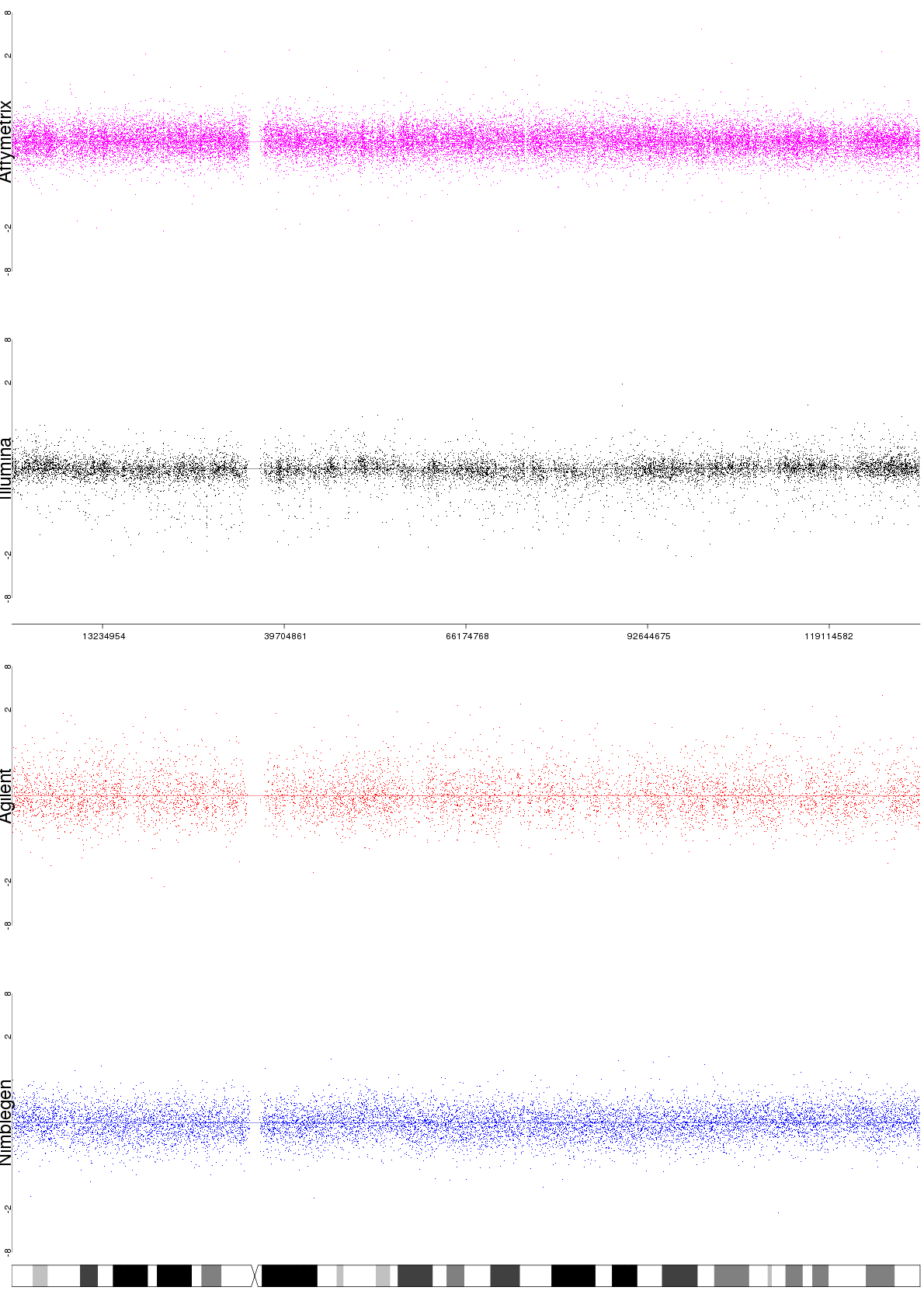

Supplement: Additional file 12 — All sample/chromosome plots for the tumours. Zip folder containing PNGs of all whole-chromosome plots for the tumours. [file 1471-2164-10-588-S12.ZIP › T7207/T7207 chromosome 12.png]

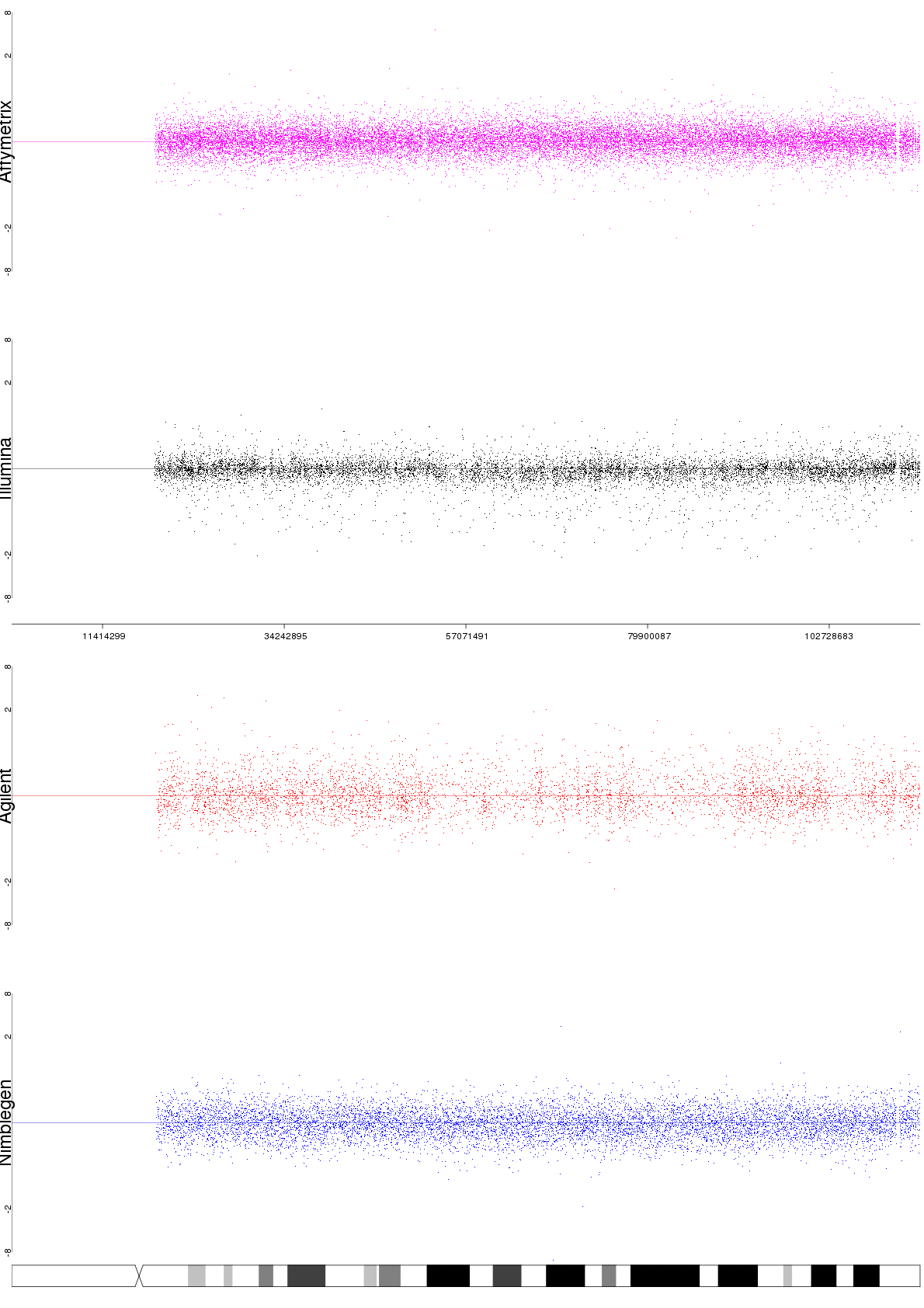

Supplement: Additional file 12 — All sample/chromosome plots for the tumours. Zip folder containing PNGs of all whole-chromosome plots for the tumours. [file 1471-2164-10-588-S12.ZIP › T7207/T7207 chromosome 13.png]

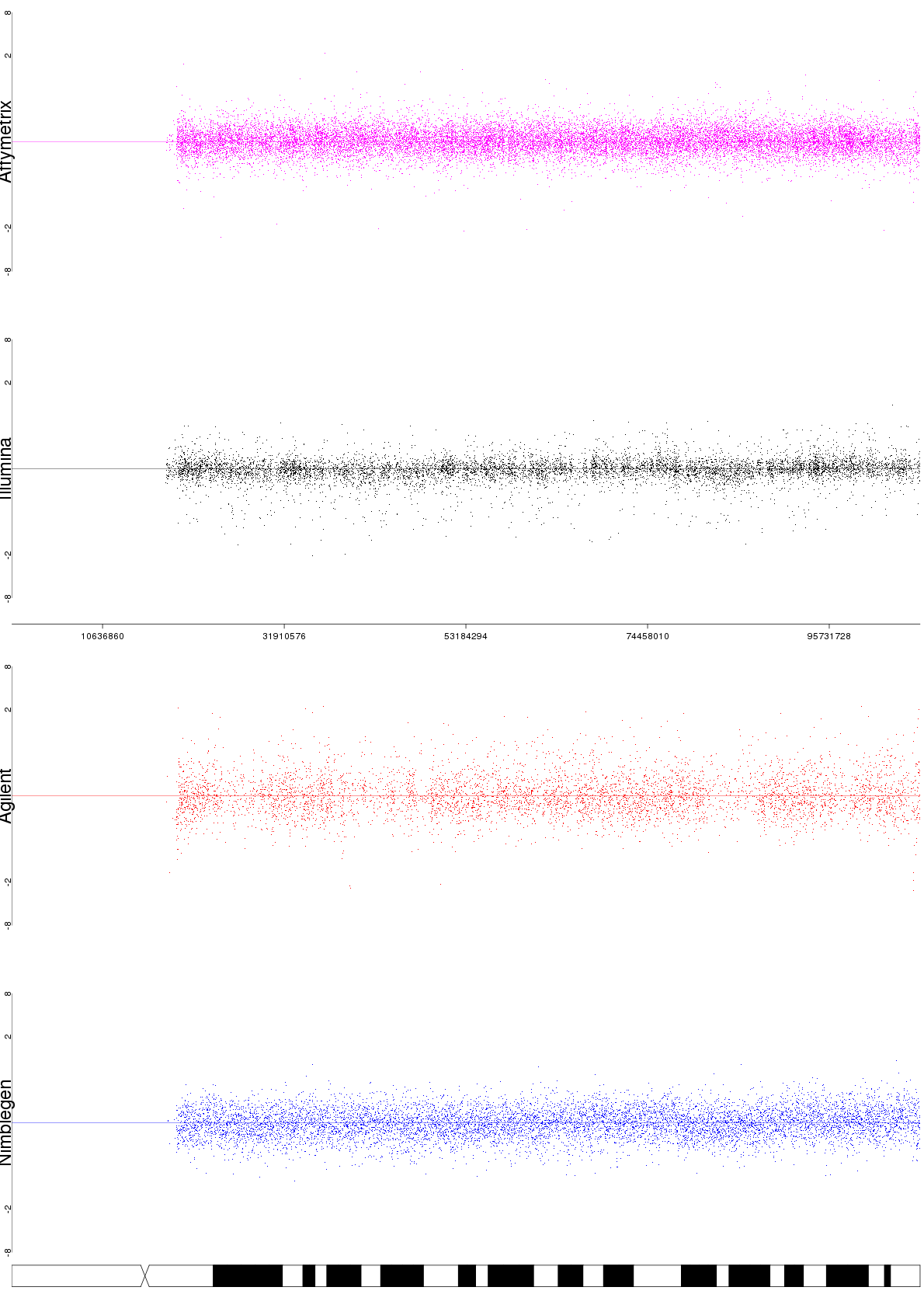

Supplement: Additional file 12 — All sample/chromosome plots for the tumours. Zip folder containing PNGs of all whole-chromosome plots for the tumours. [file 1471-2164-10-588-S12.ZIP › T7207/T7207 chromosome 14.png]

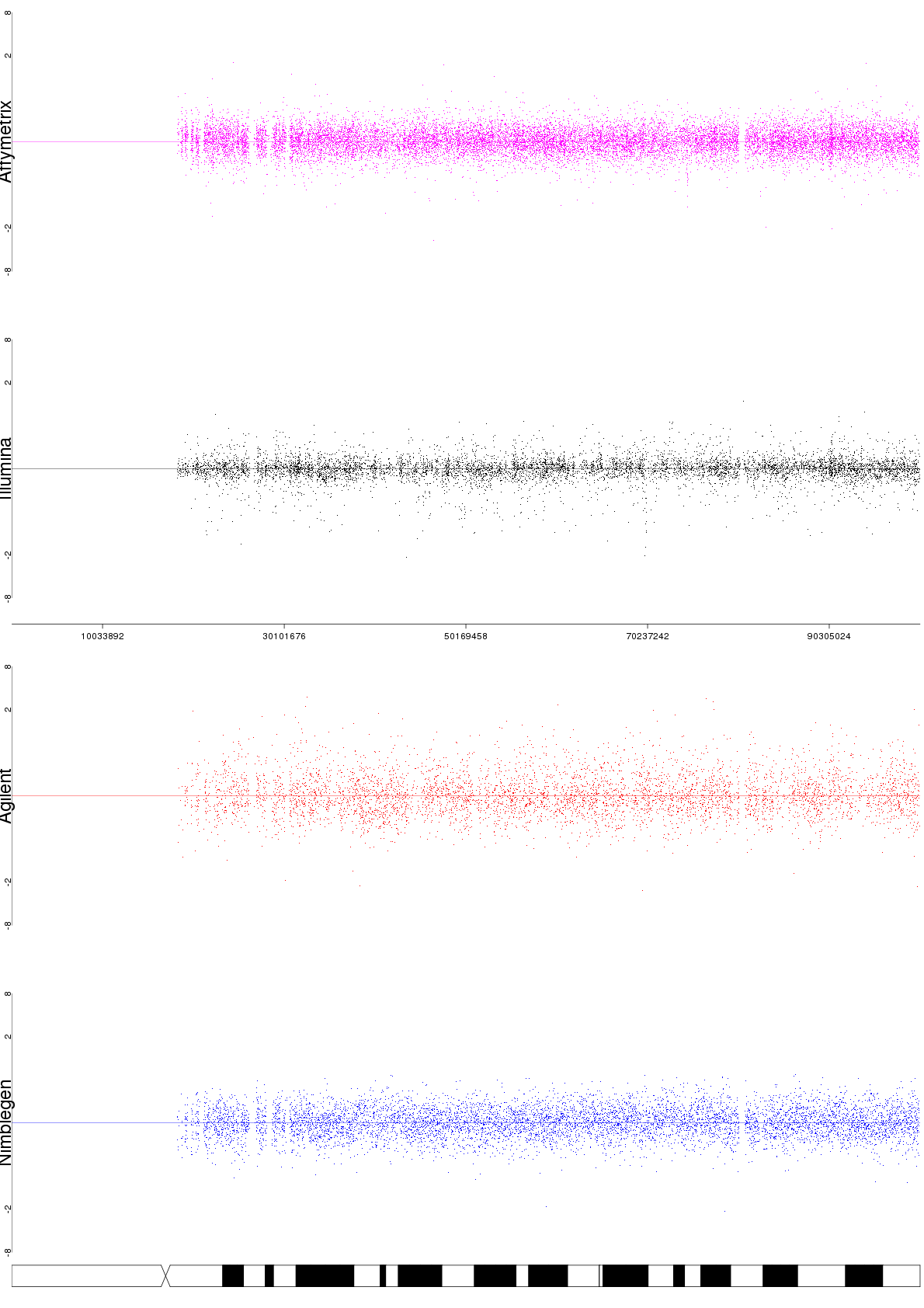

Supplement: Additional file 12 — All sample/chromosome plots for the tumours. Zip folder containing PNGs of all whole-chromosome plots for the tumours. [file 1471-2164-10-588-S12.ZIP › T7207/T7207 chromosome 15.png]

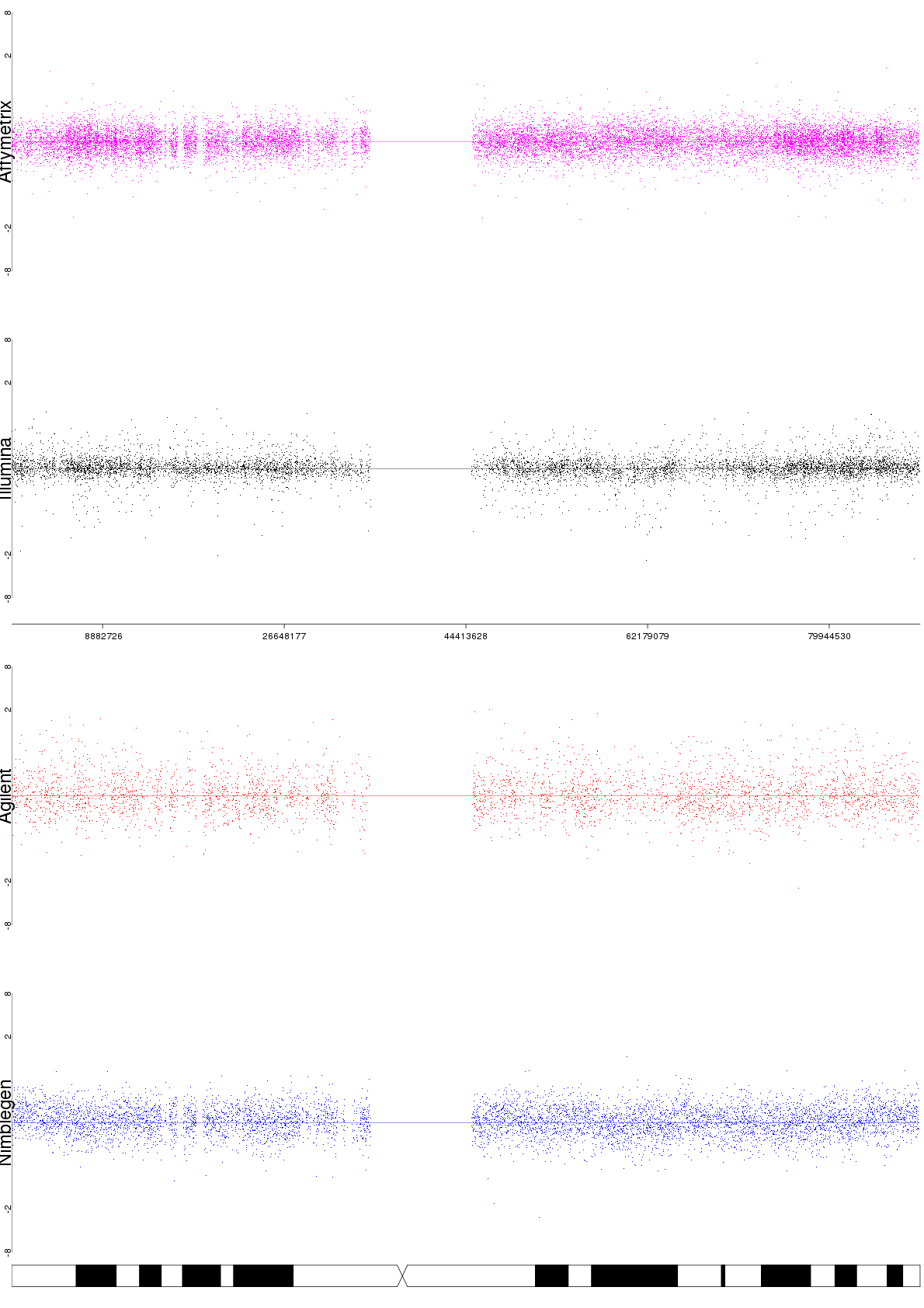

Supplement: Additional file 12 — All sample/chromosome plots for the tumours. Zip folder containing PNGs of all whole-chromosome plots for the tumours. [file 1471-2164-10-588-S12.ZIP › T7207/T7207 chromosome 16.png]

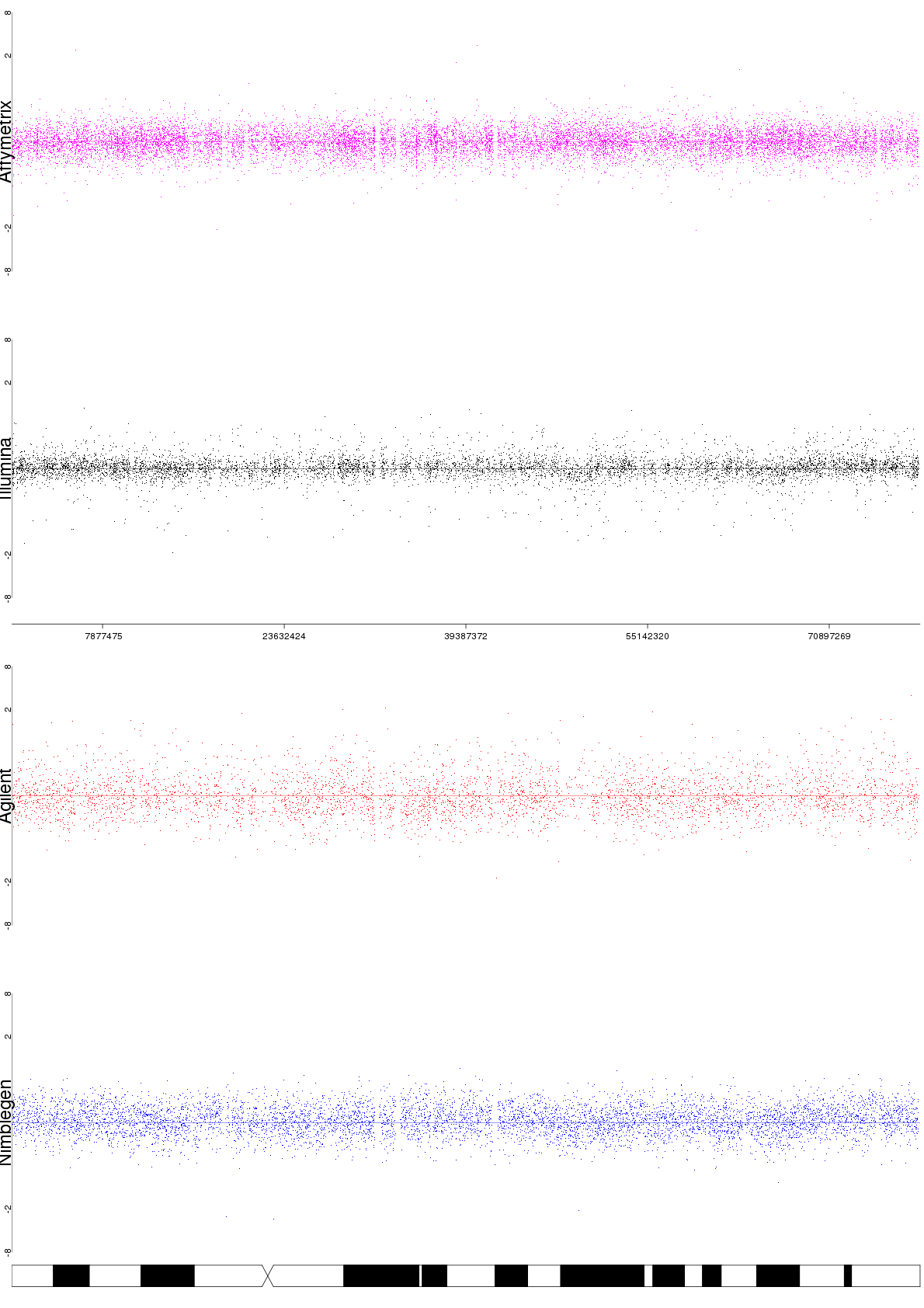

Supplement: Additional file 12 — All sample/chromosome plots for the tumours. Zip folder containing PNGs of all whole-chromosome plots for the tumours. [file 1471-2164-10-588-S12.ZIP › T7207/T7207 chromosome 17.png]

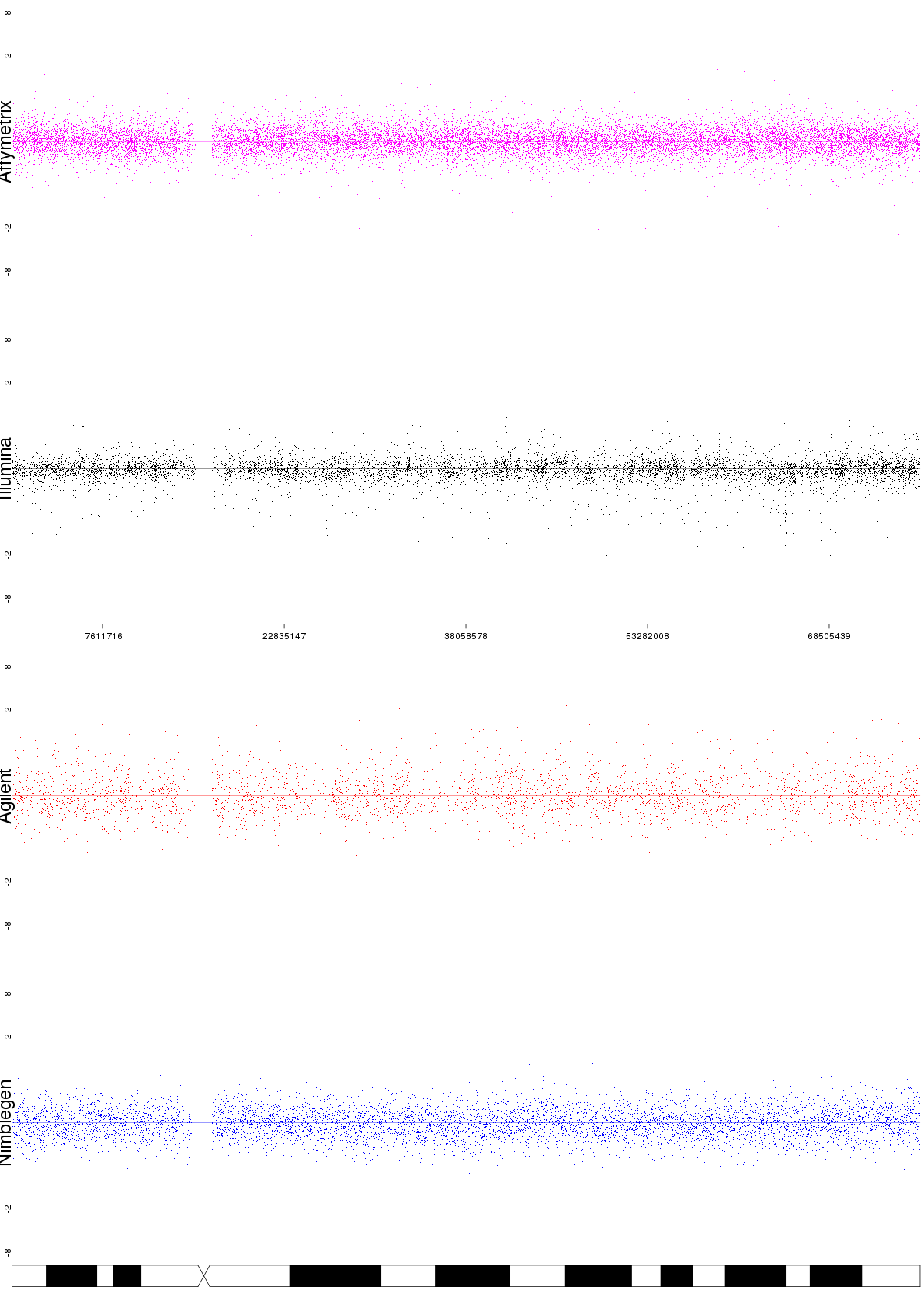

Supplement: Additional file 12 — All sample/chromosome plots for the tumours. Zip folder containing PNGs of all whole-chromosome plots for the tumours. [file 1471-2164-10-588-S12.ZIP › T7207/T7207 chromosome 18.png]

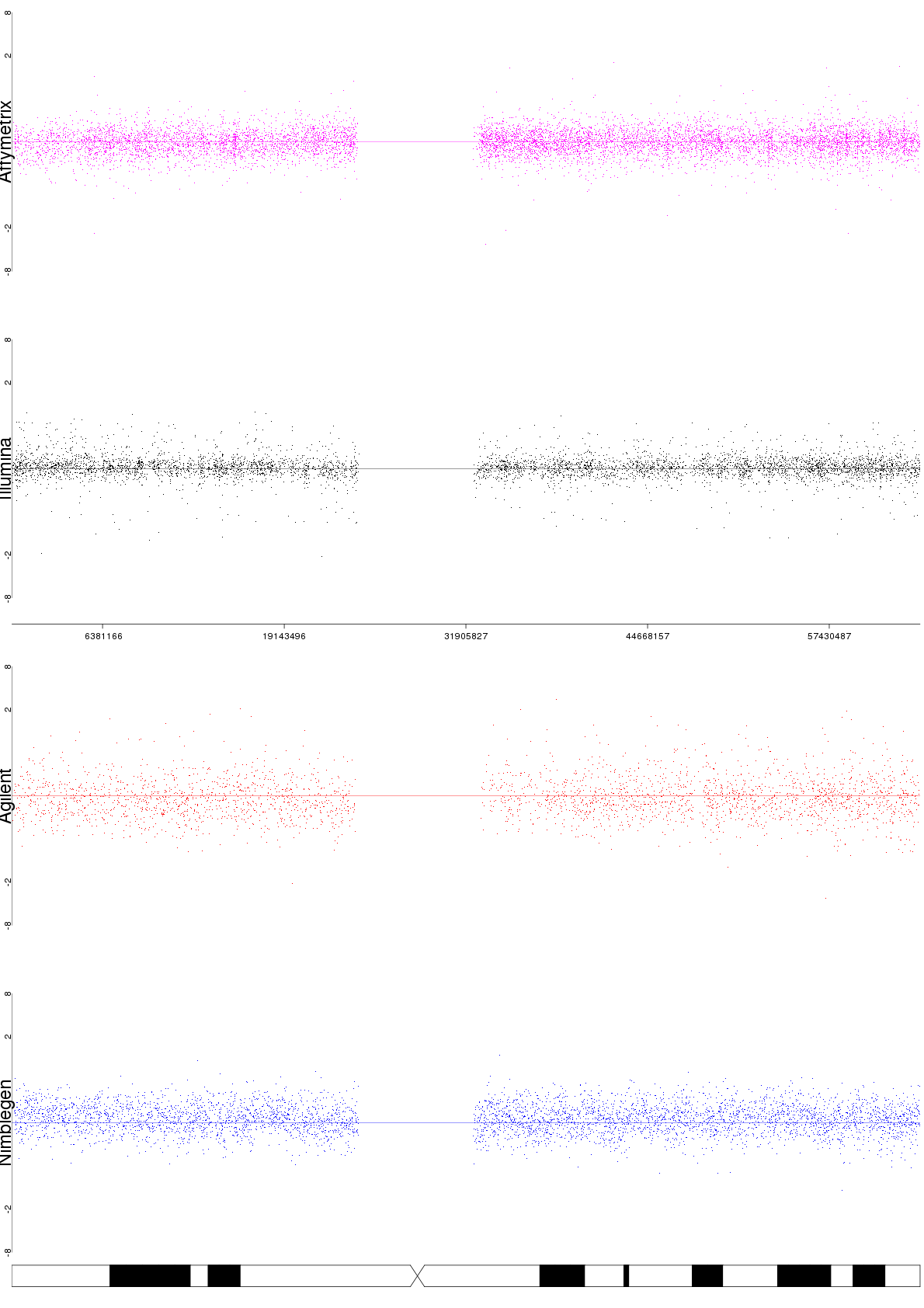

Supplement: Additional file 12 — All sample/chromosome plots for the tumours. Zip folder containing PNGs of all whole-chromosome plots for the tumours. [file 1471-2164-10-588-S12.ZIP › T7207/T7207 chromosome 19.png]

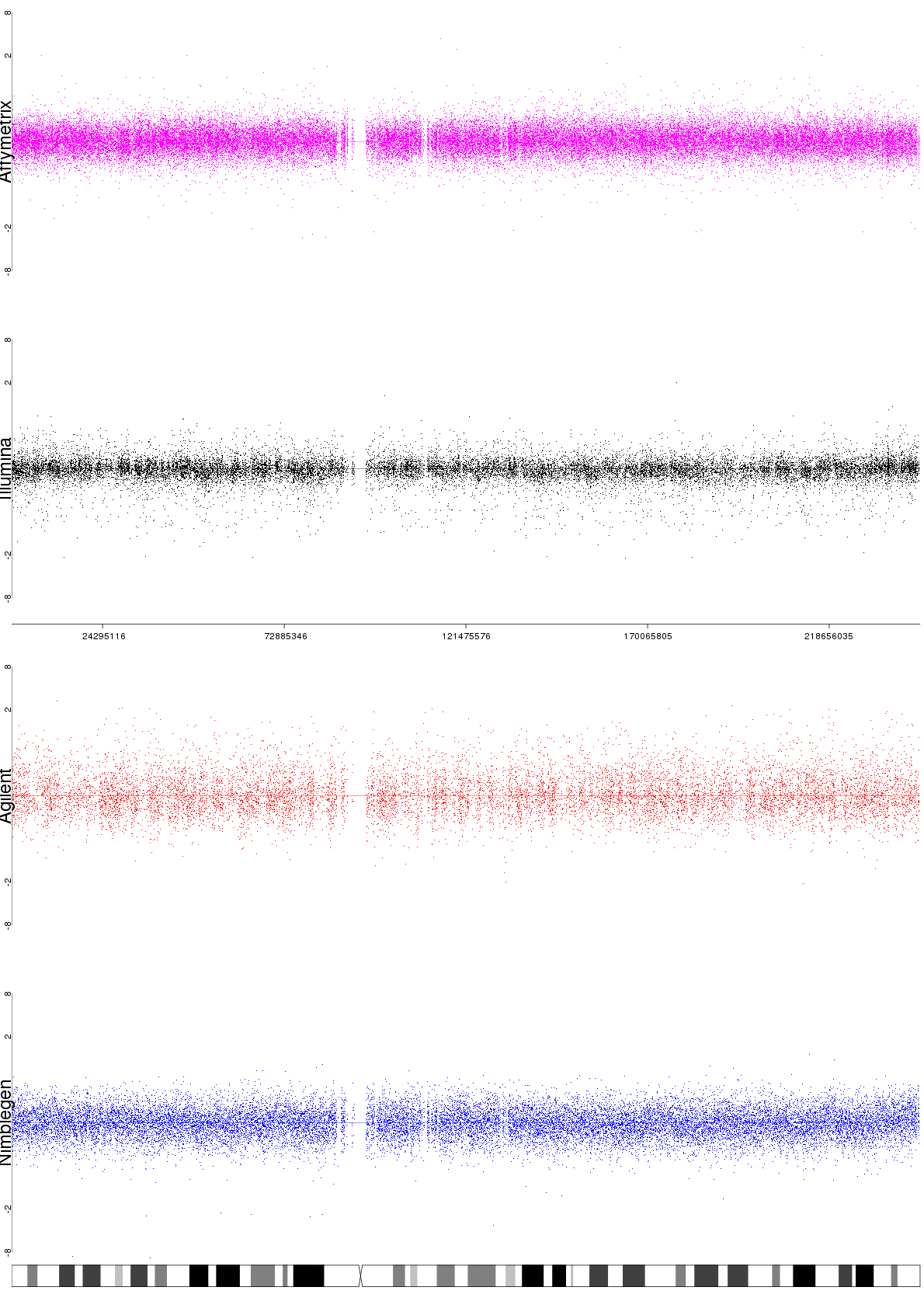

Supplement: Additional file 12 — All sample/chromosome plots for the tumours. Zip folder containing PNGs of all whole-chromosome plots for the tumours. [file 1471-2164-10-588-S12.ZIP › T7207/T7207 chromosome 2.png]

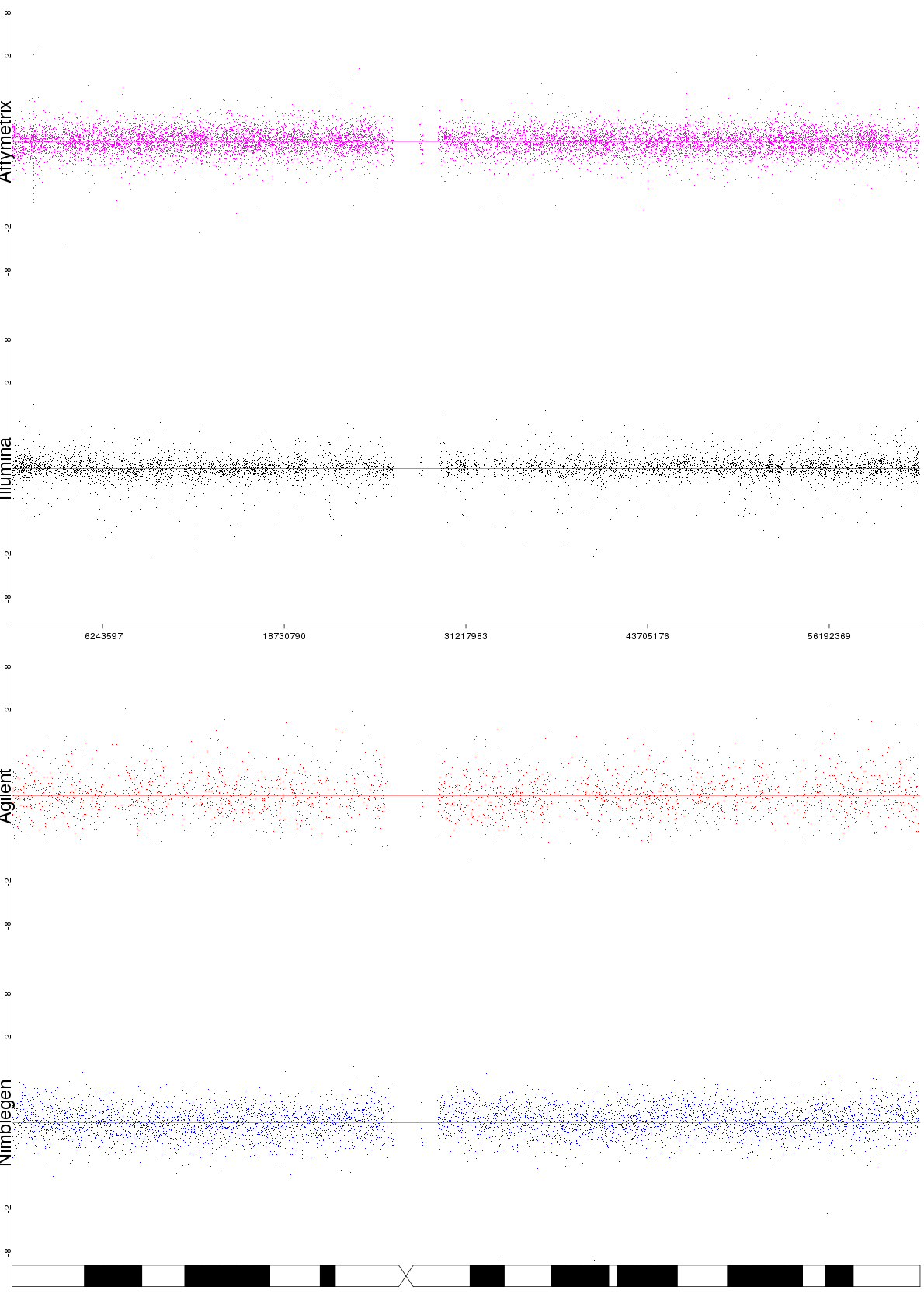

Supplement: Additional file 12 — All sample/chromosome plots for the tumours. Zip folder containing PNGs of all whole-chromosome plots for the tumours. [file 1471-2164-10-588-S12.ZIP › T7207/T7207 chromosome 20.png]

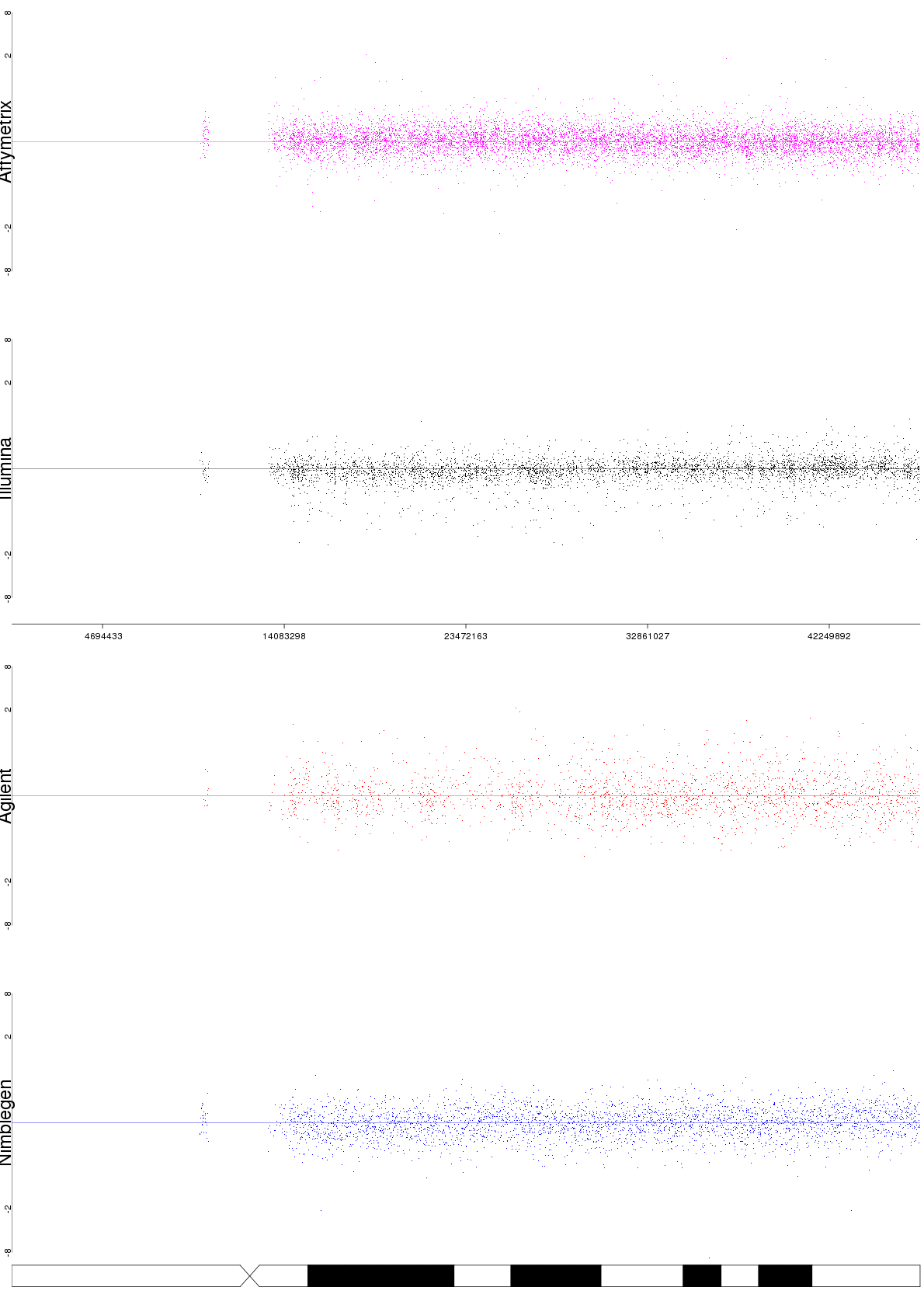

Supplement: Additional file 12 — All sample/chromosome plots for the tumours. Zip folder containing PNGs of all whole-chromosome plots for the tumours. [file 1471-2164-10-588-S12.ZIP › T7207/T7207 chromosome 21.png]

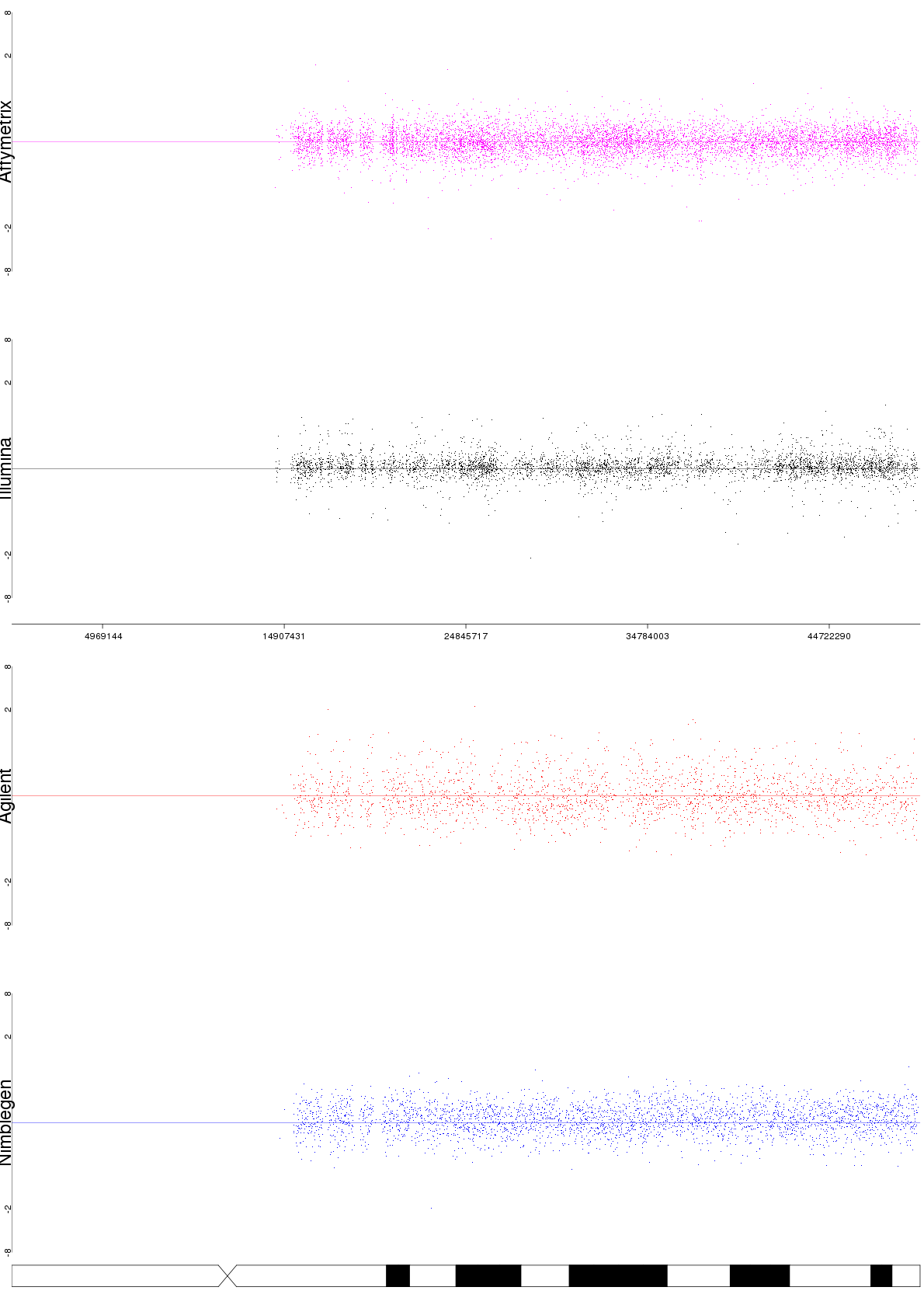

Supplement: Additional file 12 — All sample/chromosome plots for the tumours. Zip folder containing PNGs of all whole-chromosome plots for the tumours. [file 1471-2164-10-588-S12.ZIP › T7207/T7207 chromosome 22.png]

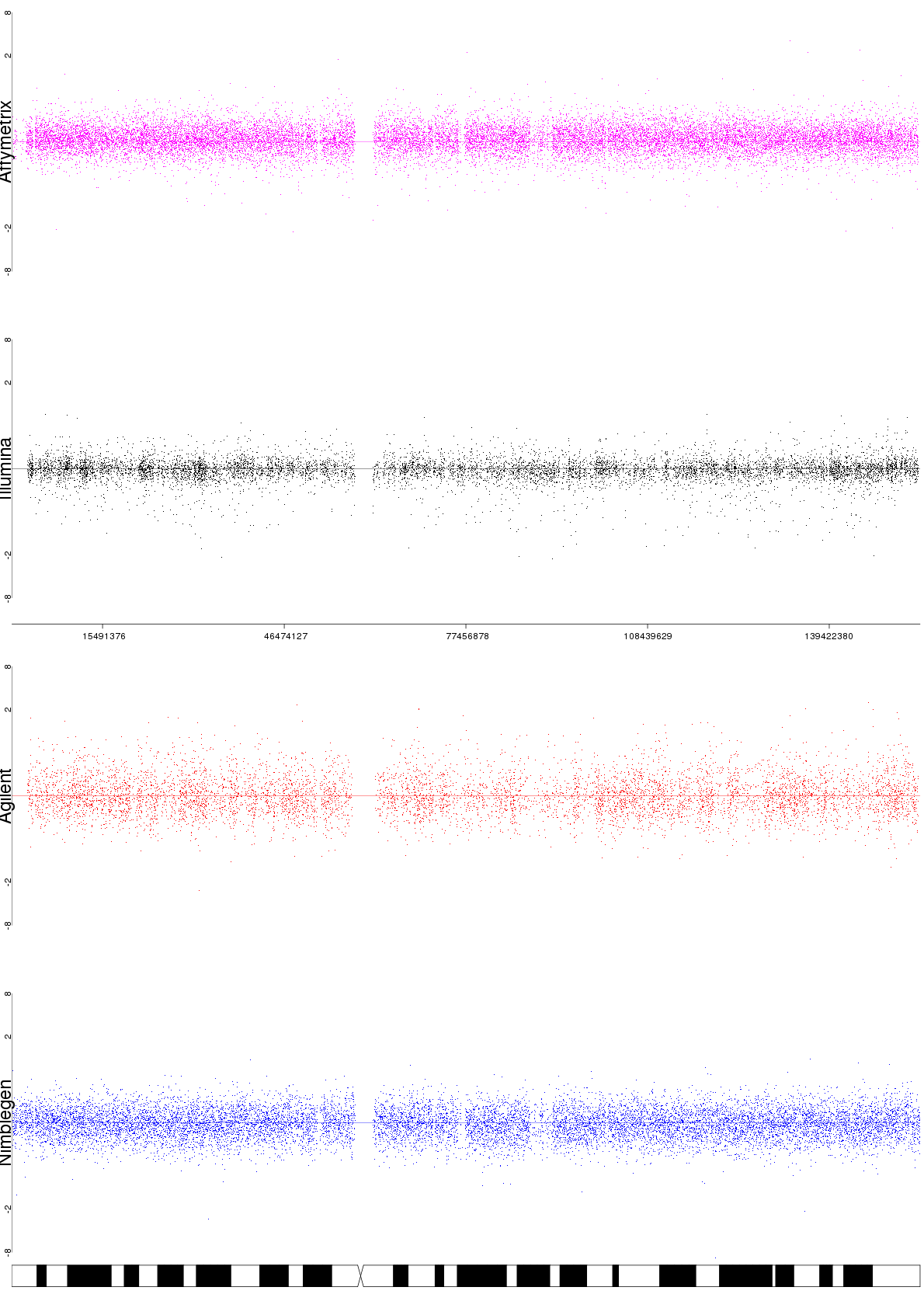

Supplement: Additional file 12 — All sample/chromosome plots for the tumours. Zip folder containing PNGs of all whole-chromosome plots for the tumours. [file 1471-2164-10-588-S12.ZIP › T7207/T7207 chromosome 23.png]

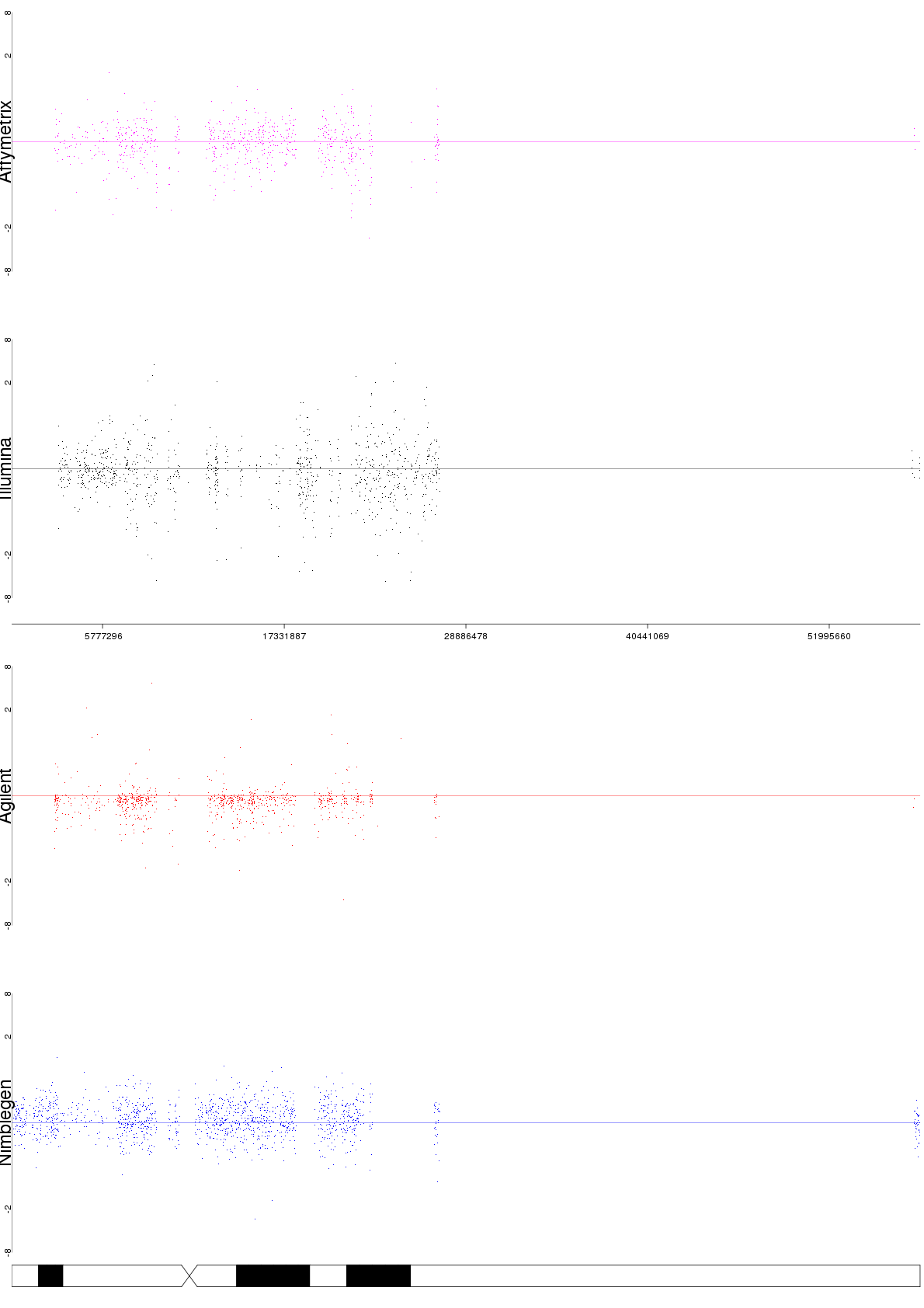

Supplement: Additional file 12 — All sample/chromosome plots for the tumours. Zip folder containing PNGs of all whole-chromosome plots for the tumours. [file 1471-2164-10-588-S12.ZIP › T7207/T7207 chromosome 24.png]

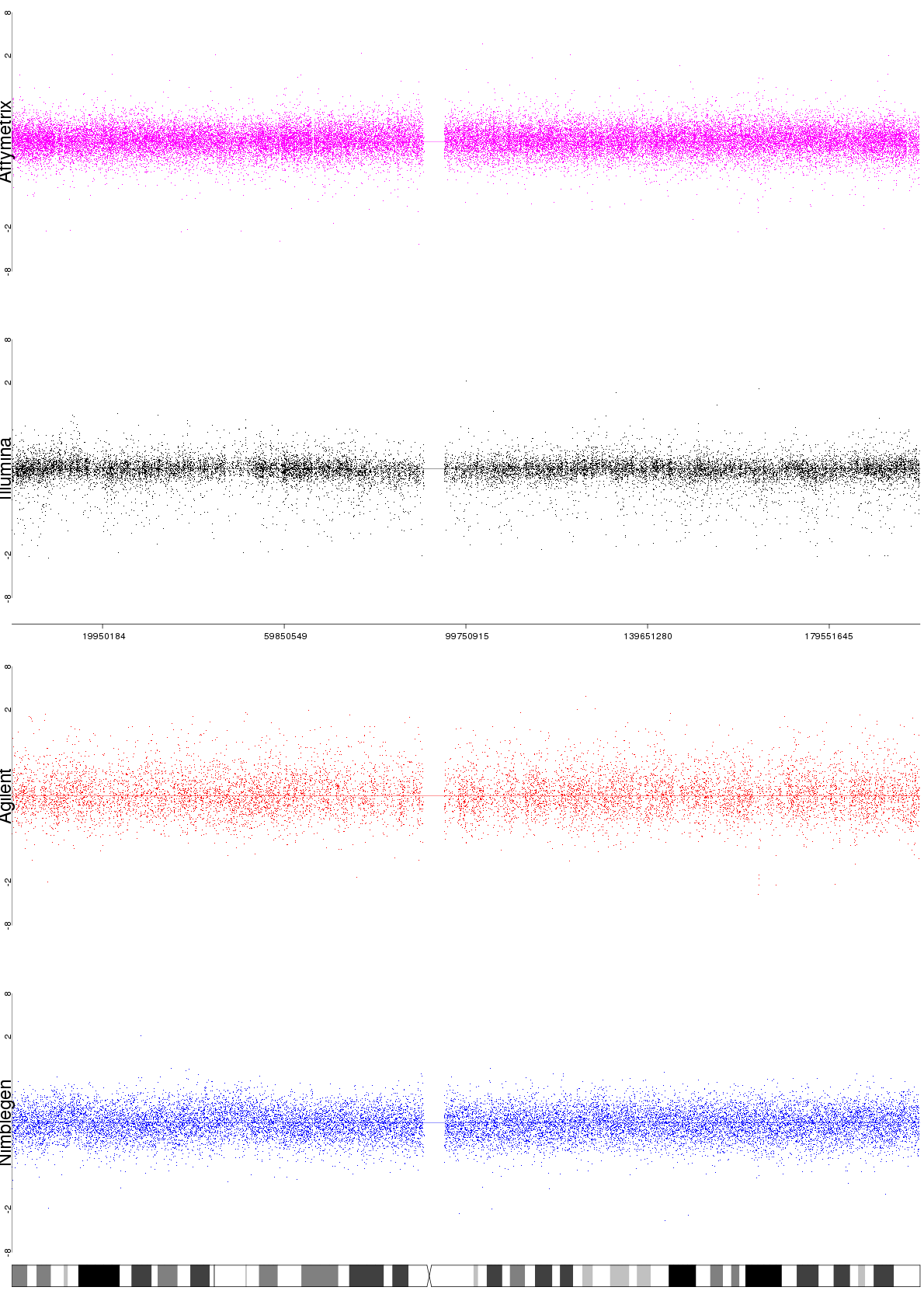

Supplement: Additional file 12 — All sample/chromosome plots for the tumours. Zip folder containing PNGs of all whole-chromosome plots for the tumours. [file 1471-2164-10-588-S12.ZIP › T7207/T7207 chromosome 3.png]

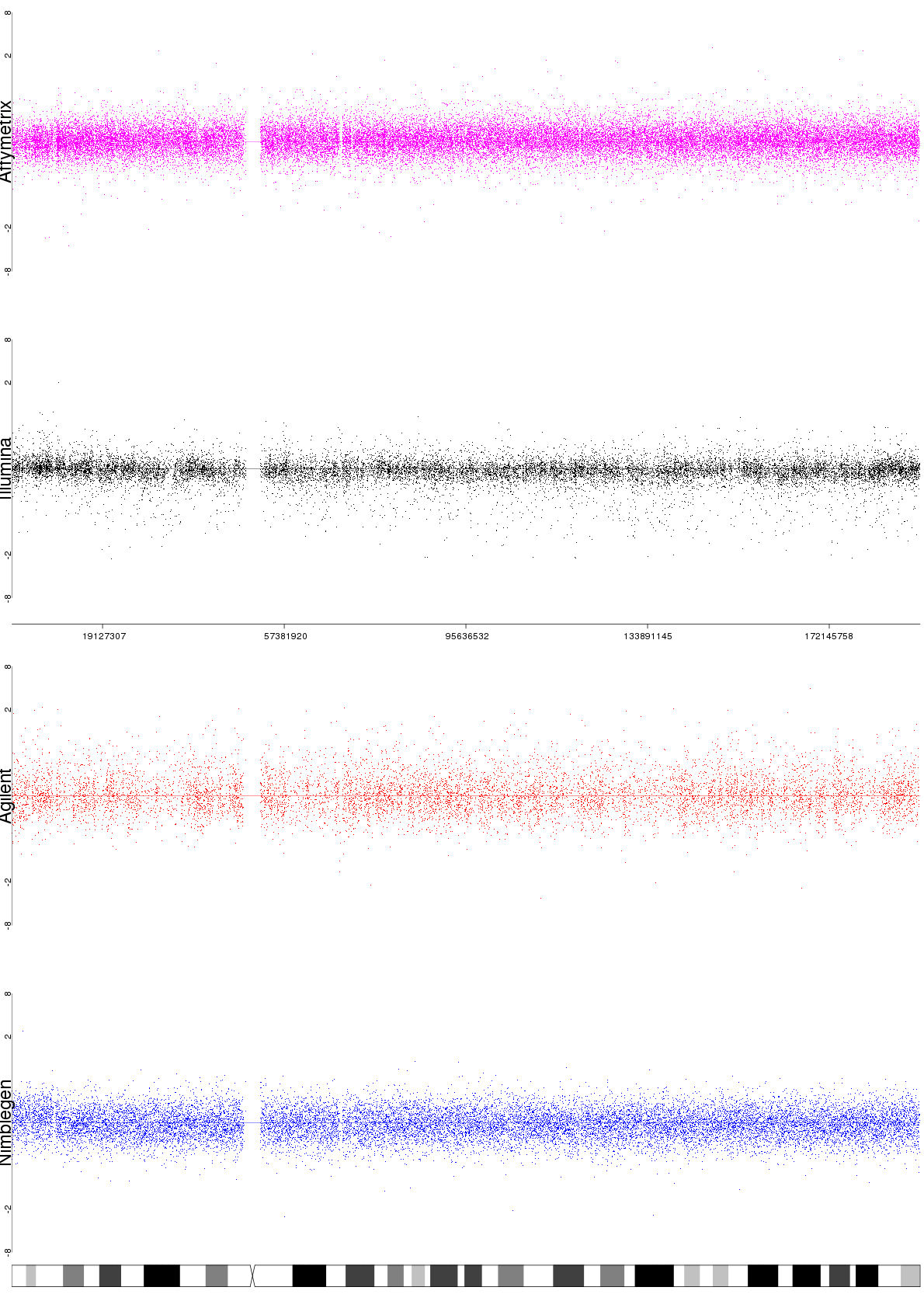

Supplement: Additional file 12 — All sample/chromosome plots for the tumours. Zip folder containing PNGs of all whole-chromosome plots for the tumours. [file 1471-2164-10-588-S12.ZIP › T7207/T7207 chromosome 4.png]

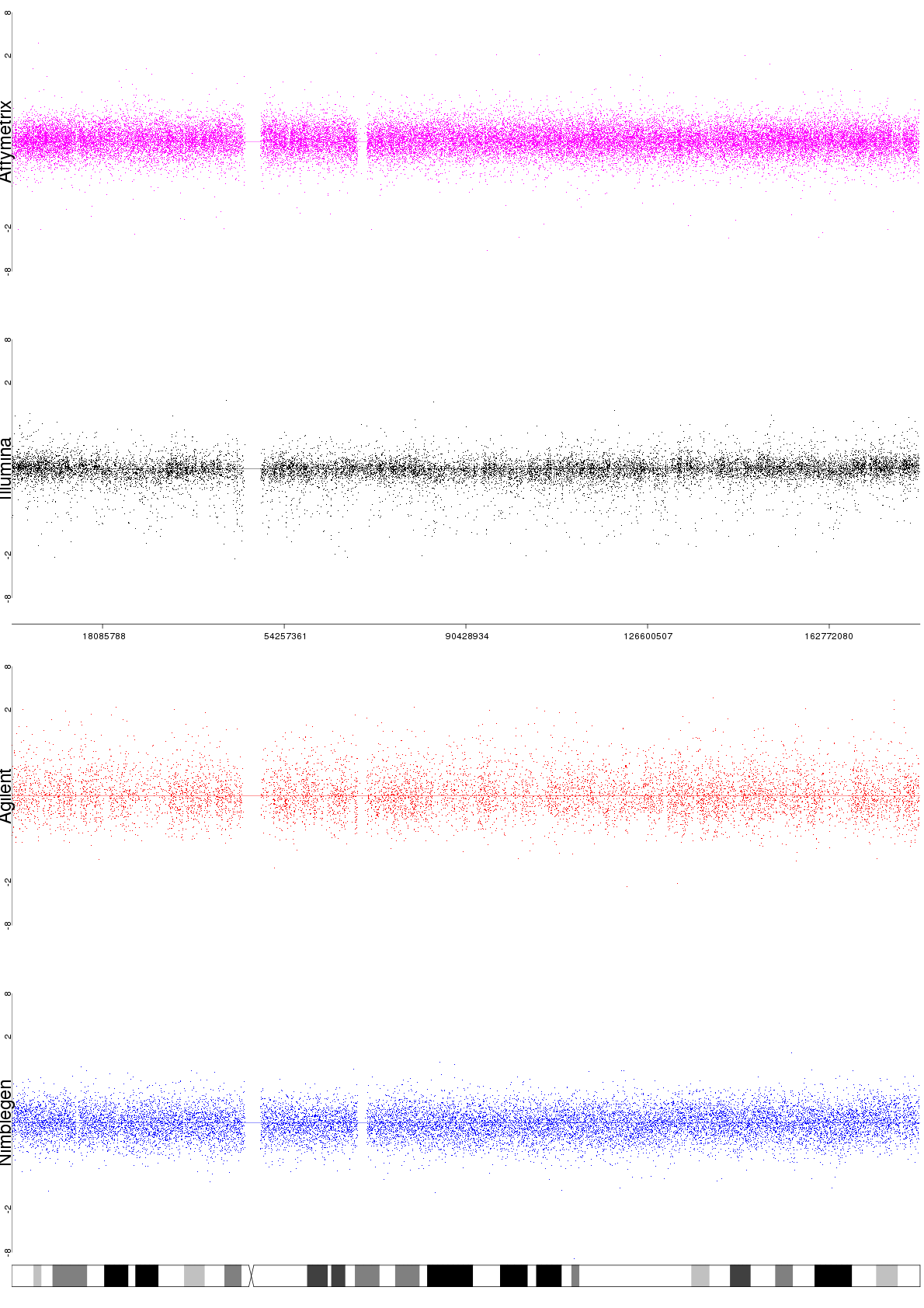

Supplement: Additional file 12 — All sample/chromosome plots for the tumours. Zip folder containing PNGs of all whole-chromosome plots for the tumours. [file 1471-2164-10-588-S12.ZIP › T7207/T7207 chromosome 5.png]

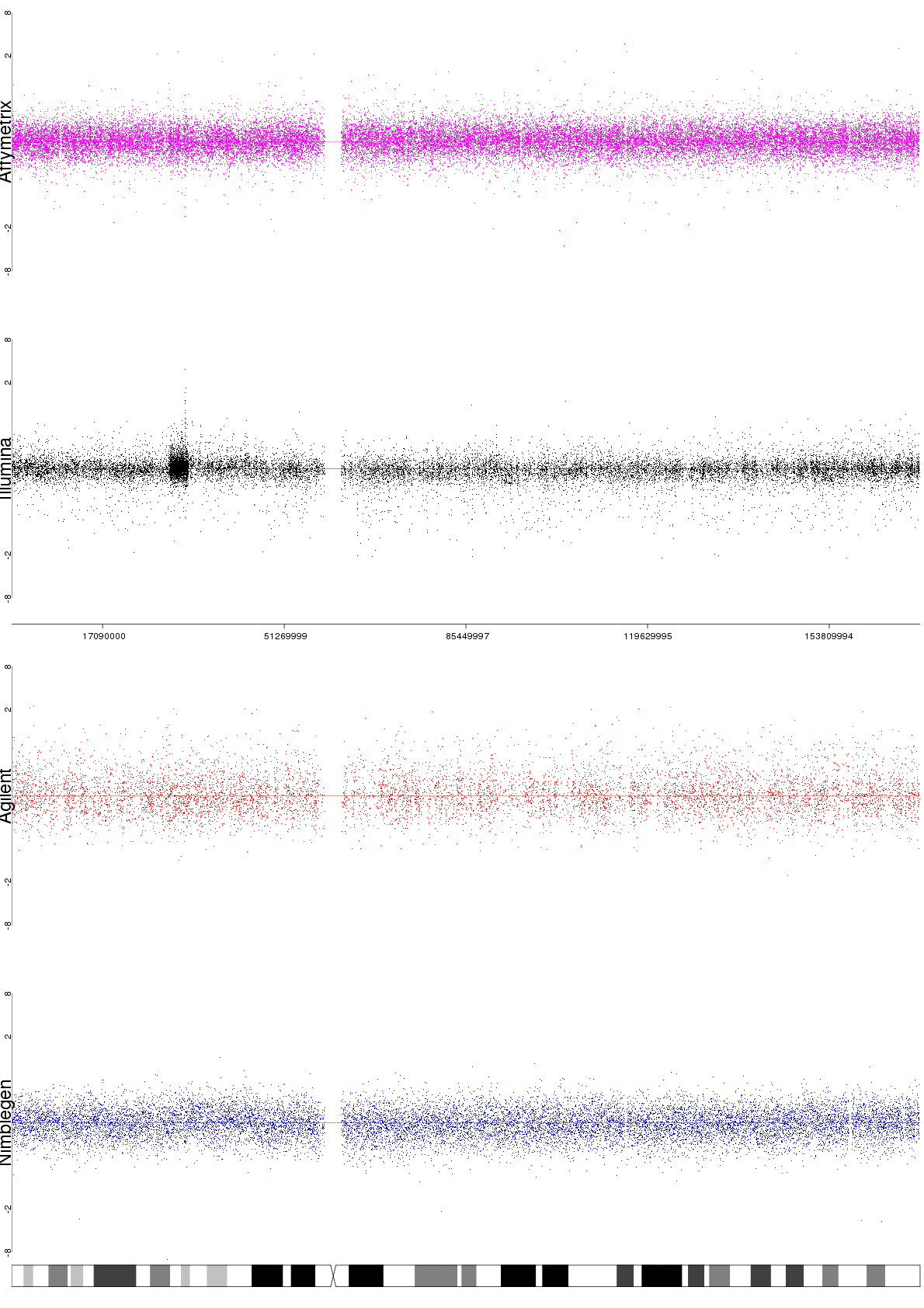

Supplement: Additional file 12 — All sample/chromosome plots for the tumours. Zip folder containing PNGs of all whole-chromosome plots for the tumours. [file 1471-2164-10-588-S12.ZIP › T7207/T7207 chromosome 6.png]

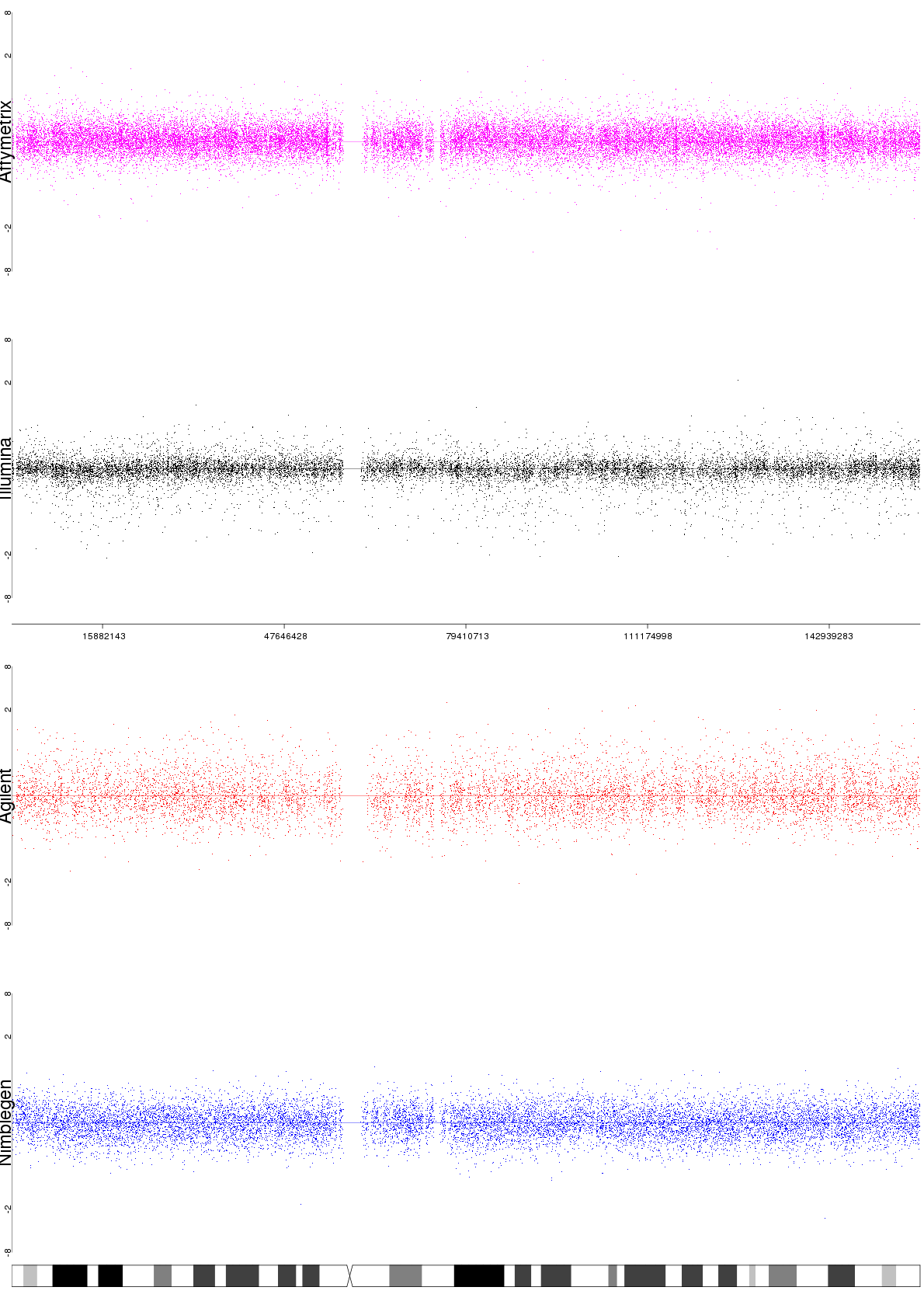

Supplement: Additional file 12 — All sample/chromosome plots for the tumours. Zip folder containing PNGs of all whole-chromosome plots for the tumours. [file 1471-2164-10-588-S12.ZIP › T7207/T7207 chromosome 7.png]

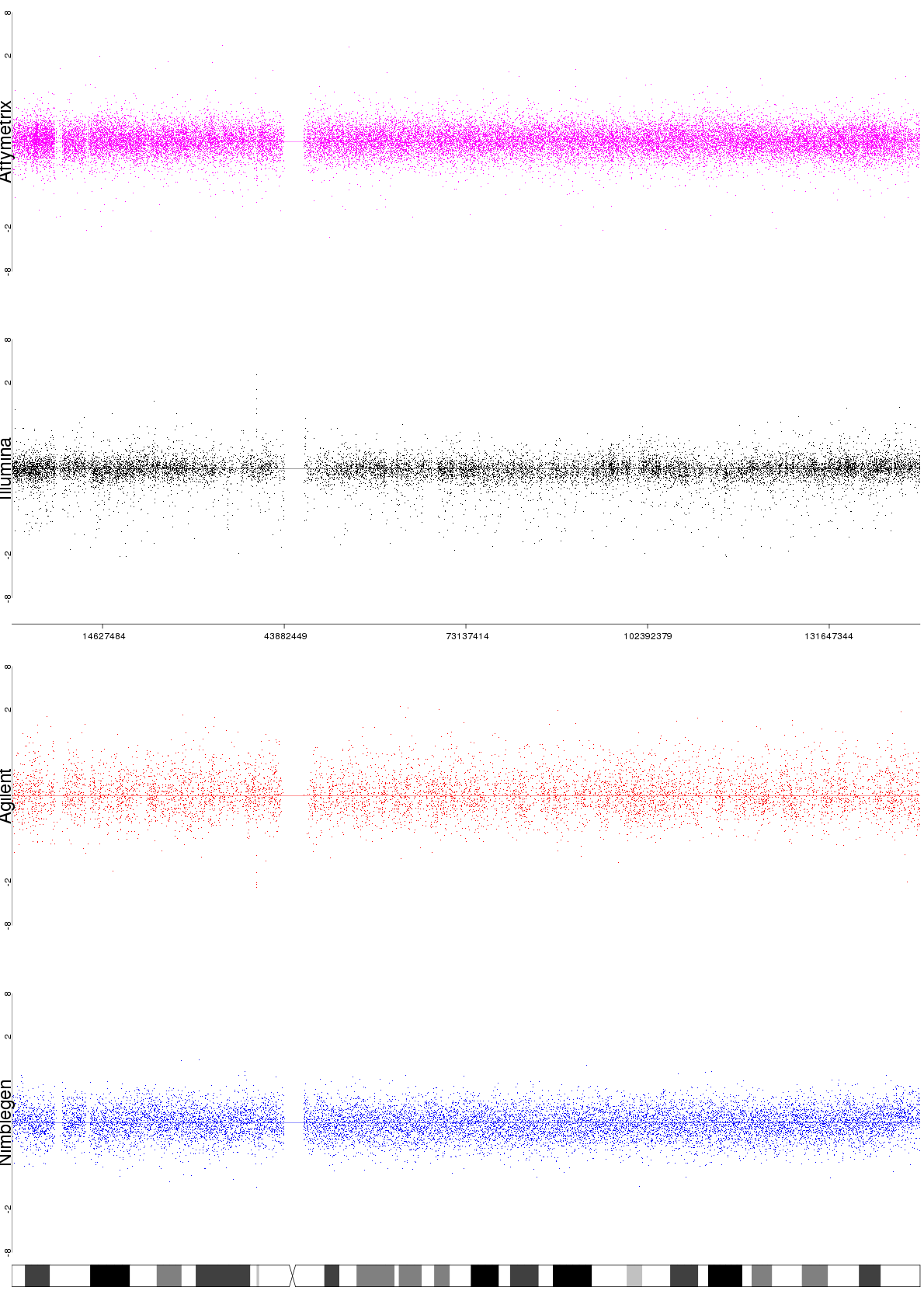

Supplement: Additional file 12 — All sample/chromosome plots for the tumours. Zip folder containing PNGs of all whole-chromosome plots for the tumours. [file 1471-2164-10-588-S12.ZIP › T7207/T7207 chromosome 8.png]

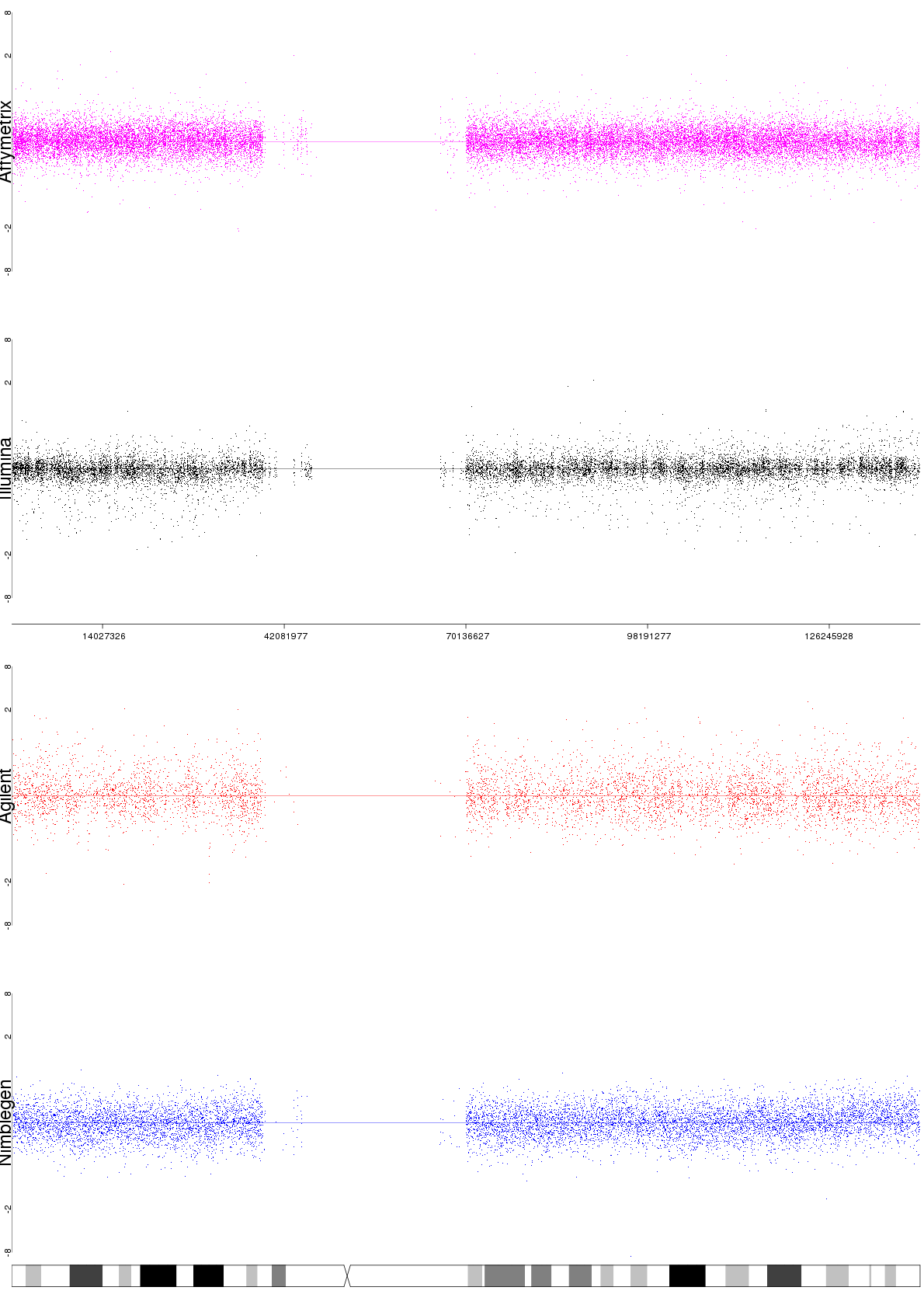

Supplement: Additional file 12 — All sample/chromosome plots for the tumours. Zip folder containing PNGs of all whole-chromosome plots for the tumours. [file 1471-2164-10-588-S12.ZIP › T7207/T7207 chromosome 9.png]

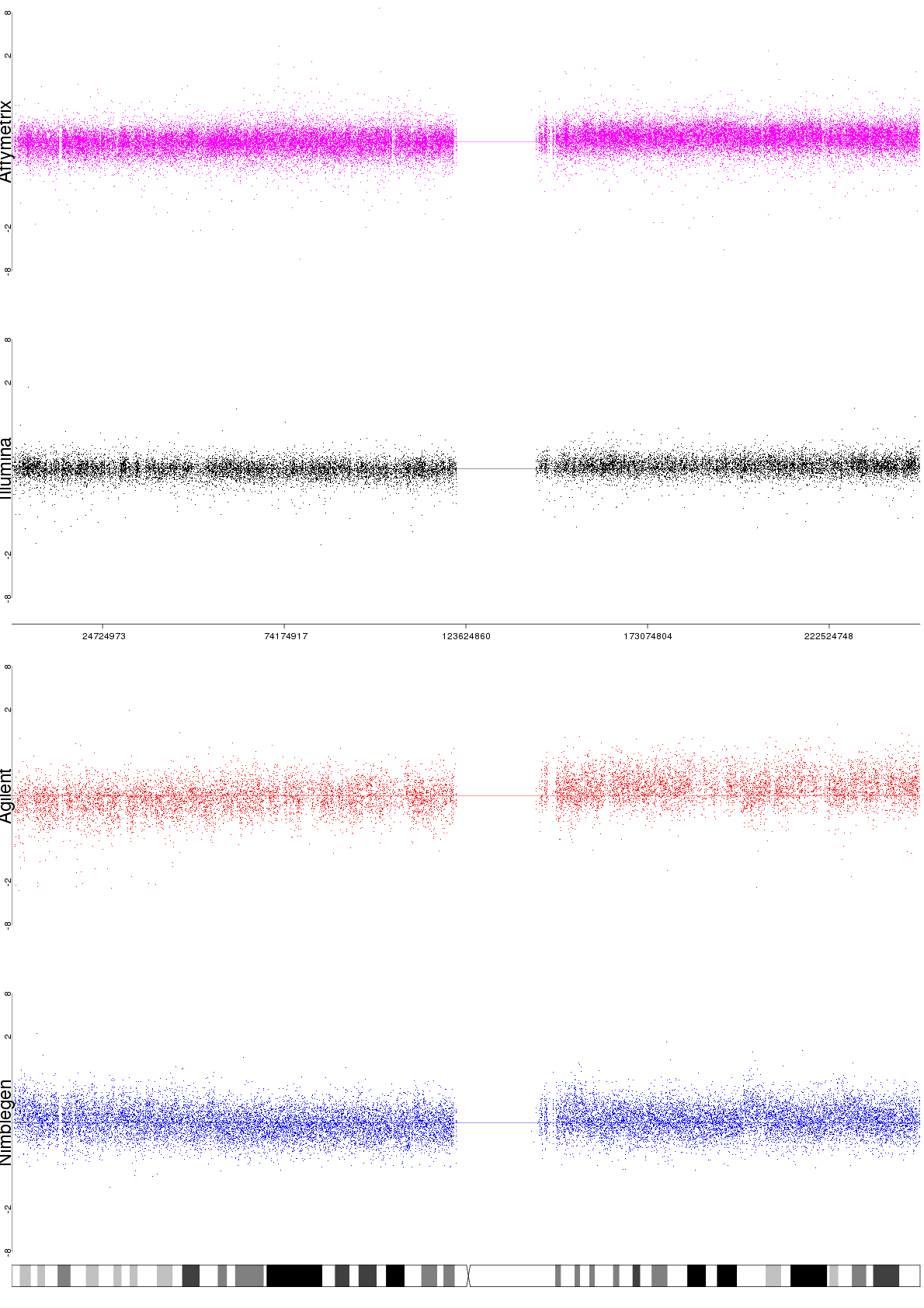

Supplement: Additional file 12 — All sample/chromosome plots for the tumours. Zip folder containing PNGs of all whole-chromosome plots for the tumours. [file 1471-2164-10-588-S12.ZIP › T7214/T7214 chromosome 1.png]

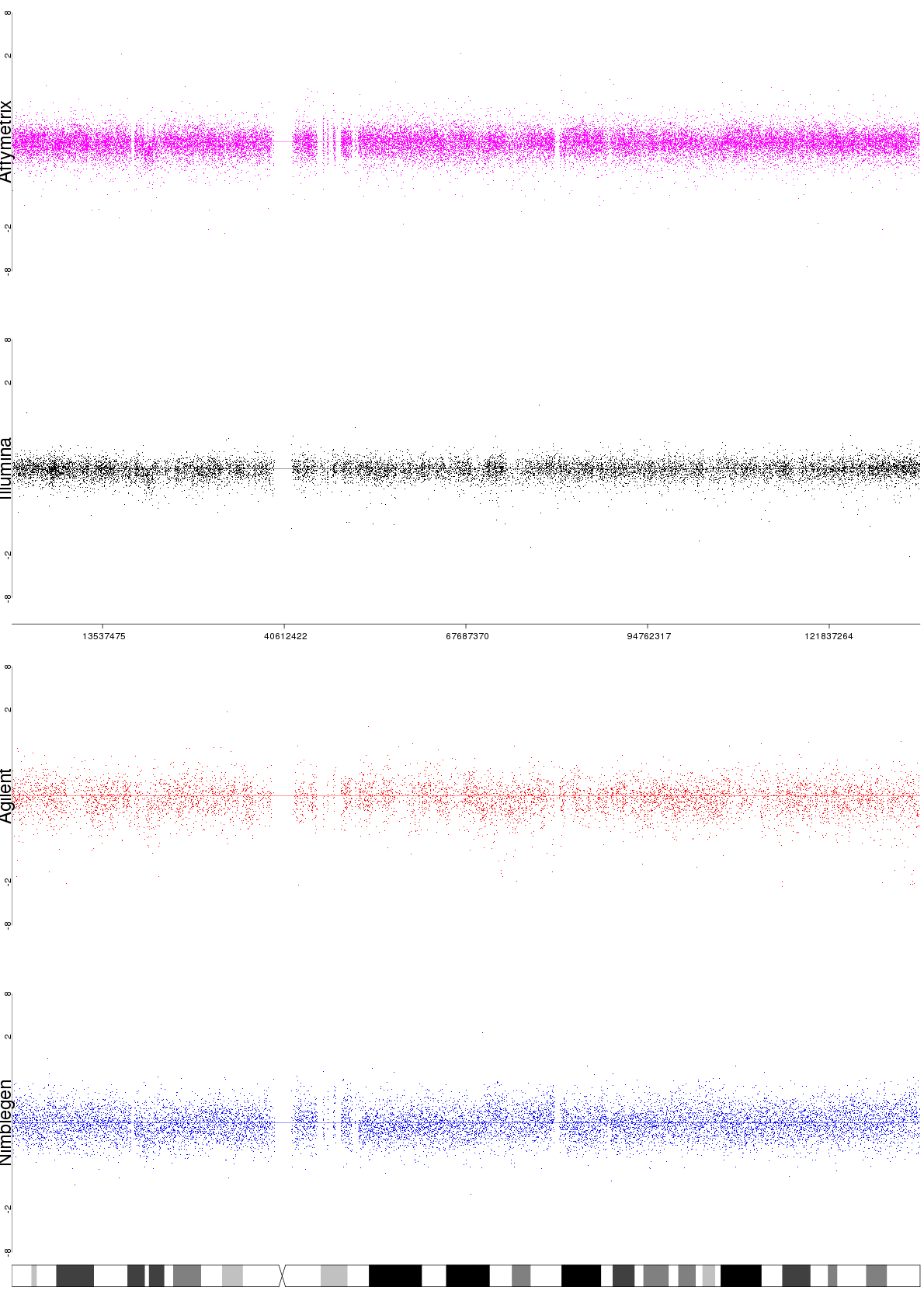

Supplement: Additional file 12 — All sample/chromosome plots for the tumours. Zip folder containing PNGs of all whole-chromosome plots for the tumours. [file 1471-2164-10-588-S12.ZIP › T7214/T7214 chromosome 10.png]

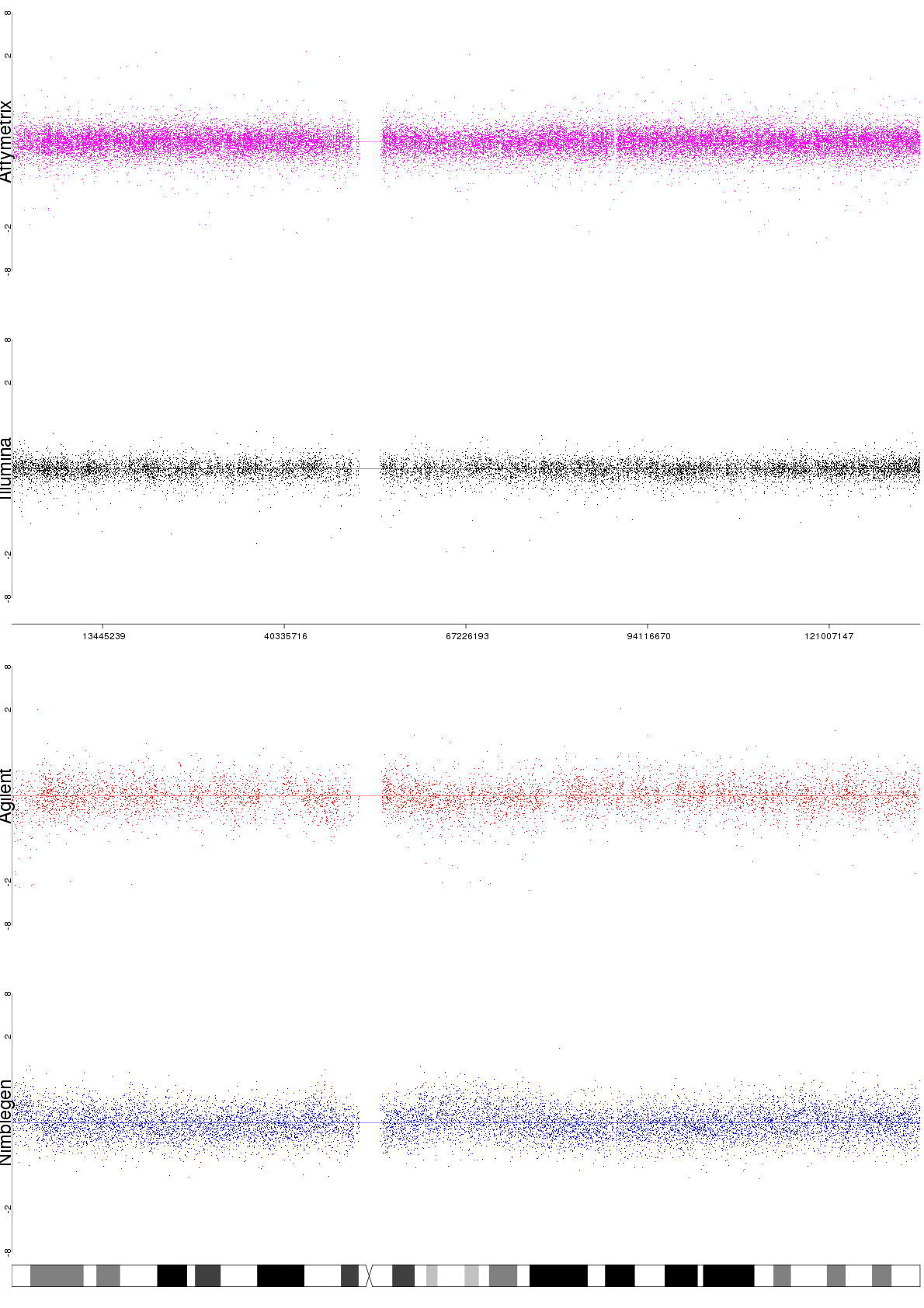

Supplement: Additional file 12 — All sample/chromosome plots for the tumours. Zip folder containing PNGs of all whole-chromosome plots for the tumours. [file 1471-2164-10-588-S12.ZIP › T7214/T7214 chromosome 11.png]

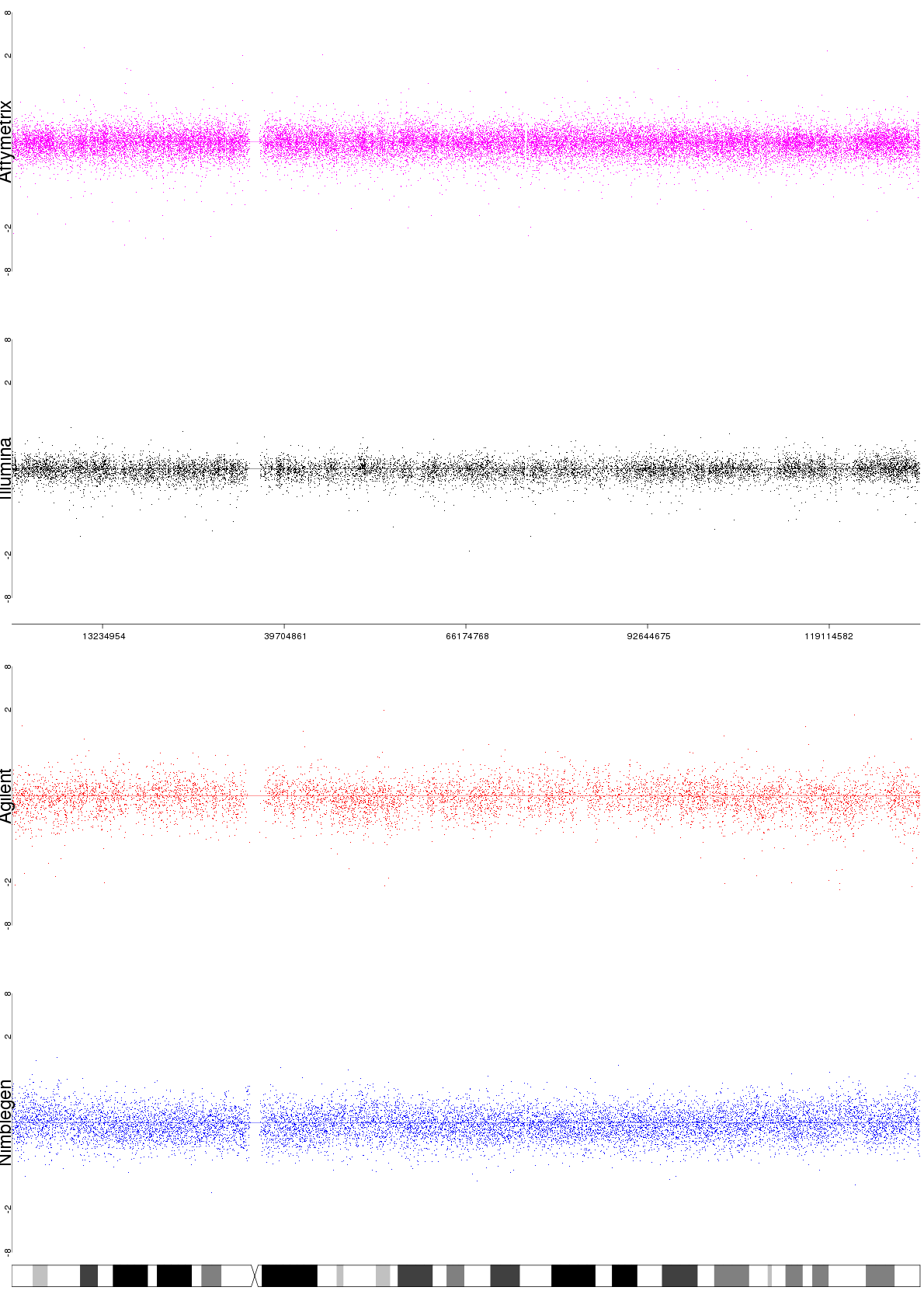

Supplement: Additional file 12 — All sample/chromosome plots for the tumours. Zip folder containing PNGs of all whole-chromosome plots for the tumours. [file 1471-2164-10-588-S12.ZIP › T7214/T7214 chromosome 12.png]

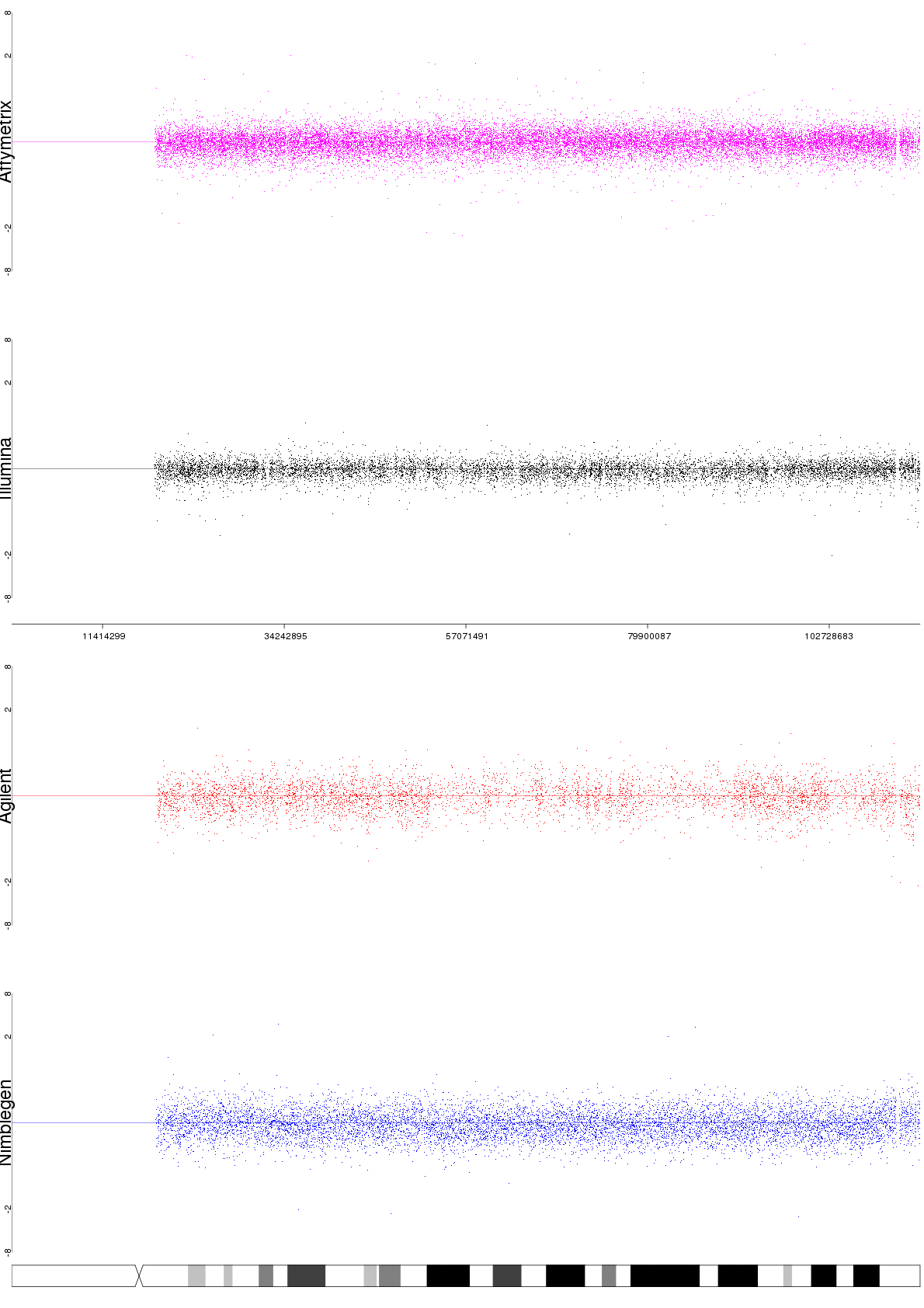

Supplement: Additional file 12 — All sample/chromosome plots for the tumours. Zip folder containing PNGs of all whole-chromosome plots for the tumours. [file 1471-2164-10-588-S12.ZIP › T7214/T7214 chromosome 13.png]

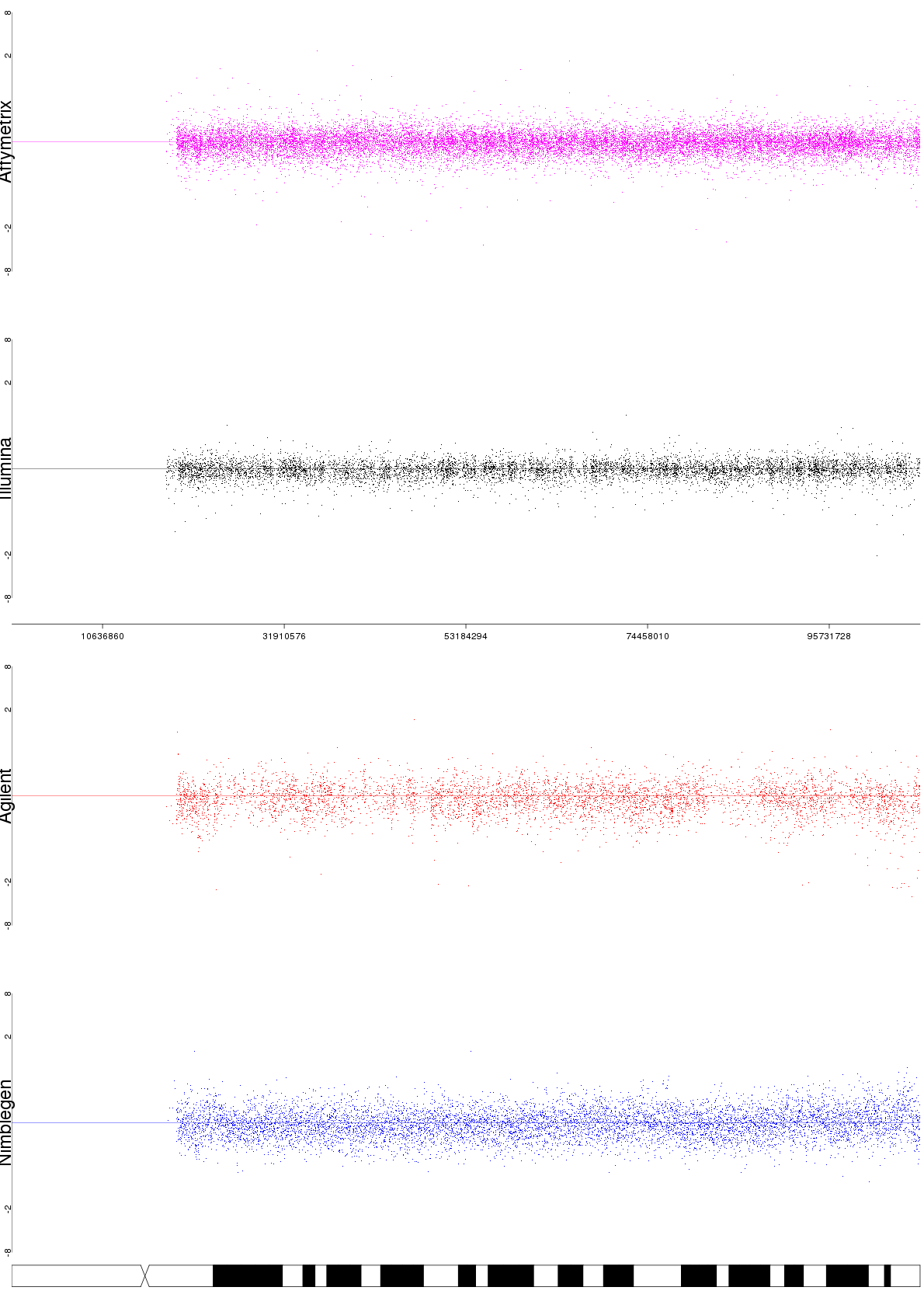

Supplement: Additional file 12 — All sample/chromosome plots for the tumours. Zip folder containing PNGs of all whole-chromosome plots for the tumours. [file 1471-2164-10-588-S12.ZIP › T7214/T7214 chromosome 14.png]

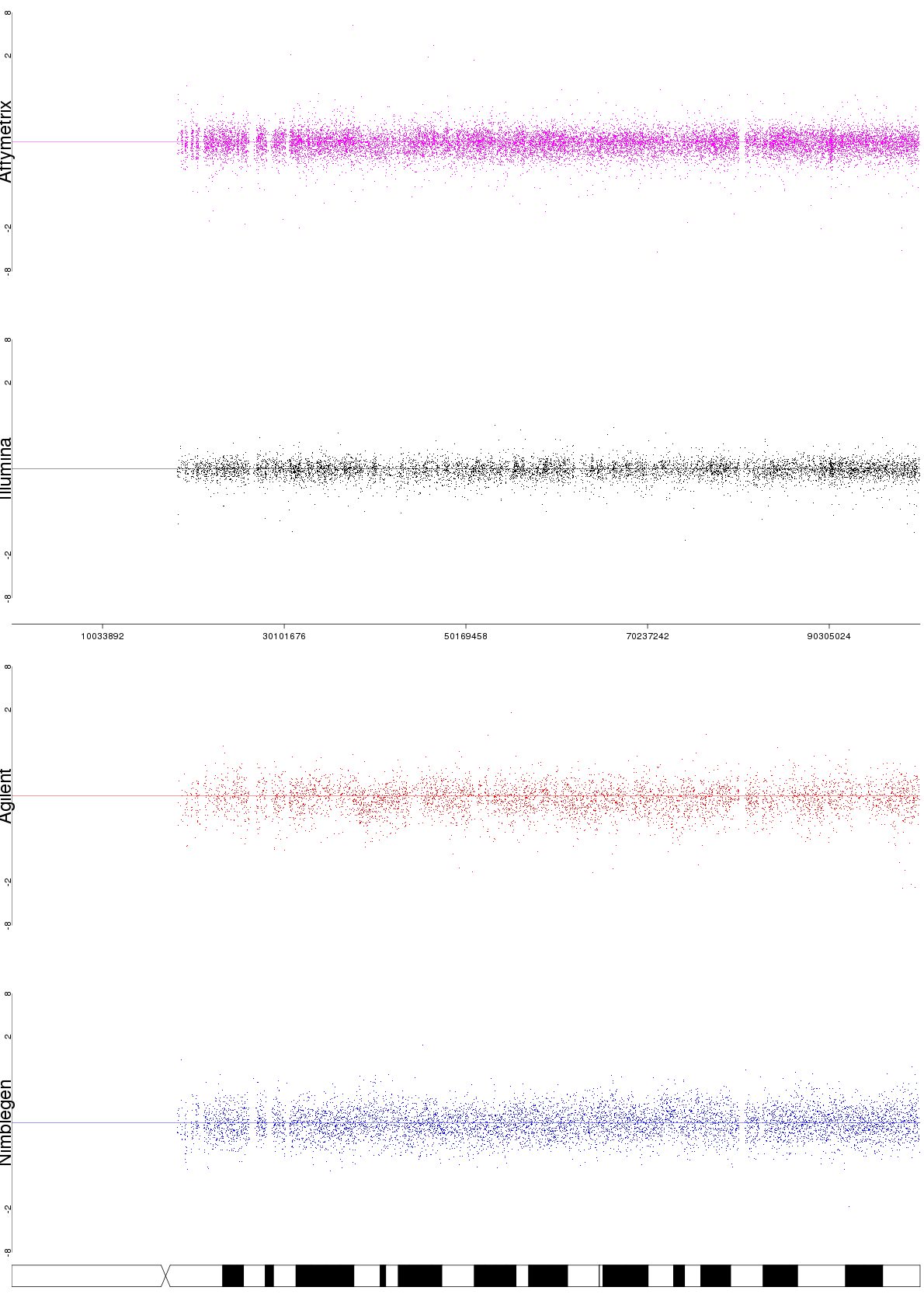

Supplement: Additional file 12 — All sample/chromosome plots for the tumours. Zip folder containing PNGs of all whole-chromosome plots for the tumours. [file 1471-2164-10-588-S12.ZIP › T7214/T7214 chromosome 15.png]

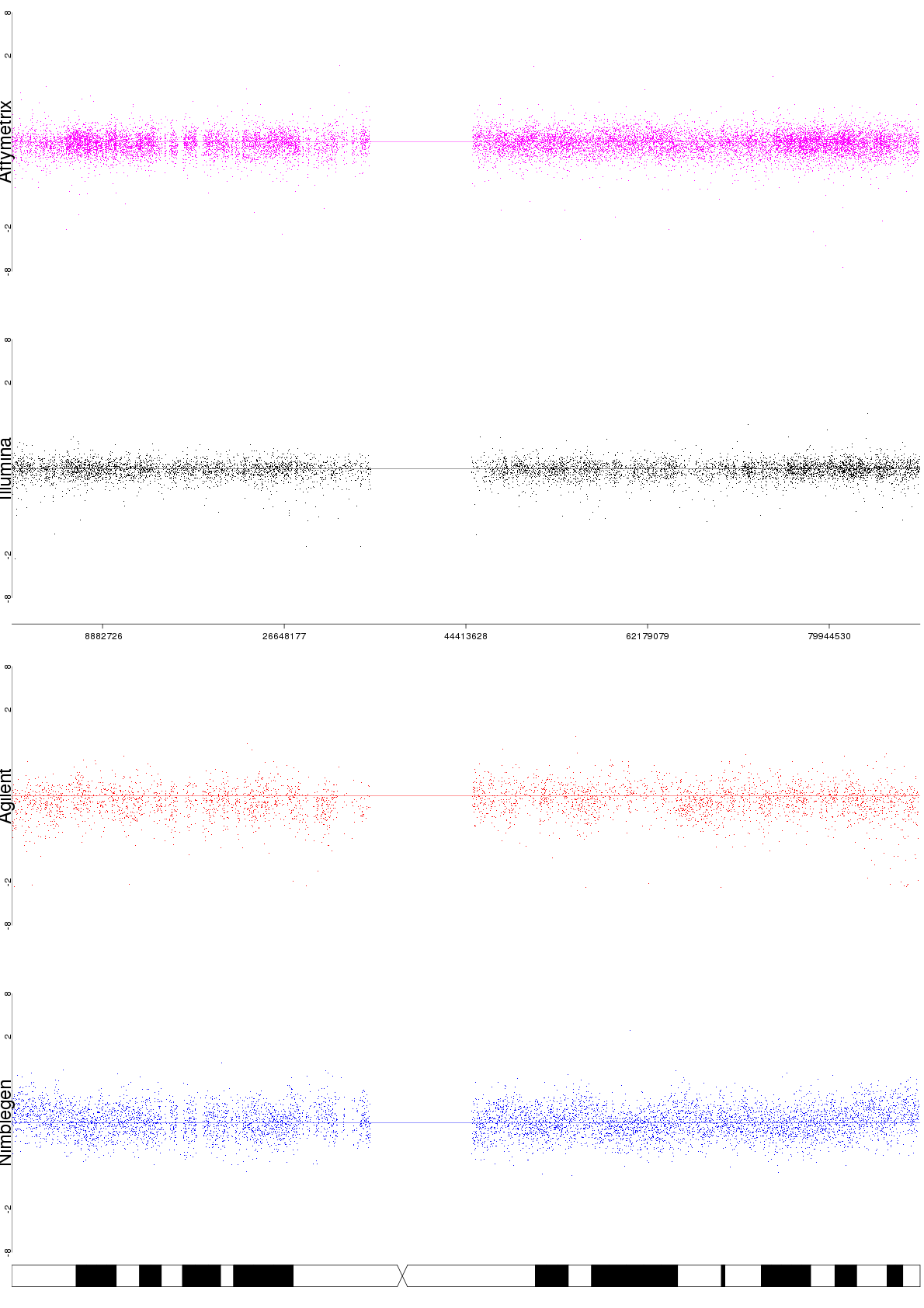

Supplement: Additional file 12 — All sample/chromosome plots for the tumours. Zip folder containing PNGs of all whole-chromosome plots for the tumours. [file 1471-2164-10-588-S12.ZIP › T7214/T7214 chromosome 16.png]

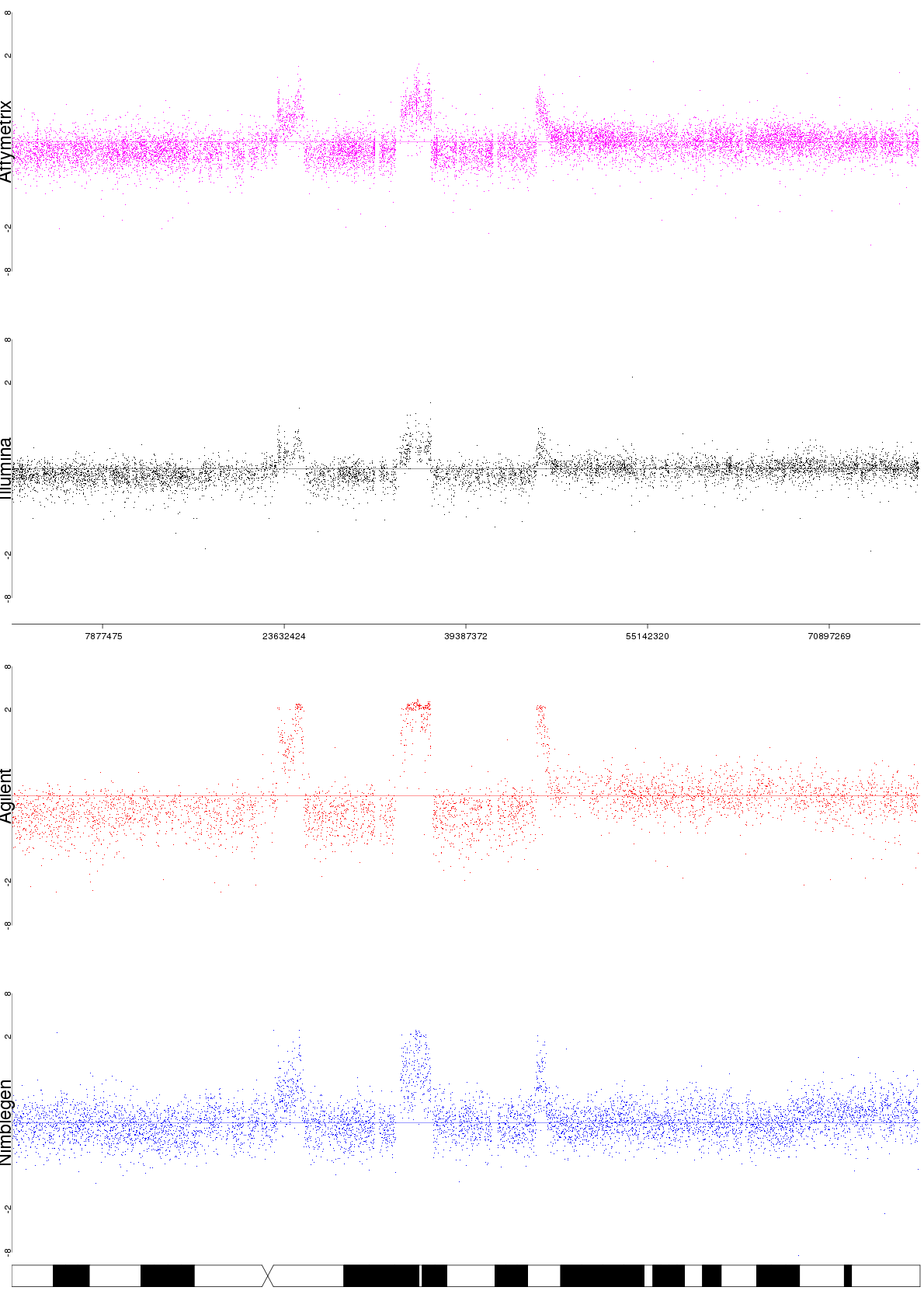

Supplement: Additional file 12 — All sample/chromosome plots for the tumours. Zip folder containing PNGs of all whole-chromosome plots for the tumours. [file 1471-2164-10-588-S12.ZIP › T7214/T7214 chromosome 17.png]

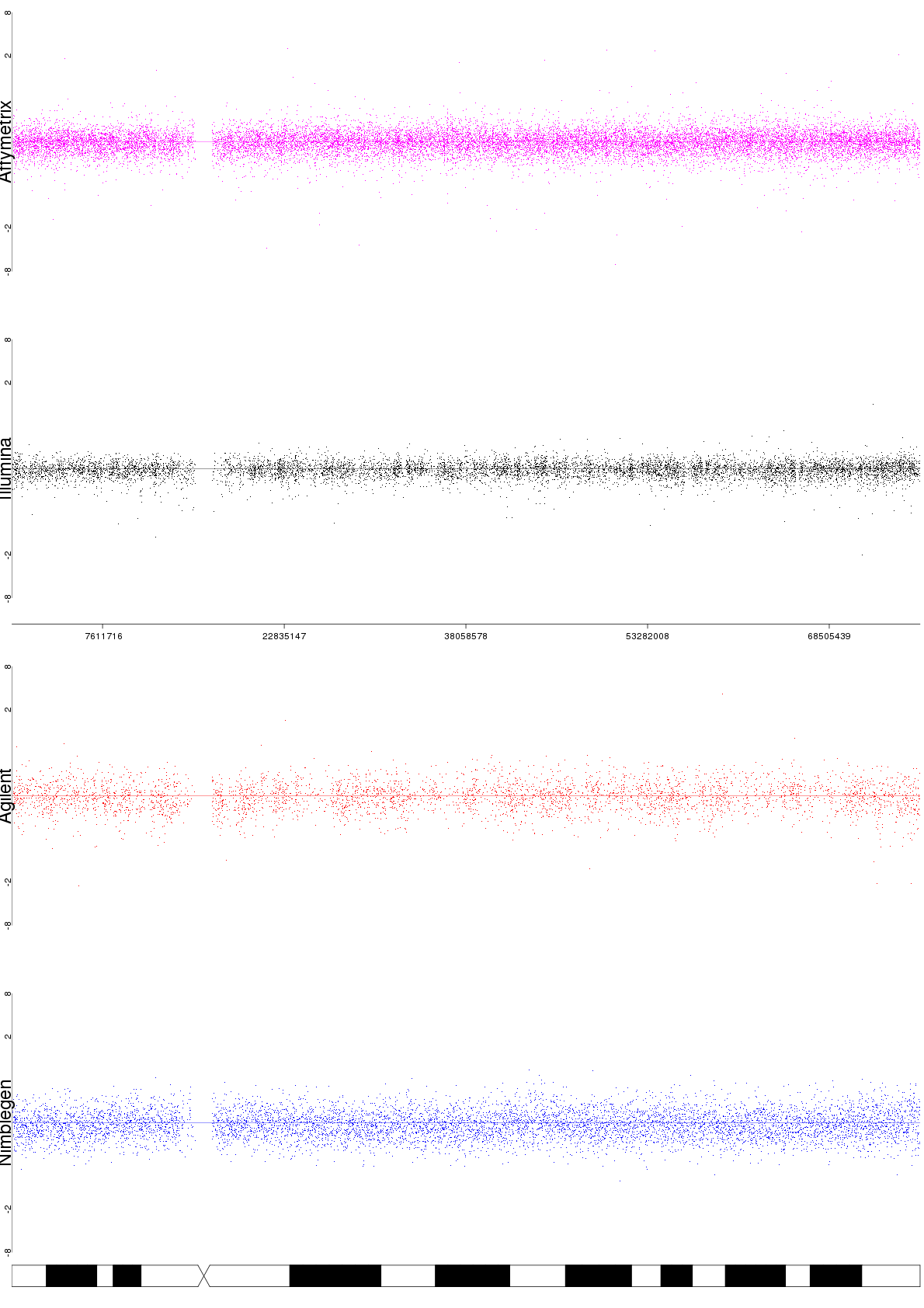

Supplement: Additional file 12 — All sample/chromosome plots for the tumours. Zip folder containing PNGs of all whole-chromosome plots for the tumours. [file 1471-2164-10-588-S12.ZIP › T7214/T7214 chromosome 18.png]

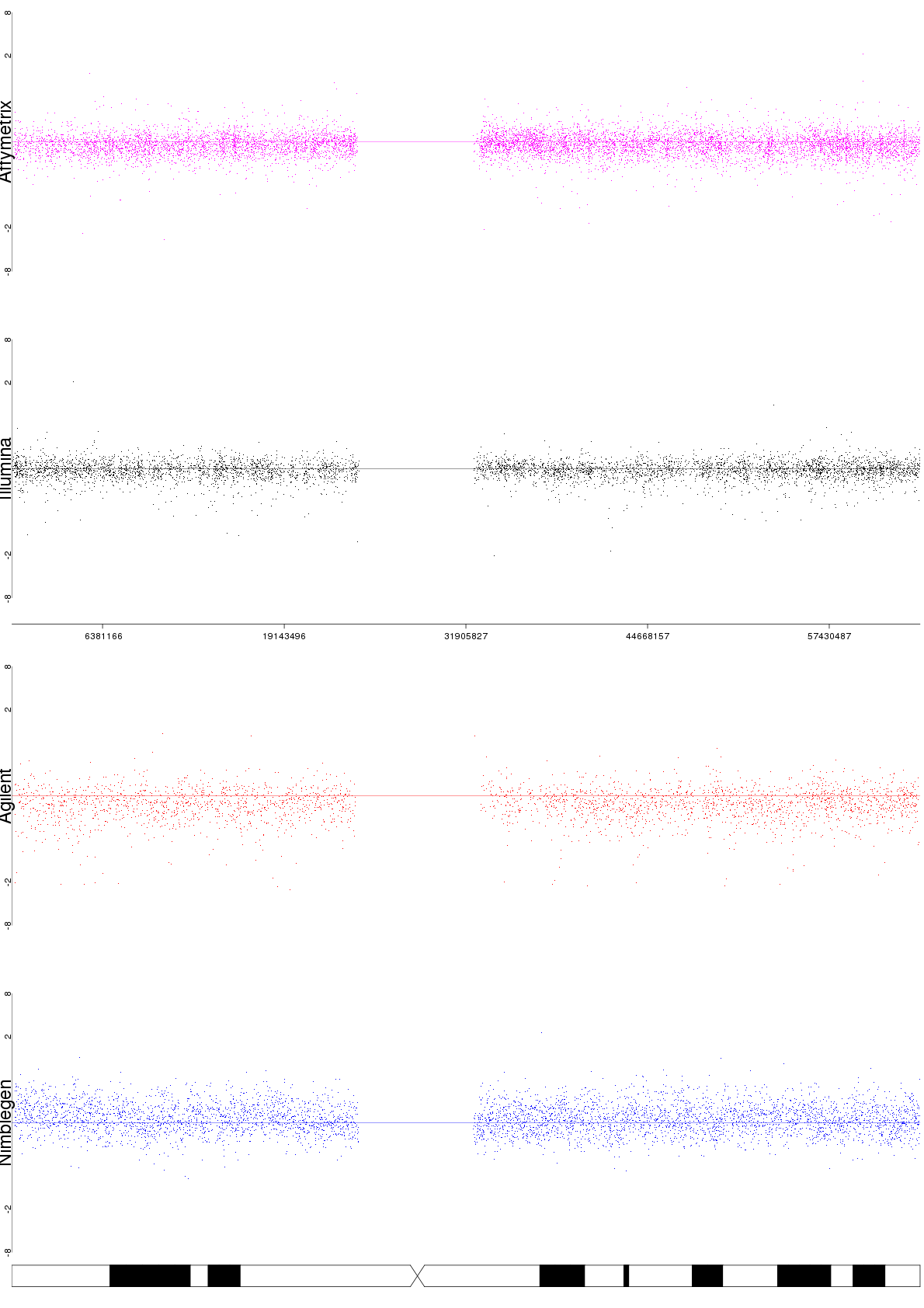

Supplement: Additional file 12 — All sample/chromosome plots for the tumours. Zip folder containing PNGs of all whole-chromosome plots for the tumours. [file 1471-2164-10-588-S12.ZIP › T7214/T7214 chromosome 19.png]

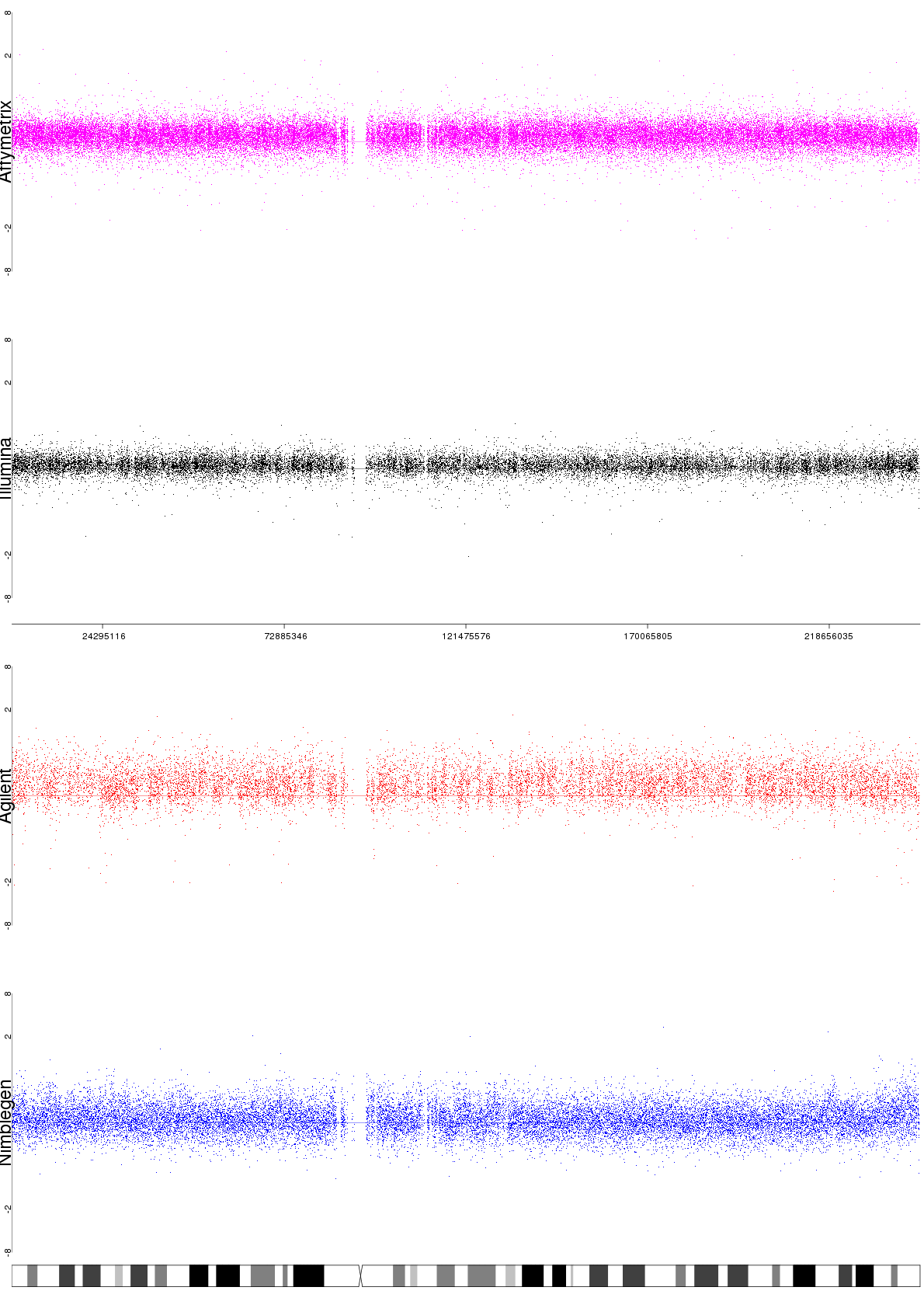

Supplement: Additional file 12 — All sample/chromosome plots for the tumours. Zip folder containing PNGs of all whole-chromosome plots for the tumours. [file 1471-2164-10-588-S12.ZIP › T7214/T7214 chromosome 2.png]

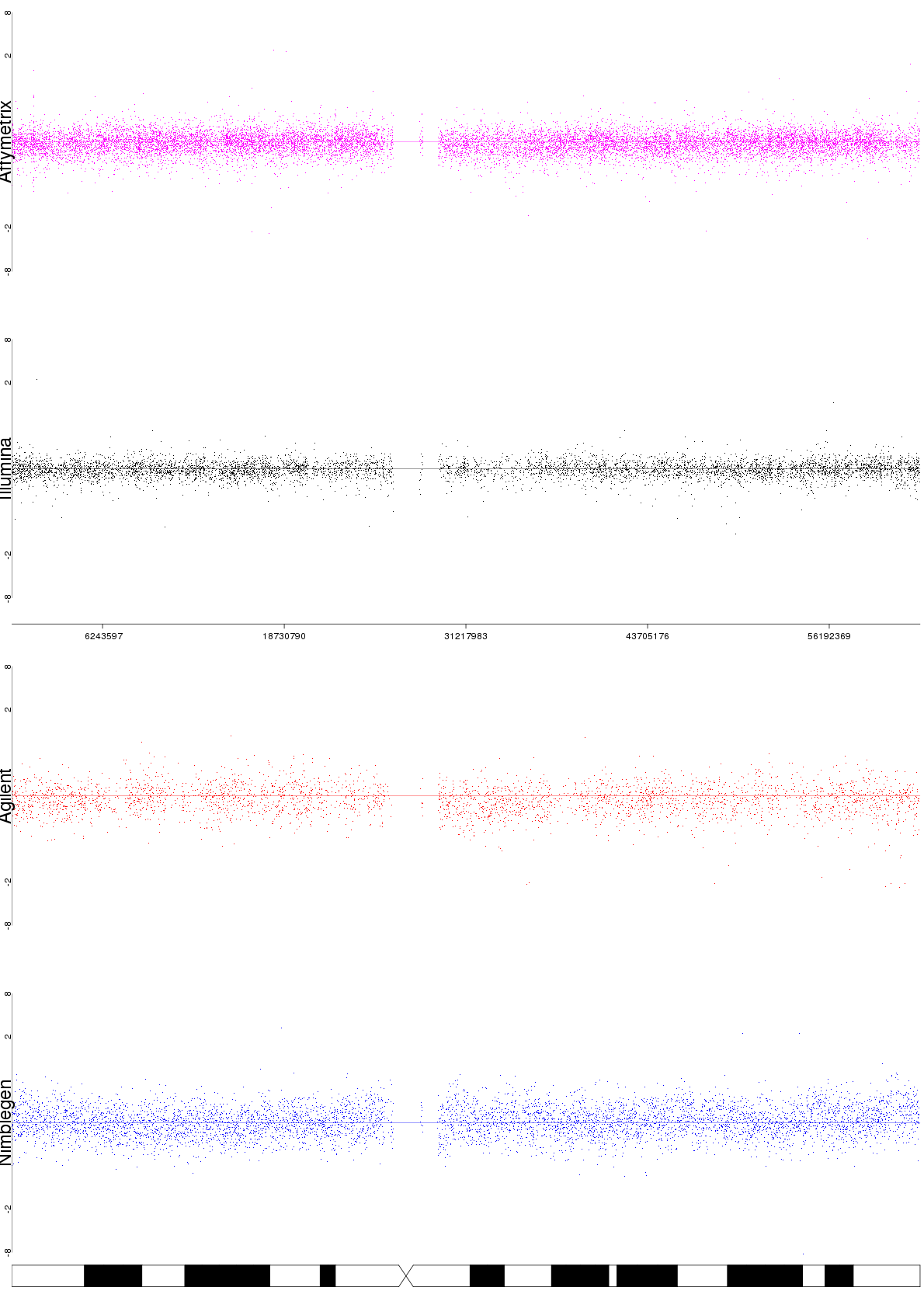

Supplement: Additional file 12 — All sample/chromosome plots for the tumours. Zip folder containing PNGs of all whole-chromosome plots for the tumours. [file 1471-2164-10-588-S12.ZIP › T7214/T7214 chromosome 20.png]

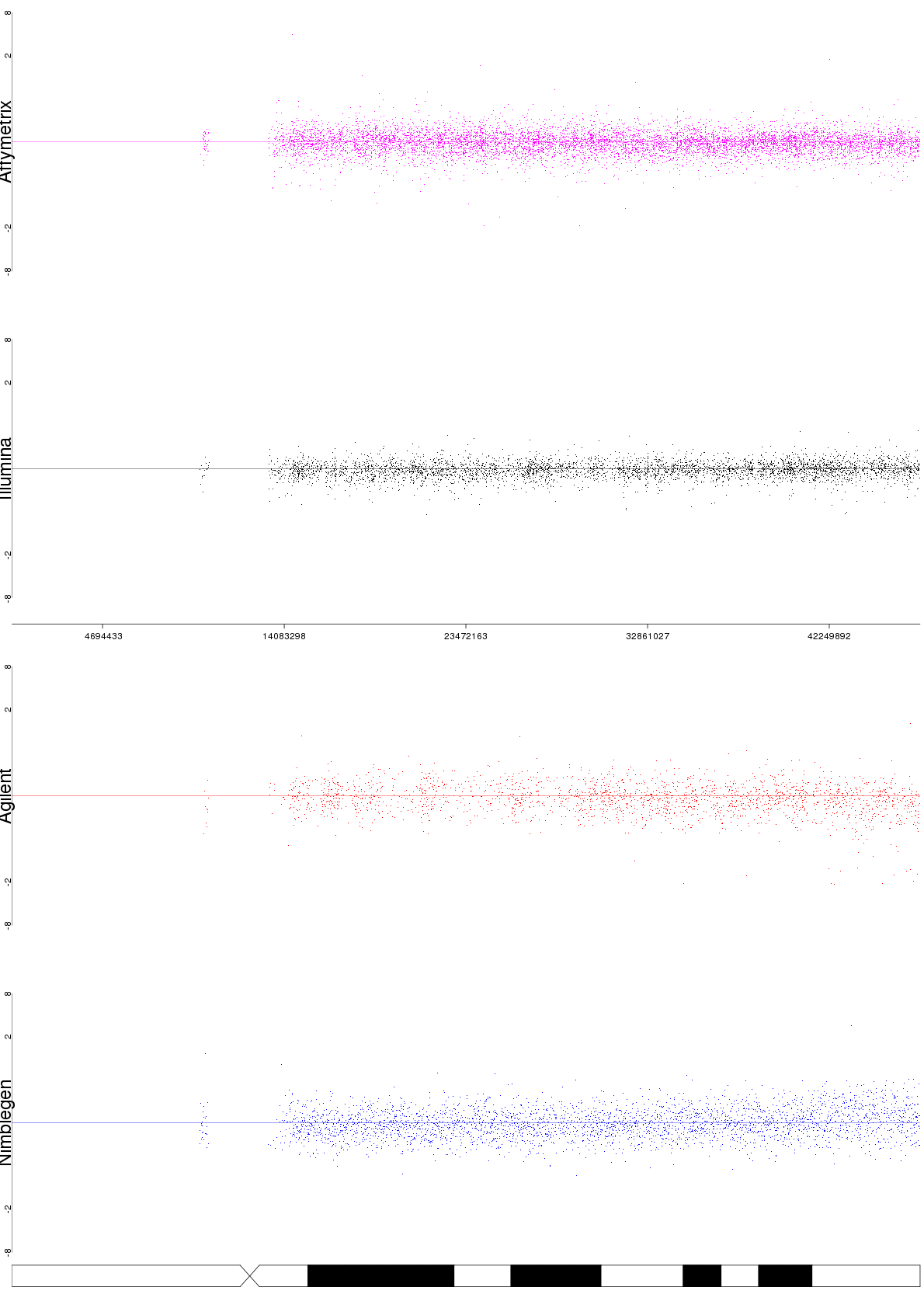

Supplement: Additional file 12 — All sample/chromosome plots for the tumours. Zip folder containing PNGs of all whole-chromosome plots for the tumours. [file 1471-2164-10-588-S12.ZIP › T7214/T7214 chromosome 21.png]

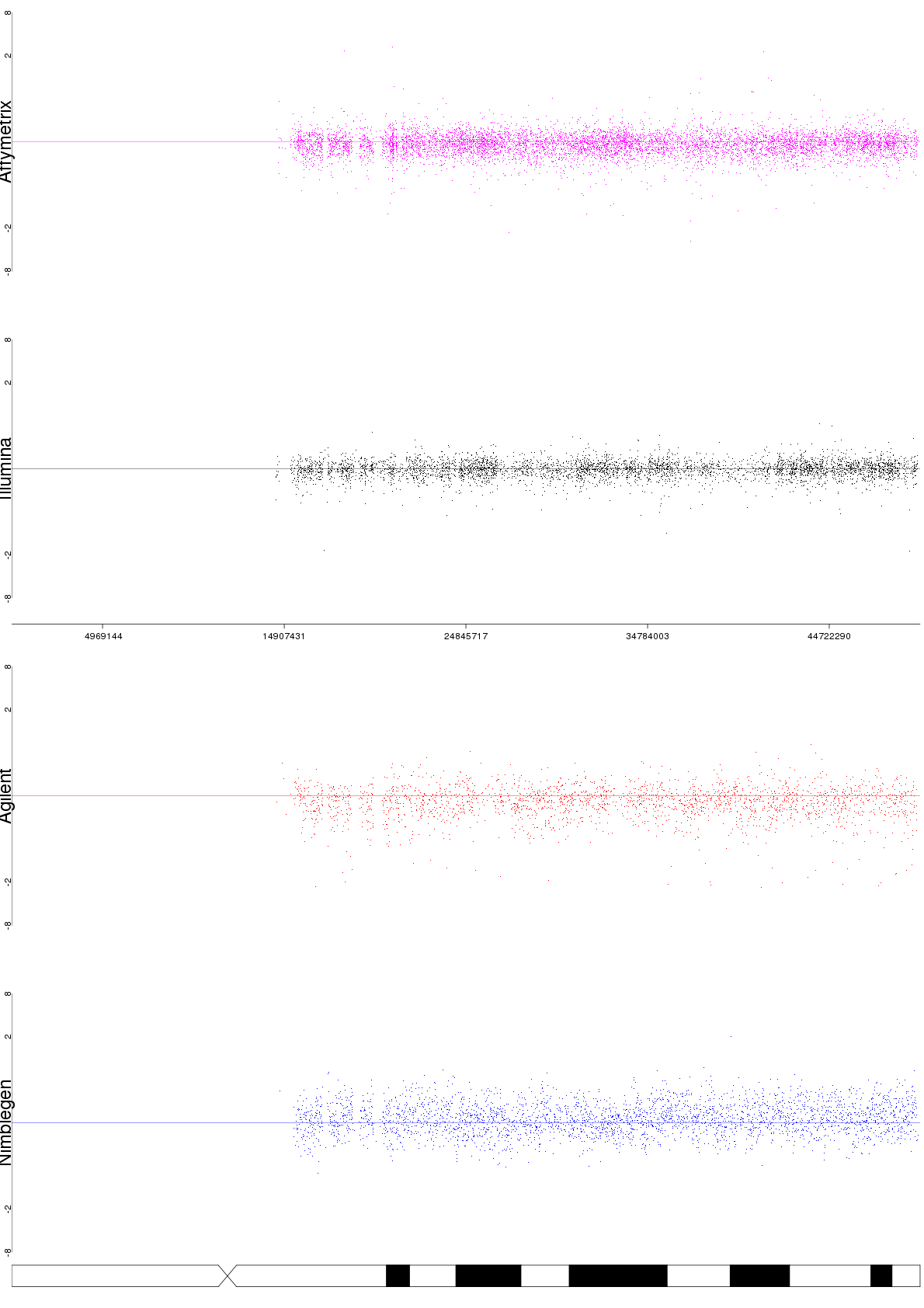

Supplement: Additional file 12 — All sample/chromosome plots for the tumours. Zip folder containing PNGs of all whole-chromosome plots for the tumours. [file 1471-2164-10-588-S12.ZIP › T7214/T7214 chromosome 22.png]

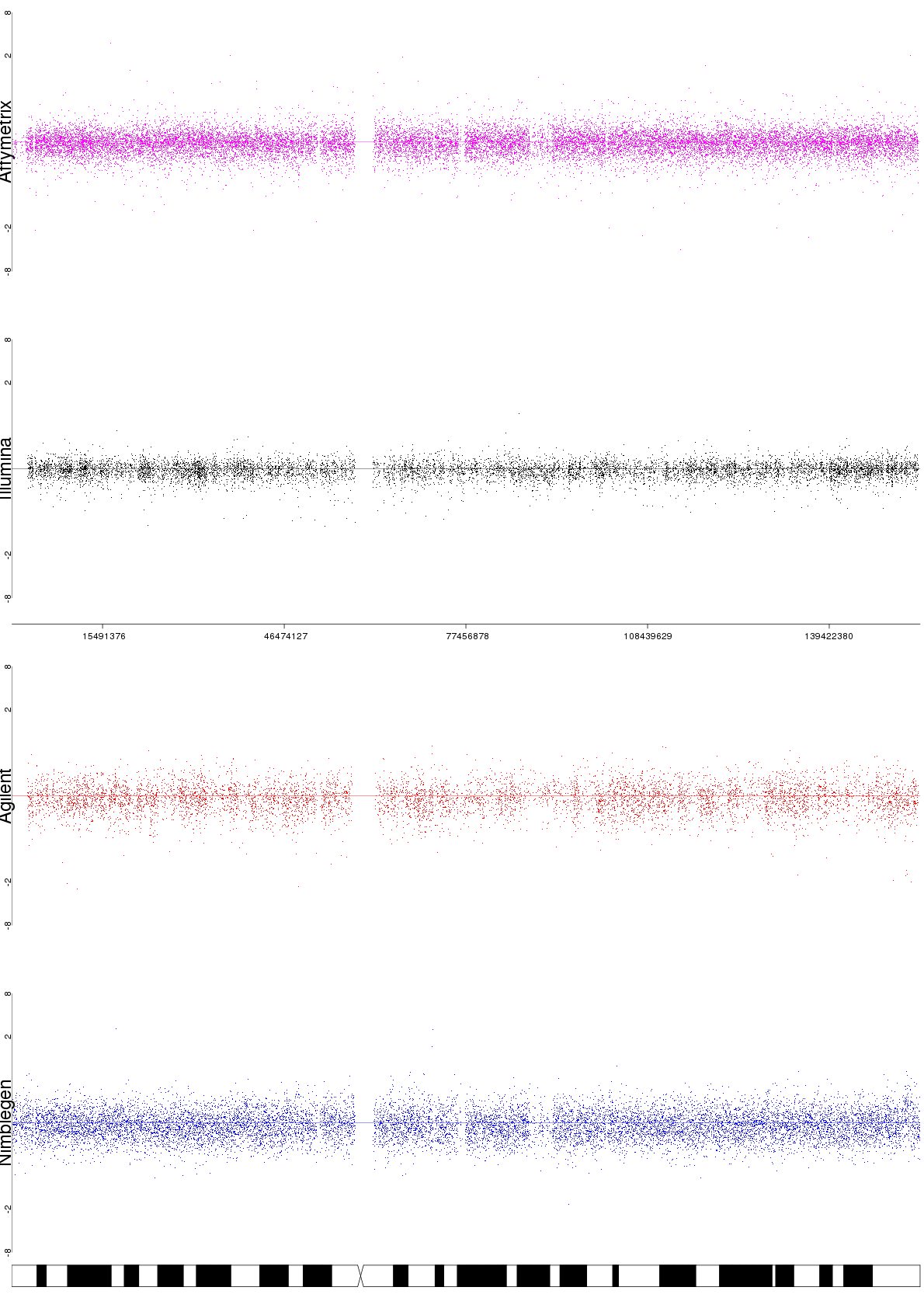

Supplement: Additional file 12 — All sample/chromosome plots for the tumours. Zip folder containing PNGs of all whole-chromosome plots for the tumours. [file 1471-2164-10-588-S12.ZIP › T7214/T7214 chromosome 23.png]

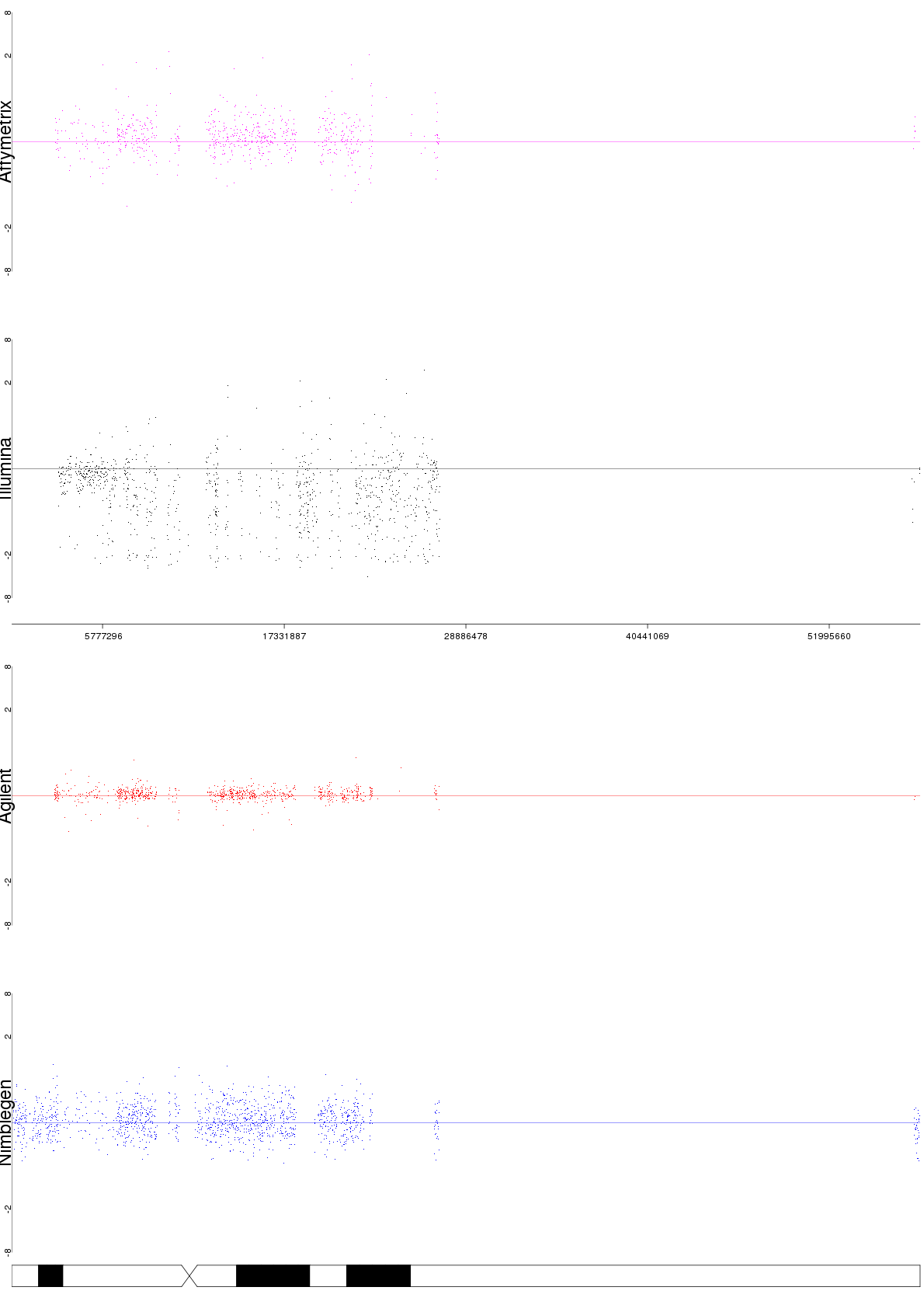

Supplement: Additional file 12 — All sample/chromosome plots for the tumours. Zip folder containing PNGs of all whole-chromosome plots for the tumours. [file 1471-2164-10-588-S12.ZIP › T7214/T7214 chromosome 24.png]

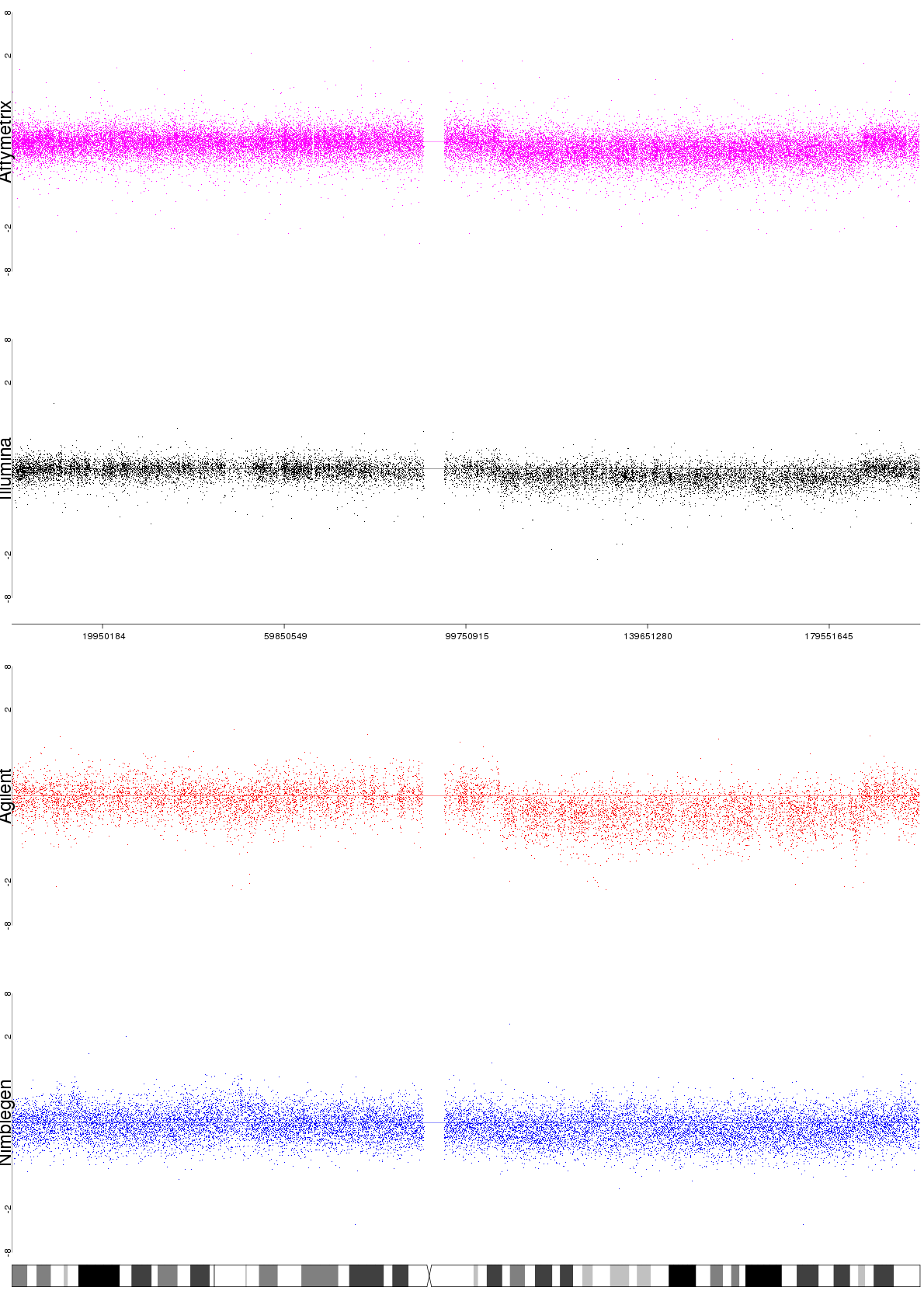

Supplement: Additional file 12 — All sample/chromosome plots for the tumours. Zip folder containing PNGs of all whole-chromosome plots for the tumours. [file 1471-2164-10-588-S12.ZIP › T7214/T7214 chromosome 3.png]

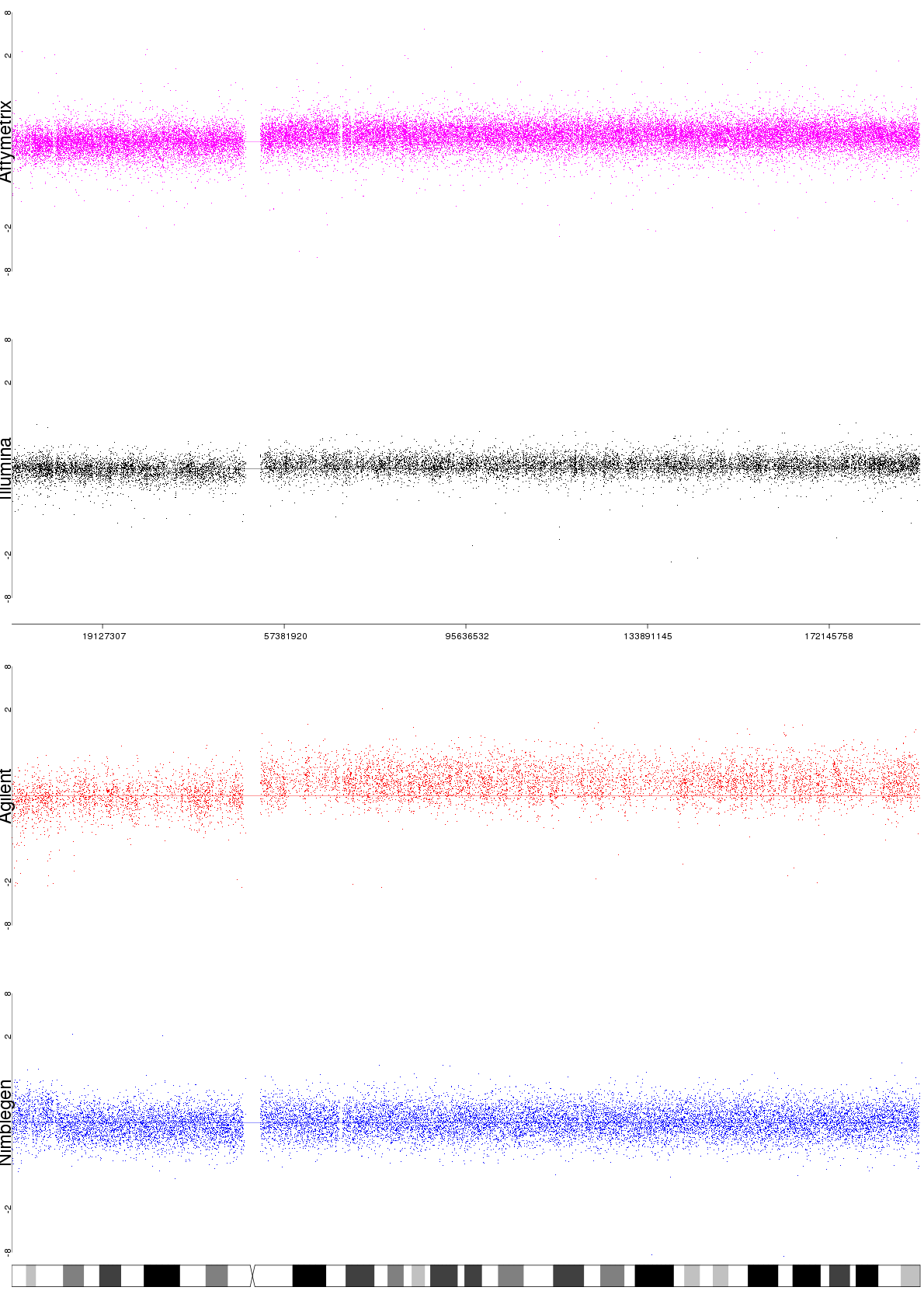

Supplement: Additional file 12 — All sample/chromosome plots for the tumours. Zip folder containing PNGs of all whole-chromosome plots for the tumours. [file 1471-2164-10-588-S12.ZIP › T7214/T7214 chromosome 4.png]

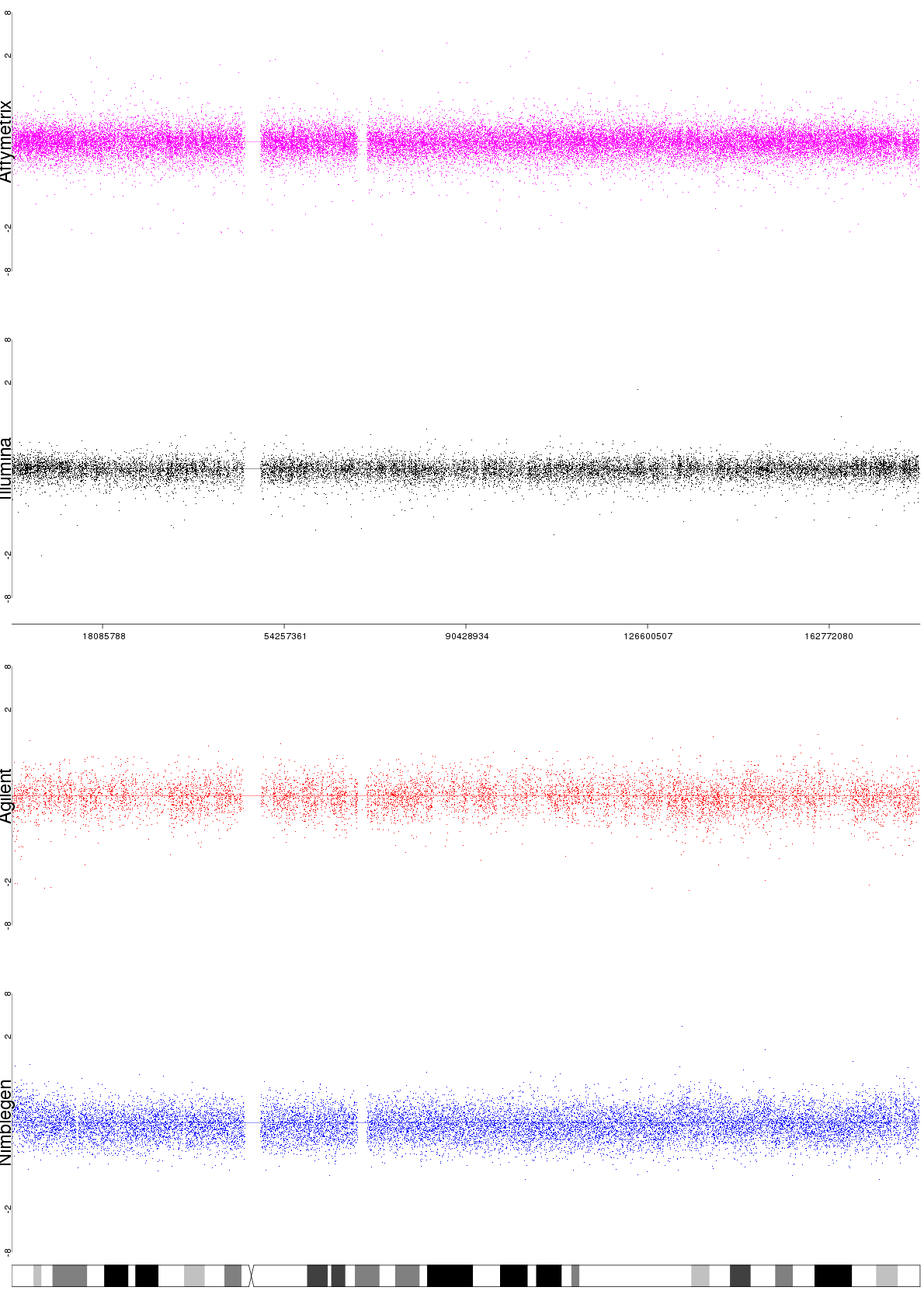

Supplement: Additional file 12 — All sample/chromosome plots for the tumours. Zip folder containing PNGs of all whole-chromosome plots for the tumours. [file 1471-2164-10-588-S12.ZIP › T7214/T7214 chromosome 5.png]

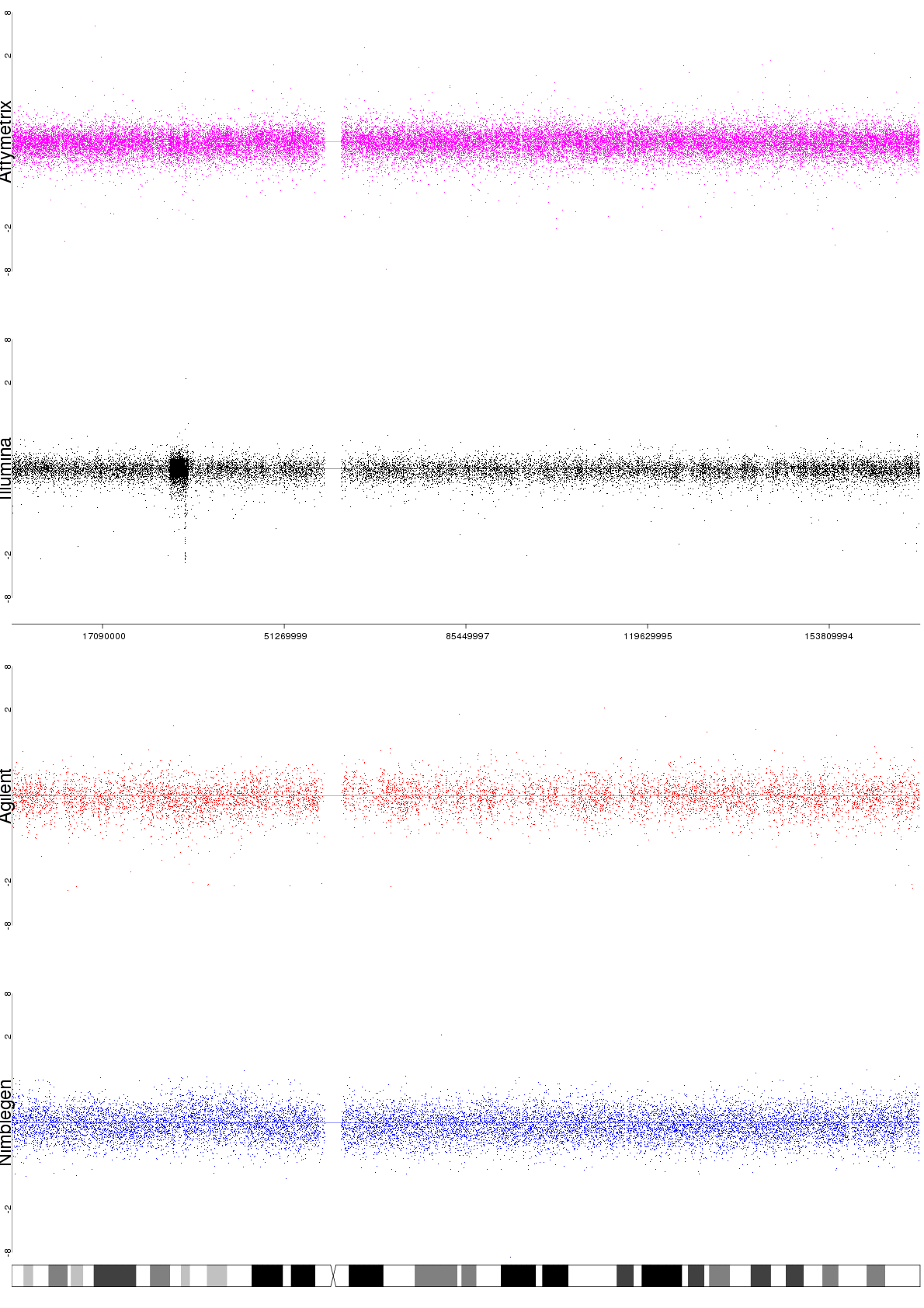

Supplement: Additional file 12 — All sample/chromosome plots for the tumours. Zip folder containing PNGs of all whole-chromosome plots for the tumours. [file 1471-2164-10-588-S12.ZIP › T7214/T7214 chromosome 6.png]

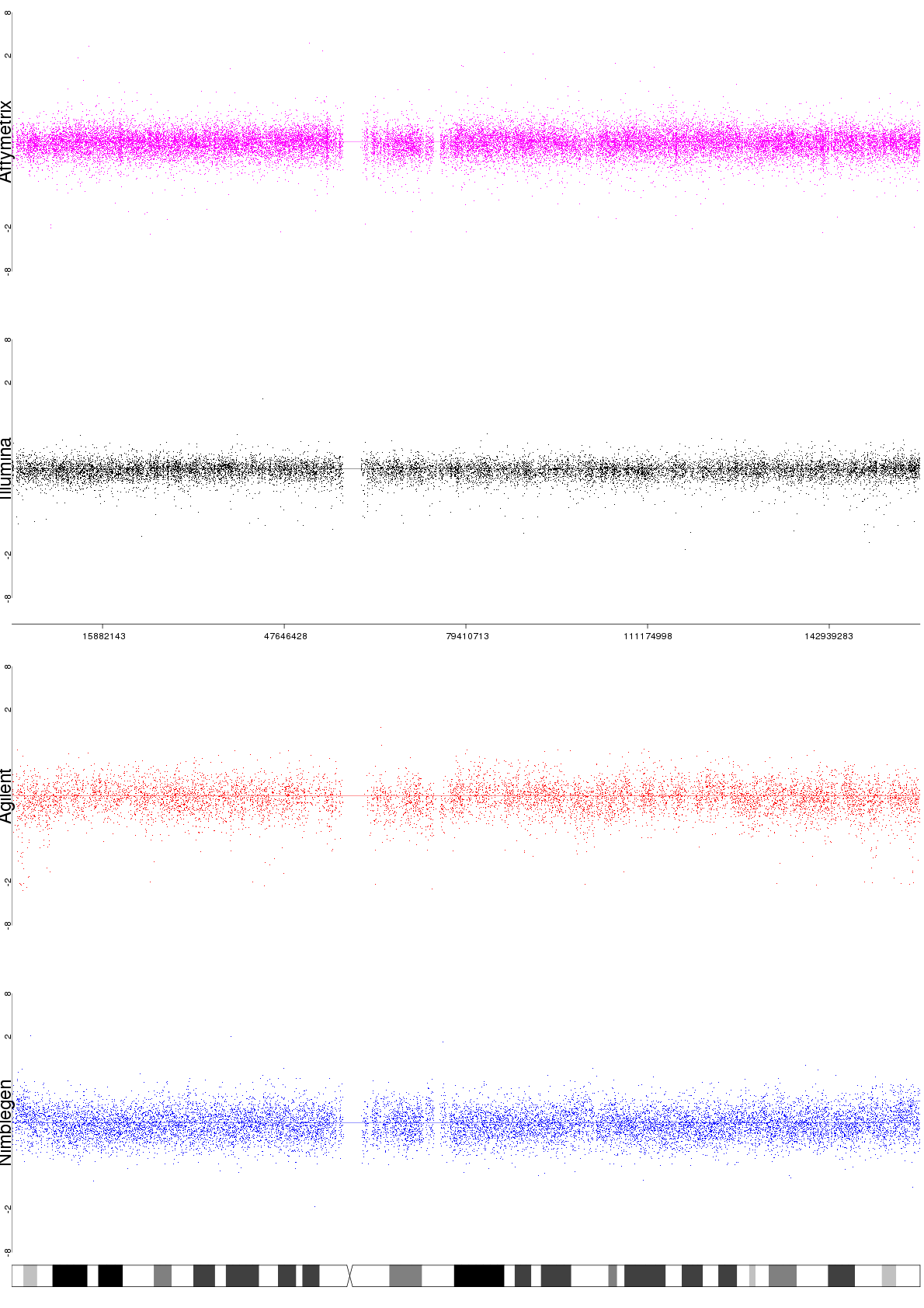

Supplement: Additional file 12 — All sample/chromosome plots for the tumours. Zip folder containing PNGs of all whole-chromosome plots for the tumours. [file 1471-2164-10-588-S12.ZIP › T7214/T7214 chromosome 7.png]

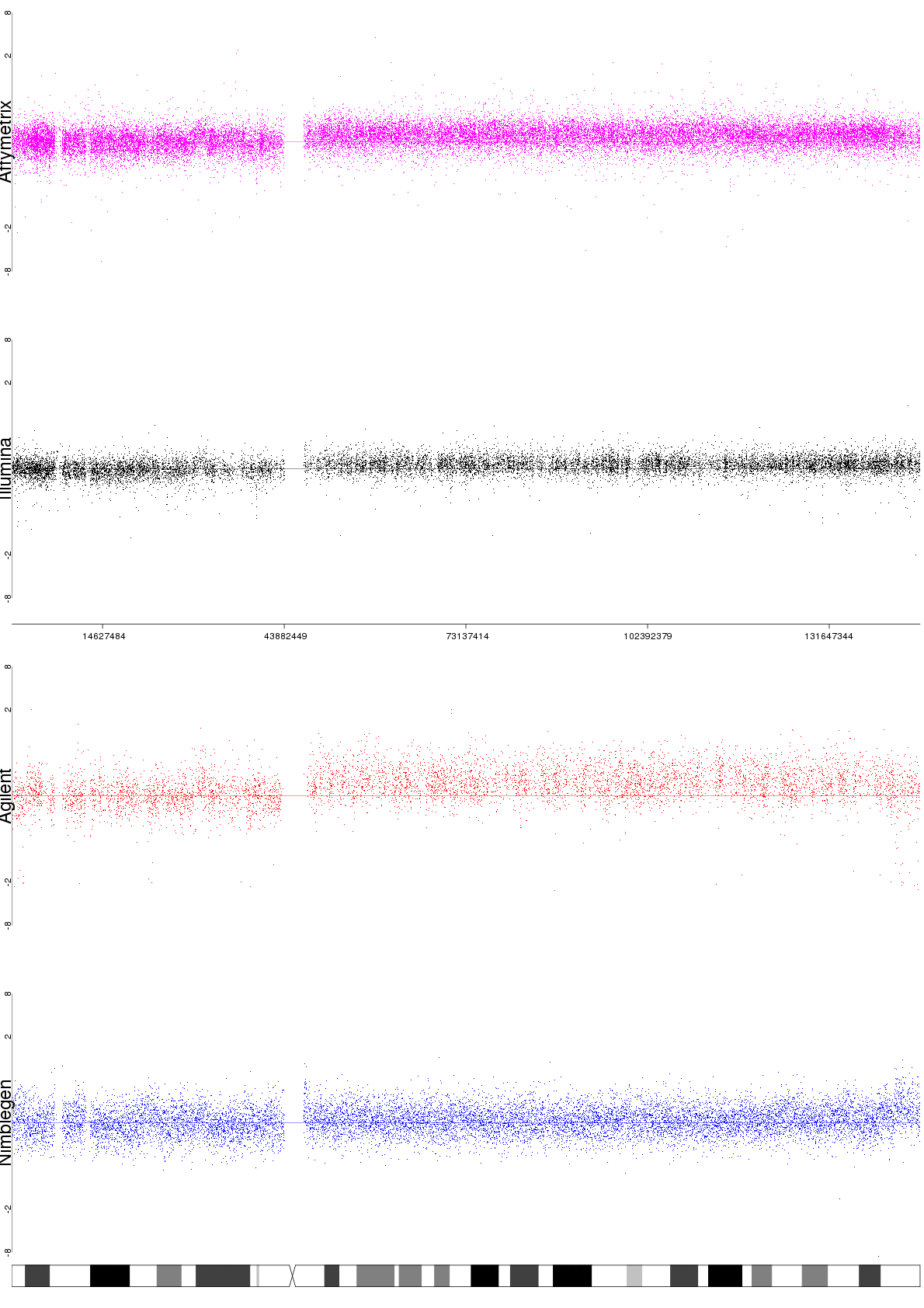

Supplement: Additional file 12 — All sample/chromosome plots for the tumours. Zip folder containing PNGs of all whole-chromosome plots for the tumours. [file 1471-2164-10-588-S12.ZIP › T7214/T7214 chromosome 8.png]

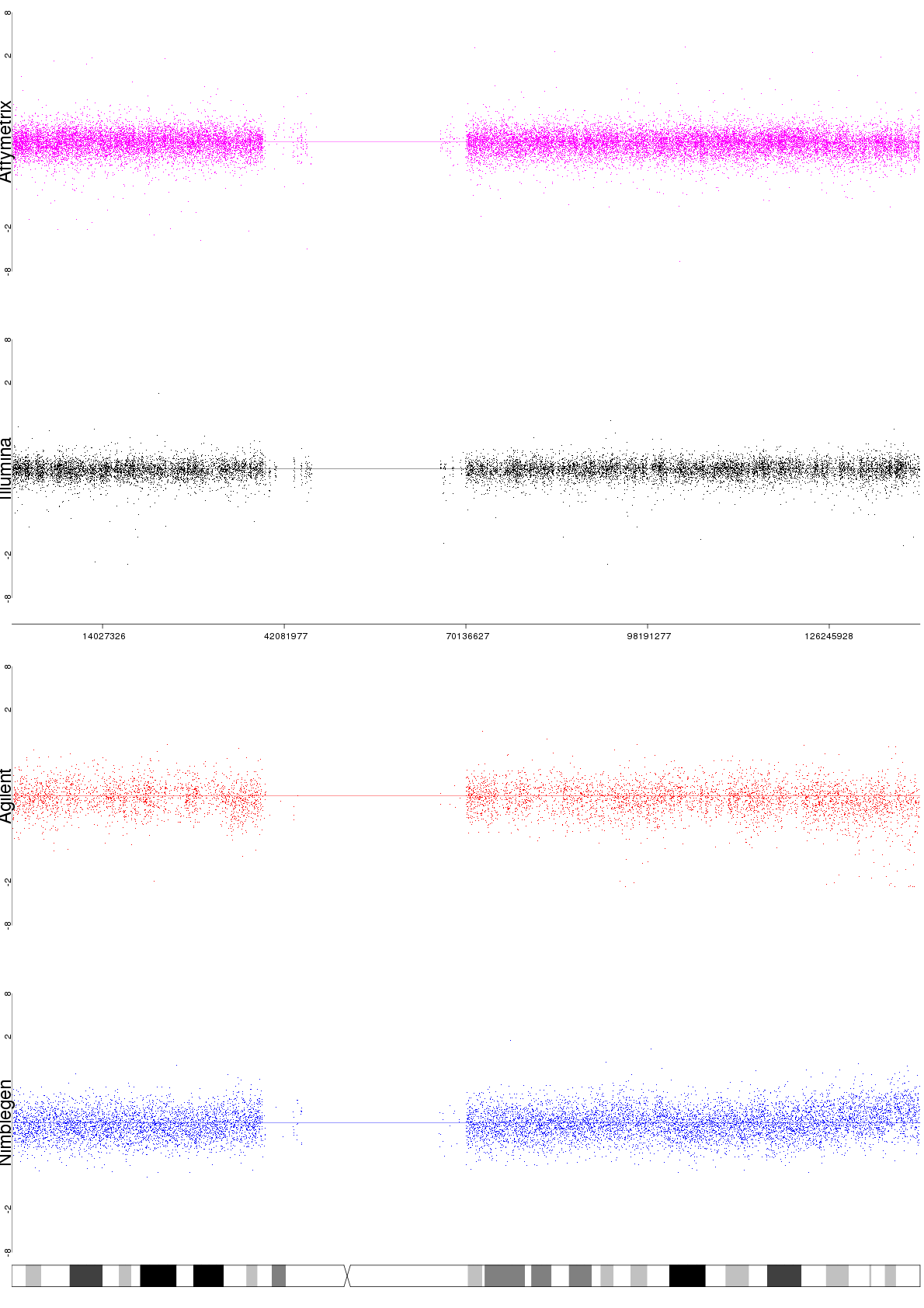

Supplement: Additional file 12 — All sample/chromosome plots for the tumours. Zip folder containing PNGs of all whole-chromosome plots for the tumours. [file 1471-2164-10-588-S12.ZIP › T7214/T7214 chromosome 9.png]

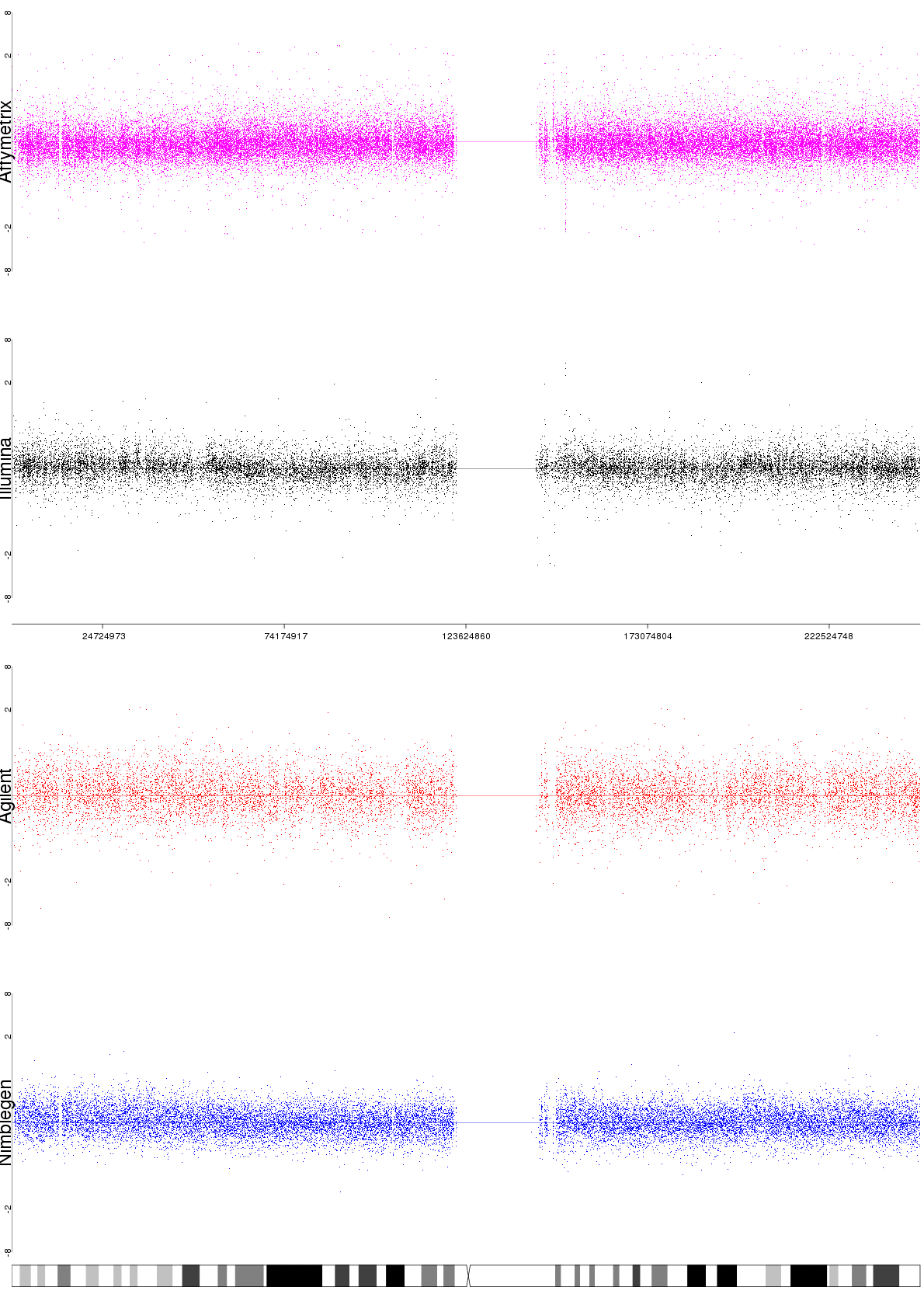

Supplement: Additional file 13 — All sample/chromosome plots for the cell-lines. Zip folder containing PNGs of all whole-chromosome plots for the cell-lines. [file 1471-2164-10-588-S13.ZIP › HapMap/HapMap chromosome 1.png]

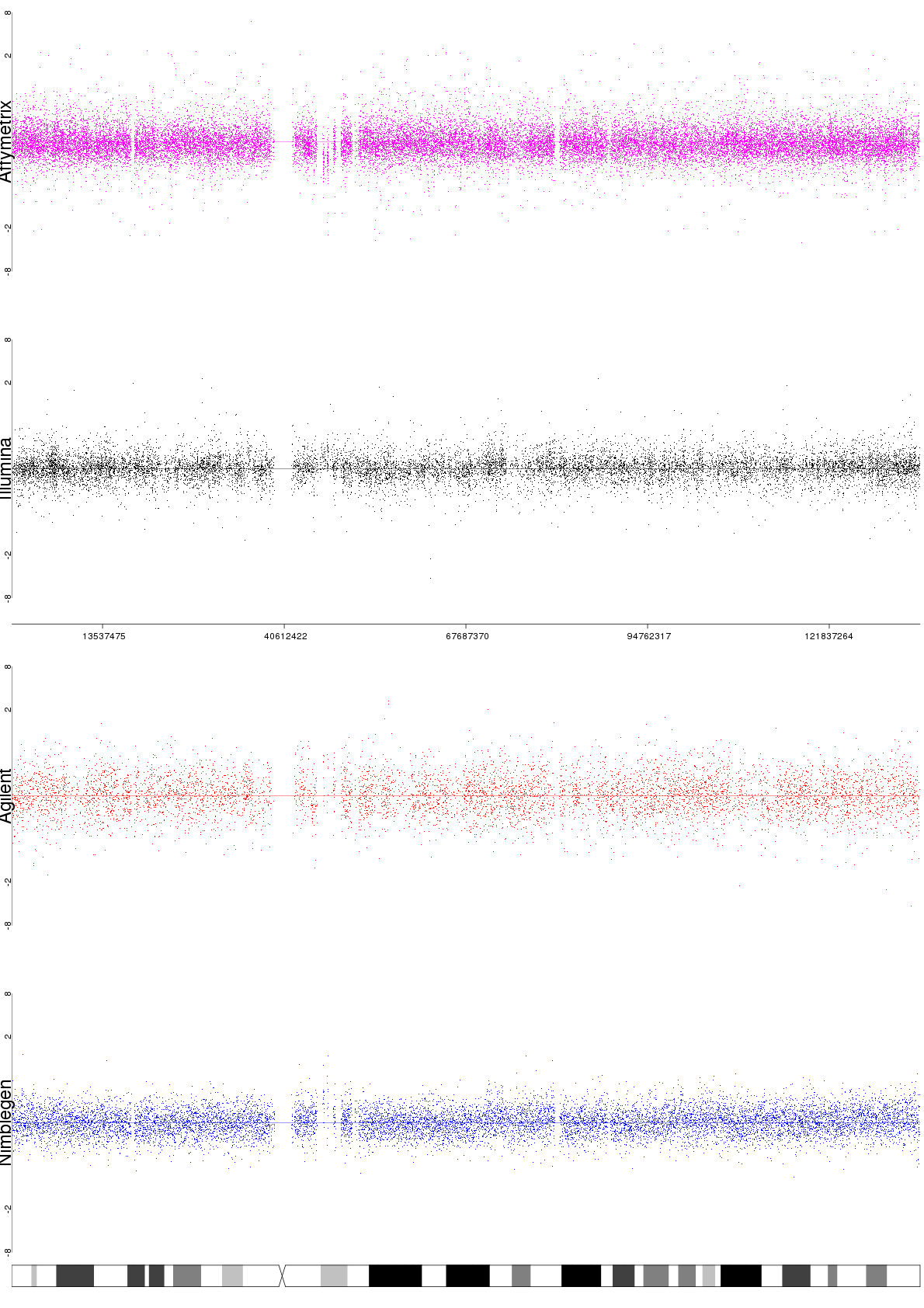

Supplement: Additional file 13 — All sample/chromosome plots for the cell-lines. Zip folder containing PNGs of all whole-chromosome plots for the cell-lines. [file 1471-2164-10-588-S13.ZIP › HapMap/HapMap chromosome 10.png]

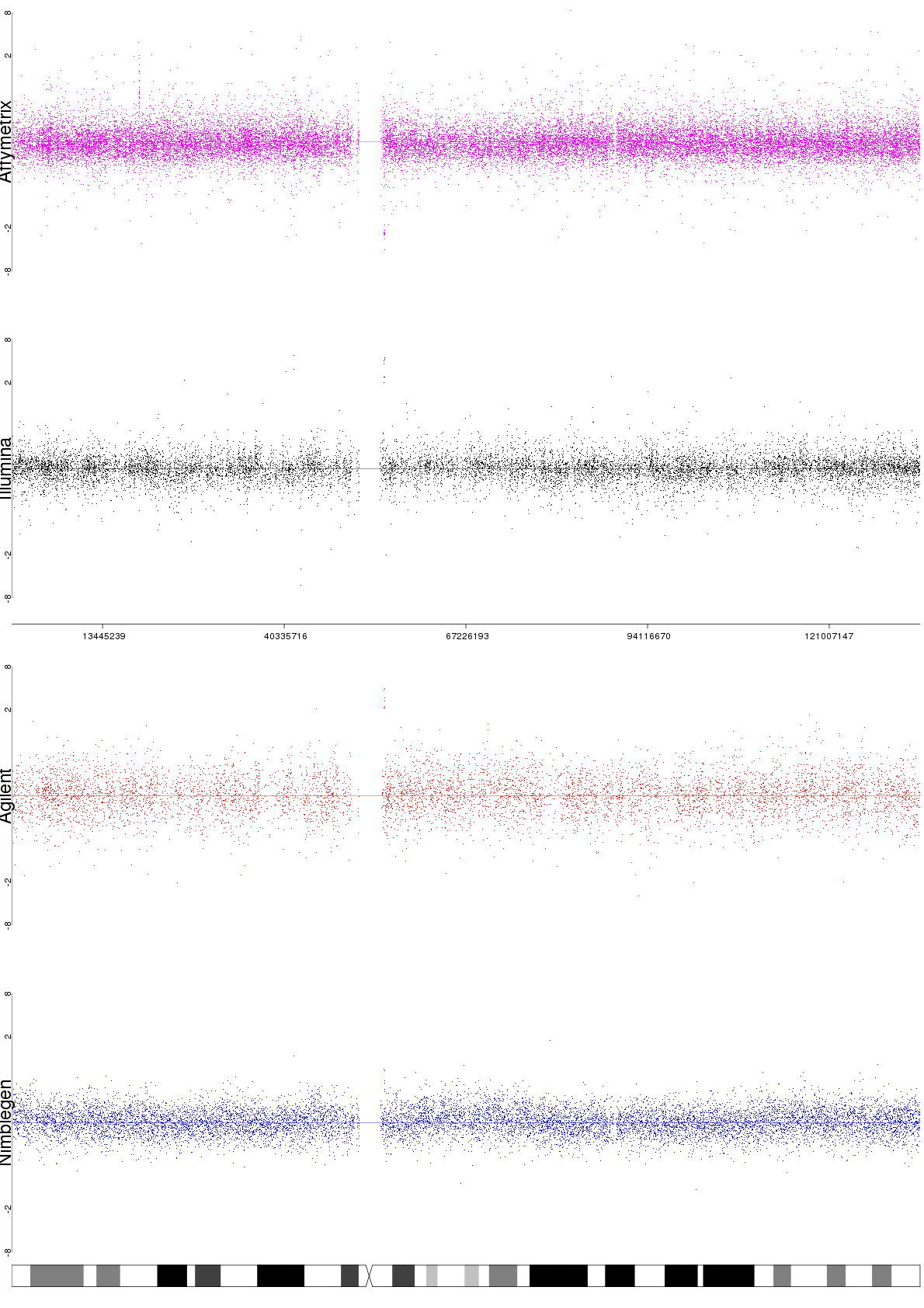

Supplement: Additional file 13 — All sample/chromosome plots for the cell-lines. Zip folder containing PNGs of all whole-chromosome plots for the cell-lines. [file 1471-2164-10-588-S13.ZIP › HapMap/HapMap chromosome 11.png]

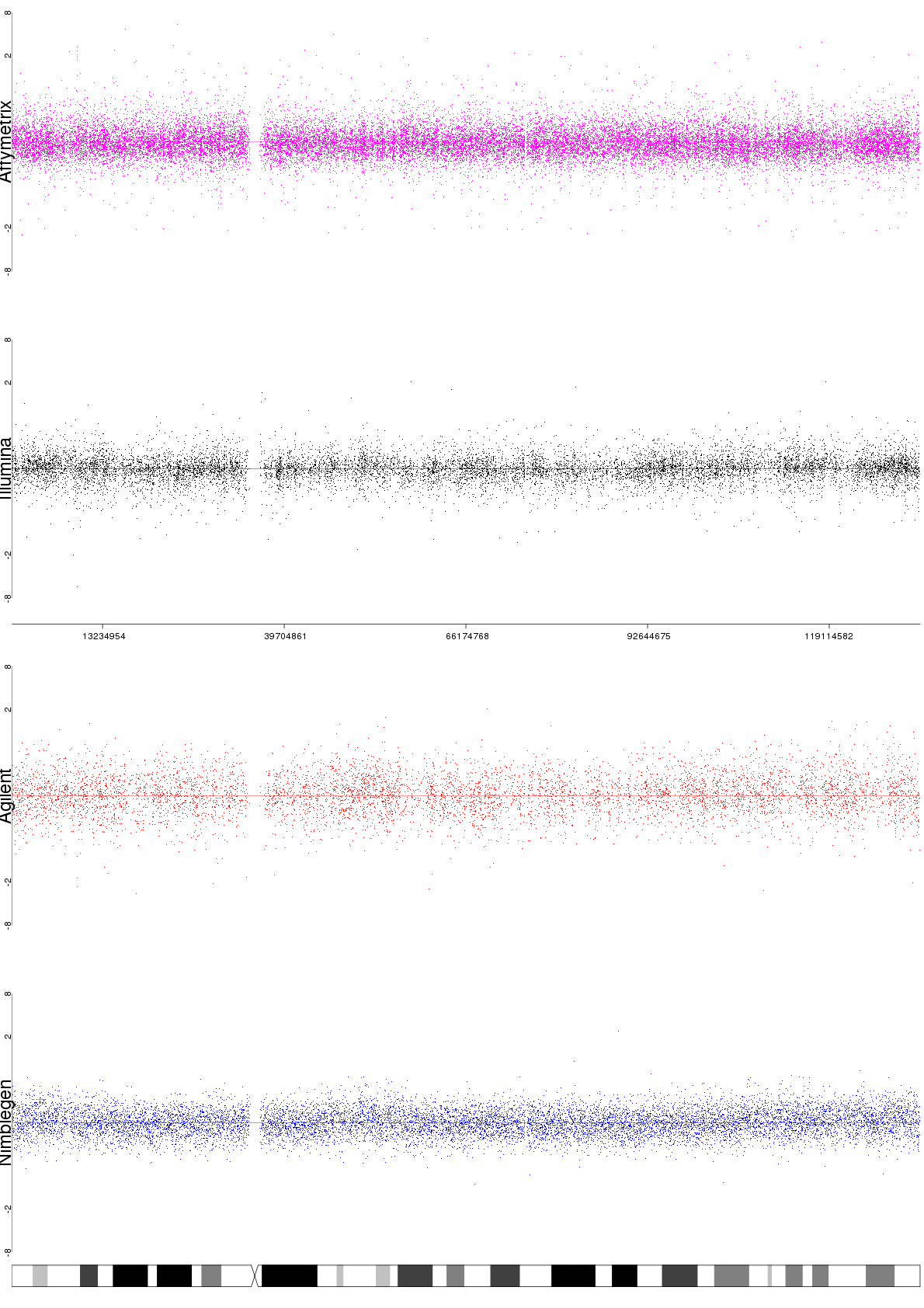

Supplement: Additional file 13 — All sample/chromosome plots for the cell-lines. Zip folder containing PNGs of all whole-chromosome plots for the cell-lines. [file 1471-2164-10-588-S13.ZIP › HapMap/HapMap chromosome 12.png]

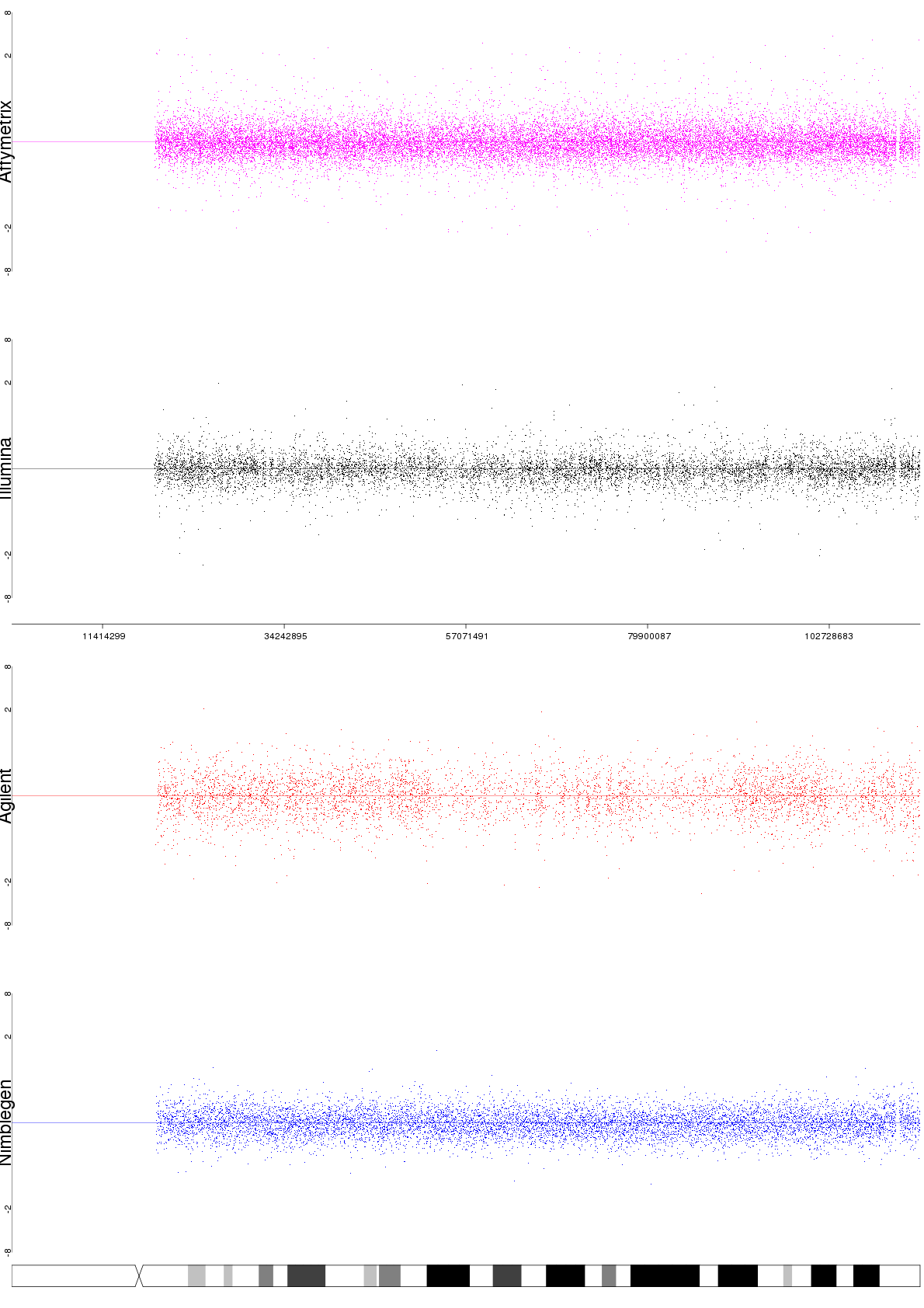

Supplement: Additional file 13 — All sample/chromosome plots for the cell-lines. Zip folder containing PNGs of all whole-chromosome plots for the cell-lines. [file 1471-2164-10-588-S13.ZIP › HapMap/HapMap chromosome 13.png]

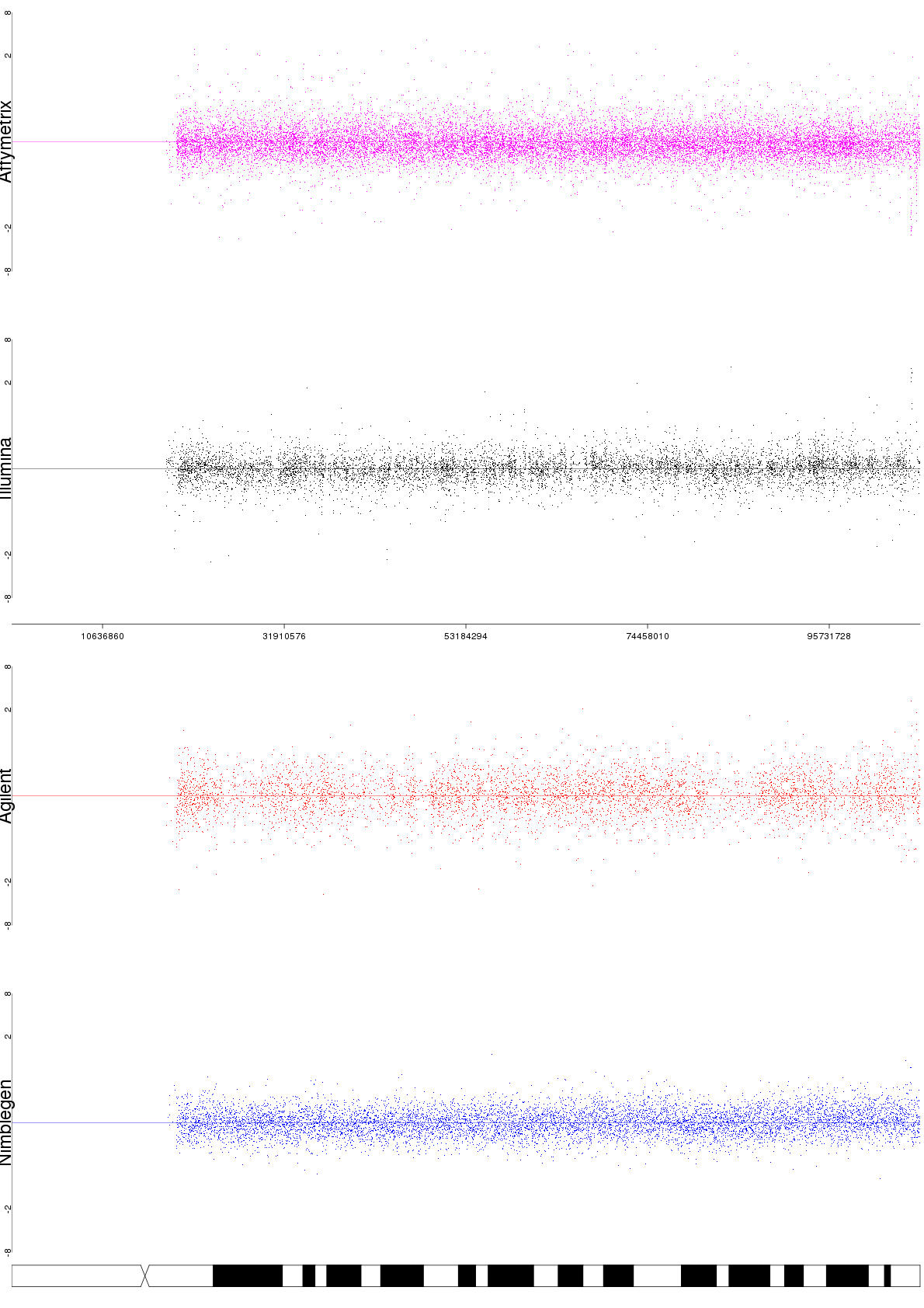

Supplement: Additional file 13 — All sample/chromosome plots for the cell-lines. Zip folder containing PNGs of all whole-chromosome plots for the cell-lines. [file 1471-2164-10-588-S13.ZIP › HapMap/HapMap chromosome 14.png]

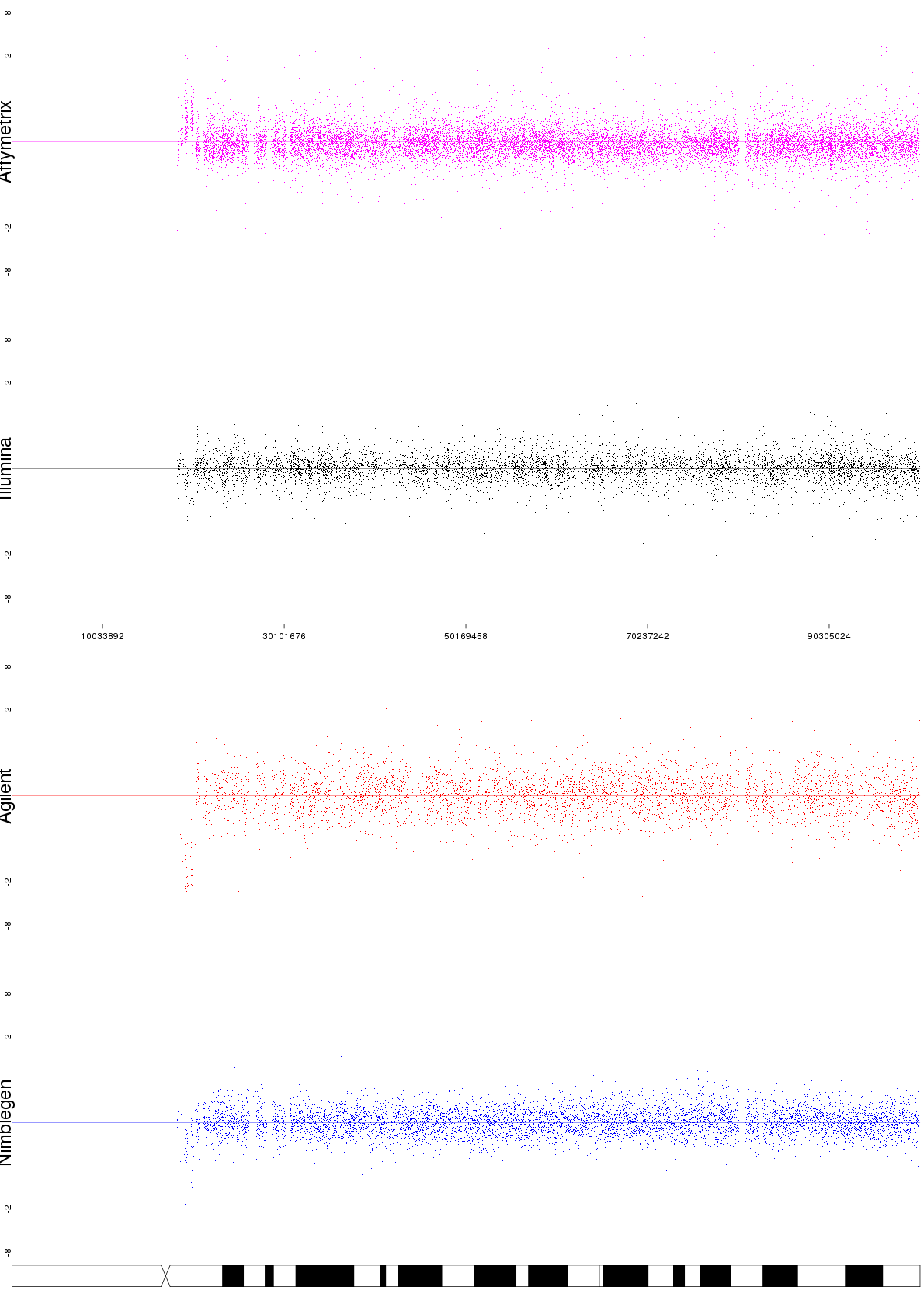

Supplement: Additional file 13 — All sample/chromosome plots for the cell-lines. Zip folder containing PNGs of all whole-chromosome plots for the cell-lines. [file 1471-2164-10-588-S13.ZIP › HapMap/HapMap chromosome 15.png]

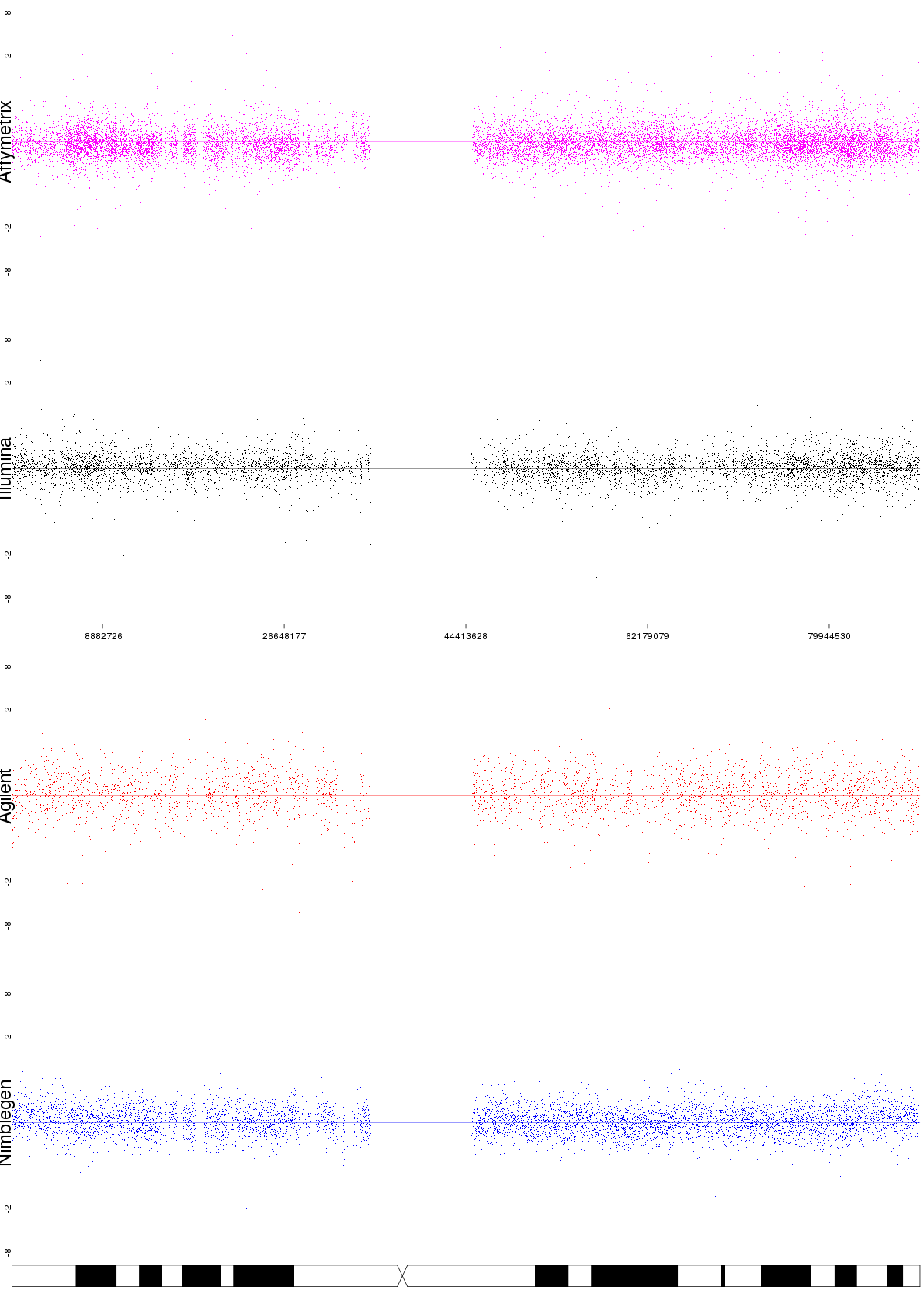

Supplement: Additional file 13 — All sample/chromosome plots for the cell-lines. Zip folder containing PNGs of all whole-chromosome plots for the cell-lines. [file 1471-2164-10-588-S13.ZIP › HapMap/HapMap chromosome 16.png]

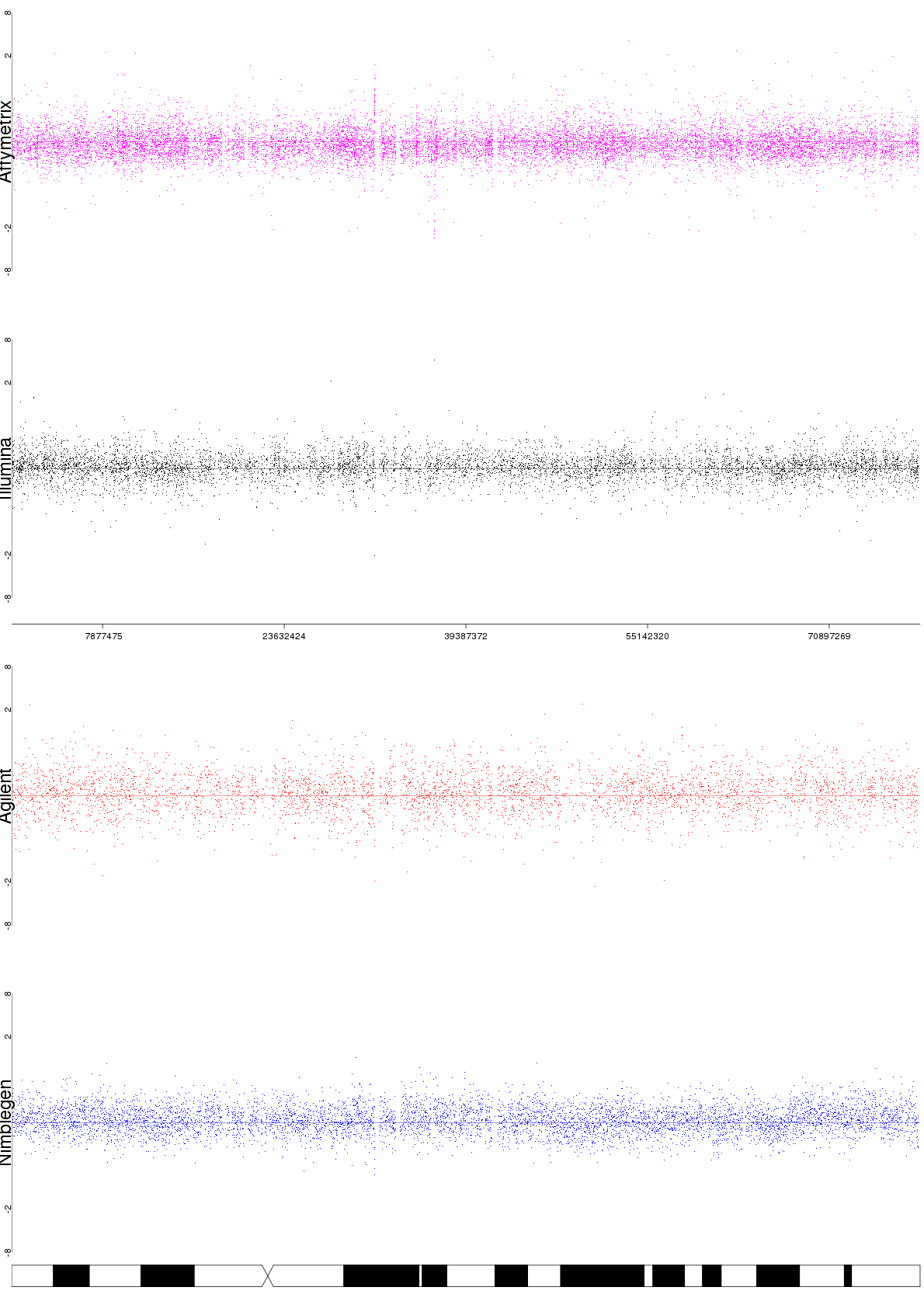

Supplement: Additional file 13 — All sample/chromosome plots for the cell-lines. Zip folder containing PNGs of all whole-chromosome plots for the cell-lines. [file 1471-2164-10-588-S13.ZIP › HapMap/HapMap chromosome 17.png]

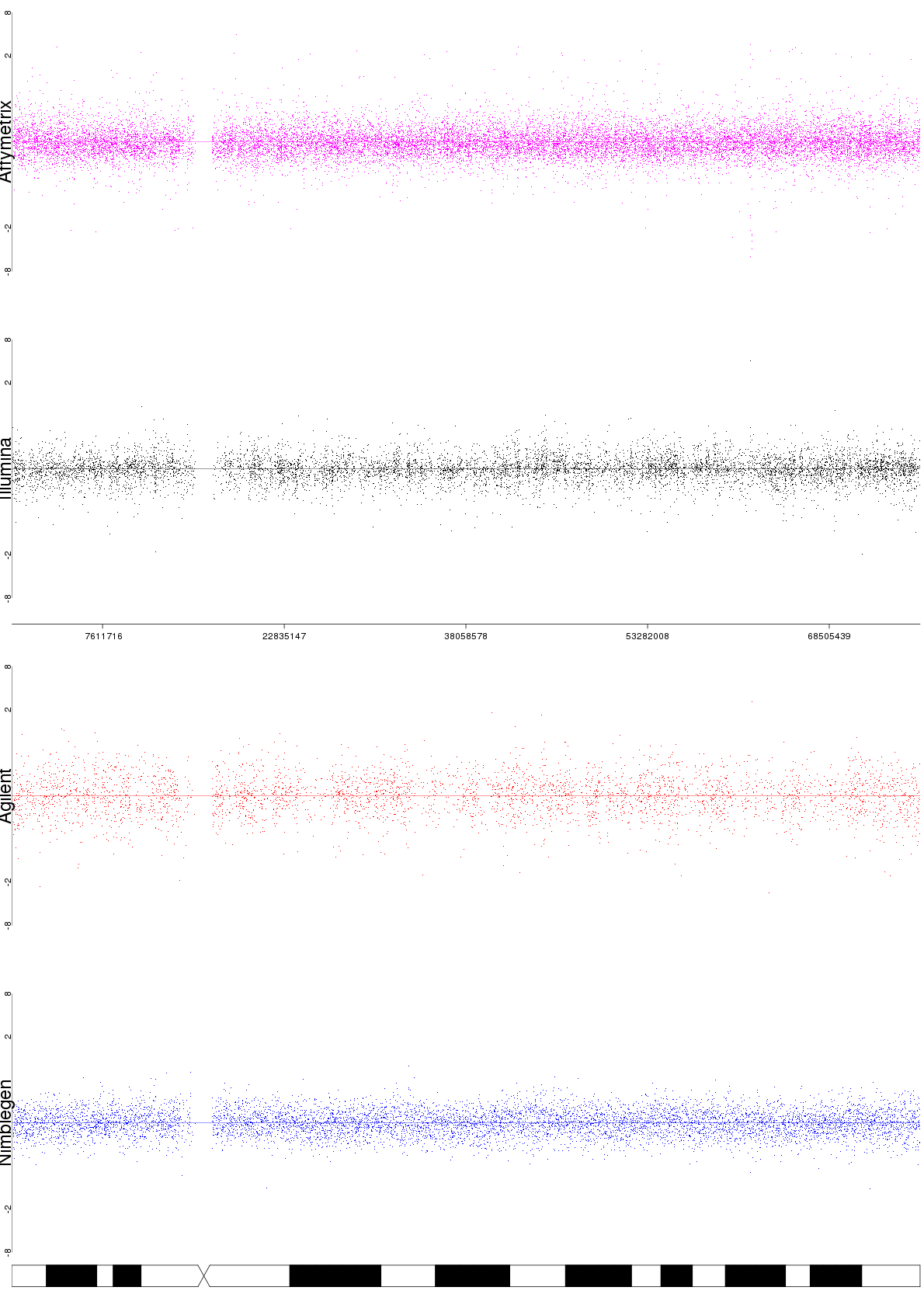

Supplement: Additional file 13 — All sample/chromosome plots for the cell-lines. Zip folder containing PNGs of all whole-chromosome plots for the cell-lines. [file 1471-2164-10-588-S13.ZIP › HapMap/HapMap chromosome 18.png]

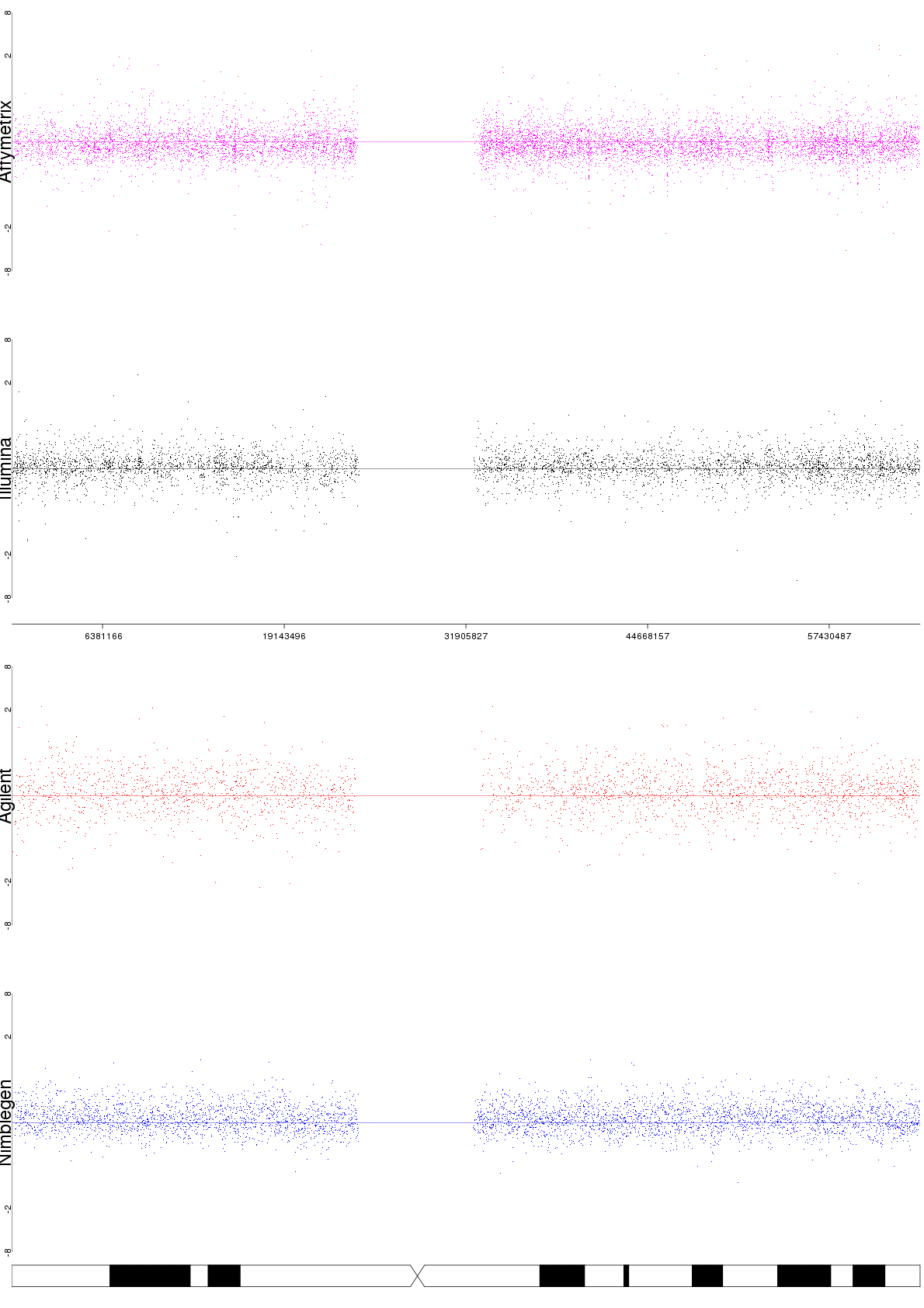

Supplement: Additional file 13 — All sample/chromosome plots for the cell-lines. Zip folder containing PNGs of all whole-chromosome plots for the cell-lines. [file 1471-2164-10-588-S13.ZIP › HapMap/HapMap chromosome 19.png]

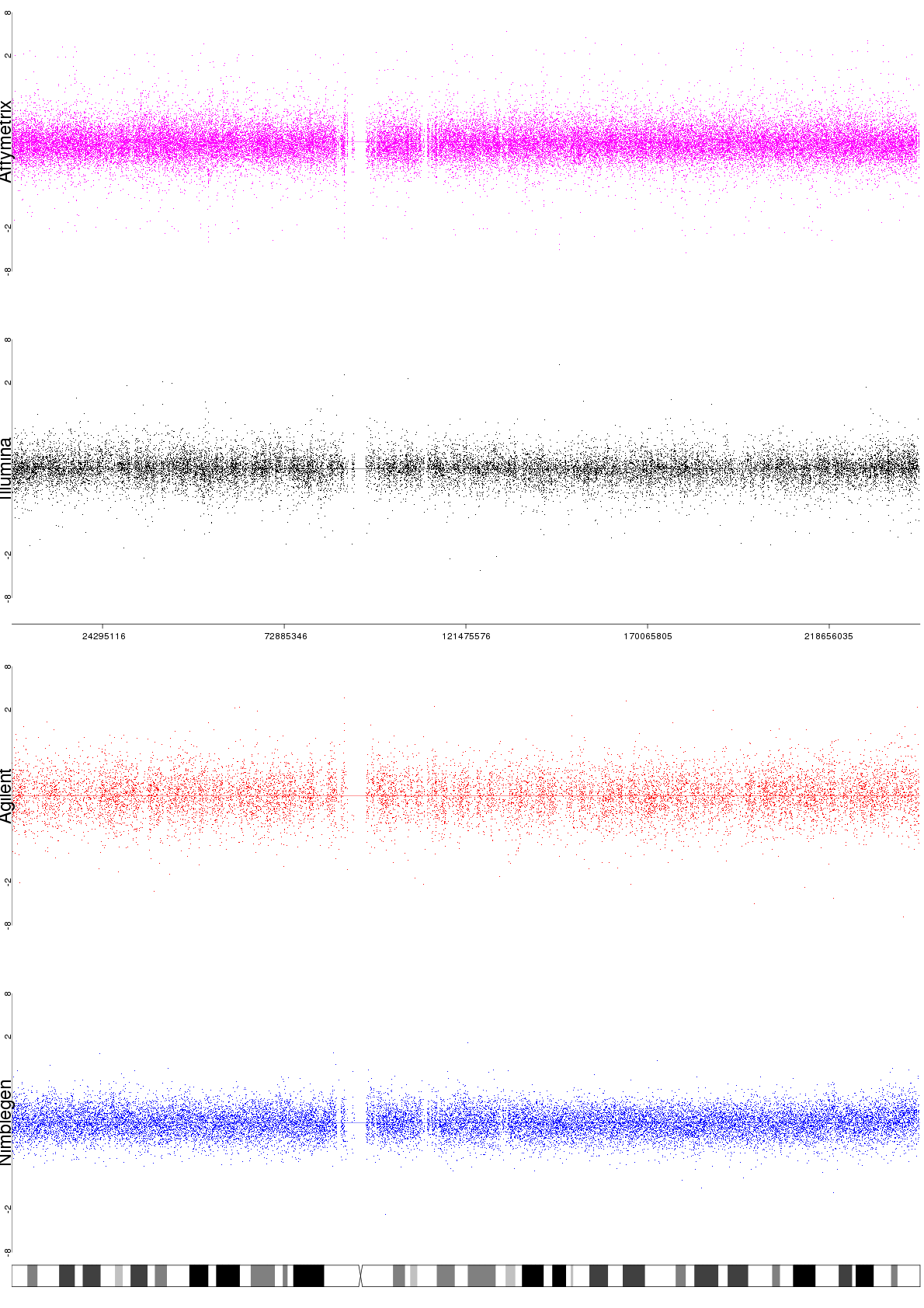

Supplement: Additional file 13 — All sample/chromosome plots for the cell-lines. Zip folder containing PNGs of all whole-chromosome plots for the cell-lines. [file 1471-2164-10-588-S13.ZIP › HapMap/HapMap chromosome 2.png]

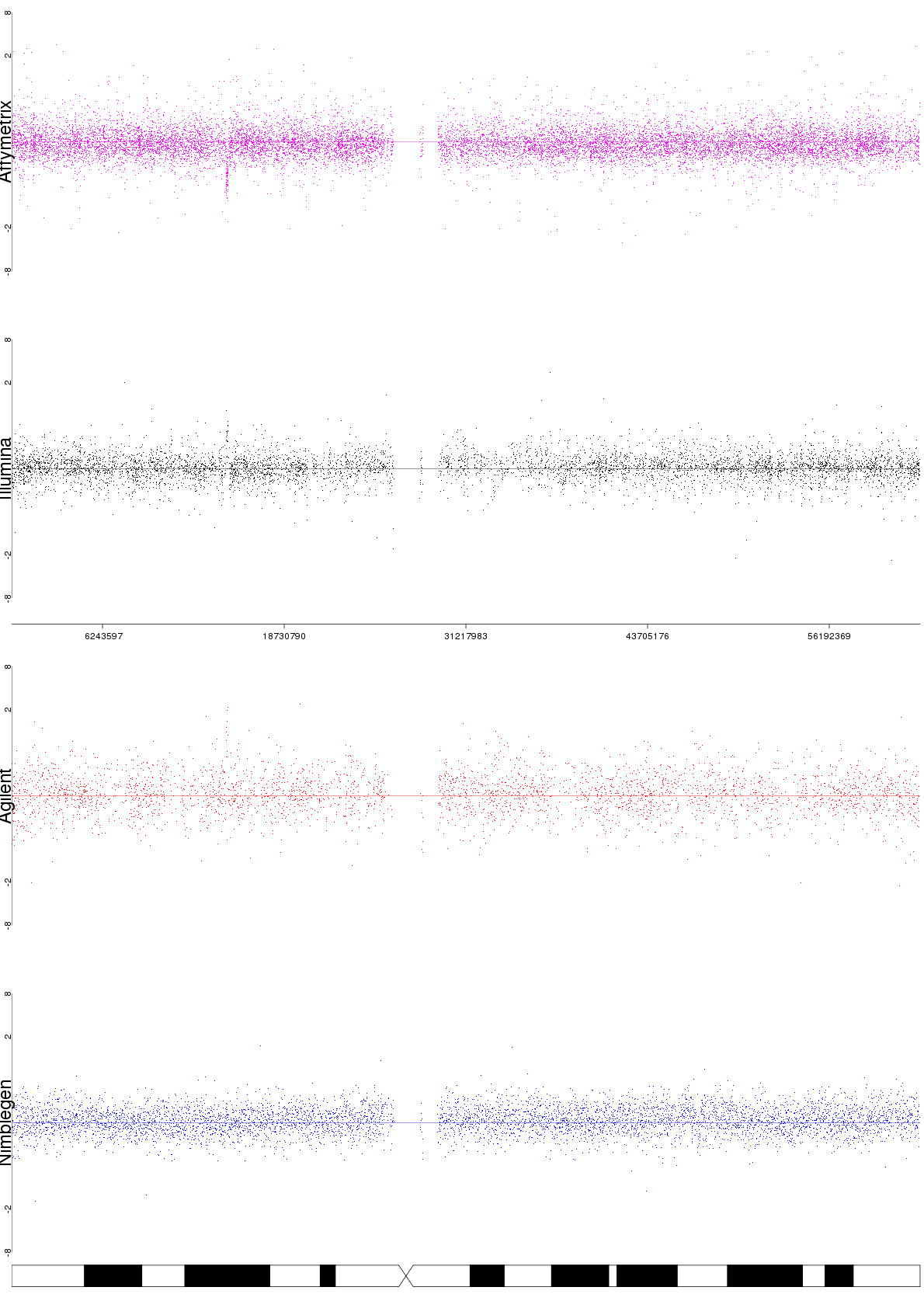

Supplement: Additional file 13 — All sample/chromosome plots for the cell-lines. Zip folder containing PNGs of all whole-chromosome plots for the cell-lines. [file 1471-2164-10-588-S13.ZIP › HapMap/HapMap chromosome 20.png]

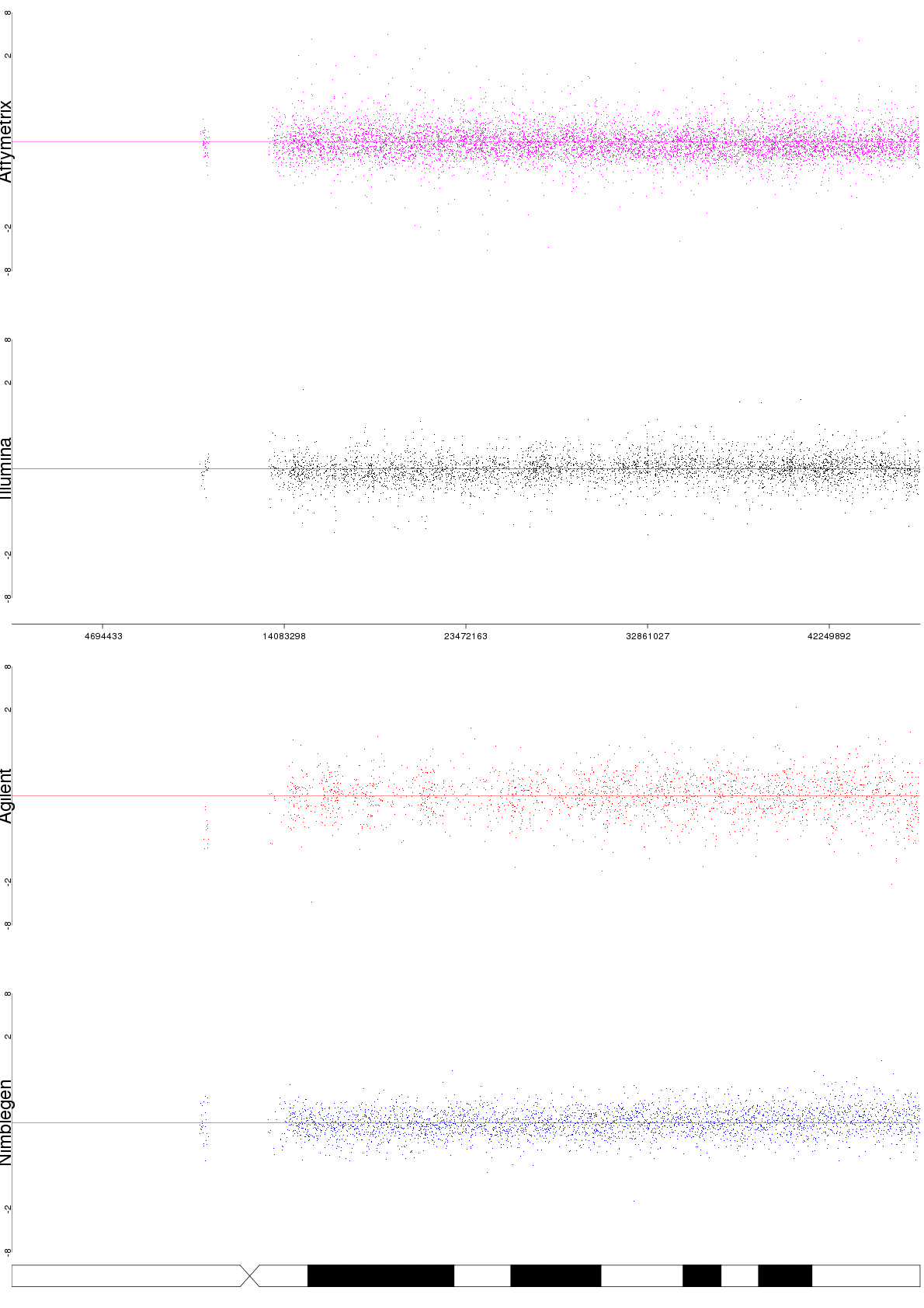

Supplement: Additional file 13 — All sample/chromosome plots for the cell-lines. Zip folder containing PNGs of all whole-chromosome plots for the cell-lines. [file 1471-2164-10-588-S13.ZIP › HapMap/HapMap chromosome 21.png]

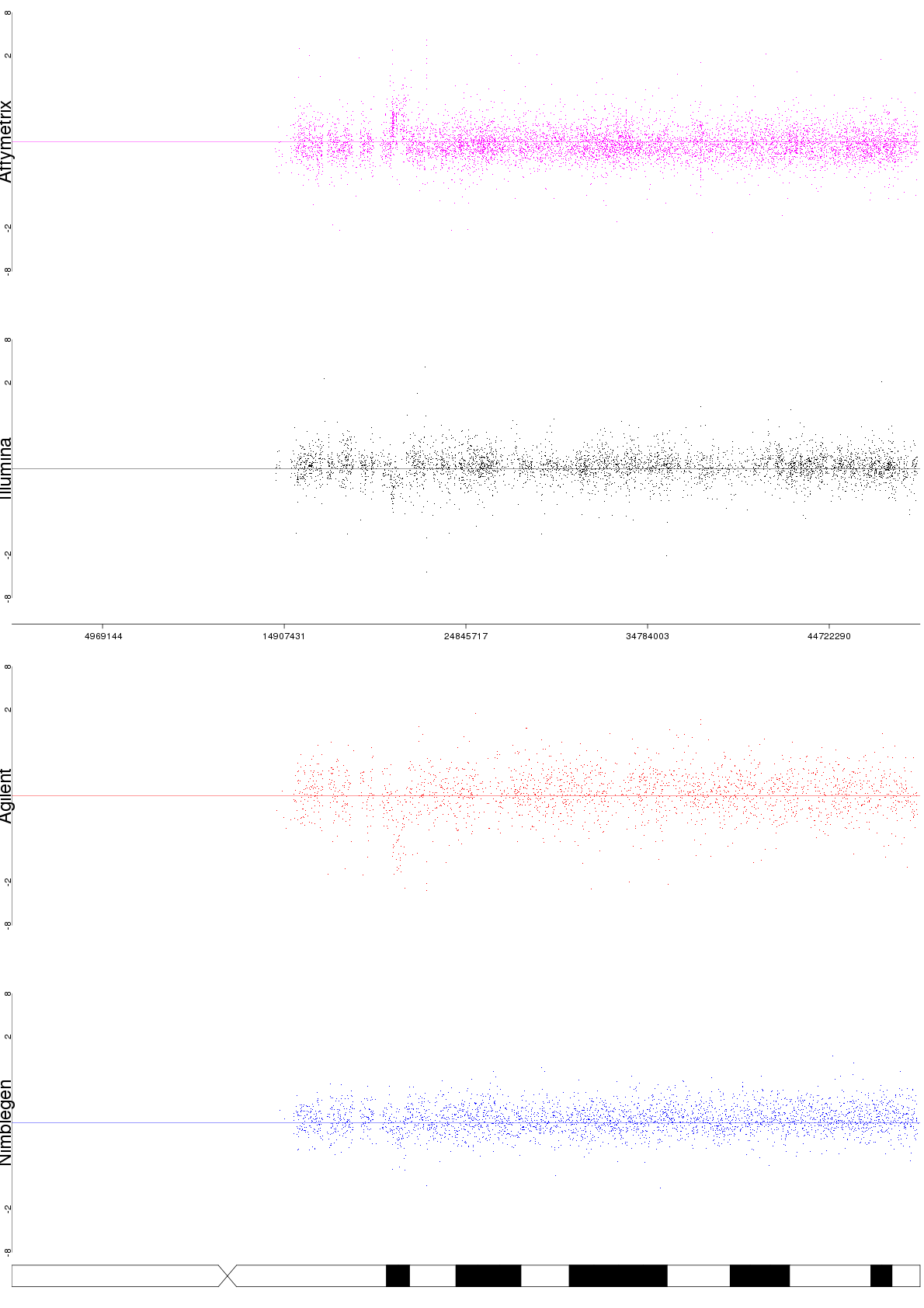

Supplement: Additional file 13 — All sample/chromosome plots for the cell-lines. Zip folder containing PNGs of all whole-chromosome plots for the cell-lines. [file 1471-2164-10-588-S13.ZIP › HapMap/HapMap chromosome 22.png]

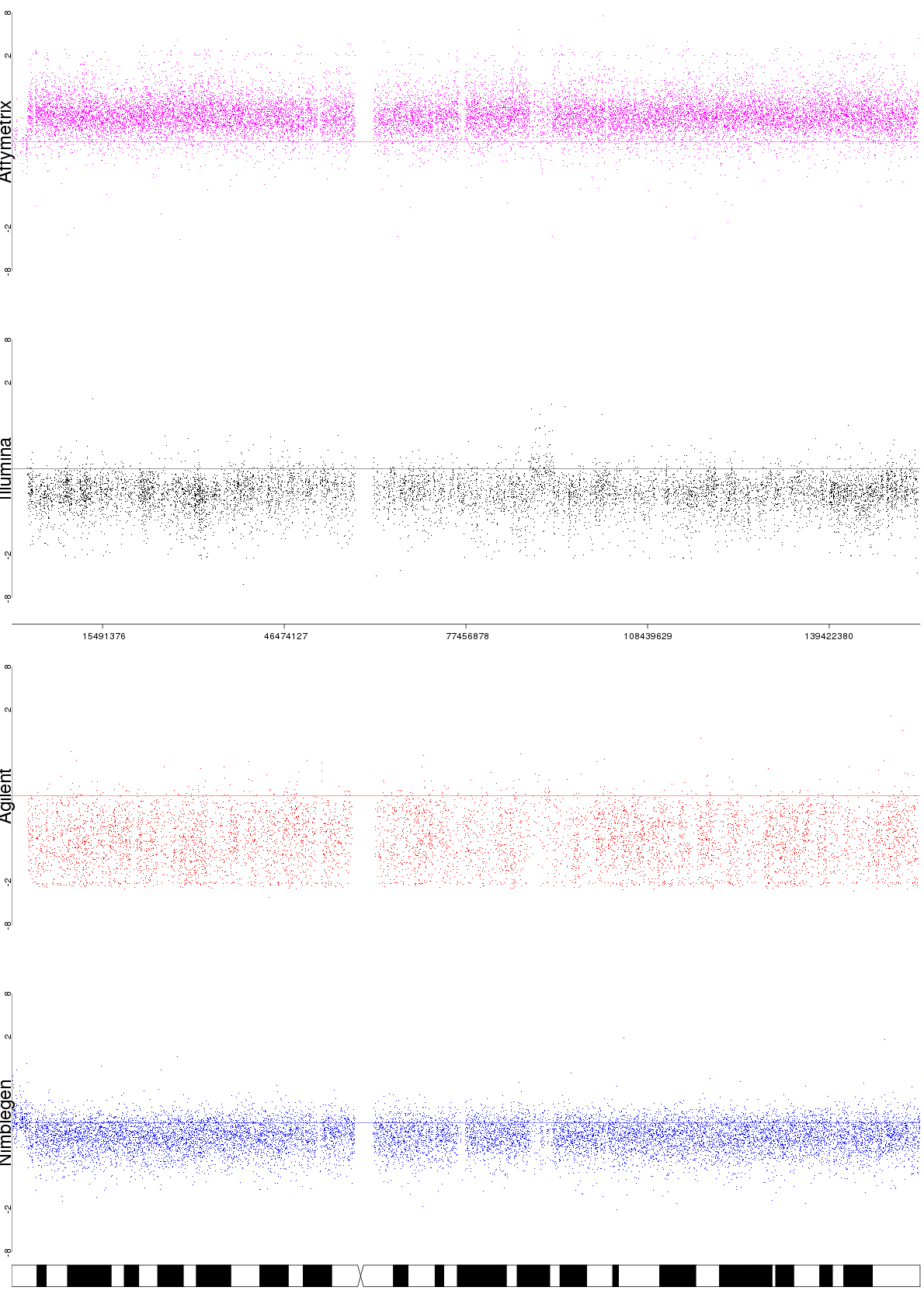

Supplement: Additional file 13 — All sample/chromosome plots for the cell-lines. Zip folder containing PNGs of all whole-chromosome plots for the cell-lines. [file 1471-2164-10-588-S13.ZIP › HapMap/HapMap chromosome 23.png]

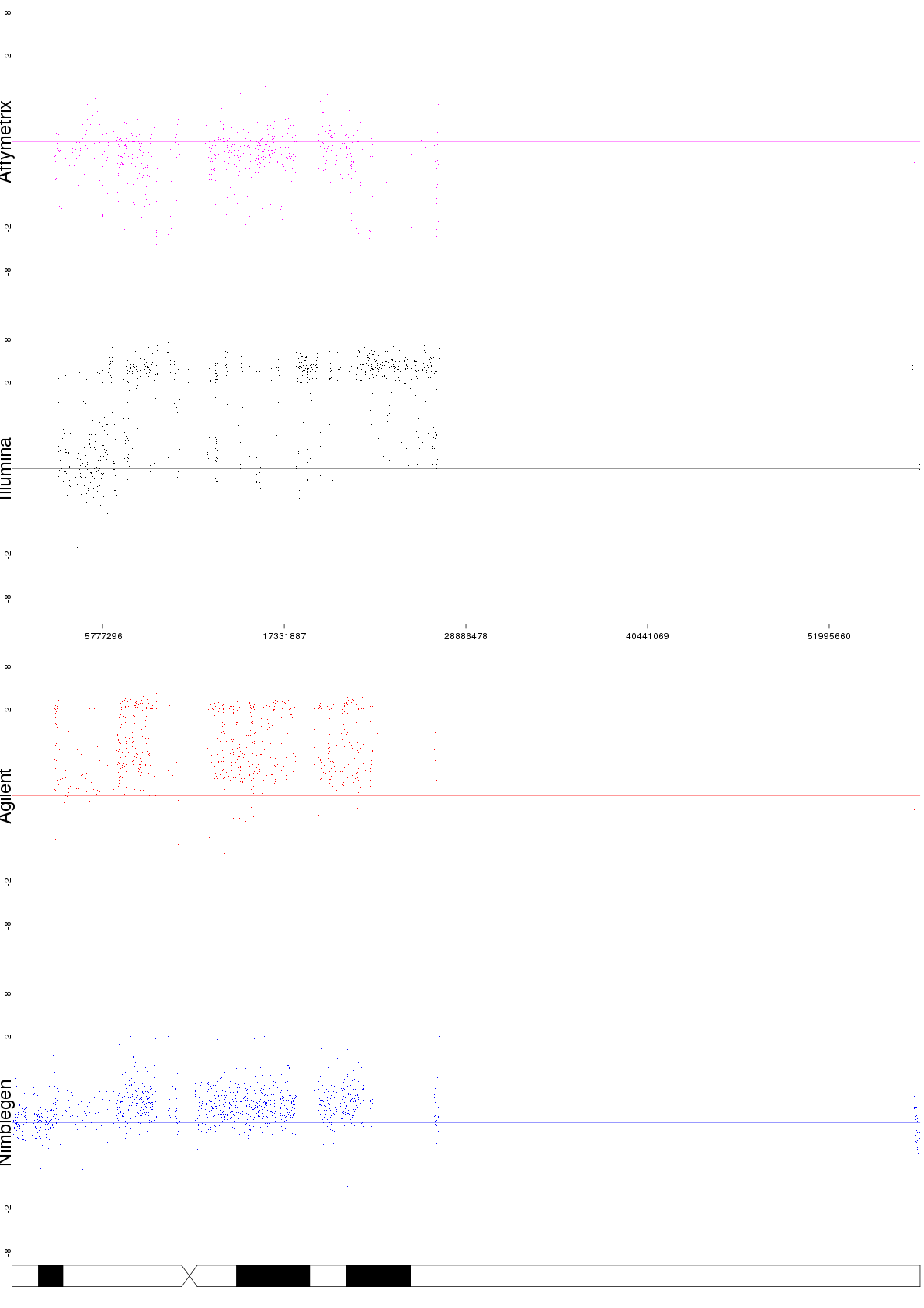

Supplement: Additional file 13 — All sample/chromosome plots for the cell-lines. Zip folder containing PNGs of all whole-chromosome plots for the cell-lines. [file 1471-2164-10-588-S13.ZIP › HapMap/HapMap chromosome 24.png]

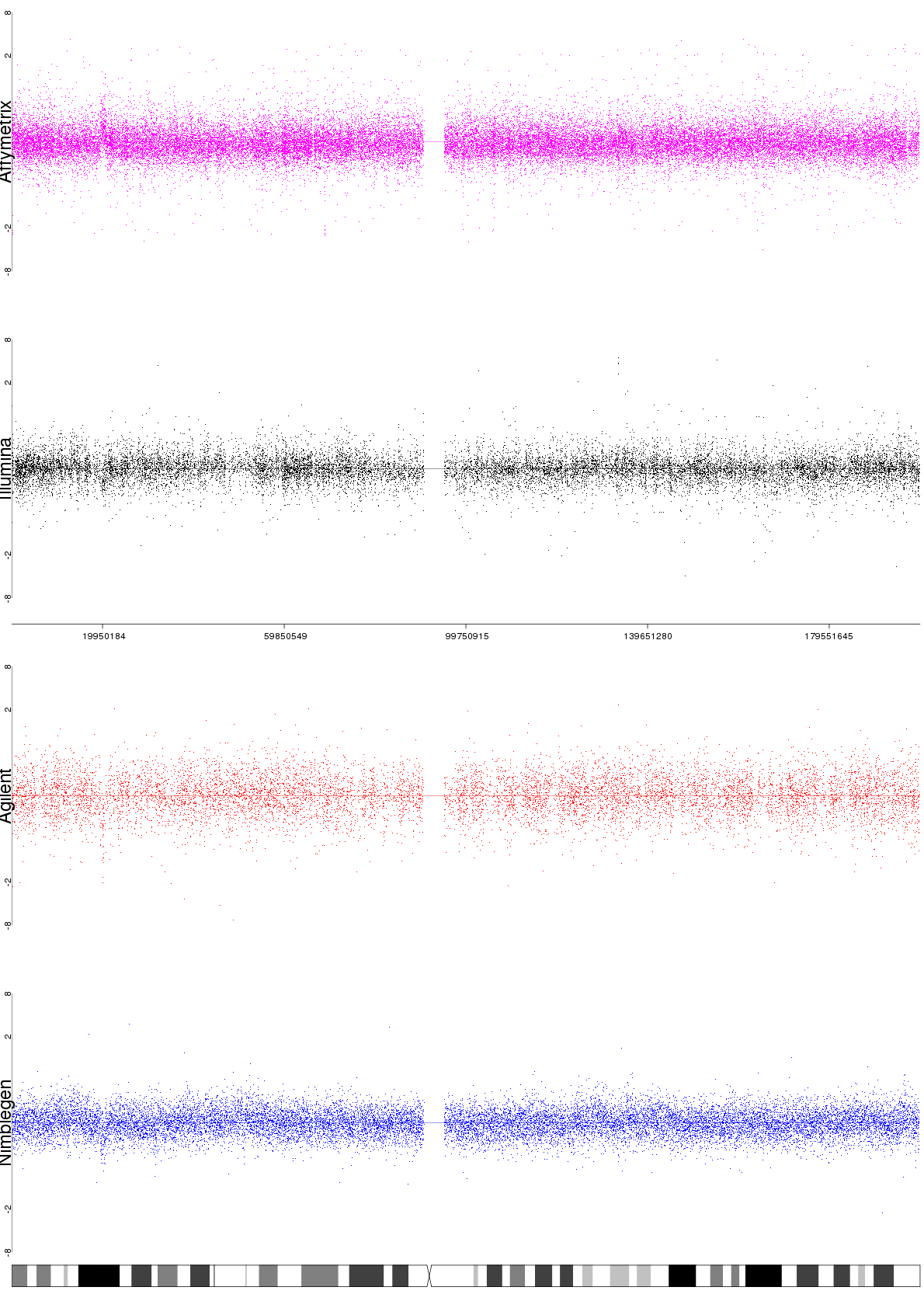

Supplement: Additional file 13 — All sample/chromosome plots for the cell-lines. Zip folder containing PNGs of all whole-chromosome plots for the cell-lines. [file 1471-2164-10-588-S13.ZIP › HapMap/HapMap chromosome 3.png]

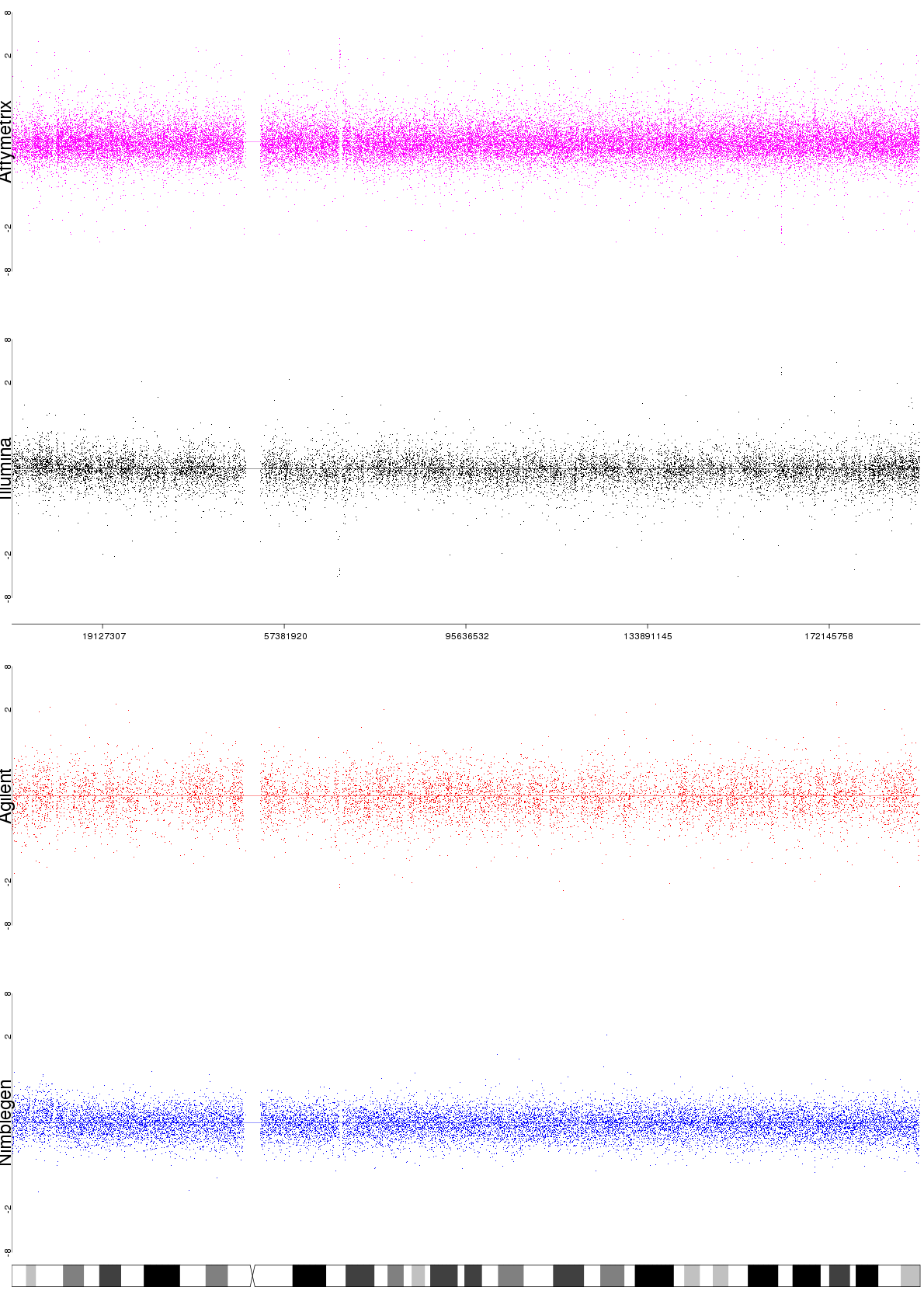

Supplement: Additional file 13 — All sample/chromosome plots for the cell-lines. Zip folder containing PNGs of all whole-chromosome plots for the cell-lines. [file 1471-2164-10-588-S13.ZIP › HapMap/HapMap chromosome 4.png]

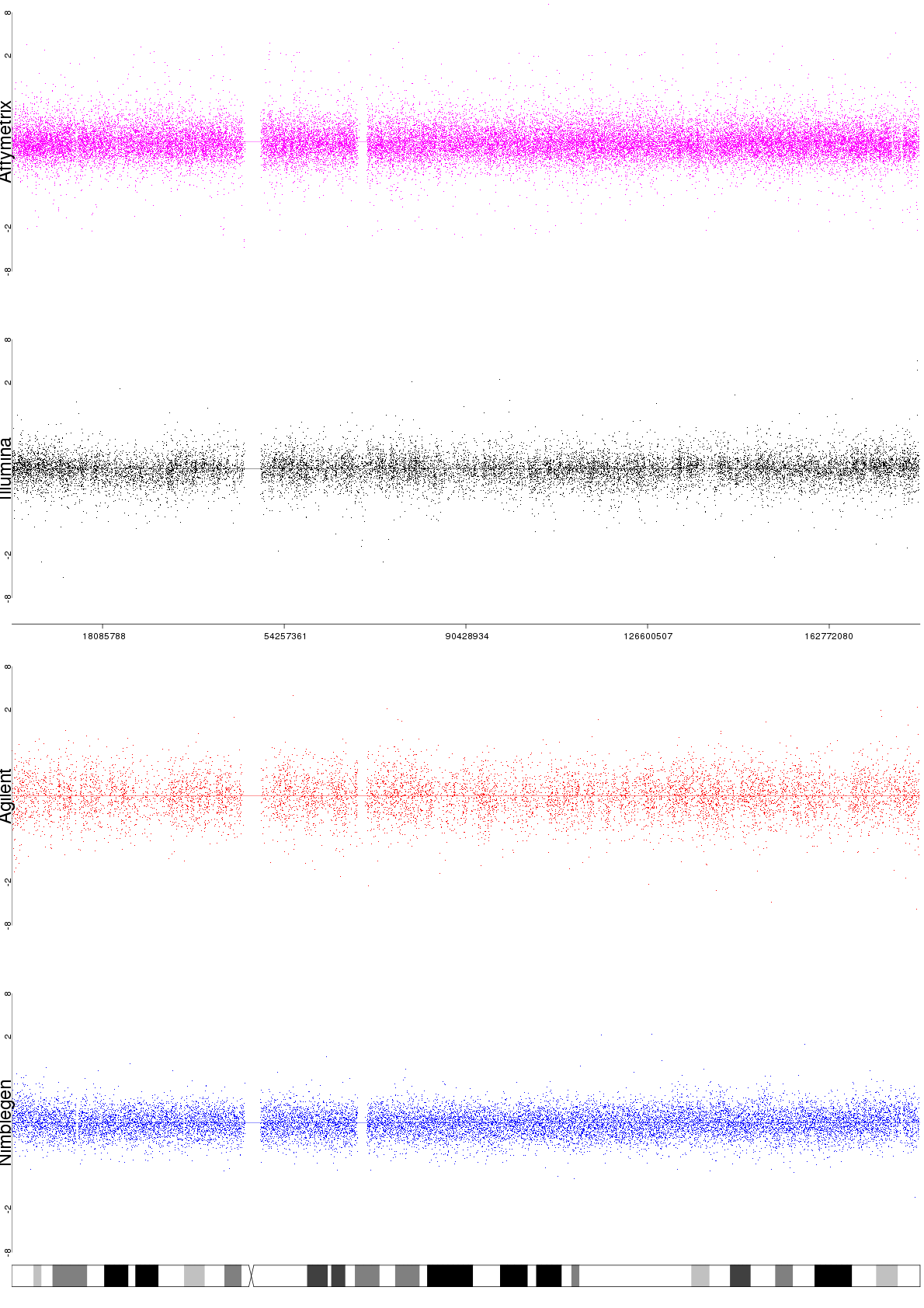

Supplement: Additional file 13 — All sample/chromosome plots for the cell-lines. Zip folder containing PNGs of all whole-chromosome plots for the cell-lines. [file 1471-2164-10-588-S13.ZIP › HapMap/HapMap chromosome 5.png]

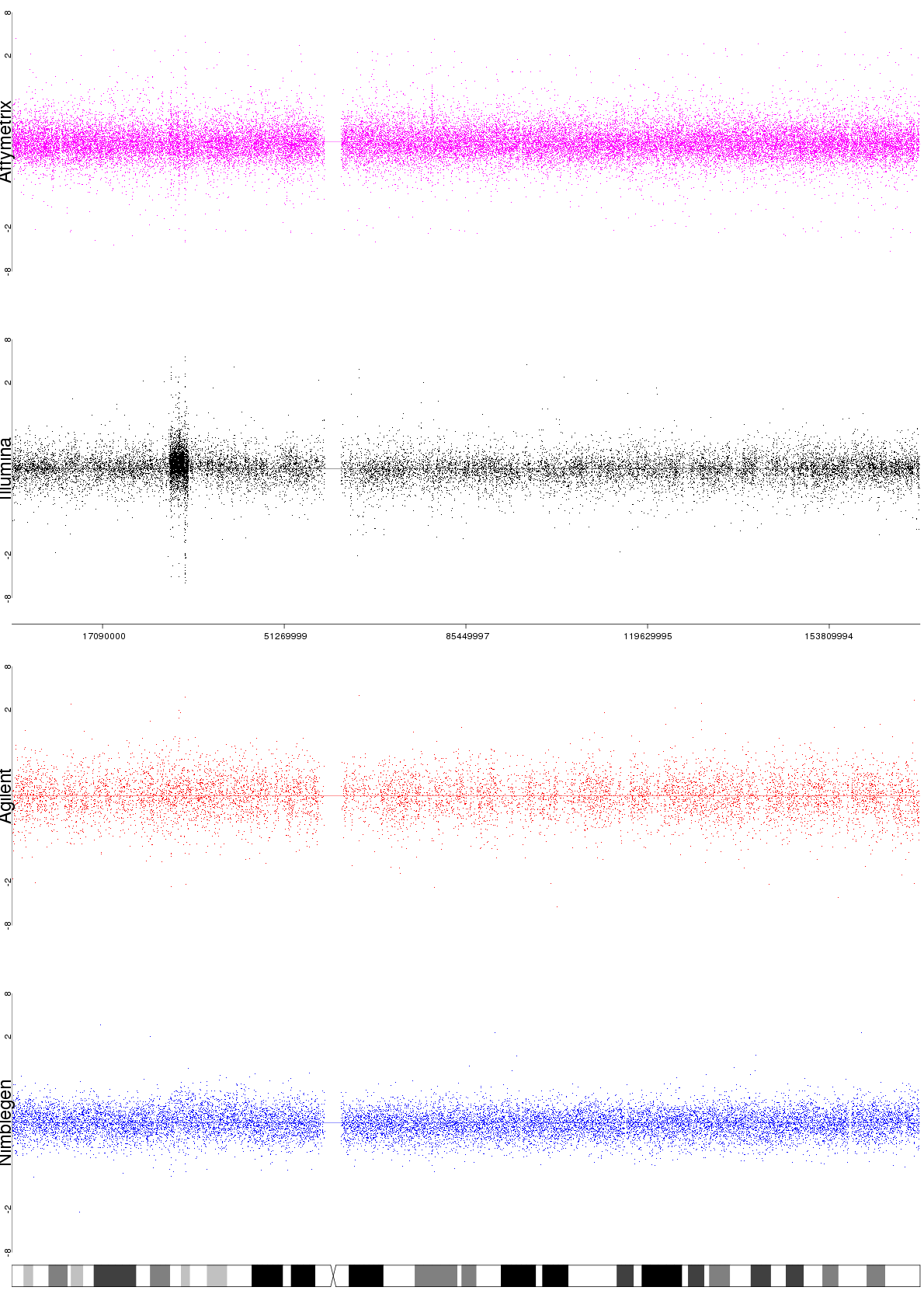

Supplement: Additional file 13 — All sample/chromosome plots for the cell-lines. Zip folder containing PNGs of all whole-chromosome plots for the cell-lines. [file 1471-2164-10-588-S13.ZIP › HapMap/HapMap chromosome 6.png]

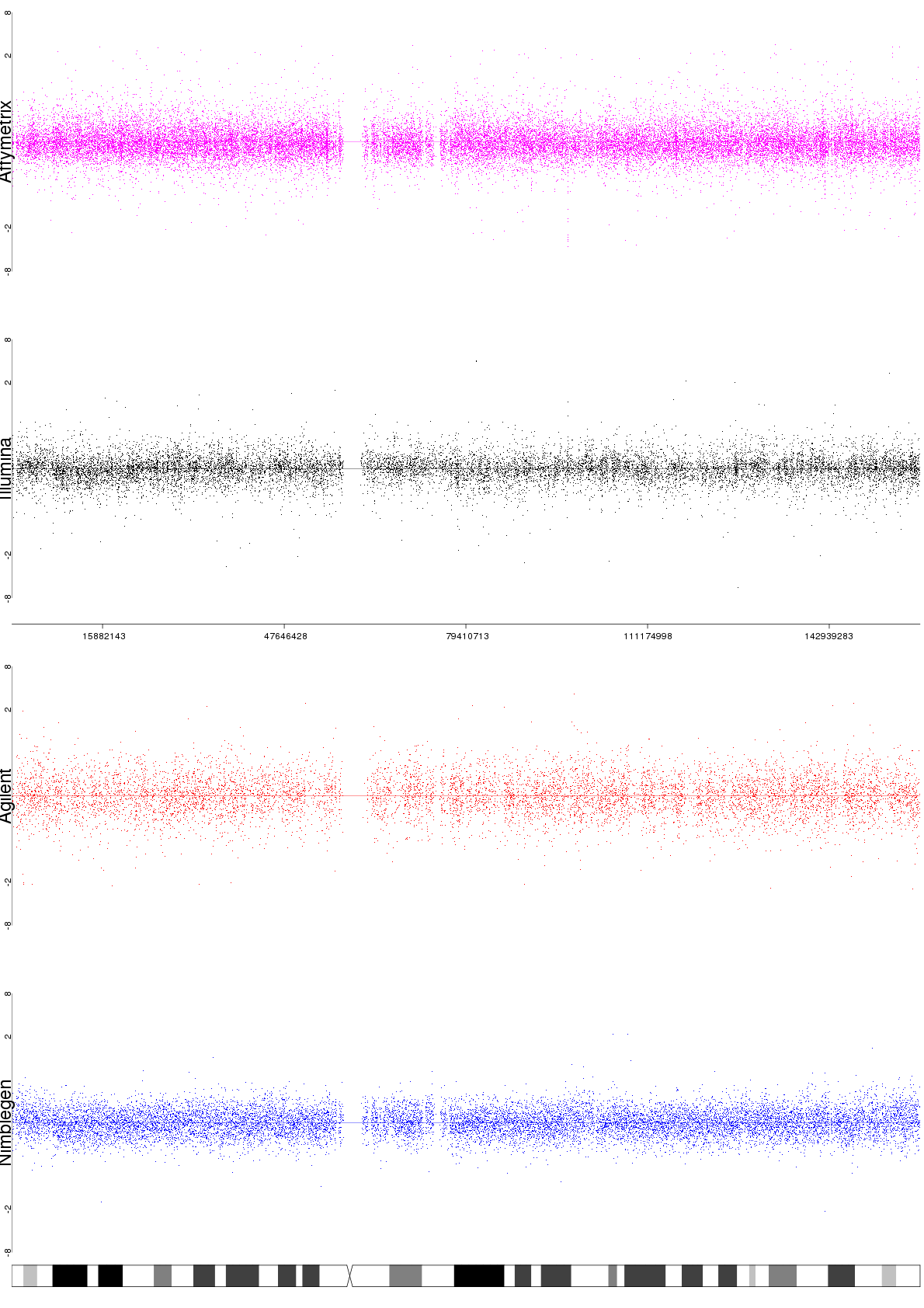

Supplement: Additional file 13 — All sample/chromosome plots for the cell-lines. Zip folder containing PNGs of all whole-chromosome plots for the cell-lines. [file 1471-2164-10-588-S13.ZIP › HapMap/HapMap chromosome 7.png]

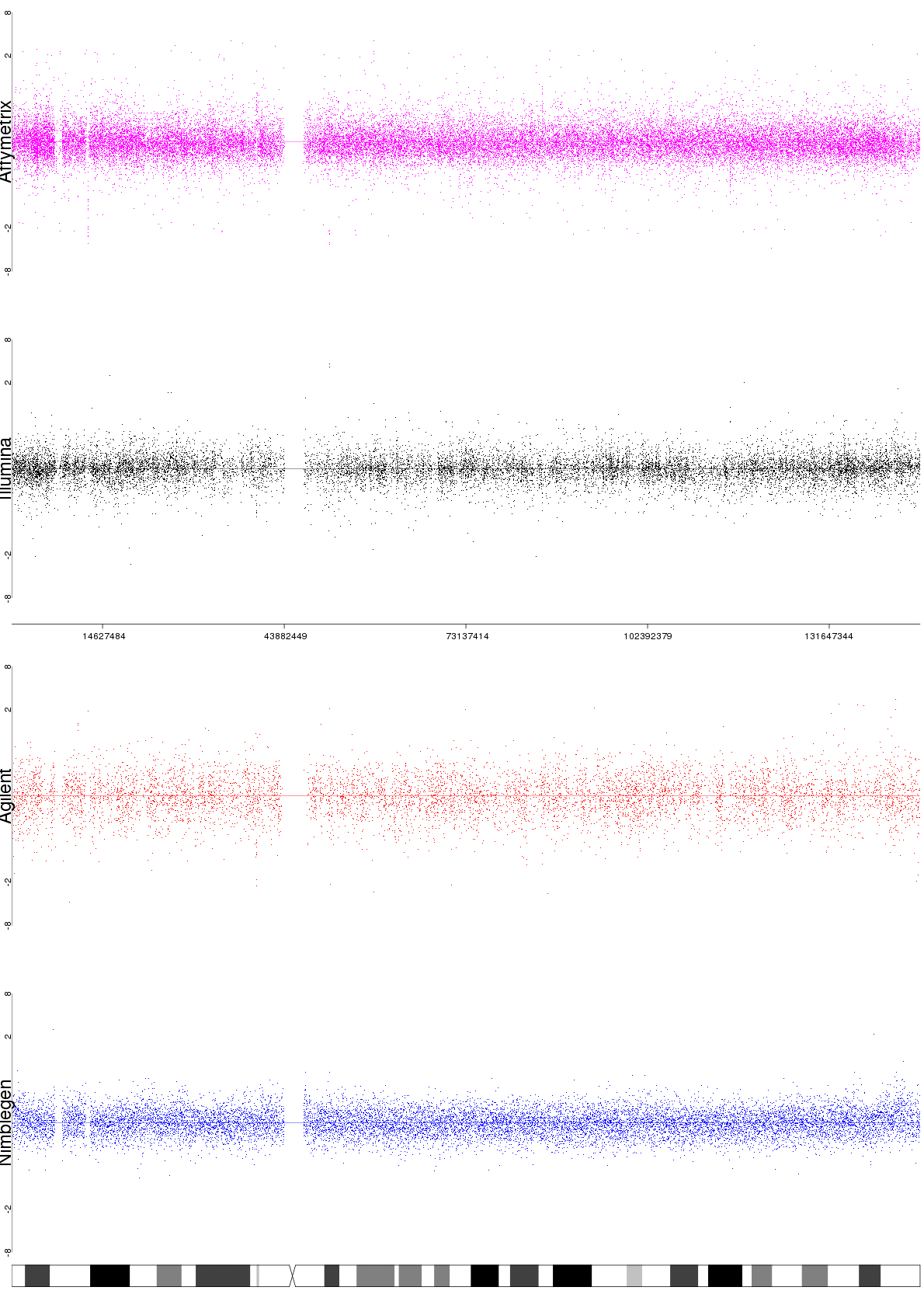

Supplement: Additional file 13 — All sample/chromosome plots for the cell-lines. Zip folder containing PNGs of all whole-chromosome plots for the cell-lines. [file 1471-2164-10-588-S13.ZIP › HapMap/HapMap chromosome 8.png]

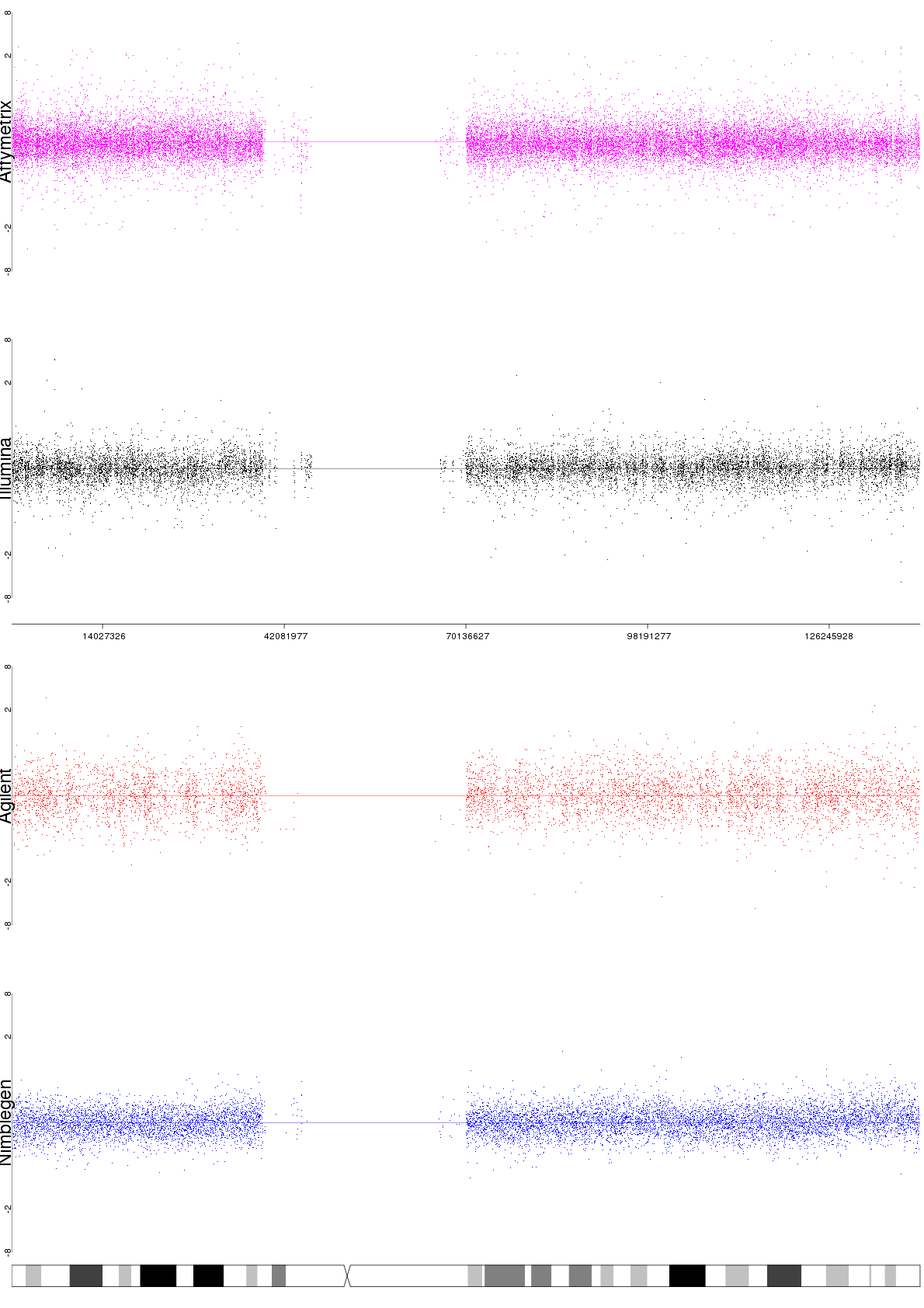

Supplement: Additional file 13 — All sample/chromosome plots for the cell-lines. Zip folder containing PNGs of all whole-chromosome plots for the cell-lines. [file 1471-2164-10-588-S13.ZIP › HapMap/HapMap chromosome 9.png]

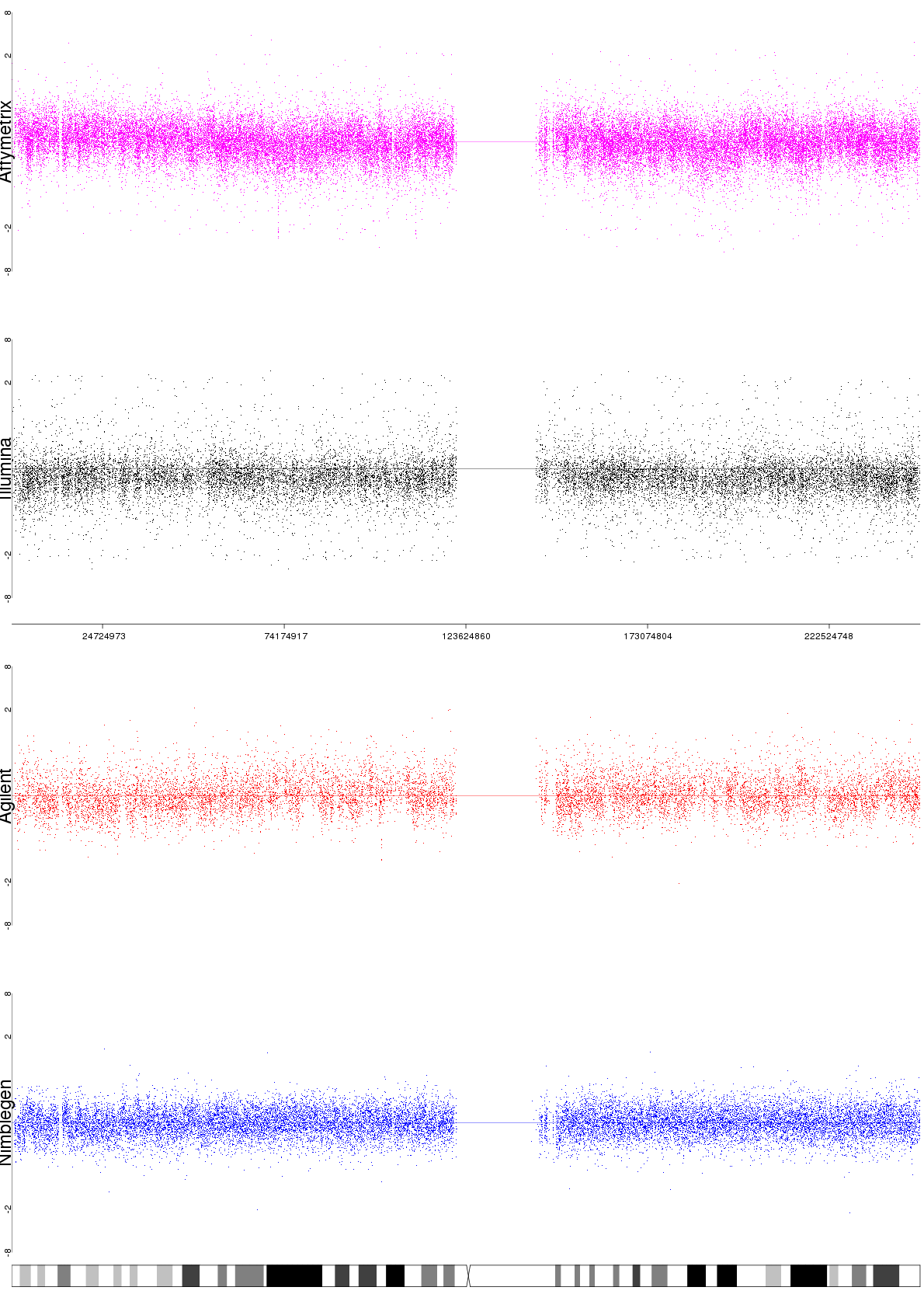

Supplement: Additional file 13 — All sample/chromosome plots for the cell-lines. Zip folder containing PNGs of all whole-chromosome plots for the cell-lines. [file 1471-2164-10-588-S13.ZIP › MT3/MT3 chromosome 1.png]

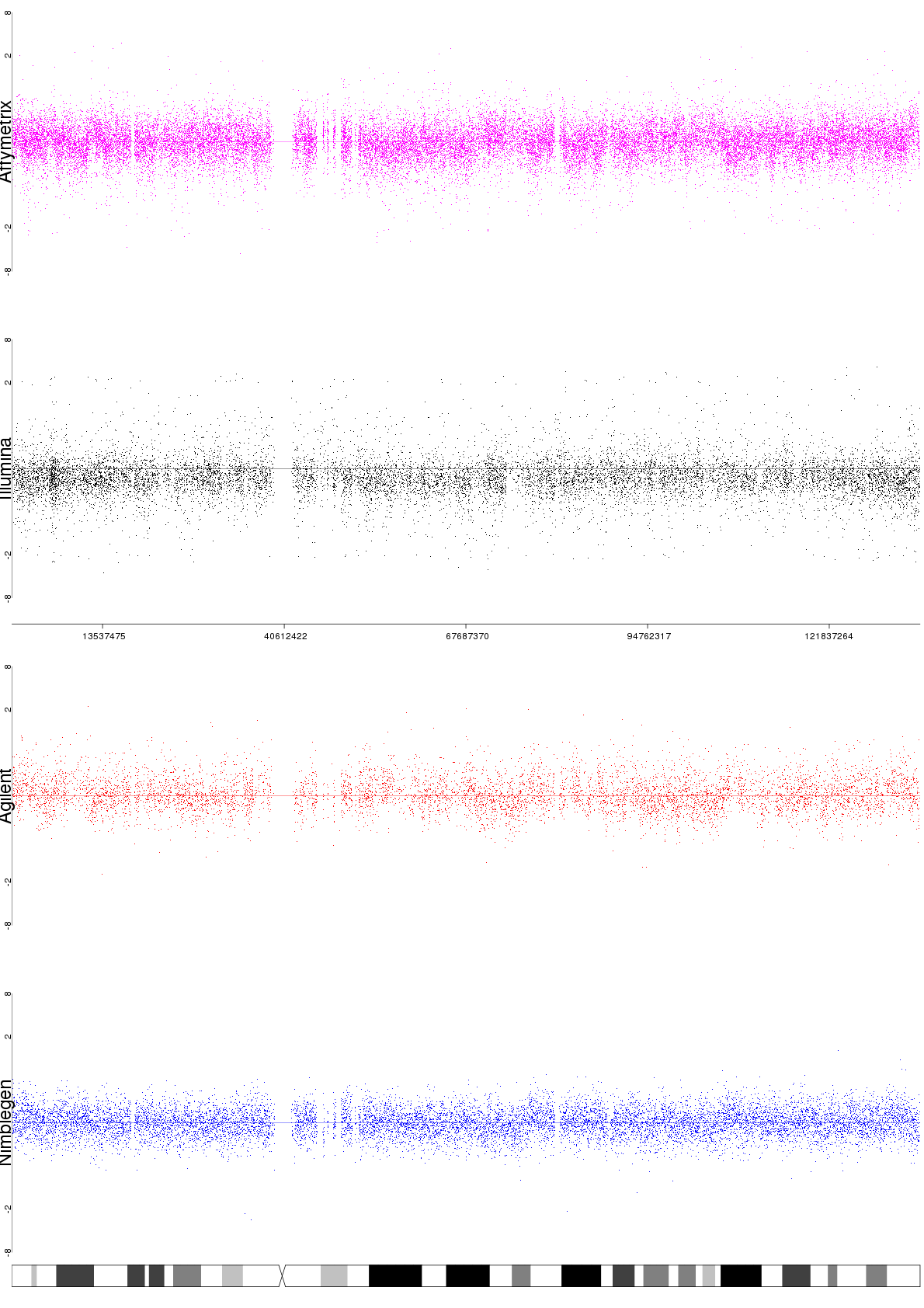

Supplement: Additional file 13 — All sample/chromosome plots for the cell-lines. Zip folder containing PNGs of all whole-chromosome plots for the cell-lines. [file 1471-2164-10-588-S13.ZIP › MT3/MT3 chromosome 10.png]

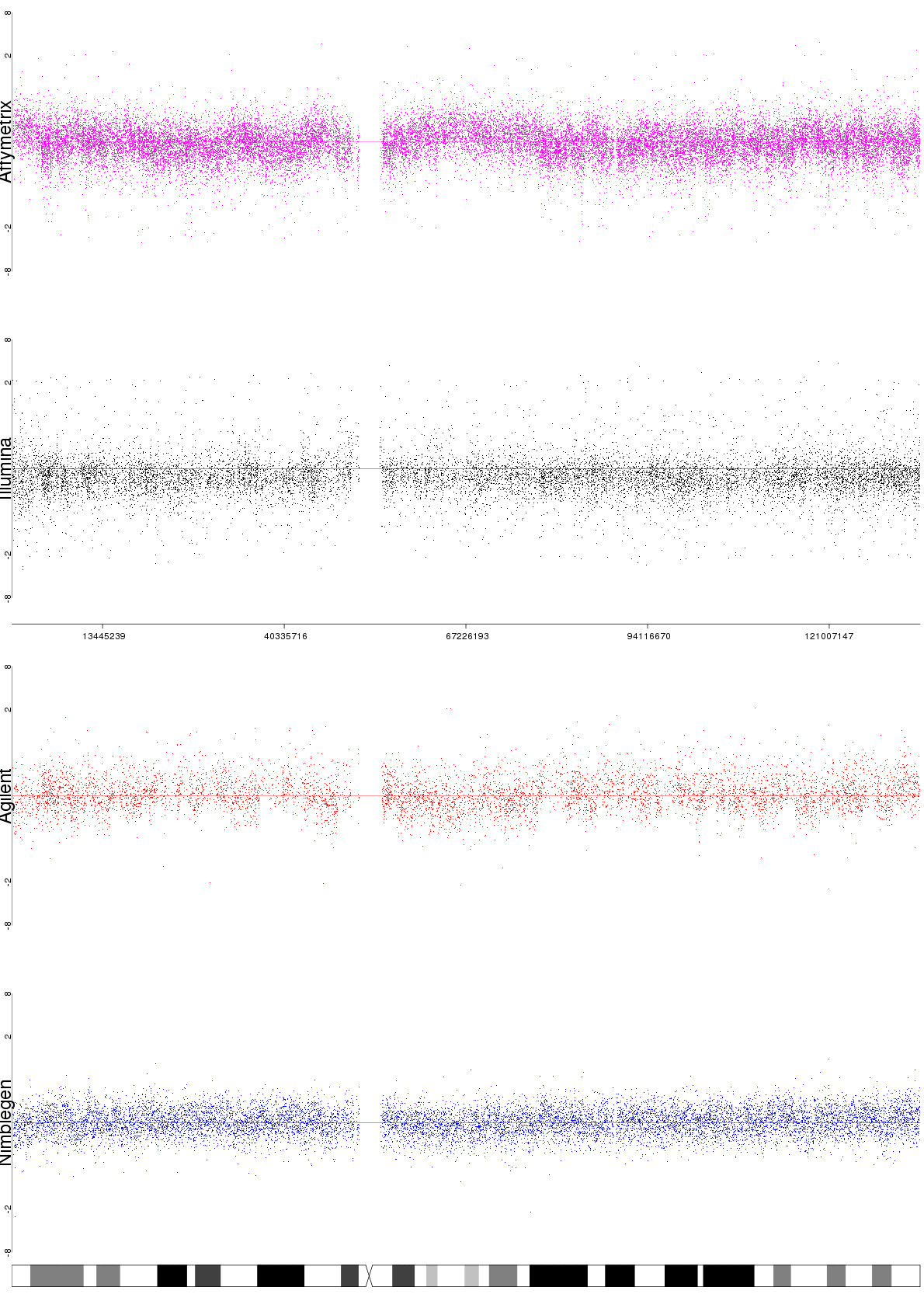

Supplement: Additional file 13 — All sample/chromosome plots for the cell-lines. Zip folder containing PNGs of all whole-chromosome plots for the cell-lines. [file 1471-2164-10-588-S13.ZIP › MT3/MT3 chromosome 11.png]

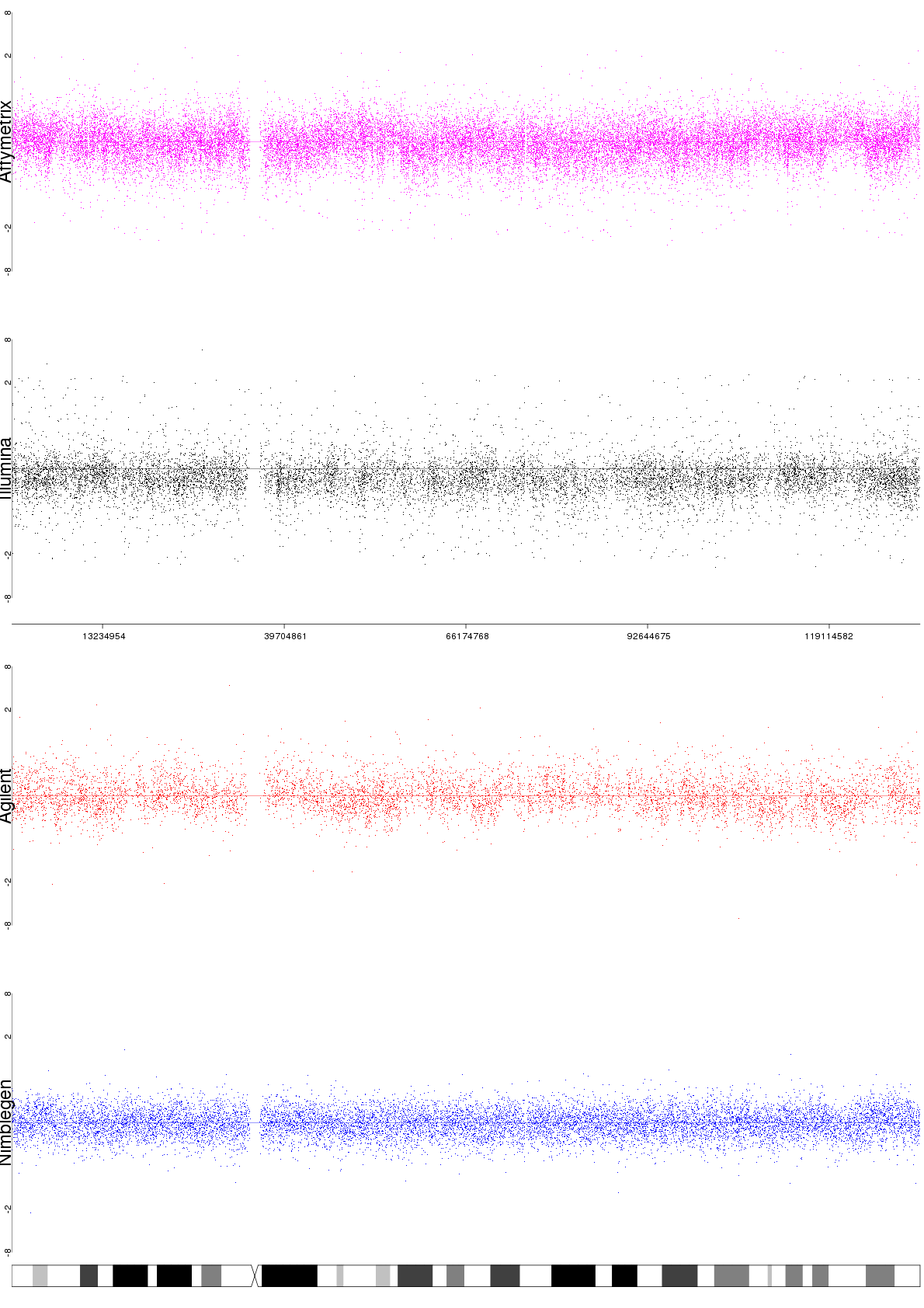

Supplement: Additional file 13 — All sample/chromosome plots for the cell-lines. Zip folder containing PNGs of all whole-chromosome plots for the cell-lines. [file 1471-2164-10-588-S13.ZIP › MT3/MT3 chromosome 12.png]

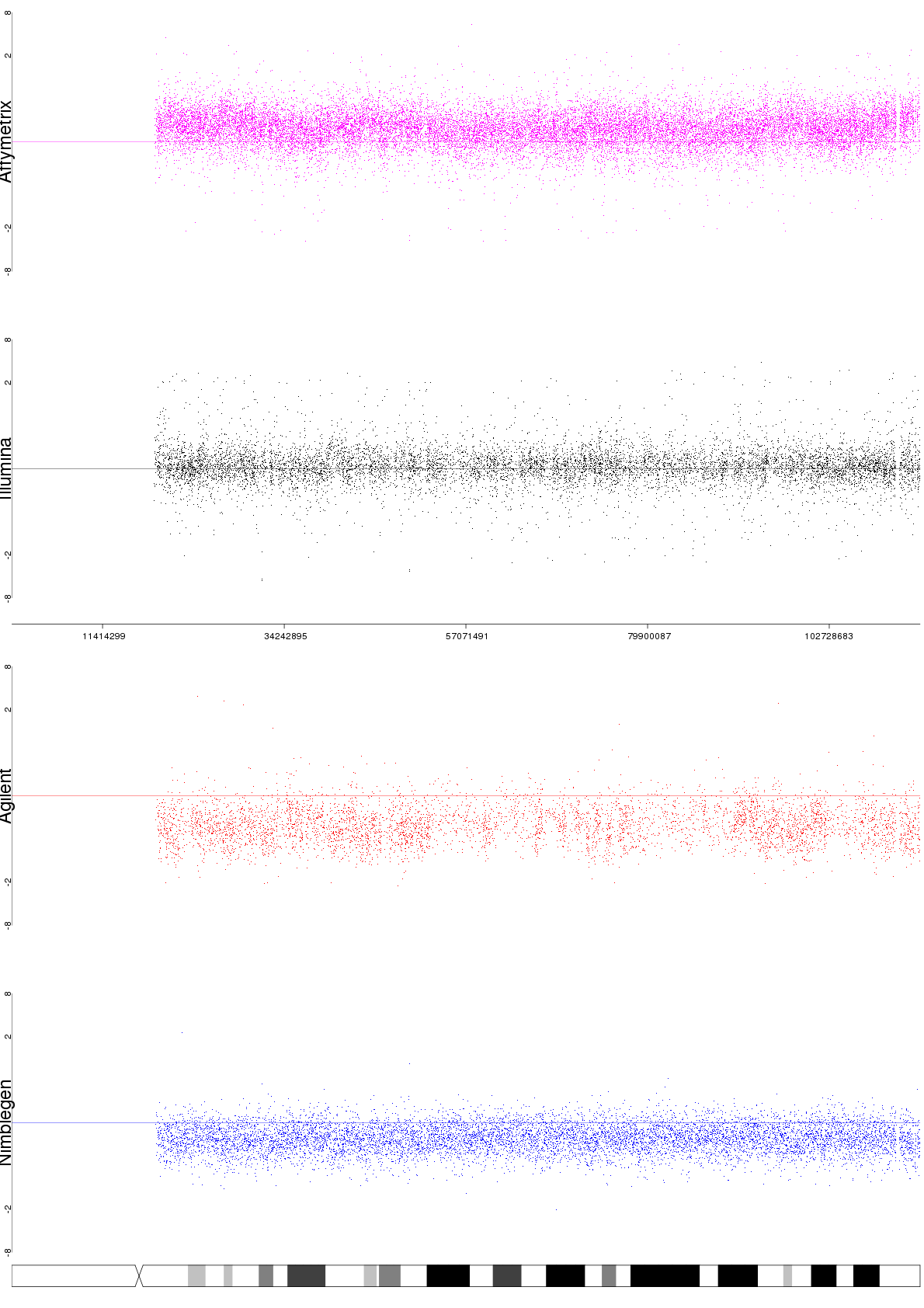

Supplement: Additional file 13 — All sample/chromosome plots for the cell-lines. Zip folder containing PNGs of all whole-chromosome plots for the cell-lines. [file 1471-2164-10-588-S13.ZIP › MT3/MT3 chromosome 13.png]

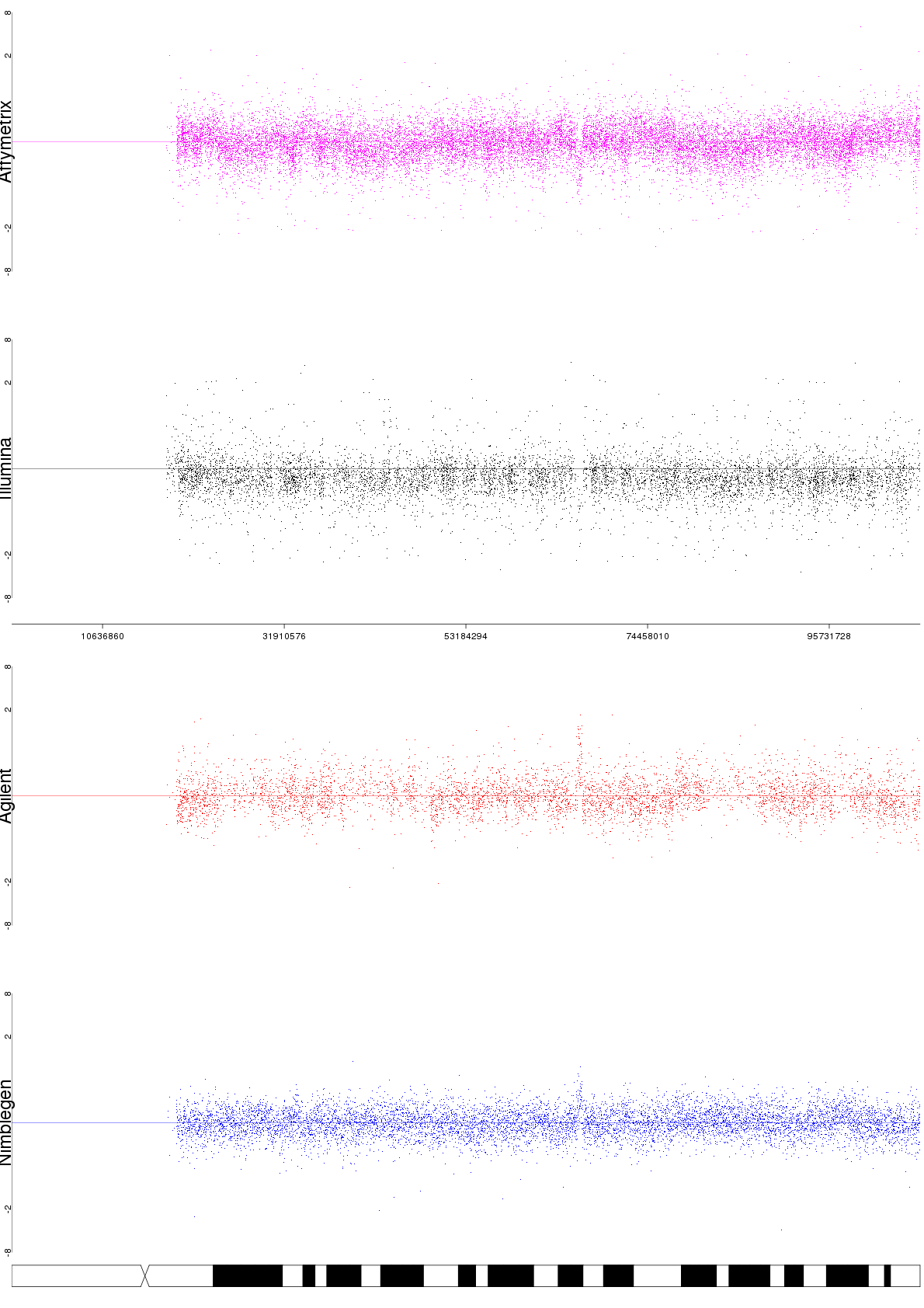

Supplement: Additional file 13 — All sample/chromosome plots for the cell-lines. Zip folder containing PNGs of all whole-chromosome plots for the cell-lines. [file 1471-2164-10-588-S13.ZIP › MT3/MT3 chromosome 14.png]

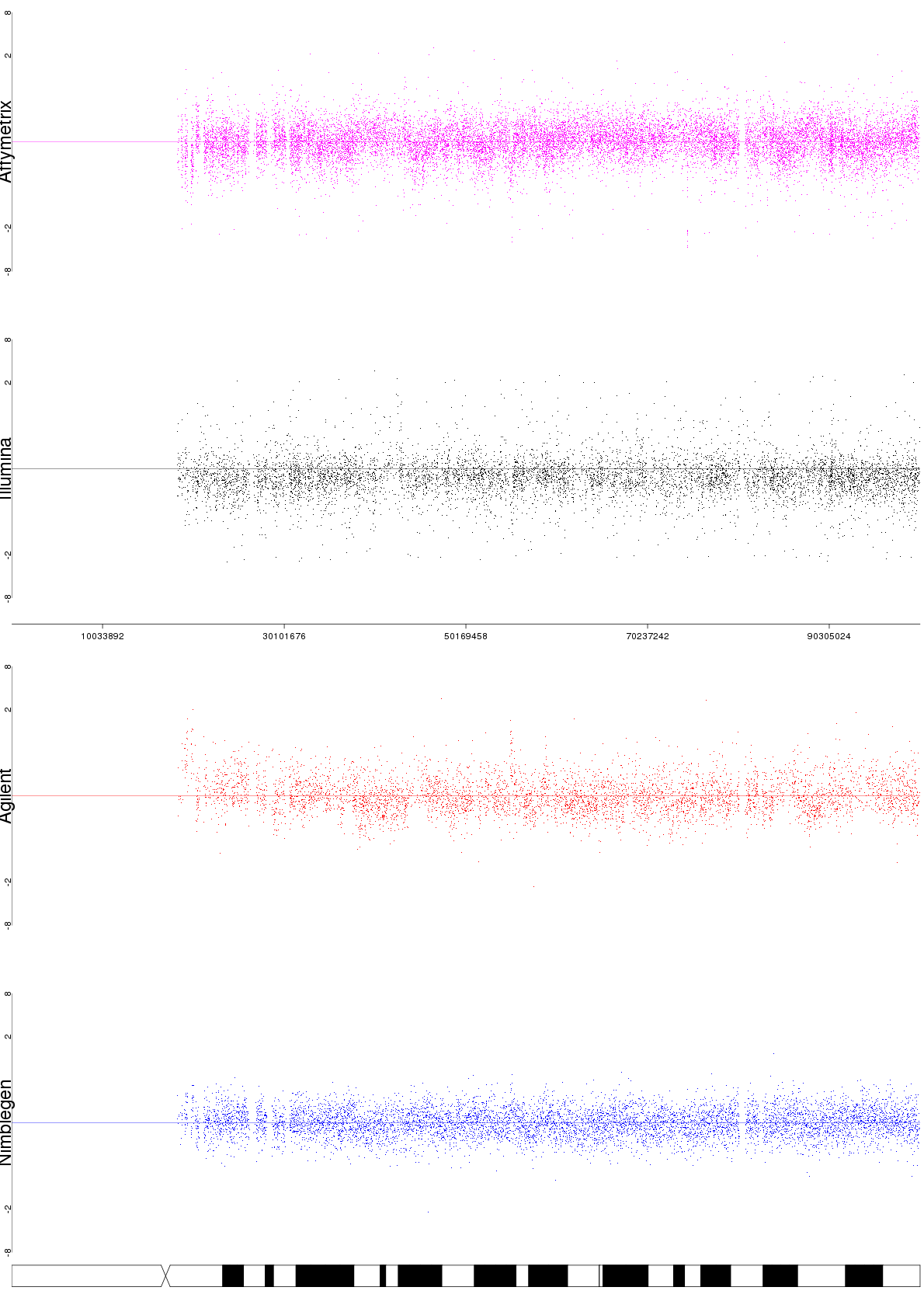

Supplement: Additional file 13 — All sample/chromosome plots for the cell-lines. Zip folder containing PNGs of all whole-chromosome plots for the cell-lines. [file 1471-2164-10-588-S13.ZIP › MT3/MT3 chromosome 15.png]

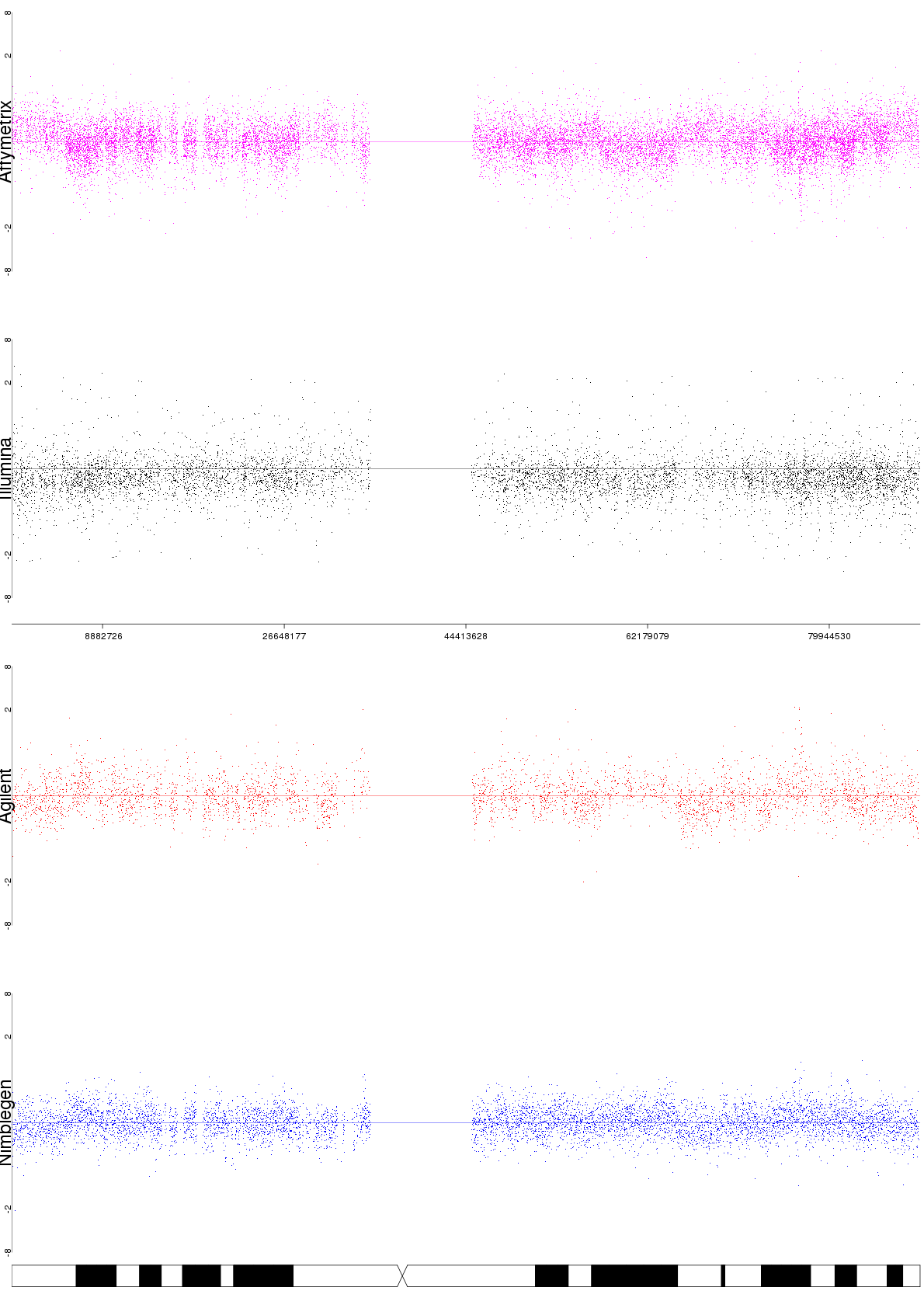

Supplement: Additional file 13 — All sample/chromosome plots for the cell-lines. Zip folder containing PNGs of all whole-chromosome plots for the cell-lines. [file 1471-2164-10-588-S13.ZIP › MT3/MT3 chromosome 16.png]

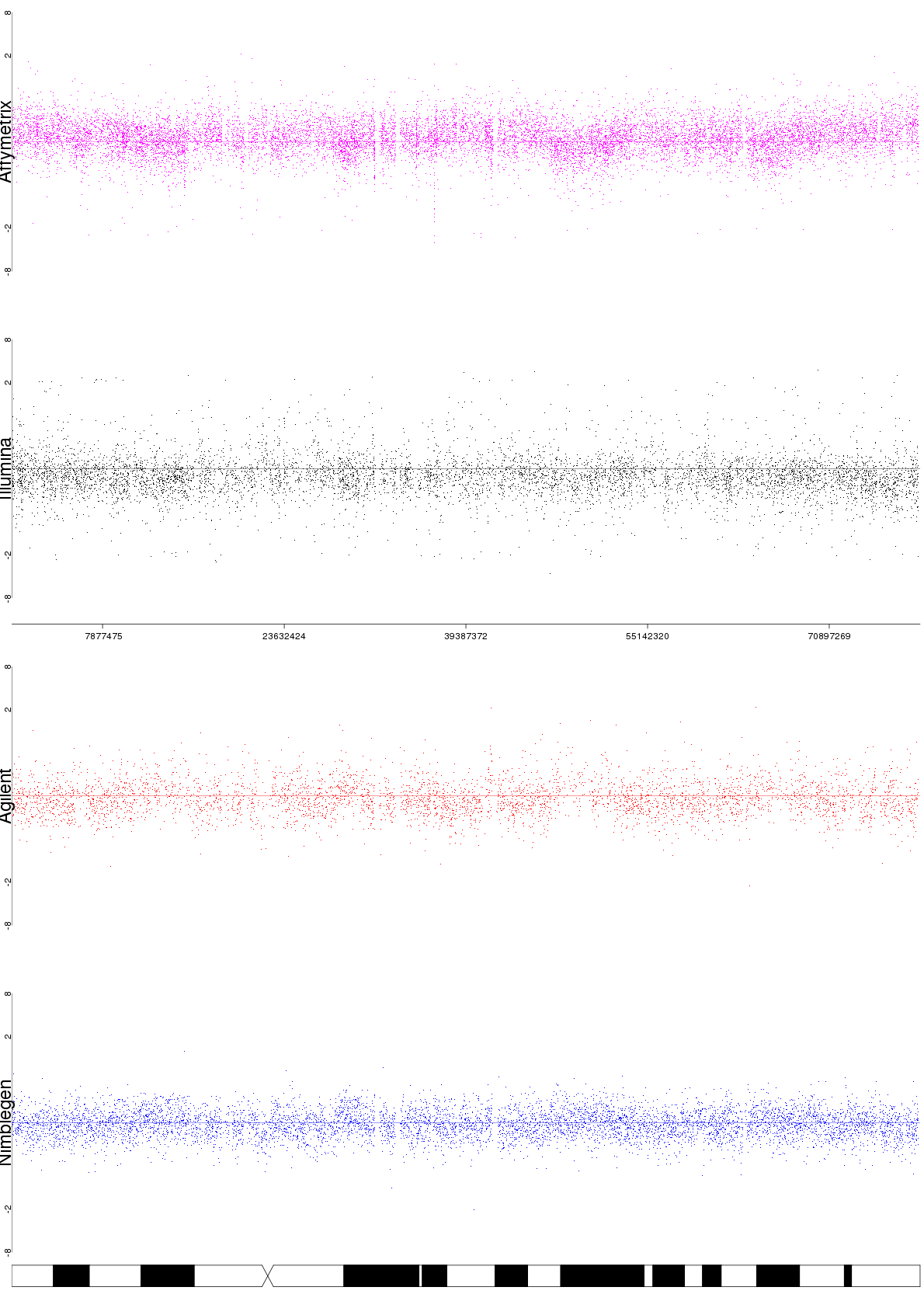

Supplement: Additional file 13 — All sample/chromosome plots for the cell-lines. Zip folder containing PNGs of all whole-chromosome plots for the cell-lines. [file 1471-2164-10-588-S13.ZIP › MT3/MT3 chromosome 17.png]

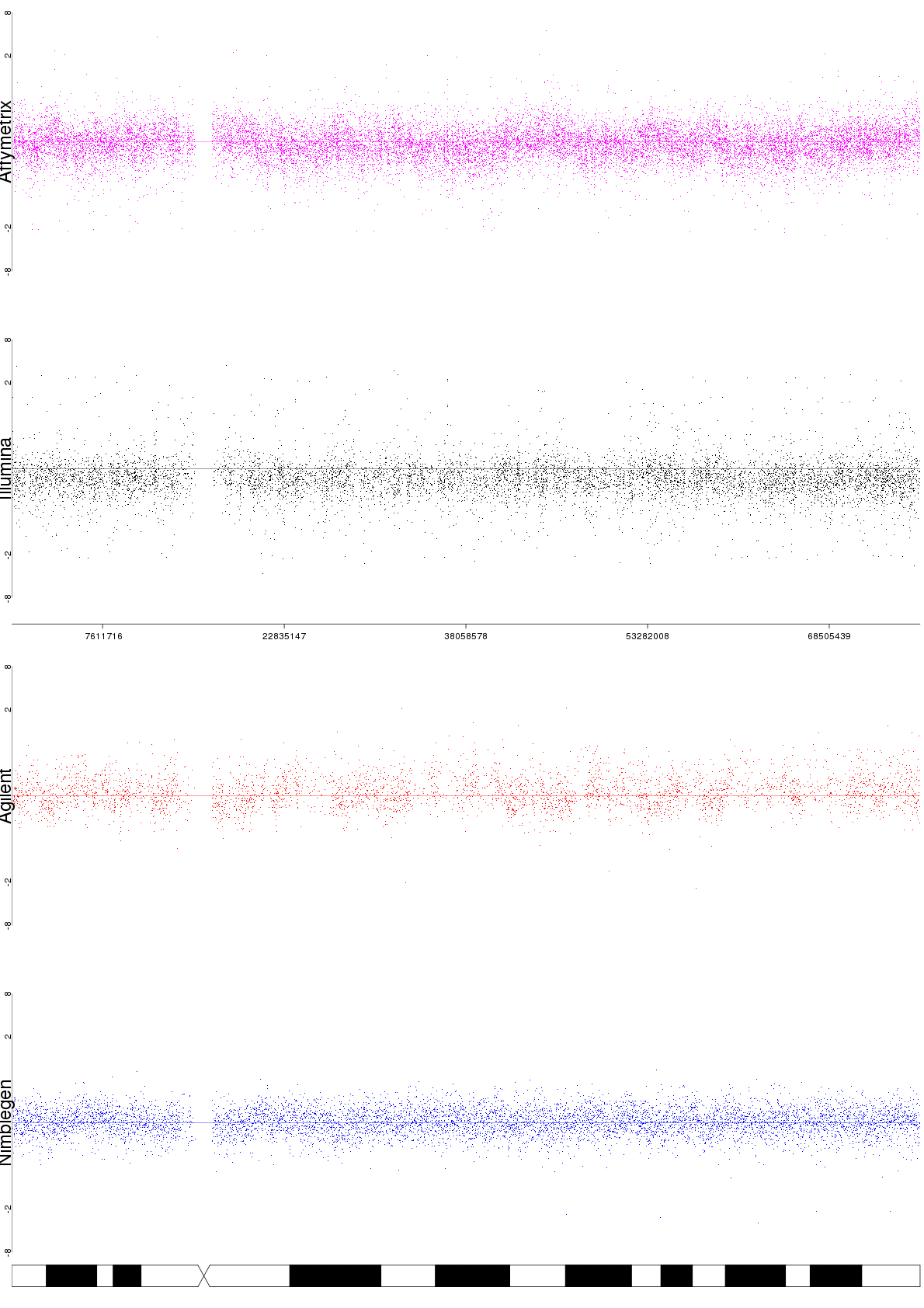

Supplement: Additional file 13 — All sample/chromosome plots for the cell-lines. Zip folder containing PNGs of all whole-chromosome plots for the cell-lines. [file 1471-2164-10-588-S13.ZIP › MT3/MT3 chromosome 18.png]

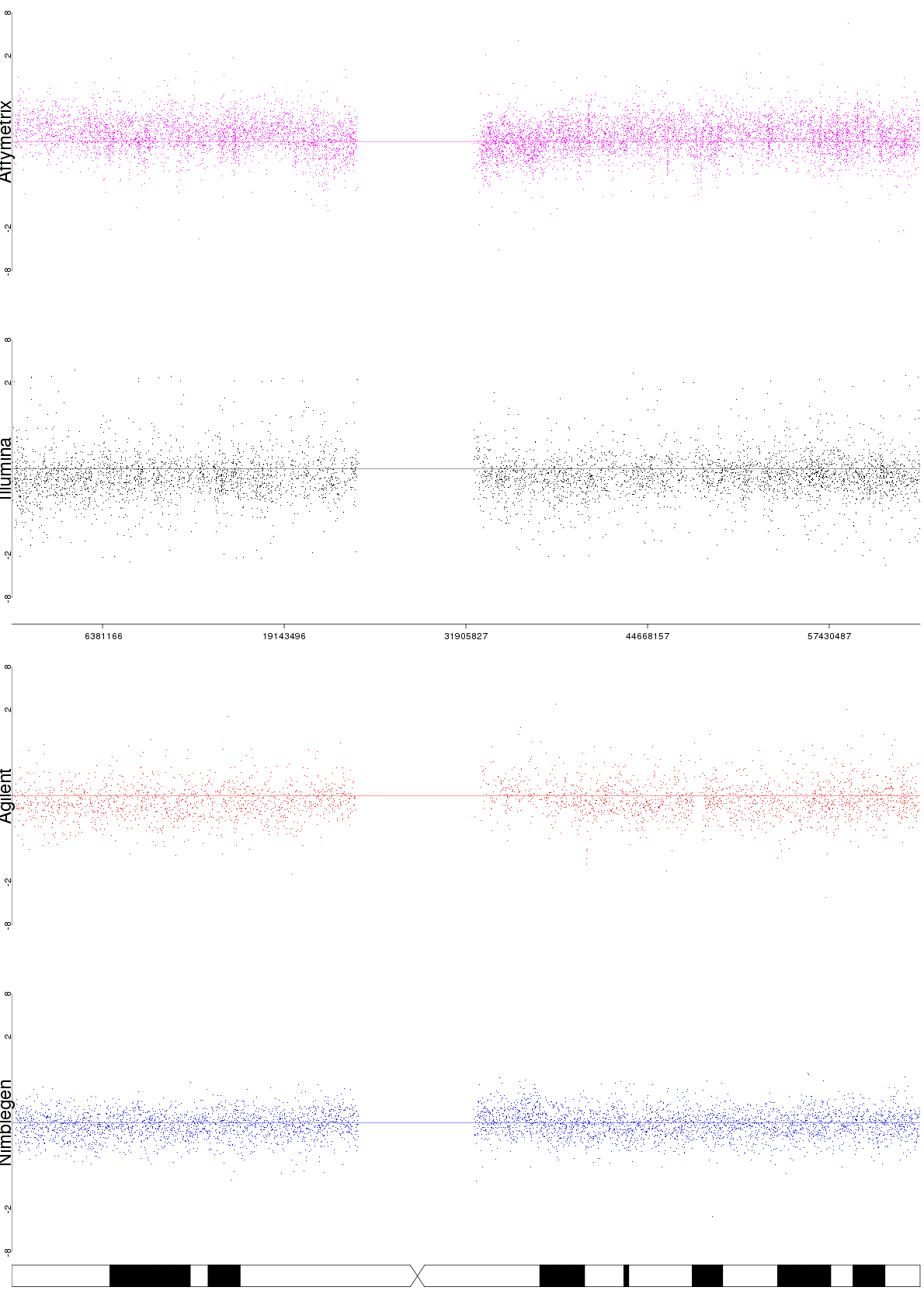

Supplement: Additional file 13 — All sample/chromosome plots for the cell-lines. Zip folder containing PNGs of all whole-chromosome plots for the cell-lines. [file 1471-2164-10-588-S13.ZIP › MT3/MT3 chromosome 19.png]

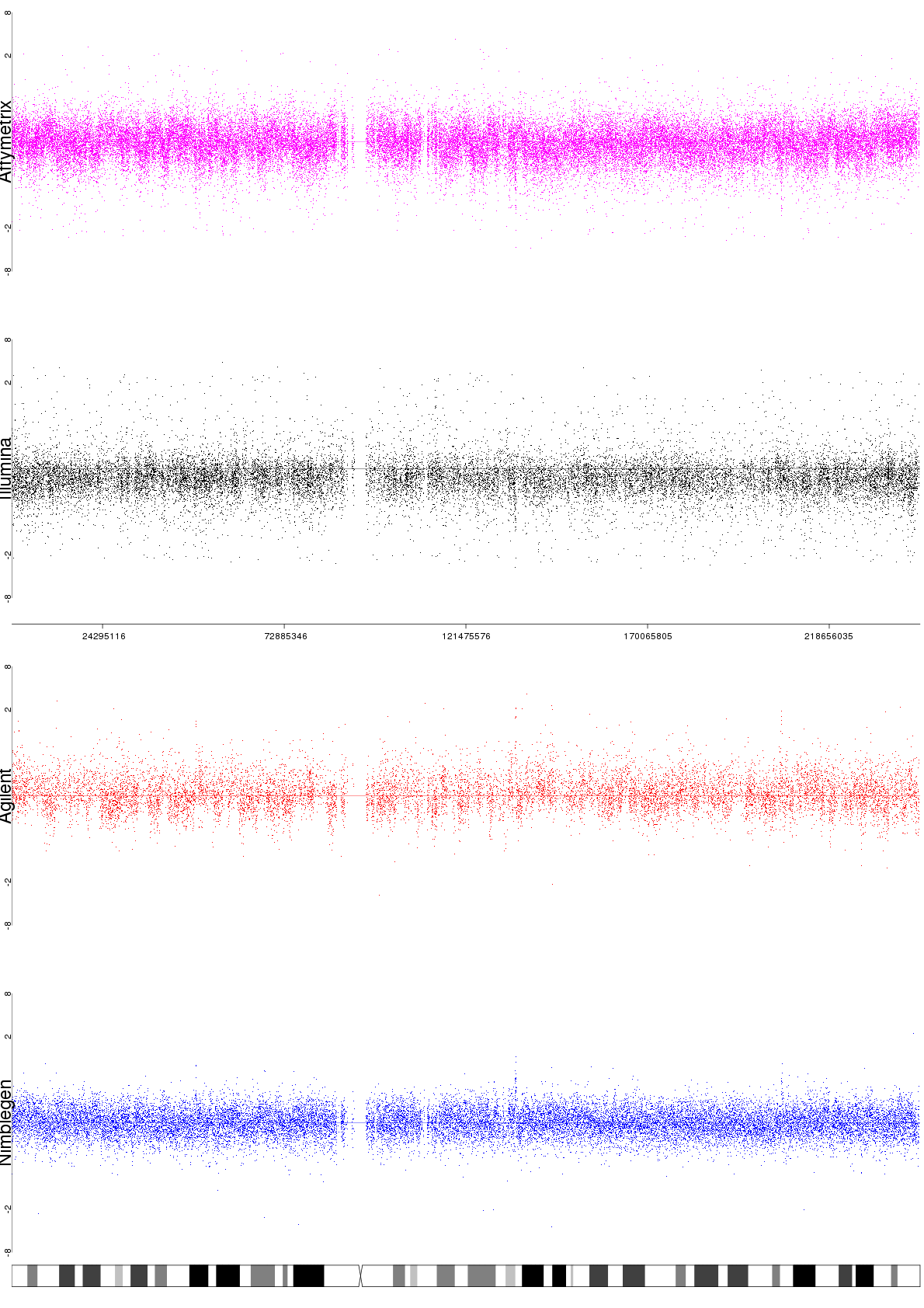

Supplement: Additional file 13 — All sample/chromosome plots for the cell-lines. Zip folder containing PNGs of all whole-chromosome plots for the cell-lines. [file 1471-2164-10-588-S13.ZIP › MT3/MT3 chromosome 2.png]

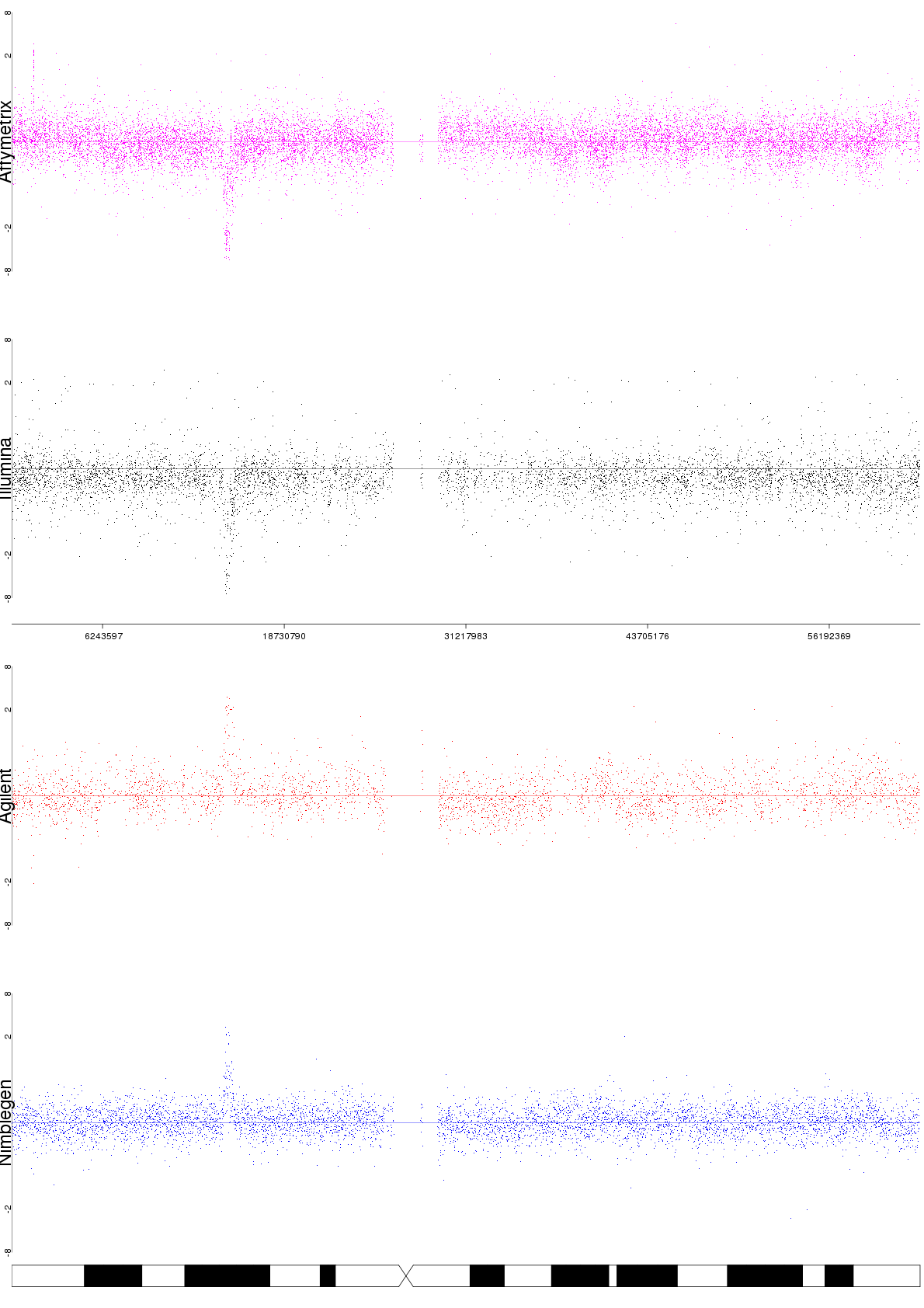

Supplement: Additional file 13 — All sample/chromosome plots for the cell-lines. Zip folder containing PNGs of all whole-chromosome plots for the cell-lines. [file 1471-2164-10-588-S13.ZIP › MT3/MT3 chromosome 20.png]

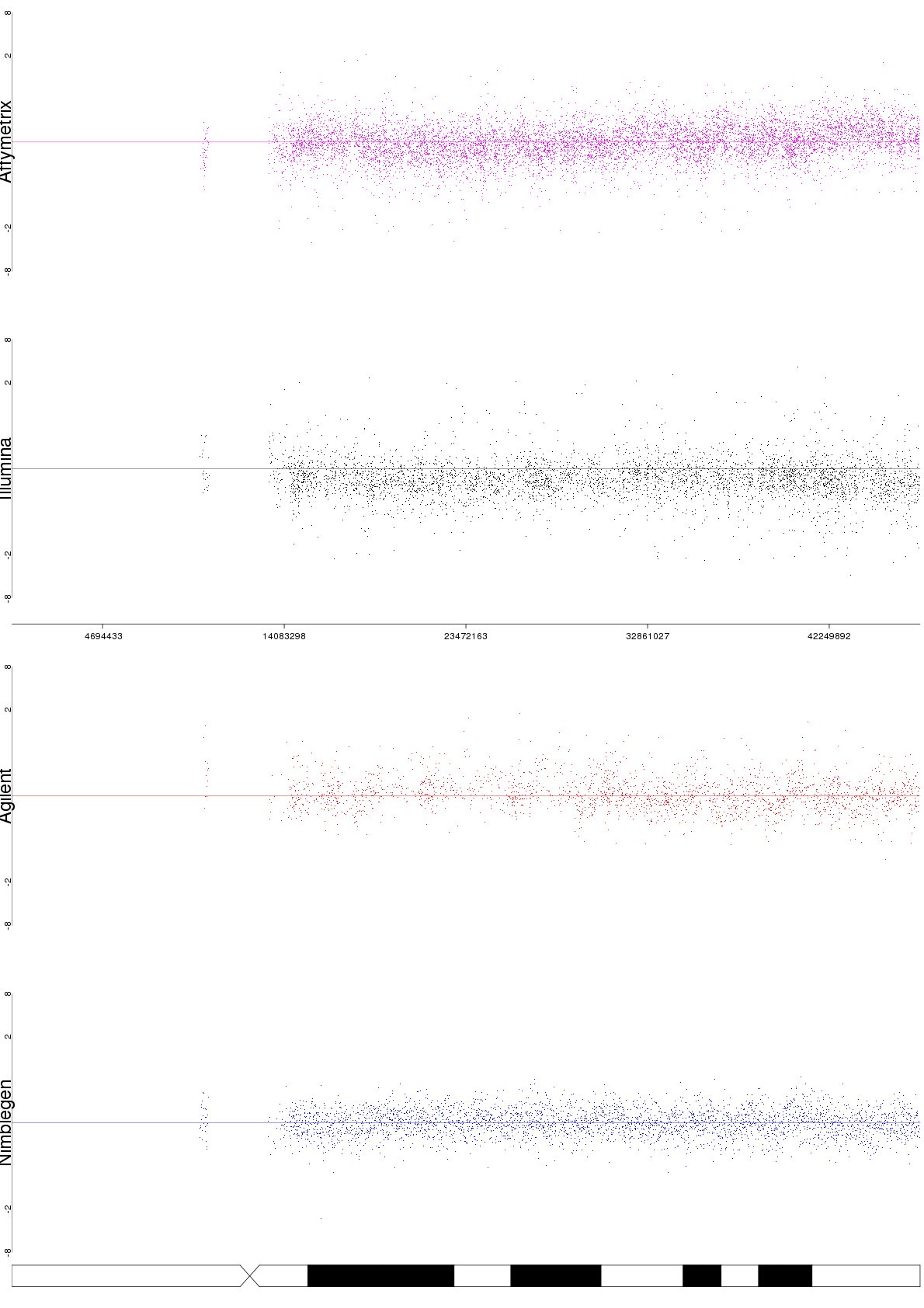

Supplement: Additional file 13 — All sample/chromosome plots for the cell-lines. Zip folder containing PNGs of all whole-chromosome plots for the cell-lines. [file 1471-2164-10-588-S13.ZIP › MT3/MT3 chromosome 21.png]

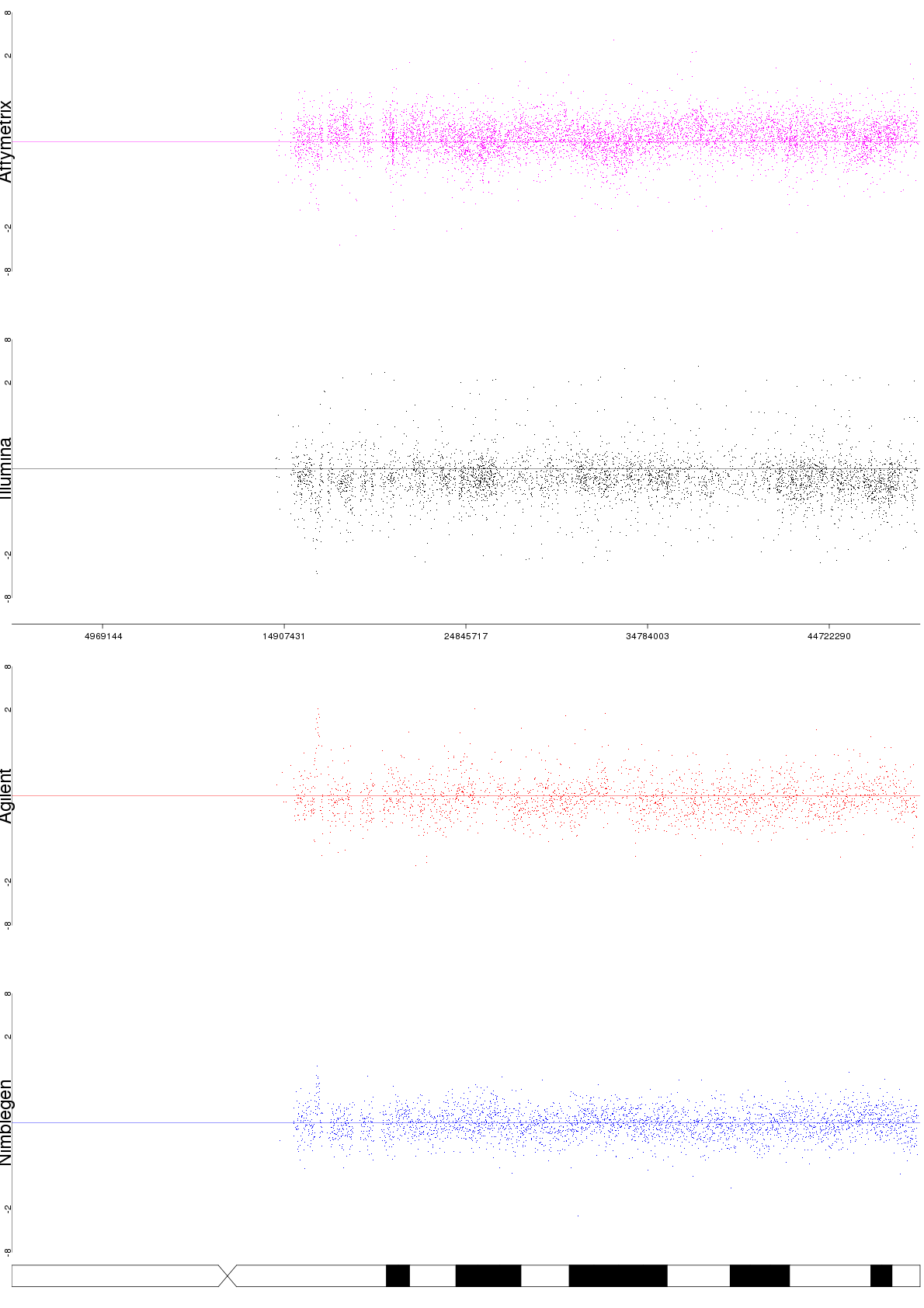

Supplement: Additional file 13 — All sample/chromosome plots for the cell-lines. Zip folder containing PNGs of all whole-chromosome plots for the cell-lines. [file 1471-2164-10-588-S13.ZIP › MT3/MT3 chromosome 22.png]

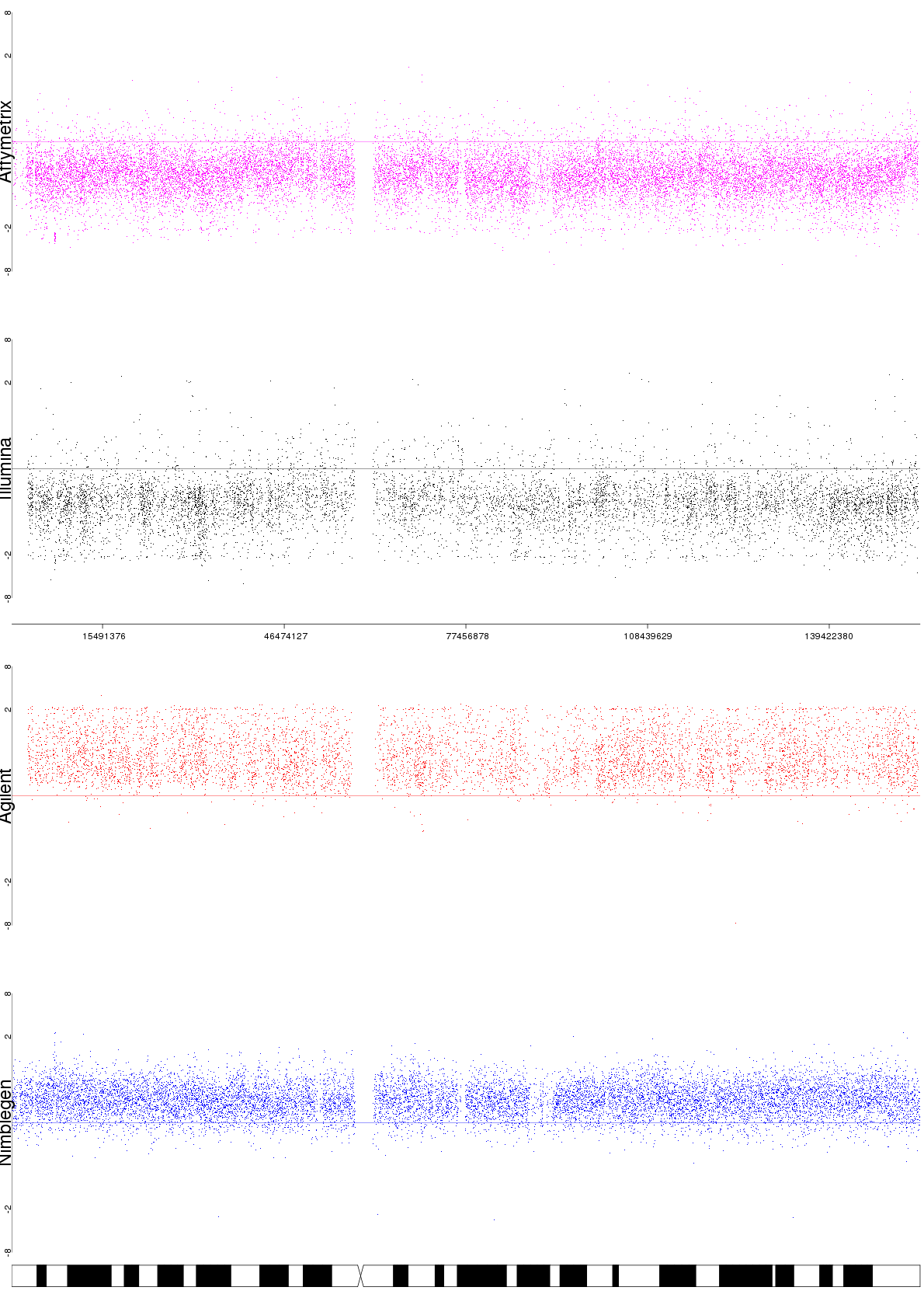

Supplement: Additional file 13 — All sample/chromosome plots for the cell-lines. Zip folder containing PNGs of all whole-chromosome plots for the cell-lines. [file 1471-2164-10-588-S13.ZIP › MT3/MT3 chromosome 23.png]

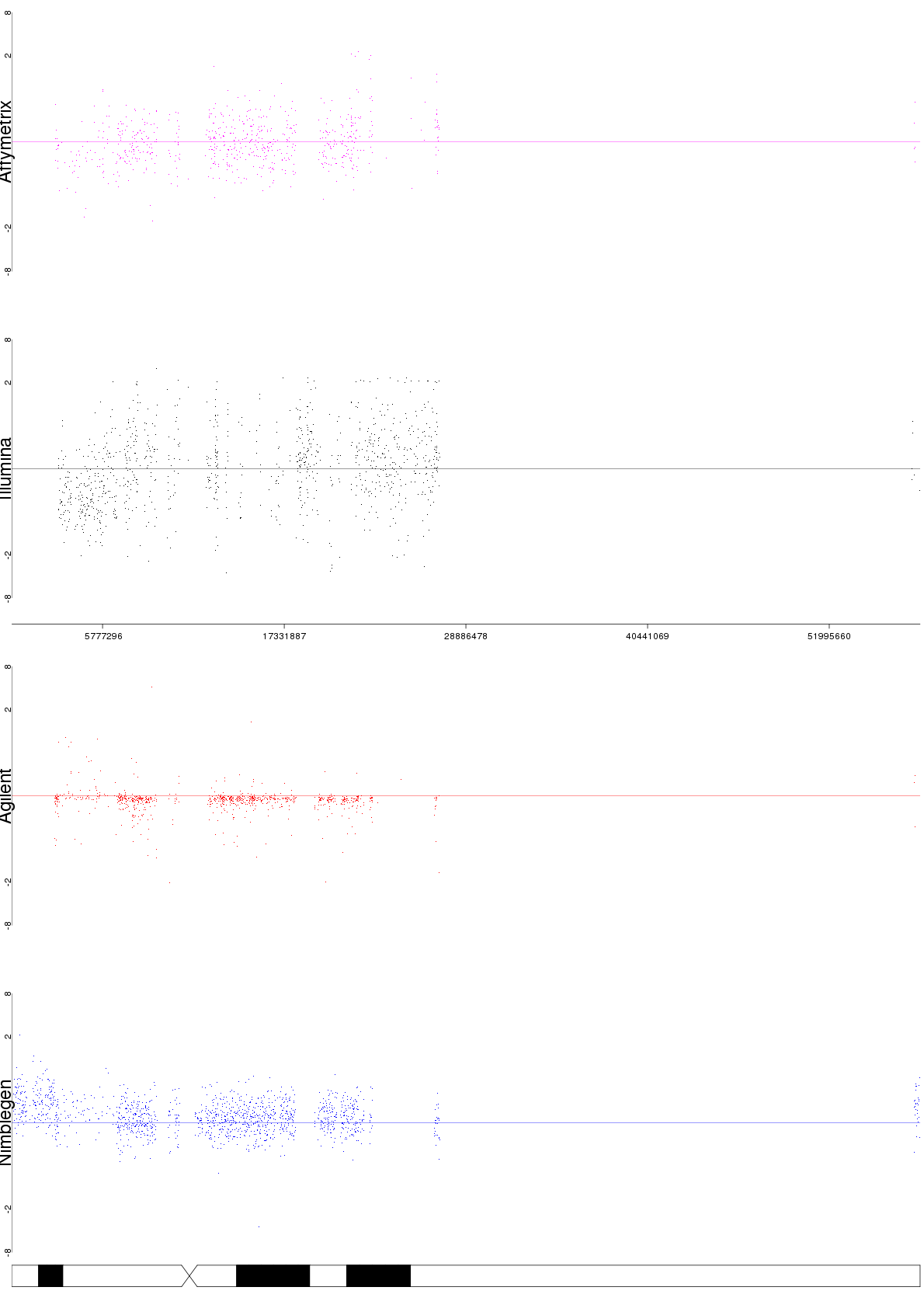

Supplement: Additional file 13 — All sample/chromosome plots for the cell-lines. Zip folder containing PNGs of all whole-chromosome plots for the cell-lines. [file 1471-2164-10-588-S13.ZIP › MT3/MT3 chromosome 24.png]

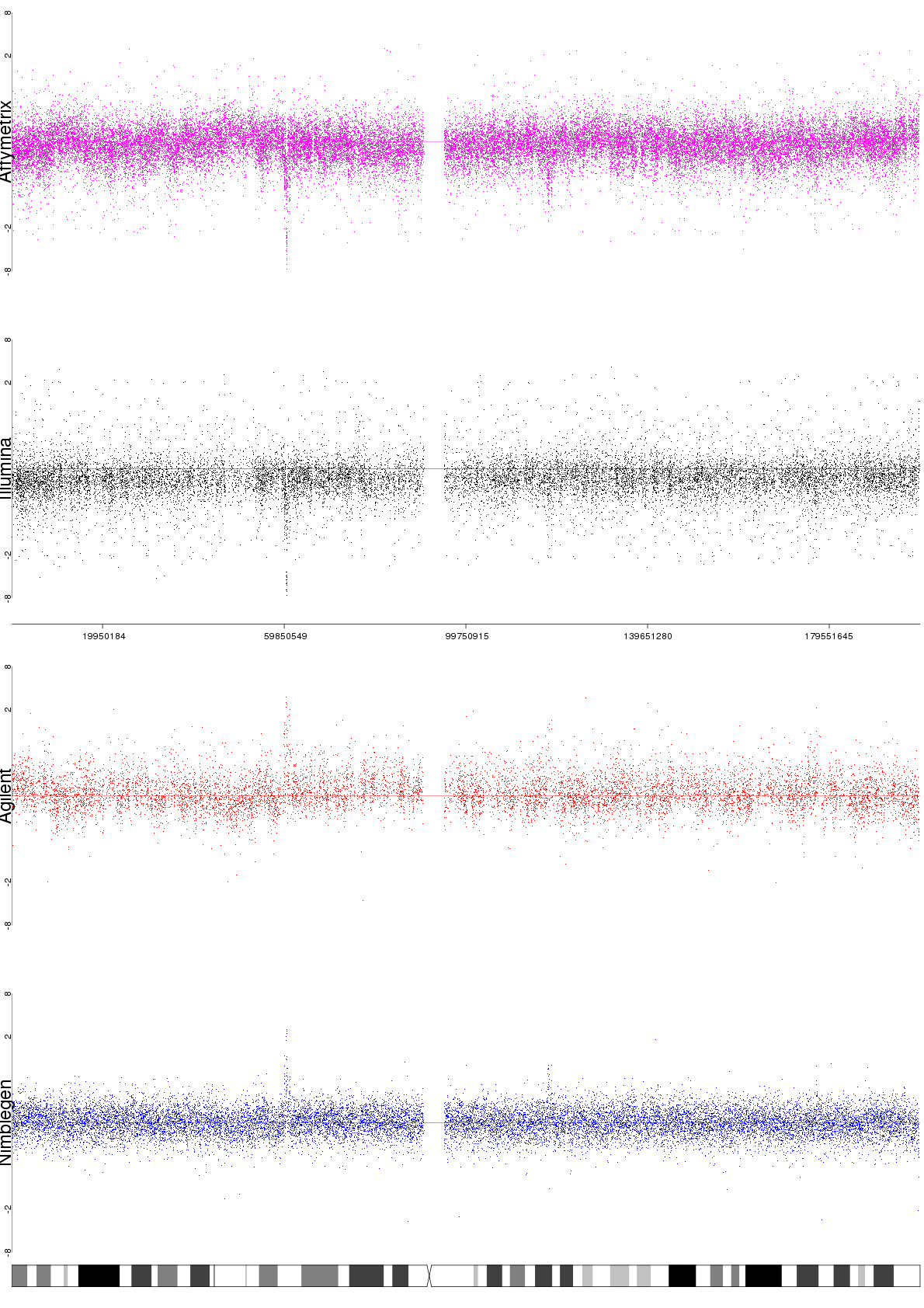

Supplement: Additional file 13 — All sample/chromosome plots for the cell-lines. Zip folder containing PNGs of all whole-chromosome plots for the cell-lines. [file 1471-2164-10-588-S13.ZIP › MT3/MT3 chromosome 3.png]

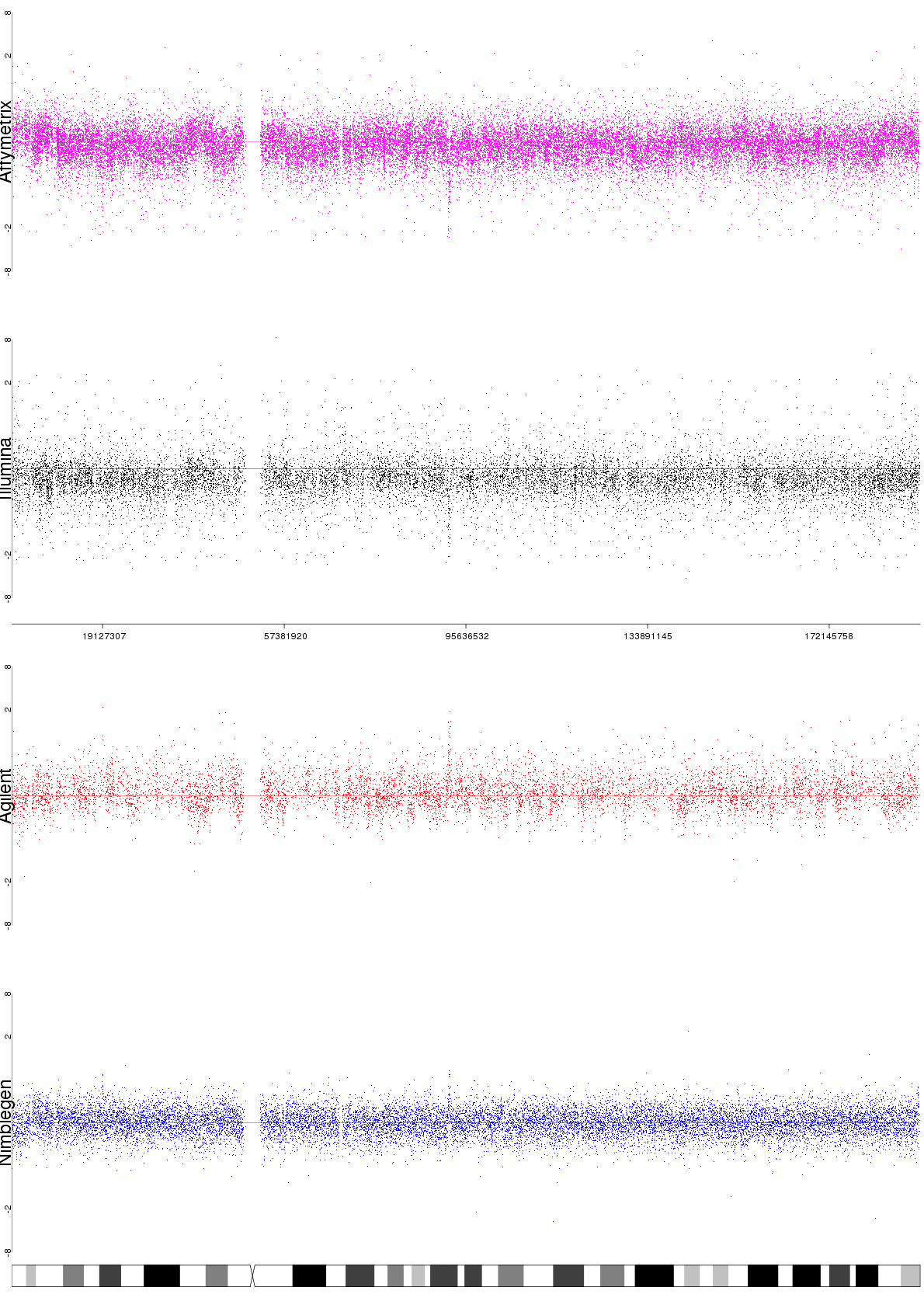

Supplement: Additional file 13 — All sample/chromosome plots for the cell-lines. Zip folder containing PNGs of all whole-chromosome plots for the cell-lines. [file 1471-2164-10-588-S13.ZIP › MT3/MT3 chromosome 4.png]

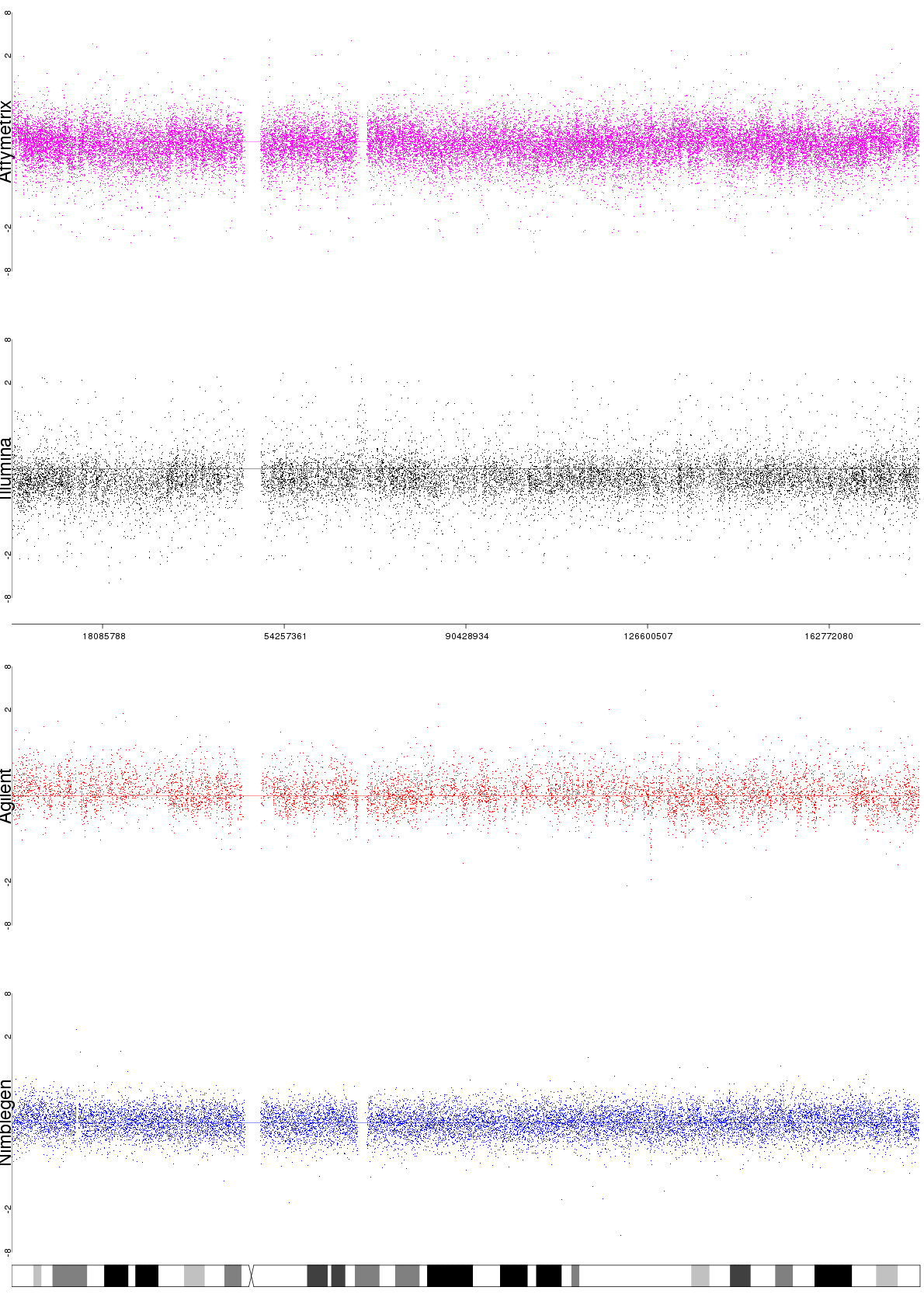

Supplement: Additional file 13 — All sample/chromosome plots for the cell-lines. Zip folder containing PNGs of all whole-chromosome plots for the cell-lines. [file 1471-2164-10-588-S13.ZIP › MT3/MT3 chromosome 5.png]

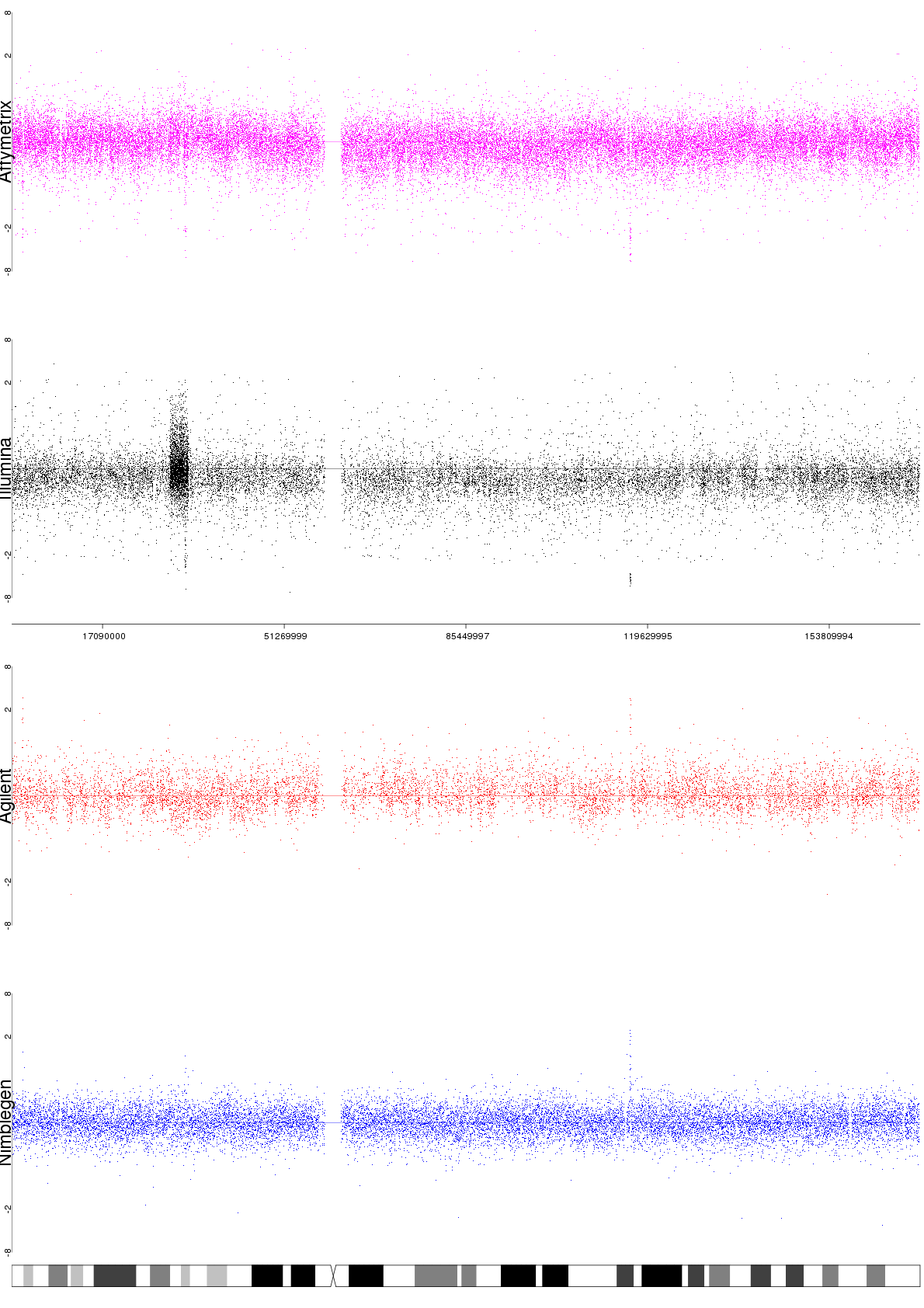

Supplement: Additional file 13 — All sample/chromosome plots for the cell-lines. Zip folder containing PNGs of all whole-chromosome plots for the cell-lines. [file 1471-2164-10-588-S13.ZIP › MT3/MT3 chromosome 6.png]

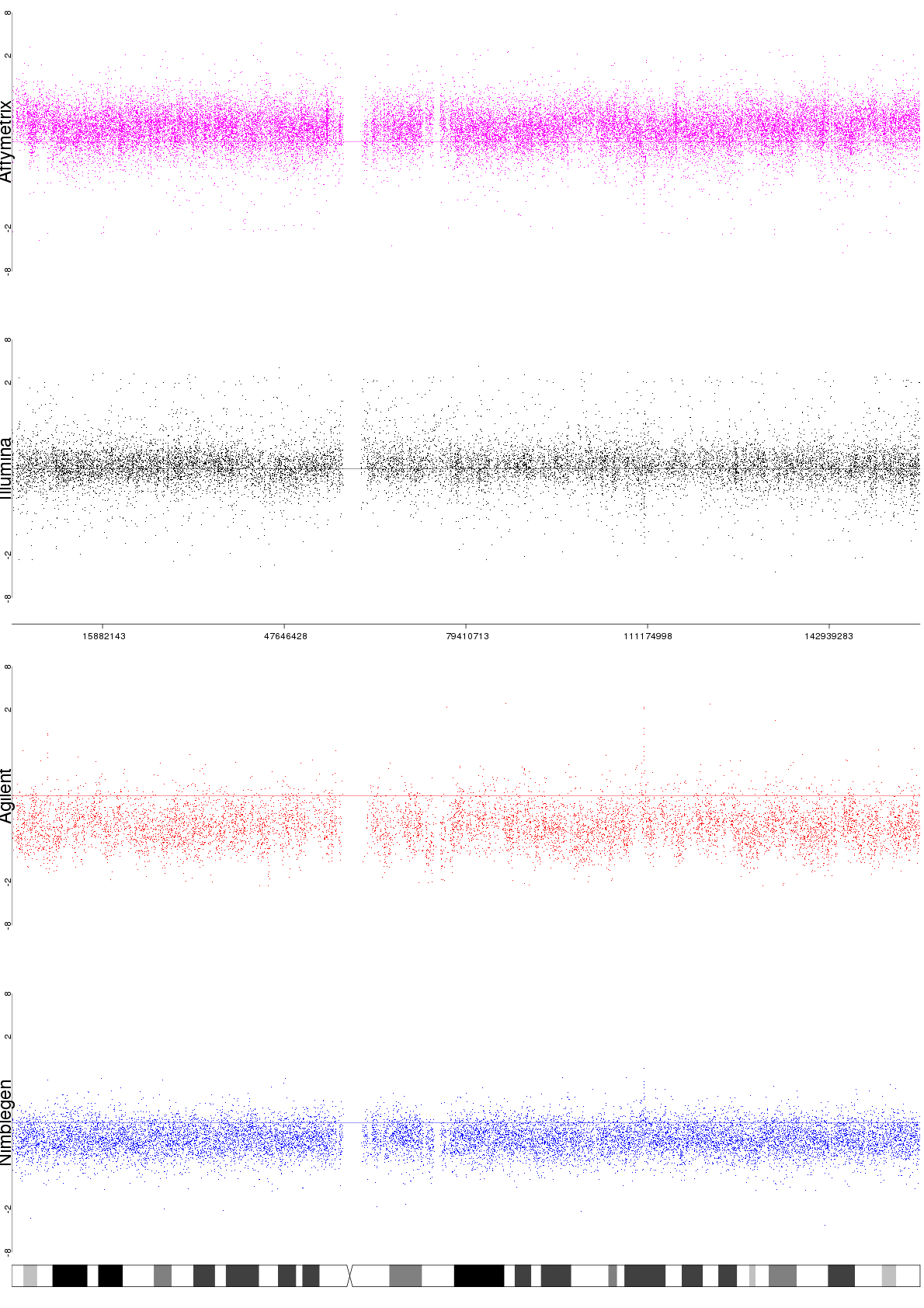

Supplement: Additional file 13 — All sample/chromosome plots for the cell-lines. Zip folder containing PNGs of all whole-chromosome plots for the cell-lines. [file 1471-2164-10-588-S13.ZIP › MT3/MT3 chromosome 7.png]

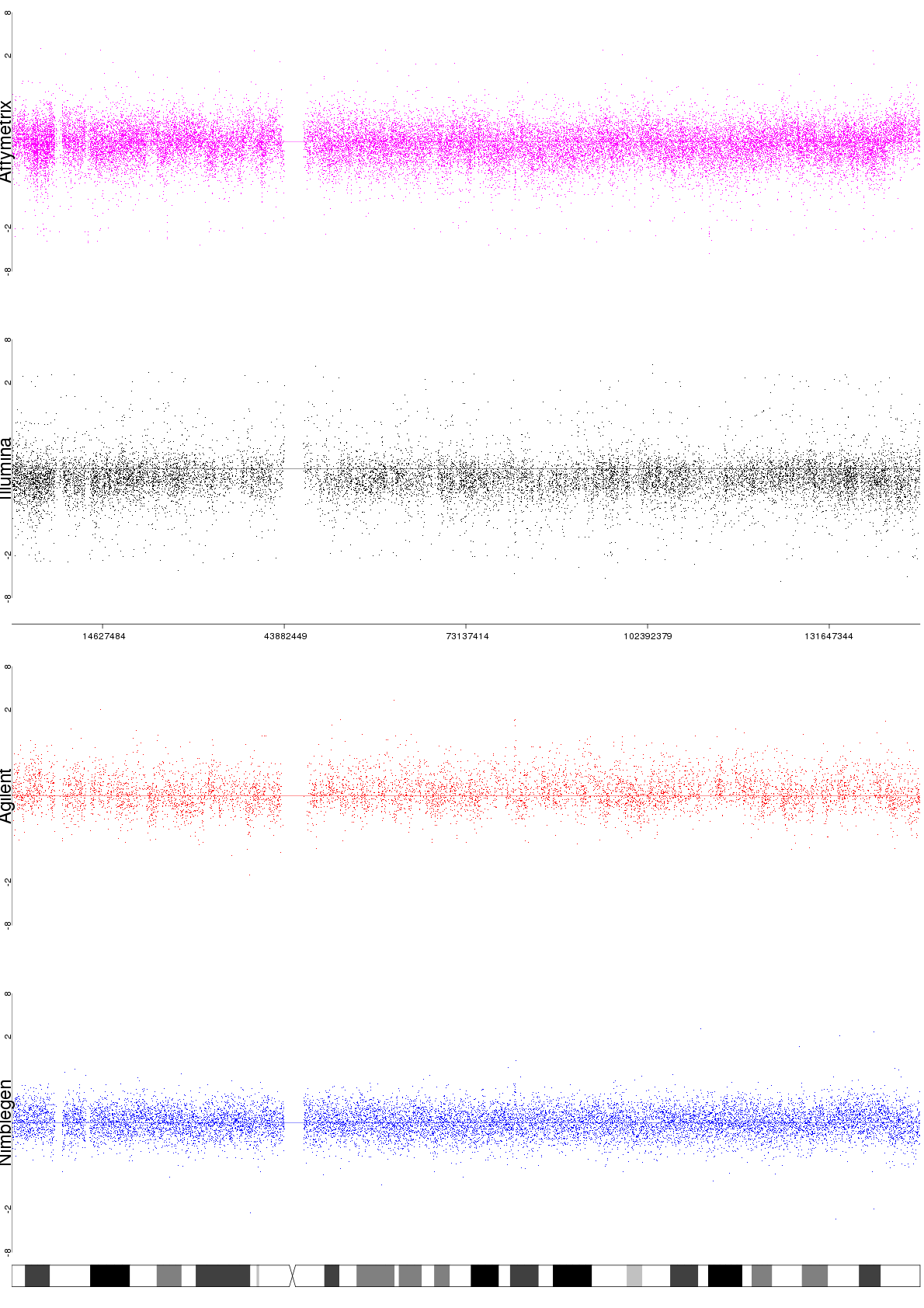

Supplement: Additional file 13 — All sample/chromosome plots for the cell-lines. Zip folder containing PNGs of all whole-chromosome plots for the cell-lines. [file 1471-2164-10-588-S13.ZIP › MT3/MT3 chromosome 8.png]

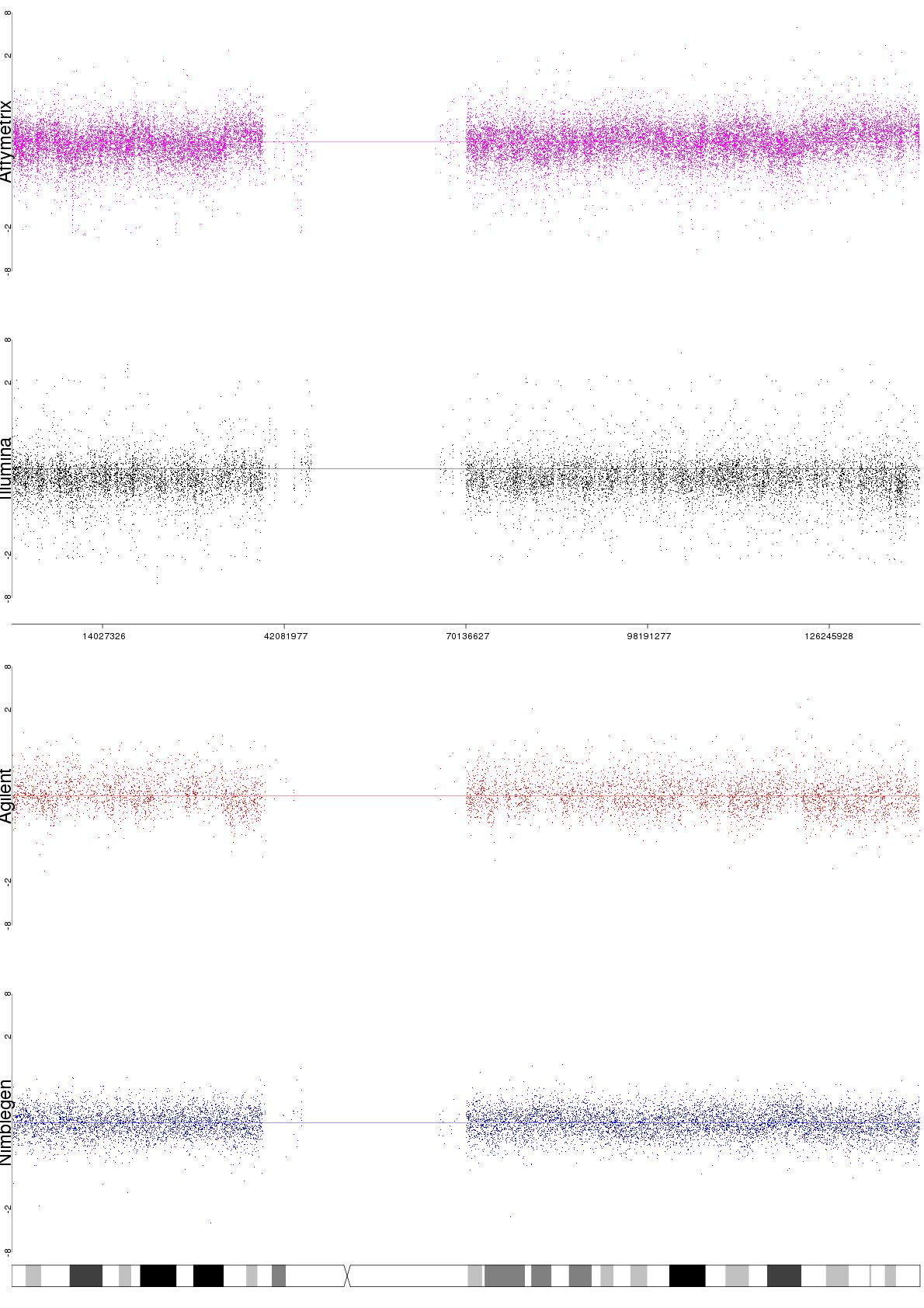

Supplement: Additional file 13 — All sample/chromosome plots for the cell-lines. Zip folder containing PNGs of all whole-chromosome plots for the cell-lines. [file 1471-2164-10-588-S13.ZIP › MT3/MT3 chromosome 9.png]

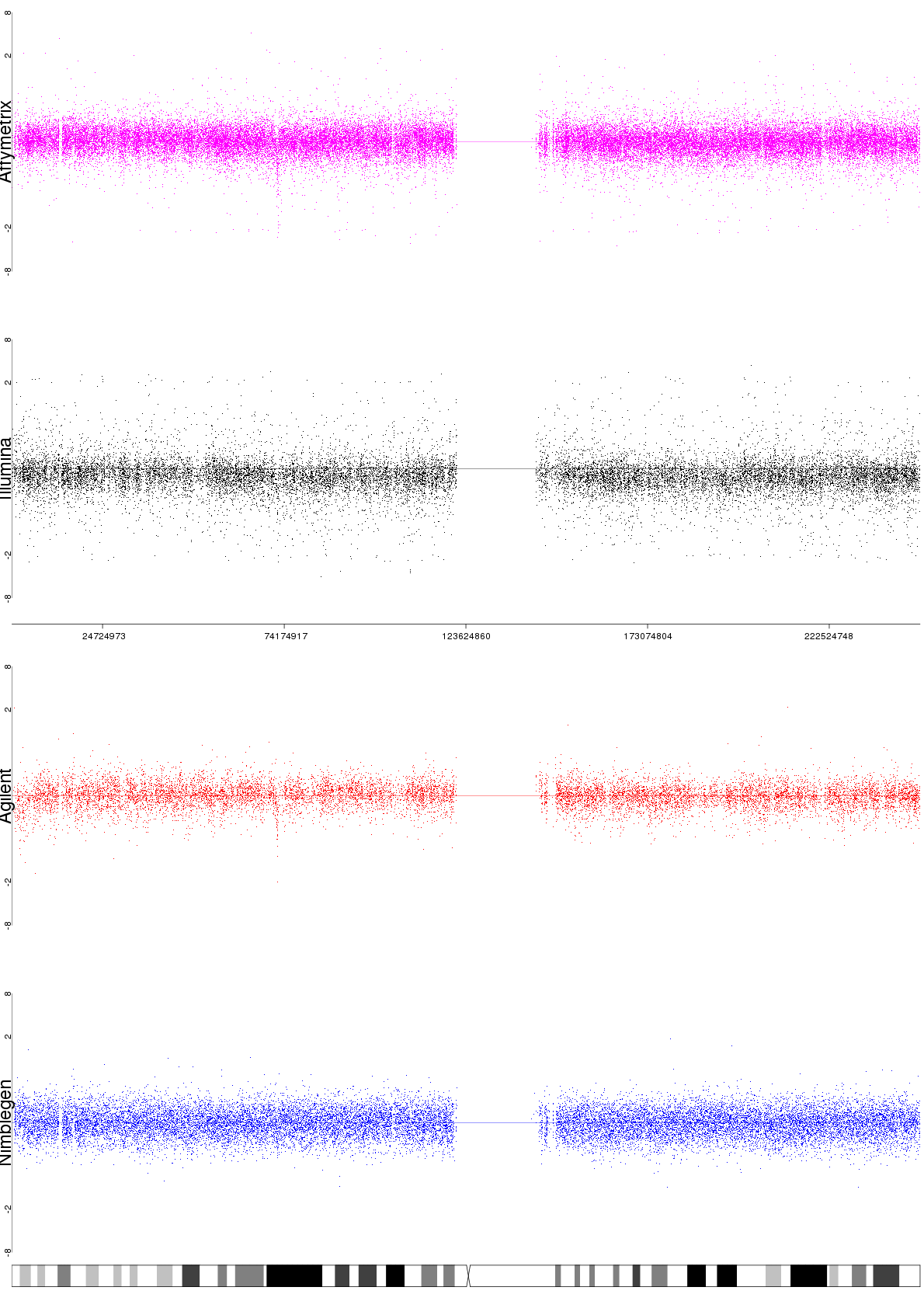

Supplement: Additional file 13 — All sample/chromosome plots for the cell-lines. Zip folder containing PNGs of all whole-chromosome plots for the cell-lines. [file 1471-2164-10-588-S13.ZIP › Sum159/SUM159 chromosome 1.png]

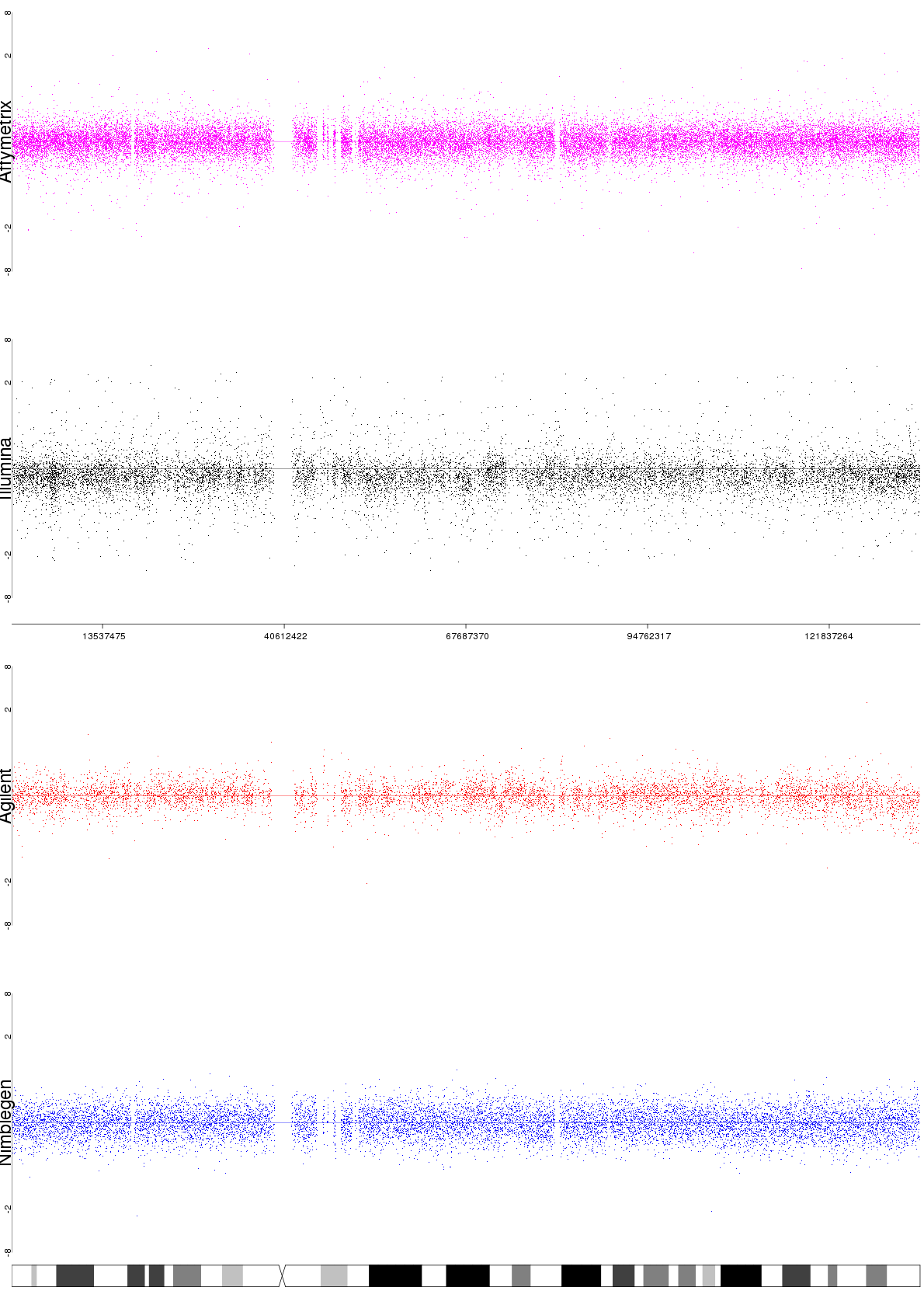

Supplement: Additional file 13 — All sample/chromosome plots for the cell-lines. Zip folder containing PNGs of all whole-chromosome plots for the cell-lines. [file 1471-2164-10-588-S13.ZIP › Sum159/SUM159 chromosome 10.png]

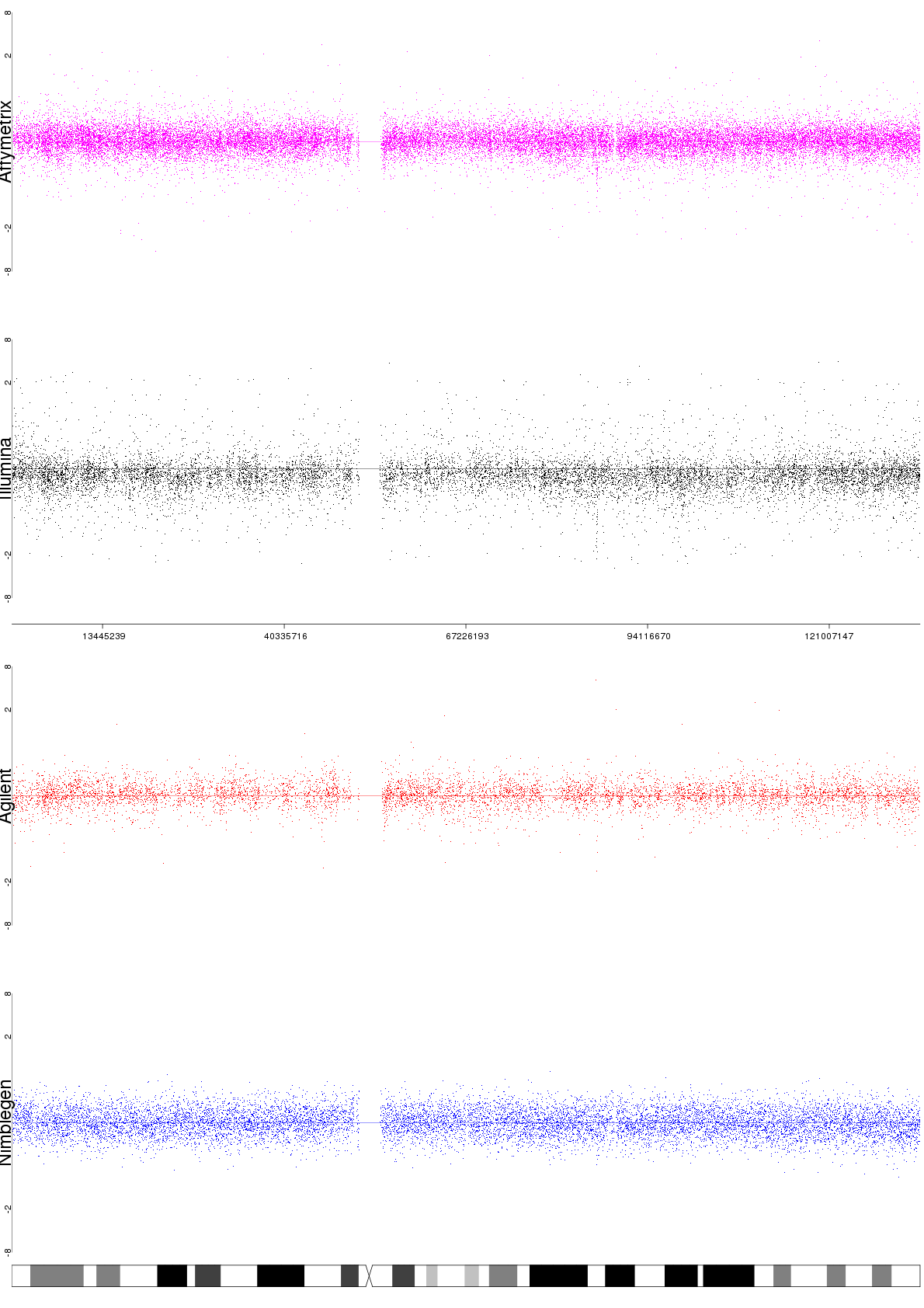

Supplement: Additional file 13 — All sample/chromosome plots for the cell-lines. Zip folder containing PNGs of all whole-chromosome plots for the cell-lines. [file 1471-2164-10-588-S13.ZIP › Sum159/SUM159 chromosome 11.png]

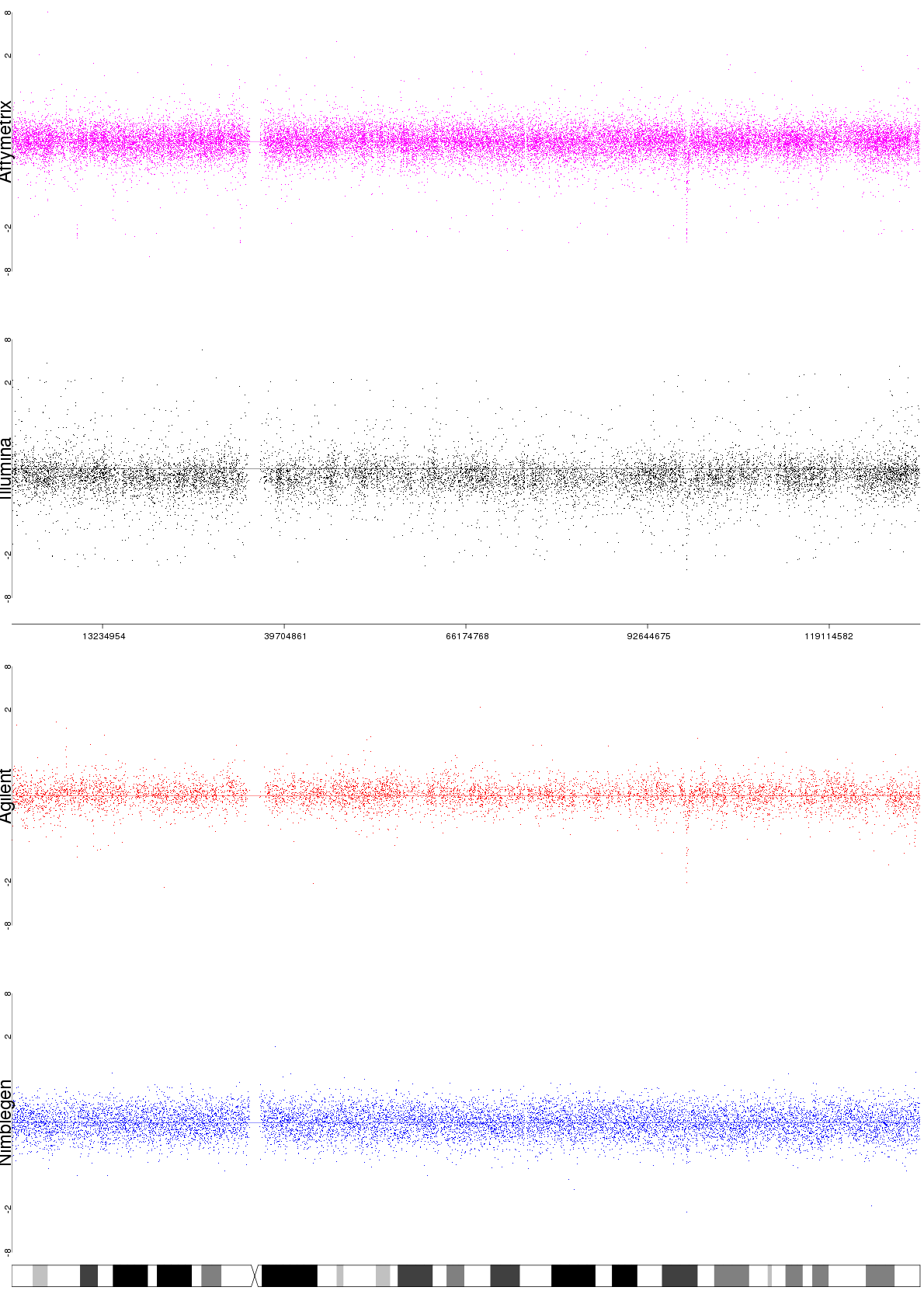

Supplement: Additional file 13 — All sample/chromosome plots for the cell-lines. Zip folder containing PNGs of all whole-chromosome plots for the cell-lines. [file 1471-2164-10-588-S13.ZIP › Sum159/SUM159 chromosome 12.png]

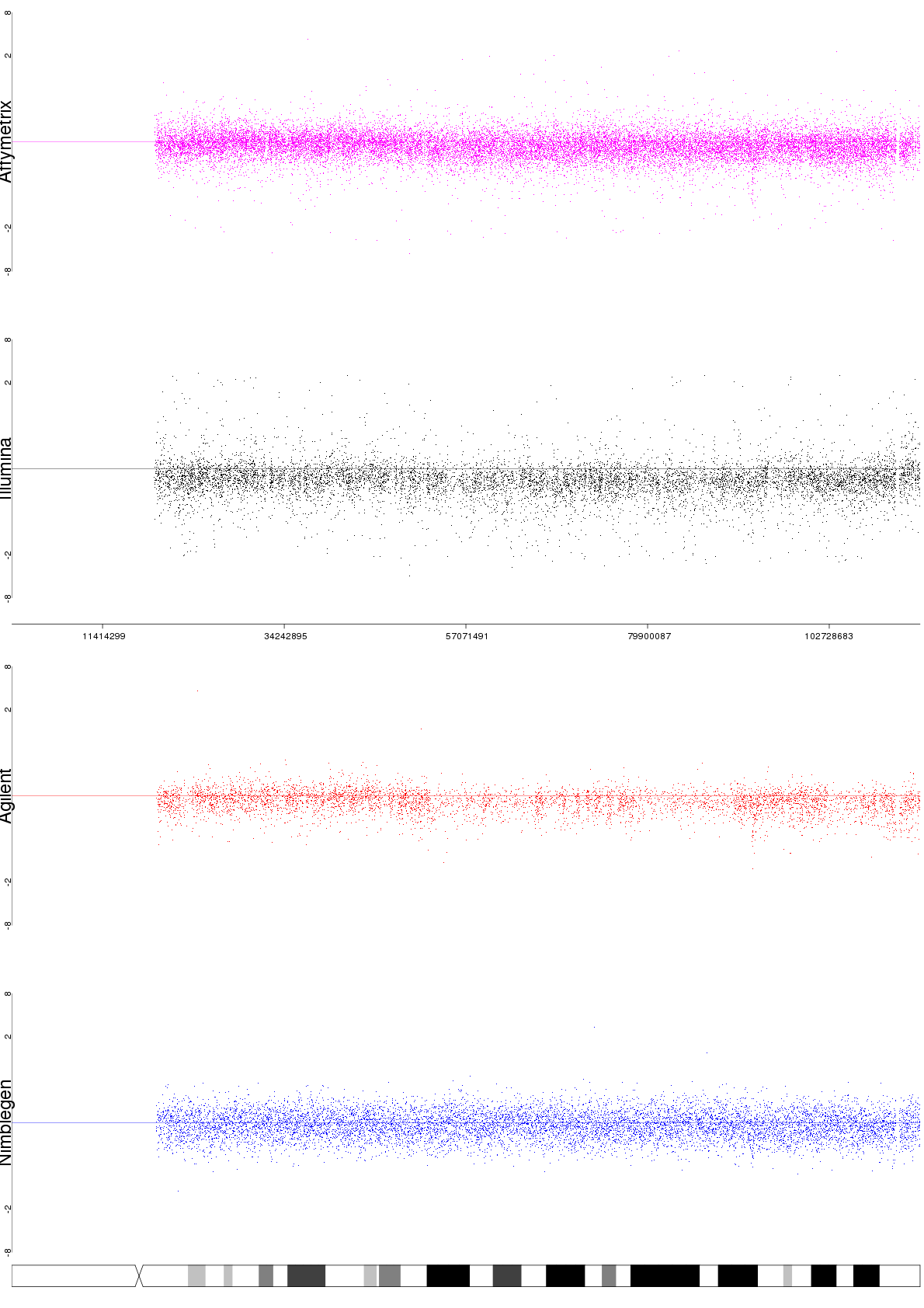

Supplement: Additional file 13 — All sample/chromosome plots for the cell-lines. Zip folder containing PNGs of all whole-chromosome plots for the cell-lines. [file 1471-2164-10-588-S13.ZIP › Sum159/SUM159 chromosome 13.png]
