# Supplementary figures and images for: The pitfalls of platform comparison: DNA copy number array technologies assessed (part 3 of 3)
Source: BMC Genomics. 2009 Dec 8;10:588. doi: 10.1186/1471-2164-10-588 (PMC2797821; doi:10.1186/1471-2164-10-588)

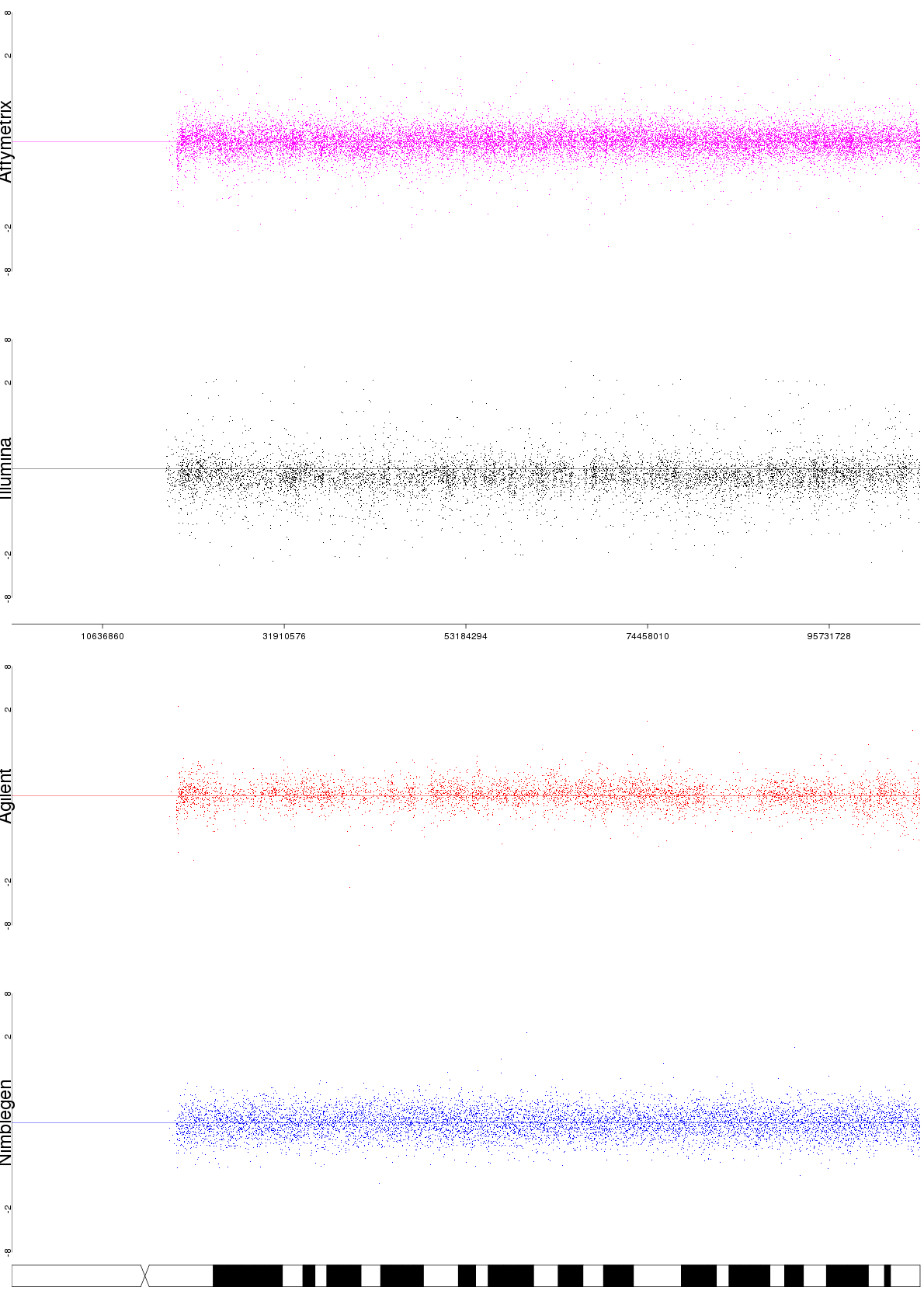

Supplement: Additional file 13 — All sample/chromosome plots for the cell-lines. Zip folder containing PNGs of all whole-chromosome plots for the cell-lines. [file 1471-2164-10-588-S13.ZIP › Sum159/SUM159 chromosome 14.png]

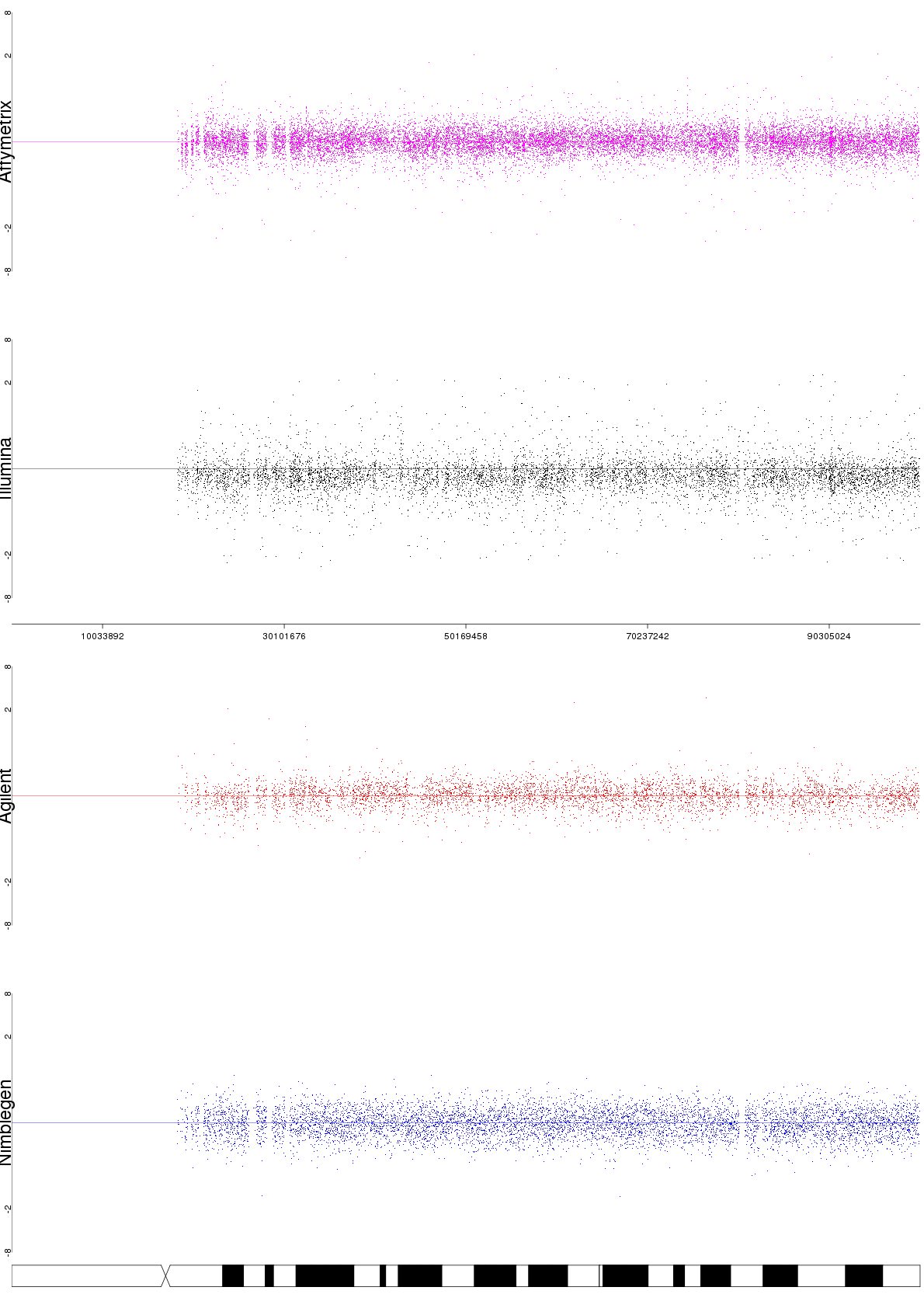

Supplement: Additional file 13 — All sample/chromosome plots for the cell-lines. Zip folder containing PNGs of all whole-chromosome plots for the cell-lines. [file 1471-2164-10-588-S13.ZIP › Sum159/SUM159 chromosome 15.png]

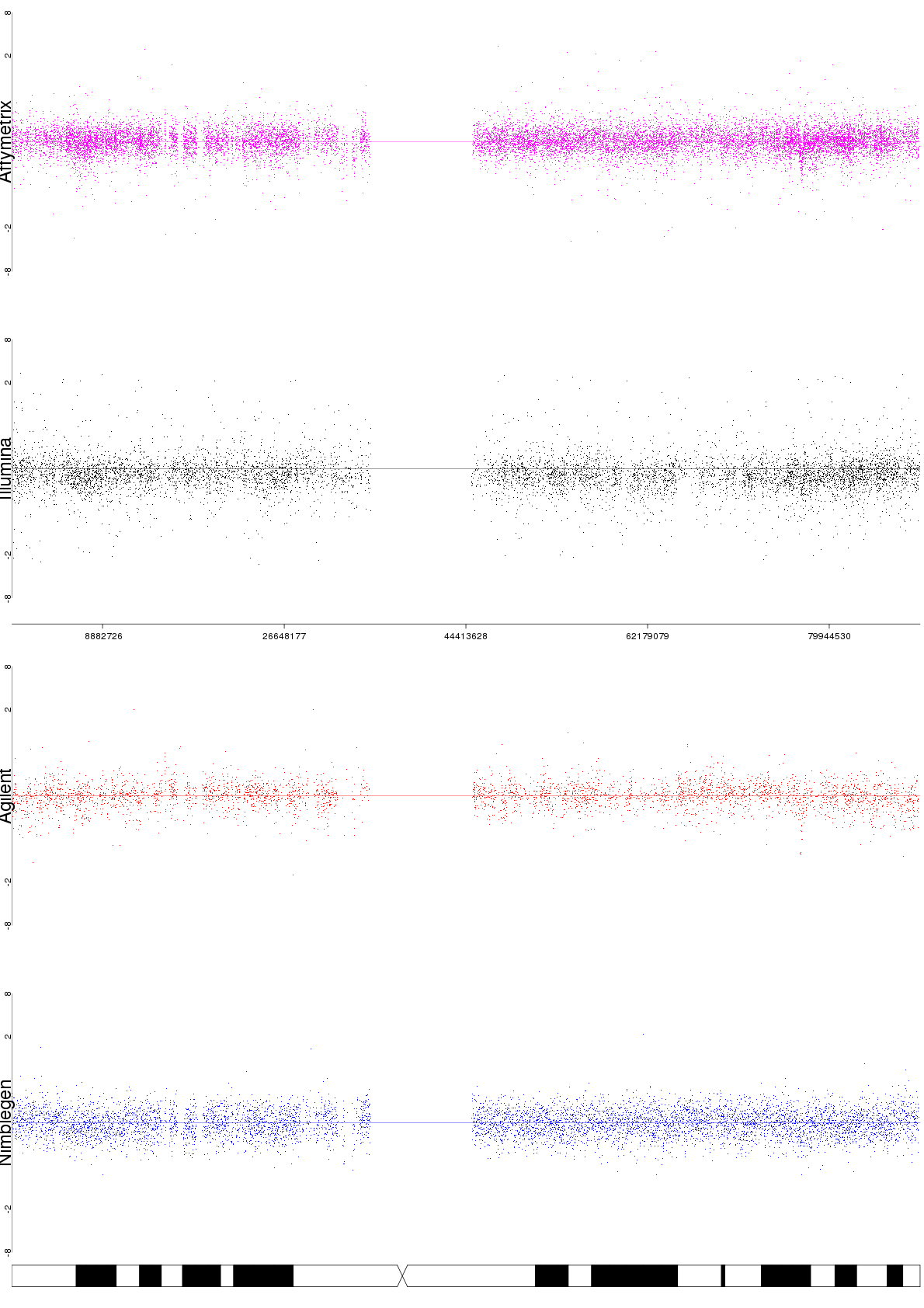

Supplement: Additional file 13 — All sample/chromosome plots for the cell-lines. Zip folder containing PNGs of all whole-chromosome plots for the cell-lines. [file 1471-2164-10-588-S13.ZIP › Sum159/SUM159 chromosome 16.png]

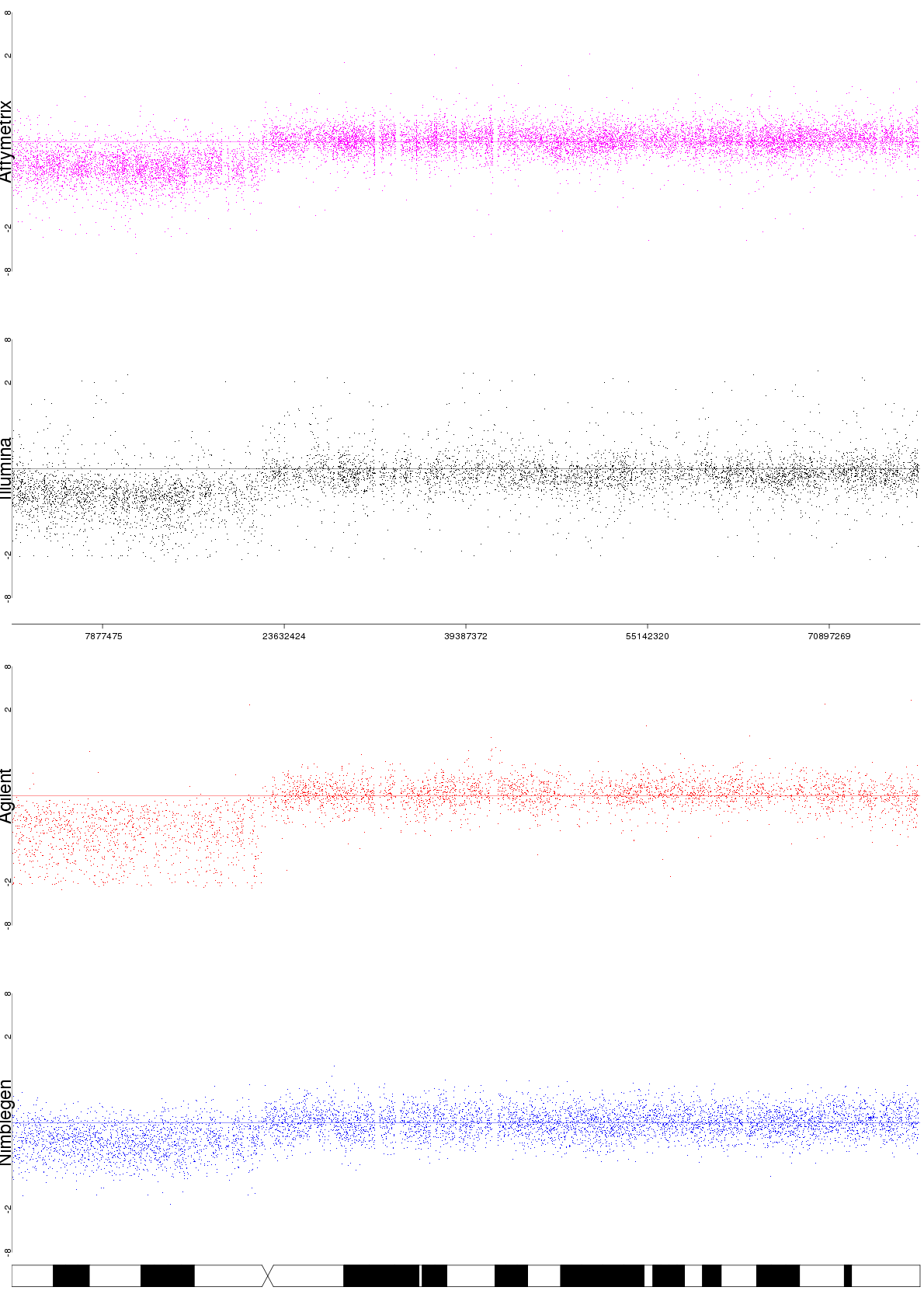

Supplement: Additional file 13 — All sample/chromosome plots for the cell-lines. Zip folder containing PNGs of all whole-chromosome plots for the cell-lines. [file 1471-2164-10-588-S13.ZIP › Sum159/SUM159 chromosome 17.png]

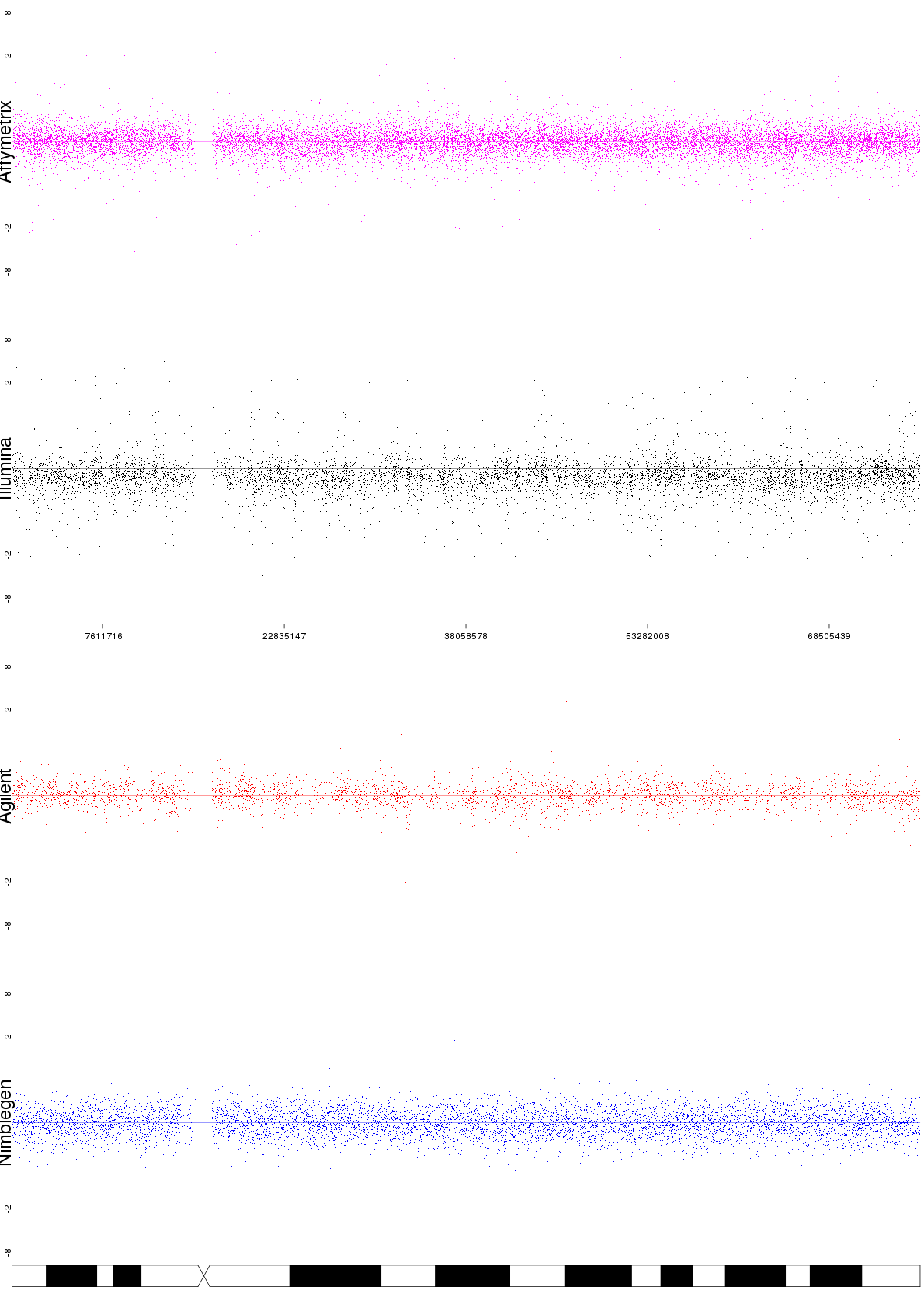

Supplement: Additional file 13 — All sample/chromosome plots for the cell-lines. Zip folder containing PNGs of all whole-chromosome plots for the cell-lines. [file 1471-2164-10-588-S13.ZIP › Sum159/SUM159 chromosome 18.png]

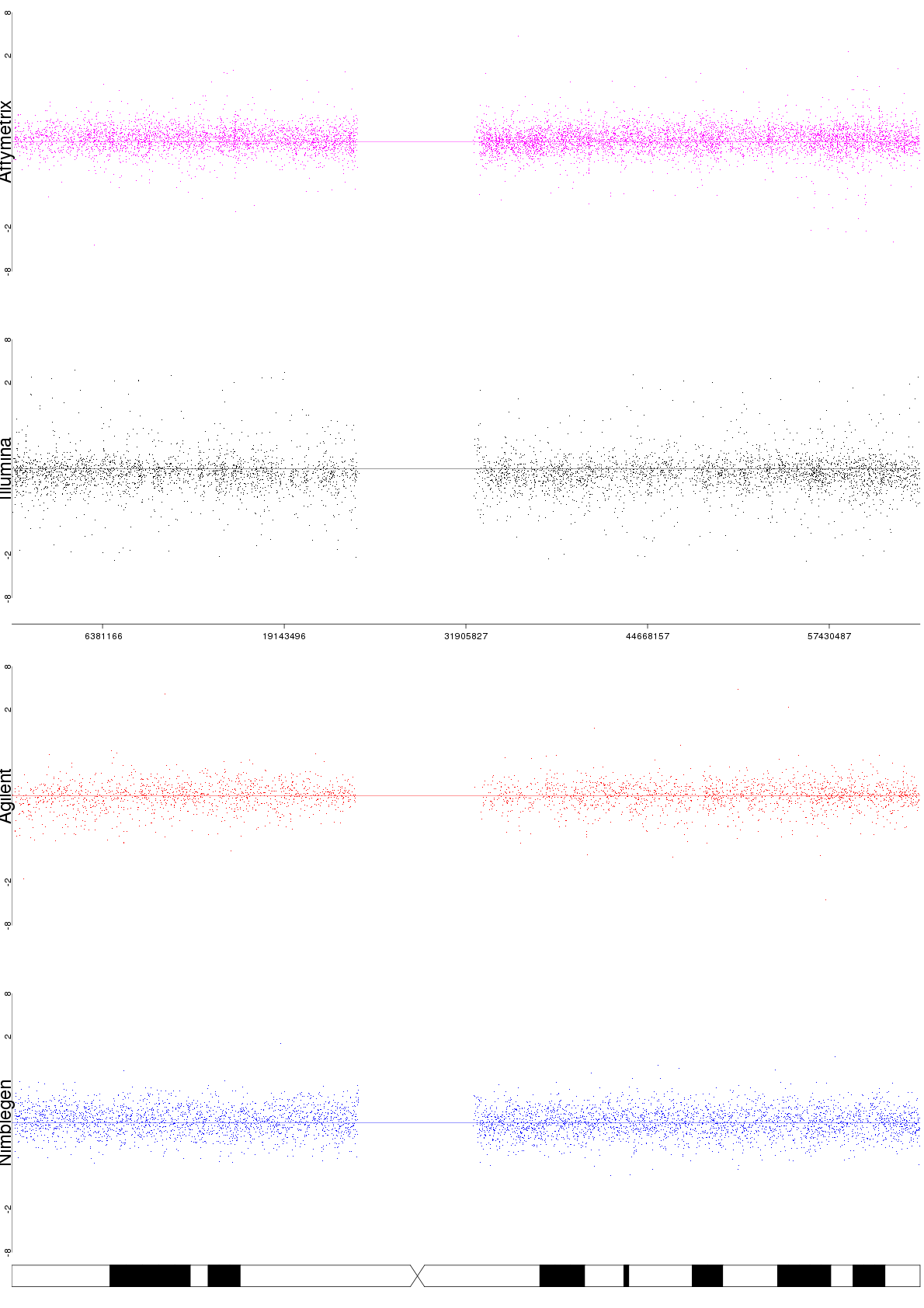

Supplement: Additional file 13 — All sample/chromosome plots for the cell-lines. Zip folder containing PNGs of all whole-chromosome plots for the cell-lines. [file 1471-2164-10-588-S13.ZIP › Sum159/SUM159 chromosome 19.png]

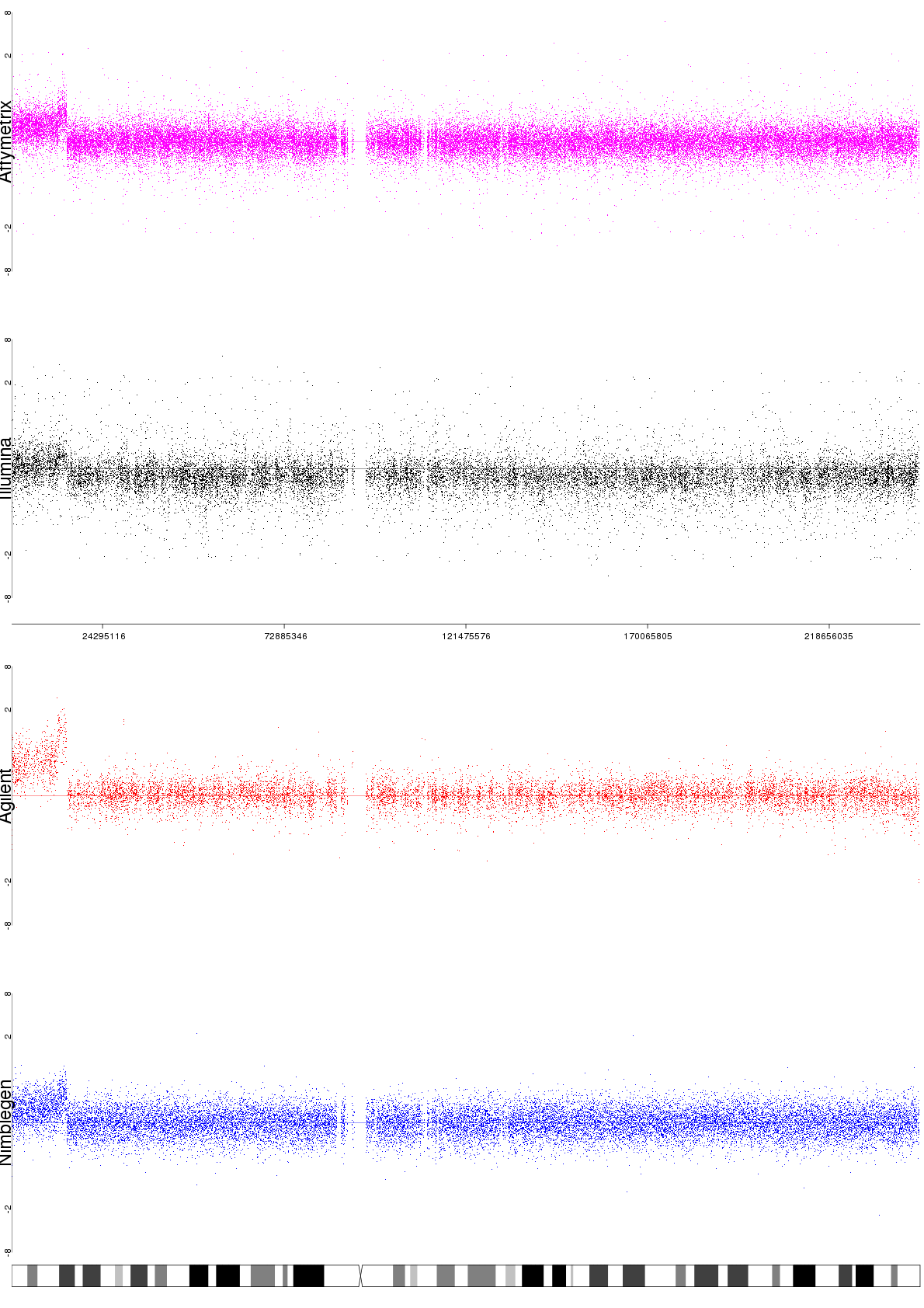

Supplement: Additional file 13 — All sample/chromosome plots for the cell-lines. Zip folder containing PNGs of all whole-chromosome plots for the cell-lines. [file 1471-2164-10-588-S13.ZIP › Sum159/SUM159 chromosome 2.png]

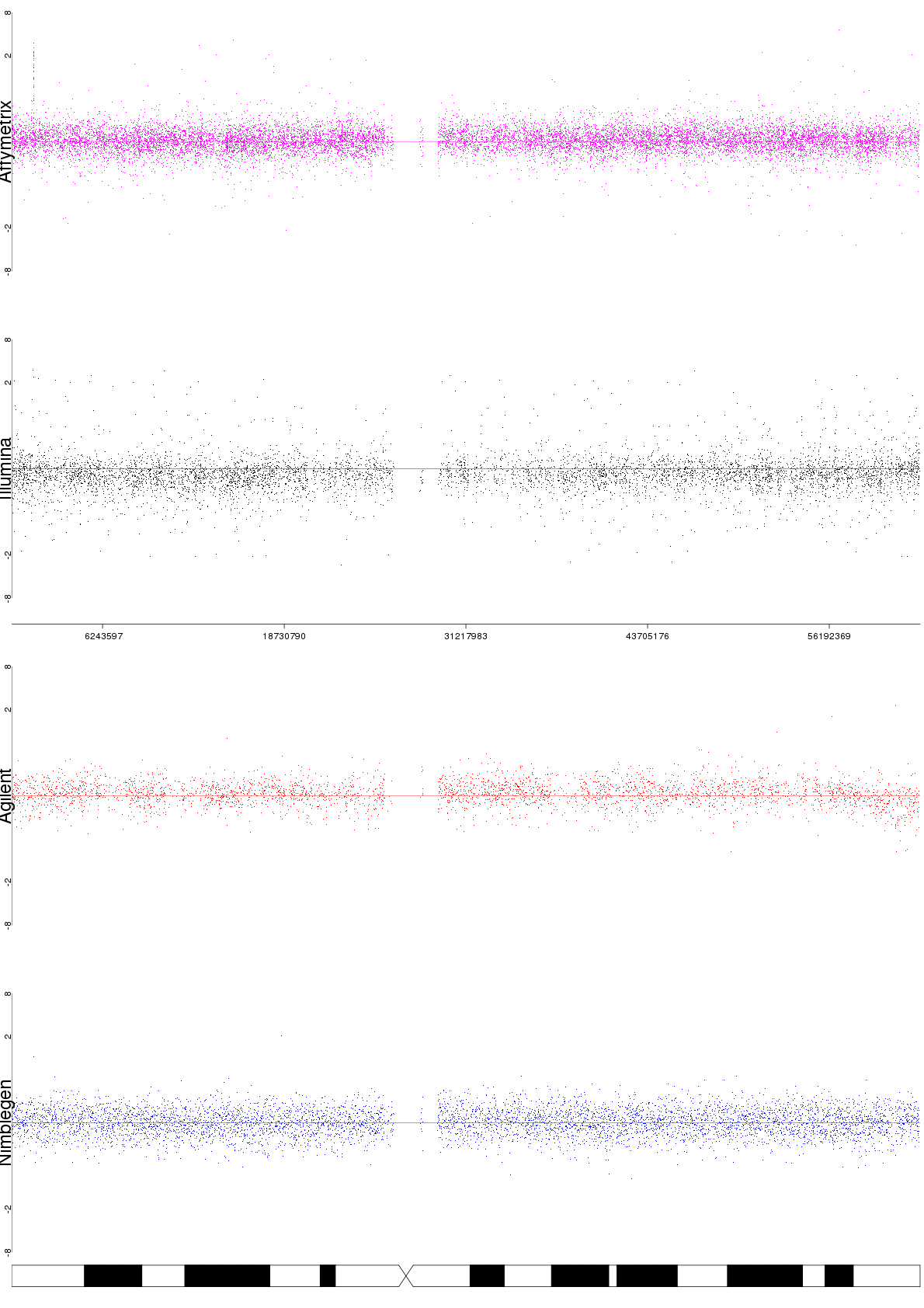

Supplement: Additional file 13 — All sample/chromosome plots for the cell-lines. Zip folder containing PNGs of all whole-chromosome plots for the cell-lines. [file 1471-2164-10-588-S13.ZIP › Sum159/SUM159 chromosome 20.png]

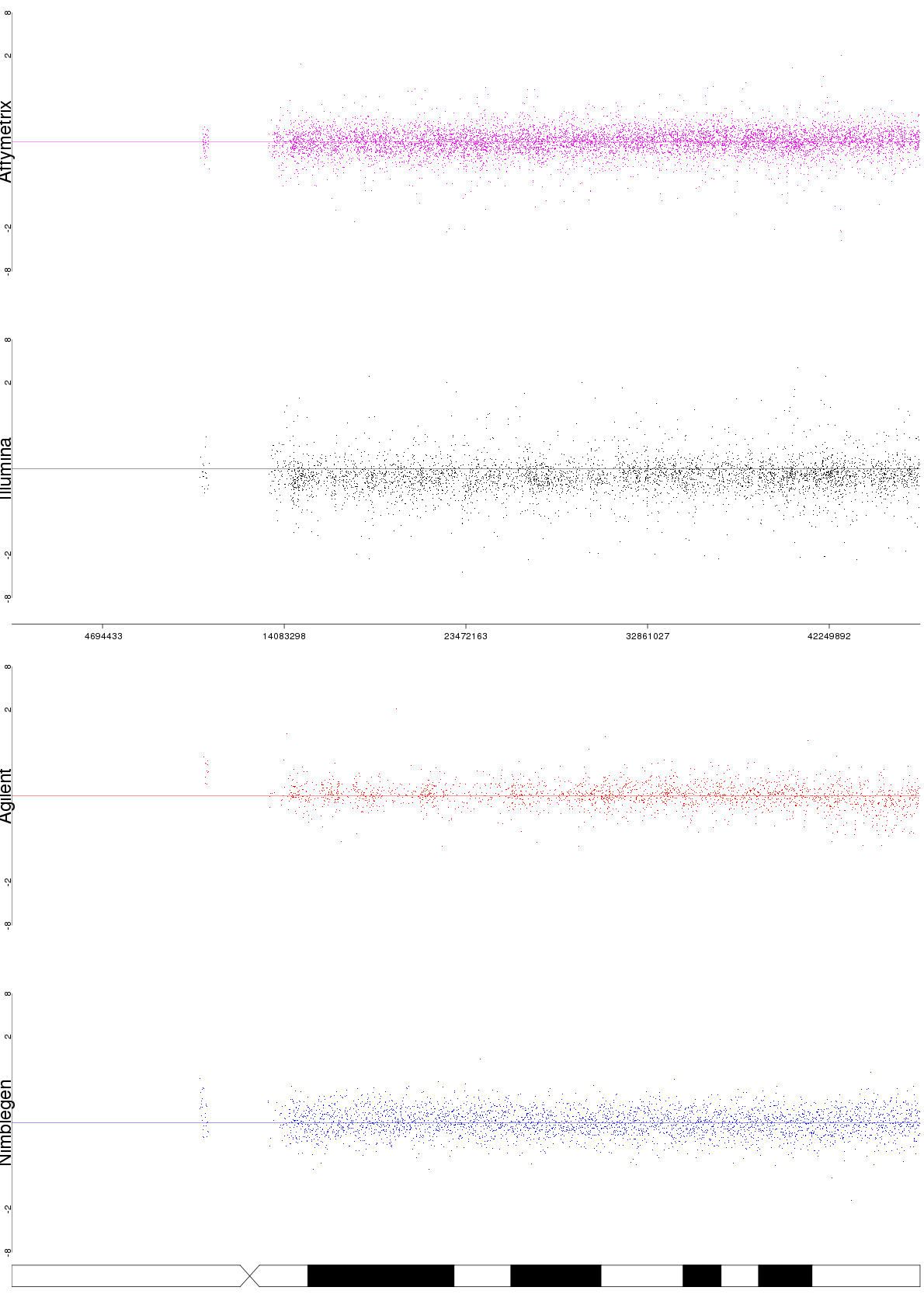

Supplement: Additional file 13 — All sample/chromosome plots for the cell-lines. Zip folder containing PNGs of all whole-chromosome plots for the cell-lines. [file 1471-2164-10-588-S13.ZIP › Sum159/SUM159 chromosome 21.png]

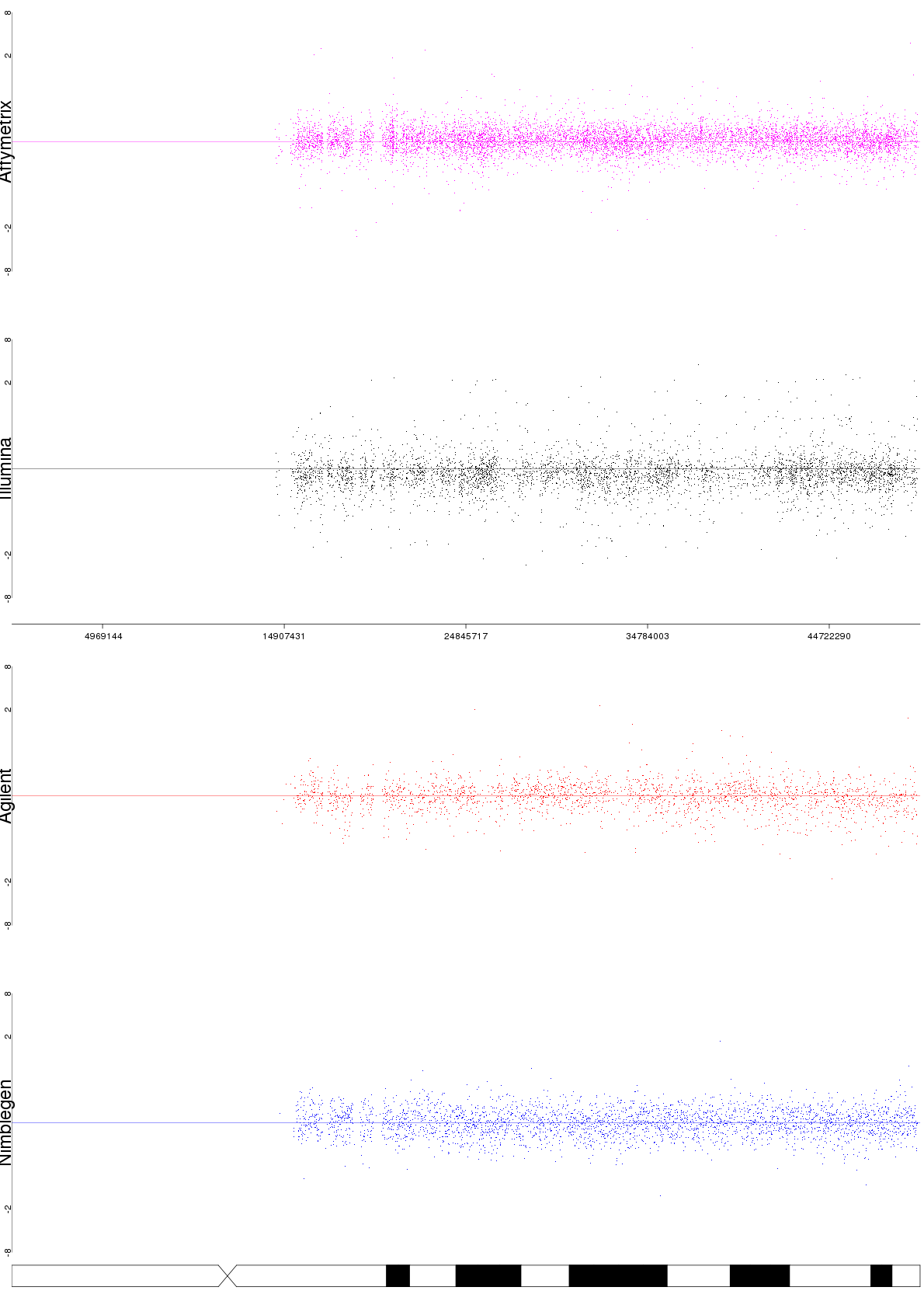

Supplement: Additional file 13 — All sample/chromosome plots for the cell-lines. Zip folder containing PNGs of all whole-chromosome plots for the cell-lines. [file 1471-2164-10-588-S13.ZIP › Sum159/SUM159 chromosome 22.png]

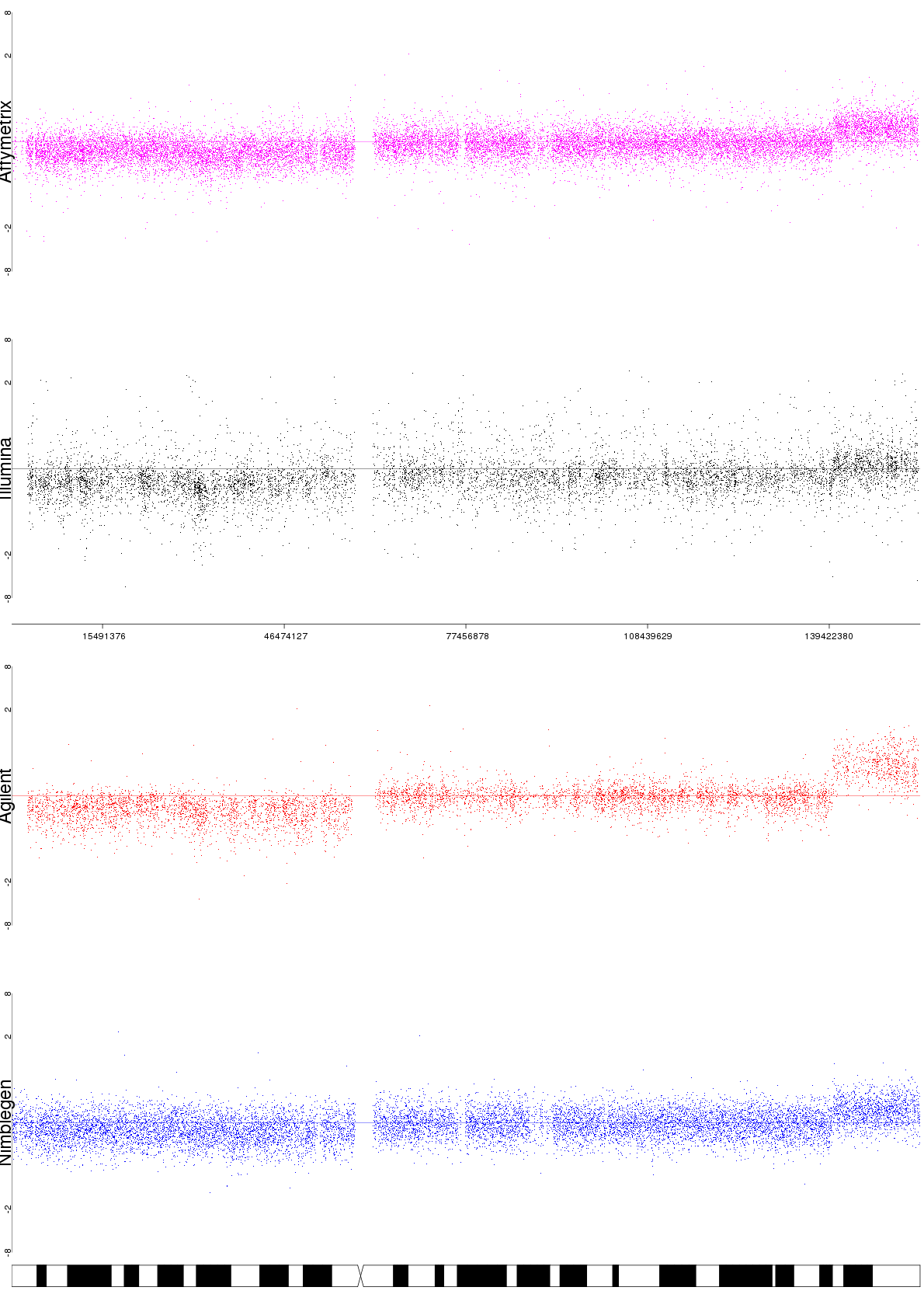

Supplement: Additional file 13 — All sample/chromosome plots for the cell-lines. Zip folder containing PNGs of all whole-chromosome plots for the cell-lines. [file 1471-2164-10-588-S13.ZIP › Sum159/SUM159 chromosome 23.png]

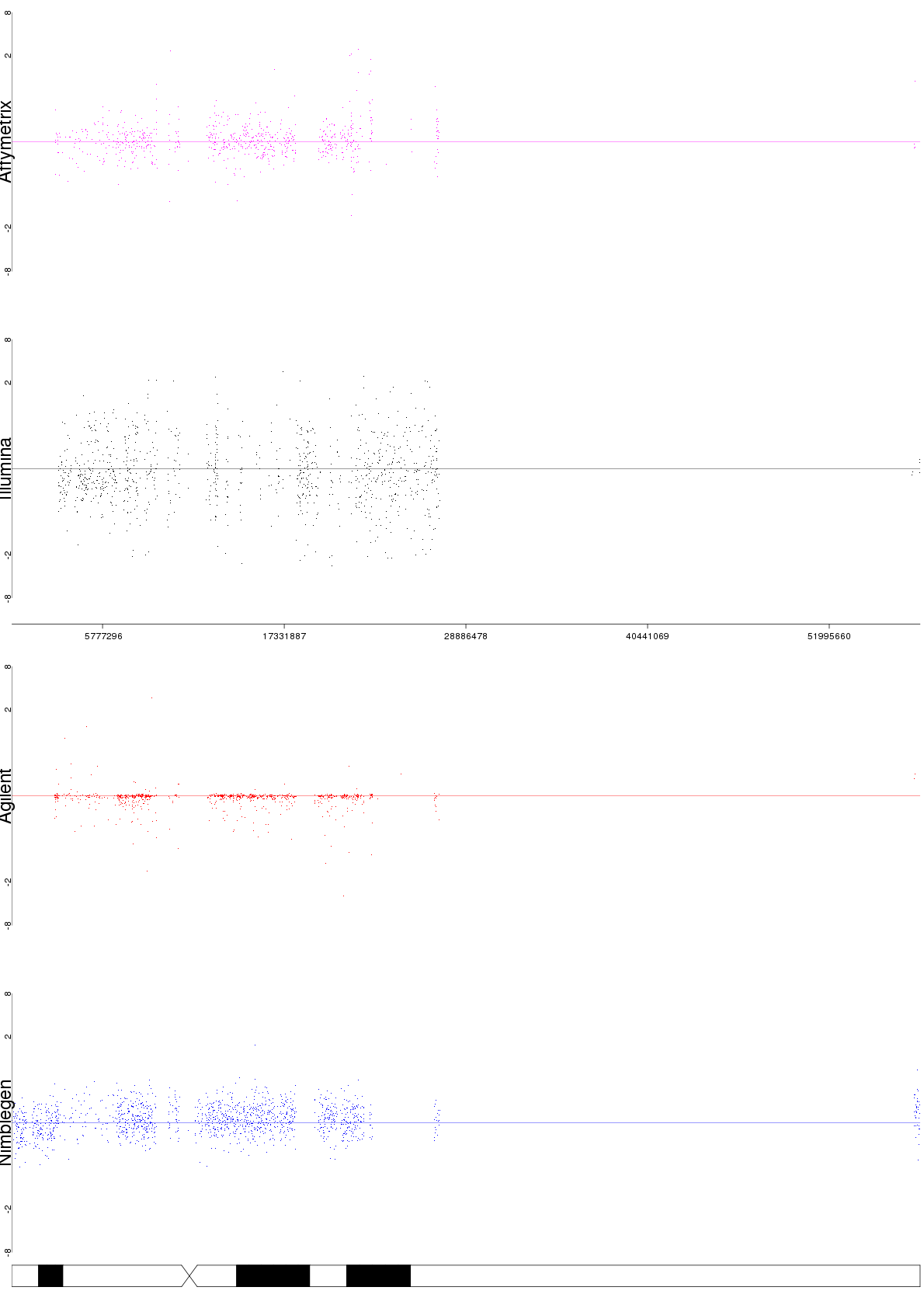

Supplement: Additional file 13 — All sample/chromosome plots for the cell-lines. Zip folder containing PNGs of all whole-chromosome plots for the cell-lines. [file 1471-2164-10-588-S13.ZIP › Sum159/SUM159 chromosome 24.png]

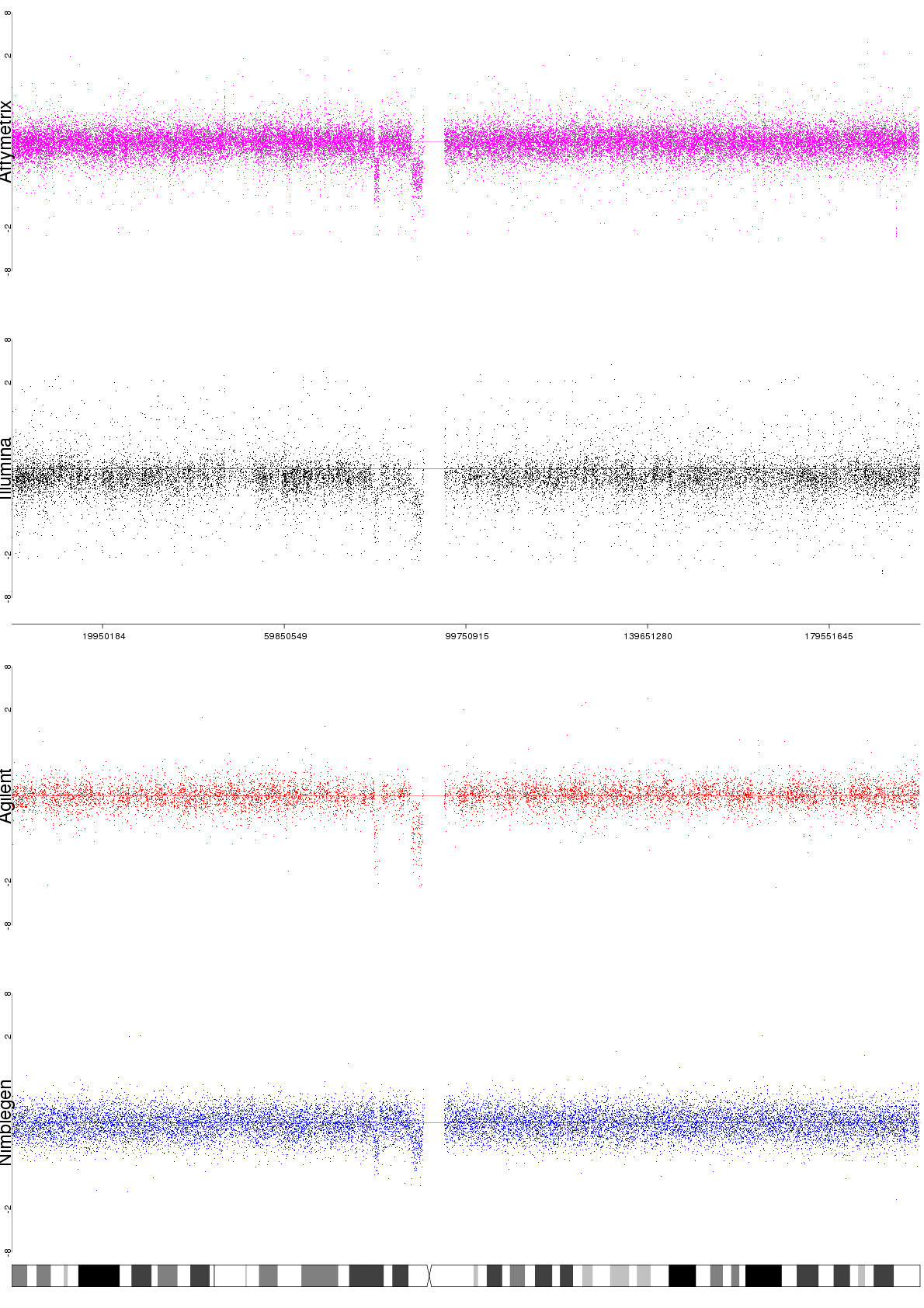

Supplement: Additional file 13 — All sample/chromosome plots for the cell-lines. Zip folder containing PNGs of all whole-chromosome plots for the cell-lines. [file 1471-2164-10-588-S13.ZIP › Sum159/SUM159 chromosome 3.png]

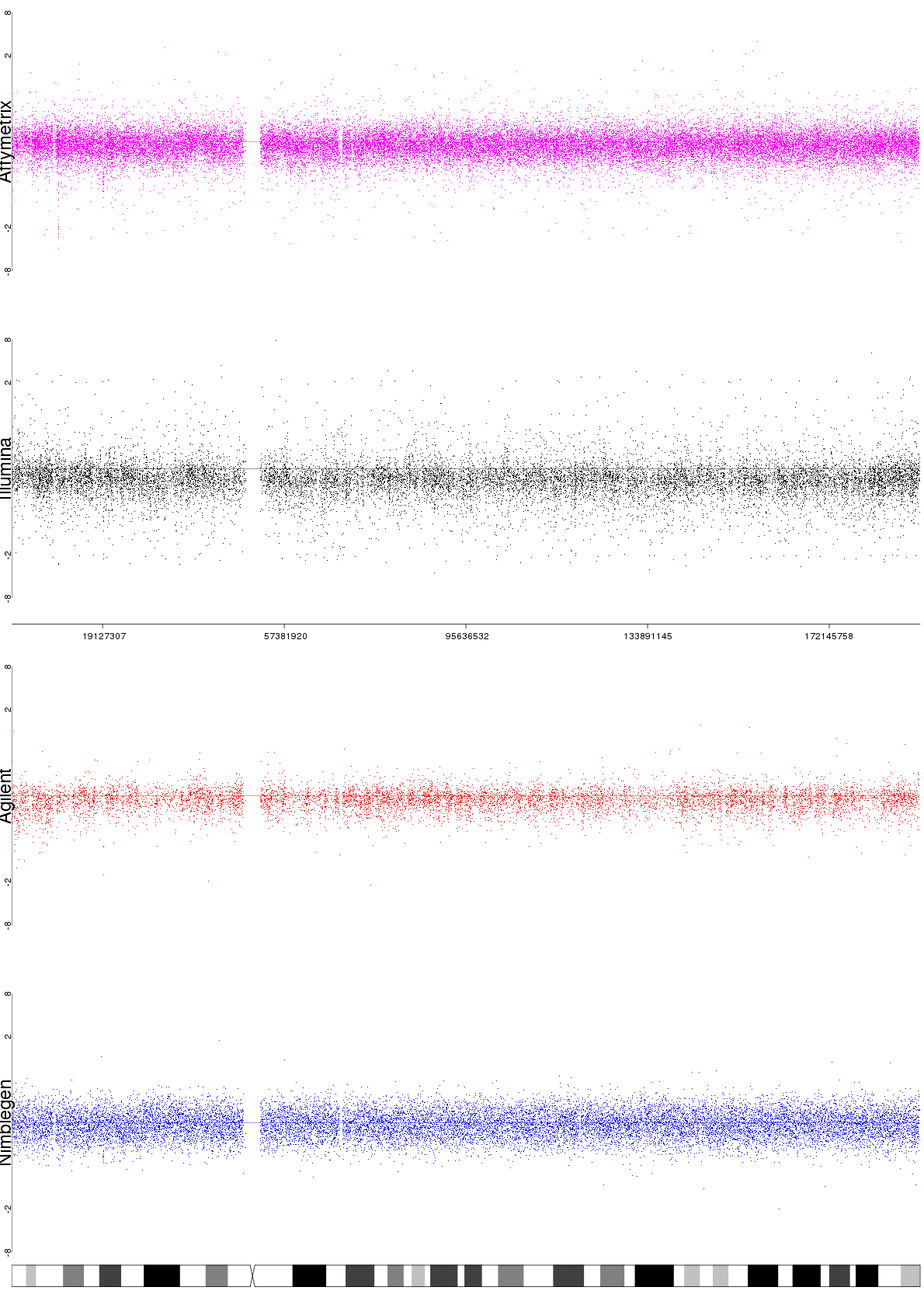

Supplement: Additional file 13 — All sample/chromosome plots for the cell-lines. Zip folder containing PNGs of all whole-chromosome plots for the cell-lines. [file 1471-2164-10-588-S13.ZIP › Sum159/SUM159 chromosome 4.png]

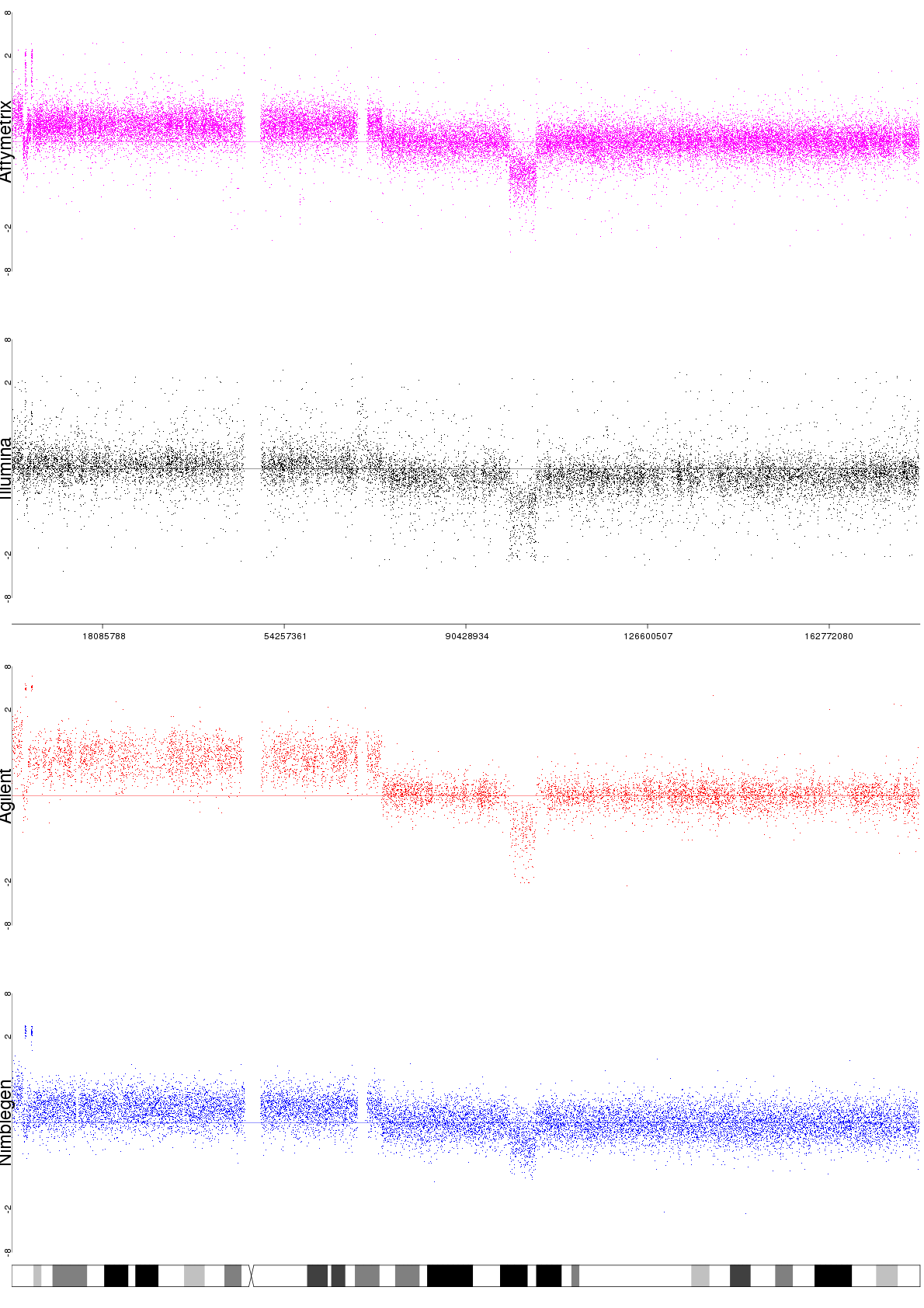

Supplement: Additional file 13 — All sample/chromosome plots for the cell-lines. Zip folder containing PNGs of all whole-chromosome plots for the cell-lines. [file 1471-2164-10-588-S13.ZIP › Sum159/SUM159 chromosome 5.png]

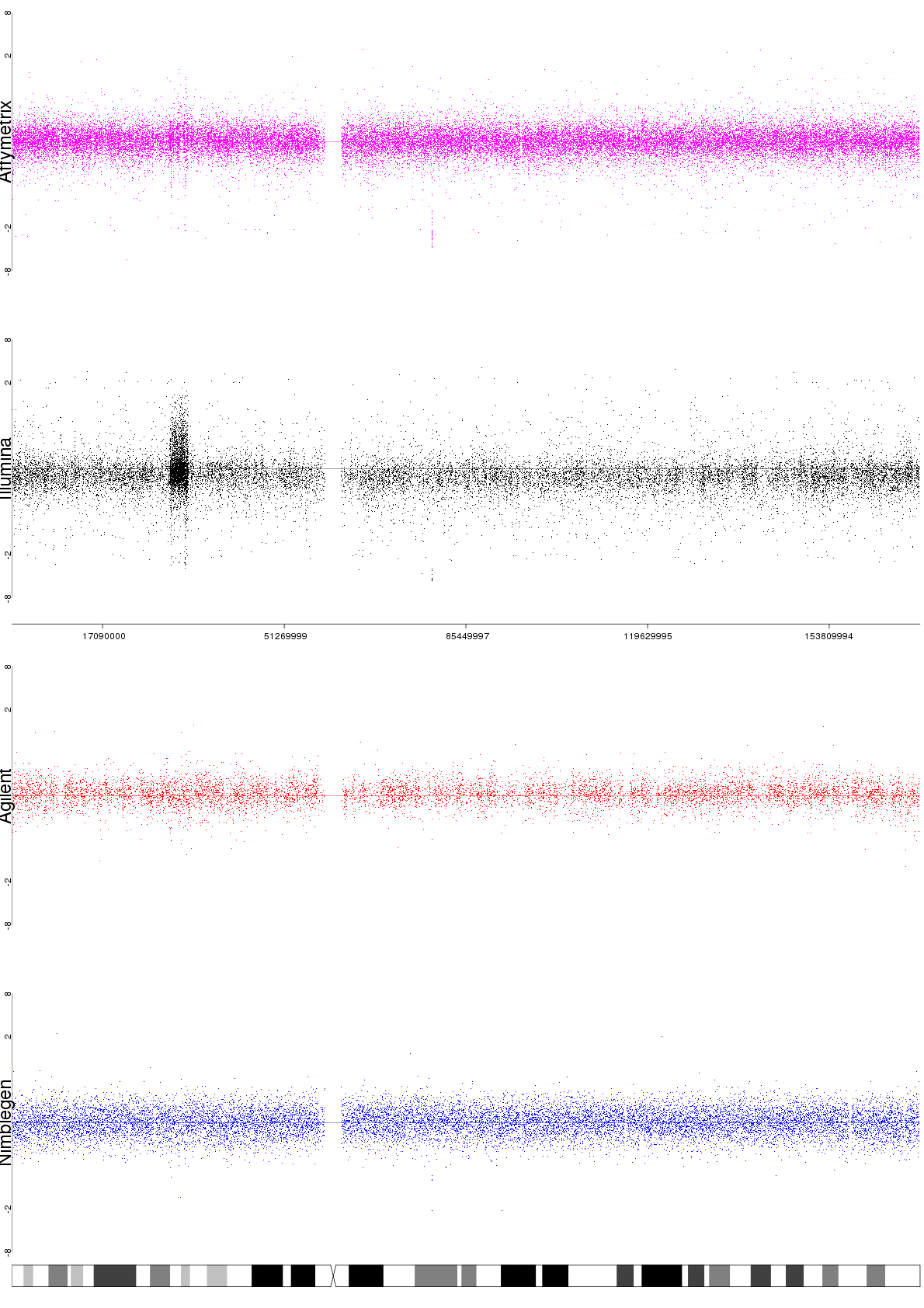

Supplement: Additional file 13 — All sample/chromosome plots for the cell-lines. Zip folder containing PNGs of all whole-chromosome plots for the cell-lines. [file 1471-2164-10-588-S13.ZIP › Sum159/SUM159 chromosome 6.png]

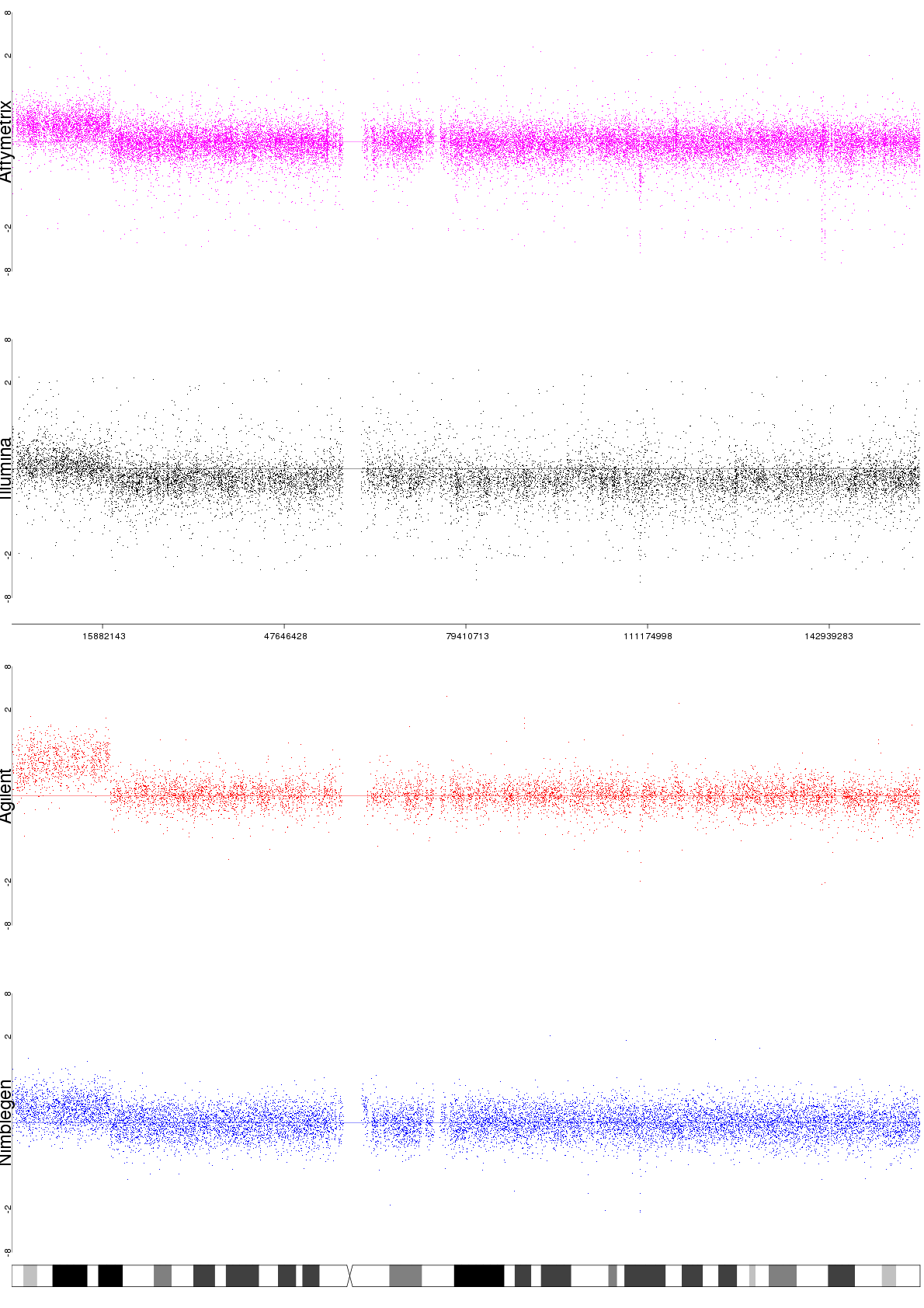

Supplement: Additional file 13 — All sample/chromosome plots for the cell-lines. Zip folder containing PNGs of all whole-chromosome plots for the cell-lines. [file 1471-2164-10-588-S13.ZIP › Sum159/SUM159 chromosome 7.png]

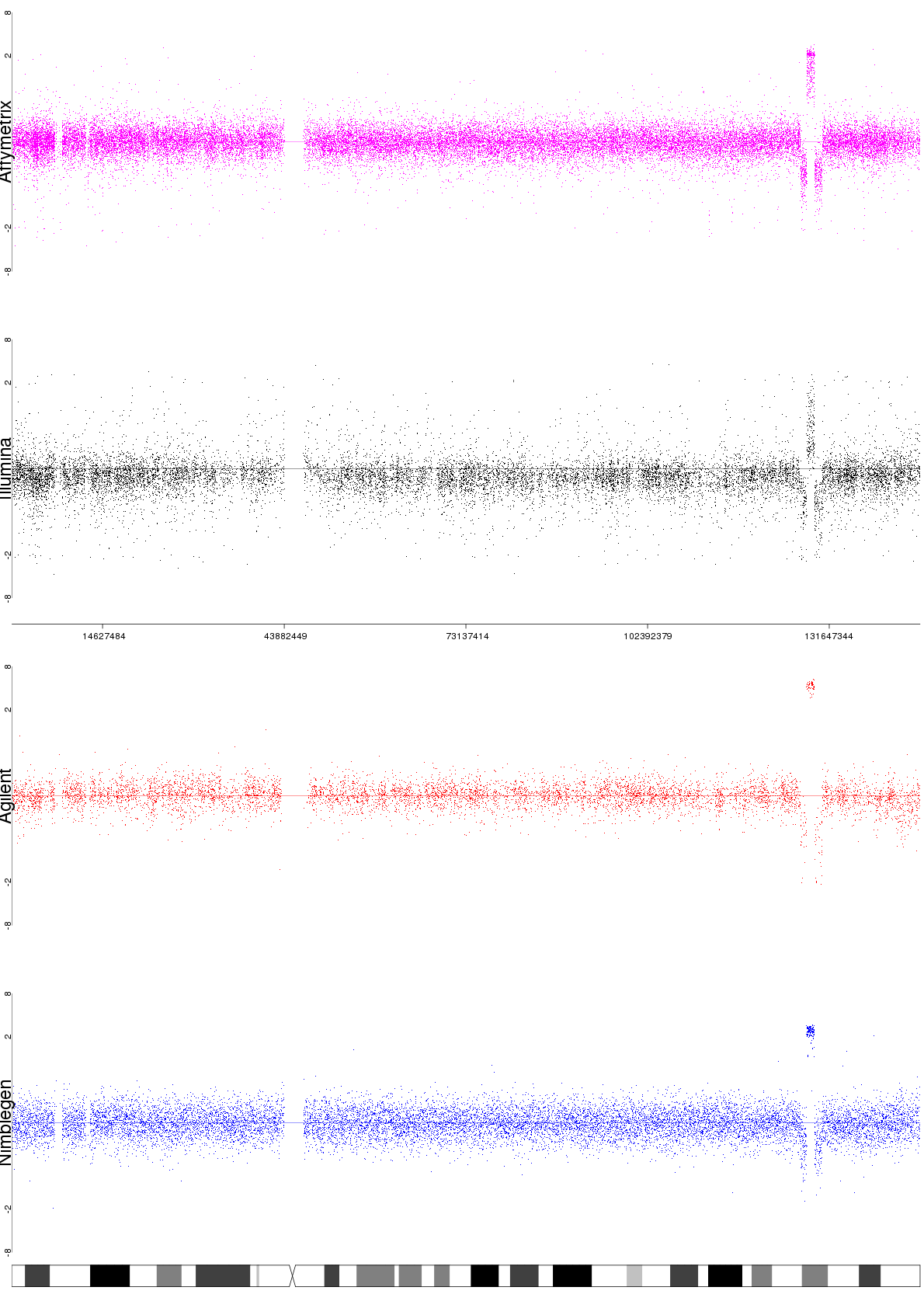

Supplement: Additional file 13 — All sample/chromosome plots for the cell-lines. Zip folder containing PNGs of all whole-chromosome plots for the cell-lines. [file 1471-2164-10-588-S13.ZIP › Sum159/SUM159 chromosome 8.png]

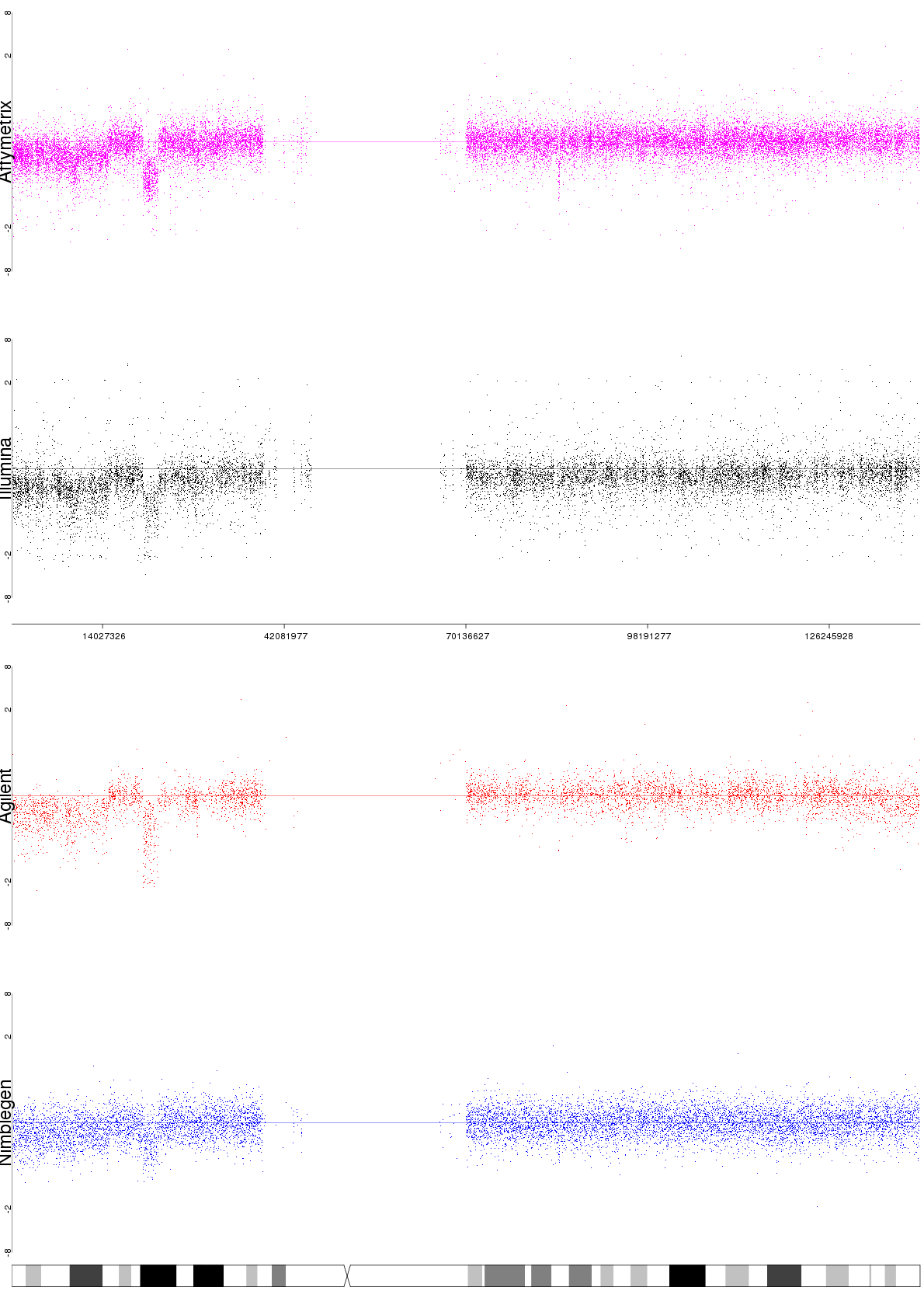

Supplement: Additional file 13 — All sample/chromosome plots for the cell-lines. Zip folder containing PNGs of all whole-chromosome plots for the cell-lines. [file 1471-2164-10-588-S13.ZIP › Sum159/SUM159 chromosome 9.png]
